# Machine Learning Classification of Time since BNT162b2 COVID-19 Vaccination Based on Array-Measured Antibody Activity

**DOI:** 10.3390/life13061304

**Published:** 2023-05-31

**Authors:** Qing-Lan Ma, Fei-Ming Huang, Wei Guo, Kai-Yan Feng, Tao Huang, Yu-Dong Cai

**Affiliations:** 1 School of Life Sciences, Shanghai University, Shanghai 200444, China; mql1117@shu.edu.cn (Q.-L.M.); hfm123@shu.edu.cn (F.-M.H.); 2 Key Laboratory of Stem Cell Biology, Shanghai Jiao Tong University School of Medicine (SJTUSM) & Shanghai Institutes for Biological Sciences (SIBS), Chinese Academy of Sciences (CAS), Shanghai 200030, China; gw_1992@sjtu.edu.cn; 3 Department of Computer Science, Guangdong AIB Polytechnic College, Guangzhou 510507, China; kyfeng@gdaib.edu.cn; 4 Bio-Med Big Data Center, CAS Key Laboratory of Computational Biology, Shanghai Institute of Nutrition and Health, University of Chinese Academy of Sciences, Chinese Academy of Sciences, Shanghai 200031, China; 5 CAS Key Laboratory of Tissue Microenvironment and Tumor, Shanghai Institute of Nutrition and Health, University of Chinese Academy of Sciences, Chinese Academy of Sciences, Shanghai 200031, China

**Keywords:** antigen, COVID-19 vaccination, machine learning

## Abstract

Vaccines trigger an immunological response that includes B and T cells, with B cells producing antibodies. SARS-CoV-2 immunity weakens over time after vaccination. Discovering key changes in antigen-reactive antibodies over time after vaccination could help improve vaccine efficiency. In this study, we collected data on blood antibody levels in a cohort of healthcare workers vaccinated for COVID-19 and obtained 73 antigens in samples from four groups according to the duration after vaccination, including 104 unvaccinated healthcare workers, 534 healthcare workers within 60 days after vaccination, 594 healthcare workers between 60 and 180 days after vaccination, and 141 healthcare workers over 180 days after vaccination. Our work was a reanalysis of the data originally collected at Irvine University. This data was obtained in Orange County, California, USA, with the collection process commencing in December 2020. British variant (B.1.1.7), South African variant (B.1.351), and Brazilian/Japanese variant (P.1) were the most prevalent strains during the sampling period. An efficient machine learning based framework containing four feature selection methods (least absolute shrinkage and selection operator, light gradient boosting machine, Monte Carlo feature selection, and maximum relevance minimum redundancy) and four classification algorithms (decision tree, k-nearest neighbor, random forest, and support vector machine) was designed to select essential antibodies against specific antigens. Several efficient classifiers with a weighted F1 value around 0.75 were constructed. The antigen microarray used for identifying antibody levels in the coronavirus features ten distinct SARS-CoV-2 antigens, comprising various segments of both nucleocapsid protein (NP) and spike protein (S). This study revealed that S1 + S2, S1.mFcTag, S1.HisTag, S1, S2, Spike.RBD.His.Bac, Spike.RBD.rFc, and S1.RBD.mFc were most highly ranked among all features, where S1 and S2 are the subunits of Spike, and the suffixes represent the tagging information of different recombinant proteins. Meanwhile, the classification rules were obtained from the optimal decision tree to explain quantitatively the roles of antigens in the classification. This study identified antibodies associated with decreased clinical immunity based on populations with different time spans after vaccination. These antibodies have important implications for maintaining long-term immunity to SARS-CoV-2.

## 1. Introduction

Severe acute respiratory syndrome coronavirus 2 (SARS-CoV-2) is the novel coronavirus strain causing Coronavirus Disease 2019 (COVID-19) [1]. On 11 March 2020, COVID-19 was finally classified as a pandemic by the World Health Organization (WHO) [2]. More than 6.3 million people have died from COVID-19 globally, according to the WHO, and more than 500 million cases have been confirmed. Additionally, more than 11 billion doses of vaccine have been distributed [3]. Fever, sore throat, dry cough, and pneumonia symptoms are among the clinical manifestations of COVID-19 [4]. During the span of this study, the Omicron variant was prevalent. The Omicron variant, which evolved from the Alpha variant, has increased infectivity compared to earlier variants [5]. Increased infectiousness and antibody evasion have been linked to the mutations in the SARS-CoV-2 spike protein [6].

Scientists have developed COVID-19 vaccines to combat the pandemic. To date, some types of vaccines against SARS-CoV-2 have been developed and widely used worldwide, such as the RNA-based type, non-replicating viral vector type, and protein-based type [7]. BNT16b2 (Pfizer—New York, NY, USA and BioNTech—Mainz, Germany), mRNA-1273 (Moderna—Cambridge, MA, USA), Ad26.COV2.S (Johnson & Johnson—New Brunswick, NJ, USA), CIGB-66 Abdala (Cuban Genetic Engineering and Biotechnology Center—Havana, Cuba), and other common vaccines require one to three doses, depending on the type [7,8,9,10]. BNT162b2 contains mRNA encoding a full-length stable S glycoprotein that elicits dose-dependent SARS-CoV-2 neutralizing antibody titers [11]. Two doses of BNT162B2 exhibit approximately 95% protection against severe illness [9,12,13,14,15]. As of early 2023, all vaccines have efficacy in reducing COVID-19 severe cases and death while their efficiency in controlling viral infection and mild symptoms is not very satisfactory [9,10,16,17]. Vaccine coverage must be extended to all countries while maintaining and improving public health control mechanisms to control COVID-19 morbidity and mortality worldwide.

However, the efficacy of the BNT162b2 mRNA vaccine against SARS-CoV-2 decreases over time [11,18]. In addition, there have been reports of vaccine-induced protection waning progressively due to the emergence of new variants [19,20]. Whether the decline in vaccine protection is linked to a decrease in virus resistance remains unclear. Vaccines trigger a complicated immunological response that includes B and T cells, with B cells producing antibodies [18,21,22]. Spike (S), membrane (M), nucleocapsid (N), and envelope (E) are the four structural proteins encoded by SARS-CoV-2 [23,24,25]. Most of the antibodies generated by vaccination are directed against the S protein, specifically the receptor-binding domain (RBD) [7,26]. A recent study of antibody alterations following two doses of inactivated COVID-19 vaccine, separated into three groups based on immunization duration, revealed that the levels of antibodies (anti-Spike IgG) decrease with time [27]. While existing studies have begun to chart the territory of antibody profiles post-COVID-19 vaccination [28,29,30,31], the detailed interplay between antibody and vaccination remains incompletely revealed. More comprehensive research is urgently needed to pinpoint the most critical antibodies that neutralize the virus effectively and determine their duration in the human body. This knowledge is paramount for enhancing vaccine strategies, potentially developing superior treatments, and guiding public health policies regarding booster shots and containment measures, ultimately fortifying our fight against the pandemic.

In the current study, we investigated the influence of vaccines on antibody synthesis and monitored changes in antibody levels in the body over time following vaccination. Data on blood antibody levels in a cohort of volunteers vaccinated for COVID-19 vaccines were sourced from the Gene Expression Omnibus (GEO). The GEO data used for our analyses were originally measured using antigen microarrays [32]. The volunteers were examined for their reaction before receiving the mRNA vaccine (Pfizer or Moderna), shortly after receiving the first and second doses, and up to 6 months later. Vaccine-induced antibodies are mainly directed against the S1 and RBD domains of the S protein and to a lesser extent against the S2 domain. Antibody levels were increasing significantly 2 months after vaccination and begin to decline after 6 months. Seventy-three antigens and 1373 volunteer records were involved in the study of Hosseinian et al. [32]. In the present study, 1373 samples were classified into four groups according to the time of vaccination: before vaccination, within 60 days of vaccination, 60–180 days after vaccination, and over 180 days after vaccination. Multiple machine learning methods were integrated to identify key antigen-reactive antibodies that changed after COVID-19 vaccination over time and to establish quantitative rules for accurate prediction. Several essential antigen-reactive antibodies and classification rules were obtained, some of which were extensively analyzed. The results of this study could serve as a basis for developing effective vaccines with long-lasting protection and elucidating the defense mechanisms of COVID-19 vaccines.

## 2. Materials and Methods

### 2.1. Data and Preprocessing

Individualized antibody reactivity levels for SARS-CoV-2 antigens induced by mRNA vaccines were quantified using a coronavirus antigen microarray (CoVAM) following the procedure described by Hosseinian et al. [32]. Data were sourced from the GEO database using accession number GSE199668. The samples were divided into four classes according to the time of vaccination: 104 unvaccinated healthcare workers, 534 healthcare workers within 60 days after vaccination, 594 healthcare workers between 60 and 180 days after vaccination, and 141 healthcare workers over 180 days after vaccination [32]. In terms of features, the CoVAM contained 10 SARS-CoV-2 antigens, including nucleocapsid protein (NP) and several varying fragments of the S protein, as well as 4 SARS, 3 MERS, 12 Common CoV, 8 influenza, and 36 other antigens. In terms of feature naming, the virus name was placed at the beginning to distinguish between the different sources of antibodies, followed by the protein name, and the specific tag name followed after the protein name. The feature names and their descriptions are provided in Table S1. The normalized fluorescence intensity was used to characterize the expression levels of antigen-reactive antibodies in blood. The above features and four classes comprised the classification problem. By investigating the problem, essential features can be obtained.

### 2.2. Feature Selection Methods

Several features were adopted to represent samples. Some of them were important to classify samples into different classes, whereas others were not. In machine learning, the important features can be extracted by feature selection methods. To date, many such methods have been proposed. It is a challenge to select the correct one to process a given dataset. Generally, one single method can only output a part of the essential features as each method has its limitations. In this study, we adopted four feature selection methods: least absolute shrinkage and selection operator (LASSO) [33,34], light gradient boosting machine (LighGBM) [35], Monte Carlo feature selection (MCFS) [36] and maximum relevance minimum redundancy (mRMR) [37]. These methods were designed following different principles, meaning that they can overview the given dataset from different aspects. Thus, more essential features can be obtained by applying them to the same dataset. Their brief descriptions are as follows.

Least Absolute Shrinkage and Selection Operator. The LASSO is a statistical method used for regularization and feature selection [33,34]. This method reduces the regression coefficients of the redundant features to zero. The feature selection phase occurs after the reduction, where non-zero-valued features are sorted by the absolute value of their coefficients. This study used the LASSO program implemented in Scikit-learn [38], which was run with default parameters.

Light Gradient Boosting Machine. The LightGBM is a free and open-source distributed gradient boosting framework for machine learning that was created by Microsoft [35]. It performs regression and classification by transforming weak decision tree (DT) classifiers into strong learners. In addition to regression and classification, this method ranks features according to their importance, measured by the number of times they are picked up for building DTs. A high ranking is given to features that are used frequently. LightGBM was implemented through a Python module, which can be obtained at https://lightgbm.readthedocs.io/en/latest/ (accessed on 10 May 2020). This program was also performed under default parameters.

Monte Carlo Feature Selection. The MCFS is a useful tool for selecting informative features according to their relative importance in building DTs [36,39,40,41]. Subsets of features are randomly constructed many times. For each subset, some samples are randomly selected for training, and the others are used for testing. For instance, a DT is built based on two out of three of the samples that are randomly selected, and the rest is used for testing, which is also repeated many times. The relative importance (RI) of each feature can then be estimated by considering the number of times they are used to construct the DTs, the information gain of the features, and the weighted accuracy of the DTs. Finally, features can be sorted according to their RI scores. The MCFS program adopted in this study was retrieved from http://www.ipipan.eu/staff/m.draminski/mcfs.html (accessed on 4 June 2019). Additionally, it was executed using default parameters.

Maximum Relevance Minimum Redundancy. The mRMR is a classic and powerful feature selection method [37]. It measures the importance of features according to two aspects: (1) relevance to class variable, (2) redundancy to other features. The relevance and redundancy are all measured by mutual information (MI). Similar to the above methods, mRMR also generates a feature list to indicate the importance of features. At first, the list is empty. Then, a loop procedure is executed. In each round, one feature with maximum relevance to class variable and minimum redundancy to features in the current list is selected from all remaining features, which is appended to the current list. The loop procedure stops until all features have been put into the list. The mRMR program used in this study was obtained from http://home.penglab.com/proj/mRMR/ (accessed on 2 May 2018) and it was executed with the default settings.

The above four feature selection methods were applied to the dataset mentioned in Section 2.1, resulting in four feature lists, which were termed as LASSO, LightGBM, MCFS and mRMR feature lists.

### 2.3. Incremental Feature Selection

Although the feature selection methods can sort features in lists, it still retains a gap for extracting essential features. It is not easy to determine how many top features should be selected. In view of this, incremental feature selection (IFS) was employed in this study [42]. It can find out the optimal number of features for building the classifiers with best performance [43,44,45]. In the present study, one step interval was applied to each given list in the IFS method. Under this setting, a series of feature subsets were constructed in the following manner. The first subset contained the first feature in the list, the second one contained the top two features, and so on. A classifier was built for each feature subset based on one classification algorithm and samples encoded by features in this subset. All classifiers were tested by tenfold cross-validation [46]. According to the evaluation results, the classifier providing the highest performance was selected. It was termed as the optimal classifier and the optimal feature set was defined as the corresponding feature subset.

### 2.4. Synthetic Minority Oversampling Technique

As mentioned in Section 2.1, there are significant differences in the size of the four classes. The classifier built on such datasets may generate bias. This should be tackled by using some advanced computational methods. Here, we selected the synthetic minority oversampling technique (SMOTE) [47,48,49]. The idea of this method is to generate synthetic samples for each minority class, thereby balancing the dataset. In detail, it randomly chooses a sample from one minority class and determines its *k* nearest neighbors in the same class. One of its neighbors is randomly selected and a synthetic sample is generated by the linear combination of the sample and its chosen neighbor. This newly generated sample is put into the minority class, thereby enlarging its size. This procedure can be performed several rounds until the minority class contains the same number of samples as the majority class. Herein, we used the SMOTE tool from https://github.com/scikit-learn-contrib/imbalanced-learn (accessed on 24 March 2020) with default parameters.

### 2.5. Classification Algorithms

In the IFS method, one classification algorithm should be employed for building classifiers. This study adopted four classification algorithms: DT [50], K-nearest neighbor (KNN) [51], support vector machine (SVM) [52], and random forest (RF) [53]. These algorithms have wide applications in tackling various medical and biological problems [54,55,56,57,58,59,60]. DT uses a tree-like model to build classifiers, which can be extended by maximizing Gini index or information gain in each tree node [50]. The KNN algorithm finds the nearest neighbors of a new sample and categorizes the new sample into one that is shared by most of its nearest neighbors [51]. The SVM can map samples into a high-dimensional space and finds a hyperplane that distinctly classifies samples in different classes. The test samples are then mapped into the same space and the category to which they belong are predicted based on which side of the hyperplane they fall [52]. A RF consists of a large number of individual DTs that operate as an ensemble [53]. Each decision tree in an RF generates class predictions on a test sample, and the class with the most votes is taken as the prediction result.

### 2.6. Performance Assessment

The weighted F1 is a widely used measurement in multi-class classification, which was selected as the key measurement to assess the performance of the classifier. For the calculation of the measurement, the F1-measure in each class should be calculated in advance. It is defined as the harmonic mean of the other two measurements: recall and precision, where recall is the proportion of correctly predicted positive samples among all positive samples, precision is the proportion of correctly predicted positive samples among all predicted positive samples. The weighted F1 is the weighted average of all F1-measure values on different classes, where the weight for one class is defined as the proportion of samples in this class.

In addition, other measurements were also employed to give a full display of the performance of classifiers. The first one was Macro F1, which is another way to integrate the F1-measure values of different classes, which is defined as the mean of all F1-measure values. The second one was prediction accuracy (ACC) which is the most classic measurement to assess the performance of classifiers. It is defined as the ratio of the number of correctly predicted samples and the overall sample number. However, when the dataset is imbalanced, ACC is not accurate enough. Matthew correlation coefficients (MCC) [61] is a more balanced measurement than ACC. Two matrices are used to calculate MCC. One is to store the true class of each sample and the other one is to store the predicted class of each sample. MCC assesses the relationship between these two matrices.

### 2.7. Extraction of Essential Features for Each Class

Based on the IFS method, some essential features can be obtained. However, it is not clear which class they are highly related to. In view of this, we reconstructed a dataset for each class and applied the above feature selection methods to it. For one class, one dataset was generated, in which samples in this class were considered as positive samples and other samples were regarded as negative samples. Then, LASSO, LightGBM, MCFS, and mRMR were adopted to investigate this dataset, resulting in four feature lists. From each list, the top 20 features were picked, thereby obtaining four feature subsets. By investigating the overlap of these feature subsets, some essential features that occurred in multiple subsets can be obtained, which were deemed to be highly related to the given class.

## 3. Results

**Figure 1:**
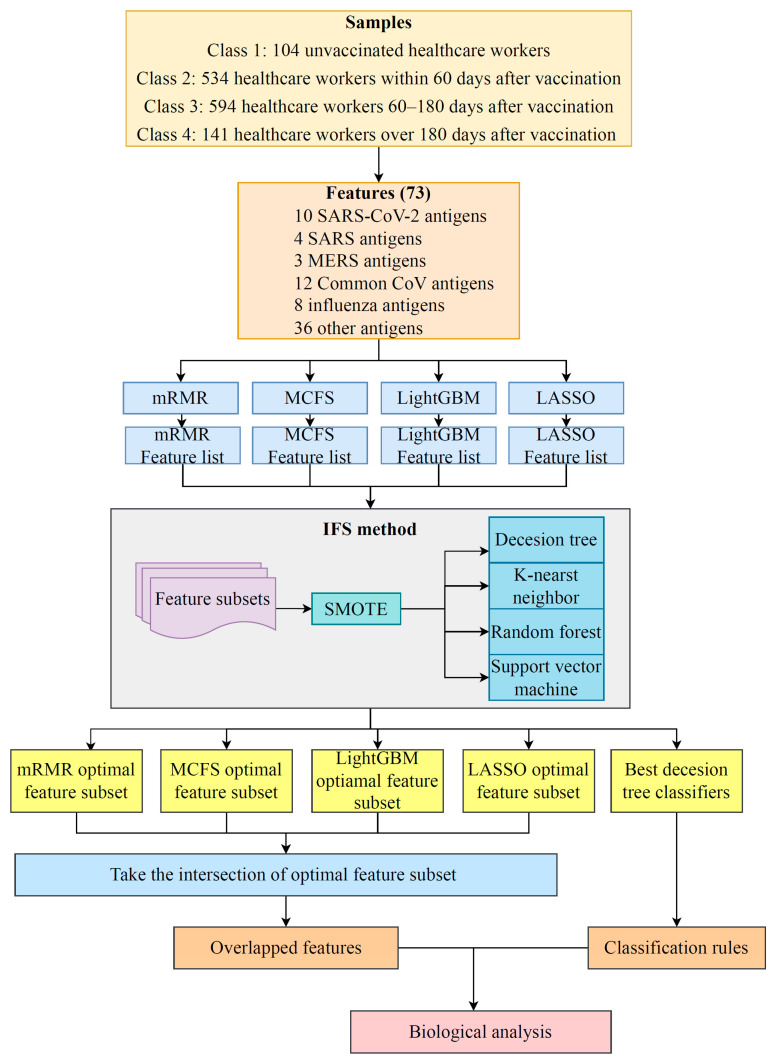
Flow chart of the entire analytical process. The 73 antigens in samples from four classes were ranked in terms of feature importance by four feature selection algorithms, including LASSO, LightGBM, mRMR, and MCFS. Such procedure generated four feature lists, which were fed into the IFS method. Efficient classifiers were set up, which used the optimal feature subset from each list. At the same time, classification rules were also built. Obtained optimal feature subsets were investigated to obtain antigens recurring in multiple subsets. Lastly, a biological analysis was performed on the above-obtained antigens and classification rules.

In this study, a dataset on the antibody reactivity levels for SARS-CoV-2 antigens induced by mRNA vaccines was investigated. The overall computational framework is illustrated in Figure 1. The results in each step are presented in this section.

### 3.1. Results of Feature Selection Methods

According to the framework, four feature selection methods were used to rank the 73 antigens based on the degree to which they contributed to the classification. These lists are provided in Table S2. For easy descriptions, they were called LASSO, LightGBM, MCFS and mRMR feature lists.

### 3.2. IFS Results and Feature Intersection

**Figure 2:**
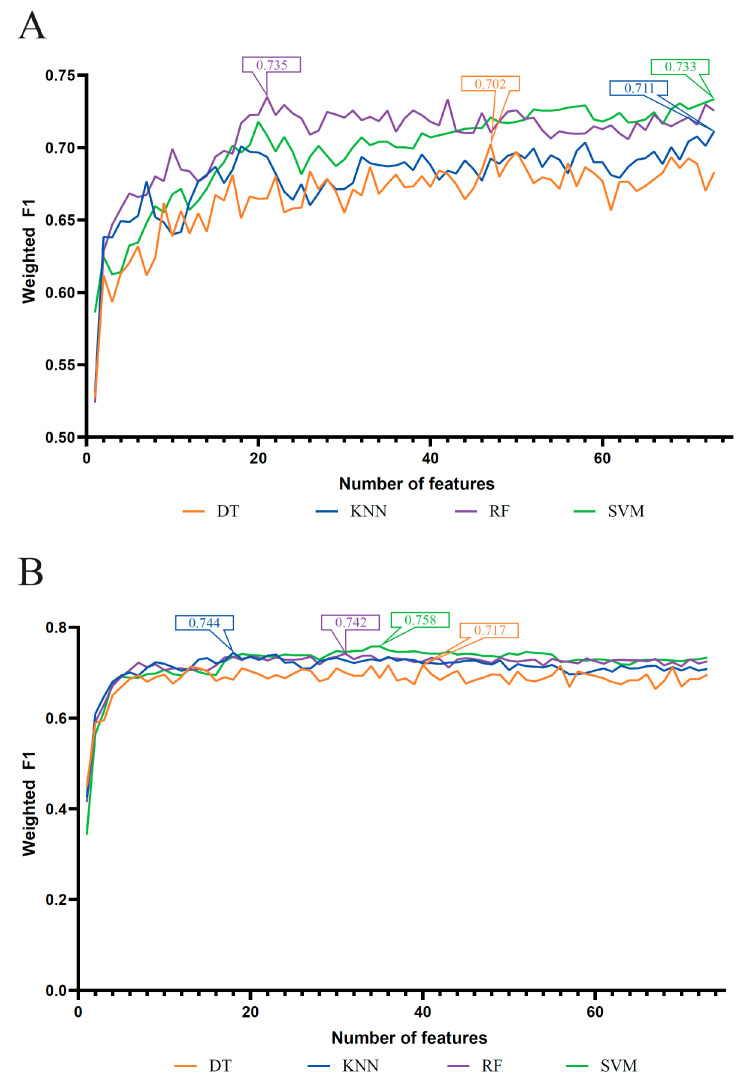
IFS curves of four classification algorithms based on IFS results on the LASSO and LightGBM feature lists. (**A**) IFS curves of the LASSO feature list, (**B**) IFS curves of the LightGBM feature list. The number in each box was the highest weighted F1 for one classification algorithm.

**Figure 3:**
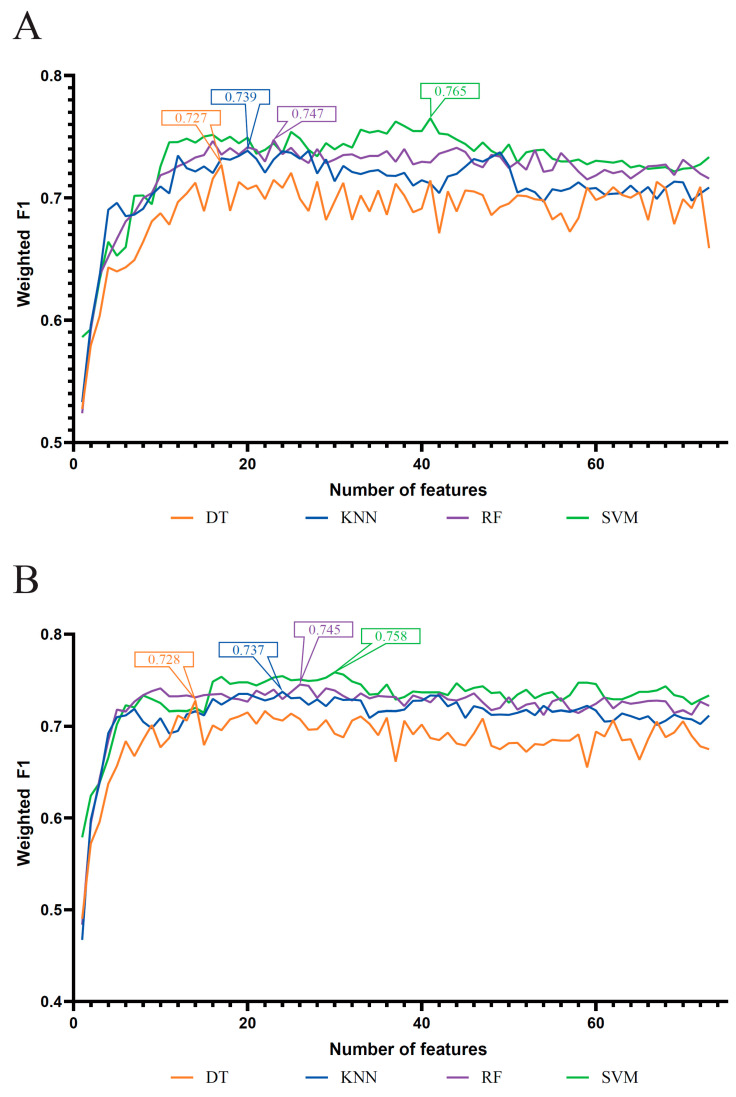
IFS curves of four classification algorithms based on the IFS results on the MCFS and mRMR feature lists. (**A**) IFS curves on the MCFS feature list, (**B**) IFS curves on the mRMR feature list. The number in each box was the highest weighted F1 for one classification algorithm.

As mentioned above, four feature lists were obtained. Each list was put into the IFS method one by one. DT, KNN, RF, and SVM were adopted in the IFS method. The performance of each classification algorithm under some top features in each list is listed in Table S3. Using the weighted F1 as the major measurement, we compared the performance of the classifiers using the same classification algorithm and feature list. Several IFS curves were generated by plotting the weighted F1 on the y-axis and the number of features on the x-axis, as shown in Figure 2 and Figure 3.

For the LASSO feature list, Figure 2A shows the IFS curves based on four classification algorithms. When the top 47, 73, 21 and 73 features in each list were used, the DT, KNN, RF and SVM can yield the highest weighted F1 values of 0.702, 0.711, 0.735 and 0.733, respectively. Accordingly, the optimal DT, KNN, RF and SVM classifiers can be built with the corresponding top features. Their detailed performance, including ACC, MCC, macro F1 and weighted F1, is provided in Table 1. Evidently, the optimal RF classifier was better than the other three optimal classifiers.

For the LightGBM feature list, the obtained four curves are illustrated in Figure 2B. From this figure, four optimal classifiers can be obtained, which adopted the top 40, 18, 31 and 35 features in the list. They generated the weighted F1 values of 0.717, 0.744, 0.742 and 0.758. Table 1 also lists the performance of these optimal classifiers. Clearly, the optimal SVM classifier was a little better than the other three optimal classifiers.

For the MCFS feature list, the IFS results on this list were summarized as four IFS curves, as shown in Figure 3A. It can be observed that the optimal DT/KNN/RF/SVM classifier adopted the top 17/20/23/41 features in this list. The detailed performance of these optimal classifiers is provided in Table 1. Evidently, the optimal SVM classifier was the best among four optimal classifiers, which produced a weighted F1 of 0.765.

As for the last mRMR feature list, Figure 3B displays the IFS curves on four classification algorithms. The highest weighted F1 values for the classification algorithms were 0.728 (DT), 0.737 (KNN), 0.745 (RF) and 0.758 (SVM), respectively. This performance was obtained by using the top 14, 24, 26 and 30 features in the corresponding feature list. Thus, the optimal DT, KNN, RF and SVM classifiers can be set up using these features. Table 1 lists their detailed performance. The optimal SVM classifier yielded better performance than the other three optimal classifiers.

**Figure 4:**
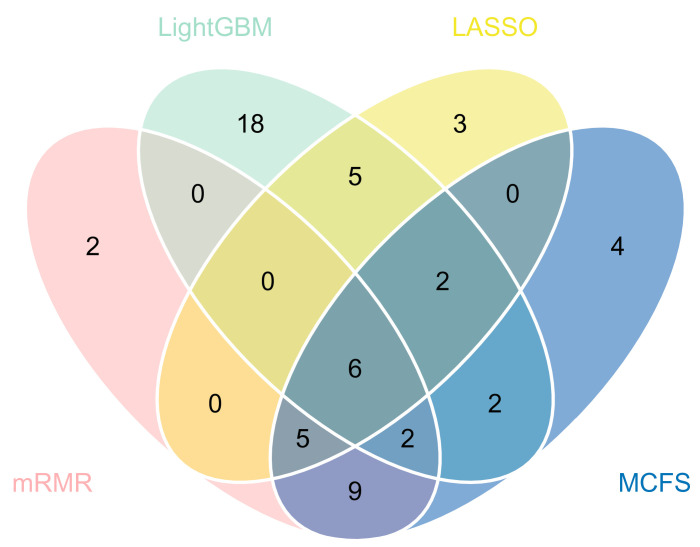
Venn diagrams of the optimal feature subsets extracted from the LASSO, LightGBM, MCFS, and mRMR feature lists. The overlapping circles indicated antigens that were included in multiple optimal feature subsets.

According to the above results, we can find the best classifiers of four feature lists. In detail, the best classifier in the LASSO feature list was the optimal RF classifier, whereas it was the optimal SVM classifier in the other three lists. We picked up the optimal feature subsets for further investigation. A Venn diagram was plotted for these subsets, as illustrated in Figure 4. The intersection results of these optimal feature subsets are available in Table S4. The antigens appearing in several feature subsets suggest that they were identified as important by multiple feature selection methods. They can play important roles in differentiating healthcare workers at different time spans after vaccination. The biological significance of some antigens (features) will be discussed in Section 4.

### 3.3. Essential Features for Each Class

**Figure 5:**
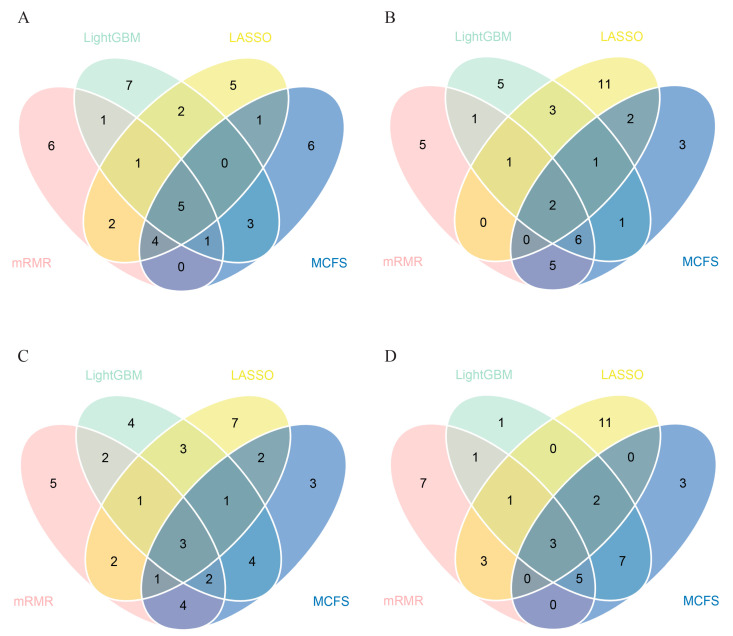
Venn diagrams of top features identified by LASSO, LightGBM, MCFS, and mRMR methods for four classes. For each class, the top 20 antigens in the four feature lists were selected for taking the intersection. These antigens were considered to be highly associated with one particular class. (**A**) Venn diagram for unvaccinated healthcare workers; (**B**) Venn diagram for healthcare workers within 60 days after vaccination; (**C**) Venn diagram for healthcare workers between 60 and 180 days after vaccination; (**D**) Venn diagram for healthcare workers over 180 days after vaccination.

The essential features obtained above may not be highly related to one class. To extract the essential features for each class, four datasets corresponding to the four classes were constructed, as described in Section 2.7. Then, LASSO, LightGBM, MCFS and mRMR were applied to each dataset. Four feature lists were obtained. The top 20 features were selected for taking the intersection. A Venn diagram was drawn for each class, as illustrated in Figure 5. The specific antigen names are listed in Table S5. For the first class, namely, unvaccinated healthcare workers, antigens such as SARS.CoV.2.S1.RBD.mFc and SARS.CoV.S1.HisTag were identified by all four feature selection methods. For the second class, namely, healthcare workers within 60 days after vaccination, SARS.CoV.2.S1.mFcTag and HuIgM.0.30 were deemed to be important by all feature selection methods. For the third class, namely, healthcare workers between 60–180 days after vaccination, three features (SARS.CoV.2.S1.mFcTag, HuIgM.0.30, and SARS.CoV.2.S1.RBD.mFc) were identified to be essential. For the fourth class, namely, healthcare workers over 180 days after vaccination, MERS.CoV.S1.RBD.367.606.rFcTag, Flu.B_Mal/.HA1, and a-HuIgG_0.03 were screened out by all methods. The discussion on the importance and functionality of some features will be provided in detail in Section 4.

### 3.4. Classification Rules

It can be observed from Table 1 that the optimal DT classifier was generally inferior to the other three optimal classifiers on the same feature list. However, the DT classifier has a great merit that was not shared by the other three classifiers. It can provide a group of classification rules, which made the classification procedures completely open. The optimal DT classifiers on four feature lists adopted the top 47, 40, 17 and 14, respectively, features in the corresponding lists. All healthcare workers were represented by the above features, respectively. Four trees were built by DT, from which four rule groups were established. These rules are provided in Table S6; 190, 183, 202, and 226 classification rules, respectively, were contained in four groups. Each rule is composed of antigen features and their associated fluorescence intensity values, which explains how the feature’s high or low fluorescence intensity influences the capacity to identify the classes of samples. A detailed discussion of some quantitative rules can be found in Section 4.

## 4. Discussion

We identified a set of antigen-reactive antibodies as potential features that could reveal the effect of COVID-19 vaccines on anti-viral immune activation and reflect changes in antibody levels in the body over time after vaccination by using data on serum antibody levels in volunteers after receiving COVID-19 vaccines. This confirms the potential of such features to contribute to the development of effective vaccines with long-lasting protection. The serum antibody data we analyzed were detected by a coronavirus antigen microarray (CoVAM). The microarray approach has been extensively applied in SARS-CoV-2 research due to its excellent sensitivity and specificity [62,63,64]. Recently, this method was frequently employed for measuring antibody levels following mRNA vaccination [30,65]. Recent publications have found that some identified features, as well as the relevant quantification rules, are linked to vaccine-induced anti-viral immune activation and duration.

### 4.1. Key Features for Identifying the Effect of COVID-19 Vaccines on Antibody Production

Using these computational methods, we discovered a set of unique viral antigens-reactive antibodies selected by at least three methods. The antigens we analyzed are from epidemic coronaviruses, including SARS-CoV-2, SARS-CoV, MERS-CoV, common cold coronaviruses, and multiple subtypes of influenza. S1, S2, and RBD are components of SARS-CoV-2’s spike protein, which it uses to infect cells. Moreover, ‘tags’ were attached to these proteins to make them easier to study. For example, ‘mFcTag’ is a piece from a mouse antibody, and ‘HisTag’ is a chain of specific building blocks, both used for tracking and purifying the protein. These top-specific antibodies are closely related to the components of various COVID-19 vaccines, suggesting the protective effect of these vaccines. In the present study, we analyzed 13 specific antibodies, listed in Table 2. In this section, we compared the changes in significant viral antigen-reactive antibodies in the serum of vaccinated and unvaccinated individuals. We also discussed the plausibility and cross-immunization of important antibodies (including non-SARS-CoV-2 antibodies) induced by COVID-19 vaccines.

The top eight features identified were from SARS-CoV-2: S1 + S2, S1.mFcTag, S1.HisTag, S1, S2, Spike.RBD.His.Bac, Spike.RBD.rFc, and S1.RBD.mFc. The compositions of COVID-19 vaccines are listed in a recent paper comparing these vaccines [7]. The S protein of SARS-CoV-2 was chosen as a promising target by the majority of COVID-19 vaccines because blocking the interaction between the RBD of echinocandin and human angiotensin-converting enzyme 2 (ACE2) is effective in preventing infection [66,67]. In addition, the RBD is part of the S protein’s S1 subunit [68,69]. Suthar et al. highlighted that the S protein of SARS-CoV-2, particularly RBD, stimulates the production of neutralizing antibody NAbs [70]. Similarly, an animal study revealed that RBD-specific IgG accounts for half of the antibody responses induced by S proteins. As a result, given that popular COVID-19 vaccines such as BNT162B1 encode the S protein of SARS-CoV-2, they can stimulate the production of S protein (including S1 and S2 subunits) and RBD-specific antibodies.

SARS.CoV.S1.HisTag and SARS.CoV.S1.RBD.HisTag are top features from SARS-CoV. SARS-CoV and SARS-CoV-2, both belonging to β-B coronavirus, and share 79% of their gene sequences [71,72], and the S protein shares 76% of its amino acid identity [73]. SARS-CoV-2 and SARS-CoV share the same host cell receptor ACE2 and are structurally similar; thus, they may exhibit some degree of cross-immunity [67]. These data suggest the effectiveness of SARS-CoV-reactive antibodies against SARS-CoV-2. These results were further confirmed by Wec et al., who isolated several antibodies from a SARS survivor that neutralized coronaviruses such as SARS-CoV-2 [74]. Min et al. identified several monoclonal antibodies against SARS-CoV S protein or RBD that are cross-immunoreactive with SARS-CoV-2 [26], which agrees with our predicted features.

MERS.CoV.S1.RBD.367.606.rFcTag from MERS-CoV was the next feature identified. MERS-CoV also belongs to β coronavirus and shares a 50% sequence similarity to SARS-CoV-2 [71], a coronavirus with a high lethality rate. The S protein of MERS-CoV and the RBD in it share some similarities to SARS-CoV-2, suggesting that the cross-immunity of the RBD-specific antibody to the S protein of MERS-CoV against SARS-CoV-2 is less than that of the SARS-CoV-specific antibody, but still exists.

The last two identified features, hCoV.HKU1.NP and hCoV.229E.S1, are antigens from β coronavirus hCoV-HKU1 and α coronavirus hCoV-229E, respectively. Cross-immunization with SAR-CoV-2 is possible due to their close relationship. HCoVs are composed of proteins called spike (S), membrane (M), envelope (E), and nucleocapsid (N) [75]. In addition to the S protein, the N protein is an important antibody target [70,76], implying that hCoV.HKU1.NP-specific antibodies contribute to SARS-CoV-2 prevention. Although hCOV-228E is less closely related to SARS-CoV-2 than the other coronaviruses we mentioned above, the potential preventive effect of its specific antibodies against COVID-19 cannot be ruled out. However, given that hCoV-HKU1 and hCoV-229E are common coronaviruses, the detection of these antibodies in the sera of volunteers may be attributed to their previous infection.

Research on pan-coronavirus vaccines has attracted increasing attention to prevent novel SAR-CoV-2 variants. Some studies reported that conserved regions on the inner surface of the RBD are potential targets for pan-coronavirus vaccines [77]. New studies of mRNA vaccines against a variety of the more common coronaviruses are underway [78]. In summary, the positive serum test for non-SARS-CoV-2 antigens could be due to the ability of certain antibodies induced by COVID-19 vaccines to act on other coronaviruses. Therefore, the non-SARS-CoV-2 antigens we mentioned above can be seen as useful features.

### 4.2. Features Related to Time since Vaccination for Determining the Duration of Specific Antibodies after COVID-19 Vaccination

**Figure 6:**
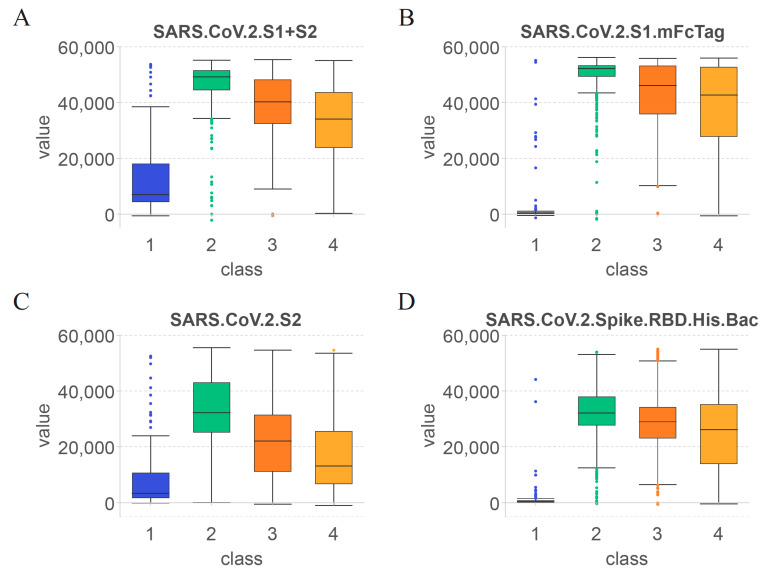
Fluorescence intensity distribution of top antigens in four subclasses. Box plots show trends of four important antigen-reactive antibodies according to each subclass assigned by time after vaccination. (**A**) S1 + S2, (**B**) S1.mFcTag, (**C**) S2, (**D**) Spike.RBD.His.Bac. Numbers in the abscissa represent the indices of four classes. Classes 1–4 represent unvaccinated healthcare workers, healthcare workers within 60 days after vaccination, healthcare workers between 60 and 180 days after vaccination, and healthcare workers over 180 days after vaccination, respectively.

The essential antigen-reactive antibodies were identified using computational methods and divided into four classes based on vaccination time. The top features from each subclass were selected for discussion. Figure 6 shows the values of these top features in each of the four classes to visualize the changes in the antibodies that target specific antigens over time. Unlike the previous section, this section focuses on the changes in important antibodies at different periods after vaccination according to subclasses, including unvaccinated cases.

The S protein of SARS-CoV-2 is currently the antigen targeted by a majority of COVID-19 vaccines [7,11,16,27,79]. The top features we identified are contained in the S protein of SARS-CoV-2, and antibodies against them all change significantly over time after vaccination.

As shown in Figure 6A, the first identified feature was SARS.CoV.2.S1 + S2. Based on the overall structure of the S protein of SARS-CoV-2 [80], the specificity of the SARS.CoV.2.S1 + S2-reactive antibodies was the lowest among the four selected features. As shown in Figure 6B–D, the second, third, and last identified features were SARS.CoV.2.S1.mFcTag, SARS.CoV.2.S2, and SARS.CoV.2.Spike.RBD.His.Bac, respectively.

According to the changes in the value of each feature in class 1 (unvaccinated healthcare workers), SARS.CoV.2.S1 + S2 and SARS.CoV.2.S2 showed elevated levels, whereas SARS.CoV.2.S1.mFcTag and SARS.CoV.2.Spike.RBD.His.Bac were almost undetectable in serum. Thus, antibodies against the S2 subunit of the S protein were produced earlier after immunization and resulted in relevant specific protection. However, volunteers infected with SARS-CoV-2 before COVID-19 vaccination may also increase the levels of SARS.CoV.2.S1 + S2 and SARS.CoV.2.S2.

Comparison of the levels of the four features in class 2 (healthcare workers within 60 days after vaccination) revealed that SARS.CoV.2.S1.mFcTag showed the most significant increase, and the values were relatively concentrated within a month after vaccination. The values of SARS.CoV.2.S2 increased less significantly and were less consistent than those of SARS.CoV.2.S1.mFcTag. A study of healthcare workers found a 14-day boost in serum anti-S antibodies, followed by a significant drop in anti-S antibody levels until 42 days after vaccination [81]. Therefore, the levels of other antigens contained within the S protein of SARS-CoV-2 can also elevate antibodies against them within 42 days after vaccination, which agrees with the results of the present study.

Based on the trend from class 2 (healthcare workers within 60 days after vaccination) to class 4 (healthcare workers over 180 days after vaccination), the values of all features showed varying degrees of decline after 60 days. Among them, the values of SARS.CoV.2.Spike.RBD.His.Bac and SARS.CoV.2.S1.mFcTag declined slower than those of the other features and stimulated some stable antibodies that existed for a longer period. By contrast, the levels of SARS.CoV.2.S1 + S2 and SARS.CoV.2.S2 decreased more rapidly, suggesting that the S2 subunit is less ideal as an antibody target than the S1 subunit and RBD after COVID-19 vaccination. Similarly, previous studies reported that the antibodies identified in the serum following immunization are predominantly anti-S or anti-RBD antibodies [9,10,14] which appears to support this hypothesis.

The levels of features in class 4 (healthcare workers over 180 days after vaccination) were maintained at high levels, except for SARS.CoV.2.S, which was lower. This result indicates that the features found after COVID-19 immunization can persist for more than 6 months (180 days). The immunogenicity of mRNA-1273 lasts for at least 3 months [82], whereas that of BNT162b2 lasts for at least 2 months [12]. The varied compositions based on the type of vaccines can lead to variation in the duration of specific antibody presence. However, the four features identified imply that the S-protein and RBD-specific antibodies are present in the serum for long periods in general.

### 4.3. Rules for Quantitative Time after COVID-19 Vaccination and Antibody Levels

In addition to the qualitative features, a set of quantitative rules for accurate classification at the time after COVID-19 vaccination were established. All criteria were linked to specific antibody levels, and they were selected using at least two sorting methods. Some top features have been validated as having the ability to classify samples. In the present study, we selected the most typical rules for each time group for further discussion. Table 3 lists all of the rules, followed by a comprehensive analysis.

Rule 0 applies four criteria to identify unvaccinated samples. The thresholds for SARS.CoV.2.S1.mFcTag and SARS.CoV.2.S1.HisTag are outlined in Table 3. The low levels of anti-S1 antibodies suggested by these values are consistent with the lack of vaccination. Studies indicate that even a single vaccine dose can trigger a robust anti-S1/2 antibody response in SARS-CoV-2-infected individuals [83], and that antibody responses are not immediate following a single vaccine dose [13], validating the accuracy of these criteria. The third criterion, SARS.CoV.2.S1.RBD.mFc, should be within the range set out in Table 1, typically low in unvaccinated individuals. Vaccination raises anti-RBD IgG levels in the body [84], so this range helps to distinguish vaccinated individuals. The final criterion is hCoV.OC43.HE, an antigen from a common coronavirus that causes similar symptoms to the common cold, whose threshold is listed in Table 3. If its serum level is above the threshold specified in Table 1, it suggests prior exposure to hCoV.OC43, or possibly transient vaccine-induced cross-reactive antibodies to other HCoVs [85]. Over time, vaccinations prompt the production of more precisely targeted antibodies [18], which further aids in excluding vaccinated individuals.

Rule 1 incorporates three criteria for identifying individuals 0 to 60 days post-vaccination. The first criterion is SARS.CoV.2.S1.mFcTag, which should not exceed the limit outlined in Table 3. High levels of anti-S/RBD antibodies are typically observed 8 weeks after mRNA-1273 or BNT162b2 vaccination [14], and given that most vaccines generate antibody responses against S proteins, including the S1 subunit, an increase in anti-S1 antibodies is expected post-vaccination. However, due to the finite antibody production by vaccines [86], a maximum value is set within this period [9]. The second and third criteria refer to SARS.CoV.2.S2 and SARS.CoV.2.S1 + S2. Their serum levels should exceed the thresholds specified in Table 3. As the S1 and S2 subunits are included in the S protein, changes in the level of S1 + S2 specific antibodies should have a strong correlation with anti-S antibodies. A recent study has reported that the levels of anti-S antibodies in serum significantly increase 14 days after vaccination [81], supporting the high thresholds for SARS.CoV.2.S1 + S2 in this rule. Anti-S2 antibody levels also increase significantly post-vaccination [87], although their reactivity is generally lower than that of anti-S1 and anti-RBD responses [13]. These results confirm that the high value of SARS.CoV.2.S2 facilitates the differentiation while the lowest value of SARS.CoV.2.S2 in Rule 1 can be lower than that of SARS.CoV.2.S1 + S2.

Rule 2 utilizes three criteria to identify individuals 60–180 days post-vaccination. The first two criteria, SARS.CoV.2.S1.mFcTag and SARS.CoV.2.S1.RBD.mFc, should have serum levels above the threshold set in Table 3, and between the range specified for SARS.CoV.2.S1.RBD.mFc. The vaccine’s protective capability is associated with antibody count, and research indicates that COVID-19 vaccine efficacy decreases from 1 to 6 months post-vaccination [19], suggesting a corresponding decline in antigen-reactive antibodies. Although no study has yet confirmed the range levels outlined in our rule, it is reasonable to predict that SARS.CoV.2.S1.mFcTag levels would be lower than in Rule 1, while SARS.CoV.2.S1.RBD.mFc levels would be higher than in Rule 0. The final criterion, SARS.CoV.S1.HisTag, stands out from the first two as it pertains to an antigen from SARS-CoV, not SARS-CoV-2. Given the substantial sequence similarity between SARS-CoV and SARS-CoV-2 [88], the existence of cross-reactive non-specific epitopes led us to include SARS.CoV.S1.HisTag as a criterion in Rule 2. Lv et al. reported that some SARS-CoV-2-infected individuals can create cross-reactive antibodies that bind to the RBD of SARS-CoV [89], implying that the COVID-19 vaccination can stimulate similar cross-reactive antibodies in individuals.

The final rule (Rule 3), for people who have been vaccinated for more than 180 days, sets thresholds for SARS.CoV.2.S1.mFcTag and SARS.CoV.2.S1.RBD.mFc as set out in Table 3. These values are similar to Rule 2, probably because the vaccine-induced production of these antibodies drops to its lowest level after 180 days [90,91]. In contrast to Rule 2, this rule sets a cap on SARS.CoV.2.S1.mFcTag levels, indicating an overall decrease. This helps rule out those vaccinated for COVID-19 within the past 180 days. Similarly, higher predicted SARS.CoV.2.S1.mFcTag and SARS.CoV.2.S1.RBD.mFc levels in this rule indicate the vaccine stimulates lasting anti-S1/RBD antibodies, effectively distinguishing unvaccinated individuals.

### 4.4. Limitations of this Study

There are some limitations in this study. First, several machine learning algorithms, including feature selection and classification algorithms, were adopted. The selection of essential antigens relied highly on the performance of the classification algorithms. It is known that an efficient classifier may not adopt two similar features. If these two features were all essential antigens, one would be omitted, i.e., some essential antigens may not be detected by our machine learning based framework. Second, a major limitation of microarray is the limited antibody coverage, which means only specific antibodies can be measured according to the predefined set of antigens on the array surface. Further study is required to take more COVID-19-related antibodies into consideration. Finally, the main purpose of this study was to discover essential antigens that were highly related to the classification of healthcare workers or one class, rather than to develop a machine learning classifier. Therefore, no test/train split was conducted on the dataset, and so accuracy metrics reported here should be considered as unvalidated in either an independent or test set.

## 5. Conclusions

Combining data on serum antibody levels in volunteers after COVID-19 vaccination and advanced machine learning methods, a set of antigen-reactive antibodies were extracted, which could reveal the effect of the vaccine on antiviral immune activation and reflect changes in antibody levels in the body over time after vaccination. In the computational framework, four efficient feature selecting algorithms, namely, LASSO, LightGBM, MCFS, and mRMR, were used to rank the features according to their contributions to the classification. Then, through the IFS method, the optimal features for four classification algorithms (DT, KNN, RF, SVM) in each feature list were confirmed. Subsequently, the overlapping features were identified by taking the intersection of the optimal feature subsets corresponding to the four feature selection algorithms, such as SARS.CoV.2.S1.mFcTag, SARS.CoV.2.Spike.RBD.His.Bac, and SARS.CoV.2.S1 + S2. Meanwhile, we determined the specific features that were highly related to one class. In addition, classification rules were constructed, which can quantitatively explain the important roles of features in the classification. Our findings have the potential to improve vaccine efficacy assessment and enable personalized vaccination strategies, ultimately contributing to more effective public health measures against COVID-19 and similar viral outbreaks.

## Figures and Tables

**Table 1:** Performance of optimal classifiers on different classification algorithms and feature lists.

Feature List	Classification Algorithm	Number of Features	ACC	MCC	Macro F1	Weighted F1
LASSO feature list	DT	47	0.704	0.554	0.744	0.702
	KNN	73	0.716	0.574	0.776	0.711
	RF	21	0.741	0.622	0.787	0.735
	SVM	73	0.737	0.603	0.796	0.733
LightGBM feature list	DT	40	0.720	0.573	0.762	0.717
	KNN	18	0.747	0.618	0.802	0.744
	RF	31	0.752	0.649	0.796	0.742
	SVM	35	0.761	0.640	0.806	0.758
MCFS feature list	DT	17	0.729	0.589	0.771	0.727
	KNN	20	0.742	0.611	0.799	0.739
	RF	23	0.756	0.649	0.801	0.747
	SVM	41	0.768	0.652	0.811	0.765
mRMR feature list	DT	14	0.730	0.594	0.763	0.728
	KNN	24	0.741	0.612	0.797	0.737
	RF	26	0.754	0.646	0.797	0.745
	SVM	30	0.762	0.643	0.805	0.758

**Table 2:** Top antigens identified by computational methods (The symbol ‘✔’ indicates that the antigen was identified by the corresponding method).

Target Antigens	LASSO	LightGBM	MCFS	mRMR
SARS.CoV.2.S1.mFcTag	✔	✔	✔	✔
MERS.CoV.S1.RBD.367.606.rFcTag	✔	✔	✔	✔
SARS.CoV.2.Spike.RBD.His.Bac	✔	✔	✔	✔
SARS.CoV.S1.HisTag	✔	✔	✔	✔
SARS.CoV.2.S1.RBD.mFc	✔	✔	✔	✔
SARS.CoV.2.S1 + S2	✔	✔	✔	✔
SARS.CoV.2.S2		✔	✔	✔
hCoV.HKU1.NP		✔	✔	✔
SARS.CoV.2.Spike.RBD.rFc	✔		✔	✔
SARS.CoV.2.S1	✔		✔	✔
SARS.CoV.2.S1.HisTag	✔		✔	✔
SARS.CoV.S1.RBD.HisTag	✔		✔	✔
hCoV.229E.S1	✔	✔	✔	

**Table 3:** Representative Rules.

Rules	Criteria	Predicted Class (Days after Vaccination)
Rule 0	SARS.CoV.2.S1.mFcTag ≤ 5354.39	Unvaccinated healthcare workers
	−383.87 < SARS.CoV.2.S1.HisTag	
	−414.30 < SARS.CoV.2.S1.RBD.mFc ≤ 3773.83	
	414.54 < hCoV.OC43.HE	
Rule 1	SARS.CoV.2.S1.mFcTag ≤ 54,010.17	Healthcare workers within 60 days after vaccination
	37,653.75 < SARS.CoV.2.S2	
	48,882.58 < SARS.CoV.2.S1 + S2	
Rule 2	5354.39 < SARS.CoV.2.S1.mFcTag	Healthcare workers between 60 and 180 days after vaccination
	3773.83 < SARS.CoV.2.S1.RBD.mFc ≤ 33,656.48	
	400.30 < SARS.CoV.S1.HisTag ≤ 15,087.42	
Rule 3	5354.39 < SARS.CoV.2.S1.mFcTag ≤ 34,194.92	Healthcare workers over 180 days after vaccination
	3773.83 < SARS.CoV.2.S1.RBD.mFc	

## Data Availability

The data presented in this study are openly available in Gene Expression Omnibus database, reference number [32].

## Supplementary Materials

The following supporting information can be downloaded at: https://www.mdpi.com/article/10.3390/life13061304/s1, Table S1: Feature names and their descriptions; Table S2: Feature lists obtained by LASSO, LightGBM, MCFS, and mRMR methods; Table S3: IFS results with different classification algorithms on four feature lists; Table S4: Intersection results of the optimal feature subsets identified by LASSO, LightGBM, MCFS, and mRMR methods; Table S5: Results of the intersection of top 20 features identified by LASSO, LightGBM, MCFS, and mRMR methods for each class; Table S6: Classification rules generated by the optimal DT classifiers on different feature lists.
