# Supplementary material for: Machine Learning Classification of Time since BNT162b2 COVID-19 Vaccination Based on Array-Measured Antibody Activity
Source: Life (Basel). 2023 May 31;13(6):1304. doi: 10.3390/life13061304 (PMC10305362; doi:10.3390/life13061304)
Supplement: Supplementary file 1 [file life-13-01304-s001.zip › Table S6.pdf]

Table S6. Classification rules generated by the optimal DT classifiers on different feature lists.

(1) Rules on the LASSO feature list

Rules_0	passed counts:247
node_0: feature_name=HuIgG_0.03	feature_id[44].value <= threshold=3298.333251953125
node_1: feature_name=SARS.CoV.2.S1.mFcTag	feature_id[0].value > threshold=5354.388916015625
node_9: feature_name=HuIgM_0.03	feature_id[45].value > threshold=-80.66666793823242
node_27: feature_name=HuIgM_0.03	feature_id[45].value <= threshold=61.33333206176758
node_28: feature_name=a-HuIgG_0.30	feature_id[30].value <= threshold=34878.66796875
node_29: feature_name=HuIgG_0.03	feature_id[44].value > threshold=769.8333435058594
node_35: feature_name=SARS.CoV.S1.HisTag	feature_id[14].value > threshold=400.3000030517578
node_39: feature_name=hCoV.HKU1.S1_S2	feature_id[22].value <= threshold=42662.666015625
node_40: feature_name=Flu.B_Phu/.HA1	feature_id[11].value <= threshold=49118.416015625
node_41: feature_name=hCoV.OC43.HE	feature_id[10].value <= threshold=25184.06640625
node_42: feature_name=HuIgM_0.30	feature_id[13].value <= threshold=1223.5
node_43: feature_name=a-HuIgA_0.03	feature_id[29].value > threshold=-59.66666603088379
node_47: feature_name=SARS.CoV.S1.HisTag	feature_id[14].value <= threshold=25028.0
node_48: feature_name=HuIgM_0.03	feature_id[45].value <= threshold=35.33333206176758
Class: healthcare workers between 60 and 180 days after vaccination	
Rules_1	passed counts:77
node_0: feature_name=HuIgG_0.03	feature_id[44].value > threshold=3298.333251953125
node_78: feature_name=a-HuIgG_0.03	feature_id[1].value <= threshold=19665.666015625
node_79: feature_name=SARS.CoV.2.S1.mFcTag	feature_id[0].value <= threshold=54010.166015625
node_80: feature_name=SARS.CoV.2.S1+S2	feature_id[18].value > threshold=48317.708984375

node_224: feature_name=a-MoIgG_0.10	feature_id[38].value > threshold=2066.8333129882812
node_226: feature_name=HuIgM_0.30	feature_id[13].value <= threshold=750.1666870117188
node_227: feature_name=SARS.CoV.S1.RBD.HisTag	feature_id[20].value > threshold=7585.25
node_233: feature_name=SARS.CoV.2.S1.HisTag	feature_id[16].value > threshold=29169.625
node_235: feature_name=a-MoIgA_0.03	feature_id[37].value > threshold=-137.16666412353516
Class: healthcare workers within 60 days after vaccination	
Rules_2	passed counts:74
node_0: feature_name=HuIgG_0.03	feature_id[44].value <= threshold=3298.333251953125
node_1: feature_name=SARS.CoV.2.S1.mFcTag	feature_id[0].value <= threshold=5354.388916015625
node_2: feature_name=SARS.CoV.2.S1.HisTag	feature_id[16].value > threshold=-383.86944580078125
node_4: feature_name=hCoV.OC43.HE	feature_id[10].value > threshold=414.5370330810547
Class: unvaccinated healthcare workers	
Rules_3	passed counts:59
node_0: feature_name=HuIgG_0.03	feature_id[44].value > threshold=3298.333251953125
node_78: feature_name=a-HuIgG_0.03	feature_id[1].value <= threshold=19665.666015625
node_79: feature_name=SARS.CoV.2.S1.mFcTag	feature_id[0].value <= threshold=54010.166015625
node_80: feature_name=SARS.CoV.2.S1+S2	feature_id[18].value > threshold=48317.708984375
node_224: feature_name=a-MoIgG_0.10	feature_id[38].value > threshold=2066.8333129882812
node_226: feature_name=HuIgM_0.30	feature_id[13].value > threshold=750.1666870117188
node_238: feature_name=HuIgM_0.30	feature_id[13].value > threshold=760.3333435058594
node_240: feature_name=hCoV.OC43.HE	feature_id[10].value > threshold=3879.75
node_246: feature_name=a-HuIgA_0.30	feature_id[27].value > threshold=1379.8333740234375
node_248: feature_name=Flu.H1N1.HA1	feature_id[26].value <= threshold=53891.083984375

node_249: feature_name=MERS.CoV.S1.RBD.367.606.rFcTag	feature_id[3].value > threshold=1486.7666625976562
node_253: feature_name=HuIgM_0.03	feature_id[45].value > threshold=-56.33333396911621
node_279: feature_name=hCoV.229E.S1	feature_id[2].value <= threshold=51743.583984375
node_280: feature_name=Flu.B_Mal/.HA1	feature_id[4].value <= threshold=48206.833984375
node_281: feature_name=a-MoIgM_0.10	feature_id[40].value > threshold=989.5000305175781
node_285: feature_name=HuIgM_0.30	feature_id[13].value <= threshold=2008.3333129882812
Class: healthcare workers within 60 days after vaccination	
Rules_4	passed counts:41
node_0: feature_name=HuIgG_0.03	feature_id[44].value > threshold=3298.333251953125
node_78: feature_name=a-HuIgG_0.03	feature_id[1].value <= threshold=19665.666015625
node_79: feature_name=SARS.CoV.2.S1.mFcTag	feature_id[0].value <= threshold=54010.166015625
node_80: feature_name=SARS.CoV.2.S1+S2	feature_id[18].value <= threshold=48317.708984375
node_81: feature_name=SARS.CoV.2.Spike.RBD.His.Bac	feature_id[9].value > threshold=16347.4580078125
node_111: feature_name=hCoV.NL63.S1	feature_id[5].value <= threshold=4866.533447265625
node_112: feature_name=SARS.CoV.2.S1.mFcTag	feature_id[0].value > threshold=40690.80078125
node_116: feature_name=a-MoIgG_0.10	feature_id[38].value > threshold=2424.6666259765625
node_118: feature_name=DcCoV.HKU23.NP	feature_id[21].value > threshold=3458.25
node_120: feature_name=hCoV.OC43.S1_S2ECD.HisTag	feature_id[24].value <= threshold=48433.232421875
Class: healthcare workers within 60 days after vaccination	
Rules_5	passed counts:38
node_0: feature_name=HuIgG_0.03	feature_id[44].value > threshold=3298.333251953125
node_78: feature_name=a-HuIgG_0.03	feature_id[1].value <= threshold=19665.666015625
node_79: feature_name=SARS.CoV.2.S1.mFcTag	feature_id[0].value <= threshold=54010.166015625

node_80: feature_name=SARS.CoV.2.S1+S2	feature_id[18].value <= threshold=48317.708984375
node_81: feature_name=SARS.CoV.2.Spike.RBD.His.Bac	feature_id[9].value > threshold=16347.4580078125
node_111: feature_name=hCoV.NL63.S1	feature_id[5].value > threshold=4866.533447265625
node_123: feature_name=MERS.CoV.S1.RBD.367.606.rFcTag	feature_id[3].value > threshold=2789.3416748046875
node_161: feature_name=SARS.CoV.S1.HisTag	feature_id[14].value <= threshold=4926.39990234375
node_162: feature_name=a-MoIgA_0.10	feature_id[36].value <= threshold=3108.3333740234375
node_163: feature_name=Flu.B_Mal/.HA1+HA2	feature_id[25].value > threshold=38684.7265625
node_167: feature_name=SARS.CoV.NP	feature_id[19].value <= threshold=18293.6826171875
node_168: feature_name=a-HuIgG_0.03	feature_id[1].value > threshold=10603.0
node_172: feature_name=a-HuIgM_0.30	feature_id[32].value <= threshold=29969.8330078125
Class: healthcare workers between 60 and 180 days after vaccination	
Rules_6	passed counts:36
node_0: feature_name=HuIgG_0.03	feature_id[44].value > threshold=3298.333251953125
node_78: feature_name=a-HuIgG_0.03	feature_id[1].value <= threshold=19665.666015625
node_79: feature_name=SARS.CoV.2.S1.mFcTag	feature_id[0].value <= threshold=54010.166015625
node_80: feature_name=SARS.CoV.2.S1+S2	feature_id[18].value > threshold=48317.708984375
node_224: feature_name=a-MoIgG_0.10	feature_id[38].value > threshold=2066.8333129882812
node_226: feature_name=HuIgM_0.30	feature_id[13].value > threshold=750.1666870117188
node_238: feature_name=HuIgM_0.30	feature_id[13].value > threshold=760.3333435058594
node_240: feature_name=hCoV.OC43.HE	feature_id[10].value > threshold=3879.75
node_246: feature_name=a-HuIgA_0.30	feature_id[27].value > threshold=1379.8333740234375
node_248: feature_name=Flu.H1N1.HA1	feature_id[26].value <= threshold=53891.083984375
node_249: feature_name=MERS.CoV.S1.RBD.367.606.rFcTag	feature_id[3].value > threshold=1486.7666625976562
node_253: feature_name=HuIgM_0.03	feature_id[45].value <= threshold=-56.33333396911621

node_254: feature_name=MoIgA_0.30	feature_id[46].value > threshold=900.1666564941406
node_258: feature_name=SARS.CoV.2.S1.mFcTag	feature_id[0].value <= threshold=53346.333984375
node_259: feature_name=SARS.CoV.2.S1	feature_id[15].value > threshold=47301.333984375
Class: healthcare workers within 60 days after vaccination	
Rules_7	passed counts:33
node_0: feature_name=HuIgG_0.03	feature_id[44].value <= threshold=3298.333251953125
node_1: feature_name=SARS.CoV.2.S1.mFcTag	feature_id[0].value > threshold=5354.388916015625
node_9: feature_name=HuIgM_0.03	feature_id[45].value <= threshold=-80.66666793823242
node_10: feature_name=SARS.CoV.2.Spike.RBD.His.Bac	feature_id[9].value <= threshold=29393.55859375
node_11: feature_name=DcCoV.HKU23.NP	feature_id[21].value > threshold=-639.3333129882812
node_13: feature_name=MERS.CoV.S1.ECD.1-1297.HisTag	feature_id[12].value > threshold=1026.1666564941406
Class: healthcare workers over 180 days after vaccination	
Rules_8	passed counts:30
node_0: feature_name=HuIgG_0.03	feature_id[44].value > threshold=3298.333251953125
node_78: feature_name=a-HuIgG_0.03	feature_id[1].value <= threshold=19665.666015625
node_79: feature_name=SARS.CoV.2.S1.mFcTag	feature_id[0].value <= threshold=54010.166015625
node_80: feature_name=SARS.CoV.2.S1+S2	feature_id[18].value <= threshold=48317.708984375
node_81: feature_name=SARS.CoV.2.Spike.RBD.His.Bac	feature_id[9].value <= threshold=16347.4580078125
node_82: feature_name=SARS.CoV.2.S1	feature_id[15].value > threshold=2844.6334228515625
node_90: feature_name=a-MoIgG_0.10	feature_id[38].value > threshold=1482.3333129882812
node_98: feature_name=Flu.H1N1.HA1	feature_id[26].value <= threshold=52934.66015625
node_99: feature_name=Flu.B_Phu/.HA1	feature_id[11].value > threshold=27915.5419921875
node_105: feature_name=SARS.CoV.NP	feature_id[19].value > threshold=310.9027671813965

Class: healthcare workers within 60 days after vaccination	
Rules_9	passed counts:27
node_0: feature_name=HuIgG_0.03	feature_id[44].value > threshold=3298.333251953125
node_78: feature_name=a-HuIgG_0.03	feature_id[1].value <= threshold=19665.666015625
node_79: feature_name=SARS.CoV.2.S1.mFcTag	feature_id[0].value <= threshold=54010.166015625
node_80: feature_name=SARS.CoV.2.S1+S2	feature_id[18].value <= threshold=48317.708984375
node_81: feature_name=SARS.CoV.2.Spike.RBD.His.Bac	feature_id[9].value > threshold=16347.4580078125
node_111: feature_name=hCoV.NL63.S1	feature_id[5].value > threshold=4866.533447265625
node_123: feature_name=MERS.CoV.S1.RBD.367.606.rFcTag	feature_id[3].value > threshold=2789.3416748046875
node_161: feature_name=SARS.CoV.S1.HisTag	feature_id[14].value > threshold=4926.39990234375
node_179: feature_name=hCoV.HKU1.S1_S2	feature_id[22].value <= threshold=48084.783203125
node_180: feature_name=SARS.CoV.2.Spike.RBD.His.Bac	feature_id[9].value > threshold=31607.125
node_200: feature_name=SARS.CoV.2.S1.HisTag	feature_id[16].value <= threshold=43902.201171875
node_201: feature_name=Flu.H1N1.HA1+HA2	feature_id[6].value > threshold=30743.5
node_205: feature_name=Flu.B_Phu/.HA1	feature_id[11].value > threshold=18442.14111328125
node_207: feature_name=SARS.CoV.2.Spike.RBD.rFc	feature_id[7].value > threshold=39119.033203125
node_209: feature_name=MERS.CoV.S1.ECD.1-1297.HisTag	feature_id[12].value > threshold=11020.70849609375
Class: healthcare workers between 60 and 180 days after vaccination	
Rules_10	passed counts:26
node_0: feature_name=HuIgG_0.03	feature_id[44].value > threshold=3298.333251953125
node_78: feature_name=a-HuIgG_0.03	feature_id[1].value > threshold=19665.666015625
node_360: feature_name=HuIgM_0.03	feature_id[45].value > threshold=-189.0
node_366: feature_name=HuIgM_0.30	feature_id[13].value > threshold=441.8333282470703

node_368: feature_name=a-HuIgG_0.10	feature_id[31].value > threshold=32914.5
node_372: feature_name=a-MoIgG_0.10	feature_id[38].value <= threshold=20281.6669921875
node_373: feature_name=hCoV.229E.S1	feature_id[2].value <= threshold=54372.5
Class: healthcare workers over 180 days after vaccination	
Rules_11	passed counts:25
node_0: feature_name=HuIgG_0.03	feature_id[44].value > threshold=3298.333251953125
node_78: feature_name=a-HuIgG_0.03	feature_id[1].value <= threshold=19665.666015625
node_79: feature_name=SARS.CoV.2.S1.mFcTag	feature_id[0].value > threshold=54010.166015625
node_297: feature_name=a-MoIgG_0.10	feature_id[38].value > threshold=2382.8333740234375
node_303: feature_name=SARS.CoV.2.S1	feature_id[15].value <= threshold=51713.66796875
node_304: feature_name=hCoV.HKU1.S1_AA1.760	feature_id[8].value > threshold=16192.1669921875
node_310: feature_name=a-HuIgM_0.10	feature_id[33].value > threshold=176.5
node_314: feature_name=SARS.CoV.2.S1+S2	feature_id[18].value > threshold=50299.93359375
node_318: feature_name=a-HuIgG_0.30	feature_id[30].value > threshold=47587.833984375
node_336: feature_name=hCoV.HKU1.S1_AA1.760	feature_id[8].value <= threshold=45964.3828125
node_337: feature_name=SARS.CoV.2.S1+S2	feature_id[18].value > threshold=50700.5
node_339: feature_name=a-MoIgA_0.30	feature_id[35].value > threshold=1534.1666259765625
Class: healthcare workers between 60 and 180 days after vaccination	
Rules_12	passed counts:23
node_0: feature_name=HuIgG_0.03	feature_id[44].value > threshold=3298.333251953125
node_78: feature_name=a-HuIgG_0.03	feature_id[1].value <= threshold=19665.666015625
node_79: feature_name=SARS.CoV.2.S1.mFcTag	feature_id[0].value > threshold=54010.166015625
node_297: feature_name=a-MoIgG_0.10	feature_id[38].value > threshold=2382.8333740234375

node_303: feature_name=SARS.CoV.2.S1	feature_id[15].value <= threshold=51713.66796875
node_304: feature_name=hCoV.HKU1.S1_AA1.760	feature_id[8].value > threshold=16192.1669921875
node_310: feature_name=a-HuIgM_0.10	feature_id[33].value > threshold=176.5
node_314: feature_name=SARS.CoV.2.S1+S2	feature_id[18].value <= threshold=50299.93359375
node_315: feature_name=a-HuIgA_0.03	feature_id[29].value > threshold=-68.0
Class: healthcare workers between 60 and 180 days after vaccination	
Rules_13	passed counts:23
node_0: feature_name=HuIgG_0.03	feature_id[44].value > threshold=3298.333251953125
node_78: feature_name=a-HuIgG_0.03	feature_id[1].value <= threshold=19665.666015625
node_79: feature_name=SARS.CoV.2.S1.mFcTag	feature_id[0].value <= threshold=54010.166015625
node_80: feature_name=SARS.CoV.2.S1+S2	feature_id[18].value <= threshold=48317.708984375
node_81: feature_name=SARS.CoV.2.Spike.RBD.His.Bac	feature_id[9].value > threshold=16347.4580078125
node_111: feature_name=hCoV.NL63.S1	feature_id[5].value > threshold=4866.533447265625
node_123: feature_name=MERS.CoV.S1.RBD.367.606.rFcTag	feature_id[3].value <= threshold=2789.3416748046875
node_124: feature_name=SARS.CoV.2.S1+S2	feature_id[18].value > threshold=41798.25
node_140: feature_name=SARS.CoV.2.S1.RBD.mFc	feature_id[17].value > threshold=44491.166015625
Class: healthcare workers within 60 days after vaccination	
Rules_14	passed counts:22
node_0: feature_name=HuIgG_0.03	feature_id[44].value > threshold=3298.333251953125
node_78: feature_name=a-HuIgG_0.03	feature_id[1].value <= threshold=19665.666015625
node_79: feature_name=SARS.CoV.2.S1.mFcTag	feature_id[0].value <= threshold=54010.166015625
node_80: feature_name=SARS.CoV.2.S1+S2	feature_id[18].value > threshold=48317.708984375
node_224: feature_name=a-MoIgG_0.10	feature_id[38].value > threshold=2066.8333129882812

node_226: feature_name=HuIgM_0.30	feature_id[13].value > threshold=750.1666870117188
node_238: feature_name=HuIgM_0.30	feature_id[13].value > threshold=760.3333435058594
node_240: feature_name=hCoV.OC43.HE	feature_id[10].value > threshold=3879.75
node_246: feature_name=a-HuIgA_0.30	feature_id[27].value > threshold=1379.8333740234375
node_248: feature_name=Flu.H1N1.HA1	feature_id[26].value <= threshold=53891.083984375
node_249: feature_name=MERS.CoV.S1.RBD.367.606.rFcTag	feature_id[3].value > threshold=1486.7666625976562
node_253: feature_name=HuIgM_0.03	feature_id[45].value <= threshold=-56.33333396911621
node_254: feature_name=MoIgA_0.30	feature_id[46].value > threshold=900.1666564941406
node_258: feature_name=SARS.CoV.2.S1.mFcTag	feature_id[0].value <= threshold=53346.333984375
node_259: feature_name=SARS.CoV.2.S1	feature_id[15].value <= threshold=47301.333984375
node_260: feature_name=a-MoIgM_0.03	feature_id[41].value > threshold=127.16666793823242
node_262: feature_name=hCoV.OC43.HE	feature_id[10].value > threshold=12272.5166015625
node_268: feature_name=HuIgM_0.03	feature_id[45].value <= threshold=-57.16666603088379
Class: healthcare workers within 60 days after vaccination	
Rules_15	passed counts:22
node_0: feature_name=HuIgG_0.03	feature_id[44].value > threshold=3298.333251953125
node_78: feature_name=a-HuIgG_0.03	feature_id[1].value <= threshold=19665.666015625
node_79: feature_name=SARS.CoV.2.S1.mFcTag	feature_id[0].value <= threshold=54010.166015625
node_80: feature_name=SARS.CoV.2.S1+S2	feature_id[18].value <= threshold=48317.708984375
node_81: feature_name=SARS.CoV.2.Spike.RBD.His.Bac	feature_id[9].value > threshold=16347.4580078125
node_111: feature_name=hCoV.NL63.S1	feature_id[5].value > threshold=4866.533447265625
node_123: feature_name=MERS.CoV.S1.RBD.367.606.rFcTag	feature_id[3].value <= threshold=2789.3416748046875
node_124: feature_name=SARS.CoV.2.S1+S2	feature_id[18].value > threshold=41798.25
node_140: feature_name=SARS.CoV.2.S1.RBD.mFc	feature_id[17].value <= threshold=44491.166015625

node_141: feature_name=a-HuIgM_0.03	feature_id[34].value > threshold=-6.833333492279053
node_149: feature_name=a-MoIgA_0.30	feature_id[35].value > threshold=935.3333435058594
node_151: feature_name=HuIgM_0.30	feature_id[13].value <= threshold=1110.5
node_152: feature_name=a-HuIgG_0.30	feature_id[30].value <= threshold=46032.0
Class: healthcare workers within 60 days after vaccination	
Rules_16	passed counts:21
node_0: feature_name=HuIgG_0.03	feature_id[44].value > threshold=3298.333251953125
node_78: feature_name=a-HuIgG_0.03	feature_id[1].value <= threshold=19665.666015625
node_79: feature_name=SARS.CoV.2.S1.mFcTag	feature_id[0].value > threshold=54010.166015625
node_297: feature_name=a-MoIgG_0.10	feature_id[38].value > threshold=2382.8333740234375
node_303: feature_name=SARS.CoV.2.S1	feature_id[15].value > threshold=51713.66796875
node_345: feature_name=SARS.CoV.2.S1.HisTag	feature_id[16].value > threshold=51766.75
node_347: feature_name=HuIgA_0.03	feature_id[43].value > threshold=-60.33333396911621
node_353: feature_name=SARS.CoV.2.Spike.RBD.His.Bac	feature_id[9].value <= threshold=51551.166015625
node_354: feature_name=SARS.CoV.NP	feature_id[19].value > threshold=3082.949951171875
Class: healthcare workers within 60 days after vaccination	
Rules_17	passed counts:19
node_0: feature_name=HuIgG_0.03	feature_id[44].value > threshold=3298.333251953125
node_78: feature_name=a-HuIgG_0.03	feature_id[1].value <= threshold=19665.666015625
node_79: feature_name=SARS.CoV.2.S1.mFcTag	feature_id[0].value <= threshold=54010.166015625
node_80: feature_name=SARS.CoV.2.S1+S2	feature_id[18].value <= threshold=48317.708984375
node_81: feature_name=SARS.CoV.2.Spike.RBD.His.Bac	feature_id[9].value > threshold=16347.4580078125
node_111: feature_name=hCoV.NL63.S1	feature_id[5].value > threshold=4866.533447265625

node_123: feature_name=MERS.CoV.S1.RBD.367.606.rFcTag	feature_id[3].value > threshold=2789.3416748046875
node_161: feature_name=SARS.CoV.S1.HisTag	feature_id[14].value > threshold=4926.39990234375
node_179: feature_name=hCoV.HKU1.S1_S2	feature_id[22].value > threshold=48084.783203125
node_217: feature_name=MoIgA_0.30	feature_id[46].value <= threshold=24299.6669921875
node_218: feature_name=DcCoV.HKU23.NP	feature_id[21].value > threshold=20749.25
Class: healthcare workers within 60 days after vaccination	
Rules_18	passed counts:18
node_0: feature_name=HuIgG_0.03	feature_id[44].value > threshold=3298.333251953125
node_78: feature_name=a-HuIgG_0.03	feature_id[1].value <= threshold=19665.666015625
node_79: feature_name=SARS.CoV.2.S1.mFcTag	feature_id[0].value <= threshold=54010.166015625
node_80: feature_name=SARS.CoV.2.S1+S2	feature_id[18].value <= threshold=48317.708984375
node_81: feature_name=SARS.CoV.2.Spike.RBD.His.Bac	feature_id[9].value > threshold=16347.4580078125
node_111: feature_name=hCoV.NL63.S1	feature_id[5].value > threshold=4866.533447265625
node_123: feature_name=MERS.CoV.S1.RBD.367.606.rFcTag	feature_id[3].value > threshold=2789.3416748046875
node_161: feature_name=SARS.CoV.S1.HisTag	feature_id[14].value > threshold=4926.39990234375
node_179: feature_name=hCoV.HKU1.S1_S2	feature_id[22].value <= threshold=48084.783203125
node_180: feature_name=SARS.CoV.2.Spike.RBD.His.Bac	feature_id[9].value <= threshold=31607.125
node_181: feature_name=SARS.CoV.2.S1	feature_id[15].value > threshold=33718.333984375
node_191: feature_name=a-HuIgA_0.30	feature_id[27].value > threshold=1819.5
node_193: feature_name=HuIgM_0.30	feature_id[13].value <= threshold=1081.8333740234375
node_194: feature_name=hCoV.NL63.S1	feature_id[5].value > threshold=6020.75830078125
Class: healthcare workers within 60 days after vaccination	
Rules_19	passed counts:17

node_0: feature_name=HuIgG_0.03	feature_id[44].value > threshold=3298.333251953125
node_78: feature_name=a-HuIgG_0.03	feature_id[1].value <= threshold=19665.666015625
node_79: feature_name=SARS.CoV.2.S1.mFcTag	feature_id[0].value > threshold=54010.166015625
node_297: feature_name=a-MoIgG_0.10	feature_id[38].value > threshold=2382.8333740234375
node_303: feature_name=SARS.CoV.2.S1	feature_id[15].value <= threshold=51713.66796875
node_304: feature_name=hCoV.HKU1.S1_AA1.760	feature_id[8].value > threshold=16192.1669921875
node_310: feature_name=a-HuIgM_0.10	feature_id[33].value > threshold=176.5
node_314: feature_name=SARS.CoV.2.S1+S2	feature_id[18].value > threshold=50299.93359375
node_318: feature_name=a-HuIgG_0.30	feature_id[30].value <= threshold=47587.833984375
node_319: feature_name=MERS.CoV.S1.RBD.367.606.rFcTag	feature_id[3].value > threshold=3945.61669921875
node_323: feature_name=a-MoIgG_0.10	feature_id[38].value <= threshold=6842.166748046875
node_324: feature_name=a-HuIgA_0.10	feature_id[28].value <= threshold=2405.8333740234375
Class: healthcare workers between 60 and 180 days after vaccination	
Rules_20	passed counts:17
node_0: feature_name=HuIgG_0.03	feature_id[44].value <= threshold=3298.333251953125
node_1: feature_name=SARS.CoV.2.S1.mFcTag	feature_id[0].value > threshold=5354.388916015625
node_9: feature_name=HuIgM_0.03	feature_id[45].value > threshold=-80.66666793823242
node_27: feature_name=HuIgM_0.03	feature_id[45].value > threshold=61.33333206176758
node_75: feature_name=HuIgA_0.10	feature_id[42].value <= threshold=1386.3333435058594
Class: healthcare workers over 180 days after vaccination	
Rules_21	passed counts:15
node_0: feature_name=HuIgG_0.03	feature_id[44].value > threshold=3298.333251953125
node_78: feature_name=a-HuIgG_0.03	feature_id[1].value <= threshold=19665.666015625

node_79: feature_name=SARS.CoV.2.S1.mFcTag	feature_id[0].value <= threshold=54010.166015625
node_80: feature_name=SARS.CoV.2.S1+S2	feature_id[18].value <= threshold=48317.708984375
node_81: feature_name=SARS.CoV.2.Spike.RBD.His.Bac	feature_id[9].value > threshold=16347.4580078125
node_111: feature_name=hCoV.NL63.S1	feature_id[5].value > threshold=4866.533447265625
node_123: feature_name=MERS.CoV.S1.RBD.367.606.rFcTag	feature_id[3].value <= threshold=2789.3416748046875
node_124: feature_name=SARS.CoV.2.S1+S2	feature_id[18].value <= threshold=41798.25
node_125: feature_name=hCoV.OC43.S1_S2ECD.HisTag	feature_id[24].value <= threshold=49554.1328125
node_126: feature_name=SARS.CoV.2.Spike.RBD.His.Bac	feature_id[9].value > threshold=23295.4169921875
node_130: feature_name=Flu.H1N1.HA1	feature_id[26].value > threshold=30553.0
node_134: feature_name=MERS.CoV.S1.RBD.367.606.rFcTag	feature_id[3].value > threshold=983.2499694824219
node_136: feature_name=a-MoIgA_0.03	feature_id[37].value <= threshold=503.50001525878906
Class: healthcare workers between 60 and 180 days after vaccination	
Rules_22	passed counts:13
node_0: feature_name=HuIgG_0.03	feature_id[44].value > threshold=3298.333251953125
node_78: feature_name=a-HuIgG_0.03	feature_id[1].value <= threshold=19665.666015625
node_79: feature_name=SARS.CoV.2.S1.mFcTag	feature_id[0].value > threshold=54010.166015625
node_297: feature_name=a-MoIgG_0.10	feature_id[38].value > threshold=2382.8333740234375
node_303: feature_name=SARS.CoV.2.S1	feature_id[15].value <= threshold=51713.66796875
node_304: feature_name=hCoV.HKU1.S1_AA1.760	feature_id[8].value > threshold=16192.1669921875
node_310: feature_name=a-HuIgM_0.10	feature_id[33].value > threshold=176.5
node_314: feature_name=SARS.CoV.2.S1+S2	feature_id[18].value > threshold=50299.93359375
node_318: feature_name=a-HuIgG_0.30	feature_id[30].value <= threshold=47587.833984375
node_319: feature_name=MERS.CoV.S1.RBD.367.606.rFcTag	feature_id[3].value <= threshold=3945.61669921875
node_320: feature_name=Flu.H1N1.HA1	feature_id[26].value <= threshold=52361.833984375

Class: healthcare workers within 60 days after vaccination	
Rules_23	passed counts:13
node_0: feature_name=HuIgG_0.03	feature_id[44].value > threshold=3298.333251953125
node_78: feature_name=a-HuIgG_0.03	feature_id[1].value <= threshold=19665.666015625
node_79: feature_name=SARS.CoV.2.S1.mFcTag	feature_id[0].value <= threshold=54010.166015625
node_80: feature_name=SARS.CoV.2.S1+S2	feature_id[18].value <= threshold=48317.708984375
node_81: feature_name=SARS.CoV.2.Spike.RBD.His.Bac	feature_id[9].value > threshold=16347.4580078125
node_111: feature_name=hCoV.NL63.S1	feature_id[5].value > threshold=4866.533447265625
node_123: feature_name=MERS.CoV.S1.RBD.367.606.rFcTag	feature_id[3].value > threshold=2789.3416748046875
node_161: feature_name=SARS.CoV.S1.HisTag	feature_id[14].value > threshold=4926.39990234375
node_179: feature_name=hCoV.HKU1.S1_S2	feature_id[22].value <= threshold=48084.783203125
node_180: feature_name=SARS.CoV.2.Spike.RBD.His.Bac	feature_id[9].value <= threshold=31607.125
node_181: feature_name=SARS.CoV.2.S1	feature_id[15].value <= threshold=33718.333984375
node_182: feature_name=a-HuIgG_0.03	feature_id[1].value <= threshold=15402.5
node_183: feature_name=a-HuIgM_0.30	feature_id[32].value > threshold=1969.0000610351562
node_185: feature_name=SARS.CoV.NP	feature_id[19].value > threshold=1277.2083435058594
Class: healthcare workers between 60 and 180 days after vaccination	
Rules_24	passed counts:13
node_0: feature_name=HuIgG_0.03	feature_id[44].value <= threshold=3298.333251953125
node_1: feature_name=SARS.CoV.2.S1.mFcTag	feature_id[0].value > threshold=5354.388916015625
node_9: feature_name=HuIgM_0.03	feature_id[45].value > threshold=-80.66666793823242
node_27: feature_name=HuIgM_0.03	feature_id[45].value <= threshold=61.33333206176758
node_28: feature_name=a-HuIgG_0.30	feature_id[30].value > threshold=34878.66796875

node_64: feature_name=a-HuIgG_0.03	feature_id[1].value > threshold=9340.3330078125
node_68: feature_name=a-HuIgA_0.30	feature_id[27].value > threshold=1918.1666870117188
node_72: feature_name=HuIgM_0.03	feature_id[45].value <= threshold=20.333333492279053
Class: healthcare workers over 180 days after vaccination	
Rules_25	passed counts:12
node_0: feature_name=HuIgG_0.03	feature_id[44].value > threshold=3298.333251953125
node_78: feature_name=a-HuIgG_0.03	feature_id[1].value <= threshold=19665.666015625
node_79: feature_name=SARS.CoV.2.S1.mFcTag	feature_id[0].value <= threshold=54010.166015625
node_80: feature_name=SARS.CoV.2.S1+S2	feature_id[18].value <= threshold=48317.708984375
node_81: feature_name=SARS.CoV.2.Spike.RBD.His.Bac	feature_id[9].value <= threshold=16347.4580078125
node_82: feature_name=SARS.CoV.2.S1	feature_id[15].value <= threshold=2844.6334228515625
node_83: feature_name=SARS.CoV.2.S1+S2	feature_id[18].value > threshold=3298.25
Class: unvaccinated healthcare workers	
Rules_26	passed counts:10
node_0: feature_name=HuIgG_0.03	feature_id[44].value > threshold=3298.333251953125
node_78: feature_name=a-HuIgG_0.03	feature_id[1].value <= threshold=19665.666015625
node_79: feature_name=SARS.CoV.2.S1.mFcTag	feature_id[0].value > threshold=54010.166015625
node_297: feature_name=a-MoIgG_0.10	feature_id[38].value > threshold=2382.8333740234375
node_303: feature_name=SARS.CoV.2.S1	feature_id[15].value <= threshold=51713.66796875
node_304: feature_name=hCoV.HKU1.S1_AA1.760	feature_id[8].value > threshold=16192.1669921875
node_310: feature_name=a-HuIgM_0.10	feature_id[33].value <= threshold=176.5
node_311: feature_name=a-HuIgM_0.03	feature_id[34].value > threshold=-86.66666793823242
Class: healthcare workers within 60 days after vaccination	

Rules_27	passed counts:9
node_0: feature_name=HuIgG_0.03	feature_id[44].value > threshold=3298.333251953125
node_78: feature_name=a-HuIgG_0.03	feature_id[1].value <= threshold=19665.666015625
node_79: feature_name=SARS.CoV.2.S1.mFcTag	feature_id[0].value > threshold=54010.166015625
node_297: feature_name=a-MoIgG_0.10	feature_id[38].value > threshold=2382.8333740234375
node_303: feature_name=SARS.CoV.2.S1	feature_id[15].value <= threshold=51713.66796875
node_304: feature_name=hCoV.HKU1.S1_AA1.760	feature_id[8].value > threshold=16192.1669921875
node_310: feature_name=a-HuIgM_0.10	feature_id[33].value > threshold=176.5
node_314: feature_name=SARS.CoV.2.S1+S2	feature_id[18].value > threshold=50299.93359375
node_318: feature_name=a-HuIgG_0.30	feature_id[30].value <= threshold=47587.833984375
node_319: feature_name=MERS.CoV.S1.RBD.367.606.rFcTag	feature_id[3].value > threshold=3945.61669921875
node_323: feature_name=a-MoIgG_0.10	feature_id[38].value > threshold=6842.166748046875
node_327: feature_name=SARS.CoV.2.S1.mFcTag	feature_id[0].value > threshold=54403.482421875
node_333: feature_name=Flu.B_Mal/.HA1+HA2	feature_id[25].value <= threshold=53690.8828125
Class: healthcare workers within 60 days after vaccination	
Rules_28	passed counts:9
node_0: feature_name=HuIgG_0.03	feature_id[44].value > threshold=3298.333251953125
node_78: feature_name=a-HuIgG_0.03	feature_id[1].value <= threshold=19665.666015625
node_79: feature_name=SARS.CoV.2.S1.mFcTag	feature_id[0].value > threshold=54010.166015625
node_297: feature_name=a-MoIgG_0.10	feature_id[38].value > threshold=2382.8333740234375
node_303: feature_name=SARS.CoV.2.S1	feature_id[15].value <= threshold=51713.66796875
node_304: feature_name=hCoV.HKU1.S1_AA1.760	feature_id[8].value <= threshold=16192.1669921875
node_305: feature_name=a-HuIgM_0.03	feature_id[34].value > threshold=20.666666984558105

Class: healthcare workers within 60 days after vaccination	
Rules_29	passed counts:9
node_0: feature_name=HuIgG_0.03	feature_id[44].value <= threshold=3298.333251953125
node_1: feature_name=SARS.CoV.2.S1.mFcTag	feature_id[0].value > threshold=5354.388916015625
node_9: feature_name=HuIgM_0.03	feature_id[45].value <= threshold=-80.66666793823242
node_10: feature_name=SARS.CoV.2.Spike.RBD.His.Bac	feature_id[9].value > threshold=29393.55859375
node_18: feature_name=Flu.B_Mal/.HA1	feature_id[4].value > threshold=38994.25
node_20: feature_name=SARS.CoV.S1.HisTag	feature_id[14].value > threshold=11923.35009765625
node_24: feature_name=SARS.CoV.2.S1.RBD.mFc	feature_id[17].value > threshold=44304.56640625
Class: healthcare workers over 180 days after vaccination	
Rules_30	passed counts:8
node_0: feature_name=HuIgG_0.03	feature_id[44].value > threshold=3298.333251953125
node_78: feature_name=a-HuIgG_0.03	feature_id[1].value > threshold=19665.666015625
node_360: feature_name=HuIgM_0.03	feature_id[45].value <= threshold=-189.0
node_361: feature_name=a-HuIgA_0.03	feature_id[29].value <= threshold=3214.5
Class: healthcare workers between 60 and 180 days after vaccination	
Rules_31	passed counts:8
node_0: feature_name=HuIgG_0.03	feature_id[44].value > threshold=3298.333251953125
node_78: feature_name=a-HuIgG_0.03	feature_id[1].value <= threshold=19665.666015625
node_79: feature_name=SARS.CoV.2.S1.mFcTag	feature_id[0].value <= threshold=54010.166015625
node_80: feature_name=SARS.CoV.2.S1+S2	feature_id[18].value > threshold=48317.708984375
node_224: feature_name=a-MoIgG_0.10	feature_id[38].value > threshold=2066.8333129882812

node_226: feature_name=HuIgM_0.30	feature_id[13].value > threshold=750.1666870117188
node_238: feature_name=HuIgM_0.30	feature_id[13].value > threshold=760.3333435058594
node_240: feature_name=hCoV.OC43.HE	feature_id[10].value > threshold=3879.75
node_246: feature_name=a-HuIgA_0.30	feature_id[27].value > threshold=1379.8333740234375
node_248: feature_name=Flu.H1N1.HA1	feature_id[26].value <= threshold=53891.083984375
node_249: feature_name=MERS.CoV.S1.RBD.367.606.rFcTag	feature_id[3].value > threshold=1486.7666625976562
node_253: feature_name=HuIgM_0.03	feature_id[45].value <= threshold=-56.33333396911621
node_254: feature_name=MoIgA_0.30	feature_id[46].value > threshold=900.1666564941406
node_258: feature_name=SARS.CoV.2.S1.mFcTag	feature_id[0].value > threshold=53346.333984375
node_272: feature_name=a-MoIgA_0.03	feature_id[37].value > threshold=2.3333334028720856
node_274: feature_name=Flu.B_Mal/.HA1+HA2	feature_id[25].value <= threshold=53981.70703125
node_275: feature_name=a-MoIgG_0.10	feature_id[38].value <= threshold=22321.6669921875
Class: healthcare workers between 60 and 180 days after vaccination	
Rules_32	passed counts:8
node_0: feature_name=HuIgG_0.03	feature_id[44].value > threshold=3298.333251953125
node_78: feature_name=a-HuIgG_0.03	feature_id[1].value <= threshold=19665.666015625
node_79: feature_name=SARS.CoV.2.S1.mFcTag	feature_id[0].value <= threshold=54010.166015625
node_80: feature_name=SARS.CoV.2.S1+S2	feature_id[18].value <= threshold=48317.708984375
node_81: feature_name=SARS.CoV.2.Spike.RBD.His.Bac	feature_id[9].value > threshold=16347.4580078125
node_111: feature_name=hCoV.NL63.S1	feature_id[5].value > threshold=4866.533447265625
node_123: feature_name=MERS.CoV.S1.RBD.367.606.rFcTag	feature_id[3].value <= threshold=2789.3416748046875
node_124: feature_name=SARS.CoV.2.S1+S2	feature_id[18].value > threshold=41798.25
node_140: feature_name=SARS.CoV.2.S1.RBD.mFc	feature_id[17].value <= threshold=44491.166015625
node_141: feature_name=a-HuIgM_0.03	feature_id[34].value <= threshold=-6.833333492279053

node_142: feature_name=hCoV.OC43.HE	feature_id[10].value > threshold=7956.5
node_144: feature_name=SARS.CoV.2.S1.mFcTag	feature_id[0].value <= threshold=52734.0
node_145: feature_name=Flu.H1N1.HA1	feature_id[26].value > threshold=26772.75
Class: healthcare workers between 60 and 180 days after vaccination	
Rules_33	passed counts:8
node_0: feature_name=HuIgG_0.03	feature_id[44].value <= threshold=3298.333251953125
node_1: feature_name=SARS.CoV.2.S1.mFcTag	feature_id[0].value > threshold=5354.388916015625
node_9: feature_name=HuIgM_0.03	feature_id[45].value > threshold=-80.66666793823242
node_27: feature_name=HuIgM_0.03	feature_id[45].value <= threshold=61.33333206176758
node_28: feature_name=a-HuIgG_0.30	feature_id[30].value > threshold=34878.66796875
node_64: feature_name=a-HuIgG_0.03	feature_id[1].value <= threshold=9340.3330078125
node_65: feature_name=SARS.CoV.2.Spike.RBD.His.Bac	feature_id[9].value > threshold=13221.6298828125
Class: healthcare workers within 60 days after vaccination	
Rules_34	passed counts:8
node_0: feature_name=HuIgG_0.03	feature_id[44].value <= threshold=3298.333251953125
node_1: feature_name=SARS.CoV.2.S1.mFcTag	feature_id[0].value > threshold=5354.388916015625
node_9: feature_name=HuIgM_0.03	feature_id[45].value <= threshold=-80.66666793823242
node_10: feature_name=SARS.CoV.2.Spike.RBD.His.Bac	feature_id[9].value > threshold=29393.55859375
node_18: feature_name=Flu.B_Mal/.HA1	feature_id[4].value <= threshold=38994.25
Class: healthcare workers between 60 and 180 days after vaccination	
Rules_35	passed counts:7
node_0: feature_name=HuIgG_0.03	feature_id[44].value > threshold=3298.333251953125

node_78: feature_name=a-HuIgG_0.03	feature_id[1].value <= threshold=19665.666015625
node_79: feature_name=SARS.CoV.2.S1.mFcTag	feature_id[0].value > threshold=54010.166015625
node_297: feature_name=a-MoIgG_0.10	feature_id[38].value <= threshold=2382.8333740234375
node_298: feature_name=Flu.B_Mal/.HA1	feature_id[4].value > threshold=41908.115234375
Class: healthcare workers over 180 days after vaccination	
Rules_36	passed counts:7
node_0: feature_name=HuIgG_0.03	feature_id[44].value > threshold=3298.333251953125
node_78: feature_name=a-HuIgG_0.03	feature_id[1].value <= threshold=19665.666015625
node_79: feature_name=SARS.CoV.2.S1.mFcTag	feature_id[0].value <= threshold=54010.166015625
node_80: feature_name=SARS.CoV.2.S1+S2	feature_id[18].value > threshold=48317.708984375
node_224: feature_name=a-MoIgG_0.10	feature_id[38].value > threshold=2066.8333129882812
node_226: feature_name=HuIgM_0.30	feature_id[13].value > threshold=750.1666870117188
node_238: feature_name=HuIgM_0.30	feature_id[13].value > threshold=760.3333435058594
node_240: feature_name=hCoV.OC43.HE	feature_id[10].value <= threshold=3879.75
node_241: feature_name=a-HuIgM_0.10	feature_id[33].value > threshold=899.8333435058594
node_243: feature_name=HuIgA_0.10	feature_id[42].value > threshold=138.1666717529297
Class: healthcare workers between 60 and 180 days after vaccination	
Rules_37	passed counts:7
node_0: feature_name=HuIgG_0.03	feature_id[44].value > threshold=3298.333251953125
node_78: feature_name=a-HuIgG_0.03	feature_id[1].value <= threshold=19665.666015625
node_79: feature_name=SARS.CoV.2.S1.mFcTag	feature_id[0].value <= threshold=54010.166015625
node_80: feature_name=SARS.CoV.2.S1+S2	feature_id[18].value <= threshold=48317.708984375
node_81: feature_name=SARS.CoV.2.Spike.RBD.His.Bac	feature_id[9].value > threshold=16347.4580078125

node_111: feature_name=hCoV.NL63.S1	feature_id[5].value > threshold=4866.533447265625
node_123: feature_name=MERS.CoV.S1.RBD.367.606.rFcTag	feature_id[3].value <= threshold=2789.3416748046875
node_124: feature_name=SARS.CoV.2.S1+S2	feature_id[18].value <= threshold=41798.25
node_125: feature_name=hCoV.OC43.S1_S2ECD.HisTag	feature_id[24].value <= threshold=49554.1328125
node_126: feature_name=SARS.CoV.2.Spike.RBD.His.Bac	feature_id[9].value <= threshold=23295.4169921875
node_127: feature_name=hCoV.OC43.S1_S2ECD.HisTag	feature_id[24].value > threshold=28407.0830078125
Class: healthcare workers within 60 days after vaccination	
Rules_38	passed counts:7
node_0: feature_name=HuIgG_0.03	feature_id[44].value <= threshold=3298.333251953125
node_1: feature_name=SARS.CoV.2.S1.mFcTag	feature_id[0].value > threshold=5354.388916015625
node_9: feature_name=HuIgM_0.03	feature_id[45].value > threshold=-80.66666793823242
node_27: feature_name=HuIgM_0.03	feature_id[45].value <= threshold=61.33333206176758
node_28: feature_name=a-HuIgG_0.30	feature_id[30].value <= threshold=34878.66796875
node_29: feature_name=HuIgG_0.03	feature_id[44].value <= threshold=769.8333435058594
node_30: feature_name=SARS.CoV.2.Spike.RBD.His.Bac	feature_id[9].value <= threshold=12895.0166015625
Class: healthcare workers over 180 days after vaccination	
Rules_39	passed counts:6
node_0: feature_name=HuIgG_0.03	feature_id[44].value <= threshold=3298.333251953125
node_1: feature_name=SARS.CoV.2.S1.mFcTag	feature_id[0].value > threshold=5354.388916015625
node_9: feature_name=HuIgM_0.03	feature_id[45].value > threshold=-80.66666793823242
node_27: feature_name=HuIgM_0.03	feature_id[45].value <= threshold=61.33333206176758
node_28: feature_name=a-HuIgG_0.30	feature_id[30].value <= threshold=34878.66796875
node_29: feature_name=HuIgG_0.03	feature_id[44].value <= threshold=769.8333435058594

node_30: feature_name=SARS.CoV.2.Spike.RBD.His.Bac	feature_id[9].value > threshold=12895.0166015625
node_32: feature_name=a-HuIgM_0.03	feature_id[34].value > threshold=-72.50000190734863
Class: healthcare workers between 60 and 180 days after vaccination	
Rules_40	passed counts:5
node_0: feature_name=HuIgG_0.03	feature_id[44].value > threshold=3298.333251953125
node_78: feature_name=a-HuIgG_0.03	feature_id[1].value > threshold=19665.666015625
node_360: feature_name=HuIgM_0.03	feature_id[45].value > threshold=-189.0
node_366: feature_name=HuIgM_0.30	feature_id[13].value > threshold=441.8333282470703
node_368: feature_name=a-HuIgG_0.10	feature_id[31].value <= threshold=32914.5
node_369: feature_name=MERS.CoV.S1.RBD.367.606.rFcTag	feature_id[3].value <= threshold=6711.89990234375
Class: healthcare workers between 60 and 180 days after vaccination	
Rules_41	passed counts:5
node_0: feature_name=HuIgG_0.03	feature_id[44].value > threshold=3298.333251953125
node_78: feature_name=a-HuIgG_0.03	feature_id[1].value <= threshold=19665.666015625
node_79: feature_name=SARS.CoV.2.S1.mFcTag	feature_id[0].value <= threshold=54010.166015625
node_80: feature_name=SARS.CoV.2.S1+S2	feature_id[18].value > threshold=48317.708984375
node_224: feature_name=a-MoIgG_0.10	feature_id[38].value > threshold=2066.8333129882812
node_226: feature_name=HuIgM_0.30	feature_id[13].value > threshold=750.1666870117188
node_238: feature_name=HuIgM_0.30	feature_id[13].value > threshold=760.3333435058594
node_240: feature_name=hCoV.OC43.HE	feature_id[10].value > threshold=3879.75
node_246: feature_name=a-HuIgA_0.30	feature_id[27].value > threshold=1379.8333740234375
node_248: feature_name=Flu.H1N1.HA1	feature_id[26].value <= threshold=53891.083984375
node_249: feature_name=MERS.CoV.S1.RBD.367.606.rFcTag	feature_id[3].value > threshold=1486.7666625976562

node_253: feature_name=HuIgM_0.03	feature_id[45].value <= threshold=-56.33333396911621
node_254: feature_name=MoIgA_0.30	feature_id[46].value > threshold=900.1666564941406
node_258: feature_name=SARS.CoV.2.S1.mFcTag	feature_id[0].value > threshold=53346.333984375
node_272: feature_name=a-MoIgA_0.03	feature_id[37].value <= threshold=2.3333334028720856
Class: healthcare workers within 60 days after vaccination	
Rules_42	passed counts:5
node_0: feature_name=HuIgG_0.03	feature_id[44].value > threshold=3298.333251953125
node_78: feature_name=a-HuIgG_0.03	feature_id[1].value <= threshold=19665.666015625
node_79: feature_name=SARS.CoV.2.S1.mFcTag	feature_id[0].value <= threshold=54010.166015625
node_80: feature_name=SARS.CoV.2.S1+S2	feature_id[18].value > threshold=48317.708984375
node_224: feature_name=a-MoIgG_0.10	feature_id[38].value > threshold=2066.8333129882812
node_226: feature_name=HuIgM_0.30	feature_id[13].value > threshold=750.1666870117188
node_238: feature_name=HuIgM_0.30	feature_id[13].value > threshold=760.3333435058594
node_240: feature_name=hCoV.OC43.HE	feature_id[10].value > threshold=3879.75
node_246: feature_name=a-HuIgA_0.30	feature_id[27].value > threshold=1379.8333740234375
node_248: feature_name=Flu.H1N1.HA1	feature_id[26].value <= threshold=53891.083984375
node_249: feature_name=MERS.CoV.S1.RBD.367.606.rFcTag	feature_id[3].value > threshold=1486.7666625976562
node_253: feature_name=HuIgM_0.03	feature_id[45].value <= threshold=-56.33333396911621
node_254: feature_name=MoIgA_0.30	feature_id[46].value <= threshold=900.1666564941406
node_255: feature_name=SARS.CoV.2.S1.RBD.mFc	feature_id[17].value <= threshold=51784.3828125
Class: healthcare workers between 60 and 180 days after vaccination	
Rules_43	passed counts:5
node_0: feature_name=HuIgG_0.03	feature_id[44].value > threshold=3298.333251953125

node_78: feature_name=a-HuIgG_0.03	feature_id[1].value <= threshold=19665.666015625
node_79: feature_name=SARS.CoV.2.S1.mFcTag	feature_id[0].value <= threshold=54010.166015625
node_80: feature_name=SARS.CoV.2.S1+S2	feature_id[18].value <= threshold=48317.708984375
node_81: feature_name=SARS.CoV.2.Spike.RBD.His.Bac	feature_id[9].value > threshold=16347.4580078125
node_111: feature_name=hCoV.NL63.S1	feature_id[5].value > threshold=4866.533447265625
node_123: feature_name=MERS.CoV.S1.RBD.367.606.rFcTag	feature_id[3].value > threshold=2789.3416748046875
node_161: feature_name=SARS.CoV.S1.HisTag	feature_id[14].value > threshold=4926.39990234375
node_179: feature_name=hCoV.HKU1.S1_S2	feature_id[22].value <= threshold=48084.783203125
node_180: feature_name=SARS.CoV.2.Spike.RBD.His.Bac	feature_id[9].value > threshold=31607.125
node_200: feature_name=SARS.CoV.2.S1.HisTag	feature_id[16].value > threshold=43902.201171875
node_214: feature_name=SARS.CoV.2.S1.mFcTag	feature_id[0].value > threshold=51362.833984375
Class: healthcare workers within 60 days after vaccination	
Rules_44	passed counts:5
node_0: feature_name=HuIgG_0.03	feature_id[44].value > threshold=3298.333251953125
node_78: feature_name=a-HuIgG_0.03	feature_id[1].value <= threshold=19665.666015625
node_79: feature_name=SARS.CoV.2.S1.mFcTag	feature_id[0].value <= threshold=54010.166015625
node_80: feature_name=SARS.CoV.2.S1+S2	feature_id[18].value <= threshold=48317.708984375
node_81: feature_name=SARS.CoV.2.Spike.RBD.His.Bac	feature_id[9].value > threshold=16347.4580078125
node_111: feature_name=hCoV.NL63.S1	feature_id[5].value > threshold=4866.533447265625
node_123: feature_name=MERS.CoV.S1.RBD.367.606.rFcTag	feature_id[3].value > threshold=2789.3416748046875
node_161: feature_name=SARS.CoV.S1.HisTag	feature_id[14].value > threshold=4926.39990234375
node_179: feature_name=hCoV.HKU1.S1_S2	feature_id[22].value <= threshold=48084.783203125
node_180: feature_name=SARS.CoV.2.Spike.RBD.His.Bac	feature_id[9].value <= threshold=31607.125
node_181: feature_name=SARS.CoV.2.S1	feature_id[15].value > threshold=33718.333984375

node_191: feature_name=a-HuIgA_0.30	feature_id[27].value > threshold=1819.5
node_193: feature_name=HuIgM_0.30	feature_id[13].value > threshold=1081.8333740234375
node_197: feature_name=hCoV.HKU1.S1_S2	feature_id[22].value <= threshold=44172.416015625
Class: healthcare workers between 60 and 180 days after vaccination	
Rules_45	passed counts:5
node_0: feature_name=HuIgG_0.03	feature_id[44].value > threshold=3298.333251953125
node_78: feature_name=a-HuIgG_0.03	feature_id[1].value <= threshold=19665.666015625
node_79: feature_name=SARS.CoV.2.S1.mFcTag	feature_id[0].value <= threshold=54010.166015625
node_80: feature_name=SARS.CoV.2.S1+S2	feature_id[18].value <= threshold=48317.708984375
node_81: feature_name=SARS.CoV.2.Spike.RBD.His.Bac	feature_id[9].value > threshold=16347.4580078125
node_111: feature_name=hCoV.NL63.S1	feature_id[5].value <= threshold=4866.533447265625
node_112: feature_name=SARS.CoV.2.S1.mFcTag	feature_id[0].value <= threshold=40690.80078125
node_113: feature_name=a-HuIgM_0.30	feature_id[32].value > threshold=1087.5
Class: healthcare workers between 60 and 180 days after vaccination	
Rules_46	passed counts:5
node_0: feature_name=HuIgG_0.03	feature_id[44].value <= threshold=3298.333251953125
node_1: feature_name=SARS.CoV.2.S1.mFcTag	feature_id[0].value > threshold=5354.388916015625
node_9: feature_name=HuIgM_0.03	feature_id[45].value > threshold=-80.66666793823242
node_27: feature_name=HuIgM_0.03	feature_id[45].value <= threshold=61.33333206176758
node_28: feature_name=a-HuIgG_0.30	feature_id[30].value > threshold=34878.66796875
node_64: feature_name=a-HuIgG_0.03	feature_id[1].value > threshold=9340.3330078125
node_68: feature_name=a-HuIgA_0.30	feature_id[27].value <= threshold=1918.1666870117188
node_69: feature_name=SARS.CoV.2.Spike.RBD.rFc	feature_id[7].value > threshold=20694.49951171875

Class: healthcare workers between 60 and 180 days after vaccination	
Rules_47	passed counts:4
node_0: feature_name=HuIgG_0.03	feature_id[44].value > threshold=3298.333251953125
node_78: feature_name=a-HuIgG_0.03	feature_id[1].value > threshold=19665.666015625
node_360: feature_name=HuIgM_0.03	feature_id[45].value > threshold=-189.0
node_366: feature_name=HuIgM_0.30	feature_id[13].value <= threshold=441.8333282470703
Class: healthcare workers between 60 and 180 days after vaccination	
Rules_48	passed counts:4
node_0: feature_name=HuIgG_0.03	feature_id[44].value > threshold=3298.333251953125
node_78: feature_name=a-HuIgG_0.03	feature_id[1].value <= threshold=19665.666015625
node_79: feature_name=SARS.CoV.2.S1.mFcTag	feature_id[0].value > threshold=54010.166015625
node_297: feature_name=a-MoIgG_0.10	feature_id[38].value > threshold=2382.8333740234375
node_303: feature_name=SARS.CoV.2.S1	feature_id[15].value <= threshold=51713.66796875
node_304: feature_name=hCoV.HKU1.S1_AA1.760	feature_id[8].value > threshold=16192.1669921875
node_310: feature_name=a-HuIgM_0.10	feature_id[33].value > threshold=176.5
node_314: feature_name=SARS.CoV.2.S1+S2	feature_id[18].value > threshold=50299.93359375
node_318: feature_name=a-HuIgG_0.30	feature_id[30].value <= threshold=47587.833984375
node_319: feature_name=MERS.CoV.S1.RBD.367.606.rFcTag	feature_id[3].value > threshold=3945.61669921875
node_323: feature_name=a-MoIgG_0.10	feature_id[38].value > threshold=6842.166748046875
node_327: feature_name=SARS.CoV.2.S1.mFcTag	feature_id[0].value <= threshold=54403.482421875
node_328: feature_name=HuIgG_0.03	feature_id[44].value > threshold=5438.833251953125
Class: healthcare workers between 60 and 180 days after vaccination	

Rules_49	passed counts:4
node_0: feature_name=HuIgG_0.03	feature_id[44].value > threshold=3298.333251953125
node_78: feature_name=a-HuIgG_0.03	feature_id[1].value <= threshold=19665.666015625
node_79: feature_name=SARS.CoV.2.S1.mFcTag	feature_id[0].value > threshold=54010.166015625
node_297: feature_name=a-MoIgG_0.10	feature_id[38].value > threshold=2382.8333740234375
node_303: feature_name=SARS.CoV.2.S1	feature_id[15].value <= threshold=51713.66796875
node_304: feature_name=hCoV.HKU1.S1_AA1.760	feature_id[8].value <= threshold=16192.1669921875
node_305: feature_name=a-HuIgM_0.03	feature_id[34].value <= threshold=20.666666984558105
node_306: feature_name=a-HuIgM_0.03	feature_id[34].value > threshold=-3.000000238418579
Class: healthcare workers between 60 and 180 days after vaccination	
Rules_50	passed counts:4
node_0: feature_name=HuIgG_0.03	feature_id[44].value > threshold=3298.333251953125
node_78: feature_name=a-HuIgG_0.03	feature_id[1].value <= threshold=19665.666015625
node_79: feature_name=SARS.CoV.2.S1.mFcTag	feature_id[0].value <= threshold=54010.166015625
node_80: feature_name=SARS.CoV.2.S1+S2	feature_id[18].value > threshold=48317.708984375
node_224: feature_name=a-MoIgG_0.10	feature_id[38].value > threshold=2066.8333129882812
node_226: feature_name=HuIgM_0.30	feature_id[13].value > threshold=750.1666870117188
node_238: feature_name=HuIgM_0.30	feature_id[13].value > threshold=760.3333435058594
node_240: feature_name=hCoV.OC43.HE	feature_id[10].value > threshold=3879.75
node_246: feature_name=a-HuIgA_0.30	feature_id[27].value > threshold=1379.8333740234375
node_248: feature_name=Flu.H1N1.HA1	feature_id[26].value <= threshold=53891.083984375
node_249: feature_name=MERS.CoV.S1.RBD.367.606.rFcTag	feature_id[3].value > threshold=1486.7666625976562
node_253: feature_name=HuIgM_0.03	feature_id[45].value <= threshold=-56.33333396911621
node_254: feature_name=MoIgA_0.30	feature_id[46].value > threshold=900.1666564941406

node_258: feature_name=SARS.CoV.2.S1.mFcTag	feature_id[0].value <= threshold=53346.333984375
node_259: feature_name=SARS.CoV.2.S1	feature_id[15].value <= threshold=47301.333984375
node_260: feature_name=a-MoIgM_0.03	feature_id[41].value > threshold=127.16666793823242
node_262: feature_name=hCoV.OC43.HE	feature_id[10].value <= threshold=12272.5166015625
node_263: feature_name=SARS.CoV.2.S1+S2	feature_id[18].value > threshold=49907.841796875
node_265: feature_name=a-MoIgG_0.10	feature_id[38].value <= threshold=23887.5
Class: healthcare workers between 60 and 180 days after vaccination	
Rules_51	passed counts:4
node_0: feature_name=HuIgG_0.03	feature_id[44].value > threshold=3298.333251953125
node_78: feature_name=a-HuIgG_0.03	feature_id[1].value <= threshold=19665.666015625
node_79: feature_name=SARS.CoV.2.S1.mFcTag	feature_id[0].value <= threshold=54010.166015625
node_80: feature_name=SARS.CoV.2.S1+S2	feature_id[18].value > threshold=48317.708984375
node_224: feature_name=a-MoIgG_0.10	feature_id[38].value > threshold=2066.8333129882812
node_226: feature_name=HuIgM_0.30	feature_id[13].value > threshold=750.1666870117188
node_238: feature_name=HuIgM_0.30	feature_id[13].value > threshold=760.3333435058594
node_240: feature_name=hCoV.OC43.HE	feature_id[10].value > threshold=3879.75
node_246: feature_name=a-HuIgA_0.30	feature_id[27].value > threshold=1379.8333740234375
node_248: feature_name=Flu.H1N1.HA1	feature_id[26].value <= threshold=53891.083984375
node_249: feature_name=MERS.CoV.S1.RBD.367.606.rFcTag	feature_id[3].value > threshold=1486.7666625976562
node_253: feature_name=HuIgM_0.03	feature_id[45].value <= threshold=-56.33333396911621
node_254: feature_name=MoIgA_0.30	feature_id[46].value > threshold=900.1666564941406
node_258: feature_name=SARS.CoV.2.S1.mFcTag	feature_id[0].value <= threshold=53346.333984375
node_259: feature_name=SARS.CoV.2.S1	feature_id[15].value <= threshold=47301.333984375
node_260: feature_name=a-MoIgM_0.03	feature_id[41].value > threshold=127.16666793823242

node_262: feature_name=hCoV.OC43.HE	feature_id[10].value <= threshold=12272.5166015625
node_263: feature_name=SARS.CoV.2.S1+S2	feature_id[18].value <= threshold=49907.841796875
Class: healthcare workers within 60 days after vaccination	
Rules_52	passed counts:4
node_0: feature_name=HuIgG_0.03	feature_id[44].value > threshold=3298.333251953125
node_78: feature_name=a-HuIgG_0.03	feature_id[1].value <= threshold=19665.666015625
node_79: feature_name=SARS.CoV.2.S1.mFcTag	feature_id[0].value <= threshold=54010.166015625
node_80: feature_name=SARS.CoV.2.S1+S2	feature_id[18].value > threshold=48317.708984375
node_224: feature_name=a-MoIgG_0.10	feature_id[38].value > threshold=2066.8333129882812
node_226: feature_name=HuIgM_0.30	feature_id[13].value > threshold=750.1666870117188
node_238: feature_name=HuIgM_0.30	feature_id[13].value > threshold=760.3333435058594
node_240: feature_name=hCoV.OC43.HE	feature_id[10].value <= threshold=3879.75
node_241: feature_name=a-HuIgM_0.10	feature_id[33].value <= threshold=899.8333435058594
Class: healthcare workers within 60 days after vaccination	
Rules_53	passed counts:4
node_0: feature_name=HuIgG_0.03	feature_id[44].value > threshold=3298.333251953125
node_78: feature_name=a-HuIgG_0.03	feature_id[1].value <= threshold=19665.666015625
node_79: feature_name=SARS.CoV.2.S1.mFcTag	feature_id[0].value <= threshold=54010.166015625
node_80: feature_name=SARS.CoV.2.S1+S2	feature_id[18].value <= threshold=48317.708984375
node_81: feature_name=SARS.CoV.2.Spike.RBD.His.Bac	feature_id[9].value > threshold=16347.4580078125
node_111: feature_name=hCoV.NL63.S1	feature_id[5].value > threshold=4866.533447265625
node_123: feature_name=MERS.CoV.S1.RBD.367.606.rFcTag	feature_id[3].value > threshold=2789.3416748046875
node_161: feature_name=SARS.CoV.S1.HisTag	feature_id[14].value > threshold=4926.39990234375

node_179: feature_name=hCoV.HKU1.S1_S2	feature_id[22].value > threshold=48084.783203125
node_217: feature_name=MoIgA_0.30	feature_id[46].value <= threshold=24299.6669921875
node_218: feature_name=DcCoV.HKU23.NP	feature_id[21].value <= threshold=20749.25
node_219: feature_name=DcCoV.HKU23.NP	feature_id[21].value > threshold=13731.44140625
Class: healthcare workers between 60 and 180 days after vaccination	
Rules_54	passed counts:4
node_0: feature_name=HuIgG_0.03	feature_id[44].value > threshold=3298.333251953125
node_78: feature_name=a-HuIgG_0.03	feature_id[1].value <= threshold=19665.666015625
node_79: feature_name=SARS.CoV.2.S1.mFcTag	feature_id[0].value <= threshold=54010.166015625
node_80: feature_name=SARS.CoV.2.S1+S2	feature_id[18].value <= threshold=48317.708984375
node_81: feature_name=SARS.CoV.2.Spike.RBD.His.Bac	feature_id[9].value > threshold=16347.4580078125
node_111: feature_name=hCoV.NL63.S1	feature_id[5].value > threshold=4866.533447265625
node_123: feature_name=MERS.CoV.S1.RBD.367.606.rFcTag	feature_id[3].value > threshold=2789.3416748046875
node_161: feature_name=SARS.CoV.S1.HisTag	feature_id[14].value > threshold=4926.39990234375
node_179: feature_name=hCoV.HKU1.S1_S2	feature_id[22].value <= threshold=48084.783203125
node_180: feature_name=SARS.CoV.2.Spike.RBD.His.Bac	feature_id[9].value <= threshold=31607.125
node_181: feature_name=SARS.CoV.2.S1	feature_id[15].value > threshold=33718.333984375
node_191: feature_name=a-HuIgA_0.30	feature_id[27].value <= threshold=1819.5
Class: healthcare workers between 60 and 180 days after vaccination	
Rules_55	passed counts:4
node_0: feature_name=HuIgG_0.03	feature_id[44].value > threshold=3298.333251953125
node_78: feature_name=a-HuIgG_0.03	feature_id[1].value <= threshold=19665.666015625
node_79: feature_name=SARS.CoV.2.S1.mFcTag	feature_id[0].value <= threshold=54010.166015625

node_80: feature_name=SARS.CoV.2.S1+S2	feature_id[18].value <= threshold=48317.708984375
node_81: feature_name=SARS.CoV.2.Spike.RBD.His.Bac	feature_id[9].value > threshold=16347.4580078125
node_111: feature_name=hCoV.NL63.S1	feature_id[5].value > threshold=4866.533447265625
node_123: feature_name=MERS.CoV.S1.RBD.367.606.rFcTag	feature_id[3].value > threshold=2789.3416748046875
node_161: feature_name=SARS.CoV.S1.HisTag	feature_id[14].value <= threshold=4926.39990234375
node_162: feature_name=a-MoIgA_0.10	feature_id[36].value <= threshold=3108.3333740234375
node_163: feature_name=Flu.B_Mal/.HA1+HA2	feature_id[25].value > threshold=38684.7265625
node_167: feature_name=SARS.CoV.NP	feature_id[19].value <= threshold=18293.6826171875
node_168: feature_name=a-HuIgG_0.03	feature_id[1].value <= threshold=10603.0
node_169: feature_name=a-HuIgM_0.10	feature_id[33].value <= threshold=483.1666717529297
Class: healthcare workers between 60 and 180 days after vaccination	
Rules_56	passed counts:4
node_0: feature_name=HuIgG_0.03	feature_id[44].value > threshold=3298.333251953125
node_78: feature_name=a-HuIgG_0.03	feature_id[1].value <= threshold=19665.666015625
node_79: feature_name=SARS.CoV.2.S1.mFcTag	feature_id[0].value <= threshold=54010.166015625
node_80: feature_name=SARS.CoV.2.S1+S2	feature_id[18].value <= threshold=48317.708984375
node_81: feature_name=SARS.CoV.2.Spike.RBD.His.Bac	feature_id[9].value > threshold=16347.4580078125
node_111: feature_name=hCoV.NL63.S1	feature_id[5].value > threshold=4866.533447265625
node_123: feature_name=MERS.CoV.S1.RBD.367.606.rFcTag	feature_id[3].value <= threshold=2789.3416748046875
node_124: feature_name=SARS.CoV.2.S1+S2	feature_id[18].value > threshold=41798.25
node_140: feature_name=SARS.CoV.2.S1.RBD.mFc	feature_id[17].value <= threshold=44491.166015625
node_141: feature_name=a-HuIgM_0.03	feature_id[34].value > threshold=-6.833333492279053
node_149: feature_name=a-MoIgA_0.30	feature_id[35].value > threshold=935.3333435058594
node_151: feature_name=HuIgM_0.30	feature_id[13].value > threshold=1110.5

node_157: feature_name=a-MoIgA_0.30	feature_id[35].value > threshold=2098.6666259765625
Class: healthcare workers within 60 days after vaccination	
Rules_57	passed counts:4
node_0: feature_name=HuIgG_0.03	feature_id[44].value > threshold=3298.333251953125
node_78: feature_name=a-HuIgG_0.03	feature_id[1].value <= threshold=19665.666015625
node_79: feature_name=SARS.CoV.2.S1.mFcTag	feature_id[0].value <= threshold=54010.166015625
node_80: feature_name=SARS.CoV.2.S1+S2	feature_id[18].value <= threshold=48317.708984375
node_81: feature_name=SARS.CoV.2.Spike.RBD.His.Bac	feature_id[9].value <= threshold=16347.4580078125
node_82: feature_name=SARS.CoV.2.S1	feature_id[15].value <= threshold=2844.6334228515625
node_83: feature_name=SARS.CoV.2.S1+S2	feature_id[18].value <= threshold=3298.25
node_84: feature_name=hCoV.OC43.HE	feature_id[10].value > threshold=-45.900001525878906
Class: healthcare workers within 60 days after vaccination	
Rules_58	passed counts:3
node_0: feature_name=HuIgG_0.03	feature_id[44].value > threshold=3298.333251953125
node_78: feature_name=a-HuIgG_0.03	feature_id[1].value <= threshold=19665.666015625
node_79: feature_name=SARS.CoV.2.S1.mFcTag	feature_id[0].value > threshold=54010.166015625
node_297: feature_name=a-MoIgG_0.10	feature_id[38].value > threshold=2382.8333740234375
node_303: feature_name=SARS.CoV.2.S1	feature_id[15].value > threshold=51713.66796875
node_345: feature_name=SARS.CoV.2.S1.HisTag	feature_id[16].value > threshold=51766.75
node_347: feature_name=HuIgA_0.03	feature_id[43].value > threshold=-60.33333396911621
node_353: feature_name=SARS.CoV.2.Spike.RBD.His.Bac	feature_id[9].value > threshold=51551.166015625
node_357: feature_name=SARS.CoV.S1.HisTag	feature_id[14].value > threshold=37552.33203125
Class: healthcare workers within 60 days after vaccination	

Rules_59	passed counts:3
node_0: feature_name=HuIgG_0.03	feature_id[44].value > threshold=3298.333251953125
node_78: feature_name=a-HuIgG_0.03	feature_id[1].value <= threshold=19665.666015625
node_79: feature_name=SARS.CoV.2.S1.mFcTag	feature_id[0].value > threshold=54010.166015625
node_297: feature_name=a-MoIgG_0.10	feature_id[38].value > threshold=2382.8333740234375
node_303: feature_name=SARS.CoV.2.S1	feature_id[15].value > threshold=51713.66796875
node_345: feature_name=SARS.CoV.2.S1.HisTag	feature_id[16].value > threshold=51766.75
node_347: feature_name=HuIgA_0.03	feature_id[43].value > threshold=-60.33333396911621
node_353: feature_name=SARS.CoV.2.Spike.RBD.His.Bac	feature_id[9].value > threshold=51551.166015625
node_357: feature_name=SARS.CoV.S1.HisTag	feature_id[14].value <= threshold=37552.33203125
Class: healthcare workers between 60 and 180 days after vaccination	
Rules_60	passed counts:3
node_0: feature_name=HuIgG_0.03	feature_id[44].value > threshold=3298.333251953125
node_78: feature_name=a-HuIgG_0.03	feature_id[1].value <= threshold=19665.666015625
node_79: feature_name=SARS.CoV.2.S1.mFcTag	feature_id[0].value > threshold=54010.166015625
node_297: feature_name=a-MoIgG_0.10	feature_id[38].value > threshold=2382.8333740234375
node_303: feature_name=SARS.CoV.2.S1	feature_id[15].value > threshold=51713.66796875
node_345: feature_name=SARS.CoV.2.S1.HisTag	feature_id[16].value > threshold=51766.75
node_347: feature_name=HuIgA_0.03	feature_id[43].value <= threshold=-60.33333396911621
node_348: feature_name=hCoV.HKU1.S1_S2	feature_id[22].value <= threshold=51287.134765625
Class: healthcare workers between 60 and 180 days after vaccination	
Rules_61	passed counts:3

node_0: feature_name=HuIgG_0.03	feature_id[44].value > threshold=3298.333251953125
node_78: feature_name=a-HuIgG_0.03	feature_id[1].value <= threshold=19665.666015625
node_79: feature_name=SARS.CoV.2.S1.mFcTag	feature_id[0].value > threshold=54010.166015625
node_297: feature_name=a-MoIgG_0.10	feature_id[38].value > threshold=2382.8333740234375
node_303: feature_name=SARS.CoV.2.S1	feature_id[15].value > threshold=51713.66796875
node_345: feature_name=SARS.CoV.2.S1.HisTag	feature_id[16].value <= threshold=51766.75
Class: healthcare workers between 60 and 180 days after vaccination	
Rules_62	passed counts:3
node_0: feature_name=HuIgG_0.03	feature_id[44].value > threshold=3298.333251953125
node_78: feature_name=a-HuIgG_0.03	feature_id[1].value <= threshold=19665.666015625
node_79: feature_name=SARS.CoV.2.S1.mFcTag	feature_id[0].value > threshold=54010.166015625
node_297: feature_name=a-MoIgG_0.10	feature_id[38].value > threshold=2382.8333740234375
node_303: feature_name=SARS.CoV.2.S1	feature_id[15].value <= threshold=51713.66796875
node_304: feature_name=hCoV.HKU1.S1_AA1.760	feature_id[8].value > threshold=16192.1669921875
node_310: feature_name=a-HuIgM_0.10	feature_id[33].value > threshold=176.5
node_314: feature_name=SARS.CoV.2.S1+S2	feature_id[18].value > threshold=50299.93359375
node_318: feature_name=a-HuIgG_0.30	feature_id[30].value > threshold=47587.833984375
node_336: feature_name=hCoV.HKU1.S1_AA1.760	feature_id[8].value > threshold=45964.3828125
node_342: feature_name=a-HuIgG_0.03	feature_id[1].value > threshold=13373.1669921875
Class: healthcare workers within 60 days after vaccination	
Rules_63	passed counts:3
node_0: feature_name=HuIgG_0.03	feature_id[44].value > threshold=3298.333251953125
node_78: feature_name=a-HuIgG_0.03	feature_id[1].value <= threshold=19665.666015625

node_79: feature_name=SARS.CoV.2.S1.mFcTag	feature_id[0].value > threshold=54010.166015625
node_297: feature_name=a-MoIgG_0.10	feature_id[38].value > threshold=2382.8333740234375
node_303: feature_name=SARS.CoV.2.S1	feature_id[15].value <= threshold=51713.66796875
node_304: feature_name=hCoV.HKU1.S1_AA1.760	feature_id[8].value > threshold=16192.1669921875
node_310: feature_name=a-HuIgM_0.10	feature_id[33].value > threshold=176.5
node_314: feature_name=SARS.CoV.2.S1+S2	feature_id[18].value > threshold=50299.93359375
node_318: feature_name=a-HuIgG_0.30	feature_id[30].value <= threshold=47587.833984375
node_319: feature_name=MERS.CoV.S1.RBD.367.606.rFcTag	feature_id[3].value <= threshold=3945.61669921875
node_320: feature_name=Flu.H1N1.HA1	feature_id[26].value > threshold=52361.833984375
Class: healthcare workers between 60 and 180 days after vaccination	
Rules_64	passed counts:3
node_0: feature_name=HuIgG_0.03	feature_id[44].value > threshold=3298.333251953125
node_78: feature_name=a-HuIgG_0.03	feature_id[1].value <= threshold=19665.666015625
node_79: feature_name=SARS.CoV.2.S1.mFcTag	feature_id[0].value > threshold=54010.166015625
node_297: feature_name=a-MoIgG_0.10	feature_id[38].value > threshold=2382.8333740234375
node_303: feature_name=SARS.CoV.2.S1	feature_id[15].value <= threshold=51713.66796875
node_304: feature_name=hCoV.HKU1.S1_AA1.760	feature_id[8].value > threshold=16192.1669921875
node_310: feature_name=a-HuIgM_0.10	feature_id[33].value <= threshold=176.5
node_311: feature_name=a-HuIgM_0.03	feature_id[34].value <= threshold=-86.66666793823242
Class: healthcare workers between 60 and 180 days after vaccination	
Rules_65	passed counts:3
node_0: feature_name=HuIgG_0.03	feature_id[44].value > threshold=3298.333251953125
node_78: feature_name=a-HuIgG_0.03	feature_id[1].value <= threshold=19665.666015625

node_79: feature_name=SARS.CoV.2.S1.mFcTag	feature_id[0].value > threshold=54010.166015625
node_297: feature_name=a-MoIgG_0.10	feature_id[38].value > threshold=2382.8333740234375
node_303: feature_name=SARS.CoV.2.S1	feature_id[15].value <= threshold=51713.66796875
node_304: feature_name=hCoV.HKU1.S1_AA1.760	feature_id[8].value <= threshold=16192.1669921875
node_305: feature_name=a-HuIgM_0.03	feature_id[34].value <= threshold=20.666666984558105
node_306: feature_name=a-HuIgM_0.03	feature_id[34].value <= threshold=-3.000000238418579
Class: healthcare workers within 60 days after vaccination	
Rules_66	passed counts:3
node_0: feature_name=HuIgG_0.03	feature_id[44].value > threshold=3298.333251953125
node_78: feature_name=a-HuIgG_0.03	feature_id[1].value <= threshold=19665.666015625
node_79: feature_name=SARS.CoV.2.S1.mFcTag	feature_id[0].value <= threshold=54010.166015625
node_80: feature_name=SARS.CoV.2.S1+S2	feature_id[18].value > threshold=48317.708984375
node_224: feature_name=a-MoIgG_0.10	feature_id[38].value > threshold=2066.8333129882812
node_226: feature_name=HuIgM_0.30	feature_id[13].value > threshold=750.1666870117188
node_238: feature_name=HuIgM_0.30	feature_id[13].value > threshold=760.3333435058594
node_240: feature_name=hCoV.OC43.HE	feature_id[10].value > threshold=3879.75
node_246: feature_name=a-HuIgA_0.30	feature_id[27].value > threshold=1379.8333740234375
node_248: feature_name=Flu.H1N1.HA1	feature_id[26].value <= threshold=53891.083984375
node_249: feature_name=MERS.CoV.S1.RBD.367.606.rFcTag	feature_id[3].value > threshold=1486.7666625976562
node_253: feature_name=HuIgM_0.03	feature_id[45].value > threshold=-56.33333396911621
node_279: feature_name=hCoV.229E.S1	feature_id[2].value <= threshold=51743.583984375
node_280: feature_name=Flu.B_Mal/.HA1	feature_id[4].value <= threshold=48206.833984375
node_281: feature_name=a-MoIgM_0.10	feature_id[40].value > threshold=989.5000305175781
node_285: feature_name=HuIgM_0.30	feature_id[13].value > threshold=2008.3333129882812

node_287: feature_name=SARS.CoV.2.S1.RBD.mFc	feature_id[17].value > threshold=43325.44921875
Class: healthcare workers within 60 days after vaccination	
Rules_67	passed counts:3
node_0: feature_name=HuIgG_0.03	feature_id[44].value > threshold=3298.333251953125
node_78: feature_name=a-HuIgG_0.03	feature_id[1].value <= threshold=19665.666015625
node_79: feature_name=SARS.CoV.2.S1.mFcTag	feature_id[0].value <= threshold=54010.166015625
node_80: feature_name=SARS.CoV.2.S1+S2	feature_id[18].value > threshold=48317.708984375
node_224: feature_name=a-MoIgG_0.10	feature_id[38].value > threshold=2066.8333129882812
node_226: feature_name=HuIgM_0.30	feature_id[13].value > threshold=750.1666870117188
node_238: feature_name=HuIgM_0.30	feature_id[13].value > threshold=760.3333435058594
node_240: feature_name=hCoV.OC43.HE	feature_id[10].value > threshold=3879.75
node_246: feature_name=a-HuIgA_0.30	feature_id[27].value > threshold=1379.8333740234375
node_248: feature_name=Flu.H1N1.HA1	feature_id[26].value <= threshold=53891.083984375
node_249: feature_name=MERS.CoV.S1.RBD.367.606.rFcTag	feature_id[3].value > threshold=1486.7666625976562
node_253: feature_name=HuIgM_0.03	feature_id[45].value <= threshold=-56.33333396911621
node_254: feature_name=MoIgA_0.30	feature_id[46].value > threshold=900.1666564941406
node_258: feature_name=SARS.CoV.2.S1.mFcTag	feature_id[0].value > threshold=53346.333984375
node_272: feature_name=a-MoIgA_0.03	feature_id[37].value > threshold=2.3333334028720856
node_274: feature_name=Flu.B_Mal/.HA1+HA2	feature_id[25].value > threshold=53981.70703125
Class: healthcare workers within 60 days after vaccination	
Rules_68	passed counts:3
node_0: feature_name=HuIgG_0.03	feature_id[44].value > threshold=3298.333251953125
node_78: feature_name=a-HuIgG_0.03	feature_id[1].value <= threshold=19665.666015625

node_79: feature_name=SARS.CoV.2.S1.mFcTag	feature_id[0].value <= threshold=54010.166015625
node_80: feature_name=SARS.CoV.2.S1+S2	feature_id[18].value > threshold=48317.708984375
node_224: feature_name=a-MoIgG_0.10	feature_id[38].value > threshold=2066.8333129882812
node_226: feature_name=HuIgM_0.30	feature_id[13].value > threshold=750.1666870117188
node_238: feature_name=HuIgM_0.30	feature_id[13].value > threshold=760.3333435058594
node_240: feature_name=hCoV.OC43.HE	feature_id[10].value > threshold=3879.75
node_246: feature_name=a-HuIgA_0.30	feature_id[27].value > threshold=1379.8333740234375
node_248: feature_name=Flu.H1N1.HA1	feature_id[26].value <= threshold=53891.083984375
node_249: feature_name=MERS.CoV.S1.RBD.367.606.rFcTag	feature_id[3].value > threshold=1486.7666625976562
node_253: feature_name=HuIgM_0.03	feature_id[45].value <= threshold=-56.33333396911621
node_254: feature_name=MoIgA_0.30	feature_id[46].value > threshold=900.1666564941406
node_258: feature_name=SARS.CoV.2.S1.mFcTag	feature_id[0].value <= threshold=53346.333984375
node_259: feature_name=SARS.CoV.2.S1	feature_id[15].value <= threshold=47301.333984375
node_260: feature_name=a-MoIgM_0.03	feature_id[41].value <= threshold=127.16666793823242
Class: healthcare workers between 60 and 180 days after vaccination	
Rules_69	passed counts:3
node_0: feature_name=HuIgG_0.03	feature_id[44].value > threshold=3298.333251953125
node_78: feature_name=a-HuIgG_0.03	feature_id[1].value <= threshold=19665.666015625
node_79: feature_name=SARS.CoV.2.S1.mFcTag	feature_id[0].value <= threshold=54010.166015625
node_80: feature_name=SARS.CoV.2.S1+S2	feature_id[18].value > threshold=48317.708984375
node_224: feature_name=a-MoIgG_0.10	feature_id[38].value > threshold=2066.8333129882812
node_226: feature_name=HuIgM_0.30	feature_id[13].value > threshold=750.1666870117188
node_238: feature_name=HuIgM_0.30	feature_id[13].value > threshold=760.3333435058594
node_240: feature_name=hCoV.OC43.HE	feature_id[10].value > threshold=3879.75

node_246: feature_name=a-HuIgA_0.30	feature_id[27].value > threshold=1379.8333740234375
node_248: feature_name=Flu.H1N1.HA1	feature_id[26].value <= threshold=53891.083984375
node_249: feature_name=MERS.CoV.S1.RBD.367.606.rFcTag	feature_id[3].value <= threshold=1486.7666625976562
node_250: feature_name=a-MoIgM_0.10	feature_id[40].value > threshold=1601.8333129882812
Class: healthcare workers between 60 and 180 days after vaccination	
Rules_70	passed counts:3
node_0: feature_name=HuIgG_0.03	feature_id[44].value > threshold=3298.333251953125
node_78: feature_name=a-HuIgG_0.03	feature_id[1].value <= threshold=19665.666015625
node_79: feature_name=SARS.CoV.2.S1.mFcTag	feature_id[0].value <= threshold=54010.166015625
node_80: feature_name=SARS.CoV.2.S1+S2	feature_id[18].value > threshold=48317.708984375
node_224: feature_name=a-MoIgG_0.10	feature_id[38].value > threshold=2066.8333129882812
node_226: feature_name=HuIgM_0.30	feature_id[13].value > threshold=750.1666870117188
node_238: feature_name=HuIgM_0.30	feature_id[13].value <= threshold=760.3333435058594
Class: healthcare workers between 60 and 180 days after vaccination	
Rules_71	passed counts:3
node_0: feature_name=HuIgG_0.03	feature_id[44].value > threshold=3298.333251953125
node_78: feature_name=a-HuIgG_0.03	feature_id[1].value <= threshold=19665.666015625
node_79: feature_name=SARS.CoV.2.S1.mFcTag	feature_id[0].value <= threshold=54010.166015625
node_80: feature_name=SARS.CoV.2.S1+S2	feature_id[18].value > threshold=48317.708984375
node_224: feature_name=a-MoIgG_0.10	feature_id[38].value > threshold=2066.8333129882812
node_226: feature_name=HuIgM_0.30	feature_id[13].value <= threshold=750.1666870117188
node_227: feature_name=SARS.CoV.S1.RBD.HisTag	feature_id[20].value <= threshold=7585.25
node_228: feature_name=MERS.CoV.S1.ECD.1-1297.HisTag	feature_id[12].value > threshold=22314.0

node_230: feature_name=SARS.CoV.2.Spike.RBD.rFc	feature_id[7].value > threshold=25894.986328125
Class: healthcare workers between 60 and 180 days after vaccination	
Rules_72	passed counts:3
node_0: feature_name=HuIgG_0.03	feature_id[44].value > threshold=3298.333251953125
node_78: feature_name=a-HuIgG_0.03	feature_id[1].value <= threshold=19665.666015625
node_79: feature_name=SARS.CoV.2.S1.mFcTag	feature_id[0].value <= threshold=54010.166015625
node_80: feature_name=SARS.CoV.2.S1+S2	feature_id[18].value > threshold=48317.708984375
node_224: feature_name=a-MoIgG_0.10	feature_id[38].value > threshold=2066.8333129882812
node_226: feature_name=HuIgM_0.30	feature_id[13].value <= threshold=750.1666870117188
node_227: feature_name=SARS.CoV.S1.RBD.HisTag	feature_id[20].value <= threshold=7585.25
node_228: feature_name=MERS.CoV.S1.ECD.1-1297.HisTag	feature_id[12].value <= threshold=22314.0
Class: healthcare workers within 60 days after vaccination	
Rules_73	passed counts:3
node_0: feature_name=HuIgG_0.03	feature_id[44].value > threshold=3298.333251953125
node_78: feature_name=a-HuIgG_0.03	feature_id[1].value <= threshold=19665.666015625
node_79: feature_name=SARS.CoV.2.S1.mFcTag	feature_id[0].value <= threshold=54010.166015625
node_80: feature_name=SARS.CoV.2.S1+S2	feature_id[18].value > threshold=48317.708984375
node_224: feature_name=a-MoIgG_0.10	feature_id[38].value <= threshold=2066.8333129882812
Class: healthcare workers over 180 days after vaccination	
Rules_74	passed counts:3
node_0: feature_name=HuIgG_0.03	feature_id[44].value > threshold=3298.333251953125
node_78: feature_name=a-HuIgG_0.03	feature_id[1].value <= threshold=19665.666015625

node_79: feature_name=SARS.CoV.2.S1.mFcTag	feature_id[0].value <= threshold=54010.166015625
node_80: feature_name=SARS.CoV.2.S1+S2	feature_id[18].value <= threshold=48317.708984375
node_81: feature_name=SARS.CoV.2.Spike.RBD.His.Bac	feature_id[9].value > threshold=16347.4580078125
node_111: feature_name=hCoV.NL63.S1	feature_id[5].value > threshold=4866.533447265625
node_123: feature_name=MERS.CoV.S1.RBD.367.606.rFcTag	feature_id[3].value > threshold=2789.3416748046875
node_161: feature_name=SARS.CoV.S1.HisTag	feature_id[14].value > threshold=4926.39990234375
node_179: feature_name=hCoV.HKU1.S1_S2	feature_id[22].value > threshold=48084.783203125
node_217: feature_name=MoIgA_0.30	feature_id[46].value > threshold=24299.6669921875
Class: healthcare workers between 60 and 180 days after vaccination	
Rules_75	passed counts:3
node_0: feature_name=HuIgG_0.03	feature_id[44].value > threshold=3298.333251953125
node_78: feature_name=a-HuIgG_0.03	feature_id[1].value <= threshold=19665.666015625
node_79: feature_name=SARS.CoV.2.S1.mFcTag	feature_id[0].value <= threshold=54010.166015625
node_80: feature_name=SARS.CoV.2.S1+S2	feature_id[18].value <= threshold=48317.708984375
node_81: feature_name=SARS.CoV.2.Spike.RBD.His.Bac	feature_id[9].value > threshold=16347.4580078125
node_111: feature_name=hCoV.NL63.S1	feature_id[5].value > threshold=4866.533447265625
node_123: feature_name=MERS.CoV.S1.RBD.367.606.rFcTag	feature_id[3].value > threshold=2789.3416748046875
node_161: feature_name=SARS.CoV.S1.HisTag	feature_id[14].value > threshold=4926.39990234375
node_179: feature_name=hCoV.HKU1.S1_S2	feature_id[22].value > threshold=48084.783203125
node_217: feature_name=MoIgA_0.30	feature_id[46].value <= threshold=24299.6669921875
node_218: feature_name=DcCoV.HKU23.NP	feature_id[21].value <= threshold=20749.25
node_219: feature_name=DcCoV.HKU23.NP	feature_id[21].value <= threshold=13731.44140625
Class: healthcare workers within 60 days after vaccination	

Rules_76	passed counts:3
node_0: feature_name=HuIgG_0.03	feature_id[44].value > threshold=3298.333251953125
node_78: feature_name=a-HuIgG_0.03	feature_id[1].value <= threshold=19665.666015625
node_79: feature_name=SARS.CoV.2.S1.mFcTag	feature_id[0].value <= threshold=54010.166015625
node_80: feature_name=SARS.CoV.2.S1+S2	feature_id[18].value <= threshold=48317.708984375
node_81: feature_name=SARS.CoV.2.Spike.RBD.His.Bac	feature_id[9].value > threshold=16347.4580078125
node_111: feature_name=hCoV.NL63.S1	feature_id[5].value > threshold=4866.533447265625
node_123: feature_name=MERS.CoV.S1.RBD.367.606.rFcTag	feature_id[3].value > threshold=2789.3416748046875
node_161: feature_name=SARS.CoV.S1.HisTag	feature_id[14].value > threshold=4926.39990234375
node_179: feature_name=hCoV.HKU1.S1_S2	feature_id[22].value <= threshold=48084.783203125
node_180: feature_name=SARS.CoV.2.Spike.RBD.His.Bac	feature_id[9].value <= threshold=31607.125
node_181: feature_name=SARS.CoV.2.S1	feature_id[15].value > threshold=33718.333984375
node_191: feature_name=a-HuIgA_0.30	feature_id[27].value > threshold=1819.5
node_193: feature_name=HuIgM_0.30	feature_id[13].value > threshold=1081.8333740234375
node_197: feature_name=hCoV.HKU1.S1_S2	feature_id[22].value > threshold=44172.416015625
Class: healthcare workers within 60 days after vaccination	
Rules_77	passed counts:3
node_0: feature_name=HuIgG_0.03	feature_id[44].value > threshold=3298.333251953125
node_78: feature_name=a-HuIgG_0.03	feature_id[1].value <= threshold=19665.666015625
node_79: feature_name=SARS.CoV.2.S1.mFcTag	feature_id[0].value <= threshold=54010.166015625
node_80: feature_name=SARS.CoV.2.S1+S2	feature_id[18].value <= threshold=48317.708984375
node_81: feature_name=SARS.CoV.2.Spike.RBD.His.Bac	feature_id[9].value > threshold=16347.4580078125
node_111: feature_name=hCoV.NL63.S1	feature_id[5].value > threshold=4866.533447265625
node_123: feature_name=MERS.CoV.S1.RBD.367.606.rFcTag	feature_id[3].value > threshold=2789.3416748046875

node_161: feature_name=SARS.CoV.S1.HisTag	feature_id[14].value <= threshold=4926.39990234375
node_162: feature_name=a-MoIgA_0.10	feature_id[36].value > threshold=3108.3333740234375
Class: healthcare workers within 60 days after vaccination	
Rules_78	passed counts:3
node_0: feature_name=HuIgG_0.03	feature_id[44].value > threshold=3298.333251953125
node_78: feature_name=a-HuIgG_0.03	feature_id[1].value <= threshold=19665.666015625
node_79: feature_name=SARS.CoV.2.S1.mFcTag	feature_id[0].value <= threshold=54010.166015625
node_80: feature_name=SARS.CoV.2.S1+S2	feature_id[18].value <= threshold=48317.708984375
node_81: feature_name=SARS.CoV.2.Spike.RBD.His.Bac	feature_id[9].value > threshold=16347.4580078125
node_111: feature_name=hCoV.NL63.S1	feature_id[5].value > threshold=4866.533447265625
node_123: feature_name=MERS.CoV.S1.RBD.367.606.rFcTag	feature_id[3].value > threshold=2789.3416748046875
node_161: feature_name=SARS.CoV.S1.HisTag	feature_id[14].value <= threshold=4926.39990234375
node_162: feature_name=a-MoIgA_0.10	feature_id[36].value <= threshold=3108.3333740234375
node_163: feature_name=Flu.B_Mal/.HA1+HA2	feature_id[25].value > threshold=38684.7265625
node_167: feature_name=SARS.CoV.NP	feature_id[19].value > threshold=18293.6826171875
node_175: feature_name=Flu.B_Phu/.HA1	feature_id[11].value > threshold=37557.841796875
Class: healthcare workers within 60 days after vaccination	
Rules_79	passed counts:3
node_0: feature_name=HuIgG_0.03	feature_id[44].value > threshold=3298.333251953125
node_78: feature_name=a-HuIgG_0.03	feature_id[1].value <= threshold=19665.666015625
node_79: feature_name=SARS.CoV.2.S1.mFcTag	feature_id[0].value <= threshold=54010.166015625
node_80: feature_name=SARS.CoV.2.S1+S2	feature_id[18].value <= threshold=48317.708984375
node_81: feature_name=SARS.CoV.2.Spike.RBD.His.Bac	feature_id[9].value > threshold=16347.4580078125

node_111: feature_name=hCoV.NL63.S1	feature_id[5].value > threshold=4866.533447265625
node_123: feature_name=MERS.CoV.S1.RBD.367.606.rFcTag	feature_id[3].value > threshold=2789.3416748046875
node_161: feature_name=SARS.CoV.S1.HisTag	feature_id[14].value <= threshold=4926.39990234375
node_162: feature_name=a-MoIgA_0.10	feature_id[36].value <= threshold=3108.3333740234375
node_163: feature_name=Flu.B_Mal/.HA1+HA2	feature_id[25].value > threshold=38684.7265625
node_167: feature_name=SARS.CoV.NP	feature_id[19].value <= threshold=18293.6826171875
node_168: feature_name=a-HuIgG_0.03	feature_id[1].value <= threshold=10603.0
node_169: feature_name=a-HuIgM_0.10	feature_id[33].value > threshold=483.1666717529297
Class: healthcare workers within 60 days after vaccination	
Rules_80	passed counts:3
node_0: feature_name=HuIgG_0.03	feature_id[44].value > threshold=3298.333251953125
node_78: feature_name=a-HuIgG_0.03	feature_id[1].value <= threshold=19665.666015625
node_79: feature_name=SARS.CoV.2.S1.mFcTag	feature_id[0].value <= threshold=54010.166015625
node_80: feature_name=SARS.CoV.2.S1+S2	feature_id[18].value <= threshold=48317.708984375
node_81: feature_name=SARS.CoV.2.Spike.RBD.His.Bac	feature_id[9].value > threshold=16347.4580078125
node_111: feature_name=hCoV.NL63.S1	feature_id[5].value > threshold=4866.533447265625
node_123: feature_name=MERS.CoV.S1.RBD.367.606.rFcTag	feature_id[3].value > threshold=2789.3416748046875
node_161: feature_name=SARS.CoV.S1.HisTag	feature_id[14].value <= threshold=4926.39990234375
node_162: feature_name=a-MoIgA_0.10	feature_id[36].value <= threshold=3108.3333740234375
node_163: feature_name=Flu.B_Mal/.HA1+HA2	feature_id[25].value <= threshold=38684.7265625
node_164: feature_name=a-MoIgA_0.30	feature_id[35].value <= threshold=7878.1668701171875
Class: healthcare workers within 60 days after vaccination	
Rules_81	passed counts:3

node_0: feature_name=HuIgG_0.03	feature_id[44].value > threshold=3298.333251953125
node_78: feature_name=a-HuIgG_0.03	feature_id[1].value <= threshold=19665.666015625
node_79: feature_name=SARS.CoV.2.S1.mFcTag	feature_id[0].value <= threshold=54010.166015625
node_80: feature_name=SARS.CoV.2.S1+S2	feature_id[18].value <= threshold=48317.708984375
node_81: feature_name=SARS.CoV.2.Spike.RBD.His.Bac	feature_id[9].value > threshold=16347.4580078125
node_111: feature_name=hCoV.NL63.S1	feature_id[5].value > threshold=4866.533447265625
node_123: feature_name=MERS.CoV.S1.RBD.367.606.rFcTag	feature_id[3].value <= threshold=2789.3416748046875
node_124: feature_name=SARS.CoV.2.S1+S2	feature_id[18].value > threshold=41798.25
node_140: feature_name=SARS.CoV.2.S1.RBD.mFc	feature_id[17].value <= threshold=44491.166015625
node_141: feature_name=a-HuIgM_0.03	feature_id[34].value > threshold=-6.833333492279053
node_149: feature_name=a-MoIgA_0.30	feature_id[35].value > threshold=935.3333435058594
node_151: feature_name=HuIgM_0.30	feature_id[13].value > threshold=1110.5
node_157: feature_name=a-MoIgA_0.30	feature_id[35].value <= threshold=2098.6666259765625
Class: healthcare workers between 60 and 180 days after vaccination	
Rules_82	passed counts:3
node_0: feature_name=HuIgG_0.03	feature_id[44].value > threshold=3298.333251953125
node_78: feature_name=a-HuIgG_0.03	feature_id[1].value <= threshold=19665.666015625
node_79: feature_name=SARS.CoV.2.S1.mFcTag	feature_id[0].value <= threshold=54010.166015625
node_80: feature_name=SARS.CoV.2.S1+S2	feature_id[18].value <= threshold=48317.708984375
node_81: feature_name=SARS.CoV.2.Spike.RBD.His.Bac	feature_id[9].value > threshold=16347.4580078125
node_111: feature_name=hCoV.NL63.S1	feature_id[5].value > threshold=4866.533447265625
node_123: feature_name=MERS.CoV.S1.RBD.367.606.rFcTag	feature_id[3].value <= threshold=2789.3416748046875
node_124: feature_name=SARS.CoV.2.S1+S2	feature_id[18].value > threshold=41798.25
node_140: feature_name=SARS.CoV.2.S1.RBD.mFc	feature_id[17].value <= threshold=44491.166015625

node_141: feature_name=a-HuIgM_0.03	feature_id[34].value <= threshold=-6.833333492279053
node_142: feature_name=hCoV.OC43.HE	feature_id[10].value <= threshold=7956.5
Class: healthcare workers within 60 days after vaccination	
Rules_83	passed counts:3
node_0: feature_name=HuIgG_0.03	feature_id[44].value > threshold=3298.333251953125
node_78: feature_name=a-HuIgG_0.03	feature_id[1].value <= threshold=19665.666015625
node_79: feature_name=SARS.CoV.2.S1.mFcTag	feature_id[0].value <= threshold=54010.166015625
node_80: feature_name=SARS.CoV.2.S1+S2	feature_id[18].value <= threshold=48317.708984375
node_81: feature_name=SARS.CoV.2.Spike.RBD.His.Bac	feature_id[9].value > threshold=16347.4580078125
node_111: feature_name=hCoV.NL63.S1	feature_id[5].value > threshold=4866.533447265625
node_123: feature_name=MERS.CoV.S1.RBD.367.606.rFcTag	feature_id[3].value <= threshold=2789.3416748046875
node_124: feature_name=SARS.CoV.2.S1+S2	feature_id[18].value <= threshold=41798.25
node_125: feature_name=hCoV.OC43.S1_S2ECD.HisTag	feature_id[24].value > threshold=49554.1328125
Class: healthcare workers over 180 days after vaccination	
Rules_84	passed counts:3
node_0: feature_name=HuIgG_0.03	feature_id[44].value > threshold=3298.333251953125
node_78: feature_name=a-HuIgG_0.03	feature_id[1].value <= threshold=19665.666015625
node_79: feature_name=SARS.CoV.2.S1.mFcTag	feature_id[0].value <= threshold=54010.166015625
node_80: feature_name=SARS.CoV.2.S1+S2	feature_id[18].value <= threshold=48317.708984375
node_81: feature_name=SARS.CoV.2.Spike.RBD.His.Bac	feature_id[9].value > threshold=16347.4580078125
node_111: feature_name=hCoV.NL63.S1	feature_id[5].value > threshold=4866.533447265625
node_123: feature_name=MERS.CoV.S1.RBD.367.606.rFcTag	feature_id[3].value <= threshold=2789.3416748046875
node_124: feature_name=SARS.CoV.2.S1+S2	feature_id[18].value <= threshold=41798.25

node_125: feature_name=hCoV.OC43.S1_S2ECD.HisTag	feature_id[24].value <= threshold=49554.1328125
node_126: feature_name=SARS.CoV.2.Spike.RBD.His.Bac	feature_id[9].value <= threshold=23295.4169921875
node_127: feature_name=hCoV.OC43.S1_S2ECD.HisTag	feature_id[24].value <= threshold=28407.0830078125
Class: healthcare workers between 60 and 180 days after vaccination	
Rules_85	passed counts:3
node_0: feature_name=HuIgG_0.03	feature_id[44].value > threshold=3298.333251953125
node_78: feature_name=a-HuIgG_0.03	feature_id[1].value <= threshold=19665.666015625
node_79: feature_name=SARS.CoV.2.S1.mFcTag	feature_id[0].value <= threshold=54010.166015625
node_80: feature_name=SARS.CoV.2.S1+S2	feature_id[18].value <= threshold=48317.708984375
node_81: feature_name=SARS.CoV.2.Spike.RBD.His.Bac	feature_id[9].value <= threshold=16347.4580078125
node_82: feature_name=SARS.CoV.2.S1	feature_id[15].value > threshold=2844.6334228515625
node_90: feature_name=a-MoIgG_0.10	feature_id[38].value > threshold=1482.3333129882812
node_98: feature_name=Flu.H1N1.HA1	feature_id[26].value > threshold=52934.66015625
node_108: feature_name=a-MoIgA_0.03	feature_id[37].value > threshold=26.999999046325684
Class: unvaccinated healthcare workers	
Rules_86	passed counts:3
node_0: feature_name=HuIgG_0.03	feature_id[44].value > threshold=3298.333251953125
node_78: feature_name=a-HuIgG_0.03	feature_id[1].value <= threshold=19665.666015625
node_79: feature_name=SARS.CoV.2.S1.mFcTag	feature_id[0].value <= threshold=54010.166015625
node_80: feature_name=SARS.CoV.2.S1+S2	feature_id[18].value <= threshold=48317.708984375
node_81: feature_name=SARS.CoV.2.Spike.RBD.His.Bac	feature_id[9].value <= threshold=16347.4580078125
node_82: feature_name=SARS.CoV.2.S1	feature_id[15].value > threshold=2844.6334228515625
node_90: feature_name=a-MoIgG_0.10	feature_id[38].value <= threshold=1482.3333129882812

node_91: feature_name=SARS.CoV.2.Spike.RBD.rFc	feature_id[7].value > threshold=6995.25
Class: healthcare workers over 180 days after vaccination	
Rules_87	passed counts:3
node_0: feature_name=HuIgG_0.03	feature_id[44].value <= threshold=3298.333251953125
node_1: feature_name=SARS.CoV.2.S1.mFcTag	feature_id[0].value > threshold=5354.388916015625
node_9: feature_name=HuIgM_0.03	feature_id[45].value > threshold=-80.66666793823242
node_27: feature_name=HuIgM_0.03	feature_id[45].value <= threshold=61.33333206176758
node_28: feature_name=a-HuIgG_0.30	feature_id[30].value > threshold=34878.66796875
node_64: feature_name=a-HuIgG_0.03	feature_id[1].value <= threshold=9340.3330078125
node_65: feature_name=SARS.CoV.2.Spike.RBD.His.Bac	feature_id[9].value <= threshold=13221.6298828125
Class: unvaccinated healthcare workers	
Rules_88	passed counts:3
node_0: feature_name=HuIgG_0.03	feature_id[44].value <= threshold=3298.333251953125
node_1: feature_name=SARS.CoV.2.S1.mFcTag	feature_id[0].value > threshold=5354.388916015625
node_9: feature_name=HuIgM_0.03	feature_id[45].value > threshold=-80.66666793823242
node_27: feature_name=HuIgM_0.03	feature_id[45].value <= threshold=61.33333206176758
node_28: feature_name=a-HuIgG_0.30	feature_id[30].value <= threshold=34878.66796875
node_29: feature_name=HuIgG_0.03	feature_id[44].value > threshold=769.8333435058594
node_35: feature_name=SARS.CoV.S1.HisTag	feature_id[14].value > threshold=400.3000030517578
node_39: feature_name=hCoV.HKU1.S1_S2	feature_id[22].value <= threshold=42662.666015625
node_40: feature_name=Flu.B_Phu/.HA1	feature_id[11].value <= threshold=49118.416015625
node_41: feature_name=hCoV.OC43.HE	feature_id[10].value <= threshold=25184.06640625
node_42: feature_name=HuIgM_0.30	feature_id[13].value <= threshold=1223.5

node_43: feature_name=a-HuIgA_0.03	feature_id[29].value > threshold=-59.66666603088379
node_47: feature_name=SARS.CoV.S1.HisTag	feature_id[14].value > threshold=25028.0
node_53: feature_name=a-HuIgA_0.30	feature_id[27].value > threshold=976.3333435058594
Class: healthcare workers between 60 and 180 days after vaccination	
Rules_89	passed counts:3
node_0: feature_name=HuIgG_0.03	feature_id[44].value <= threshold=3298.333251953125
node_1: feature_name=SARS.CoV.2.S1.mFcTag	feature_id[0].value > threshold=5354.388916015625
node_9: feature_name=HuIgM_0.03	feature_id[45].value > threshold=-80.66666793823242
node_27: feature_name=HuIgM_0.03	feature_id[45].value <= threshold=61.33333206176758
node_28: feature_name=a-HuIgG_0.30	feature_id[30].value <= threshold=34878.66796875
node_29: feature_name=HuIgG_0.03	feature_id[44].value > threshold=769.8333435058594
node_35: feature_name=SARS.CoV.S1.HisTag	feature_id[14].value > threshold=400.3000030517578
node_39: feature_name=hCoV.HKU1.S1_S2	feature_id[22].value <= threshold=42662.666015625
node_40: feature_name=Flu.B_Phu/.HA1	feature_id[11].value <= threshold=49118.416015625
node_41: feature_name=hCoV.OC43.HE	feature_id[10].value <= threshold=25184.06640625
node_42: feature_name=HuIgM_0.30	feature_id[13].value <= threshold=1223.5
node_43: feature_name=a-HuIgA_0.03	feature_id[29].value > threshold=-59.66666603088379
node_47: feature_name=SARS.CoV.S1.HisTag	feature_id[14].value <= threshold=25028.0
node_48: feature_name=HuIgM_0.03	feature_id[45].value > threshold=35.33333206176758
node_50: feature_name=Flu.H1N1.HA1	feature_id[26].value <= threshold=36011.291015625
Class: healthcare workers between 60 and 180 days after vaccination	
Rules_90	passed counts:3
node_0: feature_name=HuIgG_0.03	feature_id[44].value <= threshold=3298.333251953125

node_1: feature_name=SARS.CoV.2.S1.mFcTag	feature_id[0].value > threshold=5354.388916015625
node_9: feature_name=HuIgM_0.03	feature_id[45].value <= threshold=-80.66666793823242
node_10: feature_name=SARS.CoV.2.Spike.RBD.His.Bac	feature_id[9].value > threshold=29393.55859375
node_18: feature_name=Flu.B_Mal/.HA1	feature_id[4].value > threshold=38994.25
node_20: feature_name=SARS.CoV.S1.HisTag	feature_id[14].value <= threshold=11923.35009765625
node_21: feature_name=SARS.CoV.2.Spike.RBD.rFc	feature_id[7].value > threshold=44823.91796875
Class: healthcare workers between 60 and 180 days after vaccination	
Rules_91	passed counts:3
node_0: feature_name=HuIgG_0.03	feature_id[44].value <= threshold=3298.333251953125
node_1: feature_name=SARS.CoV.2.S1.mFcTag	feature_id[0].value <= threshold=5354.388916015625
node_2: feature_name=SARS.CoV.2.S1.HisTag	feature_id[16].value > threshold=-383.86944580078125
node_4: feature_name=hCoV.OC43.HE	feature_id[10].value <= threshold=414.5370330810547
node_5: feature_name=SARS.CoV.2.S1.HisTag	feature_id[16].value > threshold=1.5555554628372192
Class: unvaccinated healthcare workers	
Rules_92	passed counts:2
node_0: feature_name=HuIgG_0.03	feature_id[44].value > threshold=3298.333251953125
node_78: feature_name=a-HuIgG_0.03	feature_id[1].value > threshold=19665.666015625
node_360: feature_name=HuIgM_0.03	feature_id[45].value > threshold=-189.0
node_366: feature_name=HuIgM_0.30	feature_id[13].value > threshold=441.8333282470703
node_368: feature_name=a-HuIgG_0.10	feature_id[31].value > threshold=32914.5
node_372: feature_name=a-MoIgG_0.10	feature_id[38].value > threshold=20281.6669921875
Class: healthcare workers within 60 days after vaccination	

Rules_93	passed counts:2
node_0: feature_name=HuIgG_0.03	feature_id[44].value > threshold=3298.333251953125
node_78: feature_name=a-HuIgG_0.03	feature_id[1].value > threshold=19665.666015625
node_360: feature_name=HuIgM_0.03	feature_id[45].value > threshold=-189.0
node_366: feature_name=HuIgM_0.30	feature_id[13].value > threshold=441.8333282470703
node_368: feature_name=a-HuIgG_0.10	feature_id[31].value > threshold=32914.5
node_372: feature_name=a-MoIgG_0.10	feature_id[38].value <= threshold=20281.6669921875
node_373: feature_name=hCoV.229E.S1	feature_id[2].value > threshold=54372.5
node_375: feature_name=hCoV.HKU1.S1_S2	feature_id[22].value > threshold=44467.44921875
Class: healthcare workers between 60 and 180 days after vaccination	
Rules_94	passed counts:2
node_0: feature_name=HuIgG_0.03	feature_id[44].value > threshold=3298.333251953125
node_78: feature_name=a-HuIgG_0.03	feature_id[1].value > threshold=19665.666015625
node_360: feature_name=HuIgM_0.03	feature_id[45].value > threshold=-189.0
node_366: feature_name=HuIgM_0.30	feature_id[13].value > threshold=441.8333282470703
node_368: feature_name=a-HuIgG_0.10	feature_id[31].value <= threshold=32914.5
node_369: feature_name=MERS.CoV.S1.RBD.367.606.rFcTag	feature_id[3].value > threshold=6711.89990234375
Class: healthcare workers over 180 days after vaccination	
Rules_95	passed counts:2
node_0: feature_name=HuIgG_0.03	feature_id[44].value > threshold=3298.333251953125
node_78: feature_name=a-HuIgG_0.03	feature_id[1].value <= threshold=19665.666015625
node_79: feature_name=SARS.CoV.2.S1.mFcTag	feature_id[0].value > threshold=54010.166015625
node_297: feature_name=a-MoIgG_0.10	feature_id[38].value <= threshold=2382.8333740234375

node_298: feature_name=Flu.B_Mal/.HA1	feature_id[4].value <= threshold=41908.115234375
node_299: feature_name=a-HuIgA_0.10	feature_id[28].value > threshold=164.00000381469727
Class: healthcare workers between 60 and 180 days after vaccination	
Rules_96	passed counts:2
node_0: feature_name=HuIgG_0.03	feature_id[44].value > threshold=3298.333251953125
node_78: feature_name=a-HuIgG_0.03	feature_id[1].value <= threshold=19665.666015625
node_79: feature_name=SARS.CoV.2.S1.mFcTag	feature_id[0].value <= threshold=54010.166015625
node_80: feature_name=SARS.CoV.2.S1+S2	feature_id[18].value > threshold=48317.708984375
node_224: feature_name=a-MoIgG_0.10	feature_id[38].value > threshold=2066.8333129882812
node_226: feature_name=HuIgM_0.30	feature_id[13].value > threshold=750.1666870117188
node_238: feature_name=HuIgM_0.30	feature_id[13].value > threshold=760.3333435058594
node_240: feature_name=hCoV.OC43.HE	feature_id[10].value > threshold=3879.75
node_246: feature_name=a-HuIgA_0.30	feature_id[27].value > threshold=1379.8333740234375
node_248: feature_name=Flu.H1N1.HA1	feature_id[26].value > threshold=53891.083984375
Class: healthcare workers between 60 and 180 days after vaccination	
Rules_97	passed counts:2
node_0: feature_name=HuIgG_0.03	feature_id[44].value > threshold=3298.333251953125
node_78: feature_name=a-HuIgG_0.03	feature_id[1].value <= threshold=19665.666015625
node_79: feature_name=SARS.CoV.2.S1.mFcTag	feature_id[0].value <= threshold=54010.166015625
node_80: feature_name=SARS.CoV.2.S1+S2	feature_id[18].value > threshold=48317.708984375
node_224: feature_name=a-MoIgG_0.10	feature_id[38].value > threshold=2066.8333129882812
node_226: feature_name=HuIgM_0.30	feature_id[13].value > threshold=750.1666870117188
node_238: feature_name=HuIgM_0.30	feature_id[13].value > threshold=760.3333435058594

node_240: feature_name=hCoV.OC43.HE	feature_id[10].value > threshold=3879.75
node_246: feature_name=a-HuIgA_0.30	feature_id[27].value > threshold=1379.8333740234375
node_248: feature_name=Flu.H1N1.HA1	feature_id[26].value <= threshold=53891.083984375
node_249: feature_name=MERS.CoV.S1.RBD.367.606.rFcTag	feature_id[3].value > threshold=1486.7666625976562
node_253: feature_name=HuIgM_0.03	feature_id[45].value > threshold=-56.33333396911621
node_279: feature_name=hCoV.229E.S1	feature_id[2].value <= threshold=51743.583984375
node_280: feature_name=Flu.B_Mal/.HA1	feature_id[4].value > threshold=48206.833984375
node_290: feature_name=MERS.CoV.S1.ECD.1-1297.HisTag	feature_id[12].value > threshold=25489.541015625
Class: healthcare workers within 60 days after vaccination	
Rules_98	passed counts:2
node_0: feature_name=HuIgG_0.03	feature_id[44].value > threshold=3298.333251953125
node_78: feature_name=a-HuIgG_0.03	feature_id[1].value <= threshold=19665.666015625
node_79: feature_name=SARS.CoV.2.S1.mFcTag	feature_id[0].value <= threshold=54010.166015625
node_80: feature_name=SARS.CoV.2.S1+S2	feature_id[18].value > threshold=48317.708984375
node_224: feature_name=a-MoIgG_0.10	feature_id[38].value > threshold=2066.8333129882812
node_226: feature_name=HuIgM_0.30	feature_id[13].value > threshold=750.1666870117188
node_238: feature_name=HuIgM_0.30	feature_id[13].value > threshold=760.3333435058594
node_240: feature_name=hCoV.OC43.HE	feature_id[10].value > threshold=3879.75
node_246: feature_name=a-HuIgA_0.30	feature_id[27].value <= threshold=1379.8333740234375
Class: healthcare workers between 60 and 180 days after vaccination	
Rules_99	passed counts:2
node_0: feature_name=HuIgG_0.03	feature_id[44].value > threshold=3298.333251953125
node_78: feature_name=a-HuIgG_0.03	feature_id[1].value <= threshold=19665.666015625

node_79: feature_name=SARS.CoV.2.S1.mFcTag	feature_id[0].value <= threshold=54010.166015625
node_80: feature_name=SARS.CoV.2.S1+S2	feature_id[18].value > threshold=48317.708984375
node_224: feature_name=a-MoIgG_0.10	feature_id[38].value > threshold=2066.8333129882812
node_226: feature_name=HuIgM_0.30	feature_id[13].value > threshold=750.1666870117188
node_238: feature_name=HuIgM_0.30	feature_id[13].value > threshold=760.3333435058594
node_240: feature_name=hCoV.OC43.HE	feature_id[10].value <= threshold=3879.75
node_241: feature_name=a-HuIgM_0.10	feature_id[33].value > threshold=899.8333435058594
node_243: feature_name=HuIgA_0.10	feature_id[42].value <= threshold=138.1666717529297
Class: healthcare workers within 60 days after vaccination	
Rules_100	passed counts:2
node_0: feature_name=HuIgG_0.03	feature_id[44].value > threshold=3298.333251953125
node_78: feature_name=a-HuIgG_0.03	feature_id[1].value <= threshold=19665.666015625
node_79: feature_name=SARS.CoV.2.S1.mFcTag	feature_id[0].value <= threshold=54010.166015625
node_80: feature_name=SARS.CoV.2.S1+S2	feature_id[18].value <= threshold=48317.708984375
node_81: feature_name=SARS.CoV.2.Spike.RBD.His.Bac	feature_id[9].value > threshold=16347.4580078125
node_111: feature_name=hCoV.NL63.S1	feature_id[5].value > threshold=4866.533447265625
node_123: feature_name=MERS.CoV.S1.RBD.367.606.rFcTag	feature_id[3].value > threshold=2789.3416748046875
node_161: feature_name=SARS.CoV.S1.HisTag	feature_id[14].value > threshold=4926.39990234375
node_179: feature_name=hCoV.HKU1.S1_S2	feature_id[22].value <= threshold=48084.783203125
node_180: feature_name=SARS.CoV.2.Spike.RBD.His.Bac	feature_id[9].value > threshold=31607.125
node_200: feature_name=SARS.CoV.2.S1.HisTag	feature_id[16].value > threshold=43902.201171875
node_214: feature_name=SARS.CoV.2.S1.mFcTag	feature_id[0].value <= threshold=51362.833984375
Class: healthcare workers between 60 and 180 days after vaccination	

Rules_101	passed counts:2
node_0: feature_name=HuIgG_0.03	feature_id[44].value > threshold=3298.333251953125
node_78: feature_name=a-HuIgG_0.03	feature_id[1].value <= threshold=19665.666015625
node_79: feature_name=SARS.CoV.2.S1.mFcTag	feature_id[0].value <= threshold=54010.166015625
node_80: feature_name=SARS.CoV.2.S1+S2	feature_id[18].value <= threshold=48317.708984375
node_81: feature_name=SARS.CoV.2.Spike.RBD.His.Bac	feature_id[9].value > threshold=16347.4580078125
node_111: feature_name=hCoV.NL63.S1	feature_id[5].value > threshold=4866.533447265625
node_123: feature_name=MERS.CoV.S1.RBD.367.606.rFcTag	feature_id[3].value > threshold=2789.3416748046875
node_161: feature_name=SARS.CoV.S1.HisTag	feature_id[14].value > threshold=4926.39990234375
node_179: feature_name=hCoV.HKU1.S1_S2	feature_id[22].value <= threshold=48084.783203125
node_180: feature_name=SARS.CoV.2.Spike.RBD.His.Bac	feature_id[9].value > threshold=31607.125
node_200: feature_name=SARS.CoV.2.S1.HisTag	feature_id[16].value <= threshold=43902.201171875
node_201: feature_name=Flu.H1N1.HA1+HA2	feature_id[6].value <= threshold=30743.5
node_202: feature_name=a-HuIgG_0.30	feature_id[30].value > threshold=42035.166015625
Class: healthcare workers within 60 days after vaccination	
Rules_102	passed counts:2
node_0: feature_name=HuIgG_0.03	feature_id[44].value > threshold=3298.333251953125
node_78: feature_name=a-HuIgG_0.03	feature_id[1].value <= threshold=19665.666015625
node_79: feature_name=SARS.CoV.2.S1.mFcTag	feature_id[0].value <= threshold=54010.166015625
node_80: feature_name=SARS.CoV.2.S1+S2	feature_id[18].value <= threshold=48317.708984375
node_81: feature_name=SARS.CoV.2.Spike.RBD.His.Bac	feature_id[9].value > threshold=16347.4580078125
node_111: feature_name=hCoV.NL63.S1	feature_id[5].value > threshold=4866.533447265625
node_123: feature_name=MERS.CoV.S1.RBD.367.606.rFcTag	feature_id[3].value > threshold=2789.3416748046875
node_161: feature_name=SARS.CoV.S1.HisTag	feature_id[14].value > threshold=4926.39990234375

node_179: feature_name=hCoV.HKU1.S1_S2	feature_id[22].value <= threshold=48084.783203125
node_180: feature_name=SARS.CoV.2.Spike.RBD.His.Bac	feature_id[9].value <= threshold=31607.125
node_181: feature_name=SARS.CoV.2.S1	feature_id[15].value <= threshold=33718.333984375
node_182: feature_name=a-HuIgG_0.03	feature_id[1].value > threshold=15402.5
node_188: feature_name=a-MoIgA_0.10	feature_id[36].value > threshold=5932.166656494141
Class: healthcare workers within 60 days after vaccination	
Rules_103	passed counts:2
node_0: feature_name=HuIgG_0.03	feature_id[44].value > threshold=3298.333251953125
node_78: feature_name=a-HuIgG_0.03	feature_id[1].value <= threshold=19665.666015625
node_79: feature_name=SARS.CoV.2.S1.mFcTag	feature_id[0].value <= threshold=54010.166015625
node_80: feature_name=SARS.CoV.2.S1+S2	feature_id[18].value <= threshold=48317.708984375
node_81: feature_name=SARS.CoV.2.Spike.RBD.His.Bac	feature_id[9].value > threshold=16347.4580078125
node_111: feature_name=hCoV.NL63.S1	feature_id[5].value > threshold=4866.533447265625
node_123: feature_name=MERS.CoV.S1.RBD.367.606.rFcTag	feature_id[3].value > threshold=2789.3416748046875
node_161: feature_name=SARS.CoV.S1.HisTag	feature_id[14].value > threshold=4926.39990234375
node_179: feature_name=hCoV.HKU1.S1_S2	feature_id[22].value <= threshold=48084.783203125
node_180: feature_name=SARS.CoV.2.Spike.RBD.His.Bac	feature_id[9].value <= threshold=31607.125
node_181: feature_name=SARS.CoV.2.S1	feature_id[15].value <= threshold=33718.333984375
node_182: feature_name=a-HuIgG_0.03	feature_id[1].value > threshold=15402.5
node_188: feature_name=a-MoIgA_0.10	feature_id[36].value <= threshold=5932.166656494141
Class: healthcare workers over 180 days after vaccination	
Rules_104	passed counts:2
node_0: feature_name=HuIgG_0.03	feature_id[44].value > threshold=3298.333251953125

node_78: feature_name=a-HuIgG_0.03	feature_id[1].value <= threshold=19665.666015625
node_79: feature_name=SARS.CoV.2.S1.mFcTag	feature_id[0].value <= threshold=54010.166015625
node_80: feature_name=SARS.CoV.2.S1+S2	feature_id[18].value <= threshold=48317.708984375
node_81: feature_name=SARS.CoV.2.Spike.RBD.His.Bac	feature_id[9].value > threshold=16347.4580078125
node_111: feature_name=hCoV.NL63.S1	feature_id[5].value > threshold=4866.533447265625
node_123: feature_name=MERS.CoV.S1.RBD.367.606.rFcTag	feature_id[3].value > threshold=2789.3416748046875
node_161: feature_name=SARS.CoV.S1.HisTag	feature_id[14].value > threshold=4926.39990234375
node_179: feature_name=hCoV.HKU1.S1_S2	feature_id[22].value <= threshold=48084.783203125
node_180: feature_name=SARS.CoV.2.Spike.RBD.His.Bac	feature_id[9].value <= threshold=31607.125
node_181: feature_name=SARS.CoV.2.S1	feature_id[15].value <= threshold=33718.333984375
node_182: feature_name=a-HuIgG_0.03	feature_id[1].value <= threshold=15402.5
node_183: feature_name=a-HuIgM_0.30	feature_id[32].value <= threshold=1969.0000610351562
Class: healthcare workers within 60 days after vaccination	
Rules_105	passed counts:2
node_0: feature_name=HuIgG_0.03	feature_id[44].value > threshold=3298.333251953125
node_78: feature_name=a-HuIgG_0.03	feature_id[1].value <= threshold=19665.666015625
node_79: feature_name=SARS.CoV.2.S1.mFcTag	feature_id[0].value <= threshold=54010.166015625
node_80: feature_name=SARS.CoV.2.S1+S2	feature_id[18].value <= threshold=48317.708984375
node_81: feature_name=SARS.CoV.2.Spike.RBD.His.Bac	feature_id[9].value > threshold=16347.4580078125
node_111: feature_name=hCoV.NL63.S1	feature_id[5].value > threshold=4866.533447265625
node_123: feature_name=MERS.CoV.S1.RBD.367.606.rFcTag	feature_id[3].value <= threshold=2789.3416748046875
node_124: feature_name=SARS.CoV.2.S1+S2	feature_id[18].value > threshold=41798.25
node_140: feature_name=SARS.CoV.2.S1.RBD.mFc	feature_id[17].value <= threshold=44491.166015625
node_141: feature_name=a-HuIgM_0.03	feature_id[34].value > threshold=-6.833333492279053

node_149: feature_name=a-MoIgA_0.30	feature_id[35].value <= threshold=935.3333435058594
Class: healthcare workers between 60 and 180 days after vaccination	
Rules_106	passed counts:2
node_0: feature_name=HuIgG_0.03	feature_id[44].value > threshold=3298.333251953125
node_78: feature_name=a-HuIgG_0.03	feature_id[1].value <= threshold=19665.666015625
node_79: feature_name=SARS.CoV.2.S1.mFcTag	feature_id[0].value <= threshold=54010.166015625
node_80: feature_name=SARS.CoV.2.S1+S2	feature_id[18].value <= threshold=48317.708984375
node_81: feature_name=SARS.CoV.2.Spike.RBD.His.Bac	feature_id[9].value > threshold=16347.4580078125
node_111: feature_name=hCoV.NL63.S1	feature_id[5].value > threshold=4866.533447265625
node_123: feature_name=MERS.CoV.S1.RBD.367.606.rFcTag	feature_id[3].value <= threshold=2789.3416748046875
node_124: feature_name=SARS.CoV.2.S1+S2	feature_id[18].value <= threshold=41798.25
node_125: feature_name=hCoV.OC43.S1_S2ECD.HisTag	feature_id[24].value <= threshold=49554.1328125
node_126: feature_name=SARS.CoV.2.Spike.RBD.His.Bac	feature_id[9].value > threshold=23295.4169921875
node_130: feature_name=Flu.H1N1.HA1	feature_id[26].value <= threshold=30553.0
node_131: feature_name=MERS.CoV.S1.ECD.1-1297.HisTag	feature_id[12].value > threshold=13902.69140625
Class: healthcare workers within 60 days after vaccination	
Rules_107	passed counts:2
node_0: feature_name=HuIgG_0.03	feature_id[44].value > threshold=3298.333251953125
node_78: feature_name=a-HuIgG_0.03	feature_id[1].value <= threshold=19665.666015625
node_79: feature_name=SARS.CoV.2.S1.mFcTag	feature_id[0].value <= threshold=54010.166015625
node_80: feature_name=SARS.CoV.2.S1+S2	feature_id[18].value <= threshold=48317.708984375
node_81: feature_name=SARS.CoV.2.Spike.RBD.His.Bac	feature_id[9].value > threshold=16347.4580078125
node_111: feature_name=hCoV.NL63.S1	feature_id[5].value <= threshold=4866.533447265625

node_112: feature_name=SARS.CoV.2.S1.mFcTag	feature_id[0].value > threshold=40690.80078125
node_116: feature_name=a-MoIgG_0.10	feature_id[38].value <= threshold=2424.6666259765625
Class: healthcare workers between 60 and 180 days after vaccination	
Rules_108	passed counts:2
node_0: feature_name=HuIgG_0.03	feature_id[44].value > threshold=3298.333251953125
node_78: feature_name=a-HuIgG_0.03	feature_id[1].value <= threshold=19665.666015625
node_79: feature_name=SARS.CoV.2.S1.mFcTag	feature_id[0].value <= threshold=54010.166015625
node_80: feature_name=SARS.CoV.2.S1+S2	feature_id[18].value <= threshold=48317.708984375
node_81: feature_name=SARS.CoV.2.Spike.RBD.His.Bac	feature_id[9].value <= threshold=16347.4580078125
node_82: feature_name=SARS.CoV.2.S1	feature_id[15].value > threshold=2844.6334228515625
node_90: feature_name=a-MoIgG_0.10	feature_id[38].value > threshold=1482.3333129882812
node_98: feature_name=Flu.H1N1.HA1	feature_id[26].value <= threshold=52934.66015625
node_99: feature_name=Flu.B_Phu/.HA1	feature_id[11].value <= threshold=27915.5419921875
node_100: feature_name=hCoV.229E.S1	feature_id[2].value > threshold=33022.865234375
Class: unvaccinated healthcare workers	
Rules_109	passed counts:2
node_0: feature_name=HuIgG_0.03	feature_id[44].value > threshold=3298.333251953125
node_78: feature_name=a-HuIgG_0.03	feature_id[1].value <= threshold=19665.666015625
node_79: feature_name=SARS.CoV.2.S1.mFcTag	feature_id[0].value <= threshold=54010.166015625
node_80: feature_name=SARS.CoV.2.S1+S2	feature_id[18].value <= threshold=48317.708984375
node_81: feature_name=SARS.CoV.2.Spike.RBD.His.Bac	feature_id[9].value <= threshold=16347.4580078125
node_82: feature_name=SARS.CoV.2.S1	feature_id[15].value > threshold=2844.6334228515625
node_90: feature_name=a-MoIgG_0.10	feature_id[38].value > threshold=1482.3333129882812

node_98: feature_name=Flu.H1N1.HA1	feature_id[26].value <= threshold=52934.66015625
node_99: feature_name=Flu.B_Phu/.HA1	feature_id[11].value <= threshold=27915.5419921875
node_100: feature_name=hCoV.229E.S1	feature_id[2].value <= threshold=33022.865234375
node_101: feature_name=SARS.CoV.2.S1.mFcTag	feature_id[0].value <= threshold=25623.3671875
Class: healthcare workers within 60 days after vaccination	
Rules_110	passed counts:2
node_0: feature_name=HuIgG_0.03	feature_id[44].value <= threshold=3298.333251953125
node_1: feature_name=SARS.CoV.2.S1.mFcTag	feature_id[0].value > threshold=5354.388916015625
node_9: feature_name=HuIgM_0.03	feature_id[45].value > threshold=-80.66666793823242
node_27: feature_name=HuIgM_0.03	feature_id[45].value <= threshold=61.33333206176758
node_28: feature_name=a-HuIgG_0.30	feature_id[30].value <= threshold=34878.66796875
node_29: feature_name=HuIgG_0.03	feature_id[44].value > threshold=769.8333435058594
node_35: feature_name=SARS.CoV.S1.HisTag	feature_id[14].value > threshold=400.3000030517578
node_39: feature_name=hCoV.HKU1.S1_S2	feature_id[22].value > threshold=42662.666015625
node_61: feature_name=SARS.CoV.2.S1.HisTag	feature_id[16].value <= threshold=41690.958984375
Class: healthcare workers within 60 days after vaccination	
Rules_111	passed counts:2
node_0: feature_name=HuIgG_0.03	feature_id[44].value <= threshold=3298.333251953125
node_1: feature_name=SARS.CoV.2.S1.mFcTag	feature_id[0].value > threshold=5354.388916015625
node_9: feature_name=HuIgM_0.03	feature_id[45].value > threshold=-80.66666793823242
node_27: feature_name=HuIgM_0.03	feature_id[45].value <= threshold=61.33333206176758
node_28: feature_name=a-HuIgG_0.30	feature_id[30].value <= threshold=34878.66796875
node_29: feature_name=HuIgG_0.03	feature_id[44].value > threshold=769.8333435058594

node_35: feature_name=SARS.CoV.S1.HisTag	feature_id[14].value > threshold=400.3000030517578
node_39: feature_name=hCoV.HKU1.S1_S2	feature_id[22].value <= threshold=42662.666015625
node_40: feature_name=Flu.B_Phu/.HA1	feature_id[11].value <= threshold=49118.416015625
node_41: feature_name=hCoV.OC43.HE	feature_id[10].value <= threshold=25184.06640625
node_42: feature_name=HuIgM_0.30	feature_id[13].value <= threshold=1223.5
node_43: feature_name=a-HuIgA_0.03	feature_id[29].value <= threshold=-59.66666603088379
node_44: feature_name=Flu.B_Phu/.HA1	feature_id[11].value <= threshold=28467.833984375
Class: healthcare workers between 60 and 180 days after vaccination	
Rules_112	passed counts:2
node_0: feature_name=HuIgG_0.03	feature_id[44].value <= threshold=3298.333251953125
node_1: feature_name=SARS.CoV.2.S1.mFcTag	feature_id[0].value > threshold=5354.388916015625
node_9: feature_name=HuIgM_0.03	feature_id[45].value > threshold=-80.66666793823242
node_27: feature_name=HuIgM_0.03	feature_id[45].value <= threshold=61.33333206176758
node_28: feature_name=a-HuIgG_0.30	feature_id[30].value <= threshold=34878.66796875
node_29: feature_name=HuIgG_0.03	feature_id[44].value > threshold=769.8333435058594
node_35: feature_name=SARS.CoV.S1.HisTag	feature_id[14].value <= threshold=400.3000030517578
node_36: feature_name=HuIgA_0.10	feature_id[42].value <= threshold=4194.666564941406
Class: unvaccinated healthcare workers	
Rules_113	passed counts:2
node_0: feature_name=HuIgG_0.03	feature_id[44].value <= threshold=3298.333251953125
node_1: feature_name=SARS.CoV.2.S1.mFcTag	feature_id[0].value > threshold=5354.388916015625
node_9: feature_name=HuIgM_0.03	feature_id[45].value <= threshold=-80.66666793823242
node_10: feature_name=SARS.CoV.2.Spike.RBD.His.Bac	feature_id[9].value <= threshold=29393.55859375

node_11: feature_name=DcCoV.HKU23.NP	feature_id[21].value > threshold=-639.3333129882812
node_13: feature_name=MERS.CoV.S1.ECD.1-1297.HisTag	feature_id[12].value <= threshold=1026.1666564941406
node_14: feature_name=SARS.CoV.2.S1+S2	feature_id[18].value <= threshold=14681.66650390625
Class: healthcare workers over 180 days after vaccination	
Rules_114	passed counts:1
node_0: feature_name=HuIgG_0.03	feature_id[44].value > threshold=3298.333251953125
node_78: feature_name=a-HuIgG_0.03	feature_id[1].value > threshold=19665.666015625
node_360: feature_name=HuIgM_0.03	feature_id[45].value > threshold=-189.0
node_366: feature_name=HuIgM_0.30	feature_id[13].value > threshold=441.8333282470703
node_368: feature_name=a-HuIgG_0.10	feature_id[31].value > threshold=32914.5
node_372: feature_name=a-MoIgG_0.10	feature_id[38].value <= threshold=20281.6669921875
node_373: feature_name=hCoV.229E.S1	feature_id[2].value > threshold=54372.5
node_375: feature_name=hCoV.HKU1.S1_S2	feature_id[22].value <= threshold=44467.44921875
Class: healthcare workers over 180 days after vaccination	
Rules_115	passed counts:1
node_0: feature_name=HuIgG_0.03	feature_id[44].value > threshold=3298.333251953125
node_78: feature_name=a-HuIgG_0.03	feature_id[1].value > threshold=19665.666015625
node_360: feature_name=HuIgM_0.03	feature_id[45].value <= threshold=-189.0
node_361: feature_name=a-HuIgA_0.03	feature_id[29].value > threshold=3214.5
node_363: feature_name=MoIgA_0.30	feature_id[46].value > threshold=4640.5001220703125
Class: healthcare workers within 60 days after vaccination	
Rules_116	passed counts:1

node_0: feature_name=HuIgG_0.03	feature_id[44].value > threshold=3298.333251953125
node_78: feature_name=a-HuIgG_0.03	feature_id[1].value > threshold=19665.666015625
node_360: feature_name=HuIgM_0.03	feature_id[45].value <= threshold=-189.0
node_361: feature_name=a-HuIgA_0.03	feature_id[29].value > threshold=3214.5
node_363: feature_name=MoIgA_0.30	feature_id[46].value <= threshold=4640.5001220703125
Class: healthcare workers over 180 days after vaccination	
Rules_117	passed counts:1
node_0: feature_name=HuIgG_0.03	feature_id[44].value > threshold=3298.333251953125
node_78: feature_name=a-HuIgG_0.03	feature_id[1].value <= threshold=19665.666015625
node_79: feature_name=SARS.CoV.2.S1.mFcTag	feature_id[0].value > threshold=54010.166015625
node_297: feature_name=a-MoIgG_0.10	feature_id[38].value > threshold=2382.8333740234375
node_303: feature_name=SARS.CoV.2.S1	feature_id[15].value > threshold=51713.66796875
node_345: feature_name=SARS.CoV.2.S1.HisTag	feature_id[16].value > threshold=51766.75
node_347: feature_name=HuIgA_0.03	feature_id[43].value > threshold=-60.33333396911621
node_353: feature_name=SARS.CoV.2.Spike.RBD.His.Bac	feature_id[9].value <= threshold=51551.166015625
node_354: feature_name=SARS.CoV.NP	feature_id[19].value <= threshold=3082.949951171875
Class: healthcare workers between 60 and 180 days after vaccination	
Rules_118	passed counts:1
node_0: feature_name=HuIgG_0.03	feature_id[44].value > threshold=3298.333251953125
node_78: feature_name=a-HuIgG_0.03	feature_id[1].value <= threshold=19665.666015625
node_79: feature_name=SARS.CoV.2.S1.mFcTag	feature_id[0].value > threshold=54010.166015625
node_297: feature_name=a-MoIgG_0.10	feature_id[38].value > threshold=2382.8333740234375
node_303: feature_name=SARS.CoV.2.S1	feature_id[15].value > threshold=51713.66796875

node_345: feature_name=SARS.CoV.2.S1.HisTag	feature_id[16].value > threshold=51766.75
node_347: feature_name=HuIgA_0.03	feature_id[43].value <= threshold=-60.33333396911621
node_348: feature_name=hCoV.HKU1.S1_S2	feature_id[22].value > threshold=51287.134765625
node_350: feature_name=a-HuIgG_0.03	feature_id[1].value > threshold=17172.0
Class: unvaccinated healthcare workers	
Rules_119	passed counts:1
node_0: feature_name=HuIgG_0.03	feature_id[44].value > threshold=3298.333251953125
node_78: feature_name=a-HuIgG_0.03	feature_id[1].value <= threshold=19665.666015625
node_79: feature_name=SARS.CoV.2.S1.mFcTag	feature_id[0].value > threshold=54010.166015625
node_297: feature_name=a-MoIgG_0.10	feature_id[38].value > threshold=2382.8333740234375
node_303: feature_name=SARS.CoV.2.S1	feature_id[15].value > threshold=51713.66796875
node_345: feature_name=SARS.CoV.2.S1.HisTag	feature_id[16].value > threshold=51766.75
node_347: feature_name=HuIgA_0.03	feature_id[43].value <= threshold=-60.33333396911621
node_348: feature_name=hCoV.HKU1.S1_S2	feature_id[22].value > threshold=51287.134765625
node_350: feature_name=a-HuIgG_0.03	feature_id[1].value <= threshold=17172.0
Class: healthcare workers within 60 days after vaccination	
Rules_120	passed counts:1
node_0: feature_name=HuIgG_0.03	feature_id[44].value > threshold=3298.333251953125
node_78: feature_name=a-HuIgG_0.03	feature_id[1].value <= threshold=19665.666015625
node_79: feature_name=SARS.CoV.2.S1.mFcTag	feature_id[0].value > threshold=54010.166015625
node_297: feature_name=a-MoIgG_0.10	feature_id[38].value > threshold=2382.8333740234375
node_303: feature_name=SARS.CoV.2.S1	feature_id[15].value <= threshold=51713.66796875
node_304: feature_name=hCoV.HKU1.S1_AA1.760	feature_id[8].value > threshold=16192.1669921875

node_310: feature_name=a-HuIgM_0.10	feature_id[33].value > threshold=176.5
node_314: feature_name=SARS.CoV.2.S1+S2	feature_id[18].value > threshold=50299.93359375
node_318: feature_name=a-HuIgG_0.30	feature_id[30].value > threshold=47587.833984375
node_336: feature_name=hCoV.HKU1.S1_AA1.760	feature_id[8].value > threshold=45964.3828125
node_342: feature_name=a-HuIgG_0.03	feature_id[1].value <= threshold=13373.1669921875
Class: healthcare workers between 60 and 180 days after vaccination	
Rules_121	passed counts:1
node_0: feature_name=HuIgG_0.03	feature_id[44].value > threshold=3298.333251953125
node_78: feature_name=a-HuIgG_0.03	feature_id[1].value <= threshold=19665.666015625
node_79: feature_name=SARS.CoV.2.S1.mFcTag	feature_id[0].value > threshold=54010.166015625
node_297: feature_name=a-MoIgG_0.10	feature_id[38].value > threshold=2382.8333740234375
node_303: feature_name=SARS.CoV.2.S1	feature_id[15].value <= threshold=51713.66796875
node_304: feature_name=hCoV.HKU1.S1_AA1.760	feature_id[8].value > threshold=16192.1669921875
node_310: feature_name=a-HuIgM_0.10	feature_id[33].value > threshold=176.5
node_314: feature_name=SARS.CoV.2.S1+S2	feature_id[18].value > threshold=50299.93359375
node_318: feature_name=a-HuIgG_0.30	feature_id[30].value > threshold=47587.833984375
node_336: feature_name=hCoV.HKU1.S1_AA1.760	feature_id[8].value <= threshold=45964.3828125
node_337: feature_name=SARS.CoV.2.S1+S2	feature_id[18].value > threshold=50700.5
node_339: feature_name=a-MoIgA_0.30	feature_id[35].value <= threshold=1534.1666259765625
Class: healthcare workers within 60 days after vaccination	
Rules_122	passed counts:1
node_0: feature_name=HuIgG_0.03	feature_id[44].value > threshold=3298.333251953125
node_78: feature_name=a-HuIgG_0.03	feature_id[1].value <= threshold=19665.666015625

node_79: feature_name=SARS.CoV.2.S1.mFcTag	feature_id[0].value > threshold=54010.166015625
node_297: feature_name=a-MoIgG_0.10	feature_id[38].value > threshold=2382.8333740234375
node_303: feature_name=SARS.CoV.2.S1	feature_id[15].value <= threshold=51713.66796875
node_304: feature_name=hCoV.HKU1.S1_AA1.760	feature_id[8].value > threshold=16192.1669921875
node_310: feature_name=a-HuIgM_0.10	feature_id[33].value > threshold=176.5
node_314: feature_name=SARS.CoV.2.S1+S2	feature_id[18].value > threshold=50299.93359375
node_318: feature_name=a-HuIgG_0.30	feature_id[30].value > threshold=47587.833984375
node_336: feature_name=hCoV.HKU1.S1_AA1.760	feature_id[8].value <= threshold=45964.3828125
node_337: feature_name=SARS.CoV.2.S1+S2	feature_id[18].value <= threshold=50700.5
Class: healthcare workers within 60 days after vaccination	
Rules_123	passed counts:1
node_0: feature_name=HuIgG_0.03	feature_id[44].value > threshold=3298.333251953125
node_78: feature_name=a-HuIgG_0.03	feature_id[1].value <= threshold=19665.666015625
node_79: feature_name=SARS.CoV.2.S1.mFcTag	feature_id[0].value > threshold=54010.166015625
node_297: feature_name=a-MoIgG_0.10	feature_id[38].value > threshold=2382.8333740234375
node_303: feature_name=SARS.CoV.2.S1	feature_id[15].value <= threshold=51713.66796875
node_304: feature_name=hCoV.HKU1.S1_AA1.760	feature_id[8].value > threshold=16192.1669921875
node_310: feature_name=a-HuIgM_0.10	feature_id[33].value > threshold=176.5
node_314: feature_name=SARS.CoV.2.S1+S2	feature_id[18].value > threshold=50299.93359375
node_318: feature_name=a-HuIgG_0.30	feature_id[30].value <= threshold=47587.833984375
node_319: feature_name=MERS.CoV.S1.RBD.367.606.rFcTag	feature_id[3].value > threshold=3945.61669921875
node_323: feature_name=a-MoIgG_0.10	feature_id[38].value > threshold=6842.166748046875
node_327: feature_name=SARS.CoV.2.S1.mFcTag	feature_id[0].value > threshold=54403.482421875
node_333: feature_name=Flu.B_Mal/.HA1+HA2	feature_id[25].value > threshold=53690.8828125

Class: healthcare workers between 60 and 180 days after vaccination	
Rules_124	passed counts:1
node_0: feature_name=HuIgG_0.03	feature_id[44].value > threshold=3298.333251953125
node_78: feature_name=a-HuIgG_0.03	feature_id[1].value <= threshold=19665.666015625
node_79: feature_name=SARS.CoV.2.S1.mFcTag	feature_id[0].value > threshold=54010.166015625
node_297: feature_name=a-MoIgG_0.10	feature_id[38].value > threshold=2382.8333740234375
node_303: feature_name=SARS.CoV.2.S1	feature_id[15].value <= threshold=51713.66796875
node_304: feature_name=hCoV.HKU1.S1_AA1.760	feature_id[8].value > threshold=16192.1669921875
node_310: feature_name=a-HuIgM_0.10	feature_id[33].value > threshold=176.5
node_314: feature_name=SARS.CoV.2.S1+S2	feature_id[18].value > threshold=50299.93359375
node_318: feature_name=a-HuIgG_0.30	feature_id[30].value <= threshold=47587.833984375
node_319: feature_name=MERS.CoV.S1.RBD.367.606.rFcTag	feature_id[3].value > threshold=3945.61669921875
node_323: feature_name=a-MoIgG_0.10	feature_id[38].value > threshold=6842.166748046875
node_327: feature_name=SARS.CoV.2.S1.mFcTag	feature_id[0].value <= threshold=54403.482421875
node_328: feature_name=HuIgG_0.03	feature_id[44].value <= threshold=5438.833251953125
node_329: feature_name=SARS.CoV.2.S1.mFcTag	feature_id[0].value > threshold=54295.33203125
Class: unvaccinated healthcare workers	
Rules_125	passed counts:1
node_0: feature_name=HuIgG_0.03	feature_id[44].value > threshold=3298.333251953125
node_78: feature_name=a-HuIgG_0.03	feature_id[1].value <= threshold=19665.666015625
node_79: feature_name=SARS.CoV.2.S1.mFcTag	feature_id[0].value > threshold=54010.166015625
node_297: feature_name=a-MoIgG_0.10	feature_id[38].value > threshold=2382.8333740234375
node_303: feature_name=SARS.CoV.2.S1	feature_id[15].value <= threshold=51713.66796875

node_304: feature_name=hCoV.HKU1.S1_AA1.760	feature_id[8].value > threshold=16192.1669921875
node_310: feature_name=a-HuIgM_0.10	feature_id[33].value > threshold=176.5
node_314: feature_name=SARS.CoV.2.S1+S2	feature_id[18].value > threshold=50299.93359375
node_318: feature_name=a-HuIgG_0.30	feature_id[30].value <= threshold=47587.833984375
node_319: feature_name=MERS.CoV.S1.RBD.367.606.rFcTag	feature_id[3].value > threshold=3945.61669921875
node_323: feature_name=a-MoIgG_0.10	feature_id[38].value > threshold=6842.166748046875
node_327: feature_name=SARS.CoV.2.S1.mFcTag	feature_id[0].value <= threshold=54403.482421875
node_328: feature_name=HuIgG_0.03	feature_id[44].value <= threshold=5438.833251953125
node_329: feature_name=SARS.CoV.2.S1.mFcTag	feature_id[0].value <= threshold=54295.33203125
Class: healthcare workers within 60 days after vaccination	
Rules_126	passed counts:1
node_0: feature_name=HuIgG_0.03	feature_id[44].value > threshold=3298.333251953125
node_78: feature_name=a-HuIgG_0.03	feature_id[1].value <= threshold=19665.666015625
node_79: feature_name=SARS.CoV.2.S1.mFcTag	feature_id[0].value > threshold=54010.166015625
node_297: feature_name=a-MoIgG_0.10	feature_id[38].value > threshold=2382.8333740234375
node_303: feature_name=SARS.CoV.2.S1	feature_id[15].value <= threshold=51713.66796875
node_304: feature_name=hCoV.HKU1.S1_AA1.760	feature_id[8].value > threshold=16192.1669921875
node_310: feature_name=a-HuIgM_0.10	feature_id[33].value > threshold=176.5
node_314: feature_name=SARS.CoV.2.S1+S2	feature_id[18].value > threshold=50299.93359375
node_318: feature_name=a-HuIgG_0.30	feature_id[30].value <= threshold=47587.833984375
node_319: feature_name=MERS.CoV.S1.RBD.367.606.rFcTag	feature_id[3].value > threshold=3945.61669921875
node_323: feature_name=a-MoIgG_0.10	feature_id[38].value <= threshold=6842.166748046875
node_324: feature_name=a-HuIgA_0.10	feature_id[28].value > threshold=2405.8333740234375
Class: healthcare workers within 60 days after vaccination	

Rules_127	passed counts:1
node_0: feature_name=HuIgG_0.03	feature_id[44].value > threshold=3298.333251953125
node_78: feature_name=a-HuIgG_0.03	feature_id[1].value <= threshold=19665.666015625
node_79: feature_name=SARS.CoV.2.S1.mFcTag	feature_id[0].value > threshold=54010.166015625
node_297: feature_name=a-MoIgG_0.10	feature_id[38].value > threshold=2382.8333740234375
node_303: feature_name=SARS.CoV.2.S1	feature_id[15].value <= threshold=51713.66796875
node_304: feature_name=hCoV.HKU1.S1_AA1.760	feature_id[8].value > threshold=16192.1669921875
node_310: feature_name=a-HuIgM_0.10	feature_id[33].value > threshold=176.5
node_314: feature_name=SARS.CoV.2.S1+S2	feature_id[18].value <= threshold=50299.93359375
node_315: feature_name=a-HuIgA_0.03	feature_id[29].value <= threshold=-68.0
Class: healthcare workers within 60 days after vaccination	
Rules_128	passed counts:1
node_0: feature_name=HuIgG_0.03	feature_id[44].value > threshold=3298.333251953125
node_78: feature_name=a-HuIgG_0.03	feature_id[1].value <= threshold=19665.666015625
node_79: feature_name=SARS.CoV.2.S1.mFcTag	feature_id[0].value > threshold=54010.166015625
node_297: feature_name=a-MoIgG_0.10	feature_id[38].value <= threshold=2382.8333740234375
node_298: feature_name=Flu.B_Mal/.HA1	feature_id[4].value <= threshold=41908.115234375
node_299: feature_name=a-HuIgA_0.10	feature_id[28].value <= threshold=164.00000381469727
Class: healthcare workers within 60 days after vaccination	
Rules_129	passed counts:1
node_0: feature_name=HuIgG_0.03	feature_id[44].value > threshold=3298.333251953125
node_78: feature_name=a-HuIgG_0.03	feature_id[1].value <= threshold=19665.666015625

node_79: feature_name=SARS.CoV.2.S1.mFcTag	feature_id[0].value <= threshold=54010.166015625
node_80: feature_name=SARS.CoV.2.S1+S2	feature_id[18].value > threshold=48317.708984375
node_224: feature_name=a-MoIgG_0.10	feature_id[38].value > threshold=2066.8333129882812
node_226: feature_name=HuIgM_0.30	feature_id[13].value > threshold=750.1666870117188
node_238: feature_name=HuIgM_0.30	feature_id[13].value > threshold=760.3333435058594
node_240: feature_name=hCoV.OC43.HE	feature_id[10].value > threshold=3879.75
node_246: feature_name=a-HuIgA_0.30	feature_id[27].value > threshold=1379.8333740234375
node_248: feature_name=Flu.H1N1.HA1	feature_id[26].value <= threshold=53891.083984375
node_249: feature_name=MERS.CoV.S1.RBD.367.606.rFcTag	feature_id[3].value > threshold=1486.7666625976562
node_253: feature_name=HuIgM_0.03	feature_id[45].value > threshold=-56.33333396911621
node_279: feature_name=hCoV.229E.S1	feature_id[2].value > threshold=51743.583984375
Class: healthcare workers between 60 and 180 days after vaccination	
Rules_130	passed counts:1
node_0: feature_name=HuIgG_0.03	feature_id[44].value > threshold=3298.333251953125
node_78: feature_name=a-HuIgG_0.03	feature_id[1].value <= threshold=19665.666015625
node_79: feature_name=SARS.CoV.2.S1.mFcTag	feature_id[0].value <= threshold=54010.166015625
node_80: feature_name=SARS.CoV.2.S1+S2	feature_id[18].value > threshold=48317.708984375
node_224: feature_name=a-MoIgG_0.10	feature_id[38].value > threshold=2066.8333129882812
node_226: feature_name=HuIgM_0.30	feature_id[13].value > threshold=750.1666870117188
node_238: feature_name=HuIgM_0.30	feature_id[13].value > threshold=760.3333435058594
node_240: feature_name=hCoV.OC43.HE	feature_id[10].value > threshold=3879.75
node_246: feature_name=a-HuIgA_0.30	feature_id[27].value > threshold=1379.8333740234375
node_248: feature_name=Flu.H1N1.HA1	feature_id[26].value <= threshold=53891.083984375
node_249: feature_name=MERS.CoV.S1.RBD.367.606.rFcTag	feature_id[3].value > threshold=1486.7666625976562

node_253: feature_name=HuIgM_0.03	feature_id[45].value > threshold=-56.33333396911621
node_279: feature_name=hCoV.229E.S1	feature_id[2].value <= threshold=51743.583984375
node_280: feature_name=Flu.B_Mal/.HA1	feature_id[4].value > threshold=48206.833984375
node_290: feature_name=MERS.CoV.S1.ECD.1-1297.HisTag	feature_id[12].value <= threshold=25489.541015625
node_291: feature_name=a-HuIgM_0.10	feature_id[33].value > threshold=1208.5
Class: healthcare workers over 180 days after vaccination	
Rules_131	passed counts:1
node_0: feature_name=HuIgG_0.03	feature_id[44].value > threshold=3298.333251953125
node_78: feature_name=a-HuIgG_0.03	feature_id[1].value <= threshold=19665.666015625
node_79: feature_name=SARS.CoV.2.S1.mFcTag	feature_id[0].value <= threshold=54010.166015625
node_80: feature_name=SARS.CoV.2.S1+S2	feature_id[18].value > threshold=48317.708984375
node_224: feature_name=a-MoIgG_0.10	feature_id[38].value > threshold=2066.8333129882812
node_226: feature_name=HuIgM_0.30	feature_id[13].value > threshold=750.1666870117188
node_238: feature_name=HuIgM_0.30	feature_id[13].value > threshold=760.3333435058594
node_240: feature_name=hCoV.OC43.HE	feature_id[10].value > threshold=3879.75
node_246: feature_name=a-HuIgA_0.30	feature_id[27].value > threshold=1379.8333740234375
node_248: feature_name=Flu.H1N1.HA1	feature_id[26].value <= threshold=53891.083984375
node_249: feature_name=MERS.CoV.S1.RBD.367.606.rFcTag	feature_id[3].value > threshold=1486.7666625976562
node_253: feature_name=HuIgM_0.03	feature_id[45].value > threshold=-56.33333396911621
node_279: feature_name=hCoV.229E.S1	feature_id[2].value <= threshold=51743.583984375
node_280: feature_name=Flu.B_Mal/.HA1	feature_id[4].value > threshold=48206.833984375
node_290: feature_name=MERS.CoV.S1.ECD.1-1297.HisTag	feature_id[12].value <= threshold=25489.541015625
node_291: feature_name=a-HuIgM_0.10	feature_id[33].value <= threshold=1208.5
Class: healthcare workers between 60 and 180 days after vaccination	

Rules_132	passed counts:1
node_0: feature_name=HuIgG_0.03	feature_id[44].value > threshold=3298.333251953125
node_78: feature_name=a-HuIgG_0.03	feature_id[1].value <= threshold=19665.666015625
node_79: feature_name=SARS.CoV.2.S1.mFcTag	feature_id[0].value <= threshold=54010.166015625
node_80: feature_name=SARS.CoV.2.S1+S2	feature_id[18].value > threshold=48317.708984375
node_224: feature_name=a-MoIgG_0.10	feature_id[38].value > threshold=2066.8333129882812
node_226: feature_name=HuIgM_0.30	feature_id[13].value > threshold=750.1666870117188
node_238: feature_name=HuIgM_0.30	feature_id[13].value > threshold=760.3333435058594
node_240: feature_name=hCoV.OC43.HE	feature_id[10].value > threshold=3879.75
node_246: feature_name=a-HuIgA_0.30	feature_id[27].value > threshold=1379.8333740234375
node_248: feature_name=Flu.H1N1.HA1	feature_id[26].value <= threshold=53891.083984375
node_249: feature_name=MERS.CoV.S1.RBD.367.606.rFcTag	feature_id[3].value > threshold=1486.7666625976562
node_253: feature_name=HuIgM_0.03	feature_id[45].value > threshold=-56.33333396911621
node_279: feature_name=hCoV.229E.S1	feature_id[2].value <= threshold=51743.583984375
node_280: feature_name=Flu.B_Mal/.HA1	feature_id[4].value <= threshold=48206.833984375
node_281: feature_name=a-MoIgM_0.10	feature_id[40].value > threshold=989.5000305175781
node_285: feature_name=HuIgM_0.30	feature_id[13].value > threshold=2008.3333129882812
node_287: feature_name=SARS.CoV.2.S1.RBD.mFc	feature_id[17].value <= threshold=43325.44921875
Class: healthcare workers between 60 and 180 days after vaccination	
Rules_133	passed counts:1
node_0: feature_name=HuIgG_0.03	feature_id[44].value > threshold=3298.333251953125
node_78: feature_name=a-HuIgG_0.03	feature_id[1].value <= threshold=19665.666015625
node_79: feature_name=SARS.CoV.2.S1.mFcTag	feature_id[0].value <= threshold=54010.166015625

node_80: feature_name=SARS.CoV.2.S1+S2	feature_id[18].value > threshold=48317.708984375
node_224: feature_name=a-MoIgG_0.10	feature_id[38].value > threshold=2066.8333129882812
node_226: feature_name=HuIgM_0.30	feature_id[13].value > threshold=750.1666870117188
node_238: feature_name=HuIgM_0.30	feature_id[13].value > threshold=760.3333435058594
node_240: feature_name=hCoV.OC43.HE	feature_id[10].value > threshold=3879.75
node_246: feature_name=a-HuIgA_0.30	feature_id[27].value > threshold=1379.8333740234375
node_248: feature_name=Flu.H1N1.HA1	feature_id[26].value <= threshold=53891.083984375
node_249: feature_name=MERS.CoV.S1.RBD.367.606.rFcTag	feature_id[3].value > threshold=1486.7666625976562
node_253: feature_name=HuIgM_0.03	feature_id[45].value > threshold=-56.33333396911621
node_279: feature_name=hCoV.229E.S1	feature_id[2].value <= threshold=51743.583984375
node_280: feature_name=Flu.B_Mal/.HA1	feature_id[4].value <= threshold=48206.833984375
node_281: feature_name=a-MoIgM_0.10	feature_id[40].value <= threshold=989.5000305175781
node_282: feature_name=SARS.CoV.S1.HisTag	feature_id[14].value > threshold=22347.5166015625
Class: healthcare workers within 60 days after vaccination	
Rules_134	passed counts:1
node_0: feature_name=HuIgG_0.03	feature_id[44].value > threshold=3298.333251953125
node_78: feature_name=a-HuIgG_0.03	feature_id[1].value <= threshold=19665.666015625
node_79: feature_name=SARS.CoV.2.S1.mFcTag	feature_id[0].value <= threshold=54010.166015625
node_80: feature_name=SARS.CoV.2.S1+S2	feature_id[18].value > threshold=48317.708984375
node_224: feature_name=a-MoIgG_0.10	feature_id[38].value > threshold=2066.8333129882812
node_226: feature_name=HuIgM_0.30	feature_id[13].value > threshold=750.1666870117188
node_238: feature_name=HuIgM_0.30	feature_id[13].value > threshold=760.3333435058594
node_240: feature_name=hCoV.OC43.HE	feature_id[10].value > threshold=3879.75
node_246: feature_name=a-HuIgA_0.30	feature_id[27].value > threshold=1379.8333740234375

node_248: feature_name=Flu.H1N1.HA1	feature_id[26].value <= threshold=53891.083984375
node_249: feature_name=MERS.CoV.S1.RBD.367.606.rFcTag	feature_id[3].value > threshold=1486.7666625976562
node_253: feature_name=HuIgM_0.03	feature_id[45].value > threshold=-56.33333396911621
node_279: feature_name=hCoV.229E.S1	feature_id[2].value <= threshold=51743.583984375
node_280: feature_name=Flu.B_Mal/.HA1	feature_id[4].value <= threshold=48206.833984375
node_281: feature_name=a-MoIgM_0.10	feature_id[40].value <= threshold=989.5000305175781
node_282: feature_name=SARS.CoV.S1.HisTag	feature_id[14].value <= threshold=22347.5166015625
Class: healthcare workers between 60 and 180 days after vaccination	
Rules_135	passed counts:1
node_0: feature_name=HuIgG_0.03	feature_id[44].value > threshold=3298.333251953125
node_78: feature_name=a-HuIgG_0.03	feature_id[1].value <= threshold=19665.666015625
node_79: feature_name=SARS.CoV.2.S1.mFcTag	feature_id[0].value <= threshold=54010.166015625
node_80: feature_name=SARS.CoV.2.S1+S2	feature_id[18].value > threshold=48317.708984375
node_224: feature_name=a-MoIgG_0.10	feature_id[38].value > threshold=2066.8333129882812
node_226: feature_name=HuIgM_0.30	feature_id[13].value > threshold=750.1666870117188
node_238: feature_name=HuIgM_0.30	feature_id[13].value > threshold=760.3333435058594
node_240: feature_name=hCoV.OC43.HE	feature_id[10].value > threshold=3879.75
node_246: feature_name=a-HuIgA_0.30	feature_id[27].value > threshold=1379.8333740234375
node_248: feature_name=Flu.H1N1.HA1	feature_id[26].value <= threshold=53891.083984375
node_249: feature_name=MERS.CoV.S1.RBD.367.606.rFcTag	feature_id[3].value > threshold=1486.7666625976562
node_253: feature_name=HuIgM_0.03	feature_id[45].value <= threshold=-56.33333396911621
node_254: feature_name=MoIgA_0.30	feature_id[46].value > threshold=900.1666564941406
node_258: feature_name=SARS.CoV.2.S1.mFcTag	feature_id[0].value > threshold=53346.333984375
node_272: feature_name=a-MoIgA_0.03	feature_id[37].value > threshold=2.3333334028720856

node_274: feature_name=Flu.B_Mal/.HA1+HA2	feature_id[25].value <= threshold=53981.70703125
node_275: feature_name=a-MoIgG_0.10	feature_id[38].value > threshold=22321.6669921875
Class: healthcare workers within 60 days after vaccination	
Rules_136	passed counts:1
node_0: feature_name=HuIgG_0.03	feature_id[44].value > threshold=3298.333251953125
node_78: feature_name=a-HuIgG_0.03	feature_id[1].value <= threshold=19665.666015625
node_79: feature_name=SARS.CoV.2.S1.mFcTag	feature_id[0].value <= threshold=54010.166015625
node_80: feature_name=SARS.CoV.2.S1+S2	feature_id[18].value > threshold=48317.708984375
node_224: feature_name=a-MoIgG_0.10	feature_id[38].value > threshold=2066.8333129882812
node_226: feature_name=HuIgM_0.30	feature_id[13].value > threshold=750.1666870117188
node_238: feature_name=HuIgM_0.30	feature_id[13].value > threshold=760.3333435058594
node_240: feature_name=hCoV.OC43.HE	feature_id[10].value > threshold=3879.75
node_246: feature_name=a-HuIgA_0.30	feature_id[27].value > threshold=1379.8333740234375
node_248: feature_name=Flu.H1N1.HA1	feature_id[26].value <= threshold=53891.083984375
node_249: feature_name=MERS.CoV.S1.RBD.367.606.rFcTag	feature_id[3].value > threshold=1486.7666625976562
node_253: feature_name=HuIgM_0.03	feature_id[45].value <= threshold=-56.33333396911621
node_254: feature_name=MoIgA_0.30	feature_id[46].value > threshold=900.1666564941406
node_258: feature_name=SARS.CoV.2.S1.mFcTag	feature_id[0].value <= threshold=53346.333984375
node_259: feature_name=SARS.CoV.2.S1	feature_id[15].value <= threshold=47301.333984375
node_260: feature_name=a-MoIgM_0.03	feature_id[41].value > threshold=127.16666793823242
node_262: feature_name=hCoV.OC43.HE	feature_id[10].value > threshold=12272.5166015625
node_268: feature_name=HuIgM_0.03	feature_id[45].value > threshold=-57.16666603088379
Class: healthcare workers between 60 and 180 days after vaccination	

Rules_137	passed counts:1
node_0: feature_name=HuIgG_0.03	feature_id[44].value > threshold=3298.333251953125
node_78: feature_name=a-HuIgG_0.03	feature_id[1].value <= threshold=19665.666015625
node_79: feature_name=SARS.CoV.2.S1.mFcTag	feature_id[0].value <= threshold=54010.166015625
node_80: feature_name=SARS.CoV.2.S1+S2	feature_id[18].value > threshold=48317.708984375
node_224: feature_name=a-MoIgG_0.10	feature_id[38].value > threshold=2066.8333129882812
node_226: feature_name=HuIgM_0.30	feature_id[13].value > threshold=750.1666870117188
node_238: feature_name=HuIgM_0.30	feature_id[13].value > threshold=760.3333435058594
node_240: feature_name=hCoV.OC43.HE	feature_id[10].value > threshold=3879.75
node_246: feature_name=a-HuIgA_0.30	feature_id[27].value > threshold=1379.8333740234375
node_248: feature_name=Flu.H1N1.HA1	feature_id[26].value <= threshold=53891.083984375
node_249: feature_name=MERS.CoV.S1.RBD.367.606.rFcTag	feature_id[3].value > threshold=1486.7666625976562
node_253: feature_name=HuIgM_0.03	feature_id[45].value <= threshold=-56.33333396911621
node_254: feature_name=MoIgA_0.30	feature_id[46].value > threshold=900.1666564941406
node_258: feature_name=SARS.CoV.2.S1.mFcTag	feature_id[0].value <= threshold=53346.333984375
node_259: feature_name=SARS.CoV.2.S1	feature_id[15].value <= threshold=47301.333984375
node_260: feature_name=a-MoIgM_0.03	feature_id[41].value > threshold=127.16666793823242
node_262: feature_name=hCoV.OC43.HE	feature_id[10].value <= threshold=12272.5166015625
node_263: feature_name=SARS.CoV.2.S1+S2	feature_id[18].value > threshold=49907.841796875
node_265: feature_name=a-MoIgG_0.10	feature_id[38].value > threshold=23887.5
Class: healthcare workers within 60 days after vaccination	
Rules_138	passed counts:1
node_0: feature_name=HuIgG_0.03	feature_id[44].value > threshold=3298.333251953125
node_78: feature_name=a-HuIgG_0.03	feature_id[1].value <= threshold=19665.666015625

node_79: feature_name=SARS.CoV.2.S1.mFcTag	feature_id[0].value <= threshold=54010.166015625
node_80: feature_name=SARS.CoV.2.S1+S2	feature_id[18].value > threshold=48317.708984375
node_224: feature_name=a-MoIgG_0.10	feature_id[38].value > threshold=2066.8333129882812
node_226: feature_name=HuIgM_0.30	feature_id[13].value > threshold=750.1666870117188
node_238: feature_name=HuIgM_0.30	feature_id[13].value > threshold=760.3333435058594
node_240: feature_name=hCoV.OC43.HE	feature_id[10].value > threshold=3879.75
node_246: feature_name=a-HuIgA_0.30	feature_id[27].value > threshold=1379.8333740234375
node_248: feature_name=Flu.H1N1.HA1	feature_id[26].value <= threshold=53891.083984375
node_249: feature_name=MERS.CoV.S1.RBD.367.606.rFcTag	feature_id[3].value > threshold=1486.7666625976562
node_253: feature_name=HuIgM_0.03	feature_id[45].value <= threshold=-56.33333396911621
node_254: feature_name=MoIgA_0.30	feature_id[46].value <= threshold=900.1666564941406
node_255: feature_name=SARS.CoV.2.S1.RBD.mFc	feature_id[17].value > threshold=51784.3828125
Class: healthcare workers within 60 days after vaccination	
Rules_139	passed counts:1
node_0: feature_name=HuIgG_0.03	feature_id[44].value > threshold=3298.333251953125
node_78: feature_name=a-HuIgG_0.03	feature_id[1].value <= threshold=19665.666015625
node_79: feature_name=SARS.CoV.2.S1.mFcTag	feature_id[0].value <= threshold=54010.166015625
node_80: feature_name=SARS.CoV.2.S1+S2	feature_id[18].value > threshold=48317.708984375
node_224: feature_name=a-MoIgG_0.10	feature_id[38].value > threshold=2066.8333129882812
node_226: feature_name=HuIgM_0.30	feature_id[13].value > threshold=750.1666870117188
node_238: feature_name=HuIgM_0.30	feature_id[13].value > threshold=760.3333435058594
node_240: feature_name=hCoV.OC43.HE	feature_id[10].value > threshold=3879.75
node_246: feature_name=a-HuIgA_0.30	feature_id[27].value > threshold=1379.8333740234375
node_248: feature_name=Flu.H1N1.HA1	feature_id[26].value <= threshold=53891.083984375

node_249: feature_name=MERS.CoV.S1.RBD.367.606.rFcTag	feature_id[3].value <= threshold=1486.7666625976562
node_250: feature_name=a-MoIgM_0.10	feature_id[40].value <= threshold=1601.8333129882812
Class: healthcare workers within 60 days after vaccination	
Rules_140	passed counts:1
node_0: feature_name=HuIgG_0.03	feature_id[44].value > threshold=3298.333251953125
node_78: feature_name=a-HuIgG_0.03	feature_id[1].value <= threshold=19665.666015625
node_79: feature_name=SARS.CoV.2.S1.mFcTag	feature_id[0].value <= threshold=54010.166015625
node_80: feature_name=SARS.CoV.2.S1+S2	feature_id[18].value > threshold=48317.708984375
node_224: feature_name=a-MoIgG_0.10	feature_id[38].value > threshold=2066.8333129882812
node_226: feature_name=HuIgM_0.30	feature_id[13].value <= threshold=750.1666870117188
node_227: feature_name=SARS.CoV.S1.RBD.HisTag	feature_id[20].value > threshold=7585.25
node_233: feature_name=SARS.CoV.2.S1.HisTag	feature_id[16].value > threshold=29169.625
node_235: feature_name=a-MoIgA_0.03	feature_id[37].value <= threshold=-137.16666412353516
Class: healthcare workers between 60 and 180 days after vaccination	
Rules_141	passed counts:1
node_0: feature_name=HuIgG_0.03	feature_id[44].value > threshold=3298.333251953125
node_78: feature_name=a-HuIgG_0.03	feature_id[1].value <= threshold=19665.666015625
node_79: feature_name=SARS.CoV.2.S1.mFcTag	feature_id[0].value <= threshold=54010.166015625
node_80: feature_name=SARS.CoV.2.S1+S2	feature_id[18].value > threshold=48317.708984375
node_224: feature_name=a-MoIgG_0.10	feature_id[38].value > threshold=2066.8333129882812
node_226: feature_name=HuIgM_0.30	feature_id[13].value <= threshold=750.1666870117188
node_227: feature_name=SARS.CoV.S1.RBD.HisTag	feature_id[20].value > threshold=7585.25
node_233: feature_name=SARS.CoV.2.S1.HisTag	feature_id[16].value <= threshold=29169.625

Class: healthcare workers between 60 and 180 days after vaccination	
Rules_142	passed counts:1
node_0: feature_name=HuIgG_0.03	feature_id[44].value > threshold=3298.333251953125
node_78: feature_name=a-HuIgG_0.03	feature_id[1].value <= threshold=19665.666015625
node_79: feature_name=SARS.CoV.2.S1.mFcTag	feature_id[0].value <= threshold=54010.166015625
node_80: feature_name=SARS.CoV.2.S1+S2	feature_id[18].value > threshold=48317.708984375
node_224: feature_name=a-MoIgG_0.10	feature_id[38].value > threshold=2066.8333129882812
node_226: feature_name=HuIgM_0.30	feature_id[13].value <= threshold=750.1666870117188
node_227: feature_name=SARS.CoV.S1.RBD.HisTag	feature_id[20].value <= threshold=7585.25
node_228: feature_name=MERS.CoV.S1.ECD.1-1297.HisTag	feature_id[12].value > threshold=22314.0
node_230: feature_name=SARS.CoV.2.Spike.RBD.rFc	feature_id[7].value <= threshold=25894.986328125
Class: unvaccinated healthcare workers	
Rules_143	passed counts:1
node_0: feature_name=HuIgG_0.03	feature_id[44].value > threshold=3298.333251953125
node_78: feature_name=a-HuIgG_0.03	feature_id[1].value <= threshold=19665.666015625
node_79: feature_name=SARS.CoV.2.S1.mFcTag	feature_id[0].value <= threshold=54010.166015625
node_80: feature_name=SARS.CoV.2.S1+S2	feature_id[18].value <= threshold=48317.708984375
node_81: feature_name=SARS.CoV.2.Spike.RBD.His.Bac	feature_id[9].value > threshold=16347.4580078125
node_111: feature_name=hCoV.NL63.S1	feature_id[5].value > threshold=4866.533447265625
node_123: feature_name=MERS.CoV.S1.RBD.367.606.rFcTag	feature_id[3].value > threshold=2789.3416748046875
node_161: feature_name=SARS.CoV.S1.HisTag	feature_id[14].value > threshold=4926.39990234375
node_179: feature_name=hCoV.HKU1.S1_S2	feature_id[22].value <= threshold=48084.783203125
node_180: feature_name=SARS.CoV.2.Spike.RBD.His.Bac	feature_id[9].value > threshold=31607.125

node_200: feature_name=SARS.CoV.2.S1.HisTag	feature_id[16].value <= threshold=43902.201171875
node_201: feature_name=Flu.H1N1.HA1+HA2	feature_id[6].value > threshold=30743.5
node_205: feature_name=Flu.B_Phu/.HA1	feature_id[11].value > threshold=18442.14111328125
node_207: feature_name=SARS.CoV.2.Spike.RBD.rFc	feature_id[7].value > threshold=39119.033203125
node_209: feature_name=MERS.CoV.S1.ECD.1-1297.HisTag	feature_id[12].value <= threshold=11020.70849609375
node_210: feature_name=hCoV.OC43.S1_S2ECD.HisTag	feature_id[24].value > threshold=37992.375
Class: healthcare workers within 60 days after vaccination	
Rules_144	passed counts:1
node_0: feature_name=HuIgG_0.03	feature_id[44].value > threshold=3298.333251953125
node_78: feature_name=a-HuIgG_0.03	feature_id[1].value <= threshold=19665.666015625
node_79: feature_name=SARS.CoV.2.S1.mFcTag	feature_id[0].value <= threshold=54010.166015625
node_80: feature_name=SARS.CoV.2.S1+S2	feature_id[18].value <= threshold=48317.708984375
node_81: feature_name=SARS.CoV.2.Spike.RBD.His.Bac	feature_id[9].value > threshold=16347.4580078125
node_111: feature_name=hCoV.NL63.S1	feature_id[5].value > threshold=4866.533447265625
node_123: feature_name=MERS.CoV.S1.RBD.367.606.rFcTag	feature_id[3].value > threshold=2789.3416748046875
node_161: feature_name=SARS.CoV.S1.HisTag	feature_id[14].value > threshold=4926.39990234375
node_179: feature_name=hCoV.HKU1.S1_S2	feature_id[22].value <= threshold=48084.783203125
node_180: feature_name=SARS.CoV.2.Spike.RBD.His.Bac	feature_id[9].value > threshold=31607.125
node_200: feature_name=SARS.CoV.2.S1.HisTag	feature_id[16].value <= threshold=43902.201171875
node_201: feature_name=Flu.H1N1.HA1+HA2	feature_id[6].value > threshold=30743.5
node_205: feature_name=Flu.B_Phu/.HA1	feature_id[11].value > threshold=18442.14111328125
node_207: feature_name=SARS.CoV.2.Spike.RBD.rFc	feature_id[7].value > threshold=39119.033203125
node_209: feature_name=MERS.CoV.S1.ECD.1-1297.HisTag	feature_id[12].value <= threshold=11020.70849609375
node_210: feature_name=hCoV.OC43.S1_S2ECD.HisTag	feature_id[24].value <= threshold=37992.375

Class: healthcare workers between 60 and 180 days after vaccination	
Rules_145	passed counts:1
node_0: feature_name=HuIgG_0.03	feature_id[44].value > threshold=3298.333251953125
node_78: feature_name=a-HuIgG_0.03	feature_id[1].value <= threshold=19665.666015625
node_79: feature_name=SARS.CoV.2.S1.mFcTag	feature_id[0].value <= threshold=54010.166015625
node_80: feature_name=SARS.CoV.2.S1+S2	feature_id[18].value <= threshold=48317.708984375
node_81: feature_name=SARS.CoV.2.Spike.RBD.His.Bac	feature_id[9].value > threshold=16347.4580078125
node_111: feature_name=hCoV.NL63.S1	feature_id[5].value > threshold=4866.533447265625
node_123: feature_name=MERS.CoV.S1.RBD.367.606.rFcTag	feature_id[3].value > threshold=2789.3416748046875
node_161: feature_name=SARS.CoV.S1.HisTag	feature_id[14].value > threshold=4926.39990234375
node_179: feature_name=hCoV.HKU1.S1_S2	feature_id[22].value <= threshold=48084.783203125
node_180: feature_name=SARS.CoV.2.Spike.RBD.His.Bac	feature_id[9].value > threshold=31607.125
node_200: feature_name=SARS.CoV.2.S1.HisTag	feature_id[16].value <= threshold=43902.201171875
node_201: feature_name=Flu.H1N1.HA1+HA2	feature_id[6].value > threshold=30743.5
node_205: feature_name=Flu.B_Phu/.HA1	feature_id[11].value > threshold=18442.14111328125
node_207: feature_name=SARS.CoV.2.Spike.RBD.rFc	feature_id[7].value <= threshold=39119.033203125
Class: healthcare workers within 60 days after vaccination	
Rules_146	passed counts:1
node_0: feature_name=HuIgG_0.03	feature_id[44].value > threshold=3298.333251953125
node_78: feature_name=a-HuIgG_0.03	feature_id[1].value <= threshold=19665.666015625
node_79: feature_name=SARS.CoV.2.S1.mFcTag	feature_id[0].value <= threshold=54010.166015625
node_80: feature_name=SARS.CoV.2.S1+S2	feature_id[18].value <= threshold=48317.708984375
node_81: feature_name=SARS.CoV.2.Spike.RBD.His.Bac	feature_id[9].value > threshold=16347.4580078125

node_111: feature_name=hCoV.NL63.S1	feature_id[5].value > threshold=4866.533447265625
node_123: feature_name=MERS.CoV.S1.RBD.367.606.rFcTag	feature_id[3].value > threshold=2789.3416748046875
node_161: feature_name=SARS.CoV.S1.HisTag	feature_id[14].value > threshold=4926.39990234375
node_179: feature_name=hCoV.HKU1.S1_S2	feature_id[22].value <= threshold=48084.783203125
node_180: feature_name=SARS.CoV.2.Spike.RBD.His.Bac	feature_id[9].value > threshold=31607.125
node_200: feature_name=SARS.CoV.2.S1.HisTag	feature_id[16].value <= threshold=43902.201171875
node_201: feature_name=Flu.H1N1.HA1+HA2	feature_id[6].value > threshold=30743.5
node_205: feature_name=Flu.B_Phu/.HA1	feature_id[11].value <= threshold=18442.14111328125
Class: healthcare workers over 180 days after vaccination	
Rules_147	passed counts:1
node_0: feature_name=HuIgG_0.03	feature_id[44].value > threshold=3298.333251953125
node_78: feature_name=a-HuIgG_0.03	feature_id[1].value <= threshold=19665.666015625
node_79: feature_name=SARS.CoV.2.S1.mFcTag	feature_id[0].value <= threshold=54010.166015625
node_80: feature_name=SARS.CoV.2.S1+S2	feature_id[18].value <= threshold=48317.708984375
node_81: feature_name=SARS.CoV.2.Spike.RBD.His.Bac	feature_id[9].value > threshold=16347.4580078125
node_111: feature_name=hCoV.NL63.S1	feature_id[5].value > threshold=4866.533447265625
node_123: feature_name=MERS.CoV.S1.RBD.367.606.rFcTag	feature_id[3].value > threshold=2789.3416748046875
node_161: feature_name=SARS.CoV.S1.HisTag	feature_id[14].value > threshold=4926.39990234375
node_179: feature_name=hCoV.HKU1.S1_S2	feature_id[22].value <= threshold=48084.783203125
node_180: feature_name=SARS.CoV.2.Spike.RBD.His.Bac	feature_id[9].value > threshold=31607.125
node_200: feature_name=SARS.CoV.2.S1.HisTag	feature_id[16].value <= threshold=43902.201171875
node_201: feature_name=Flu.H1N1.HA1+HA2	feature_id[6].value <= threshold=30743.5
node_202: feature_name=a-HuIgG_0.30	feature_id[30].value <= threshold=42035.166015625
Class: healthcare workers between 60 and 180 days after vaccination	

Rules_148	passed counts:1
node_0: feature_name=HuIgG_0.03	feature_id[44].value > threshold=3298.333251953125
node_78: feature_name=a-HuIgG_0.03	feature_id[1].value <= threshold=19665.666015625
node_79: feature_name=SARS.CoV.2.S1.mFcTag	feature_id[0].value <= threshold=54010.166015625
node_80: feature_name=SARS.CoV.2.S1+S2	feature_id[18].value <= threshold=48317.708984375
node_81: feature_name=SARS.CoV.2.Spike.RBD.His.Bac	feature_id[9].value > threshold=16347.4580078125
node_111: feature_name=hCoV.NL63.S1	feature_id[5].value > threshold=4866.533447265625
node_123: feature_name=MERS.CoV.S1.RBD.367.606.rFcTag	feature_id[3].value > threshold=2789.3416748046875
node_161: feature_name=SARS.CoV.S1.HisTag	feature_id[14].value > threshold=4926.39990234375
node_179: feature_name=hCoV.HKU1.S1_S2	feature_id[22].value <= threshold=48084.783203125
node_180: feature_name=SARS.CoV.2.Spike.RBD.His.Bac	feature_id[9].value <= threshold=31607.125
node_181: feature_name=SARS.CoV.2.S1	feature_id[15].value > threshold=33718.333984375
node_191: feature_name=a-HuIgA_0.30	feature_id[27].value > threshold=1819.5
node_193: feature_name=HuIgM_0.30	feature_id[13].value <= threshold=1081.8333740234375
node_194: feature_name=hCoV.NL63.S1	feature_id[5].value <= threshold=6020.75830078125
Class: healthcare workers over 180 days after vaccination	
Rules_149	passed counts:1
node_0: feature_name=HuIgG_0.03	feature_id[44].value > threshold=3298.333251953125
node_78: feature_name=a-HuIgG_0.03	feature_id[1].value <= threshold=19665.666015625
node_79: feature_name=SARS.CoV.2.S1.mFcTag	feature_id[0].value <= threshold=54010.166015625
node_80: feature_name=SARS.CoV.2.S1+S2	feature_id[18].value <= threshold=48317.708984375
node_81: feature_name=SARS.CoV.2.Spike.RBD.His.Bac	feature_id[9].value > threshold=16347.4580078125
node_111: feature_name=hCoV.NL63.S1	feature_id[5].value > threshold=4866.533447265625

node_123: feature_name=MERS.CoV.S1.RBD.367.606.rFcTag	feature_id[3].value > threshold=2789.3416748046875
node_161: feature_name=SARS.CoV.S1.HisTag	feature_id[14].value > threshold=4926.39990234375
node_179: feature_name=hCoV.HKU1.S1_S2	feature_id[22].value <= threshold=48084.783203125
node_180: feature_name=SARS.CoV.2.Spike.RBD.His.Bac	feature_id[9].value <= threshold=31607.125
node_181: feature_name=SARS.CoV.2.S1	feature_id[15].value <= threshold=33718.333984375
node_182: feature_name=a-HuIgG_0.03	feature_id[1].value <= threshold=15402.5
node_183: feature_name=a-HuIgM_0.30	feature_id[32].value > threshold=1969.0000610351562
node_185: feature_name=SARS.CoV.NP	feature_id[19].value <= threshold=1277.2083435058594
Class: healthcare workers within 60 days after vaccination	
Rules_150	passed counts:1
node_0: feature_name=HuIgG_0.03	feature_id[44].value > threshold=3298.333251953125
node_78: feature_name=a-HuIgG_0.03	feature_id[1].value <= threshold=19665.666015625
node_79: feature_name=SARS.CoV.2.S1.mFcTag	feature_id[0].value <= threshold=54010.166015625
node_80: feature_name=SARS.CoV.2.S1+S2	feature_id[18].value <= threshold=48317.708984375
node_81: feature_name=SARS.CoV.2.Spike.RBD.His.Bac	feature_id[9].value > threshold=16347.4580078125
node_111: feature_name=hCoV.NL63.S1	feature_id[5].value > threshold=4866.533447265625
node_123: feature_name=MERS.CoV.S1.RBD.367.606.rFcTag	feature_id[3].value > threshold=2789.3416748046875
node_161: feature_name=SARS.CoV.S1.HisTag	feature_id[14].value <= threshold=4926.39990234375
node_162: feature_name=a-MoIgA_0.10	feature_id[36].value <= threshold=3108.3333740234375
node_163: feature_name=Flu.B_Mal/.HA1+HA2	feature_id[25].value > threshold=38684.7265625
node_167: feature_name=SARS.CoV.NP	feature_id[19].value > threshold=18293.6826171875
node_175: feature_name=Flu.B_Phu/.HA1	feature_id[11].value <= threshold=37557.841796875
Class: healthcare workers between 60 and 180 days after vaccination	

Rules_151	passed counts:1
node_0: feature_name=HuIgG_0.03	feature_id[44].value > threshold=3298.333251953125
node_78: feature_name=a-HuIgG_0.03	feature_id[1].value <= threshold=19665.666015625
node_79: feature_name=SARS.CoV.2.S1.mFcTag	feature_id[0].value <= threshold=54010.166015625
node_80: feature_name=SARS.CoV.2.S1+S2	feature_id[18].value <= threshold=48317.708984375
node_81: feature_name=SARS.CoV.2.Spike.RBD.His.Bac	feature_id[9].value > threshold=16347.4580078125
node_111: feature_name=hCoV.NL63.S1	feature_id[5].value > threshold=4866.533447265625
node_123: feature_name=MERS.CoV.S1.RBD.367.606.rFcTag	feature_id[3].value > threshold=2789.3416748046875
node_161: feature_name=SARS.CoV.S1.HisTag	feature_id[14].value <= threshold=4926.39990234375
node_162: feature_name=a-MoIgA_0.10	feature_id[36].value <= threshold=3108.3333740234375
node_163: feature_name=Flu.B_Mal/.HA1+HA2	feature_id[25].value > threshold=38684.7265625
node_167: feature_name=SARS.CoV.NP	feature_id[19].value <= threshold=18293.6826171875
node_168: feature_name=a-HuIgG_0.03	feature_id[1].value > threshold=10603.0
node_172: feature_name=a-HuIgM_0.30	feature_id[32].value > threshold=29969.8330078125
Class: healthcare workers within 60 days after vaccination	
Rules_152	passed counts:1
node_0: feature_name=HuIgG_0.03	feature_id[44].value > threshold=3298.333251953125
node_78: feature_name=a-HuIgG_0.03	feature_id[1].value <= threshold=19665.666015625
node_79: feature_name=SARS.CoV.2.S1.mFcTag	feature_id[0].value <= threshold=54010.166015625
node_80: feature_name=SARS.CoV.2.S1+S2	feature_id[18].value <= threshold=48317.708984375
node_81: feature_name=SARS.CoV.2.Spike.RBD.His.Bac	feature_id[9].value > threshold=16347.4580078125
node_111: feature_name=hCoV.NL63.S1	feature_id[5].value > threshold=4866.533447265625
node_123: feature_name=MERS.CoV.S1.RBD.367.606.rFcTag	feature_id[3].value > threshold=2789.3416748046875
node_161: feature_name=SARS.CoV.S1.HisTag	feature_id[14].value <= threshold=4926.39990234375

node_162: feature_name=a-MoIgA_0.10	feature_id[36].value <= threshold=3108.3333740234375
node_163: feature_name=Flu.B_Mal/.HA1+HA2	feature_id[25].value <= threshold=38684.7265625
node_164: feature_name=a-MoIgA_0.30	feature_id[35].value > threshold=7878.1668701171875
Class: healthcare workers between 60 and 180 days after vaccination	
Rules_153	passed counts:1
node_0: feature_name=HuIgG_0.03	feature_id[44].value > threshold=3298.333251953125
node_78: feature_name=a-HuIgG_0.03	feature_id[1].value <= threshold=19665.666015625
node_79: feature_name=SARS.CoV.2.S1.mFcTag	feature_id[0].value <= threshold=54010.166015625
node_80: feature_name=SARS.CoV.2.S1+S2	feature_id[18].value <= threshold=48317.708984375
node_81: feature_name=SARS.CoV.2.Spike.RBD.His.Bac	feature_id[9].value > threshold=16347.4580078125
node_111: feature_name=hCoV.NL63.S1	feature_id[5].value > threshold=4866.533447265625
node_123: feature_name=MERS.CoV.S1.RBD.367.606.rFcTag	feature_id[3].value <= threshold=2789.3416748046875
node_124: feature_name=SARS.CoV.2.S1+S2	feature_id[18].value > threshold=41798.25
node_140: feature_name=SARS.CoV.2.S1.RBD.mFc	feature_id[17].value <= threshold=44491.166015625
node_141: feature_name=a-HuIgM_0.03	feature_id[34].value > threshold=-6.833333492279053
node_149: feature_name=a-MoIgA_0.30	feature_id[35].value > threshold=935.3333435058594
node_151: feature_name=HuIgM_0.30	feature_id[13].value <= threshold=1110.5
node_152: feature_name=a-HuIgG_0.30	feature_id[30].value > threshold=46032.0
node_154: feature_name=a-MoIgM_0.03	feature_id[41].value > threshold=1308.5
Class: healthcare workers within 60 days after vaccination	
Rules_154	passed counts:1
node_0: feature_name=HuIgG_0.03	feature_id[44].value > threshold=3298.333251953125
node_78: feature_name=a-HuIgG_0.03	feature_id[1].value <= threshold=19665.666015625

node_79: feature_name=SARS.CoV.2.S1.mFcTag	feature_id[0].value <= threshold=54010.166015625
node_80: feature_name=SARS.CoV.2.S1+S2	feature_id[18].value <= threshold=48317.708984375
node_81: feature_name=SARS.CoV.2.Spike.RBD.His.Bac	feature_id[9].value > threshold=16347.4580078125
node_111: feature_name=hCoV.NL63.S1	feature_id[5].value > threshold=4866.533447265625
node_123: feature_name=MERS.CoV.S1.RBD.367.606.rFcTag	feature_id[3].value <= threshold=2789.3416748046875
node_124: feature_name=SARS.CoV.2.S1+S2	feature_id[18].value > threshold=41798.25
node_140: feature_name=SARS.CoV.2.S1.RBD.mFc	feature_id[17].value <= threshold=44491.166015625
node_141: feature_name=a-HuIgM_0.03	feature_id[34].value > threshold=-6.833333492279053
node_149: feature_name=a-MoIgA_0.30	feature_id[35].value > threshold=935.3333435058594
node_151: feature_name=HuIgM_0.30	feature_id[13].value <= threshold=1110.5
node_152: feature_name=a-HuIgG_0.30	feature_id[30].value > threshold=46032.0
node_154: feature_name=a-MoIgM_0.03	feature_id[41].value <= threshold=1308.5
Class: healthcare workers between 60 and 180 days after vaccination	
Rules_155	passed counts:1
node_0: feature_name=HuIgG_0.03	feature_id[44].value > threshold=3298.333251953125
node_78: feature_name=a-HuIgG_0.03	feature_id[1].value <= threshold=19665.666015625
node_79: feature_name=SARS.CoV.2.S1.mFcTag	feature_id[0].value <= threshold=54010.166015625
node_80: feature_name=SARS.CoV.2.S1+S2	feature_id[18].value <= threshold=48317.708984375
node_81: feature_name=SARS.CoV.2.Spike.RBD.His.Bac	feature_id[9].value > threshold=16347.4580078125
node_111: feature_name=hCoV.NL63.S1	feature_id[5].value > threshold=4866.533447265625
node_123: feature_name=MERS.CoV.S1.RBD.367.606.rFcTag	feature_id[3].value <= threshold=2789.3416748046875
node_124: feature_name=SARS.CoV.2.S1+S2	feature_id[18].value > threshold=41798.25
node_140: feature_name=SARS.CoV.2.S1.RBD.mFc	feature_id[17].value <= threshold=44491.166015625
node_141: feature_name=a-HuIgM_0.03	feature_id[34].value <= threshold=-6.833333492279053

node_142: feature_name=hCoV.OC43.HE	feature_id[10].value > threshold=7956.5
node_144: feature_name=SARS.CoV.2.S1.mFcTag	feature_id[0].value > threshold=52734.0
Class: healthcare workers within 60 days after vaccination	
Rules_156	passed counts:1
node_0: feature_name=HuIgG_0.03	feature_id[44].value > threshold=3298.333251953125
node_78: feature_name=a-HuIgG_0.03	feature_id[1].value <= threshold=19665.666015625
node_79: feature_name=SARS.CoV.2.S1.mFcTag	feature_id[0].value <= threshold=54010.166015625
node_80: feature_name=SARS.CoV.2.S1+S2	feature_id[18].value <= threshold=48317.708984375
node_81: feature_name=SARS.CoV.2.Spike.RBD.His.Bac	feature_id[9].value > threshold=16347.4580078125
node_111: feature_name=hCoV.NL63.S1	feature_id[5].value > threshold=4866.533447265625
node_123: feature_name=MERS.CoV.S1.RBD.367.606.rFcTag	feature_id[3].value <= threshold=2789.3416748046875
node_124: feature_name=SARS.CoV.2.S1+S2	feature_id[18].value > threshold=41798.25
node_140: feature_name=SARS.CoV.2.S1.RBD.mFc	feature_id[17].value <= threshold=44491.166015625
node_141: feature_name=a-HuIgM_0.03	feature_id[34].value <= threshold=-6.833333492279053
node_142: feature_name=hCoV.OC43.HE	feature_id[10].value > threshold=7956.5
node_144: feature_name=SARS.CoV.2.S1.mFcTag	feature_id[0].value <= threshold=52734.0
node_145: feature_name=Flu.H1N1.HA1	feature_id[26].value <= threshold=26772.75
Class: healthcare workers within 60 days after vaccination	
Rules_157	passed counts:1
node_0: feature_name=HuIgG_0.03	feature_id[44].value > threshold=3298.333251953125
node_78: feature_name=a-HuIgG_0.03	feature_id[1].value <= threshold=19665.666015625
node_79: feature_name=SARS.CoV.2.S1.mFcTag	feature_id[0].value <= threshold=54010.166015625
node_80: feature_name=SARS.CoV.2.S1+S2	feature_id[18].value <= threshold=48317.708984375

node_81: feature_name=SARS.CoV.2.Spike.RBD.His.Bac	feature_id[9].value > threshold=16347.4580078125
node_111: feature_name=hCoV.NL63.S1	feature_id[5].value > threshold=4866.533447265625
node_123: feature_name=MERS.CoV.S1.RBD.367.606.rFcTag	feature_id[3].value <= threshold=2789.3416748046875
node_124: feature_name=SARS.CoV.2.S1+S2	feature_id[18].value <= threshold=41798.25
node_125: feature_name=hCoV.OC43.S1_S2ECD.HisTag	feature_id[24].value <= threshold=49554.1328125
node_126: feature_name=SARS.CoV.2.Spike.RBD.His.Bac	feature_id[9].value > threshold=23295.4169921875
node_130: feature_name=Flu.H1N1.HA1	feature_id[26].value > threshold=30553.0
node_134: feature_name=MERS.CoV.S1.RBD.367.606.rFcTag	feature_id[3].value > threshold=983.2499694824219
node_136: feature_name=a-MoIgA_0.03	feature_id[37].value > threshold=503.50001525878906
Class: healthcare workers over 180 days after vaccination	
Rules_158	passed counts:1
node_0: feature_name=HuIgG_0.03	feature_id[44].value > threshold=3298.333251953125
node_78: feature_name=a-HuIgG_0.03	feature_id[1].value <= threshold=19665.666015625
node_79: feature_name=SARS.CoV.2.S1.mFcTag	feature_id[0].value <= threshold=54010.166015625
node_80: feature_name=SARS.CoV.2.S1+S2	feature_id[18].value <= threshold=48317.708984375
node_81: feature_name=SARS.CoV.2.Spike.RBD.His.Bac	feature_id[9].value > threshold=16347.4580078125
node_111: feature_name=hCoV.NL63.S1	feature_id[5].value > threshold=4866.533447265625
node_123: feature_name=MERS.CoV.S1.RBD.367.606.rFcTag	feature_id[3].value <= threshold=2789.3416748046875
node_124: feature_name=SARS.CoV.2.S1+S2	feature_id[18].value <= threshold=41798.25
node_125: feature_name=hCoV.OC43.S1_S2ECD.HisTag	feature_id[24].value <= threshold=49554.1328125
node_126: feature_name=SARS.CoV.2.Spike.RBD.His.Bac	feature_id[9].value > threshold=23295.4169921875
node_130: feature_name=Flu.H1N1.HA1	feature_id[26].value > threshold=30553.0
node_134: feature_name=MERS.CoV.S1.RBD.367.606.rFcTag	feature_id[3].value <= threshold=983.2499694824219
Class: healthcare workers within 60 days after vaccination	

Rules_159	passed counts:1
node_0: feature_name=HuIgG_0.03	feature_id[44].value > threshold=3298.333251953125
node_78: feature_name=a-HuIgG_0.03	feature_id[1].value <= threshold=19665.666015625
node_79: feature_name=SARS.CoV.2.S1.mFcTag	feature_id[0].value <= threshold=54010.166015625
node_80: feature_name=SARS.CoV.2.S1+S2	feature_id[18].value <= threshold=48317.708984375
node_81: feature_name=SARS.CoV.2.Spike.RBD.His.Bac	feature_id[9].value > threshold=16347.4580078125
node_111: feature_name=hCoV.NL63.S1	feature_id[5].value > threshold=4866.533447265625
node_123: feature_name=MERS.CoV.S1.RBD.367.606.rFcTag	feature_id[3].value <= threshold=2789.3416748046875
node_124: feature_name=SARS.CoV.2.S1+S2	feature_id[18].value <= threshold=41798.25
node_125: feature_name=hCoV.OC43.S1_S2ECD.HisTag	feature_id[24].value <= threshold=49554.1328125
node_126: feature_name=SARS.CoV.2.Spike.RBD.His.Bac	feature_id[9].value > threshold=23295.4169921875
node_130: feature_name=Flu.H1N1.HA1	feature_id[26].value <= threshold=30553.0
node_131: feature_name=MERS.CoV.S1.ECD.1-1297.HisTag	feature_id[12].value <= threshold=13902.69140625
Class: healthcare workers between 60 and 180 days after vaccination	
Rules_160	passed counts:1
node_0: feature_name=HuIgG_0.03	feature_id[44].value > threshold=3298.333251953125
node_78: feature_name=a-HuIgG_0.03	feature_id[1].value <= threshold=19665.666015625
node_79: feature_name=SARS.CoV.2.S1.mFcTag	feature_id[0].value <= threshold=54010.166015625
node_80: feature_name=SARS.CoV.2.S1+S2	feature_id[18].value <= threshold=48317.708984375
node_81: feature_name=SARS.CoV.2.Spike.RBD.His.Bac	feature_id[9].value > threshold=16347.4580078125
node_111: feature_name=hCoV.NL63.S1	feature_id[5].value <= threshold=4866.533447265625
node_112: feature_name=SARS.CoV.2.S1.mFcTag	feature_id[0].value > threshold=40690.80078125
node_116: feature_name=a-MoIgG_0.10	feature_id[38].value > threshold=2424.6666259765625

node_118: feature_name=DcCoV.HKU23.NP	feature_id[21].value > threshold=3458.25
node_120: feature_name=hCoV.OC43.S1_S2ECD.HisTag	feature_id[24].value > threshold=48433.232421875
Class: healthcare workers between 60 and 180 days after vaccination	
Rules_161	passed counts:1
node_0: feature_name=HuIgG_0.03	feature_id[44].value > threshold=3298.333251953125
node_78: feature_name=a-HuIgG_0.03	feature_id[1].value <= threshold=19665.666015625
node_79: feature_name=SARS.CoV.2.S1.mFcTag	feature_id[0].value <= threshold=54010.166015625
node_80: feature_name=SARS.CoV.2.S1+S2	feature_id[18].value <= threshold=48317.708984375
node_81: feature_name=SARS.CoV.2.Spike.RBD.His.Bac	feature_id[9].value > threshold=16347.4580078125
node_111: feature_name=hCoV.NL63.S1	feature_id[5].value <= threshold=4866.533447265625
node_112: feature_name=SARS.CoV.2.S1.mFcTag	feature_id[0].value > threshold=40690.80078125
node_116: feature_name=a-MoIgG_0.10	feature_id[38].value > threshold=2424.6666259765625
node_118: feature_name=DcCoV.HKU23.NP	feature_id[21].value <= threshold=3458.25
Class: healthcare workers between 60 and 180 days after vaccination	
Rules_162	passed counts:1
node_0: feature_name=HuIgG_0.03	feature_id[44].value > threshold=3298.333251953125
node_78: feature_name=a-HuIgG_0.03	feature_id[1].value <= threshold=19665.666015625
node_79: feature_name=SARS.CoV.2.S1.mFcTag	feature_id[0].value <= threshold=54010.166015625
node_80: feature_name=SARS.CoV.2.S1+S2	feature_id[18].value <= threshold=48317.708984375
node_81: feature_name=SARS.CoV.2.Spike.RBD.His.Bac	feature_id[9].value > threshold=16347.4580078125
node_111: feature_name=hCoV.NL63.S1	feature_id[5].value <= threshold=4866.533447265625
node_112: feature_name=SARS.CoV.2.S1.mFcTag	feature_id[0].value <= threshold=40690.80078125
node_113: feature_name=a-HuIgM_0.30	feature_id[32].value <= threshold=1087.5

Class: healthcare workers over 180 days after vaccination	
Rules_163	passed counts:1
node_0: feature_name=HuIgG_0.03	feature_id[44].value > threshold=3298.333251953125
node_78: feature_name=a-HuIgG_0.03	feature_id[1].value <= threshold=19665.666015625
node_79: feature_name=SARS.CoV.2.S1.mFcTag	feature_id[0].value <= threshold=54010.166015625
node_80: feature_name=SARS.CoV.2.S1+S2	feature_id[18].value <= threshold=48317.708984375
node_81: feature_name=SARS.CoV.2.Spike.RBD.His.Bac	feature_id[9].value <= threshold=16347.4580078125
node_82: feature_name=SARS.CoV.2.S1	feature_id[15].value > threshold=2844.6334228515625
node_90: feature_name=a-MoIgG_0.10	feature_id[38].value > threshold=1482.3333129882812
node_98: feature_name=Flu.H1N1.HA1	feature_id[26].value > threshold=52934.66015625
node_108: feature_name=a-MoIgA_0.03	feature_id[37].value <= threshold=26.999999046325684
Class: healthcare workers within 60 days after vaccination	
Rules_164	passed counts:1
node_0: feature_name=HuIgG_0.03	feature_id[44].value > threshold=3298.333251953125
node_78: feature_name=a-HuIgG_0.03	feature_id[1].value <= threshold=19665.666015625
node_79: feature_name=SARS.CoV.2.S1.mFcTag	feature_id[0].value <= threshold=54010.166015625
node_80: feature_name=SARS.CoV.2.S1+S2	feature_id[18].value <= threshold=48317.708984375
node_81: feature_name=SARS.CoV.2.Spike.RBD.His.Bac	feature_id[9].value <= threshold=16347.4580078125
node_82: feature_name=SARS.CoV.2.S1	feature_id[15].value > threshold=2844.6334228515625
node_90: feature_name=a-MoIgG_0.10	feature_id[38].value > threshold=1482.3333129882812
node_98: feature_name=Flu.H1N1.HA1	feature_id[26].value <= threshold=52934.66015625
node_99: feature_name=Flu.B_Phu/.HA1	feature_id[11].value > threshold=27915.5419921875
node_105: feature_name=SARS.CoV.NP	feature_id[19].value <= threshold=310.9027671813965

Class: unvaccinated healthcare workers	
Rules_165	passed counts:1
node_0: feature_name=HuIgG_0.03	feature_id[44].value > threshold=3298.333251953125
node_78: feature_name=a-HuIgG_0.03	feature_id[1].value <= threshold=19665.666015625
node_79: feature_name=SARS.CoV.2.S1.mFcTag	feature_id[0].value <= threshold=54010.166015625
node_80: feature_name=SARS.CoV.2.S1+S2	feature_id[18].value <= threshold=48317.708984375
node_81: feature_name=SARS.CoV.2.Spike.RBD.His.Bac	feature_id[9].value <= threshold=16347.4580078125
node_82: feature_name=SARS.CoV.2.S1	feature_id[15].value > threshold=2844.6334228515625
node_90: feature_name=a-MoIgG_0.10	feature_id[38].value > threshold=1482.3333129882812
node_98: feature_name=Flu.H1N1.HA1	feature_id[26].value <= threshold=52934.66015625
node_99: feature_name=Flu.B_Phu/.HA1	feature_id[11].value <= threshold=27915.5419921875
node_100: feature_name=hCoV.229E.S1	feature_id[2].value <= threshold=33022.865234375
node_101: feature_name=SARS.CoV.2.S1.mFcTag	feature_id[0].value > threshold=25623.3671875
Class: healthcare workers between 60 and 180 days after vaccination	
Rules_166	passed counts:1
node_0: feature_name=HuIgG_0.03	feature_id[44].value > threshold=3298.333251953125
node_78: feature_name=a-HuIgG_0.03	feature_id[1].value <= threshold=19665.666015625
node_79: feature_name=SARS.CoV.2.S1.mFcTag	feature_id[0].value <= threshold=54010.166015625
node_80: feature_name=SARS.CoV.2.S1+S2	feature_id[18].value <= threshold=48317.708984375
node_81: feature_name=SARS.CoV.2.Spike.RBD.His.Bac	feature_id[9].value <= threshold=16347.4580078125
node_82: feature_name=SARS.CoV.2.S1	feature_id[15].value > threshold=2844.6334228515625
node_90: feature_name=a-MoIgG_0.10	feature_id[38].value <= threshold=1482.3333129882812
node_91: feature_name=SARS.CoV.2.Spike.RBD.rFc	feature_id[7].value <= threshold=6995.25

node_92: feature_name=SARS.CoV.2.Spike.RBD.rFc	feature_id[7].value > threshold=210.11111068725586
node_94: feature_name=a-HuIgM_0.30	feature_id[32].value > threshold=1457.0
Class: healthcare workers within 60 days after vaccination	
Rules_167	passed counts:1
node_0: feature_name=HuIgG_0.03	feature_id[44].value > threshold=3298.333251953125
node_78: feature_name=a-HuIgG_0.03	feature_id[1].value <= threshold=19665.666015625
node_79: feature_name=SARS.CoV.2.S1.mFcTag	feature_id[0].value <= threshold=54010.166015625
node_80: feature_name=SARS.CoV.2.S1+S2	feature_id[18].value <= threshold=48317.708984375
node_81: feature_name=SARS.CoV.2.Spike.RBD.His.Bac	feature_id[9].value <= threshold=16347.4580078125
node_82: feature_name=SARS.CoV.2.S1	feature_id[15].value > threshold=2844.6334228515625
node_90: feature_name=a-MoIgG_0.10	feature_id[38].value <= threshold=1482.3333129882812
node_91: feature_name=SARS.CoV.2.Spike.RBD.rFc	feature_id[7].value <= threshold=6995.25
node_92: feature_name=SARS.CoV.2.Spike.RBD.rFc	feature_id[7].value > threshold=210.11111068725586
node_94: feature_name=a-HuIgM_0.30	feature_id[32].value <= threshold=1457.0
Class: healthcare workers between 60 and 180 days after vaccination	
Rules_168	passed counts:1
node_0: feature_name=HuIgG_0.03	feature_id[44].value > threshold=3298.333251953125
node_78: feature_name=a-HuIgG_0.03	feature_id[1].value <= threshold=19665.666015625
node_79: feature_name=SARS.CoV.2.S1.mFcTag	feature_id[0].value <= threshold=54010.166015625
node_80: feature_name=SARS.CoV.2.S1+S2	feature_id[18].value <= threshold=48317.708984375
node_81: feature_name=SARS.CoV.2.Spike.RBD.His.Bac	feature_id[9].value <= threshold=16347.4580078125
node_82: feature_name=SARS.CoV.2.S1	feature_id[15].value > threshold=2844.6334228515625
node_90: feature_name=a-MoIgG_0.10	feature_id[38].value <= threshold=1482.3333129882812

node_91: feature_name=SARS.CoV.2.Spike.RBD.rFc	feature_id[7].value <= threshold=6995.25
node_92: feature_name=SARS.CoV.2.Spike.RBD.rFc	feature_id[7].value <= threshold=210.11111068725586
Class: unvaccinated healthcare workers	
Rules_169	passed counts:1
node_0: feature_name=HuIgG_0.03	feature_id[44].value > threshold=3298.333251953125
node_78: feature_name=a-HuIgG_0.03	feature_id[1].value <= threshold=19665.666015625
node_79: feature_name=SARS.CoV.2.S1.mFcTag	feature_id[0].value <= threshold=54010.166015625
node_80: feature_name=SARS.CoV.2.S1+S2	feature_id[18].value <= threshold=48317.708984375
node_81: feature_name=SARS.CoV.2.Spike.RBD.His.Bac	feature_id[9].value <= threshold=16347.4580078125
node_82: feature_name=SARS.CoV.2.S1	feature_id[15].value <= threshold=2844.6334228515625
node_83: feature_name=SARS.CoV.2.S1+S2	feature_id[18].value <= threshold=3298.25
node_84: feature_name=hCoV.OC43.HE	feature_id[10].value <= threshold=-45.900001525878906
node_85: feature_name=SARS.CoV.2.Spike.RBD.His.Bac	feature_id[9].value > threshold=-318.49999237060547
Class: healthcare workers between 60 and 180 days after vaccination	
Rules_170	passed counts:1
node_0: feature_name=HuIgG_0.03	feature_id[44].value > threshold=3298.333251953125
node_78: feature_name=a-HuIgG_0.03	feature_id[1].value <= threshold=19665.666015625
node_79: feature_name=SARS.CoV.2.S1.mFcTag	feature_id[0].value <= threshold=54010.166015625
node_80: feature_name=SARS.CoV.2.S1+S2	feature_id[18].value <= threshold=48317.708984375
node_81: feature_name=SARS.CoV.2.Spike.RBD.His.Bac	feature_id[9].value <= threshold=16347.4580078125
node_82: feature_name=SARS.CoV.2.S1	feature_id[15].value <= threshold=2844.6334228515625
node_83: feature_name=SARS.CoV.2.S1+S2	feature_id[18].value <= threshold=3298.25
node_84: feature_name=hCoV.OC43.HE	feature_id[10].value <= threshold=-45.900001525878906

node_85: feature_name=SARS.CoV.2.Spike.RBD.His.Bac	feature_id[9].value <= threshold=-318.49999237060547
Class: healthcare workers over 180 days after vaccination	
Rules_171	passed counts:1
node_0: feature_name=HuIgG_0.03	feature_id[44].value <= threshold=3298.333251953125
node_1: feature_name=SARS.CoV.2.S1.mFcTag	feature_id[0].value > threshold=5354.388916015625
node_9: feature_name=HuIgM_0.03	feature_id[45].value > threshold=-80.66666793823242
node_27: feature_name=HuIgM_0.03	feature_id[45].value > threshold=61.33333206176758
node_75: feature_name=HuIgA_0.10	feature_id[42].value > threshold=1386.3333435058594
Class: healthcare workers between 60 and 180 days after vaccination	
Rules_172	passed counts:1
node_0: feature_name=HuIgG_0.03	feature_id[44].value <= threshold=3298.333251953125
node_1: feature_name=SARS.CoV.2.S1.mFcTag	feature_id[0].value > threshold=5354.388916015625
node_9: feature_name=HuIgM_0.03	feature_id[45].value > threshold=-80.66666793823242
node_27: feature_name=HuIgM_0.03	feature_id[45].value <= threshold=61.33333206176758
node_28: feature_name=a-HuIgG_0.30	feature_id[30].value > threshold=34878.66796875
node_64: feature_name=a-HuIgG_0.03	feature_id[1].value > threshold=9340.3330078125
node_68: feature_name=a-HuIgA_0.30	feature_id[27].value > threshold=1918.1666870117188
node_72: feature_name=HuIgM_0.03	feature_id[45].value > threshold=20.333333492279053
Class: healthcare workers between 60 and 180 days after vaccination	
Rules_173	passed counts:1
node_0: feature_name=HuIgG_0.03	feature_id[44].value <= threshold=3298.333251953125
node_1: feature_name=SARS.CoV.2.S1.mFcTag	feature_id[0].value > threshold=5354.388916015625

node_9: feature_name=HuIgM_0.03	feature_id[45].value > threshold=-80.66666793823242
node_27: feature_name=HuIgM_0.03	feature_id[45].value <= threshold=61.33333206176758
node_28: feature_name=a-HuIgG_0.30	feature_id[30].value > threshold=34878.66796875
node_64: feature_name=a-HuIgG_0.03	feature_id[1].value > threshold=9340.3330078125
node_68: feature_name=a-HuIgA_0.30	feature_id[27].value <= threshold=1918.1666870117188
node_69: feature_name=SARS.CoV.2.Spike.RBD.rFc	feature_id[7].value <= threshold=20694.49951171875
Class: healthcare workers over 180 days after vaccination	
Rules_174	passed counts:1
node_0: feature_name=HuIgG_0.03	feature_id[44].value <= threshold=3298.333251953125
node_1: feature_name=SARS.CoV.2.S1.mFcTag	feature_id[0].value > threshold=5354.388916015625
node_9: feature_name=HuIgM_0.03	feature_id[45].value > threshold=-80.66666793823242
node_27: feature_name=HuIgM_0.03	feature_id[45].value <= threshold=61.33333206176758
node_28: feature_name=a-HuIgG_0.30	feature_id[30].value <= threshold=34878.66796875
node_29: feature_name=HuIgG_0.03	feature_id[44].value > threshold=769.8333435058594
node_35: feature_name=SARS.CoV.S1.HisTag	feature_id[14].value > threshold=400.3000030517578
node_39: feature_name=hCoV.HKU1.S1_S2	feature_id[22].value > threshold=42662.666015625
node_61: feature_name=SARS.CoV.2.S1.HisTag	feature_id[16].value > threshold=41690.958984375
Class: healthcare workers between 60 and 180 days after vaccination	
Rules_175	passed counts:1
node_0: feature_name=HuIgG_0.03	feature_id[44].value <= threshold=3298.333251953125
node_1: feature_name=SARS.CoV.2.S1.mFcTag	feature_id[0].value > threshold=5354.388916015625
node_9: feature_name=HuIgM_0.03	feature_id[45].value > threshold=-80.66666793823242
node_27: feature_name=HuIgM_0.03	feature_id[45].value <= threshold=61.33333206176758

node_28: feature_name=a-HuIgG_0.30	feature_id[30].value <= threshold=34878.66796875
node_29: feature_name=HuIgG_0.03	feature_id[44].value > threshold=769.8333435058594
node_35: feature_name=SARS.CoV.S1.HisTag	feature_id[14].value > threshold=400.3000030517578
node_39: feature_name=hCoV.HKU1.S1_S2	feature_id[22].value <= threshold=42662.666015625
node_40: feature_name=Flu.B_Phu/.HA1	feature_id[11].value > threshold=49118.416015625
Class: healthcare workers over 180 days after vaccination	
Rules_176	passed counts:1
node_0: feature_name=HuIgG_0.03	feature_id[44].value <= threshold=3298.333251953125
node_1: feature_name=SARS.CoV.2.S1.mFcTag	feature_id[0].value > threshold=5354.388916015625
node_9: feature_name=HuIgM_0.03	feature_id[45].value > threshold=-80.66666793823242
node_27: feature_name=HuIgM_0.03	feature_id[45].value <= threshold=61.33333206176758
node_28: feature_name=a-HuIgG_0.30	feature_id[30].value <= threshold=34878.66796875
node_29: feature_name=HuIgG_0.03	feature_id[44].value > threshold=769.8333435058594
node_35: feature_name=SARS.CoV.S1.HisTag	feature_id[14].value > threshold=400.3000030517578
node_39: feature_name=hCoV.HKU1.S1_S2	feature_id[22].value <= threshold=42662.666015625
node_40: feature_name=Flu.B_Phu/.HA1	feature_id[11].value <= threshold=49118.416015625
node_41: feature_name=hCoV.OC43.HE	feature_id[10].value > threshold=25184.06640625
Class: healthcare workers over 180 days after vaccination	
Rules_177	passed counts:1
node_0: feature_name=HuIgG_0.03	feature_id[44].value <= threshold=3298.333251953125
node_1: feature_name=SARS.CoV.2.S1.mFcTag	feature_id[0].value > threshold=5354.388916015625
node_9: feature_name=HuIgM_0.03	feature_id[45].value > threshold=-80.66666793823242
node_27: feature_name=HuIgM_0.03	feature_id[45].value <= threshold=61.33333206176758

node_28: feature_name=a-HuIgG_0.30	feature_id[30].value <= threshold=34878.66796875
node_29: feature_name=HuIgG_0.03	feature_id[44].value > threshold=769.8333435058594
node_35: feature_name=SARS.CoV.S1.HisTag	feature_id[14].value > threshold=400.3000030517578
node_39: feature_name=hCoV.HKU1.S1_S2	feature_id[22].value <= threshold=42662.666015625
node_40: feature_name=Flu.B_Phu/.HA1	feature_id[11].value <= threshold=49118.416015625
node_41: feature_name=hCoV.OC43.HE	feature_id[10].value <= threshold=25184.06640625
node_42: feature_name=HuIgM_0.30	feature_id[13].value > threshold=1223.5
node_56: feature_name=Flu.B_Mal/.HA1	feature_id[4].value > threshold=14985.33349609375
Class: healthcare workers within 60 days after vaccination	
Rules_178	passed counts:1
node_0: feature_name=HuIgG_0.03	feature_id[44].value <= threshold=3298.333251953125
node_1: feature_name=SARS.CoV.2.S1.mFcTag	feature_id[0].value > threshold=5354.388916015625
node_9: feature_name=HuIgM_0.03	feature_id[45].value > threshold=-80.66666793823242
node_27: feature_name=HuIgM_0.03	feature_id[45].value <= threshold=61.33333206176758
node_28: feature_name=a-HuIgG_0.30	feature_id[30].value <= threshold=34878.66796875
node_29: feature_name=HuIgG_0.03	feature_id[44].value > threshold=769.8333435058594
node_35: feature_name=SARS.CoV.S1.HisTag	feature_id[14].value > threshold=400.3000030517578
node_39: feature_name=hCoV.HKU1.S1_S2	feature_id[22].value <= threshold=42662.666015625
node_40: feature_name=Flu.B_Phu/.HA1	feature_id[11].value <= threshold=49118.416015625
node_41: feature_name=hCoV.OC43.HE	feature_id[10].value <= threshold=25184.06640625
node_42: feature_name=HuIgM_0.30	feature_id[13].value > threshold=1223.5
node_56: feature_name=Flu.B_Mal/.HA1	feature_id[4].value <= threshold=14985.33349609375
Class: healthcare workers between 60 and 180 days after vaccination	

Rules_179	passed counts:1
node_0: feature_name=HuIgG_0.03	feature_id[44].value <= threshold=3298.333251953125
node_1: feature_name=SARS.CoV.2.S1.mFcTag	feature_id[0].value > threshold=5354.388916015625
node_9: feature_name=HuIgM_0.03	feature_id[45].value > threshold=-80.66666793823242
node_27: feature_name=HuIgM_0.03	feature_id[45].value <= threshold=61.33333206176758
node_28: feature_name=a-HuIgG_0.30	feature_id[30].value <= threshold=34878.66796875
node_29: feature_name=HuIgG_0.03	feature_id[44].value > threshold=769.8333435058594
node_35: feature_name=SARS.CoV.S1.HisTag	feature_id[14].value > threshold=400.3000030517578
node_39: feature_name=hCoV.HKU1.S1_S2	feature_id[22].value <= threshold=42662.666015625
node_40: feature_name=Flu.B_Phu/.HA1	feature_id[11].value <= threshold=49118.416015625
node_41: feature_name=hCoV.OC43.HE	feature_id[10].value <= threshold=25184.06640625
node_42: feature_name=HuIgM_0.30	feature_id[13].value <= threshold=1223.5
node_43: feature_name=a-HuIgA_0.03	feature_id[29].value > threshold=-59.66666603088379
node_47: feature_name=SARS.CoV.S1.HisTag	feature_id[14].value > threshold=25028.0
node_53: feature_name=a-HuIgA_0.30	feature_id[27].value <= threshold=976.3333435058594
Class: healthcare workers within 60 days after vaccination	
Rules_180	passed counts:1
node_0: feature_name=HuIgG_0.03	feature_id[44].value <= threshold=3298.333251953125
node_1: feature_name=SARS.CoV.2.S1.mFcTag	feature_id[0].value > threshold=5354.388916015625
node_9: feature_name=HuIgM_0.03	feature_id[45].value > threshold=-80.66666793823242
node_27: feature_name=HuIgM_0.03	feature_id[45].value <= threshold=61.33333206176758
node_28: feature_name=a-HuIgG_0.30	feature_id[30].value <= threshold=34878.66796875
node_29: feature_name=HuIgG_0.03	feature_id[44].value > threshold=769.8333435058594
node_35: feature_name=SARS.CoV.S1.HisTag	feature_id[14].value > threshold=400.3000030517578

node_39: feature_name=hCoV.HKU1.S1_S2	feature_id[22].value <= threshold=42662.666015625
node_40: feature_name=Flu.B_Phu/.HA1	feature_id[11].value <= threshold=49118.416015625
node_41: feature_name=hCoV.OC43.HE	feature_id[10].value <= threshold=25184.06640625
node_42: feature_name=HuIgM_0.30	feature_id[13].value <= threshold=1223.5
node_43: feature_name=a-HuIgA_0.03	feature_id[29].value > threshold=-59.66666603088379
node_47: feature_name=SARS.CoV.S1.HisTag	feature_id[14].value <= threshold=25028.0
node_48: feature_name=HuIgM_0.03	feature_id[45].value > threshold=35.33333206176758
node_50: feature_name=Flu.H1N1.HA1	feature_id[26].value > threshold=36011.291015625
Class: healthcare workers over 180 days after vaccination	
Rules_181	passed counts:1
node_0: feature_name=HuIgG_0.03	feature_id[44].value <= threshold=3298.333251953125
node_1: feature_name=SARS.CoV.2.S1.mFcTag	feature_id[0].value > threshold=5354.388916015625
node_9: feature_name=HuIgM_0.03	feature_id[45].value > threshold=-80.66666793823242
node_27: feature_name=HuIgM_0.03	feature_id[45].value <= threshold=61.33333206176758
node_28: feature_name=a-HuIgG_0.30	feature_id[30].value <= threshold=34878.66796875
node_29: feature_name=HuIgG_0.03	feature_id[44].value > threshold=769.8333435058594
node_35: feature_name=SARS.CoV.S1.HisTag	feature_id[14].value > threshold=400.3000030517578
node_39: feature_name=hCoV.HKU1.S1_S2	feature_id[22].value <= threshold=42662.666015625
node_40: feature_name=Flu.B_Phu/.HA1	feature_id[11].value <= threshold=49118.416015625
node_41: feature_name=hCoV.OC43.HE	feature_id[10].value <= threshold=25184.06640625
node_42: feature_name=HuIgM_0.30	feature_id[13].value <= threshold=1223.5
node_43: feature_name=a-HuIgA_0.03	feature_id[29].value <= threshold=-59.66666603088379
node_44: feature_name=Flu.B_Phu/.HA1	feature_id[11].value > threshold=28467.833984375
Class: healthcare workers within 60 days after vaccination	

Rules_182	passed counts:1
node_0: feature_name=HuIgG_0.03	feature_id[44].value <= threshold=3298.333251953125
node_1: feature_name=SARS.CoV.2.S1.mFcTag	feature_id[0].value > threshold=5354.388916015625
node_9: feature_name=HuIgM_0.03	feature_id[45].value > threshold=-80.66666793823242
node_27: feature_name=HuIgM_0.03	feature_id[45].value <= threshold=61.33333206176758
node_28: feature_name=a-HuIgG_0.30	feature_id[30].value <= threshold=34878.66796875
node_29: feature_name=HuIgG_0.03	feature_id[44].value > threshold=769.8333435058594
node_35: feature_name=SARS.CoV.S1.HisTag	feature_id[14].value <= threshold=400.3000030517578
node_36: feature_name=HuIgA_0.10	feature_id[42].value > threshold=4194.666564941406
Class: healthcare workers between 60 and 180 days after vaccination	
Rules_183	passed counts:1
node_0: feature_name=HuIgG_0.03	feature_id[44].value <= threshold=3298.333251953125
node_1: feature_name=SARS.CoV.2.S1.mFcTag	feature_id[0].value > threshold=5354.388916015625
node_9: feature_name=HuIgM_0.03	feature_id[45].value > threshold=-80.66666793823242
node_27: feature_name=HuIgM_0.03	feature_id[45].value <= threshold=61.33333206176758
node_28: feature_name=a-HuIgG_0.30	feature_id[30].value <= threshold=34878.66796875
node_29: feature_name=HuIgG_0.03	feature_id[44].value <= threshold=769.8333435058594
node_30: feature_name=SARS.CoV.2.Spike.RBD.His.Bac	feature_id[9].value > threshold=12895.0166015625
node_32: feature_name=a-HuIgM_0.03	feature_id[34].value <= threshold=-72.50000190734863
Class: healthcare workers over 180 days after vaccination	
Rules_184	passed counts:1
node_0: feature_name=HuIgG_0.03	feature_id[44].value <= threshold=3298.333251953125

node_1: feature_name=SARS.CoV.2.S1.mFcTag	feature_id[0].value > threshold=5354.388916015625
node_9: feature_name=HuIgM_0.03	feature_id[45].value <= threshold=-80.66666793823242
node_10: feature_name=SARS.CoV.2.Spike.RBD.His.Bac	feature_id[9].value > threshold=29393.55859375
node_18: feature_name=Flu.B_Mal/.HA1	feature_id[4].value > threshold=38994.25
node_20: feature_name=SARS.CoV.S1.HisTag	feature_id[14].value > threshold=11923.35009765625
node_24: feature_name=SARS.CoV.2.S1.RBD.mFc	feature_id[17].value <= threshold=44304.56640625
Class: healthcare workers between 60 and 180 days after vaccination	
Rules_185	passed counts:1
node_0: feature_name=HuIgG_0.03	feature_id[44].value <= threshold=3298.333251953125
node_1: feature_name=SARS.CoV.2.S1.mFcTag	feature_id[0].value > threshold=5354.388916015625
node_9: feature_name=HuIgM_0.03	feature_id[45].value <= threshold=-80.66666793823242
node_10: feature_name=SARS.CoV.2.Spike.RBD.His.Bac	feature_id[9].value > threshold=29393.55859375
node_18: feature_name=Flu.B_Mal/.HA1	feature_id[4].value > threshold=38994.25
node_20: feature_name=SARS.CoV.S1.HisTag	feature_id[14].value <= threshold=11923.35009765625
node_21: feature_name=SARS.CoV.2.Spike.RBD.rFc	feature_id[7].value <= threshold=44823.91796875
Class: healthcare workers over 180 days after vaccination	
Rules_186	passed counts:1
node_0: feature_name=HuIgG_0.03	feature_id[44].value <= threshold=3298.333251953125
node_1: feature_name=SARS.CoV.2.S1.mFcTag	feature_id[0].value > threshold=5354.388916015625
node_9: feature_name=HuIgM_0.03	feature_id[45].value <= threshold=-80.66666793823242
node_10: feature_name=SARS.CoV.2.Spike.RBD.His.Bac	feature_id[9].value <= threshold=29393.55859375
node_11: feature_name=DcCoV.HKU23.NP	feature_id[21].value > threshold=-639.3333129882812
node_13: feature_name=MERS.CoV.S1.ECD.1-1297.HisTag	feature_id[12].value <= threshold=1026.1666564941406

node_14: feature_name=SARS.CoV.2.S1+S2	feature_id[18].value > threshold=14681.66650390625
Class: healthcare workers between 60 and 180 days after vaccination	
Rules_187	passed counts:1
node_0: feature_name=HuIgG_0.03	feature_id[44].value <= threshold=3298.333251953125
node_1: feature_name=SARS.CoV.2.S1.mFcTag	feature_id[0].value > threshold=5354.388916015625
node_9: feature_name=HuIgM_0.03	feature_id[45].value <= threshold=-80.66666793823242
node_10: feature_name=SARS.CoV.2.Spike.RBD.His.Bac	feature_id[9].value <= threshold=29393.55859375
node_11: feature_name=DcCoV.HKU23.NP	feature_id[21].value <= threshold=-639.3333129882812
Class: healthcare workers between 60 and 180 days after vaccination	
Rules_188	passed counts:1
node_0: feature_name=HuIgG_0.03	feature_id[44].value <= threshold=3298.333251953125
node_1: feature_name=SARS.CoV.2.S1.mFcTag	feature_id[0].value <= threshold=5354.388916015625
node_2: feature_name=SARS.CoV.2.S1.HisTag	feature_id[16].value > threshold=-383.86944580078125
node_4: feature_name=hCoV.OC43.HE	feature_id[10].value <= threshold=414.5370330810547
node_5: feature_name=SARS.CoV.2.S1.HisTag	feature_id[16].value <= threshold=1.5555554628372192
Class: healthcare workers within 60 days after vaccination	
Rules_189	passed counts:1
node_0: feature_name=HuIgG_0.03	feature_id[44].value <= threshold=3298.333251953125
node_1: feature_name=SARS.CoV.2.S1.mFcTag	feature_id[0].value <= threshold=5354.388916015625
node_2: feature_name=SARS.CoV.2.S1.HisTag	feature_id[16].value <= threshold=-383.86944580078125
Class: healthcare workers between 60 and 180 days after vaccination	

(2) Rules on the LightGBM feature list

Rules_0	passed counts:249
node_0: feature_name=HuIgG_0.10	feature_id[2].value <= threshold=19120.166015625
node_1: feature_name=SARS.CoV.2.S1.RBD.mFc	feature_id[28].value > threshold=3773.8334350585938
node_9: feature_name=HuIgM_0.03	feature_id[14].value > threshold=-88.33333206176758
node_27: feature_name=hCoV.HKU1.S1_S2	feature_id[32].value <= threshold=42767.0
node_28: feature_name=HuIgG_0.03	feature_id[0].value > threshold=1005.0
node_38: feature_name=a-HuIgG_0.30	feature_id[4].value <= threshold=33596.333984375
node_39: feature_name=SARS.CoV.S1.HisTag	feature_id[18].value > threshold=400.3000030517578
node_43: feature_name=HuIgM_0.10	feature_id[6].value <= threshold=109.66666793823242
node_44: feature_name=MERS.CoV.NP	feature_id[15].value > threshold=-50.125
node_46: feature_name=hCoV.229E.S1_S2	feature_id[35].value <= threshold=42044.208984375
node_47: feature_name=HuIgM_0.30	feature_id[8].value <= threshold=1223.5
node_48: feature_name=SARS.CoV.S1.RBD.rFcTag	feature_id[16].value <= threshold=27979.533203125
Class: healthcare workers between 60 and 180 days after vaccination	
Rules_1	passed counts:94
node_0: feature_name=HuIgG_0.10	feature_id[2].value > threshold=19120.166015625
node_96: feature_name=a-HuIgG_0.03	feature_id[7].value <= threshold=19665.666015625
node_97: feature_name=MERS.CoV.S1.RBD.367.606.rFcTag	feature_id[5].value <= threshold=3252.625
node_98: feature_name=SARS.CoV.2.S2	feature_id[29].value > threshold=9506.32470703125
node_116: feature_name=Flu.H1N1.HA1+HA2	feature_id[10].value <= threshold=54065.41796875
node_117: feature_name=SARS.CoV.S1.RBD.rFcTag	feature_id[16].value > threshold=24639.0
node_167: feature_name=hCoV.OC43.HE	feature_id[33].value <= threshold=32466.5830078125
node_168: feature_name=HuIgM_0.10	feature_id[6].value <= threshold=22.833333015441895

node_169: feature_name=SARS.CoV.2.S1.mFcTag	feature_id[21].value <= threshold=54847.25
node_170: feature_name=a-MoIgG_0.03	feature_id[12].value <= threshold=2342.8333740234375
Class: healthcare workers within 60 days after vaccination	
Rules_2	passed counts:76
node_0: feature_name=HuIgG_0.10	feature_id[2].value <= threshold=19120.166015625
node_1: feature_name=SARS.CoV.2.S1.RBD.mFc	feature_id[28].value <= threshold=3773.8334350585938
node_2: feature_name=SARS.CoV.2.S1.RBD.mFc	feature_id[28].value > threshold=-414.2999954223633
node_4: feature_name=hCoV.OC43.HE	feature_id[33].value > threshold=414.5370330810547
Class: unvaccinated healthcare workers	
Rules_3	passed counts:48
node_0: feature_name=HuIgG_0.10	feature_id[2].value > threshold=19120.166015625
node_96: feature_name=a-HuIgG_0.03	feature_id[7].value <= threshold=19665.666015625
node_97: feature_name=MERS.CoV.S1.RBD.367.606.rFcTag	feature_id[5].value > threshold=3252.625
node_201: feature_name=SARS.CoV.2.S2	feature_id[29].value > threshold=37167.458984375
node_301: feature_name=SARS.CoV.2.S1.mFcTag	feature_id[21].value <= threshold=53322.333984375
node_302: feature_name=MoIgG_0.03	feature_id[20].value > threshold=18.166666507720947
node_318: feature_name=Flu.B_Mal/.HA1+HA2	feature_id[22].value <= threshold=54334.25
Class: healthcare workers within 60 days after vaccination	
Rules_4	passed counts:44
node_0: feature_name=HuIgG_0.10	feature_id[2].value > threshold=19120.166015625
node_96: feature_name=a-HuIgG_0.03	feature_id[7].value <= threshold=19665.666015625
node_97: feature_name=MERS.CoV.S1.RBD.367.606.rFcTag	feature_id[5].value > threshold=3252.625

node_201: feature_name=SARS.CoV.2.S2	feature_id[29].value <= threshold=37167.458984375
node_202: feature_name=SARS.CoV.2.Spike.RBD.His.Bac	feature_id[1].value > threshold=16030.75048828125
node_212: feature_name=a-MoIgA_0.30	feature_id[37].value > threshold=1303.0
node_224: feature_name=HuIgA_0.03	feature_id[26].value > threshold=25.83333396911621
node_276: feature_name=hCoV.OC43.NP	feature_id[23].value <= threshold=3591.10009765625
node_277: feature_name=hCoV.229E.S1	feature_id[30].value > threshold=28616.0
node_287: feature_name=Flu.H3N2.HA1+HA2	feature_id[38].value <= threshold=55544.548828125
Class: healthcare workers between 60 and 180 days after vaccination	
Rules_5	passed counts:38
node_0: feature_name=HuIgG_0.10	feature_id[2].value > threshold=19120.166015625
node_96: feature_name=a-HuIgG_0.03	feature_id[7].value <= threshold=19665.666015625
node_97: feature_name=MERS.CoV.S1.RBD.367.606.rFcTag	feature_id[5].value <= threshold=3252.625
node_98: feature_name=SARS.CoV.2.S2	feature_id[29].value > threshold=9506.32470703125
node_116: feature_name=Flu.H1N1.HA1+HA2	feature_id[10].value <= threshold=54065.41796875
node_117: feature_name=SARS.CoV.S1.RBD.rFcTag	feature_id[16].value <= threshold=24639.0
node_118: feature_name=SARS.CoV.2.Spike.RBD.His.Bac	feature_id[1].value <= threshold=23841.4169921875
node_119: feature_name=SARS.CoV.2.S2	feature_id[29].value <= threshold=49775.806640625
node_120: feature_name=MERS.CoV.NP	feature_id[15].value <= threshold=8014.466796875
node_121: feature_name=Flu.H1N1.HA1+HA2	feature_id[10].value <= threshold=53033.25
Class: healthcare workers within 60 days after vaccination	
Rules_6	passed counts:37
node_0: feature_name=HuIgG_0.10	feature_id[2].value <= threshold=19120.166015625
node_1: feature_name=SARS.CoV.2.S1.RBD.mFc	feature_id[28].value > threshold=3773.8334350585938

node_9: feature_name=HuIgM_0.03	feature_id[14].value <= threshold=-88.33333206176758
node_10: feature_name=HuIgA_0.03	feature_id[26].value <= threshold=304.5
node_11: feature_name=Flu.H1N1.HA1+HA2	feature_id[10].value <= threshold=53444.666015625
node_12: feature_name=MERS.CoV.S1.RBD.367.606.rFcTag	feature_id[5].value > threshold=-2425.0
node_14: feature_name=Flu.B_Mal/.HA1+HA2	feature_id[22].value <= threshold=53898.0
Class: healthcare workers over 180 days after vaccination	
Rules_7	passed counts:27
node_0: feature_name=HuIgG_0.10	feature_id[2].value > threshold=19120.166015625
node_96: feature_name=a-HuIgG_0.03	feature_id[7].value > threshold=19665.666015625
node_350: feature_name=HuIgM_0.03	feature_id[14].value > threshold=-189.0
node_354: feature_name=HuIgM_0.30	feature_id[8].value > threshold=441.8333282470703
node_356: feature_name=a-HuIgG_0.10	feature_id[3].value > threshold=32914.5
node_360: feature_name=a-MoIgG_0.03	feature_id[12].value <= threshold=2414.8333740234375
node_361: feature_name=HuIgG_0.10	feature_id[2].value <= threshold=28475.6669921875
Class: healthcare workers over 180 days after vaccination	
Rules_8	passed counts:26
node_0: feature_name=HuIgG_0.10	feature_id[2].value > threshold=19120.166015625
node_96: feature_name=a-HuIgG_0.03	feature_id[7].value <= threshold=19665.666015625
node_97: feature_name=MERS.CoV.S1.RBD.367.606.rFcTag	feature_id[5].value > threshold=3252.625
node_201: feature_name=SARS.CoV.2.S2	feature_id[29].value <= threshold=37167.458984375
node_202: feature_name=SARS.CoV.2.Spike.RBD.His.Bac	feature_id[1].value > threshold=16030.75048828125
node_212: feature_name=a-MoIgA_0.30	feature_id[37].value > threshold=1303.0
node_224: feature_name=HuIgA_0.03	feature_id[26].value <= threshold=25.83333396911621

node_225: feature_name=Flu.B_Phu/.HA1+HA2	feature_id[19].value <= threshold=54802.5
node_226: feature_name=hCoV.HKU1.NP	feature_id[11].value <= threshold=13460.3330078125
node_227: feature_name=MoIgG_0.30	feature_id[34].value > threshold=3760.5
node_229: feature_name=HuIgM_0.10	feature_id[6].value > threshold=-87.16666412353516
node_233: feature_name=a-HuIgG_0.10	feature_id[3].value > threshold=27244.3330078125
node_235: feature_name=SARS.CoV.S1.RBD.rFcTag	feature_id[16].value > threshold=20123.43359375
node_245: feature_name=hCoV.OC43.NP	feature_id[23].value > threshold=1775.816650390625
node_253: feature_name=SARS.CoV.S1.RBD.rFcTag	feature_id[16].value > threshold=21641.4169921875
node_255: feature_name=HuIgA_0.03	feature_id[26].value <= threshold=15.333333015441895
node_256: feature_name=Flu.H3N2.HA1	feature_id[25].value <= threshold=52674.0
node_257: feature_name=hCoV.HKU1.S1_S2	feature_id[32].value > threshold=37449.416015625
node_261: feature_name=SARS.CoV.2.S1+S2	feature_id[17].value > threshold=42043.224609375
node_263: feature_name=Flu.B_Mal/.HA1	feature_id[9].value <= threshold=50467.166015625
node_264: feature_name=hCoV.OC43.HE	feature_id[33].value <= threshold=29465.6669921875
Class: healthcare workers between 60 and 180 days after vaccination	
Rules_9	passed counts:24
node_0: feature_name=HuIgG_0.10	feature_id[2].value > threshold=19120.166015625
node_96: feature_name=a-HuIgG_0.03	feature_id[7].value <= threshold=19665.666015625
node_97: feature_name=MERS.CoV.S1.RBD.367.606.rFcTag	feature_id[5].value <= threshold=3252.625
node_98: feature_name=SARS.CoV.2.S2	feature_id[29].value > threshold=9506.32470703125
node_116: feature_name=Flu.H1N1.HA1+HA2	feature_id[10].value <= threshold=54065.41796875
node_117: feature_name=SARS.CoV.S1.RBD.rFcTag	feature_id[16].value <= threshold=24639.0
node_118: feature_name=SARS.CoV.2.Spike.RBD.His.Bac	feature_id[1].value > threshold=23841.4169921875
node_128: feature_name=SARS.CoV.2.S1+S2	feature_id[17].value > threshold=41778.25

node_138: feature_name=hCoV.NL63.S1_S2	feature_id[13].value <= threshold=35816.083984375
node_139: feature_name=Flu.B_Mal/.HA1	feature_id[9].value <= threshold=46360.66796875
node_140: feature_name=MERS.CoV.NP	feature_id[15].value > threshold=809.8333129882812
Class: healthcare workers within 60 days after vaccination	
Rules_10	passed counts:23
node_0: feature_name=HuIgG_0.10	feature_id[2].value > threshold=19120.166015625
node_96: feature_name=a-HuIgG_0.03	feature_id[7].value <= threshold=19665.666015625
node_97: feature_name=MERS.CoV.S1.RBD.367.606.rFcTag	feature_id[5].value <= threshold=3252.625
node_98: feature_name=SARS.CoV.2.S2	feature_id[29].value > threshold=9506.32470703125
node_116: feature_name=Flu.H1N1.HA1+HA2	feature_id[10].value <= threshold=54065.41796875
node_117: feature_name=SARS.CoV.S1.RBD.rFcTag	feature_id[16].value > threshold=24639.0
node_167: feature_name=hCoV.OC43.HE	feature_id[33].value <= threshold=32466.5830078125
node_168: feature_name=HuIgM_0.10	feature_id[6].value > threshold=22.833333015441895
node_178: feature_name=a-MoIgA_0.03	feature_id[24].value > threshold=-34.333333015441895
node_184: feature_name=HuIgG_0.10	feature_id[2].value > threshold=21401.166015625
node_188: feature_name=SARS.CoV.2.S1.mFcTag	feature_id[21].value > threshold=51001.0
Class: healthcare workers within 60 days after vaccination	
Rules_11	passed counts:22
node_0: feature_name=HuIgG_0.10	feature_id[2].value > threshold=19120.166015625
node_96: feature_name=a-HuIgG_0.03	feature_id[7].value <= threshold=19665.666015625
node_97: feature_name=MERS.CoV.S1.RBD.367.606.rFcTag	feature_id[5].value > threshold=3252.625
node_201: feature_name=SARS.CoV.2.S2	feature_id[29].value > threshold=37167.458984375
node_301: feature_name=SARS.CoV.2.S1.mFcTag	feature_id[21].value <= threshold=53322.333984375

node_302: feature_name=MoIgG_0.03	feature_id[20].value <= threshold=18.166666507720947
node_303: feature_name=MoIgG_0.30	feature_id[34].value <= threshold=7257.166748046875
node_304: feature_name=hCoV.NL63.S1_S2	feature_id[13].value <= threshold=52607.083984375
node_305: feature_name=HuIgG_0.30	feature_id[36].value > threshold=35912.0
Class: healthcare workers within 60 days after vaccination	
Rules_12	passed counts:22
node_0: feature_name=HuIgG_0.10	feature_id[2].value > threshold=19120.166015625
node_96: feature_name=a-HuIgG_0.03	feature_id[7].value <= threshold=19665.666015625
node_97: feature_name=MERS.CoV.S1.RBD.367.606.rFcTag	feature_id[5].value > threshold=3252.625
node_201: feature_name=SARS.CoV.2.S2	feature_id[29].value <= threshold=37167.458984375
node_202: feature_name=SARS.CoV.2.Spike.RBD.His.Bac	feature_id[1].value > threshold=16030.75048828125
node_212: feature_name=a-MoIgA_0.30	feature_id[37].value <= threshold=1303.0
node_213: feature_name=MERS.CoV.NP	feature_id[15].value > threshold=898.7833251953125
node_217: feature_name=hCoV.NL63.S1_S2	feature_id[13].value > threshold=30153.3330078125
node_219: feature_name=SARS.CoV.S1.HisTag	feature_id[18].value > threshold=4305.2916259765625
Class: healthcare workers within 60 days after vaccination	
Rules_13	passed counts:20
node_0: feature_name=HuIgG_0.10	feature_id[2].value > threshold=19120.166015625
node_96: feature_name=a-HuIgG_0.03	feature_id[7].value <= threshold=19665.666015625
node_97: feature_name=MERS.CoV.S1.RBD.367.606.rFcTag	feature_id[5].value > threshold=3252.625
node_201: feature_name=SARS.CoV.2.S2	feature_id[29].value > threshold=37167.458984375
node_301: feature_name=SARS.CoV.2.S1.mFcTag	feature_id[21].value > threshold=53322.333984375
node_321: feature_name=a-MoIgA_0.03	feature_id[24].value <= threshold=212.0

node_322: feature_name=Flu.H3N2.HA1	feature_id[25].value > threshold=37503.517578125
node_330: feature_name=a-MoIgG_0.03	feature_id[12].value <= threshold=556.6666564941406
node_331: feature_name=hCoV.229E.S1_S2	feature_id[35].value <= threshold=47263.833984375
node_332: feature_name=a-HuIgG_0.10	feature_id[3].value > threshold=30393.166015625
node_336: feature_name=hCoV.NL63.S1_S2	feature_id[13].value <= threshold=53473.16796875
node_337: feature_name=hCoV.HKU1.NP	feature_id[11].value <= threshold=16009.80029296875
node_338: feature_name=hCoV.HKU1.S1_S2	feature_id[32].value > threshold=41343.134765625
Class: healthcare workers between 60 and 180 days after vaccination	
Rules_14	passed counts:17
node_0: feature_name=HuIgG_0.10	feature_id[2].value <= threshold=19120.166015625
node_1: feature_name=SARS.CoV.2.S1.RBD.mFc	feature_id[28].value > threshold=3773.8334350585938
node_9: feature_name=HuIgM_0.03	feature_id[14].value > threshold=-88.33333206176758
node_27: feature_name=hCoV.HKU1.S1_S2	feature_id[32].value <= threshold=42767.0
node_28: feature_name=HuIgG_0.03	feature_id[0].value > threshold=1005.0
node_38: feature_name=a-HuIgG_0.30	feature_id[4].value > threshold=33596.333984375
node_60: feature_name=a-HuIgG_0.03	feature_id[7].value > threshold=10366.5
node_70: feature_name=hCoV.OC43.HE	feature_id[33].value <= threshold=23182.7001953125
node_71: feature_name=SARS.CoV.2.Spike.RBD.His.Bac	feature_id[1].value <= threshold=48979.25
node_72: feature_name=HuIgM_0.10	feature_id[6].value <= threshold=40.166666984558105
Class: healthcare workers between 60 and 180 days after vaccination	
Rules_15	passed counts:16
node_0: feature_name=HuIgG_0.10	feature_id[2].value > threshold=19120.166015625
node_96: feature_name=a-HuIgG_0.03	feature_id[7].value <= threshold=19665.666015625

node_97: feature_name=MERS.CoV.S1.RBD.367.606.rFcTag	feature_id[5].value <= threshold=3252.625
node_98: feature_name=SARS.CoV.2.S2	feature_id[29].value > threshold=9506.32470703125
node_116: feature_name=Flu.H1N1.HA1+HA2	feature_id[10].value <= threshold=54065.41796875
node_117: feature_name=SARS.CoV.S1.RBD.rFcTag	feature_id[16].value <= threshold=24639.0
node_118: feature_name=SARS.CoV.2.Spike.RBD.His.Bac	feature_id[1].value > threshold=23841.4169921875
node_128: feature_name=SARS.CoV.2.S1+S2	feature_id[17].value > threshold=41778.25
node_138: feature_name=hCoV.NL63.S1_S2	feature_id[13].value > threshold=35816.083984375
node_146: feature_name=a-MoIgG_0.10	feature_id[27].value > threshold=3760.5
node_156: feature_name=a-HuIgG_0.03	feature_id[7].value > threshold=9740.8330078125
node_158: feature_name=a-HuIgG_0.30	feature_id[4].value <= threshold=42960.0
Class: healthcare workers within 60 days after vaccination	
Rules_16	passed counts:16
node_0: feature_name=HuIgG_0.10	feature_id[2].value <= threshold=19120.166015625
node_1: feature_name=SARS.CoV.2.S1.RBD.mFc	feature_id[28].value > threshold=3773.8334350585938
node_9: feature_name=HuIgM_0.03	feature_id[14].value > threshold=-88.33333206176758
node_27: feature_name=hCoV.HKU1.S1_S2	feature_id[32].value <= threshold=42767.0
node_28: feature_name=HuIgG_0.03	feature_id[0].value <= threshold=1005.0
node_29: feature_name=SARS.CoV.2.S1.mFcTag	feature_id[21].value <= threshold=34194.916015625
node_30: feature_name=SARS.CoV.2.S2	feature_id[29].value <= threshold=7509.916748046875
Class: healthcare workers over 180 days after vaccination	
Rules_17	passed counts:15
node_0: feature_name=HuIgG_0.10	feature_id[2].value > threshold=19120.166015625
node_96: feature_name=a-HuIgG_0.03	feature_id[7].value <= threshold=19665.666015625

node_97: feature_name=MERS.CoV.S1.RBD.367.606.rFcTag	feature_id[5].value > threshold=3252.625
node_201: feature_name=SARS.CoV.2.S2	feature_id[29].value > threshold=37167.458984375
node_301: feature_name=SARS.CoV.2.S1.mFcTag	feature_id[21].value > threshold=53322.333984375
node_321: feature_name=a-MoIgA_0.03	feature_id[24].value <= threshold=212.0
node_322: feature_name=Flu.H3N2.HA1	feature_id[25].value > threshold=37503.517578125
node_330: feature_name=a-MoIgG_0.03	feature_id[12].value > threshold=556.6666564941406
node_344: feature_name=a-HuIgG_0.10	feature_id[3].value > threshold=30472.333984375
Class: healthcare workers within 60 days after vaccination	
Rules_18	passed counts:15
node_0: feature_name=HuIgG_0.10	feature_id[2].value > threshold=19120.166015625
node_96: feature_name=a-HuIgG_0.03	feature_id[7].value <= threshold=19665.666015625
node_97: feature_name=MERS.CoV.S1.RBD.367.606.rFcTag	feature_id[5].value > threshold=3252.625
node_201: feature_name=SARS.CoV.2.S2	feature_id[29].value > threshold=37167.458984375
node_301: feature_name=SARS.CoV.2.S1.mFcTag	feature_id[21].value > threshold=53322.333984375
node_321: feature_name=a-MoIgA_0.03	feature_id[24].value <= threshold=212.0
node_322: feature_name=Flu.H3N2.HA1	feature_id[25].value <= threshold=37503.517578125
node_323: feature_name=hCoV.229E.S1_S2	feature_id[35].value > threshold=29288.533203125
Class: healthcare workers within 60 days after vaccination	
Rules_19	passed counts:15
node_0: feature_name=HuIgG_0.10	feature_id[2].value > threshold=19120.166015625
node_96: feature_name=a-HuIgG_0.03	feature_id[7].value <= threshold=19665.666015625
node_97: feature_name=MERS.CoV.S1.RBD.367.606.rFcTag	feature_id[5].value > threshold=3252.625
node_201: feature_name=SARS.CoV.2.S2	feature_id[29].value <= threshold=37167.458984375

node_202: feature_name=SARS.CoV.2.Spike.RBD.His.Bac	feature_id[1].value > threshold=16030.75048828125
node_212: feature_name=a-MoIgA_0.30	feature_id[37].value > threshold=1303.0
node_224: feature_name=HuIgA_0.03	feature_id[26].value > threshold=25.83333396911621
node_276: feature_name=hCoV.OC43.NP	feature_id[23].value > threshold=3591.10009765625
node_290: feature_name=SARS.CoV.2.Spike.RBD.His.Bac	feature_id[1].value <= threshold=33649.125
node_291: feature_name=SARS.CoV.2.S1.mFcTag	feature_id[21].value > threshold=49171.599609375
node_293: feature_name=a-MoIgA_0.03	feature_id[24].value > threshold=-139.66666412353516
Class: healthcare workers within 60 days after vaccination	
Rules_20	passed counts:15
node_0: feature_name=HuIgG_0.10	feature_id[2].value > threshold=19120.166015625
node_96: feature_name=a-HuIgG_0.03	feature_id[7].value <= threshold=19665.666015625
node_97: feature_name=MERS.CoV.S1.RBD.367.606.rFcTag	feature_id[5].value > threshold=3252.625
node_201: feature_name=SARS.CoV.2.S2	feature_id[29].value <= threshold=37167.458984375
node_202: feature_name=SARS.CoV.2.Spike.RBD.His.Bac	feature_id[1].value > threshold=16030.75048828125
node_212: feature_name=a-MoIgA_0.30	feature_id[37].value > threshold=1303.0
node_224: feature_name=HuIgA_0.03	feature_id[26].value > threshold=25.83333396911621
node_276: feature_name=hCoV.OC43.NP	feature_id[23].value <= threshold=3591.10009765625
node_277: feature_name=hCoV.229E.S1	feature_id[30].value <= threshold=28616.0
node_278: feature_name=a-MoIgA_0.03	feature_id[24].value > threshold=-30.66666603088379
node_282: feature_name=HuIgA_0.03	feature_id[26].value <= threshold=965.3333435058594
node_283: feature_name=HuIgG_0.03	feature_id[0].value <= threshold=7378.66650390625
Class: healthcare workers between 60 and 180 days after vaccination	
Rules_21	passed counts:15

node_0: feature_name=HuIgG_0.10	feature_id[2].value > threshold=19120.166015625
node_96: feature_name=a-HuIgG_0.03	feature_id[7].value <= threshold=19665.666015625
node_97: feature_name=MERS.CoV.S1.RBD.367.606.rFcTag	feature_id[5].value > threshold=3252.625
node_201: feature_name=SARS.CoV.2.S2	feature_id[29].value <= threshold=37167.458984375
node_202: feature_name=SARS.CoV.2.Spike.RBD.His.Bac	feature_id[1].value > threshold=16030.75048828125
node_212: feature_name=a-MoIgA_0.30	feature_id[37].value > threshold=1303.0
node_224: feature_name=HuIgA_0.03	feature_id[26].value <= threshold=25.83333396911621
node_225: feature_name=Flu.B_Phu/.HA1+HA2	feature_id[19].value <= threshold=54802.5
node_226: feature_name=hCoV.HKU1.NP	feature_id[11].value <= threshold=13460.3330078125
node_227: feature_name=MoIgG_0.30	feature_id[34].value > threshold=3760.5
node_229: feature_name=HuIgM_0.10	feature_id[6].value > threshold=-87.16666412353516
node_233: feature_name=a-HuIgG_0.10	feature_id[3].value > threshold=27244.3330078125
node_235: feature_name=SARS.CoV.S1.RBD.rFcTag	feature_id[16].value <= threshold=20123.43359375
node_236: feature_name=MERS.CoV.S1.RBD.367.606.rFcTag	feature_id[5].value > threshold=4326.625
node_238: feature_name=SARS.CoV.NP	feature_id[31].value > threshold=2842.375
node_240: feature_name=Flu.H1N1.HA1+HA2	feature_id[10].value <= threshold=53053.666015625
node_241: feature_name=MoIgG_0.03	feature_id[20].value > threshold=-68.16666793823242
Class: healthcare workers between 60 and 180 days after vaccination	
Rules_22	passed counts:15
node_0: feature_name=HuIgG_0.10	feature_id[2].value > threshold=19120.166015625
node_96: feature_name=a-HuIgG_0.03	feature_id[7].value <= threshold=19665.666015625
node_97: feature_name=MERS.CoV.S1.RBD.367.606.rFcTag	feature_id[5].value > threshold=3252.625
node_201: feature_name=SARS.CoV.2.S2	feature_id[29].value <= threshold=37167.458984375
node_202: feature_name=SARS.CoV.2.Spike.RBD.His.Bac	feature_id[1].value <= threshold=16030.75048828125

node_203: feature_name=a-MoIgG_0.10	feature_id[27].value > threshold=2117.9999389648438
node_207: feature_name=SARS.CoV.S1.HisTag	feature_id[18].value > threshold=1072.50830078125
Class: healthcare workers within 60 days after vaccination	
Rules_23	passed counts:14
node_0: feature_name=HuIgG_0.10	feature_id[2].value > threshold=19120.166015625
node_96: feature_name=a-HuIgG_0.03	feature_id[7].value <= threshold=19665.666015625
node_97: feature_name=MERS.CoV.S1.RBD.367.606.rFcTag	feature_id[5].value > threshold=3252.625
node_201: feature_name=SARS.CoV.2.S2	feature_id[29].value <= threshold=37167.458984375
node_202: feature_name=SARS.CoV.2.Spike.RBD.His.Bac	feature_id[1].value > threshold=16030.75048828125
node_212: feature_name=a-MoIgA_0.30	feature_id[37].value > threshold=1303.0
node_224: feature_name=HuIgA_0.03	feature_id[26].value <= threshold=25.83333396911621
node_225: feature_name=Flu.B_Phu/.HA1+HA2	feature_id[19].value <= threshold=54802.5
node_226: feature_name=hCoV.HKU1.NP	feature_id[11].value <= threshold=13460.3330078125
node_227: feature_name=MoIgG_0.30	feature_id[34].value > threshold=3760.5
node_229: feature_name=HuIgM_0.10	feature_id[6].value > threshold=-87.16666412353516
node_233: feature_name=a-HuIgG_0.10	feature_id[3].value > threshold=27244.3330078125
node_235: feature_name=SARS.CoV.S1.RBD.rFcTag	feature_id[16].value > threshold=20123.43359375
node_245: feature_name=hCoV.OC43.NP	feature_id[23].value <= threshold=1775.816650390625
node_246: feature_name=HuIgA_0.03	feature_id[26].value <= threshold=-10.333333492279053
node_247: feature_name=SARS.CoV.2.S1.RBD.mFc	feature_id[28].value <= threshold=53114.833984375
Class: healthcare workers within 60 days after vaccination	
Rules_24	passed counts:14
node_0: feature_name=HuIgG_0.10	feature_id[2].value > threshold=19120.166015625

node_96: feature_name=a-HuIgG_0.03	feature_id[7].value <= threshold=19665.666015625
node_97: feature_name=MERS.CoV.S1.RBD.367.606.rFcTag	feature_id[5].value > threshold=3252.625
node_201: feature_name=SARS.CoV.2.S2	feature_id[29].value <= threshold=37167.458984375
node_202: feature_name=SARS.CoV.2.Spike.RBD.His.Bac	feature_id[1].value > threshold=16030.75048828125
node_212: feature_name=a-MoIgA_0.30	feature_id[37].value > threshold=1303.0
node_224: feature_name=HuIgA_0.03	feature_id[26].value <= threshold=25.83333396911621
node_225: feature_name=Flu.B_Phu/.HA1+HA2	feature_id[19].value <= threshold=54802.5
node_226: feature_name=hCoV.HKU1.NP	feature_id[11].value <= threshold=13460.3330078125
node_227: feature_name=MoIgG_0.30	feature_id[34].value > threshold=3760.5
node_229: feature_name=HuIgM_0.10	feature_id[6].value > threshold=-87.16666412353516
node_233: feature_name=a-HuIgG_0.10	feature_id[3].value > threshold=27244.3330078125
node_235: feature_name=SARS.CoV.S1.RBD.rFcTag	feature_id[16].value <= threshold=20123.43359375
node_236: feature_name=MERS.CoV.S1.RBD.367.606.rFcTag	feature_id[5].value <= threshold=4326.625
Class: healthcare workers between 60 and 180 days after vaccination	
Rules_25	passed counts:13
node_0: feature_name=HuIgG_0.10	feature_id[2].value <= threshold=19120.166015625
node_1: feature_name=SARS.CoV.2.S1.RBD.mFc	feature_id[28].value > threshold=3773.8334350585938
node_9: feature_name=HuIgM_0.03	feature_id[14].value > threshold=-88.33333206176758
node_27: feature_name=hCoV.HKU1.S1_S2	feature_id[32].value > threshold=42767.0
node_77: feature_name=Flu.B_Mal/.HA1	feature_id[9].value > threshold=41191.69921875
node_91: feature_name=hCoV.NL63.S1_S2	feature_id[13].value <= threshold=33974.650390625
Class: healthcare workers over 180 days after vaccination	
Rules_26	passed counts:13

node_0: feature_name=HuIgG_0.10	feature_id[2].value <= threshold=19120.166015625
node_1: feature_name=SARS.CoV.2.S1.RBD.mFc	feature_id[28].value > threshold=3773.8334350585938
node_9: feature_name=HuIgM_0.03	feature_id[14].value > threshold=-88.33333206176758
node_27: feature_name=hCoV.HKU1.S1_S2	feature_id[32].value > threshold=42767.0
node_77: feature_name=Flu.B_Mal/.HA1	feature_id[9].value <= threshold=41191.69921875
node_78: feature_name=SARS.CoV.2.S1.mFcTag	feature_id[21].value > threshold=41709.720703125
node_82: feature_name=HuIgM_0.10	feature_id[6].value > threshold=-43.83333396911621
node_86: feature_name=HuIgG_0.10	feature_id[2].value <= threshold=18966.0
node_87: feature_name=SARS.CoV.2.S2	feature_id[29].value > threshold=22915.0
Class: healthcare workers within 60 days after vaccination	
Rules_27	passed counts:11
node_0: feature_name=HuIgG_0.10	feature_id[2].value > threshold=19120.166015625
node_96: feature_name=a-HuIgG_0.03	feature_id[7].value <= threshold=19665.666015625
node_97: feature_name=MERS.CoV.S1.RBD.367.606.rFcTag	feature_id[5].value <= threshold=3252.625
node_98: feature_name=SARS.CoV.2.S2	feature_id[29].value > threshold=9506.32470703125
node_116: feature_name=Flu.H1N1.HA1+HA2	feature_id[10].value <= threshold=54065.41796875
node_117: feature_name=SARS.CoV.S1.RBD.rFcTag	feature_id[16].value <= threshold=24639.0
node_118: feature_name=SARS.CoV.2.Spike.RBD.His.Bac	feature_id[1].value > threshold=23841.4169921875
node_128: feature_name=SARS.CoV.2.S1+S2	feature_id[17].value <= threshold=41778.25
node_129: feature_name=SARS.CoV.S1.HisTag	feature_id[18].value <= threshold=3569.2833251953125
node_130: feature_name=SARS.CoV.2.S2	feature_id[29].value <= threshold=26478.916015625
Class: healthcare workers between 60 and 180 days after vaccination	
Rules_28	passed counts:10

node_0: feature_name=HuIgG_0.10	feature_id[2].value > threshold=19120.166015625
node_96: feature_name=a-HuIgG_0.03	feature_id[7].value <= threshold=19665.666015625
node_97: feature_name=MERS.CoV.S1.RBD.367.606.rFcTag	feature_id[5].value > threshold=3252.625
node_201: feature_name=SARS.CoV.2.S2	feature_id[29].value <= threshold=37167.458984375
node_202: feature_name=SARS.CoV.2.Spike.RBD.His.Bac	feature_id[1].value > threshold=16030.75048828125
node_212: feature_name=a-MoIgA_0.30	feature_id[37].value > threshold=1303.0
node_224: feature_name=HuIgA_0.03	feature_id[26].value > threshold=25.83333396911621
node_276: feature_name=hCoV.OC43.NP	feature_id[23].value > threshold=3591.10009765625
node_290: feature_name=SARS.CoV.2.Spike.RBD.His.Bac	feature_id[1].value > threshold=33649.125
node_296: feature_name=HuIgM_0.03	feature_id[14].value > threshold=-90.5
Class: healthcare workers between 60 and 180 days after vaccination	
Rules_29	passed counts:10
node_0: feature_name=HuIgG_0.10	feature_id[2].value > threshold=19120.166015625
node_96: feature_name=a-HuIgG_0.03	feature_id[7].value <= threshold=19665.666015625
node_97: feature_name=MERS.CoV.S1.RBD.367.606.rFcTag	feature_id[5].value > threshold=3252.625
node_201: feature_name=SARS.CoV.2.S2	feature_id[29].value <= threshold=37167.458984375
node_202: feature_name=SARS.CoV.2.Spike.RBD.His.Bac	feature_id[1].value > threshold=16030.75048828125
node_212: feature_name=a-MoIgA_0.30	feature_id[37].value > threshold=1303.0
node_224: feature_name=HuIgA_0.03	feature_id[26].value <= threshold=25.83333396911621
node_225: feature_name=Flu.B_Phu/.HA1+HA2	feature_id[19].value > threshold=54802.5
node_273: feature_name=hCoV.HKU1.S1_AA1.760	feature_id[39].value <= threshold=45921.66796875
Class: healthcare workers between 60 and 180 days after vaccination	
Rules_30	passed counts:10

node_0: feature_name=HuIgG_0.10	feature_id[2].value > threshold=19120.166015625
node_96: feature_name=a-HuIgG_0.03	feature_id[7].value <= threshold=19665.666015625
node_97: feature_name=MERS.CoV.S1.RBD.367.606.rFcTag	feature_id[5].value > threshold=3252.625
node_201: feature_name=SARS.CoV.2.S2	feature_id[29].value <= threshold=37167.458984375
node_202: feature_name=SARS.CoV.2.Spike.RBD.His.Bac	feature_id[1].value > threshold=16030.75048828125
node_212: feature_name=a-MoIgA_0.30	feature_id[37].value > threshold=1303.0
node_224: feature_name=HuIgA_0.03	feature_id[26].value <= threshold=25.83333396911621
node_225: feature_name=Flu.B_Phu/.HA1+HA2	feature_id[19].value <= threshold=54802.5
node_226: feature_name=hCoV.HKU1.NP	feature_id[11].value > threshold=13460.3330078125
node_270: feature_name=Flu.H3N2.HA1	feature_id[25].value > threshold=35417.583984375
Class: healthcare workers within 60 days after vaccination	
Rules_31	passed counts:9
node_0: feature_name=HuIgG_0.10	feature_id[2].value > threshold=19120.166015625
node_96: feature_name=a-HuIgG_0.03	feature_id[7].value <= threshold=19665.666015625
node_97: feature_name=MERS.CoV.S1.RBD.367.606.rFcTag	feature_id[5].value <= threshold=3252.625
node_98: feature_name=SARS.CoV.2.S2	feature_id[29].value <= threshold=9506.32470703125
node_99: feature_name=SARS.CoV.2.S1.RBD.mFc	feature_id[28].value <= threshold=4672.199844360352
node_100: feature_name=a-MoIgA_0.03	feature_id[24].value > threshold=14.999999523162842
node_106: feature_name=HuIgM_0.10	feature_id[6].value <= threshold=65.66666793823242
Class: unvaccinated healthcare workers	
Rules_32	passed counts:8
node_0: feature_name=HuIgG_0.10	feature_id[2].value > threshold=19120.166015625
node_96: feature_name=a-HuIgG_0.03	feature_id[7].value > threshold=19665.666015625

node_350: feature_name=HuIgM_0.03	feature_id[14].value <= threshold=-189.0
node_351: feature_name=hCoV.OC43.HE	feature_id[33].value > threshold=15191.9169921875
Class: healthcare workers between 60 and 180 days after vaccination	
Rules_33	passed counts:8
node_0: feature_name=HuIgG_0.10	feature_id[2].value > threshold=19120.166015625
node_96: feature_name=a-HuIgG_0.03	feature_id[7].value <= threshold=19665.666015625
node_97: feature_name=MERS.CoV.S1.RBD.367.606.rFcTag	feature_id[5].value <= threshold=3252.625
node_98: feature_name=SARS.CoV.2.S2	feature_id[29].value > threshold=9506.32470703125
node_116: feature_name=Flu.H1N1.HA1+HA2	feature_id[10].value > threshold=54065.41796875
node_194: feature_name=MERS.CoV.S1.RBD.367.606.rFcTag	feature_id[5].value > threshold=2551.3333740234375
node_198: feature_name=HuIgA_0.03	feature_id[26].value <= threshold=884.3333129882812
Class: healthcare workers between 60 and 180 days after vaccination	
Rules_34	passed counts:8
node_0: feature_name=HuIgG_0.10	feature_id[2].value > threshold=19120.166015625
node_96: feature_name=a-HuIgG_0.03	feature_id[7].value <= threshold=19665.666015625
node_97: feature_name=MERS.CoV.S1.RBD.367.606.rFcTag	feature_id[5].value <= threshold=3252.625
node_98: feature_name=SARS.CoV.2.S2	feature_id[29].value > threshold=9506.32470703125
node_116: feature_name=Flu.H1N1.HA1+HA2	feature_id[10].value <= threshold=54065.41796875
node_117: feature_name=SARS.CoV.S1.RBD.rFcTag	feature_id[16].value <= threshold=24639.0
node_118: feature_name=SARS.CoV.2.Spike.RBD.His.Bac	feature_id[1].value > threshold=23841.4169921875
node_128: feature_name=SARS.CoV.2.S1+S2	feature_id[17].value > threshold=41778.25
node_138: feature_name=hCoV.NL63.S1_S2	feature_id[13].value > threshold=35816.083984375
node_146: feature_name=a-MoIgG_0.10	feature_id[27].value > threshold=3760.5

node_156: feature_name=a-HuIgG_0.03	feature_id[7].value > threshold=9740.8330078125
node_158: feature_name=a-HuIgG_0.30	feature_id[4].value > threshold=42960.0
node_160: feature_name=hCoV.HKU1.NP	feature_id[11].value > threshold=3927.666748046875
node_162: feature_name=HuIgA_0.03	feature_id[26].value <= threshold=595.6666870117188
Class: healthcare workers within 60 days after vaccination	
Rules_35	passed counts:8
node_0: feature_name=HuIgG_0.10	feature_id[2].value > threshold=19120.166015625
node_96: feature_name=a-HuIgG_0.03	feature_id[7].value <= threshold=19665.666015625
node_97: feature_name=MERS.CoV.S1.RBD.367.606.rFcTag	feature_id[5].value <= threshold=3252.625
node_98: feature_name=SARS.CoV.2.S2	feature_id[29].value > threshold=9506.32470703125
node_116: feature_name=Flu.H1N1.HA1+HA2	feature_id[10].value <= threshold=54065.41796875
node_117: feature_name=SARS.CoV.S1.RBD.rFcTag	feature_id[16].value <= threshold=24639.0
node_118: feature_name=SARS.CoV.2.Spike.RBD.His.Bac	feature_id[1].value > threshold=23841.4169921875
node_128: feature_name=SARS.CoV.2.S1+S2	feature_id[17].value > threshold=41778.25
node_138: feature_name=hCoV.NL63.S1_S2	feature_id[13].value > threshold=35816.083984375
node_146: feature_name=a-MoIgG_0.10	feature_id[27].value <= threshold=3760.5
node_147: feature_name=SARS.CoV.S1.HisTag	feature_id[18].value <= threshold=3759.5833740234375
Class: healthcare workers between 60 and 180 days after vaccination	
Rules_36	passed counts:8
node_0: feature_name=HuIgG_0.10	feature_id[2].value <= threshold=19120.166015625
node_1: feature_name=SARS.CoV.2.S1.RBD.mFc	feature_id[28].value > threshold=3773.8334350585938
node_9: feature_name=HuIgM_0.03	feature_id[14].value > threshold=-88.33333206176758
node_27: feature_name=hCoV.HKU1.S1_S2	feature_id[32].value <= threshold=42767.0

node_28: feature_name=HuIgG_0.03	feature_id[0].value > threshold=1005.0
node_38: feature_name=a-HuIgG_0.30	feature_id[4].value <= threshold=33596.333984375
node_39: feature_name=SARS.CoV.S1.HisTag	feature_id[18].value > threshold=400.3000030517578
node_43: feature_name=HuIgM_0.10	feature_id[6].value <= threshold=109.66666793823242
node_44: feature_name=MERS.CoV.NP	feature_id[15].value > threshold=-50.125
node_46: feature_name=hCoV.229E.S1_S2	feature_id[35].value <= threshold=42044.208984375
node_47: feature_name=HuIgM_0.30	feature_id[8].value <= threshold=1223.5
node_48: feature_name=SARS.CoV.S1.RBD.rFcTag	feature_id[16].value > threshold=27979.533203125
node_50: feature_name=Flu.H3N2.HA1+HA2	feature_id[38].value > threshold=39487.333984375
Class: healthcare workers between 60 and 180 days after vaccination	
Rules_37	passed counts:8
node_0: feature_name=HuIgG_0.10	feature_id[2].value <= threshold=19120.166015625
node_1: feature_name=SARS.CoV.2.S1.RBD.mFc	feature_id[28].value > threshold=3773.8334350585938
node_9: feature_name=HuIgM_0.03	feature_id[14].value <= threshold=-88.33333206176758
node_10: feature_name=HuIgA_0.03	feature_id[26].value <= threshold=304.5
node_11: feature_name=Flu.H1N1.HA1+HA2	feature_id[10].value > threshold=53444.666015625
node_19: feature_name=a-MoIgA_0.03	feature_id[24].value > threshold=-97.16666793823242
node_21: feature_name=a-MoIgA_0.03	feature_id[24].value <= threshold=0.6666666269302368
Class: healthcare workers over 180 days after vaccination	
Rules_38	passed counts:7
node_0: feature_name=HuIgG_0.10	feature_id[2].value > threshold=19120.166015625
node_96: feature_name=a-HuIgG_0.03	feature_id[7].value <= threshold=19665.666015625
node_97: feature_name=MERS.CoV.S1.RBD.367.606.rFcTag	feature_id[5].value > threshold=3252.625

node_201: feature_name=SARS.CoV.2.S2	feature_id[29].value > threshold=37167.458984375
node_301: feature_name=SARS.CoV.2.S1.mFcTag	feature_id[21].value > threshold=53322.333984375
node_321: feature_name=a-MoIgA_0.03	feature_id[24].value > threshold=212.0
Class: healthcare workers between 60 and 180 days after vaccination	
Rules_39	passed counts:7
node_0: feature_name=HuIgG_0.10	feature_id[2].value > threshold=19120.166015625
node_96: feature_name=a-HuIgG_0.03	feature_id[7].value <= threshold=19665.666015625
node_97: feature_name=MERS.CoV.S1.RBD.367.606.rFcTag	feature_id[5].value > threshold=3252.625
node_201: feature_name=SARS.CoV.2.S2	feature_id[29].value <= threshold=37167.458984375
node_202: feature_name=SARS.CoV.2.Spike.RBD.His.Bac	feature_id[1].value > threshold=16030.75048828125
node_212: feature_name=a-MoIgA_0.30	feature_id[37].value > threshold=1303.0
node_224: feature_name=HuIgA_0.03	feature_id[26].value > threshold=25.83333396911621
node_276: feature_name=hCoV.OC43.NP	feature_id[23].value <= threshold=3591.10009765625
node_277: feature_name=hCoV.229E.S1	feature_id[30].value <= threshold=28616.0
node_278: feature_name=a-MoIgA_0.03	feature_id[24].value <= threshold=-30.66666603088379
node_279: feature_name=MERS.CoV.NP	feature_id[15].value <= threshold=8498.916748046875
Class: healthcare workers within 60 days after vaccination	
Rules_40	passed counts:7
node_0: feature_name=HuIgG_0.10	feature_id[2].value > threshold=19120.166015625
node_96: feature_name=a-HuIgG_0.03	feature_id[7].value <= threshold=19665.666015625
node_97: feature_name=MERS.CoV.S1.RBD.367.606.rFcTag	feature_id[5].value > threshold=3252.625
node_201: feature_name=SARS.CoV.2.S2	feature_id[29].value <= threshold=37167.458984375
node_202: feature_name=SARS.CoV.2.Spike.RBD.His.Bac	feature_id[1].value > threshold=16030.75048828125

node_212: feature_name=a-MoIgA_0.30	feature_id[37].value > threshold=1303.0
node_224: feature_name=HuIgA_0.03	feature_id[26].value <= threshold=25.83333396911621
node_225: feature_name=Flu.B_Phu/.HA1+HA2	feature_id[19].value <= threshold=54802.5
node_226: feature_name=hCoV.HKU1.NP	feature_id[11].value <= threshold=13460.3330078125
node_227: feature_name=MoIgG_0.30	feature_id[34].value <= threshold=3760.5
Class: healthcare workers within 60 days after vaccination	
Rules_41	passed counts:7
node_0: feature_name=HuIgG_0.10	feature_id[2].value > threshold=19120.166015625
node_96: feature_name=a-HuIgG_0.03	feature_id[7].value <= threshold=19665.666015625
node_97: feature_name=MERS.CoV.S1.RBD.367.606.rFcTag	feature_id[5].value <= threshold=3252.625
node_98: feature_name=SARS.CoV.2.S2	feature_id[29].value > threshold=9506.32470703125
node_116: feature_name=Flu.H1N1.HA1+HA2	feature_id[10].value <= threshold=54065.41796875
node_117: feature_name=SARS.CoV.S1.RBD.rFcTag	feature_id[16].value > threshold=24639.0
node_167: feature_name=hCoV.OC43.HE	feature_id[33].value <= threshold=32466.5830078125
node_168: feature_name=HuIgM_0.10	feature_id[6].value > threshold=22.833333015441895
node_178: feature_name=a-MoIgA_0.03	feature_id[24].value <= threshold=-34.333333015441895
node_179: feature_name=Flu.H3N2.HA1+HA2	feature_id[38].value <= threshold=54266.91796875
node_180: feature_name=a-HuIgG_0.03	feature_id[7].value > threshold=11043.33349609375
Class: healthcare workers between 60 and 180 days after vaccination	
Rules_42	passed counts:7
node_0: feature_name=HuIgG_0.10	feature_id[2].value > threshold=19120.166015625
node_96: feature_name=a-HuIgG_0.03	feature_id[7].value <= threshold=19665.666015625
node_97: feature_name=MERS.CoV.S1.RBD.367.606.rFcTag	feature_id[5].value <= threshold=3252.625

node_98: feature_name=SARS.CoV.2.S2	feature_id[29].value <= threshold=9506.32470703125
node_99: feature_name=SARS.CoV.2.S1.RBD.mFc	feature_id[28].value > threshold=4672.199844360352
node_111: feature_name=HuIgM_0.10	feature_id[6].value > threshold=-30.333333015441895
Class: healthcare workers between 60 and 180 days after vaccination	
Rules_43	passed counts:7
node_0: feature_name=HuIgG_0.10	feature_id[2].value <= threshold=19120.166015625
node_1: feature_name=SARS.CoV.2.S1.RBD.mFc	feature_id[28].value > threshold=3773.8334350585938
node_9: feature_name=HuIgM_0.03	feature_id[14].value > threshold=-88.33333206176758
node_27: feature_name=hCoV.HKU1.S1_S2	feature_id[32].value <= threshold=42767.0
node_28: feature_name=HuIgG_0.03	feature_id[0].value > threshold=1005.0
node_38: feature_name=a-HuIgG_0.30	feature_id[4].value > threshold=33596.333984375
node_60: feature_name=a-HuIgG_0.03	feature_id[7].value <= threshold=10366.5
node_61: feature_name=Flu.H3N2.HA1	feature_id[25].value > threshold=29968.0830078125
node_65: feature_name=HuIgG_0.30	feature_id[36].value <= threshold=36026.0
Class: healthcare workers within 60 days after vaccination	
Rules_44	passed counts:7
node_0: feature_name=HuIgG_0.10	feature_id[2].value <= threshold=19120.166015625
node_1: feature_name=SARS.CoV.2.S1.RBD.mFc	feature_id[28].value > threshold=3773.8334350585938
node_9: feature_name=HuIgM_0.03	feature_id[14].value > threshold=-88.33333206176758
node_27: feature_name=hCoV.HKU1.S1_S2	feature_id[32].value <= threshold=42767.0
node_28: feature_name=HuIgG_0.03	feature_id[0].value <= threshold=1005.0
node_29: feature_name=SARS.CoV.2.S1.mFcTag	feature_id[21].value > threshold=34194.916015625
node_35: feature_name=a-HuIgG_0.10	feature_id[3].value <= threshold=15372.16650390625

Class: healthcare workers between 60 and 180 days after vaccination	
Rules_45	passed counts:7
node_0: feature_name=HuIgG_0.10	feature_id[2].value <= threshold=19120.166015625
node_1: feature_name=SARS.CoV.2.S1.RBD.mFc	feature_id[28].value > threshold=3773.8334350585938
node_9: feature_name=HuIgM_0.03	feature_id[14].value <= threshold=-88.33333206176758
node_10: feature_name=HuIgA_0.03	feature_id[26].value <= threshold=304.5
node_11: feature_name=Flu.H1N1.HA1+HA2	feature_id[10].value > threshold=53444.666015625
node_19: feature_name=a-MoIgA_0.03	feature_id[24].value <= threshold=-97.16666793823242
Class: healthcare workers between 60 and 180 days after vaccination	
Rules_46	passed counts:6
node_0: feature_name=HuIgG_0.10	feature_id[2].value > threshold=19120.166015625
node_96: feature_name=a-HuIgG_0.03	feature_id[7].value <= threshold=19665.666015625
node_97: feature_name=MERS.CoV.S1.RBD.367.606.rFcTag	feature_id[5].value > threshold=3252.625
node_201: feature_name=SARS.CoV.2.S2	feature_id[29].value <= threshold=37167.458984375
node_202: feature_name=SARS.CoV.2.Spike.RBD.His.Bac	feature_id[1].value > threshold=16030.75048828125
node_212: feature_name=a-MoIgA_0.30	feature_id[37].value > threshold=1303.0
node_224: feature_name=HuIgA_0.03	feature_id[26].value <= threshold=25.83333396911621
node_225: feature_name=Flu.B_Phu/.HA1+HA2	feature_id[19].value <= threshold=54802.5
node_226: feature_name=hCoV.HKU1.NP	feature_id[11].value <= threshold=13460.3330078125
node_227: feature_name=MoIgG_0.30	feature_id[34].value > threshold=3760.5
node_229: feature_name=HuIgM_0.10	feature_id[6].value <= threshold=-87.16666412353516
node_230: feature_name=hCoV.229E.S1	feature_id[30].value <= threshold=39693.333984375
Class: healthcare workers within 60 days after vaccination	

Rules_47	passed counts:6
node_0: feature_name=HuIgG_0.10	feature_id[2].value > threshold=19120.166015625
node_96: feature_name=a-HuIgG_0.03	feature_id[7].value <= threshold=19665.666015625
node_97: feature_name=MERS.CoV.S1.RBD.367.606.rFcTag	feature_id[5].value <= threshold=3252.625
node_98: feature_name=SARS.CoV.2.S2	feature_id[29].value > threshold=9506.32470703125
node_116: feature_name=Flu.H1N1.HA1+HA2	feature_id[10].value <= threshold=54065.41796875
node_117: feature_name=SARS.CoV.S1.RBD.rFcTag	feature_id[16].value <= threshold=24639.0
node_118: feature_name=SARS.CoV.2.Spike.RBD.His.Bac	feature_id[1].value > threshold=23841.4169921875
node_128: feature_name=SARS.CoV.2.S1+S2	feature_id[17].value > threshold=41778.25
node_138: feature_name=hCoV.NL63.S1_S2	feature_id[13].value > threshold=35816.083984375
node_146: feature_name=a-MoIgG_0.10	feature_id[27].value <= threshold=3760.5
node_147: feature_name=SARS.CoV.S1.HisTag	feature_id[18].value > threshold=3759.5833740234375
node_149: feature_name=SARS.CoV.S1.HisTag	feature_id[18].value > threshold=4886.25
node_151: feature_name=HuIgM_0.30	feature_id[8].value > threshold=518.0000152587891
Class: healthcare workers between 60 and 180 days after vaccination	
Rules_48	passed counts:6
node_0: feature_name=HuIgG_0.10	feature_id[2].value > threshold=19120.166015625
node_96: feature_name=a-HuIgG_0.03	feature_id[7].value <= threshold=19665.666015625
node_97: feature_name=MERS.CoV.S1.RBD.367.606.rFcTag	feature_id[5].value <= threshold=3252.625
node_98: feature_name=SARS.CoV.2.S2	feature_id[29].value > threshold=9506.32470703125
node_116: feature_name=Flu.H1N1.HA1+HA2	feature_id[10].value <= threshold=54065.41796875
node_117: feature_name=SARS.CoV.S1.RBD.rFcTag	feature_id[16].value <= threshold=24639.0
node_118: feature_name=SARS.CoV.2.Spike.RBD.His.Bac	feature_id[1].value > threshold=23841.4169921875

node_128: feature_name=SARS.CoV.2.S1+S2	feature_id[17].value > threshold=41778.25
node_138: feature_name=hCoV.NL63.S1_S2	feature_id[13].value > threshold=35816.083984375
node_146: feature_name=a-MoIgG_0.10	feature_id[27].value <= threshold=3760.5
node_147: feature_name=SARS.CoV.S1.HisTag	feature_id[18].value > threshold=3759.5833740234375
node_149: feature_name=SARS.CoV.S1.HisTag	feature_id[18].value <= threshold=4886.25
Class: healthcare workers within 60 days after vaccination	
Rules_49	passed counts:5
node_0: feature_name=HuIgG_0.10	feature_id[2].value > threshold=19120.166015625
node_96: feature_name=a-HuIgG_0.03	feature_id[7].value > threshold=19665.666015625
node_350: feature_name=HuIgM_0.03	feature_id[14].value > threshold=-189.0
node_354: feature_name=HuIgM_0.30	feature_id[8].value > threshold=441.8333282470703
node_356: feature_name=a-HuIgG_0.10	feature_id[3].value <= threshold=32914.5
node_357: feature_name=MERS.CoV.S1.RBD.367.606.rFcTag	feature_id[5].value <= threshold=6711.89990234375
Class: healthcare workers between 60 and 180 days after vaccination	
Rules_50	passed counts:5
node_0: feature_name=HuIgG_0.10	feature_id[2].value > threshold=19120.166015625
node_96: feature_name=a-HuIgG_0.03	feature_id[7].value <= threshold=19665.666015625
node_97: feature_name=MERS.CoV.S1.RBD.367.606.rFcTag	feature_id[5].value > threshold=3252.625
node_201: feature_name=SARS.CoV.2.S2	feature_id[29].value > threshold=37167.458984375
node_301: feature_name=SARS.CoV.2.S1.mFcTag	feature_id[21].value > threshold=53322.333984375
node_321: feature_name=a-MoIgA_0.03	feature_id[24].value <= threshold=212.0
node_322: feature_name=Flu.H3N2.HA1	feature_id[25].value > threshold=37503.517578125
node_330: feature_name=a-MoIgG_0.03	feature_id[12].value > threshold=556.6666564941406

node_344: feature_name=a-HuIgG_0.10	feature_id[3].value <= threshold=30472.333984375
node_345: feature_name=MoIgG_0.30	feature_id[34].value > threshold=6414.66650390625
Class: healthcare workers between 60 and 180 days after vaccination	
Rules_51	passed counts:5
node_0: feature_name=HuIgG_0.10	feature_id[2].value > threshold=19120.166015625
node_96: feature_name=a-HuIgG_0.03	feature_id[7].value <= threshold=19665.666015625
node_97: feature_name=MERS.CoV.S1.RBD.367.606.rFcTag	feature_id[5].value > threshold=3252.625
node_201: feature_name=SARS.CoV.2.S2	feature_id[29].value > threshold=37167.458984375
node_301: feature_name=SARS.CoV.2.S1.mFcTag	feature_id[21].value > threshold=53322.333984375
node_321: feature_name=a-MoIgA_0.03	feature_id[24].value <= threshold=212.0
node_322: feature_name=Flu.H3N2.HA1	feature_id[25].value > threshold=37503.517578125
node_330: feature_name=a-MoIgG_0.03	feature_id[12].value <= threshold=556.6666564941406
node_331: feature_name=hCoV.229E.S1_S2	feature_id[35].value <= threshold=47263.833984375
node_332: feature_name=a-HuIgG_0.10	feature_id[3].value <= threshold=30393.166015625
node_333: feature_name=hCoV.OC43.HE	feature_id[33].value <= threshold=25792.5830078125
Class: healthcare workers within 60 days after vaccination	
Rules_52	passed counts:5
node_0: feature_name=HuIgG_0.10	feature_id[2].value > threshold=19120.166015625
node_96: feature_name=a-HuIgG_0.03	feature_id[7].value <= threshold=19665.666015625
node_97: feature_name=MERS.CoV.S1.RBD.367.606.rFcTag	feature_id[5].value > threshold=3252.625
node_201: feature_name=SARS.CoV.2.S2	feature_id[29].value <= threshold=37167.458984375
node_202: feature_name=SARS.CoV.2.Spike.RBD.His.Bac	feature_id[1].value > threshold=16030.75048828125
node_212: feature_name=a-MoIgA_0.30	feature_id[37].value > threshold=1303.0

node_224: feature_name=HuIgA_0.03	feature_id[26].value <= threshold=25.83333396911621
node_225: feature_name=Flu.B_Phu/.HA1+HA2	feature_id[19].value <= threshold=54802.5
node_226: feature_name=hCoV.HKU1.NP	feature_id[11].value <= threshold=13460.3330078125
node_227: feature_name=MoIgG_0.30	feature_id[34].value > threshold=3760.5
node_229: feature_name=HuIgM_0.10	feature_id[6].value > threshold=-87.16666412353516
node_233: feature_name=a-HuIgG_0.10	feature_id[3].value > threshold=27244.3330078125
node_235: feature_name=SARS.CoV.S1.RBD.rFcTag	feature_id[16].value <= threshold=20123.43359375
node_236: feature_name=MERS.CoV.S1.RBD.367.606.rFcTag	feature_id[5].value > threshold=4326.625
node_238: feature_name=SARS.CoV.NP	feature_id[31].value <= threshold=2842.375
Class: healthcare workers within 60 days after vaccination	
Rules_53	passed counts:5
node_0: feature_name=HuIgG_0.10	feature_id[2].value > threshold=19120.166015625
node_96: feature_name=a-HuIgG_0.03	feature_id[7].value <= threshold=19665.666015625
node_97: feature_name=MERS.CoV.S1.RBD.367.606.rFcTag	feature_id[5].value > threshold=3252.625
node_201: feature_name=SARS.CoV.2.S2	feature_id[29].value <= threshold=37167.458984375
node_202: feature_name=SARS.CoV.2.Spike.RBD.His.Bac	feature_id[1].value > threshold=16030.75048828125
node_212: feature_name=a-MoIgA_0.30	feature_id[37].value > threshold=1303.0
node_224: feature_name=HuIgA_0.03	feature_id[26].value <= threshold=25.83333396911621
node_225: feature_name=Flu.B_Phu/.HA1+HA2	feature_id[19].value <= threshold=54802.5
node_226: feature_name=hCoV.HKU1.NP	feature_id[11].value <= threshold=13460.3330078125
node_227: feature_name=MoIgG_0.30	feature_id[34].value > threshold=3760.5
node_229: feature_name=HuIgM_0.10	feature_id[6].value > threshold=-87.16666412353516
node_233: feature_name=a-HuIgG_0.10	feature_id[3].value <= threshold=27244.3330078125
Class: healthcare workers within 60 days after vaccination	

Rules_54	passed counts:5
node_0: feature_name=HuIgG_0.10	feature_id[2].value > threshold=19120.166015625
node_96: feature_name=a-HuIgG_0.03	feature_id[7].value <= threshold=19665.666015625
node_97: feature_name=MERS.CoV.S1.RBD.367.606.rFcTag	feature_id[5].value <= threshold=3252.625
node_98: feature_name=SARS.CoV.2.S2	feature_id[29].value <= threshold=9506.32470703125
node_99: feature_name=SARS.CoV.2.S1.RBD.mFc	feature_id[28].value <= threshold=4672.199844360352
node_100: feature_name=a-MoIgA_0.03	feature_id[24].value <= threshold=14.999999523162842
node_101: feature_name=a-HuIgG_0.03	feature_id[7].value > threshold=313.1666564941406
Class: healthcare workers within 60 days after vaccination	
Rules_55	passed counts:4
node_0: feature_name=HuIgG_0.10	feature_id[2].value > threshold=19120.166015625
node_96: feature_name=a-HuIgG_0.03	feature_id[7].value > threshold=19665.666015625
node_350: feature_name=HuIgM_0.03	feature_id[14].value > threshold=-189.0
node_354: feature_name=HuIgM_0.30	feature_id[8].value <= threshold=441.8333282470703
Class: healthcare workers between 60 and 180 days after vaccination	
Rules_56	passed counts:4
node_0: feature_name=HuIgG_0.10	feature_id[2].value > threshold=19120.166015625
node_96: feature_name=a-HuIgG_0.03	feature_id[7].value <= threshold=19665.666015625
node_97: feature_name=MERS.CoV.S1.RBD.367.606.rFcTag	feature_id[5].value > threshold=3252.625
node_201: feature_name=SARS.CoV.2.S2	feature_id[29].value > threshold=37167.458984375
node_301: feature_name=SARS.CoV.2.S1.mFcTag	feature_id[21].value > threshold=53322.333984375
node_321: feature_name=a-MoIgA_0.03	feature_id[24].value <= threshold=212.0

node_322: feature_name=Flu.H3N2.HA1	feature_id[25].value > threshold=37503.517578125
node_330: feature_name=a-MoIgG_0.03	feature_id[12].value <= threshold=556.6666564941406
node_331: feature_name=hCoV.229E.S1_S2	feature_id[35].value > threshold=47263.833984375
Class: healthcare workers within 60 days after vaccination	
Rules_57	passed counts:4
node_0: feature_name=HuIgG_0.10	feature_id[2].value > threshold=19120.166015625
node_96: feature_name=a-HuIgG_0.03	feature_id[7].value <= threshold=19665.666015625
node_97: feature_name=MERS.CoV.S1.RBD.367.606.rFcTag	feature_id[5].value > threshold=3252.625
node_201: feature_name=SARS.CoV.2.S2	feature_id[29].value > threshold=37167.458984375
node_301: feature_name=SARS.CoV.2.S1.mFcTag	feature_id[21].value <= threshold=53322.333984375
node_302: feature_name=MoIgG_0.03	feature_id[20].value <= threshold=18.166666507720947
node_303: feature_name=MoIgG_0.30	feature_id[34].value <= threshold=7257.166748046875
node_304: feature_name=hCoV.NL63.S1_S2	feature_id[13].value <= threshold=52607.083984375
node_305: feature_name=HuIgG_0.30	feature_id[36].value <= threshold=35912.0
node_306: feature_name=hCoV.229E.S1	feature_id[30].value <= threshold=31286.6669921875
Class: healthcare workers within 60 days after vaccination	
Rules_58	passed counts:4
node_0: feature_name=HuIgG_0.10	feature_id[2].value > threshold=19120.166015625
node_96: feature_name=a-HuIgG_0.03	feature_id[7].value <= threshold=19665.666015625
node_97: feature_name=MERS.CoV.S1.RBD.367.606.rFcTag	feature_id[5].value > threshold=3252.625
node_201: feature_name=SARS.CoV.2.S2	feature_id[29].value <= threshold=37167.458984375
node_202: feature_name=SARS.CoV.2.Spike.RBD.His.Bac	feature_id[1].value > threshold=16030.75048828125
node_212: feature_name=a-MoIgA_0.30	feature_id[37].value > threshold=1303.0

node_224: feature_name=HuIgA_0.03	feature_id[26].value > threshold=25.83333396911621
node_276: feature_name=hCoV.OC43.NP	feature_id[23].value <= threshold=3591.10009765625
node_277: feature_name=hCoV.229E.S1	feature_id[30].value <= threshold=28616.0
node_278: feature_name=a-MoIgA_0.03	feature_id[24].value > threshold=-30.66666603088379
node_282: feature_name=HuIgA_0.03	feature_id[26].value > threshold=965.3333435058594
Class: healthcare workers within 60 days after vaccination	
Rules_59	passed counts:4
node_0: feature_name=HuIgG_0.10	feature_id[2].value > threshold=19120.166015625
node_96: feature_name=a-HuIgG_0.03	feature_id[7].value <= threshold=19665.666015625
node_97: feature_name=MERS.CoV.S1.RBD.367.606.rFcTag	feature_id[5].value > threshold=3252.625
node_201: feature_name=SARS.CoV.2.S2	feature_id[29].value <= threshold=37167.458984375
node_202: feature_name=SARS.CoV.2.Spike.RBD.His.Bac	feature_id[1].value > threshold=16030.75048828125
node_212: feature_name=a-MoIgA_0.30	feature_id[37].value > threshold=1303.0
node_224: feature_name=HuIgA_0.03	feature_id[26].value <= threshold=25.83333396911621
node_225: feature_name=Flu.B_Phu/.HA1+HA2	feature_id[19].value <= threshold=54802.5
node_226: feature_name=hCoV.HKU1.NP	feature_id[11].value <= threshold=13460.3330078125
node_227: feature_name=MoIgG_0.30	feature_id[34].value > threshold=3760.5
node_229: feature_name=HuIgM_0.10	feature_id[6].value > threshold=-87.16666412353516
node_233: feature_name=a-HuIgG_0.10	feature_id[3].value > threshold=27244.3330078125
node_235: feature_name=SARS.CoV.S1.RBD.rFcTag	feature_id[16].value > threshold=20123.43359375
node_245: feature_name=hCoV.OC43.NP	feature_id[23].value > threshold=1775.816650390625
node_253: feature_name=SARS.CoV.S1.RBD.rFcTag	feature_id[16].value <= threshold=21641.4169921875
Class: healthcare workers within 60 days after vaccination	

Rules_60	passed counts:4
node_0: feature_name=HuIgG_0.10	feature_id[2].value > threshold=19120.166015625
node_96: feature_name=a-HuIgG_0.03	feature_id[7].value <= threshold=19665.666015625
node_97: feature_name=MERS.CoV.S1.RBD.367.606.rFcTag	feature_id[5].value > threshold=3252.625
node_201: feature_name=SARS.CoV.2.S2	feature_id[29].value <= threshold=37167.458984375
node_202: feature_name=SARS.CoV.2.Spike.RBD.His.Bac	feature_id[1].value <= threshold=16030.75048828125
node_203: feature_name=a-MoIgG_0.10	feature_id[27].value <= threshold=2117.9999389648438
node_204: feature_name=Flu.H3N2.HA1+HA2	feature_id[38].value > threshold=44847.720703125
Class: unvaccinated healthcare workers	
Rules_61	passed counts:4
node_0: feature_name=HuIgG_0.10	feature_id[2].value > threshold=19120.166015625
node_96: feature_name=a-HuIgG_0.03	feature_id[7].value <= threshold=19665.666015625
node_97: feature_name=MERS.CoV.S1.RBD.367.606.rFcTag	feature_id[5].value <= threshold=3252.625
node_98: feature_name=SARS.CoV.2.S2	feature_id[29].value > threshold=9506.32470703125
node_116: feature_name=Flu.H1N1.HA1+HA2	feature_id[10].value > threshold=54065.41796875
node_194: feature_name=MERS.CoV.S1.RBD.367.606.rFcTag	feature_id[5].value <= threshold=2551.3333740234375
node_195: feature_name=a-HuIgG_0.03	feature_id[7].value <= threshold=17733.50048828125
Class: healthcare workers within 60 days after vaccination	
Rules_62	passed counts:4
node_0: feature_name=HuIgG_0.10	feature_id[2].value > threshold=19120.166015625
node_96: feature_name=a-HuIgG_0.03	feature_id[7].value <= threshold=19665.666015625
node_97: feature_name=MERS.CoV.S1.RBD.367.606.rFcTag	feature_id[5].value <= threshold=3252.625
node_98: feature_name=SARS.CoV.2.S2	feature_id[29].value > threshold=9506.32470703125

node_116: feature_name=Flu.H1N1.HA1+HA2	feature_id[10].value <= threshold=54065.41796875
node_117: feature_name=SARS.CoV.S1.RBD.rFcTag	feature_id[16].value > threshold=24639.0
node_167: feature_name=hCoV.OC43.HE	feature_id[33].value <= threshold=32466.5830078125
node_168: feature_name=HuIgM_0.10	feature_id[6].value > threshold=22.833333015441895
node_178: feature_name=a-MoIgA_0.03	feature_id[24].value <= threshold=-34.333333015441895
node_179: feature_name=Flu.H3N2.HA1+HA2	feature_id[38].value > threshold=54266.91796875
Class: healthcare workers within 60 days after vaccination	
Rules_63	passed counts:4
node_0: feature_name=HuIgG_0.10	feature_id[2].value > threshold=19120.166015625
node_96: feature_name=a-HuIgG_0.03	feature_id[7].value <= threshold=19665.666015625
node_97: feature_name=MERS.CoV.S1.RBD.367.606.rFcTag	feature_id[5].value <= threshold=3252.625
node_98: feature_name=SARS.CoV.2.S2	feature_id[29].value > threshold=9506.32470703125
node_116: feature_name=Flu.H1N1.HA1+HA2	feature_id[10].value <= threshold=54065.41796875
node_117: feature_name=SARS.CoV.S1.RBD.rFcTag	feature_id[16].value > threshold=24639.0
node_167: feature_name=hCoV.OC43.HE	feature_id[33].value <= threshold=32466.5830078125
node_168: feature_name=HuIgM_0.10	feature_id[6].value <= threshold=22.833333015441895
node_169: feature_name=SARS.CoV.2.S1.mFcTag	feature_id[21].value > threshold=54847.25
node_175: feature_name=SARS.CoV.2.S1.RBD.mFc	feature_id[28].value > threshold=49975.58203125
Class: healthcare workers within 60 days after vaccination	
Rules_64	passed counts:4
node_0: feature_name=HuIgG_0.10	feature_id[2].value > threshold=19120.166015625
node_96: feature_name=a-HuIgG_0.03	feature_id[7].value <= threshold=19665.666015625
node_97: feature_name=MERS.CoV.S1.RBD.367.606.rFcTag	feature_id[5].value <= threshold=3252.625

node_98: feature_name=SARS.CoV.2.S2	feature_id[29].value > threshold=9506.32470703125
node_116: feature_name=Flu.H1N1.HA1+HA2	feature_id[10].value <= threshold=54065.41796875
node_117: feature_name=SARS.CoV.S1.RBD.rFcTag	feature_id[16].value <= threshold=24639.0
node_118: feature_name=SARS.CoV.2.Spike.RBD.His.Bac	feature_id[1].value > threshold=23841.4169921875
node_128: feature_name=SARS.CoV.2.S1+S2	feature_id[17].value <= threshold=41778.25
node_129: feature_name=SARS.CoV.S1.HisTag	feature_id[18].value > threshold=3569.2833251953125
node_133: feature_name=Flu.B_Mal/.HA1+HA2	feature_id[22].value <= threshold=47613.583984375
Class: healthcare workers within 60 days after vaccination	
Rules_65	passed counts:4
node_0: feature_name=HuIgG_0.10	feature_id[2].value > threshold=19120.166015625
node_96: feature_name=a-HuIgG_0.03	feature_id[7].value <= threshold=19665.666015625
node_97: feature_name=MERS.CoV.S1.RBD.367.606.rFcTag	feature_id[5].value <= threshold=3252.625
node_98: feature_name=SARS.CoV.2.S2	feature_id[29].value <= threshold=9506.32470703125
node_99: feature_name=SARS.CoV.2.S1.RBD.mFc	feature_id[28].value > threshold=4672.199844360352
node_111: feature_name=HuIgM_0.10	feature_id[6].value <= threshold=-30.333333015441895
node_112: feature_name=a-MoIgG_0.10	feature_id[27].value <= threshold=2261.1666259765625
Class: healthcare workers over 180 days after vaccination	
Rules_66	passed counts:4
node_0: feature_name=HuIgG_0.10	feature_id[2].value <= threshold=19120.166015625
node_1: feature_name=SARS.CoV.2.S1.RBD.mFc	feature_id[28].value > threshold=3773.8334350585938
node_9: feature_name=HuIgM_0.03	feature_id[14].value > threshold=-88.33333206176758
node_27: feature_name=hCoV.HKU1.S1_S2	feature_id[32].value > threshold=42767.0
node_77: feature_name=Flu.B_Mal/.HA1	feature_id[9].value <= threshold=41191.69921875

node_78: feature_name=SARS.CoV.2.S1.mFcTag	feature_id[21].value > threshold=41709.720703125
node_82: feature_name=HuIgM_0.10	feature_id[6].value <= threshold=-43.83333396911621
node_83: feature_name=SARS.CoV.S1.HisTag	feature_id[18].value > threshold=8532.783203125
Class: healthcare workers between 60 and 180 days after vaccination	
Rules_67	passed counts:4
node_0: feature_name=HuIgG_0.10	feature_id[2].value <= threshold=19120.166015625
node_1: feature_name=SARS.CoV.2.S1.RBD.mFc	feature_id[28].value > threshold=3773.8334350585938
node_9: feature_name=HuIgM_0.03	feature_id[14].value <= threshold=-88.33333206176758
node_10: feature_name=HuIgA_0.03	feature_id[26].value > threshold=304.5
Class: healthcare workers between 60 and 180 days after vaccination	
Rules_68	passed counts:3
node_0: feature_name=HuIgG_0.10	feature_id[2].value > threshold=19120.166015625
node_96: feature_name=a-HuIgG_0.03	feature_id[7].value <= threshold=19665.666015625
node_97: feature_name=MERS.CoV.S1.RBD.367.606.rFcTag	feature_id[5].value > threshold=3252.625
node_201: feature_name=SARS.CoV.2.S2	feature_id[29].value > threshold=37167.458984375
node_301: feature_name=SARS.CoV.2.S1.mFcTag	feature_id[21].value > threshold=53322.333984375
node_321: feature_name=a-MoIgA_0.03	feature_id[24].value <= threshold=212.0
node_322: feature_name=Flu.H3N2.HA1	feature_id[25].value > threshold=37503.517578125
node_330: feature_name=a-MoIgG_0.03	feature_id[12].value > threshold=556.6666564941406
node_344: feature_name=a-HuIgG_0.10	feature_id[3].value <= threshold=30472.333984375
node_345: feature_name=MoIgG_0.30	feature_id[34].value <= threshold=6414.66650390625
Class: healthcare workers within 60 days after vaccination	

Rules_69	passed counts:3
node_0: feature_name=HuIgG_0.10	feature_id[2].value > threshold=19120.166015625
node_96: feature_name=a-HuIgG_0.03	feature_id[7].value <= threshold=19665.666015625
node_97: feature_name=MERS.CoV.S1.RBD.367.606.rFcTag	feature_id[5].value > threshold=3252.625
node_201: feature_name=SARS.CoV.2.S2	feature_id[29].value > threshold=37167.458984375
node_301: feature_name=SARS.CoV.2.S1.mFcTag	feature_id[21].value <= threshold=53322.333984375
node_302: feature_name=MoIgG_0.03	feature_id[20].value <= threshold=18.166666507720947
node_303: feature_name=MoIgG_0.30	feature_id[34].value > threshold=7257.166748046875
node_315: feature_name=Flu.H3N2.HA1+HA2	feature_id[38].value > threshold=52402.140625
Class: healthcare workers between 60 and 180 days after vaccination	
Rules_70	passed counts:3
node_0: feature_name=HuIgG_0.10	feature_id[2].value > threshold=19120.166015625
node_96: feature_name=a-HuIgG_0.03	feature_id[7].value <= threshold=19665.666015625
node_97: feature_name=MERS.CoV.S1.RBD.367.606.rFcTag	feature_id[5].value > threshold=3252.625
node_201: feature_name=SARS.CoV.2.S2	feature_id[29].value > threshold=37167.458984375
node_301: feature_name=SARS.CoV.2.S1.mFcTag	feature_id[21].value <= threshold=53322.333984375
node_302: feature_name=MoIgG_0.03	feature_id[20].value <= threshold=18.166666507720947
node_303: feature_name=MoIgG_0.30	feature_id[34].value <= threshold=7257.166748046875
node_304: feature_name=hCoV.NL63.S1_S2	feature_id[13].value <= threshold=52607.083984375
node_305: feature_name=HuIgG_0.30	feature_id[36].value <= threshold=35912.0
node_306: feature_name=hCoV.229E.S1	feature_id[30].value > threshold=31286.6669921875
node_308: feature_name=SARS.CoV.2.S2	feature_id[29].value > threshold=41902.466796875
Class: healthcare workers between 60 and 180 days after vaccination	

Rules_71	passed counts:3
node_0: feature_name=HuIgG_0.10	feature_id[2].value > threshold=19120.166015625
node_96: feature_name=a-HuIgG_0.03	feature_id[7].value <= threshold=19665.666015625
node_97: feature_name=MERS.CoV.S1.RBD.367.606.rFcTag	feature_id[5].value > threshold=3252.625
node_201: feature_name=SARS.CoV.2.S2	feature_id[29].value <= threshold=37167.458984375
node_202: feature_name=SARS.CoV.2.Spike.RBD.His.Bac	feature_id[1].value > threshold=16030.75048828125
node_212: feature_name=a-MoIgA_0.30	feature_id[37].value > threshold=1303.0
node_224: feature_name=HuIgA_0.03	feature_id[26].value > threshold=25.83333396911621
node_276: feature_name=hCoV.OC43.NP	feature_id[23].value > threshold=3591.10009765625
node_290: feature_name=SARS.CoV.2.Spike.RBD.His.Bac	feature_id[1].value > threshold=33649.125
node_296: feature_name=HuIgM_0.03	feature_id[14].value <= threshold=-90.5
node_297: feature_name=hCoV.OC43.HE	feature_id[33].value <= threshold=20650.0830078125
Class: healthcare workers within 60 days after vaccination	
Rules_72	passed counts:3
node_0: feature_name=HuIgG_0.10	feature_id[2].value > threshold=19120.166015625
node_96: feature_name=a-HuIgG_0.03	feature_id[7].value <= threshold=19665.666015625
node_97: feature_name=MERS.CoV.S1.RBD.367.606.rFcTag	feature_id[5].value > threshold=3252.625
node_201: feature_name=SARS.CoV.2.S2	feature_id[29].value <= threshold=37167.458984375
node_202: feature_name=SARS.CoV.2.Spike.RBD.His.Bac	feature_id[1].value > threshold=16030.75048828125
node_212: feature_name=a-MoIgA_0.30	feature_id[37].value > threshold=1303.0
node_224: feature_name=HuIgA_0.03	feature_id[26].value > threshold=25.83333396911621
node_276: feature_name=hCoV.OC43.NP	feature_id[23].value > threshold=3591.10009765625
node_290: feature_name=SARS.CoV.2.Spike.RBD.His.Bac	feature_id[1].value <= threshold=33649.125
node_291: feature_name=SARS.CoV.2.S1.mFcTag	feature_id[21].value <= threshold=49171.599609375

Class: healthcare workers between 60 and 180 days after vaccination	
Rules_73	passed counts:3
node_0: feature_name=HuIgG_0.10	feature_id[2].value > threshold=19120.166015625
node_96: feature_name=a-HuIgG_0.03	feature_id[7].value <= threshold=19665.666015625
node_97: feature_name=MERS.CoV.S1.RBD.367.606.rFcTag	feature_id[5].value > threshold=3252.625
node_201: feature_name=SARS.CoV.2.S2	feature_id[29].value <= threshold=37167.458984375
node_202: feature_name=SARS.CoV.2.Spike.RBD.His.Bac	feature_id[1].value > threshold=16030.75048828125
node_212: feature_name=a-MoIgA_0.30	feature_id[37].value > threshold=1303.0
node_224: feature_name=HuIgA_0.03	feature_id[26].value <= threshold=25.83333396911621
node_225: feature_name=Flu.B_Phu/.HA1+HA2	feature_id[19].value <= threshold=54802.5
node_226: feature_name=hCoV.HKU1.NP	feature_id[11].value <= threshold=13460.3330078125
node_227: feature_name=MoIgG_0.30	feature_id[34].value > threshold=3760.5
node_229: feature_name=HuIgM_0.10	feature_id[6].value > threshold=-87.16666412353516
node_233: feature_name=a-HuIgG_0.10	feature_id[3].value > threshold=27244.3330078125
node_235: feature_name=SARS.CoV.S1.RBD.rFcTag	feature_id[16].value > threshold=20123.43359375
node_245: feature_name=hCoV.OC43.NP	feature_id[23].value > threshold=1775.816650390625
node_253: feature_name=SARS.CoV.S1.RBD.rFcTag	feature_id[16].value > threshold=21641.4169921875
node_255: feature_name=HuIgA_0.03	feature_id[26].value > threshold=15.333333015441895
Class: healthcare workers within 60 days after vaccination	
Rules_74	passed counts:3
node_0: feature_name=HuIgG_0.10	feature_id[2].value > threshold=19120.166015625
node_96: feature_name=a-HuIgG_0.03	feature_id[7].value <= threshold=19665.666015625
node_97: feature_name=MERS.CoV.S1.RBD.367.606.rFcTag	feature_id[5].value > threshold=3252.625

node_201: feature_name=SARS.CoV.2.S2	feature_id[29].value <= threshold=37167.458984375
node_202: feature_name=SARS.CoV.2.Spike.RBD.His.Bac	feature_id[1].value > threshold=16030.75048828125
node_212: feature_name=a-MoIgA_0.30	feature_id[37].value > threshold=1303.0
node_224: feature_name=HuIgA_0.03	feature_id[26].value <= threshold=25.83333396911621
node_225: feature_name=Flu.B_Phu/.HA1+HA2	feature_id[19].value <= threshold=54802.5
node_226: feature_name=hCoV.HKU1.NP	feature_id[11].value <= threshold=13460.3330078125
node_227: feature_name=MoIgG_0.30	feature_id[34].value > threshold=3760.5
node_229: feature_name=HuIgM_0.10	feature_id[6].value > threshold=-87.16666412353516
node_233: feature_name=a-HuIgG_0.10	feature_id[3].value > threshold=27244.3330078125
node_235: feature_name=SARS.CoV.S1.RBD.rFcTag	feature_id[16].value > threshold=20123.43359375
node_245: feature_name=hCoV.OC43.NP	feature_id[23].value > threshold=1775.816650390625
node_253: feature_name=SARS.CoV.S1.RBD.rFcTag	feature_id[16].value > threshold=21641.4169921875
node_255: feature_name=HuIgA_0.03	feature_id[26].value <= threshold=15.333333015441895
node_256: feature_name=Flu.H3N2.HA1	feature_id[25].value <= threshold=52674.0
node_257: feature_name=hCoV.HKU1.S1_S2	feature_id[32].value <= threshold=37449.416015625
node_258: feature_name=Flu.B_Mal/.HA1	feature_id[9].value > threshold=27139.75
Class: healthcare workers within 60 days after vaccination	
Rules_75	passed counts:3
node_0: feature_name=HuIgG_0.10	feature_id[2].value > threshold=19120.166015625
node_96: feature_name=a-HuIgG_0.03	feature_id[7].value <= threshold=19665.666015625
node_97: feature_name=MERS.CoV.S1.RBD.367.606.rFcTag	feature_id[5].value > threshold=3252.625
node_201: feature_name=SARS.CoV.2.S2	feature_id[29].value <= threshold=37167.458984375
node_202: feature_name=SARS.CoV.2.Spike.RBD.His.Bac	feature_id[1].value > threshold=16030.75048828125
node_212: feature_name=a-MoIgA_0.30	feature_id[37].value > threshold=1303.0

node_224: feature_name=HuIgA_0.03	feature_id[26].value <= threshold=25.83333396911621
node_225: feature_name=Flu.B_Phu/.HA1+HA2	feature_id[19].value <= threshold=54802.5
node_226: feature_name=hCoV.HKU1.NP	feature_id[11].value <= threshold=13460.3330078125
node_227: feature_name=MoIgG_0.30	feature_id[34].value > threshold=3760.5
node_229: feature_name=HuIgM_0.10	feature_id[6].value > threshold=-87.16666412353516
node_233: feature_name=a-HuIgG_0.10	feature_id[3].value > threshold=27244.3330078125
node_235: feature_name=SARS.CoV.S1.RBD.rFcTag	feature_id[16].value > threshold=20123.43359375
node_245: feature_name=hCoV.OC43.NP	feature_id[23].value <= threshold=1775.816650390625
node_246: feature_name=HuIgA_0.03	feature_id[26].value > threshold=-10.333333492279053
Class: healthcare workers between 60 and 180 days after vaccination	
Rules_76	passed counts:3
node_0: feature_name=HuIgG_0.10	feature_id[2].value > threshold=19120.166015625
node_96: feature_name=a-HuIgG_0.03	feature_id[7].value <= threshold=19665.666015625
node_97: feature_name=MERS.CoV.S1.RBD.367.606.rFcTag	feature_id[5].value > threshold=3252.625
node_201: feature_name=SARS.CoV.2.S2	feature_id[29].value <= threshold=37167.458984375
node_202: feature_name=SARS.CoV.2.Spike.RBD.His.Bac	feature_id[1].value > threshold=16030.75048828125
node_212: feature_name=a-MoIgA_0.30	feature_id[37].value > threshold=1303.0
node_224: feature_name=HuIgA_0.03	feature_id[26].value <= threshold=25.83333396911621
node_225: feature_name=Flu.B_Phu/.HA1+HA2	feature_id[19].value <= threshold=54802.5
node_226: feature_name=hCoV.HKU1.NP	feature_id[11].value <= threshold=13460.3330078125
node_227: feature_name=MoIgG_0.30	feature_id[34].value > threshold=3760.5
node_229: feature_name=HuIgM_0.10	feature_id[6].value > threshold=-87.16666412353516
node_233: feature_name=a-HuIgG_0.10	feature_id[3].value > threshold=27244.3330078125
node_235: feature_name=SARS.CoV.S1.RBD.rFcTag	feature_id[16].value <= threshold=20123.43359375

node_236: feature_name=MERS.CoV.S1.RBD.367.606.rFcTag	feature_id[5].value > threshold=4326.625
node_238: feature_name=SARS.CoV.NP	feature_id[31].value > threshold=2842.375
node_240: feature_name=Flu.H1N1.HA1+HA2	feature_id[10].value > threshold=53053.666015625
Class: healthcare workers within 60 days after vaccination	
Rules_77	passed counts:3
node_0: feature_name=HuIgG_0.10	feature_id[2].value > threshold=19120.166015625
node_96: feature_name=a-HuIgG_0.03	feature_id[7].value <= threshold=19665.666015625
node_97: feature_name=MERS.CoV.S1.RBD.367.606.rFcTag	feature_id[5].value > threshold=3252.625
node_201: feature_name=SARS.CoV.2.S2	feature_id[29].value <= threshold=37167.458984375
node_202: feature_name=SARS.CoV.2.Spike.RBD.His.Bac	feature_id[1].value > threshold=16030.75048828125
node_212: feature_name=a-MoIgA_0.30	feature_id[37].value <= threshold=1303.0
node_213: feature_name=MERS.CoV.NP	feature_id[15].value <= threshold=898.7833251953125
node_214: feature_name=hCoV.OC43.HE	feature_id[33].value <= threshold=13271.4169921875
Class: healthcare workers between 60 and 180 days after vaccination	
Rules_78	passed counts:3
node_0: feature_name=HuIgG_0.10	feature_id[2].value > threshold=19120.166015625
node_96: feature_name=a-HuIgG_0.03	feature_id[7].value <= threshold=19665.666015625
node_97: feature_name=MERS.CoV.S1.RBD.367.606.rFcTag	feature_id[5].value <= threshold=3252.625
node_98: feature_name=SARS.CoV.2.S2	feature_id[29].value > threshold=9506.32470703125
node_116: feature_name=Flu.H1N1.HA1+HA2	feature_id[10].value <= threshold=54065.41796875
node_117: feature_name=SARS.CoV.S1.RBD.rFcTag	feature_id[16].value > threshold=24639.0
node_167: feature_name=hCoV.OC43.HE	feature_id[33].value <= threshold=32466.5830078125
node_168: feature_name=HuIgM_0.10	feature_id[6].value <= threshold=22.833333015441895

node_169: feature_name=SARS.CoV.2.S1.mFcTag	feature_id[21].value <= threshold=54847.25
node_170: feature_name=a-MoIgG_0.03	feature_id[12].value > threshold=2342.8333740234375
node_172: feature_name=HuIgM_0.03	feature_id[14].value > threshold=-66.33333206176758
Class: healthcare workers within 60 days after vaccination	
Rules_79	passed counts:3
node_0: feature_name=HuIgG_0.10	feature_id[2].value > threshold=19120.166015625
node_96: feature_name=a-HuIgG_0.03	feature_id[7].value <= threshold=19665.666015625
node_97: feature_name=MERS.CoV.S1.RBD.367.606.rFcTag	feature_id[5].value <= threshold=3252.625
node_98: feature_name=SARS.CoV.2.S2	feature_id[29].value > threshold=9506.32470703125
node_116: feature_name=Flu.H1N1.HA1+HA2	feature_id[10].value <= threshold=54065.41796875
node_117: feature_name=SARS.CoV.S1.RBD.rFcTag	feature_id[16].value <= threshold=24639.0
node_118: feature_name=SARS.CoV.2.Spike.RBD.His.Bac	feature_id[1].value > threshold=23841.4169921875
node_128: feature_name=SARS.CoV.2.S1+S2	feature_id[17].value > threshold=41778.25
node_138: feature_name=hCoV.NL63.S1_S2	feature_id[13].value > threshold=35816.083984375
node_146: feature_name=a-MoIgG_0.10	feature_id[27].value > threshold=3760.5
node_156: feature_name=a-HuIgG_0.03	feature_id[7].value > threshold=9740.8330078125
node_158: feature_name=a-HuIgG_0.30	feature_id[4].value > threshold=42960.0
node_160: feature_name=hCoV.HKU1.NP	feature_id[11].value <= threshold=3927.666748046875
Class: healthcare workers between 60 and 180 days after vaccination	
Rules_80	passed counts:3
node_0: feature_name=HuIgG_0.10	feature_id[2].value > threshold=19120.166015625
node_96: feature_name=a-HuIgG_0.03	feature_id[7].value <= threshold=19665.666015625
node_97: feature_name=MERS.CoV.S1.RBD.367.606.rFcTag	feature_id[5].value <= threshold=3252.625

node_98: feature_name=SARS.CoV.2.S2	feature_id[29].value <= threshold=9506.32470703125
node_99: feature_name=SARS.CoV.2.S1.RBD.mFc	feature_id[28].value <= threshold=4672.199844360352
node_100: feature_name=a-MoIgA_0.03	feature_id[24].value > threshold=14.999999523162842
node_106: feature_name=HuIgM_0.10	feature_id[6].value > threshold=65.66666793823242
node_108: feature_name=SARS.CoV.2.S2	feature_id[29].value > threshold=2100.388916015625
Class: healthcare workers within 60 days after vaccination	
Rules_81	passed counts:3
node_0: feature_name=HuIgG_0.10	feature_id[2].value <= threshold=19120.166015625
node_1: feature_name=SARS.CoV.2.S1.RBD.mFc	feature_id[28].value > threshold=3773.8334350585938
node_9: feature_name=HuIgM_0.03	feature_id[14].value > threshold=-88.33333206176758
node_27: feature_name=hCoV.HKU1.S1_S2	feature_id[32].value > threshold=42767.0
node_77: feature_name=Flu.B_Mal/.HA1	feature_id[9].value <= threshold=41191.69921875
node_78: feature_name=SARS.CoV.2.S1.mFcTag	feature_id[21].value <= threshold=41709.720703125
node_79: feature_name=SARS.CoV.S1.HisTag	feature_id[18].value <= threshold=3125.4667358398438
Class: unvaccinated healthcare workers	
Rules_82	passed counts:3
node_0: feature_name=HuIgG_0.10	feature_id[2].value <= threshold=19120.166015625
node_1: feature_name=SARS.CoV.2.S1.RBD.mFc	feature_id[28].value > threshold=3773.8334350585938
node_9: feature_name=HuIgM_0.03	feature_id[14].value > threshold=-88.33333206176758
node_27: feature_name=hCoV.HKU1.S1_S2	feature_id[32].value <= threshold=42767.0
node_28: feature_name=HuIgG_0.03	feature_id[0].value > threshold=1005.0
node_38: feature_name=a-HuIgG_0.30	feature_id[4].value > threshold=33596.333984375
node_60: feature_name=a-HuIgG_0.03	feature_id[7].value <= threshold=10366.5

node_61: feature_name=Flu.H3N2.HA1	feature_id[25].value <= threshold=29968.0830078125
node_62: feature_name=HuIgA_0.03	feature_id[26].value > threshold=62.33333337306976
Class: healthcare workers over 180 days after vaccination	
Rules_83	passed counts:3
node_0: feature_name=HuIgG_0.10	feature_id[2].value <= threshold=19120.166015625
node_1: feature_name=SARS.CoV.2.S1.RBD.mFc	feature_id[28].value > threshold=3773.8334350585938
node_9: feature_name=HuIgM_0.03	feature_id[14].value > threshold=-88.33333206176758
node_27: feature_name=hCoV.HKU1.S1_S2	feature_id[32].value <= threshold=42767.0
node_28: feature_name=HuIgG_0.03	feature_id[0].value > threshold=1005.0
node_38: feature_name=a-HuIgG_0.30	feature_id[4].value > threshold=33596.333984375
node_60: feature_name=a-HuIgG_0.03	feature_id[7].value <= threshold=10366.5
node_61: feature_name=Flu.H3N2.HA1	feature_id[25].value <= threshold=29968.0830078125
node_62: feature_name=HuIgA_0.03	feature_id[26].value <= threshold=62.33333337306976
Class: healthcare workers between 60 and 180 days after vaccination	
Rules_84	passed counts:3
node_0: feature_name=HuIgG_0.10	feature_id[2].value <= threshold=19120.166015625
node_1: feature_name=SARS.CoV.2.S1.RBD.mFc	feature_id[28].value <= threshold=3773.8334350585938
node_2: feature_name=SARS.CoV.2.S1.RBD.mFc	feature_id[28].value > threshold=-414.2999954223633
node_4: feature_name=hCoV.OC43.HE	feature_id[33].value <= threshold=414.5370330810547
node_5: feature_name=Flu.H1N1.HA1+HA2	feature_id[10].value <= threshold=29887.27734375
Class: unvaccinated healthcare workers	
Rules_85	passed counts:2

node_0: feature_name=HuIgG_0.10	feature_id[2].value > threshold=19120.166015625
node_96: feature_name=a-HuIgG_0.03	feature_id[7].value > threshold=19665.666015625
node_350: feature_name=HuIgM_0.03	feature_id[14].value > threshold=-189.0
node_354: feature_name=HuIgM_0.30	feature_id[8].value > threshold=441.8333282470703
node_356: feature_name=a-HuIgG_0.10	feature_id[3].value > threshold=32914.5
node_360: feature_name=a-MoIgG_0.03	feature_id[12].value > threshold=2414.8333740234375
Class: healthcare workers within 60 days after vaccination	
Rules_86	passed counts:2
node_0: feature_name=HuIgG_0.10	feature_id[2].value > threshold=19120.166015625
node_96: feature_name=a-HuIgG_0.03	feature_id[7].value > threshold=19665.666015625
node_350: feature_name=HuIgM_0.03	feature_id[14].value > threshold=-189.0
node_354: feature_name=HuIgM_0.30	feature_id[8].value > threshold=441.8333282470703
node_356: feature_name=a-HuIgG_0.10	feature_id[3].value > threshold=32914.5
node_360: feature_name=a-MoIgG_0.03	feature_id[12].value <= threshold=2414.8333740234375
node_361: feature_name=HuIgG_0.10	feature_id[2].value > threshold=28475.6669921875
Class: healthcare workers between 60 and 180 days after vaccination	
Rules_87	passed counts:2
node_0: feature_name=HuIgG_0.10	feature_id[2].value > threshold=19120.166015625
node_96: feature_name=a-HuIgG_0.03	feature_id[7].value > threshold=19665.666015625
node_350: feature_name=HuIgM_0.03	feature_id[14].value > threshold=-189.0
node_354: feature_name=HuIgM_0.30	feature_id[8].value > threshold=441.8333282470703
node_356: feature_name=a-HuIgG_0.10	feature_id[3].value <= threshold=32914.5
node_357: feature_name=MERS.CoV.S1.RBD.367.606.rFcTag	feature_id[5].value > threshold=6711.89990234375

Class: healthcare workers over 180 days after vaccination	
Rules_88	passed counts:2
node_0: feature_name=HuIgG_0.10	feature_id[2].value > threshold=19120.166015625
node_96: feature_name=a-HuIgG_0.03	feature_id[7].value <= threshold=19665.666015625
node_97: feature_name=MERS.CoV.S1.RBD.367.606.rFcTag	feature_id[5].value > threshold=3252.625
node_201: feature_name=SARS.CoV.2.S2	feature_id[29].value > threshold=37167.458984375
node_301: feature_name=SARS.CoV.2.S1.mFcTag	feature_id[21].value > threshold=53322.333984375
node_321: feature_name=a-MoIgA_0.03	feature_id[24].value <= threshold=212.0
node_322: feature_name=Flu.H3N2.HA1	feature_id[25].value > threshold=37503.517578125
node_330: feature_name=a-MoIgG_0.03	feature_id[12].value <= threshold=556.6666564941406
node_331: feature_name=hCoV.229E.S1_S2	feature_id[35].value <= threshold=47263.833984375
node_332: feature_name=a-HuIgG_0.10	feature_id[3].value > threshold=30393.166015625
node_336: feature_name=hCoV.NL63.S1_S2	feature_id[13].value > threshold=53473.16796875
Class: healthcare workers within 60 days after vaccination	
Rules_89	passed counts:2
node_0: feature_name=HuIgG_0.10	feature_id[2].value > threshold=19120.166015625
node_96: feature_name=a-HuIgG_0.03	feature_id[7].value <= threshold=19665.666015625
node_97: feature_name=MERS.CoV.S1.RBD.367.606.rFcTag	feature_id[5].value > threshold=3252.625
node_201: feature_name=SARS.CoV.2.S2	feature_id[29].value > threshold=37167.458984375
node_301: feature_name=SARS.CoV.2.S1.mFcTag	feature_id[21].value <= threshold=53322.333984375
node_302: feature_name=MoIgG_0.03	feature_id[20].value <= threshold=18.166666507720947
node_303: feature_name=MoIgG_0.30	feature_id[34].value <= threshold=7257.166748046875
node_304: feature_name=hCoV.NL63.S1_S2	feature_id[13].value > threshold=52607.083984375

Class: healthcare workers between 60 and 180 days after vaccination	
Rules_90	passed counts:2
node_0: feature_name=HuIgG_0.10	feature_id[2].value > threshold=19120.166015625
node_96: feature_name=a-HuIgG_0.03	feature_id[7].value <= threshold=19665.666015625
node_97: feature_name=MERS.CoV.S1.RBD.367.606.rFcTag	feature_id[5].value > threshold=3252.625
node_201: feature_name=SARS.CoV.2.S2	feature_id[29].value <= threshold=37167.458984375
node_202: feature_name=SARS.CoV.2.Spike.RBD.His.Bac	feature_id[1].value > threshold=16030.75048828125
node_212: feature_name=a-MoIgA_0.30	feature_id[37].value > threshold=1303.0
node_224: feature_name=HuIgA_0.03	feature_id[26].value <= threshold=25.83333396911621
node_225: feature_name=Flu.B_Phu/.HA1+HA2	feature_id[19].value <= threshold=54802.5
node_226: feature_name=hCoV.HKU1.NP	feature_id[11].value <= threshold=13460.3330078125
node_227: feature_name=MoIgG_0.30	feature_id[34].value > threshold=3760.5
node_229: feature_name=HuIgM_0.10	feature_id[6].value > threshold=-87.16666412353516
node_233: feature_name=a-HuIgG_0.10	feature_id[3].value > threshold=27244.3330078125
node_235: feature_name=SARS.CoV.S1.RBD.rFcTag	feature_id[16].value > threshold=20123.43359375
node_245: feature_name=hCoV.OC43.NP	feature_id[23].value > threshold=1775.816650390625
node_253: feature_name=SARS.CoV.S1.RBD.rFcTag	feature_id[16].value > threshold=21641.4169921875
node_255: feature_name=HuIgA_0.03	feature_id[26].value <= threshold=15.333333015441895
node_256: feature_name=Flu.H3N2.HA1	feature_id[25].value > threshold=52674.0
Class: healthcare workers within 60 days after vaccination	
Rules_91	passed counts:2
node_0: feature_name=HuIgG_0.10	feature_id[2].value > threshold=19120.166015625
node_96: feature_name=a-HuIgG_0.03	feature_id[7].value <= threshold=19665.666015625

node_97: feature_name=MERS.CoV.S1.RBD.367.606.rFcTag	feature_id[5].value > threshold=3252.625
node_201: feature_name=SARS.CoV.2.S2	feature_id[29].value <= threshold=37167.458984375
node_202: feature_name=SARS.CoV.2.Spike.RBD.His.Bac	feature_id[1].value > threshold=16030.75048828125
node_212: feature_name=a-MoIgA_0.30	feature_id[37].value > threshold=1303.0
node_224: feature_name=HuIgA_0.03	feature_id[26].value <= threshold=25.83333396911621
node_225: feature_name=Flu.B_Phu/.HA1+HA2	feature_id[19].value <= threshold=54802.5
node_226: feature_name=hCoV.HKU1.NP	feature_id[11].value <= threshold=13460.3330078125
node_227: feature_name=MoIgG_0.30	feature_id[34].value > threshold=3760.5
node_229: feature_name=HuIgM_0.10	feature_id[6].value > threshold=-87.16666412353516
node_233: feature_name=a-HuIgG_0.10	feature_id[3].value > threshold=27244.3330078125
node_235: feature_name=SARS.CoV.S1.RBD.rFcTag	feature_id[16].value > threshold=20123.43359375
node_245: feature_name=hCoV.OC43.NP	feature_id[23].value > threshold=1775.816650390625
node_253: feature_name=SARS.CoV.S1.RBD.rFcTag	feature_id[16].value > threshold=21641.4169921875
node_255: feature_name=HuIgA_0.03	feature_id[26].value <= threshold=15.333333015441895
node_256: feature_name=Flu.H3N2.HA1	feature_id[25].value <= threshold=52674.0
node_257: feature_name=hCoV.HKU1.S1_S2	feature_id[32].value > threshold=37449.416015625
node_261: feature_name=SARS.CoV.2.S1+S2	feature_id[17].value <= threshold=42043.224609375
Class: healthcare workers within 60 days after vaccination	
Rules_92	passed counts:2
node_0: feature_name=HuIgG_0.10	feature_id[2].value > threshold=19120.166015625
node_96: feature_name=a-HuIgG_0.03	feature_id[7].value <= threshold=19665.666015625
node_97: feature_name=MERS.CoV.S1.RBD.367.606.rFcTag	feature_id[5].value > threshold=3252.625
node_201: feature_name=SARS.CoV.2.S2	feature_id[29].value <= threshold=37167.458984375
node_202: feature_name=SARS.CoV.2.Spike.RBD.His.Bac	feature_id[1].value > threshold=16030.75048828125

node_212: feature_name=a-MoIgA_0.30	feature_id[37].value > threshold=1303.0
node_224: feature_name=HuIgA_0.03	feature_id[26].value <= threshold=25.83333396911621
node_225: feature_name=Flu.B_Phu/.HA1+HA2	feature_id[19].value <= threshold=54802.5
node_226: feature_name=hCoV.HKU1.NP	feature_id[11].value <= threshold=13460.3330078125
node_227: feature_name=MoIgG_0.30	feature_id[34].value > threshold=3760.5
node_229: feature_name=HuIgM_0.10	feature_id[6].value > threshold=-87.16666412353516
node_233: feature_name=a-HuIgG_0.10	feature_id[3].value > threshold=27244.3330078125
node_235: feature_name=SARS.CoV.S1.RBD.rFcTag	feature_id[16].value > threshold=20123.43359375
node_245: feature_name=hCoV.OC43.NP	feature_id[23].value <= threshold=1775.816650390625
node_246: feature_name=HuIgA_0.03	feature_id[26].value <= threshold=-10.333333492279053
node_247: feature_name=SARS.CoV.2.S1.RBD.mFc	feature_id[28].value > threshold=53114.833984375
node_249: feature_name=HuIgG_0.10	feature_id[2].value <= threshold=24164.3330078125
Class: healthcare workers over 180 days after vaccination	
Rules_93	passed counts:2
node_0: feature_name=HuIgG_0.10	feature_id[2].value > threshold=19120.166015625
node_96: feature_name=a-HuIgG_0.03	feature_id[7].value <= threshold=19665.666015625
node_97: feature_name=MERS.CoV.S1.RBD.367.606.rFcTag	feature_id[5].value > threshold=3252.625
node_201: feature_name=SARS.CoV.2.S2	feature_id[29].value <= threshold=37167.458984375
node_202: feature_name=SARS.CoV.2.Spike.RBD.His.Bac	feature_id[1].value > threshold=16030.75048828125
node_212: feature_name=a-MoIgA_0.30	feature_id[37].value <= threshold=1303.0
node_213: feature_name=MERS.CoV.NP	feature_id[15].value > threshold=898.7833251953125
node_217: feature_name=hCoV.NL63.S1_S2	feature_id[13].value > threshold=30153.3330078125
node_219: feature_name=SARS.CoV.S1.HisTag	feature_id[18].value <= threshold=4305.2916259765625
node_220: feature_name=HuIgG_0.30	feature_id[36].value <= threshold=36339.833984375

Class: healthcare workers between 60 and 180 days after vaccination	
Rules_94	passed counts:2
node_0: feature_name=HuIgG_0.10	feature_id[2].value > threshold=19120.166015625
node_96: feature_name=a-HuIgG_0.03	feature_id[7].value <= threshold=19665.666015625
node_97: feature_name=MERS.CoV.S1.RBD.367.606.rFcTag	feature_id[5].value > threshold=3252.625
node_201: feature_name=SARS.CoV.2.S2	feature_id[29].value <= threshold=37167.458984375
node_202: feature_name=SARS.CoV.2.Spike.RBD.His.Bac	feature_id[1].value > threshold=16030.75048828125
node_212: feature_name=a-MoIgA_0.30	feature_id[37].value <= threshold=1303.0
node_213: feature_name=MERS.CoV.NP	feature_id[15].value > threshold=898.7833251953125
node_217: feature_name=hCoV.NL63.S1_S2	feature_id[13].value <= threshold=30153.3330078125
Class: healthcare workers over 180 days after vaccination	
Rules_95	passed counts:2
node_0: feature_name=HuIgG_0.10	feature_id[2].value > threshold=19120.166015625
node_96: feature_name=a-HuIgG_0.03	feature_id[7].value <= threshold=19665.666015625
node_97: feature_name=MERS.CoV.S1.RBD.367.606.rFcTag	feature_id[5].value > threshold=3252.625
node_201: feature_name=SARS.CoV.2.S2	feature_id[29].value <= threshold=37167.458984375
node_202: feature_name=SARS.CoV.2.Spike.RBD.His.Bac	feature_id[1].value > threshold=16030.75048828125
node_212: feature_name=a-MoIgA_0.30	feature_id[37].value <= threshold=1303.0
node_213: feature_name=MERS.CoV.NP	feature_id[15].value <= threshold=898.7833251953125
node_214: feature_name=hCoV.OC43.HE	feature_id[33].value > threshold=13271.4169921875
Class: healthcare workers over 180 days after vaccination	
Rules_96	passed counts:2

node_0: feature_name=HuIgG_0.10	feature_id[2].value > threshold=19120.166015625
node_96: feature_name=a-HuIgG_0.03	feature_id[7].value <= threshold=19665.666015625
node_97: feature_name=MERS.CoV.S1.RBD.367.606.rFcTag	feature_id[5].value > threshold=3252.625
node_201: feature_name=SARS.CoV.2.S2	feature_id[29].value <= threshold=37167.458984375
node_202: feature_name=SARS.CoV.2.Spike.RBD.His.Bac	feature_id[1].value <= threshold=16030.75048828125
node_203: feature_name=a-MoIgG_0.10	feature_id[27].value > threshold=2117.9999389648438
node_207: feature_name=SARS.CoV.S1.HisTag	feature_id[18].value <= threshold=1072.50830078125
node_208: feature_name=Flu.H3N2.HA1+HA2	feature_id[38].value <= threshold=54173.125
Class: unvaccinated healthcare workers	
Rules_97	passed counts:2
node_0: feature_name=HuIgG_0.10	feature_id[2].value > threshold=19120.166015625
node_96: feature_name=a-HuIgG_0.03	feature_id[7].value <= threshold=19665.666015625
node_97: feature_name=MERS.CoV.S1.RBD.367.606.rFcTag	feature_id[5].value <= threshold=3252.625
node_98: feature_name=SARS.CoV.2.S2	feature_id[29].value > threshold=9506.32470703125
node_116: feature_name=Flu.H1N1.HA1+HA2	feature_id[10].value <= threshold=54065.41796875
node_117: feature_name=SARS.CoV.S1.RBD.rFcTag	feature_id[16].value > threshold=24639.0
node_167: feature_name=hCoV.OC43.HE	feature_id[33].value > threshold=32466.5830078125
Class: healthcare workers between 60 and 180 days after vaccination	
Rules_98	passed counts:2
node_0: feature_name=HuIgG_0.10	feature_id[2].value > threshold=19120.166015625
node_96: feature_name=a-HuIgG_0.03	feature_id[7].value <= threshold=19665.666015625
node_97: feature_name=MERS.CoV.S1.RBD.367.606.rFcTag	feature_id[5].value <= threshold=3252.625
node_98: feature_name=SARS.CoV.2.S2	feature_id[29].value > threshold=9506.32470703125

node_116: feature_name=Flu.H1N1.HA1+HA2	feature_id[10].value <= threshold=54065.41796875
node_117: feature_name=SARS.CoV.S1.RBD.rFcTag	feature_id[16].value > threshold=24639.0
node_167: feature_name=hCoV.OC43.HE	feature_id[33].value <= threshold=32466.5830078125
node_168: feature_name=HuIgM_0.10	feature_id[6].value > threshold=22.833333015441895
node_178: feature_name=a-MoIgA_0.03	feature_id[24].value > threshold=-34.333333015441895
node_184: feature_name=HuIgG_0.10	feature_id[2].value <= threshold=21401.166015625
node_185: feature_name=SARS.CoV.S1.HisTag	feature_id[18].value > threshold=7929.375244140625
Class: healthcare workers between 60 and 180 days after vaccination	
Rules_99	passed counts:2
node_0: feature_name=HuIgG_0.10	feature_id[2].value > threshold=19120.166015625
node_96: feature_name=a-HuIgG_0.03	feature_id[7].value <= threshold=19665.666015625
node_97: feature_name=MERS.CoV.S1.RBD.367.606.rFcTag	feature_id[5].value <= threshold=3252.625
node_98: feature_name=SARS.CoV.2.S2	feature_id[29].value > threshold=9506.32470703125
node_116: feature_name=Flu.H1N1.HA1+HA2	feature_id[10].value <= threshold=54065.41796875
node_117: feature_name=SARS.CoV.S1.RBD.rFcTag	feature_id[16].value > threshold=24639.0
node_167: feature_name=hCoV.OC43.HE	feature_id[33].value <= threshold=32466.5830078125
node_168: feature_name=HuIgM_0.10	feature_id[6].value <= threshold=22.833333015441895
node_169: feature_name=SARS.CoV.2.S1.mFcTag	feature_id[21].value > threshold=54847.25
node_175: feature_name=SARS.CoV.2.S1.RBD.mFc	feature_id[28].value <= threshold=49975.58203125
Class: healthcare workers between 60 and 180 days after vaccination	
Rules_100	passed counts:2
node_0: feature_name=HuIgG_0.10	feature_id[2].value > threshold=19120.166015625
node_96: feature_name=a-HuIgG_0.03	feature_id[7].value <= threshold=19665.666015625

node_97: feature_name=MERS.CoV.S1.RBD.367.606.rFcTag	feature_id[5].value <= threshold=3252.625
node_98: feature_name=SARS.CoV.2.S2	feature_id[29].value > threshold=9506.32470703125
node_116: feature_name=Flu.H1N1.HA1+HA2	feature_id[10].value <= threshold=54065.41796875
node_117: feature_name=SARS.CoV.S1.RBD.rFcTag	feature_id[16].value <= threshold=24639.0
node_118: feature_name=SARS.CoV.2.Spike.RBD.His.Bac	feature_id[1].value > threshold=23841.4169921875
node_128: feature_name=SARS.CoV.2.S1+S2	feature_id[17].value > threshold=41778.25
node_138: feature_name=hCoV.NL63.S1_S2	feature_id[13].value > threshold=35816.083984375
node_146: feature_name=a-MoIgG_0.10	feature_id[27].value > threshold=3760.5
node_156: feature_name=a-HuIgG_0.03	feature_id[7].value > threshold=9740.8330078125
node_158: feature_name=a-HuIgG_0.30	feature_id[4].value > threshold=42960.0
node_160: feature_name=hCoV.HKU1.NP	feature_id[11].value > threshold=3927.666748046875
node_162: feature_name=HuIgA_0.03	feature_id[26].value > threshold=595.6666870117188
node_164: feature_name=hCoV.HKU1.S1_S2	feature_id[32].value <= threshold=49940.333984375
Class: healthcare workers between 60 and 180 days after vaccination	
Rules_101	passed counts:2
node_0: feature_name=HuIgG_0.10	feature_id[2].value > threshold=19120.166015625
node_96: feature_name=a-HuIgG_0.03	feature_id[7].value <= threshold=19665.666015625
node_97: feature_name=MERS.CoV.S1.RBD.367.606.rFcTag	feature_id[5].value <= threshold=3252.625
node_98: feature_name=SARS.CoV.2.S2	feature_id[29].value > threshold=9506.32470703125
node_116: feature_name=Flu.H1N1.HA1+HA2	feature_id[10].value <= threshold=54065.41796875
node_117: feature_name=SARS.CoV.S1.RBD.rFcTag	feature_id[16].value <= threshold=24639.0
node_118: feature_name=SARS.CoV.2.Spike.RBD.His.Bac	feature_id[1].value > threshold=23841.4169921875
node_128: feature_name=SARS.CoV.2.S1+S2	feature_id[17].value > threshold=41778.25
node_138: feature_name=hCoV.NL63.S1_S2	feature_id[13].value > threshold=35816.083984375

node_146: feature_name=a-MoIgG_0.10	feature_id[27].value > threshold=3760.5
node_156: feature_name=a-HuIgG_0.03	feature_id[7].value <= threshold=9740.8330078125
Class: healthcare workers between 60 and 180 days after vaccination	
Rules_102	passed counts:2
node_0: feature_name=HuIgG_0.10	feature_id[2].value > threshold=19120.166015625
node_96: feature_name=a-HuIgG_0.03	feature_id[7].value <= threshold=19665.666015625
node_97: feature_name=MERS.CoV.S1.RBD.367.606.rFcTag	feature_id[5].value <= threshold=3252.625
node_98: feature_name=SARS.CoV.2.S2	feature_id[29].value > threshold=9506.32470703125
node_116: feature_name=Flu.H1N1.HA1+HA2	feature_id[10].value <= threshold=54065.41796875
node_117: feature_name=SARS.CoV.S1.RBD.rFcTag	feature_id[16].value <= threshold=24639.0
node_118: feature_name=SARS.CoV.2.Spike.RBD.His.Bac	feature_id[1].value > threshold=23841.4169921875
node_128: feature_name=SARS.CoV.2.S1+S2	feature_id[17].value > threshold=41778.25
node_138: feature_name=hCoV.NL63.S1_S2	feature_id[13].value > threshold=35816.083984375
node_146: feature_name=a-MoIgG_0.10	feature_id[27].value <= threshold=3760.5
node_147: feature_name=SARS.CoV.S1.HisTag	feature_id[18].value > threshold=3759.5833740234375
node_149: feature_name=SARS.CoV.S1.HisTag	feature_id[18].value > threshold=4886.25
node_151: feature_name=HuIgM_0.30	feature_id[8].value <= threshold=518.0000152587891
node_152: feature_name=a-HuIgG_0.03	feature_id[7].value > threshold=10986.3330078125
Class: healthcare workers within 60 days after vaccination	
Rules_103	passed counts:2
node_0: feature_name=HuIgG_0.10	feature_id[2].value > threshold=19120.166015625
node_96: feature_name=a-HuIgG_0.03	feature_id[7].value <= threshold=19665.666015625
node_97: feature_name=MERS.CoV.S1.RBD.367.606.rFcTag	feature_id[5].value <= threshold=3252.625

node_98: feature_name=SARS.CoV.2.S2	feature_id[29].value > threshold=9506.32470703125
node_116: feature_name=Flu.H1N1.HA1+HA2	feature_id[10].value <= threshold=54065.41796875
node_117: feature_name=SARS.CoV.S1.RBD.rFcTag	feature_id[16].value <= threshold=24639.0
node_118: feature_name=SARS.CoV.2.Spike.RBD.His.Bac	feature_id[1].value > threshold=23841.4169921875
node_128: feature_name=SARS.CoV.2.S1+S2	feature_id[17].value <= threshold=41778.25
node_129: feature_name=SARS.CoV.S1.HisTag	feature_id[18].value > threshold=3569.2833251953125
node_133: feature_name=Flu.B_Mal/.HA1+HA2	feature_id[22].value > threshold=47613.583984375
node_135: feature_name=SARS.CoV.2.Spike.RBD.His.Bac	feature_id[1].value > threshold=27719.5087890625
Class: healthcare workers over 180 days after vaccination	
Rules_104	passed counts:2
node_0: feature_name=HuIgG_0.10	feature_id[2].value <= threshold=19120.166015625
node_1: feature_name=SARS.CoV.2.S1.RBD.mFc	feature_id[28].value > threshold=3773.8334350585938
node_9: feature_name=HuIgM_0.03	feature_id[14].value > threshold=-88.33333206176758
node_27: feature_name=hCoV.HKU1.S1_S2	feature_id[32].value > threshold=42767.0
node_77: feature_name=Flu.B_Mal/.HA1	feature_id[9].value > threshold=41191.69921875
node_91: feature_name=hCoV.NL63.S1_S2	feature_id[13].value > threshold=33974.650390625
node_93: feature_name=Flu.H1N1.HA1+HA2	feature_id[10].value <= threshold=54167.6015625
Class: healthcare workers between 60 and 180 days after vaccination	
Rules_105	passed counts:2
node_0: feature_name=HuIgG_0.10	feature_id[2].value <= threshold=19120.166015625
node_1: feature_name=SARS.CoV.2.S1.RBD.mFc	feature_id[28].value > threshold=3773.8334350585938
node_9: feature_name=HuIgM_0.03	feature_id[14].value > threshold=-88.33333206176758
node_27: feature_name=hCoV.HKU1.S1_S2	feature_id[32].value > threshold=42767.0

node_77: feature_name=Flu.B_Mal/.HA1	feature_id[9].value <= threshold=41191.69921875
node_78: feature_name=SARS.CoV.2.S1.mFcTag	feature_id[21].value > threshold=41709.720703125
node_82: feature_name=HuIgM_0.10	feature_id[6].value > threshold=-43.83333396911621
node_86: feature_name=HuIgG_0.10	feature_id[2].value > threshold=18966.0
Class: healthcare workers between 60 and 180 days after vaccination	
Rules_106	passed counts:2
node_0: feature_name=HuIgG_0.10	feature_id[2].value <= threshold=19120.166015625
node_1: feature_name=SARS.CoV.2.S1.RBD.mFc	feature_id[28].value > threshold=3773.8334350585938
node_9: feature_name=HuIgM_0.03	feature_id[14].value > threshold=-88.33333206176758
node_27: feature_name=hCoV.HKU1.S1_S2	feature_id[32].value > threshold=42767.0
node_77: feature_name=Flu.B_Mal/.HA1	feature_id[9].value <= threshold=41191.69921875
node_78: feature_name=SARS.CoV.2.S1.mFcTag	feature_id[21].value <= threshold=41709.720703125
node_79: feature_name=SARS.CoV.S1.HisTag	feature_id[18].value > threshold=3125.4667358398438
Class: healthcare workers over 180 days after vaccination	
Rules_107	passed counts:2
node_0: feature_name=HuIgG_0.10	feature_id[2].value <= threshold=19120.166015625
node_1: feature_name=SARS.CoV.2.S1.RBD.mFc	feature_id[28].value > threshold=3773.8334350585938
node_9: feature_name=HuIgM_0.03	feature_id[14].value > threshold=-88.33333206176758
node_27: feature_name=hCoV.HKU1.S1_S2	feature_id[32].value <= threshold=42767.0
node_28: feature_name=HuIgG_0.03	feature_id[0].value > threshold=1005.0
node_38: feature_name=a-HuIgG_0.30	feature_id[4].value > threshold=33596.333984375
node_60: feature_name=a-HuIgG_0.03	feature_id[7].value > threshold=10366.5
node_70: feature_name=hCoV.OC43.HE	feature_id[33].value > threshold=23182.7001953125

Class: healthcare workers over 180 days after vaccination	
Rules_108	passed counts:2
node_0: feature_name=HuIgG_0.10	feature_id[2].value <= threshold=19120.166015625
node_1: feature_name=SARS.CoV.2.S1.RBD.mFc	feature_id[28].value > threshold=3773.8334350585938
node_9: feature_name=HuIgM_0.03	feature_id[14].value > threshold=-88.33333206176758
node_27: feature_name=hCoV.HKU1.S1_S2	feature_id[32].value <= threshold=42767.0
node_28: feature_name=HuIgG_0.03	feature_id[0].value > threshold=1005.0
node_38: feature_name=a-HuIgG_0.30	feature_id[4].value <= threshold=33596.333984375
node_39: feature_name=SARS.CoV.S1.HisTag	feature_id[18].value > threshold=400.3000030517578
node_43: feature_name=HuIgM_0.10	feature_id[6].value > threshold=109.66666793823242
node_57: feature_name=HuIgG_0.30	feature_id[36].value > threshold=23562.0
Class: healthcare workers over 180 days after vaccination	
Rules_109	passed counts:2
node_0: feature_name=HuIgG_0.10	feature_id[2].value <= threshold=19120.166015625
node_1: feature_name=SARS.CoV.2.S1.RBD.mFc	feature_id[28].value > threshold=3773.8334350585938
node_9: feature_name=HuIgM_0.03	feature_id[14].value > threshold=-88.33333206176758
node_27: feature_name=hCoV.HKU1.S1_S2	feature_id[32].value <= threshold=42767.0
node_28: feature_name=HuIgG_0.03	feature_id[0].value > threshold=1005.0
node_38: feature_name=a-HuIgG_0.30	feature_id[4].value <= threshold=33596.333984375
node_39: feature_name=SARS.CoV.S1.HisTag	feature_id[18].value > threshold=400.3000030517578
node_43: feature_name=HuIgM_0.10	feature_id[6].value <= threshold=109.66666793823242
node_44: feature_name=MERS.CoV.NP	feature_id[15].value > threshold=-50.125
node_46: feature_name=hCoV.229E.S1_S2	feature_id[35].value <= threshold=42044.208984375

node_47: feature_name=HuIgM_0.30	feature_id[8].value <= threshold=1223.5
node_48: feature_name=SARS.CoV.S1.RBD.rFcTag	feature_id[16].value > threshold=27979.533203125
node_50: feature_name=Flu.H3N2.HA1+HA2	feature_id[38].value <= threshold=39487.333984375
Class: healthcare workers within 60 days after vaccination	
Rules_110	passed counts:2
node_0: feature_name=HuIgG_0.10	feature_id[2].value <= threshold=19120.166015625
node_1: feature_name=SARS.CoV.2.S1.RBD.mFc	feature_id[28].value > threshold=3773.8334350585938
node_9: feature_name=HuIgM_0.03	feature_id[14].value > threshold=-88.33333206176758
node_27: feature_name=hCoV.HKU1.S1_S2	feature_id[32].value <= threshold=42767.0
node_28: feature_name=HuIgG_0.03	feature_id[0].value > threshold=1005.0
node_38: feature_name=a-HuIgG_0.30	feature_id[4].value <= threshold=33596.333984375
node_39: feature_name=SARS.CoV.S1.HisTag	feature_id[18].value <= threshold=400.3000030517578
node_40: feature_name=HuIgA_0.03	feature_id[26].value <= threshold=448.0
Class: unvaccinated healthcare workers	
Rules_111	passed counts:2
node_0: feature_name=HuIgG_0.10	feature_id[2].value <= threshold=19120.166015625
node_1: feature_name=SARS.CoV.2.S1.RBD.mFc	feature_id[28].value > threshold=3773.8334350585938
node_9: feature_name=HuIgM_0.03	feature_id[14].value > threshold=-88.33333206176758
node_27: feature_name=hCoV.HKU1.S1_S2	feature_id[32].value <= threshold=42767.0
node_28: feature_name=HuIgG_0.03	feature_id[0].value <= threshold=1005.0
node_29: feature_name=SARS.CoV.2.S1.mFcTag	feature_id[21].value > threshold=34194.916015625
node_35: feature_name=a-HuIgG_0.10	feature_id[3].value > threshold=15372.16650390625
Class: healthcare workers over 180 days after vaccination	

Rules_112	passed counts:1
node_0: feature_name=HuIgG_0.10	feature_id[2].value > threshold=19120.166015625
node_96: feature_name=a-HuIgG_0.03	feature_id[7].value > threshold=19665.666015625
node_350: feature_name=HuIgM_0.03	feature_id[14].value <= threshold=-189.0
node_351: feature_name=hCoV.OC43.HE	feature_id[33].value <= threshold=15191.9169921875
Class: healthcare workers within 60 days after vaccination	
Rules_113	passed counts:1
node_0: feature_name=HuIgG_0.10	feature_id[2].value > threshold=19120.166015625
node_96: feature_name=a-HuIgG_0.03	feature_id[7].value <= threshold=19665.666015625
node_97: feature_name=MERS.CoV.S1.RBD.367.606.rFcTag	feature_id[5].value > threshold=3252.625
node_201: feature_name=SARS.CoV.2.S2	feature_id[29].value > threshold=37167.458984375
node_301: feature_name=SARS.CoV.2.S1.mFcTag	feature_id[21].value > threshold=53322.333984375
node_321: feature_name=a-MoIgA_0.03	feature_id[24].value <= threshold=212.0
node_322: feature_name=Flu.H3N2.HA1	feature_id[25].value > threshold=37503.517578125
node_330: feature_name=a-MoIgG_0.03	feature_id[12].value <= threshold=556.6666564941406
node_331: feature_name=hCoV.229E.S1_S2	feature_id[35].value <= threshold=47263.833984375
node_332: feature_name=a-HuIgG_0.10	feature_id[3].value > threshold=30393.166015625
node_336: feature_name=hCoV.NL63.S1_S2	feature_id[13].value <= threshold=53473.16796875
node_337: feature_name=hCoV.HKU1.NP	feature_id[11].value > threshold=16009.80029296875
Class: healthcare workers within 60 days after vaccination	
Rules_114	passed counts:1
node_0: feature_name=HuIgG_0.10	feature_id[2].value > threshold=19120.166015625

node_96: feature_name=a-HuIgG_0.03	feature_id[7].value <= threshold=19665.666015625
node_97: feature_name=MERS.CoV.S1.RBD.367.606.rFcTag	feature_id[5].value > threshold=3252.625
node_201: feature_name=SARS.CoV.2.S2	feature_id[29].value > threshold=37167.458984375
node_301: feature_name=SARS.CoV.2.S1.mFcTag	feature_id[21].value > threshold=53322.333984375
node_321: feature_name=a-MoIgA_0.03	feature_id[24].value <= threshold=212.0
node_322: feature_name=Flu.H3N2.HA1	feature_id[25].value > threshold=37503.517578125
node_330: feature_name=a-MoIgG_0.03	feature_id[12].value <= threshold=556.6666564941406
node_331: feature_name=hCoV.229E.S1_S2	feature_id[35].value <= threshold=47263.833984375
node_332: feature_name=a-HuIgG_0.10	feature_id[3].value > threshold=30393.166015625
node_336: feature_name=hCoV.NL63.S1_S2	feature_id[13].value <= threshold=53473.16796875
node_337: feature_name=hCoV.HKU1.NP	feature_id[11].value <= threshold=16009.80029296875
node_338: feature_name=hCoV.HKU1.S1_S2	feature_id[32].value <= threshold=41343.134765625
Class: healthcare workers within 60 days after vaccination	
Rules_115	passed counts:1
node_0: feature_name=HuIgG_0.10	feature_id[2].value > threshold=19120.166015625
node_96: feature_name=a-HuIgG_0.03	feature_id[7].value <= threshold=19665.666015625
node_97: feature_name=MERS.CoV.S1.RBD.367.606.rFcTag	feature_id[5].value > threshold=3252.625
node_201: feature_name=SARS.CoV.2.S2	feature_id[29].value > threshold=37167.458984375
node_301: feature_name=SARS.CoV.2.S1.mFcTag	feature_id[21].value > threshold=53322.333984375
node_321: feature_name=a-MoIgA_0.03	feature_id[24].value <= threshold=212.0
node_322: feature_name=Flu.H3N2.HA1	feature_id[25].value > threshold=37503.517578125
node_330: feature_name=a-MoIgG_0.03	feature_id[12].value <= threshold=556.6666564941406
node_331: feature_name=hCoV.229E.S1_S2	feature_id[35].value <= threshold=47263.833984375
node_332: feature_name=a-HuIgG_0.10	feature_id[3].value <= threshold=30393.166015625

node_333: feature_name=hCoV.OC43.HE	feature_id[33].value > threshold=25792.5830078125
Class: healthcare workers between 60 and 180 days after vaccination	
Rules_116	passed counts:1
node_0: feature_name=HuIgG_0.10	feature_id[2].value > threshold=19120.166015625
node_96: feature_name=a-HuIgG_0.03	feature_id[7].value <= threshold=19665.666015625
node_97: feature_name=MERS.CoV.S1.RBD.367.606.rFcTag	feature_id[5].value > threshold=3252.625
node_201: feature_name=SARS.CoV.2.S2	feature_id[29].value > threshold=37167.458984375
node_301: feature_name=SARS.CoV.2.S1.mFcTag	feature_id[21].value > threshold=53322.333984375
node_321: feature_name=a-MoIgA_0.03	feature_id[24].value <= threshold=212.0
node_322: feature_name=Flu.H3N2.HA1	feature_id[25].value <= threshold=37503.517578125
node_323: feature_name=hCoV.229E.S1_S2	feature_id[35].value <= threshold=29288.533203125
node_324: feature_name=a-MoIgG_0.03	feature_id[12].value > threshold=254.83333206176758
node_326: feature_name=Flu.H3N2.HA1	feature_id[25].value > threshold=32032.916015625
Class: unvaccinated healthcare workers	
Rules_117	passed counts:1
node_0: feature_name=HuIgG_0.10	feature_id[2].value > threshold=19120.166015625
node_96: feature_name=a-HuIgG_0.03	feature_id[7].value <= threshold=19665.666015625
node_97: feature_name=MERS.CoV.S1.RBD.367.606.rFcTag	feature_id[5].value > threshold=3252.625
node_201: feature_name=SARS.CoV.2.S2	feature_id[29].value > threshold=37167.458984375
node_301: feature_name=SARS.CoV.2.S1.mFcTag	feature_id[21].value > threshold=53322.333984375
node_321: feature_name=a-MoIgA_0.03	feature_id[24].value <= threshold=212.0
node_322: feature_name=Flu.H3N2.HA1	feature_id[25].value <= threshold=37503.517578125
node_323: feature_name=hCoV.229E.S1_S2	feature_id[35].value <= threshold=29288.533203125

node_324: feature_name=a-MoIgG_0.03	feature_id[12].value > threshold=254.83333206176758
node_326: feature_name=Flu.H3N2.HA1	feature_id[25].value <= threshold=32032.916015625
Class: healthcare workers between 60 and 180 days after vaccination	
Rules_118	passed counts:1
node_0: feature_name=HuIgG_0.10	feature_id[2].value > threshold=19120.166015625
node_96: feature_name=a-HuIgG_0.03	feature_id[7].value <= threshold=19665.666015625
node_97: feature_name=MERS.CoV.S1.RBD.367.606.rFcTag	feature_id[5].value > threshold=3252.625
node_201: feature_name=SARS.CoV.2.S2	feature_id[29].value > threshold=37167.458984375
node_301: feature_name=SARS.CoV.2.S1.mFcTag	feature_id[21].value > threshold=53322.333984375
node_321: feature_name=a-MoIgA_0.03	feature_id[24].value <= threshold=212.0
node_322: feature_name=Flu.H3N2.HA1	feature_id[25].value <= threshold=37503.517578125
node_323: feature_name=hCoV.229E.S1_S2	feature_id[35].value <= threshold=29288.533203125
node_324: feature_name=a-MoIgG_0.03	feature_id[12].value <= threshold=254.83333206176758
Class: healthcare workers over 180 days after vaccination	
Rules_119	passed counts:1
node_0: feature_name=HuIgG_0.10	feature_id[2].value > threshold=19120.166015625
node_96: feature_name=a-HuIgG_0.03	feature_id[7].value <= threshold=19665.666015625
node_97: feature_name=MERS.CoV.S1.RBD.367.606.rFcTag	feature_id[5].value > threshold=3252.625
node_201: feature_name=SARS.CoV.2.S2	feature_id[29].value > threshold=37167.458984375
node_301: feature_name=SARS.CoV.2.S1.mFcTag	feature_id[21].value <= threshold=53322.333984375
node_302: feature_name=MoIgG_0.03	feature_id[20].value > threshold=18.166666507720947
node_318: feature_name=Flu.B_Mal/.HA1+HA2	feature_id[22].value > threshold=54334.25
Class: healthcare workers between 60 and 180 days after vaccination	

Rules_120	passed counts:1
node_0: feature_name=HuIgG_0.10	feature_id[2].value > threshold=19120.166015625
node_96: feature_name=a-HuIgG_0.03	feature_id[7].value <= threshold=19665.666015625
node_97: feature_name=MERS.CoV.S1.RBD.367.606.rFcTag	feature_id[5].value > threshold=3252.625
node_201: feature_name=SARS.CoV.2.S2	feature_id[29].value > threshold=37167.458984375
node_301: feature_name=SARS.CoV.2.S1.mFcTag	feature_id[21].value <= threshold=53322.333984375
node_302: feature_name=MoIgG_0.03	feature_id[20].value <= threshold=18.166666507720947
node_303: feature_name=MoIgG_0.30	feature_id[34].value > threshold=7257.166748046875
node_315: feature_name=Flu.H3N2.HA1+HA2	feature_id[38].value <= threshold=52402.140625
Class: healthcare workers over 180 days after vaccination	
Rules_121	passed counts:1
node_0: feature_name=HuIgG_0.10	feature_id[2].value > threshold=19120.166015625
node_96: feature_name=a-HuIgG_0.03	feature_id[7].value <= threshold=19665.666015625
node_97: feature_name=MERS.CoV.S1.RBD.367.606.rFcTag	feature_id[5].value > threshold=3252.625
node_201: feature_name=SARS.CoV.2.S2	feature_id[29].value > threshold=37167.458984375
node_301: feature_name=SARS.CoV.2.S1.mFcTag	feature_id[21].value <= threshold=53322.333984375
node_302: feature_name=MoIgG_0.03	feature_id[20].value <= threshold=18.166666507720947
node_303: feature_name=MoIgG_0.30	feature_id[34].value <= threshold=7257.166748046875
node_304: feature_name=hCoV.NL63.S1_S2	feature_id[13].value <= threshold=52607.083984375
node_305: feature_name=HuIgG_0.30	feature_id[36].value <= threshold=35912.0
node_306: feature_name=hCoV.229E.S1	feature_id[30].value > threshold=31286.6669921875
node_308: feature_name=SARS.CoV.2.S2	feature_id[29].value <= threshold=41902.466796875
node_309: feature_name=Flu.B_Mal/.HA1+HA2	feature_id[22].value > threshold=39980.466796875

Class: unvaccinated healthcare workers	
Rules_122	passed counts:1
node_0: feature_name=HuIgG_0.10	feature_id[2].value > threshold=19120.166015625
node_96: feature_name=a-HuIgG_0.03	feature_id[7].value <= threshold=19665.666015625
node_97: feature_name=MERS.CoV.S1.RBD.367.606.rFcTag	feature_id[5].value > threshold=3252.625
node_201: feature_name=SARS.CoV.2.S2	feature_id[29].value > threshold=37167.458984375
node_301: feature_name=SARS.CoV.2.S1.mFcTag	feature_id[21].value <= threshold=53322.333984375
node_302: feature_name=MoIgG_0.03	feature_id[20].value <= threshold=18.166666507720947
node_303: feature_name=MoIgG_0.30	feature_id[34].value <= threshold=7257.166748046875
node_304: feature_name=hCoV.NL63.S1_S2	feature_id[13].value <= threshold=52607.083984375
node_305: feature_name=HuIgG_0.30	feature_id[36].value <= threshold=35912.0
node_306: feature_name=hCoV.229E.S1	feature_id[30].value > threshold=31286.6669921875
node_308: feature_name=SARS.CoV.2.S2	feature_id[29].value <= threshold=41902.466796875
node_309: feature_name=Flu.B_Mal/.HA1+HA2	feature_id[22].value <= threshold=39980.466796875
Class: healthcare workers within 60 days after vaccination	
Rules_123	passed counts:1
node_0: feature_name=HuIgG_0.10	feature_id[2].value > threshold=19120.166015625
node_96: feature_name=a-HuIgG_0.03	feature_id[7].value <= threshold=19665.666015625
node_97: feature_name=MERS.CoV.S1.RBD.367.606.rFcTag	feature_id[5].value > threshold=3252.625
node_201: feature_name=SARS.CoV.2.S2	feature_id[29].value <= threshold=37167.458984375
node_202: feature_name=SARS.CoV.2.Spike.RBD.His.Bac	feature_id[1].value > threshold=16030.75048828125
node_212: feature_name=a-MoIgA_0.30	feature_id[37].value > threshold=1303.0
node_224: feature_name=HuIgA_0.03	feature_id[26].value > threshold=25.83333396911621

node_276: feature_name=hCoV.OC43.NP	feature_id[23].value > threshold=3591.10009765625
node_290: feature_name=SARS.CoV.2.Spike.RBD.His.Bac	feature_id[1].value > threshold=33649.125
node_296: feature_name=HuIgM_0.03	feature_id[14].value <= threshold=-90.5
node_297: feature_name=hCoV.OC43.HE	feature_id[33].value > threshold=20650.0830078125
Class: healthcare workers between 60 and 180 days after vaccination	
Rules_124	passed counts:1
node_0: feature_name=HuIgG_0.10	feature_id[2].value > threshold=19120.166015625
node_96: feature_name=a-HuIgG_0.03	feature_id[7].value <= threshold=19665.666015625
node_97: feature_name=MERS.CoV.S1.RBD.367.606.rFcTag	feature_id[5].value > threshold=3252.625
node_201: feature_name=SARS.CoV.2.S2	feature_id[29].value <= threshold=37167.458984375
node_202: feature_name=SARS.CoV.2.Spike.RBD.His.Bac	feature_id[1].value > threshold=16030.75048828125
node_212: feature_name=a-MoIgA_0.30	feature_id[37].value > threshold=1303.0
node_224: feature_name=HuIgA_0.03	feature_id[26].value > threshold=25.83333396911621
node_276: feature_name=hCoV.OC43.NP	feature_id[23].value > threshold=3591.10009765625
node_290: feature_name=SARS.CoV.2.Spike.RBD.His.Bac	feature_id[1].value <= threshold=33649.125
node_291: feature_name=SARS.CoV.2.S1.mFcTag	feature_id[21].value > threshold=49171.599609375
node_293: feature_name=a-MoIgA_0.03	feature_id[24].value <= threshold=-139.66666412353516
Class: healthcare workers between 60 and 180 days after vaccination	
Rules_125	passed counts:1
node_0: feature_name=HuIgG_0.10	feature_id[2].value > threshold=19120.166015625
node_96: feature_name=a-HuIgG_0.03	feature_id[7].value <= threshold=19665.666015625
node_97: feature_name=MERS.CoV.S1.RBD.367.606.rFcTag	feature_id[5].value > threshold=3252.625
node_201: feature_name=SARS.CoV.2.S2	feature_id[29].value <= threshold=37167.458984375

node_202: feature_name=SARS.CoV.2.Spike.RBD.His.Bac	feature_id[1].value > threshold=16030.75048828125
node_212: feature_name=a-MoIgA_0.30	feature_id[37].value > threshold=1303.0
node_224: feature_name=HuIgA_0.03	feature_id[26].value > threshold=25.83333396911621
node_276: feature_name=hCoV.OC43.NP	feature_id[23].value <= threshold=3591.10009765625
node_277: feature_name=hCoV.229E.S1	feature_id[30].value > threshold=28616.0
node_287: feature_name=Flu.H3N2.HA1+HA2	feature_id[38].value > threshold=55544.548828125
Class: healthcare workers within 60 days after vaccination	
Rules_126	passed counts:1
node_0: feature_name=HuIgG_0.10	feature_id[2].value > threshold=19120.166015625
node_96: feature_name=a-HuIgG_0.03	feature_id[7].value <= threshold=19665.666015625
node_97: feature_name=MERS.CoV.S1.RBD.367.606.rFcTag	feature_id[5].value > threshold=3252.625
node_201: feature_name=SARS.CoV.2.S2	feature_id[29].value <= threshold=37167.458984375
node_202: feature_name=SARS.CoV.2.Spike.RBD.His.Bac	feature_id[1].value > threshold=16030.75048828125
node_212: feature_name=a-MoIgA_0.30	feature_id[37].value > threshold=1303.0
node_224: feature_name=HuIgA_0.03	feature_id[26].value > threshold=25.83333396911621
node_276: feature_name=hCoV.OC43.NP	feature_id[23].value <= threshold=3591.10009765625
node_277: feature_name=hCoV.229E.S1	feature_id[30].value <= threshold=28616.0
node_278: feature_name=a-MoIgA_0.03	feature_id[24].value > threshold=-30.66666603088379
node_282: feature_name=HuIgA_0.03	feature_id[26].value <= threshold=965.3333435058594
node_283: feature_name=HuIgG_0.03	feature_id[0].value > threshold=7378.66650390625
Class: healthcare workers within 60 days after vaccination	
Rules_127	passed counts:1
node_0: feature_name=HuIgG_0.10	feature_id[2].value > threshold=19120.166015625

node_96: feature_name=a-HuIgG_0.03	feature_id[7].value <= threshold=19665.666015625
node_97: feature_name=MERS.CoV.S1.RBD.367.606.rFcTag	feature_id[5].value > threshold=3252.625
node_201: feature_name=SARS.CoV.2.S2	feature_id[29].value <= threshold=37167.458984375
node_202: feature_name=SARS.CoV.2.Spike.RBD.His.Bac	feature_id[1].value > threshold=16030.75048828125
node_212: feature_name=a-MoIgA_0.30	feature_id[37].value > threshold=1303.0
node_224: feature_name=HuIgA_0.03	feature_id[26].value > threshold=25.83333396911621
node_276: feature_name=hCoV.OC43.NP	feature_id[23].value <= threshold=3591.10009765625
node_277: feature_name=hCoV.229E.S1	feature_id[30].value <= threshold=28616.0
node_278: feature_name=a-MoIgA_0.03	feature_id[24].value <= threshold=-30.66666603088379
node_279: feature_name=MERS.CoV.NP	feature_id[15].value > threshold=8498.916748046875
Class: healthcare workers between 60 and 180 days after vaccination	
Rules_128	passed counts:1
node_0: feature_name=HuIgG_0.10	feature_id[2].value > threshold=19120.166015625
node_96: feature_name=a-HuIgG_0.03	feature_id[7].value <= threshold=19665.666015625
node_97: feature_name=MERS.CoV.S1.RBD.367.606.rFcTag	feature_id[5].value > threshold=3252.625
node_201: feature_name=SARS.CoV.2.S2	feature_id[29].value <= threshold=37167.458984375
node_202: feature_name=SARS.CoV.2.Spike.RBD.His.Bac	feature_id[1].value > threshold=16030.75048828125
node_212: feature_name=a-MoIgA_0.30	feature_id[37].value > threshold=1303.0
node_224: feature_name=HuIgA_0.03	feature_id[26].value <= threshold=25.83333396911621
node_225: feature_name=Flu.B_Phu/.HA1+HA2	feature_id[19].value > threshold=54802.5
node_273: feature_name=hCoV.HKU1.S1_AA1.760	feature_id[39].value > threshold=45921.66796875
Class: healthcare workers within 60 days after vaccination	
Rules_129	passed counts:1

node_0: feature_name=HuIgG_0.10	feature_id[2].value > threshold=19120.166015625
node_96: feature_name=a-HuIgG_0.03	feature_id[7].value <= threshold=19665.666015625
node_97: feature_name=MERS.CoV.S1.RBD.367.606.rFcTag	feature_id[5].value > threshold=3252.625
node_201: feature_name=SARS.CoV.2.S2	feature_id[29].value <= threshold=37167.458984375
node_202: feature_name=SARS.CoV.2.Spike.RBD.His.Bac	feature_id[1].value > threshold=16030.75048828125
node_212: feature_name=a-MoIgA_0.30	feature_id[37].value > threshold=1303.0
node_224: feature_name=HuIgA_0.03	feature_id[26].value <= threshold=25.83333396911621
node_225: feature_name=Flu.B_Phu/.HA1+HA2	feature_id[19].value <= threshold=54802.5
node_226: feature_name=hCoV.HKU1.NP	feature_id[11].value > threshold=13460.3330078125
node_270: feature_name=Flu.H3N2.HA1	feature_id[25].value <= threshold=35417.583984375
Class: healthcare workers between 60 and 180 days after vaccination	
Rules_130	passed counts:1
node_0: feature_name=HuIgG_0.10	feature_id[2].value > threshold=19120.166015625
node_96: feature_name=a-HuIgG_0.03	feature_id[7].value <= threshold=19665.666015625
node_97: feature_name=MERS.CoV.S1.RBD.367.606.rFcTag	feature_id[5].value > threshold=3252.625
node_201: feature_name=SARS.CoV.2.S2	feature_id[29].value <= threshold=37167.458984375
node_202: feature_name=SARS.CoV.2.Spike.RBD.His.Bac	feature_id[1].value > threshold=16030.75048828125
node_212: feature_name=a-MoIgA_0.30	feature_id[37].value > threshold=1303.0
node_224: feature_name=HuIgA_0.03	feature_id[26].value <= threshold=25.83333396911621
node_225: feature_name=Flu.B_Phu/.HA1+HA2	feature_id[19].value <= threshold=54802.5
node_226: feature_name=hCoV.HKU1.NP	feature_id[11].value <= threshold=13460.3330078125
node_227: feature_name=MoIgG_0.30	feature_id[34].value > threshold=3760.5
node_229: feature_name=HuIgM_0.10	feature_id[6].value > threshold=-87.16666412353516
node_233: feature_name=a-HuIgG_0.10	feature_id[3].value > threshold=27244.3330078125

node_235: feature_name=SARS.CoV.S1.RBD.rFcTag	feature_id[16].value > threshold=20123.43359375
node_245: feature_name=hCoV.OC43.NP	feature_id[23].value > threshold=1775.816650390625
node_253: feature_name=SARS.CoV.S1.RBD.rFcTag	feature_id[16].value > threshold=21641.4169921875
node_255: feature_name=HuIgA_0.03	feature_id[26].value <= threshold=15.333333015441895
node_256: feature_name=Flu.H3N2.HA1	feature_id[25].value <= threshold=52674.0
node_257: feature_name=hCoV.HKU1.S1_S2	feature_id[32].value > threshold=37449.416015625
node_261: feature_name=SARS.CoV.2.S1+S2	feature_id[17].value > threshold=42043.224609375
node_263: feature_name=Flu.B_Mal/.HA1	feature_id[9].value > threshold=50467.166015625
Class: healthcare workers within 60 days after vaccination	
Rules_131	passed counts:1
node_0: feature_name=HuIgG_0.10	feature_id[2].value > threshold=19120.166015625
node_96: feature_name=a-HuIgG_0.03	feature_id[7].value <= threshold=19665.666015625
node_97: feature_name=MERS.CoV.S1.RBD.367.606.rFcTag	feature_id[5].value > threshold=3252.625
node_201: feature_name=SARS.CoV.2.S2	feature_id[29].value <= threshold=37167.458984375
node_202: feature_name=SARS.CoV.2.Spike.RBD.His.Bac	feature_id[1].value > threshold=16030.75048828125
node_212: feature_name=a-MoIgA_0.30	feature_id[37].value > threshold=1303.0
node_224: feature_name=HuIgA_0.03	feature_id[26].value <= threshold=25.83333396911621
node_225: feature_name=Flu.B_Phu/.HA1+HA2	feature_id[19].value <= threshold=54802.5
node_226: feature_name=hCoV.HKU1.NP	feature_id[11].value <= threshold=13460.3330078125
node_227: feature_name=MoIgG_0.30	feature_id[34].value > threshold=3760.5
node_229: feature_name=HuIgM_0.10	feature_id[6].value > threshold=-87.16666412353516
node_233: feature_name=a-HuIgG_0.10	feature_id[3].value > threshold=27244.3330078125
node_235: feature_name=SARS.CoV.S1.RBD.rFcTag	feature_id[16].value > threshold=20123.43359375
node_245: feature_name=hCoV.OC43.NP	feature_id[23].value > threshold=1775.816650390625

node_253: feature_name=SARS.CoV.S1.RBD.rFcTag	feature_id[16].value > threshold=21641.4169921875
node_255: feature_name=HuIgA_0.03	feature_id[26].value <= threshold=15.333333015441895
node_256: feature_name=Flu.H3N2.HA1	feature_id[25].value <= threshold=52674.0
node_257: feature_name=hCoV.HKU1.S1_S2	feature_id[32].value > threshold=37449.416015625
node_261: feature_name=SARS.CoV.2.S1+S2	feature_id[17].value > threshold=42043.224609375
node_263: feature_name=Flu.B_Mal/.HA1	feature_id[9].value <= threshold=50467.166015625
node_264: feature_name=hCoV.OC43.HE	feature_id[33].value > threshold=29465.6669921875
Class: healthcare workers within 60 days after vaccination	
Rules_132	passed counts:1
node_0: feature_name=HuIgG_0.10	feature_id[2].value > threshold=19120.166015625
node_96: feature_name=a-HuIgG_0.03	feature_id[7].value <= threshold=19665.666015625
node_97: feature_name=MERS.CoV.S1.RBD.367.606.rFcTag	feature_id[5].value > threshold=3252.625
node_201: feature_name=SARS.CoV.2.S2	feature_id[29].value <= threshold=37167.458984375
node_202: feature_name=SARS.CoV.2.Spike.RBD.His.Bac	feature_id[1].value > threshold=16030.75048828125
node_212: feature_name=a-MoIgA_0.30	feature_id[37].value > threshold=1303.0
node_224: feature_name=HuIgA_0.03	feature_id[26].value <= threshold=25.83333396911621
node_225: feature_name=Flu.B_Phu/.HA1+HA2	feature_id[19].value <= threshold=54802.5
node_226: feature_name=hCoV.HKU1.NP	feature_id[11].value <= threshold=13460.3330078125
node_227: feature_name=MoIgG_0.30	feature_id[34].value > threshold=3760.5
node_229: feature_name=HuIgM_0.10	feature_id[6].value > threshold=-87.16666412353516
node_233: feature_name=a-HuIgG_0.10	feature_id[3].value > threshold=27244.3330078125
node_235: feature_name=SARS.CoV.S1.RBD.rFcTag	feature_id[16].value > threshold=20123.43359375
node_245: feature_name=hCoV.OC43.NP	feature_id[23].value > threshold=1775.816650390625
node_253: feature_name=SARS.CoV.S1.RBD.rFcTag	feature_id[16].value > threshold=21641.4169921875

node_255: feature_name=HuIgA_0.03	feature_id[26].value <= threshold=15.333333015441895
node_256: feature_name=Flu.H3N2.HA1	feature_id[25].value <= threshold=52674.0
node_257: feature_name=hCoV.HKU1.S1_S2	feature_id[32].value <= threshold=37449.416015625
node_258: feature_name=Flu.B_Mal/.HA1	feature_id[9].value <= threshold=27139.75
Class: healthcare workers between 60 and 180 days after vaccination	
Rules_133	passed counts:1
node_0: feature_name=HuIgG_0.10	feature_id[2].value > threshold=19120.166015625
node_96: feature_name=a-HuIgG_0.03	feature_id[7].value <= threshold=19665.666015625
node_97: feature_name=MERS.CoV.S1.RBD.367.606.rFcTag	feature_id[5].value > threshold=3252.625
node_201: feature_name=SARS.CoV.2.S2	feature_id[29].value <= threshold=37167.458984375
node_202: feature_name=SARS.CoV.2.Spike.RBD.His.Bac	feature_id[1].value > threshold=16030.75048828125
node_212: feature_name=a-MoIgA_0.30	feature_id[37].value > threshold=1303.0
node_224: feature_name=HuIgA_0.03	feature_id[26].value <= threshold=25.83333396911621
node_225: feature_name=Flu.B_Phu/.HA1+HA2	feature_id[19].value <= threshold=54802.5
node_226: feature_name=hCoV.HKU1.NP	feature_id[11].value <= threshold=13460.3330078125
node_227: feature_name=MoIgG_0.30	feature_id[34].value > threshold=3760.5
node_229: feature_name=HuIgM_0.10	feature_id[6].value > threshold=-87.16666412353516
node_233: feature_name=a-HuIgG_0.10	feature_id[3].value > threshold=27244.3330078125
node_235: feature_name=SARS.CoV.S1.RBD.rFcTag	feature_id[16].value > threshold=20123.43359375
node_245: feature_name=hCoV.OC43.NP	feature_id[23].value <= threshold=1775.816650390625
node_246: feature_name=HuIgA_0.03	feature_id[26].value <= threshold=-10.333333492279053
node_247: feature_name=SARS.CoV.2.S1.RBD.mFc	feature_id[28].value > threshold=53114.833984375
node_249: feature_name=HuIgG_0.10	feature_id[2].value > threshold=24164.3330078125
Class: unvaccinated healthcare workers	

Rules_134	passed counts:1
node_0: feature_name=HuIgG_0.10	feature_id[2].value > threshold=19120.166015625
node_96: feature_name=a-HuIgG_0.03	feature_id[7].value <= threshold=19665.666015625
node_97: feature_name=MERS.CoV.S1.RBD.367.606.rFcTag	feature_id[5].value > threshold=3252.625
node_201: feature_name=SARS.CoV.2.S2	feature_id[29].value <= threshold=37167.458984375
node_202: feature_name=SARS.CoV.2.Spike.RBD.His.Bac	feature_id[1].value > threshold=16030.75048828125
node_212: feature_name=a-MoIgA_0.30	feature_id[37].value > threshold=1303.0
node_224: feature_name=HuIgA_0.03	feature_id[26].value <= threshold=25.83333396911621
node_225: feature_name=Flu.B_Phu/.HA1+HA2	feature_id[19].value <= threshold=54802.5
node_226: feature_name=hCoV.HKU1.NP	feature_id[11].value <= threshold=13460.3330078125
node_227: feature_name=MoIgG_0.30	feature_id[34].value > threshold=3760.5
node_229: feature_name=HuIgM_0.10	feature_id[6].value > threshold=-87.16666412353516
node_233: feature_name=a-HuIgG_0.10	feature_id[3].value > threshold=27244.3330078125
node_235: feature_name=SARS.CoV.S1.RBD.rFcTag	feature_id[16].value <= threshold=20123.43359375
node_236: feature_name=MERS.CoV.S1.RBD.367.606.rFcTag	feature_id[5].value > threshold=4326.625
node_238: feature_name=SARS.CoV.NP	feature_id[31].value > threshold=2842.375
node_240: feature_name=Flu.H1N1.HA1+HA2	feature_id[10].value <= threshold=53053.666015625
node_241: feature_name=MoIgG_0.03	feature_id[20].value <= threshold=-68.16666793823242
Class: healthcare workers within 60 days after vaccination	
Rules_135	passed counts:1
node_0: feature_name=HuIgG_0.10	feature_id[2].value > threshold=19120.166015625
node_96: feature_name=a-HuIgG_0.03	feature_id[7].value <= threshold=19665.666015625
node_97: feature_name=MERS.CoV.S1.RBD.367.606.rFcTag	feature_id[5].value > threshold=3252.625

node_201: feature_name=SARS.CoV.2.S2	feature_id[29].value <= threshold=37167.458984375
node_202: feature_name=SARS.CoV.2.Spike.RBD.His.Bac	feature_id[1].value > threshold=16030.75048828125
node_212: feature_name=a-MoIgA_0.30	feature_id[37].value > threshold=1303.0
node_224: feature_name=HuIgA_0.03	feature_id[26].value <= threshold=25.83333396911621
node_225: feature_name=Flu.B_Phu/.HA1+HA2	feature_id[19].value <= threshold=54802.5
node_226: feature_name=hCoV.HKU1.NP	feature_id[11].value <= threshold=13460.3330078125
node_227: feature_name=MoIgG_0.30	feature_id[34].value > threshold=3760.5
node_229: feature_name=HuIgM_0.10	feature_id[6].value <= threshold=-87.16666412353516
node_230: feature_name=hCoV.229E.S1	feature_id[30].value > threshold=39693.333984375
Class: healthcare workers over 180 days after vaccination	
Rules_136	passed counts:1
node_0: feature_name=HuIgG_0.10	feature_id[2].value > threshold=19120.166015625
node_96: feature_name=a-HuIgG_0.03	feature_id[7].value <= threshold=19665.666015625
node_97: feature_name=MERS.CoV.S1.RBD.367.606.rFcTag	feature_id[5].value > threshold=3252.625
node_201: feature_name=SARS.CoV.2.S2	feature_id[29].value <= threshold=37167.458984375
node_202: feature_name=SARS.CoV.2.Spike.RBD.His.Bac	feature_id[1].value > threshold=16030.75048828125
node_212: feature_name=a-MoIgA_0.30	feature_id[37].value <= threshold=1303.0
node_213: feature_name=MERS.CoV.NP	feature_id[15].value > threshold=898.7833251953125
node_217: feature_name=hCoV.NL63.S1_S2	feature_id[13].value > threshold=30153.3330078125
node_219: feature_name=SARS.CoV.S1.HisTag	feature_id[18].value <= threshold=4305.2916259765625
node_220: feature_name=HuIgG_0.30	feature_id[36].value > threshold=36339.833984375
Class: healthcare workers within 60 days after vaccination	
Rules_137	passed counts:1

node_0: feature_name=HuIgG_0.10	feature_id[2].value > threshold=19120.166015625
node_96: feature_name=a-HuIgG_0.03	feature_id[7].value <= threshold=19665.666015625
node_97: feature_name=MERS.CoV.S1.RBD.367.606.rFcTag	feature_id[5].value > threshold=3252.625
node_201: feature_name=SARS.CoV.2.S2	feature_id[29].value <= threshold=37167.458984375
node_202: feature_name=SARS.CoV.2.Spike.RBD.His.Bac	feature_id[1].value <= threshold=16030.75048828125
node_203: feature_name=a-MoIgG_0.10	feature_id[27].value > threshold=2117.9999389648438
node_207: feature_name=SARS.CoV.S1.HisTag	feature_id[18].value <= threshold=1072.50830078125
node_208: feature_name=Flu.H3N2.HA1+HA2	feature_id[38].value > threshold=54173.125
Class: healthcare workers within 60 days after vaccination	
Rules_138	passed counts:1
node_0: feature_name=HuIgG_0.10	feature_id[2].value > threshold=19120.166015625
node_96: feature_name=a-HuIgG_0.03	feature_id[7].value <= threshold=19665.666015625
node_97: feature_name=MERS.CoV.S1.RBD.367.606.rFcTag	feature_id[5].value > threshold=3252.625
node_201: feature_name=SARS.CoV.2.S2	feature_id[29].value <= threshold=37167.458984375
node_202: feature_name=SARS.CoV.2.Spike.RBD.His.Bac	feature_id[1].value <= threshold=16030.75048828125
node_203: feature_name=a-MoIgG_0.10	feature_id[27].value <= threshold=2117.9999389648438
node_204: feature_name=Flu.H3N2.HA1+HA2	feature_id[38].value <= threshold=44847.720703125
Class: healthcare workers between 60 and 180 days after vaccination	
Rules_139	passed counts:1
node_0: feature_name=HuIgG_0.10	feature_id[2].value > threshold=19120.166015625
node_96: feature_name=a-HuIgG_0.03	feature_id[7].value <= threshold=19665.666015625
node_97: feature_name=MERS.CoV.S1.RBD.367.606.rFcTag	feature_id[5].value <= threshold=3252.625
node_98: feature_name=SARS.CoV.2.S2	feature_id[29].value > threshold=9506.32470703125

node_116: feature_name=Flu.H1N1.HA1+HA2	feature_id[10].value > threshold=54065.41796875
node_194: feature_name=MERS.CoV.S1.RBD.367.606.rFcTag	feature_id[5].value > threshold=2551.3333740234375
node_198: feature_name=HuIgA_0.03	feature_id[26].value > threshold=884.3333129882812
Class: healthcare workers within 60 days after vaccination	
Rules_140	passed counts:1
node_0: feature_name=HuIgG_0.10	feature_id[2].value > threshold=19120.166015625
node_96: feature_name=a-HuIgG_0.03	feature_id[7].value <= threshold=19665.666015625
node_97: feature_name=MERS.CoV.S1.RBD.367.606.rFcTag	feature_id[5].value <= threshold=3252.625
node_98: feature_name=SARS.CoV.2.S2	feature_id[29].value > threshold=9506.32470703125
node_116: feature_name=Flu.H1N1.HA1+HA2	feature_id[10].value > threshold=54065.41796875
node_194: feature_name=MERS.CoV.S1.RBD.367.606.rFcTag	feature_id[5].value <= threshold=2551.3333740234375
node_195: feature_name=a-HuIgG_0.03	feature_id[7].value > threshold=17733.50048828125
Class: healthcare workers over 180 days after vaccination	
Rules_141	passed counts:1
node_0: feature_name=HuIgG_0.10	feature_id[2].value > threshold=19120.166015625
node_96: feature_name=a-HuIgG_0.03	feature_id[7].value <= threshold=19665.666015625
node_97: feature_name=MERS.CoV.S1.RBD.367.606.rFcTag	feature_id[5].value <= threshold=3252.625
node_98: feature_name=SARS.CoV.2.S2	feature_id[29].value > threshold=9506.32470703125
node_116: feature_name=Flu.H1N1.HA1+HA2	feature_id[10].value <= threshold=54065.41796875
node_117: feature_name=SARS.CoV.S1.RBD.rFcTag	feature_id[16].value > threshold=24639.0
node_167: feature_name=hCoV.OC43.HE	feature_id[33].value <= threshold=32466.5830078125
node_168: feature_name=HuIgM_0.10	feature_id[6].value > threshold=22.833333015441895
node_178: feature_name=a-MoIgA_0.03	feature_id[24].value > threshold=-34.333333015441895

node_184: feature_name=HuIgG_0.10	feature_id[2].value > threshold=21401.166015625
node_188: feature_name=SARS.CoV.2.S1.mFcTag	feature_id[21].value <= threshold=51001.0
node_189: feature_name=hCoV.OC43.HE	feature_id[33].value > threshold=18835.1337890625
Class: healthcare workers over 180 days after vaccination	
Rules_142	passed counts:1
node_0: feature_name=HuIgG_0.10	feature_id[2].value > threshold=19120.166015625
node_96: feature_name=a-HuIgG_0.03	feature_id[7].value <= threshold=19665.666015625
node_97: feature_name=MERS.CoV.S1.RBD.367.606.rFcTag	feature_id[5].value <= threshold=3252.625
node_98: feature_name=SARS.CoV.2.S2	feature_id[29].value > threshold=9506.32470703125
node_116: feature_name=Flu.H1N1.HA1+HA2	feature_id[10].value <= threshold=54065.41796875
node_117: feature_name=SARS.CoV.S1.RBD.rFcTag	feature_id[16].value > threshold=24639.0
node_167: feature_name=hCoV.OC43.HE	feature_id[33].value <= threshold=32466.5830078125
node_168: feature_name=HuIgM_0.10	feature_id[6].value > threshold=22.833333015441895
node_178: feature_name=a-MoIgA_0.03	feature_id[24].value > threshold=-34.333333015441895
node_184: feature_name=HuIgG_0.10	feature_id[2].value > threshold=21401.166015625
node_188: feature_name=SARS.CoV.2.S1.mFcTag	feature_id[21].value <= threshold=51001.0
node_189: feature_name=hCoV.OC43.HE	feature_id[33].value <= threshold=18835.1337890625
Class: healthcare workers within 60 days after vaccination	
Rules_143	passed counts:1
node_0: feature_name=HuIgG_0.10	feature_id[2].value > threshold=19120.166015625
node_96: feature_name=a-HuIgG_0.03	feature_id[7].value <= threshold=19665.666015625
node_97: feature_name=MERS.CoV.S1.RBD.367.606.rFcTag	feature_id[5].value <= threshold=3252.625
node_98: feature_name=SARS.CoV.2.S2	feature_id[29].value > threshold=9506.32470703125

node_116: feature_name=Flu.H1N1.HA1+HA2	feature_id[10].value <= threshold=54065.41796875
node_117: feature_name=SARS.CoV.S1.RBD.rFcTag	feature_id[16].value > threshold=24639.0
node_167: feature_name=hCoV.OC43.HE	feature_id[33].value <= threshold=32466.5830078125
node_168: feature_name=HuIgM_0.10	feature_id[6].value > threshold=22.833333015441895
node_178: feature_name=a-MoIgA_0.03	feature_id[24].value > threshold=-34.333333015441895
node_184: feature_name=HuIgG_0.10	feature_id[2].value <= threshold=21401.166015625
node_185: feature_name=SARS.CoV.S1.HisTag	feature_id[18].value <= threshold=7929.375244140625
Class: healthcare workers within 60 days after vaccination	
Rules_144	passed counts:1
node_0: feature_name=HuIgG_0.10	feature_id[2].value > threshold=19120.166015625
node_96: feature_name=a-HuIgG_0.03	feature_id[7].value <= threshold=19665.666015625
node_97: feature_name=MERS.CoV.S1.RBD.367.606.rFcTag	feature_id[5].value <= threshold=3252.625
node_98: feature_name=SARS.CoV.2.S2	feature_id[29].value > threshold=9506.32470703125
node_116: feature_name=Flu.H1N1.HA1+HA2	feature_id[10].value <= threshold=54065.41796875
node_117: feature_name=SARS.CoV.S1.RBD.rFcTag	feature_id[16].value > threshold=24639.0
node_167: feature_name=hCoV.OC43.HE	feature_id[33].value <= threshold=32466.5830078125
node_168: feature_name=HuIgM_0.10	feature_id[6].value > threshold=22.833333015441895
node_178: feature_name=a-MoIgA_0.03	feature_id[24].value <= threshold=-34.333333015441895
node_179: feature_name=Flu.H3N2.HA1+HA2	feature_id[38].value <= threshold=54266.91796875
node_180: feature_name=a-HuIgG_0.03	feature_id[7].value <= threshold=11043.33349609375
Class: healthcare workers within 60 days after vaccination	
Rules_145	passed counts:1
node_0: feature_name=HuIgG_0.10	feature_id[2].value > threshold=19120.166015625

node_96: feature_name=a-HuIgG_0.03	feature_id[7].value <= threshold=19665.666015625
node_97: feature_name=MERS.CoV.S1.RBD.367.606.rFcTag	feature_id[5].value <= threshold=3252.625
node_98: feature_name=SARS.CoV.2.S2	feature_id[29].value > threshold=9506.32470703125
node_116: feature_name=Flu.H1N1.HA1+HA2	feature_id[10].value <= threshold=54065.41796875
node_117: feature_name=SARS.CoV.S1.RBD.rFcTag	feature_id[16].value > threshold=24639.0
node_167: feature_name=hCoV.OC43.HE	feature_id[33].value <= threshold=32466.5830078125
node_168: feature_name=HuIgM_0.10	feature_id[6].value <= threshold=22.833333015441895
node_169: feature_name=SARS.CoV.2.S1.mFcTag	feature_id[21].value <= threshold=54847.25
node_170: feature_name=a-MoIgG_0.03	feature_id[12].value > threshold=2342.8333740234375
node_172: feature_name=HuIgM_0.03	feature_id[14].value <= threshold=-66.33333206176758
Class: healthcare workers between 60 and 180 days after vaccination	
Rules_146	passed counts:1
node_0: feature_name=HuIgG_0.10	feature_id[2].value > threshold=19120.166015625
node_96: feature_name=a-HuIgG_0.03	feature_id[7].value <= threshold=19665.666015625
node_97: feature_name=MERS.CoV.S1.RBD.367.606.rFcTag	feature_id[5].value <= threshold=3252.625
node_98: feature_name=SARS.CoV.2.S2	feature_id[29].value > threshold=9506.32470703125
node_116: feature_name=Flu.H1N1.HA1+HA2	feature_id[10].value <= threshold=54065.41796875
node_117: feature_name=SARS.CoV.S1.RBD.rFcTag	feature_id[16].value <= threshold=24639.0
node_118: feature_name=SARS.CoV.2.Spike.RBD.His.Bac	feature_id[1].value > threshold=23841.4169921875
node_128: feature_name=SARS.CoV.2.S1+S2	feature_id[17].value > threshold=41778.25
node_138: feature_name=hCoV.NL63.S1_S2	feature_id[13].value > threshold=35816.083984375
node_146: feature_name=a-MoIgG_0.10	feature_id[27].value > threshold=3760.5
node_156: feature_name=a-HuIgG_0.03	feature_id[7].value > threshold=9740.8330078125
node_158: feature_name=a-HuIgG_0.30	feature_id[4].value > threshold=42960.0

node_160: feature_name=hCoV.HKU1.NP	feature_id[11].value > threshold=3927.666748046875
node_162: feature_name=HuIgA_0.03	feature_id[26].value > threshold=595.6666870117188
node_164: feature_name=hCoV.HKU1.S1_S2	feature_id[32].value > threshold=49940.333984375
Class: healthcare workers within 60 days after vaccination	
Rules_147	passed counts:1
node_0: feature_name=HuIgG_0.10	feature_id[2].value > threshold=19120.166015625
node_96: feature_name=a-HuIgG_0.03	feature_id[7].value <= threshold=19665.666015625
node_97: feature_name=MERS.CoV.S1.RBD.367.606.rFcTag	feature_id[5].value <= threshold=3252.625
node_98: feature_name=SARS.CoV.2.S2	feature_id[29].value > threshold=9506.32470703125
node_116: feature_name=Flu.H1N1.HA1+HA2	feature_id[10].value <= threshold=54065.41796875
node_117: feature_name=SARS.CoV.S1.RBD.rFcTag	feature_id[16].value <= threshold=24639.0
node_118: feature_name=SARS.CoV.2.Spike.RBD.His.Bac	feature_id[1].value > threshold=23841.4169921875
node_128: feature_name=SARS.CoV.2.S1+S2	feature_id[17].value > threshold=41778.25
node_138: feature_name=hCoV.NL63.S1_S2	feature_id[13].value > threshold=35816.083984375
node_146: feature_name=a-MoIgG_0.10	feature_id[27].value <= threshold=3760.5
node_147: feature_name=SARS.CoV.S1.HisTag	feature_id[18].value > threshold=3759.5833740234375
node_149: feature_name=SARS.CoV.S1.HisTag	feature_id[18].value > threshold=4886.25
node_151: feature_name=HuIgM_0.30	feature_id[8].value <= threshold=518.0000152587891
node_152: feature_name=a-HuIgG_0.03	feature_id[7].value <= threshold=10986.3330078125
Class: healthcare workers between 60 and 180 days after vaccination	
Rules_148	passed counts:1
node_0: feature_name=HuIgG_0.10	feature_id[2].value > threshold=19120.166015625
node_96: feature_name=a-HuIgG_0.03	feature_id[7].value <= threshold=19665.666015625

node_97: feature_name=MERS.CoV.S1.RBD.367.606.rFcTag	feature_id[5].value <= threshold=3252.625
node_98: feature_name=SARS.CoV.2.S2	feature_id[29].value > threshold=9506.32470703125
node_116: feature_name=Flu.H1N1.HA1+HA2	feature_id[10].value <= threshold=54065.41796875
node_117: feature_name=SARS.CoV.S1.RBD.rFcTag	feature_id[16].value <= threshold=24639.0
node_118: feature_name=SARS.CoV.2.Spike.RBD.His.Bac	feature_id[1].value > threshold=23841.4169921875
node_128: feature_name=SARS.CoV.2.S1+S2	feature_id[17].value > threshold=41778.25
node_138: feature_name=hCoV.NL63.S1_S2	feature_id[13].value <= threshold=35816.083984375
node_139: feature_name=Flu.B_Mal/.HA1	feature_id[9].value > threshold=46360.66796875
node_143: feature_name=SARS.CoV.S1.RBD.rFcTag	feature_id[16].value > threshold=16387.74951171875
Class: healthcare workers between 60 and 180 days after vaccination	
Rules_149	passed counts:1
node_0: feature_name=HuIgG_0.10	feature_id[2].value > threshold=19120.166015625
node_96: feature_name=a-HuIgG_0.03	feature_id[7].value <= threshold=19665.666015625
node_97: feature_name=MERS.CoV.S1.RBD.367.606.rFcTag	feature_id[5].value <= threshold=3252.625
node_98: feature_name=SARS.CoV.2.S2	feature_id[29].value > threshold=9506.32470703125
node_116: feature_name=Flu.H1N1.HA1+HA2	feature_id[10].value <= threshold=54065.41796875
node_117: feature_name=SARS.CoV.S1.RBD.rFcTag	feature_id[16].value <= threshold=24639.0
node_118: feature_name=SARS.CoV.2.Spike.RBD.His.Bac	feature_id[1].value > threshold=23841.4169921875
node_128: feature_name=SARS.CoV.2.S1+S2	feature_id[17].value > threshold=41778.25
node_138: feature_name=hCoV.NL63.S1_S2	feature_id[13].value <= threshold=35816.083984375
node_139: feature_name=Flu.B_Mal/.HA1	feature_id[9].value > threshold=46360.66796875
node_143: feature_name=SARS.CoV.S1.RBD.rFcTag	feature_id[16].value <= threshold=16387.74951171875
Class: healthcare workers over 180 days after vaccination	

Rules_150	passed counts:1
node_0: feature_name=HuIgG_0.10	feature_id[2].value > threshold=19120.166015625
node_96: feature_name=a-HuIgG_0.03	feature_id[7].value <= threshold=19665.666015625
node_97: feature_name=MERS.CoV.S1.RBD.367.606.rFcTag	feature_id[5].value <= threshold=3252.625
node_98: feature_name=SARS.CoV.2.S2	feature_id[29].value > threshold=9506.32470703125
node_116: feature_name=Flu.H1N1.HA1+HA2	feature_id[10].value <= threshold=54065.41796875
node_117: feature_name=SARS.CoV.S1.RBD.rFcTag	feature_id[16].value <= threshold=24639.0
node_118: feature_name=SARS.CoV.2.Spike.RBD.His.Bac	feature_id[1].value > threshold=23841.4169921875
node_128: feature_name=SARS.CoV.2.S1+S2	feature_id[17].value > threshold=41778.25
node_138: feature_name=hCoV.NL63.S1_S2	feature_id[13].value <= threshold=35816.083984375
node_139: feature_name=Flu.B_Mal/.HA1	feature_id[9].value <= threshold=46360.66796875
node_140: feature_name=MERS.CoV.NP	feature_id[15].value <= threshold=809.8333129882812
Class: healthcare workers between 60 and 180 days after vaccination	
Rules_151	passed counts:1
node_0: feature_name=HuIgG_0.10	feature_id[2].value > threshold=19120.166015625
node_96: feature_name=a-HuIgG_0.03	feature_id[7].value <= threshold=19665.666015625
node_97: feature_name=MERS.CoV.S1.RBD.367.606.rFcTag	feature_id[5].value <= threshold=3252.625
node_98: feature_name=SARS.CoV.2.S2	feature_id[29].value > threshold=9506.32470703125
node_116: feature_name=Flu.H1N1.HA1+HA2	feature_id[10].value <= threshold=54065.41796875
node_117: feature_name=SARS.CoV.S1.RBD.rFcTag	feature_id[16].value <= threshold=24639.0
node_118: feature_name=SARS.CoV.2.Spike.RBD.His.Bac	feature_id[1].value > threshold=23841.4169921875
node_128: feature_name=SARS.CoV.2.S1+S2	feature_id[17].value <= threshold=41778.25
node_129: feature_name=SARS.CoV.S1.HisTag	feature_id[18].value > threshold=3569.2833251953125
node_133: feature_name=Flu.B_Mal/.HA1+HA2	feature_id[22].value > threshold=47613.583984375

node_135: feature_name=SARS.CoV.2.Spike.RBD.His.Bac	feature_id[1].value <= threshold=27719.5087890625
Class: healthcare workers between 60 and 180 days after vaccination	
Rules_152	passed counts:1
node_0: feature_name=HuIgG_0.10	feature_id[2].value > threshold=19120.166015625
node_96: feature_name=a-HuIgG_0.03	feature_id[7].value <= threshold=19665.666015625
node_97: feature_name=MERS.CoV.S1.RBD.367.606.rFcTag	feature_id[5].value <= threshold=3252.625
node_98: feature_name=SARS.CoV.2.S2	feature_id[29].value > threshold=9506.32470703125
node_116: feature_name=Flu.H1N1.HA1+HA2	feature_id[10].value <= threshold=54065.41796875
node_117: feature_name=SARS.CoV.S1.RBD.rFcTag	feature_id[16].value <= threshold=24639.0
node_118: feature_name=SARS.CoV.2.Spike.RBD.His.Bac	feature_id[1].value > threshold=23841.4169921875
node_128: feature_name=SARS.CoV.2.S1+S2	feature_id[17].value <= threshold=41778.25
node_129: feature_name=SARS.CoV.S1.HisTag	feature_id[18].value <= threshold=3569.2833251953125
node_130: feature_name=SARS.CoV.2.S2	feature_id[29].value > threshold=26478.916015625
Class: healthcare workers within 60 days after vaccination	
Rules_153	passed counts:1
node_0: feature_name=HuIgG_0.10	feature_id[2].value > threshold=19120.166015625
node_96: feature_name=a-HuIgG_0.03	feature_id[7].value <= threshold=19665.666015625
node_97: feature_name=MERS.CoV.S1.RBD.367.606.rFcTag	feature_id[5].value <= threshold=3252.625
node_98: feature_name=SARS.CoV.2.S2	feature_id[29].value > threshold=9506.32470703125
node_116: feature_name=Flu.H1N1.HA1+HA2	feature_id[10].value <= threshold=54065.41796875
node_117: feature_name=SARS.CoV.S1.RBD.rFcTag	feature_id[16].value <= threshold=24639.0
node_118: feature_name=SARS.CoV.2.Spike.RBD.His.Bac	feature_id[1].value <= threshold=23841.4169921875
node_119: feature_name=SARS.CoV.2.S2	feature_id[29].value > threshold=49775.806640625

Class: unvaccinated healthcare workers	
Rules_154	passed counts:1
node_0: feature_name=HuIgG_0.10	feature_id[2].value > threshold=19120.166015625
node_96: feature_name=a-HuIgG_0.03	feature_id[7].value <= threshold=19665.666015625
node_97: feature_name=MERS.CoV.S1.RBD.367.606.rFcTag	feature_id[5].value <= threshold=3252.625
node_98: feature_name=SARS.CoV.2.S2	feature_id[29].value > threshold=9506.32470703125
node_116: feature_name=Flu.H1N1.HA1+HA2	feature_id[10].value <= threshold=54065.41796875
node_117: feature_name=SARS.CoV.S1.RBD.rFcTag	feature_id[16].value <= threshold=24639.0
node_118: feature_name=SARS.CoV.2.Spike.RBD.His.Bac	feature_id[1].value <= threshold=23841.4169921875
node_119: feature_name=SARS.CoV.2.S2	feature_id[29].value <= threshold=49775.806640625
node_120: feature_name=MERS.CoV.NP	feature_id[15].value > threshold=8014.466796875
Class: healthcare workers between 60 and 180 days after vaccination	
Rules_155	passed counts:1
node_0: feature_name=HuIgG_0.10	feature_id[2].value > threshold=19120.166015625
node_96: feature_name=a-HuIgG_0.03	feature_id[7].value <= threshold=19665.666015625
node_97: feature_name=MERS.CoV.S1.RBD.367.606.rFcTag	feature_id[5].value <= threshold=3252.625
node_98: feature_name=SARS.CoV.2.S2	feature_id[29].value > threshold=9506.32470703125
node_116: feature_name=Flu.H1N1.HA1+HA2	feature_id[10].value <= threshold=54065.41796875
node_117: feature_name=SARS.CoV.S1.RBD.rFcTag	feature_id[16].value <= threshold=24639.0
node_118: feature_name=SARS.CoV.2.Spike.RBD.His.Bac	feature_id[1].value <= threshold=23841.4169921875
node_119: feature_name=SARS.CoV.2.S2	feature_id[29].value <= threshold=49775.806640625
node_120: feature_name=MERS.CoV.NP	feature_id[15].value <= threshold=8014.466796875
node_121: feature_name=Flu.H1N1.HA1+HA2	feature_id[10].value > threshold=53033.25

node_123: feature_name=hCoV.229E.S1_S2	feature_id[35].value > threshold=33883.634765625
Class: healthcare workers between 60 and 180 days after vaccination	
Rules_156	passed counts:1
node_0: feature_name=HuIgG_0.10	feature_id[2].value > threshold=19120.166015625
node_96: feature_name=a-HuIgG_0.03	feature_id[7].value <= threshold=19665.666015625
node_97: feature_name=MERS.CoV.S1.RBD.367.606.rFcTag	feature_id[5].value <= threshold=3252.625
node_98: feature_name=SARS.CoV.2.S2	feature_id[29].value > threshold=9506.32470703125
node_116: feature_name=Flu.H1N1.HA1+HA2	feature_id[10].value <= threshold=54065.41796875
node_117: feature_name=SARS.CoV.S1.RBD.rFcTag	feature_id[16].value <= threshold=24639.0
node_118: feature_name=SARS.CoV.2.Spike.RBD.His.Bac	feature_id[1].value <= threshold=23841.4169921875
node_119: feature_name=SARS.CoV.2.S2	feature_id[29].value <= threshold=49775.806640625
node_120: feature_name=MERS.CoV.NP	feature_id[15].value <= threshold=8014.466796875
node_121: feature_name=Flu.H1N1.HA1+HA2	feature_id[10].value > threshold=53033.25
node_123: feature_name=hCoV.229E.S1_S2	feature_id[35].value <= threshold=33883.634765625
Class: healthcare workers within 60 days after vaccination	
Rules_157	passed counts:1
node_0: feature_name=HuIgG_0.10	feature_id[2].value > threshold=19120.166015625
node_96: feature_name=a-HuIgG_0.03	feature_id[7].value <= threshold=19665.666015625
node_97: feature_name=MERS.CoV.S1.RBD.367.606.rFcTag	feature_id[5].value <= threshold=3252.625
node_98: feature_name=SARS.CoV.2.S2	feature_id[29].value <= threshold=9506.32470703125
node_99: feature_name=SARS.CoV.2.S1.RBD.mFc	feature_id[28].value > threshold=4672.199844360352
node_111: feature_name=HuIgM_0.10	feature_id[6].value <= threshold=-30.333333015441895
node_112: feature_name=a-MoIgG_0.10	feature_id[27].value > threshold=2261.1666259765625

Class: healthcare workers within 60 days after vaccination	
Rules_158	passed counts:1
node_0: feature_name=HuIgG_0.10	feature_id[2].value > threshold=19120.166015625
node_96: feature_name=a-HuIgG_0.03	feature_id[7].value <= threshold=19665.666015625
node_97: feature_name=MERS.CoV.S1.RBD.367.606.rFcTag	feature_id[5].value <= threshold=3252.625
node_98: feature_name=SARS.CoV.2.S2	feature_id[29].value <= threshold=9506.32470703125
node_99: feature_name=SARS.CoV.2.S1.RBD.mFc	feature_id[28].value <= threshold=4672.199844360352
node_100: feature_name=a-MoIgA_0.03	feature_id[24].value > threshold=14.999999523162842
node_106: feature_name=HuIgM_0.10	feature_id[6].value > threshold=65.66666793823242
node_108: feature_name=SARS.CoV.2.S2	feature_id[29].value <= threshold=2100.388916015625
Class: unvaccinated healthcare workers	
Rules_159	passed counts:1
node_0: feature_name=HuIgG_0.10	feature_id[2].value > threshold=19120.166015625
node_96: feature_name=a-HuIgG_0.03	feature_id[7].value <= threshold=19665.666015625
node_97: feature_name=MERS.CoV.S1.RBD.367.606.rFcTag	feature_id[5].value <= threshold=3252.625
node_98: feature_name=SARS.CoV.2.S2	feature_id[29].value <= threshold=9506.32470703125
node_99: feature_name=SARS.CoV.2.S1.RBD.mFc	feature_id[28].value <= threshold=4672.199844360352
node_100: feature_name=a-MoIgA_0.03	feature_id[24].value <= threshold=14.999999523162842
node_101: feature_name=a-HuIgG_0.03	feature_id[7].value <= threshold=313.1666564941406
node_102: feature_name=MoIgG_0.30	feature_id[34].value > threshold=26.999999284744263
Class: healthcare workers over 180 days after vaccination	
Rules_160	passed counts:1

node_0: feature_name=HuIgG_0.10	feature_id[2].value > threshold=19120.166015625
node_96: feature_name=a-HuIgG_0.03	feature_id[7].value <= threshold=19665.666015625
node_97: feature_name=MERS.CoV.S1.RBD.367.606.rFcTag	feature_id[5].value <= threshold=3252.625
node_98: feature_name=SARS.CoV.2.S2	feature_id[29].value <= threshold=9506.32470703125
node_99: feature_name=SARS.CoV.2.S1.RBD.mFc	feature_id[28].value <= threshold=4672.199844360352
node_100: feature_name=a-MoIgA_0.03	feature_id[24].value <= threshold=14.999999523162842
node_101: feature_name=a-HuIgG_0.03	feature_id[7].value <= threshold=313.1666564941406
node_102: feature_name=MoIgG_0.30	feature_id[34].value <= threshold=26.999999284744263
Class: healthcare workers between 60 and 180 days after vaccination	
Rules_161	passed counts:1
node_0: feature_name=HuIgG_0.10	feature_id[2].value <= threshold=19120.166015625
node_1: feature_name=SARS.CoV.2.S1.RBD.mFc	feature_id[28].value > threshold=3773.8334350585938
node_9: feature_name=HuIgM_0.03	feature_id[14].value > threshold=-88.33333206176758
node_27: feature_name=hCoV.HKU1.S1_S2	feature_id[32].value > threshold=42767.0
node_77: feature_name=Flu.B_Mal/.HA1	feature_id[9].value > threshold=41191.69921875
node_91: feature_name=hCoV.NL63.S1_S2	feature_id[13].value > threshold=33974.650390625
node_93: feature_name=Flu.H1N1.HA1+HA2	feature_id[10].value > threshold=54167.6015625
Class: healthcare workers over 180 days after vaccination	
Rules_162	passed counts:1
node_0: feature_name=HuIgG_0.10	feature_id[2].value <= threshold=19120.166015625
node_1: feature_name=SARS.CoV.2.S1.RBD.mFc	feature_id[28].value > threshold=3773.8334350585938
node_9: feature_name=HuIgM_0.03	feature_id[14].value > threshold=-88.33333206176758
node_27: feature_name=hCoV.HKU1.S1_S2	feature_id[32].value > threshold=42767.0

node_77: feature_name=Flu.B_Mal/.HA1	feature_id[9].value <= threshold=41191.69921875
node_78: feature_name=SARS.CoV.2.S1.mFcTag	feature_id[21].value > threshold=41709.720703125
node_82: feature_name=HuIgM_0.10	feature_id[6].value > threshold=-43.83333396911621
node_86: feature_name=HuIgG_0.10	feature_id[2].value <= threshold=18966.0
node_87: feature_name=SARS.CoV.2.S2	feature_id[29].value <= threshold=22915.0
Class: healthcare workers between 60 and 180 days after vaccination	
Rules_163	passed counts:1
node_0: feature_name=HuIgG_0.10	feature_id[2].value <= threshold=19120.166015625
node_1: feature_name=SARS.CoV.2.S1.RBD.mFc	feature_id[28].value > threshold=3773.8334350585938
node_9: feature_name=HuIgM_0.03	feature_id[14].value > threshold=-88.33333206176758
node_27: feature_name=hCoV.HKU1.S1_S2	feature_id[32].value > threshold=42767.0
node_77: feature_name=Flu.B_Mal/.HA1	feature_id[9].value <= threshold=41191.69921875
node_78: feature_name=SARS.CoV.2.S1.mFcTag	feature_id[21].value > threshold=41709.720703125
node_82: feature_name=HuIgM_0.10	feature_id[6].value <= threshold=-43.83333396911621
node_83: feature_name=SARS.CoV.S1.HisTag	feature_id[18].value <= threshold=8532.783203125
Class: healthcare workers over 180 days after vaccination	
Rules_164	passed counts:1
node_0: feature_name=HuIgG_0.10	feature_id[2].value <= threshold=19120.166015625
node_1: feature_name=SARS.CoV.2.S1.RBD.mFc	feature_id[28].value > threshold=3773.8334350585938
node_9: feature_name=HuIgM_0.03	feature_id[14].value > threshold=-88.33333206176758
node_27: feature_name=hCoV.HKU1.S1_S2	feature_id[32].value <= threshold=42767.0
node_28: feature_name=HuIgG_0.03	feature_id[0].value > threshold=1005.0
node_38: feature_name=a-HuIgG_0.30	feature_id[4].value > threshold=33596.333984375

node_60: feature_name=a-HuIgG_0.03	feature_id[7].value > threshold=10366.5
node_70: feature_name=hCoV.OC43.HE	feature_id[33].value <= threshold=23182.7001953125
node_71: feature_name=SARS.CoV.2.Spike.RBD.His.Bac	feature_id[1].value > threshold=48979.25
Class: healthcare workers over 180 days after vaccination	
Rules_165	passed counts:1
node_0: feature_name=HuIgG_0.10	feature_id[2].value <= threshold=19120.166015625
node_1: feature_name=SARS.CoV.2.S1.RBD.mFc	feature_id[28].value > threshold=3773.8334350585938
node_9: feature_name=HuIgM_0.03	feature_id[14].value > threshold=-88.33333206176758
node_27: feature_name=hCoV.HKU1.S1_S2	feature_id[32].value <= threshold=42767.0
node_28: feature_name=HuIgG_0.03	feature_id[0].value > threshold=1005.0
node_38: feature_name=a-HuIgG_0.30	feature_id[4].value > threshold=33596.333984375
node_60: feature_name=a-HuIgG_0.03	feature_id[7].value > threshold=10366.5
node_70: feature_name=hCoV.OC43.HE	feature_id[33].value <= threshold=23182.7001953125
node_71: feature_name=SARS.CoV.2.Spike.RBD.His.Bac	feature_id[1].value <= threshold=48979.25
node_72: feature_name=HuIgM_0.10	feature_id[6].value > threshold=40.166666984558105
Class: healthcare workers within 60 days after vaccination	
Rules_166	passed counts:1
node_0: feature_name=HuIgG_0.10	feature_id[2].value <= threshold=19120.166015625
node_1: feature_name=SARS.CoV.2.S1.RBD.mFc	feature_id[28].value > threshold=3773.8334350585938
node_9: feature_name=HuIgM_0.03	feature_id[14].value > threshold=-88.33333206176758
node_27: feature_name=hCoV.HKU1.S1_S2	feature_id[32].value <= threshold=42767.0
node_28: feature_name=HuIgG_0.03	feature_id[0].value > threshold=1005.0
node_38: feature_name=a-HuIgG_0.30	feature_id[4].value > threshold=33596.333984375

node_60: feature_name=a-HuIgG_0.03	feature_id[7].value <= threshold=10366.5
node_61: feature_name=Flu.H3N2.HA1	feature_id[25].value > threshold=29968.0830078125
node_65: feature_name=HuIgG_0.30	feature_id[36].value > threshold=36026.0
node_67: feature_name=HuIgM_0.10	feature_id[6].value > threshold=47.500000953674316
Class: healthcare workers over 180 days after vaccination	
Rules_167	passed counts:1
node_0: feature_name=HuIgG_0.10	feature_id[2].value <= threshold=19120.166015625
node_1: feature_name=SARS.CoV.2.S1.RBD.mFc	feature_id[28].value > threshold=3773.8334350585938
node_9: feature_name=HuIgM_0.03	feature_id[14].value > threshold=-88.33333206176758
node_27: feature_name=hCoV.HKU1.S1_S2	feature_id[32].value <= threshold=42767.0
node_28: feature_name=HuIgG_0.03	feature_id[0].value > threshold=1005.0
node_38: feature_name=a-HuIgG_0.30	feature_id[4].value > threshold=33596.333984375
node_60: feature_name=a-HuIgG_0.03	feature_id[7].value <= threshold=10366.5
node_61: feature_name=Flu.H3N2.HA1	feature_id[25].value > threshold=29968.0830078125
node_65: feature_name=HuIgG_0.30	feature_id[36].value > threshold=36026.0
node_67: feature_name=HuIgM_0.10	feature_id[6].value <= threshold=47.500000953674316
Class: healthcare workers between 60 and 180 days after vaccination	
Rules_168	passed counts:1
node_0: feature_name=HuIgG_0.10	feature_id[2].value <= threshold=19120.166015625
node_1: feature_name=SARS.CoV.2.S1.RBD.mFc	feature_id[28].value > threshold=3773.8334350585938
node_9: feature_name=HuIgM_0.03	feature_id[14].value > threshold=-88.33333206176758
node_27: feature_name=hCoV.HKU1.S1_S2	feature_id[32].value <= threshold=42767.0
node_28: feature_name=HuIgG_0.03	feature_id[0].value > threshold=1005.0

node_38: feature_name=a-HuIgG_0.30	feature_id[4].value <= threshold=33596.333984375
node_39: feature_name=SARS.CoV.S1.HisTag	feature_id[18].value > threshold=400.3000030517578
node_43: feature_name=HuIgM_0.10	feature_id[6].value > threshold=109.66666793823242
node_57: feature_name=HuIgG_0.30	feature_id[36].value <= threshold=23562.0
Class: healthcare workers between 60 and 180 days after vaccination	
Rules_169	passed counts:1
node_0: feature_name=HuIgG_0.10	feature_id[2].value <= threshold=19120.166015625
node_1: feature_name=SARS.CoV.2.S1.RBD.mFc	feature_id[28].value > threshold=3773.8334350585938
node_9: feature_name=HuIgM_0.03	feature_id[14].value > threshold=-88.33333206176758
node_27: feature_name=hCoV.HKU1.S1_S2	feature_id[32].value <= threshold=42767.0
node_28: feature_name=HuIgG_0.03	feature_id[0].value > threshold=1005.0
node_38: feature_name=a-HuIgG_0.30	feature_id[4].value <= threshold=33596.333984375
node_39: feature_name=SARS.CoV.S1.HisTag	feature_id[18].value > threshold=400.3000030517578
node_43: feature_name=HuIgM_0.10	feature_id[6].value <= threshold=109.66666793823242
node_44: feature_name=MERS.CoV.NP	feature_id[15].value > threshold=-50.125
node_46: feature_name=hCoV.229E.S1_S2	feature_id[35].value > threshold=42044.208984375
Class: healthcare workers over 180 days after vaccination	
Rules_170	passed counts:1
node_0: feature_name=HuIgG_0.10	feature_id[2].value <= threshold=19120.166015625
node_1: feature_name=SARS.CoV.2.S1.RBD.mFc	feature_id[28].value > threshold=3773.8334350585938
node_9: feature_name=HuIgM_0.03	feature_id[14].value > threshold=-88.33333206176758
node_27: feature_name=hCoV.HKU1.S1_S2	feature_id[32].value <= threshold=42767.0
node_28: feature_name=HuIgG_0.03	feature_id[0].value > threshold=1005.0

node_38: feature_name=a-HuIgG_0.30	feature_id[4].value <= threshold=33596.333984375
node_39: feature_name=SARS.CoV.S1.HisTag	feature_id[18].value > threshold=400.3000030517578
node_43: feature_name=HuIgM_0.10	feature_id[6].value <= threshold=109.66666793823242
node_44: feature_name=MERS.CoV.NP	feature_id[15].value > threshold=-50.125
node_46: feature_name=hCoV.229E.S1_S2	feature_id[35].value <= threshold=42044.208984375
node_47: feature_name=HuIgM_0.30	feature_id[8].value > threshold=1223.5
node_53: feature_name=hCoV.HKU1.S1_AA1.760	feature_id[39].value > threshold=11187.500366210938
Class: healthcare workers between 60 and 180 days after vaccination	
Rules_171	passed counts:1
node_0: feature_name=HuIgG_0.10	feature_id[2].value <= threshold=19120.166015625
node_1: feature_name=SARS.CoV.2.S1.RBD.mFc	feature_id[28].value > threshold=3773.8334350585938
node_9: feature_name=HuIgM_0.03	feature_id[14].value > threshold=-88.33333206176758
node_27: feature_name=hCoV.HKU1.S1_S2	feature_id[32].value <= threshold=42767.0
node_28: feature_name=HuIgG_0.03	feature_id[0].value > threshold=1005.0
node_38: feature_name=a-HuIgG_0.30	feature_id[4].value <= threshold=33596.333984375
node_39: feature_name=SARS.CoV.S1.HisTag	feature_id[18].value > threshold=400.3000030517578
node_43: feature_name=HuIgM_0.10	feature_id[6].value <= threshold=109.66666793823242
node_44: feature_name=MERS.CoV.NP	feature_id[15].value > threshold=-50.125
node_46: feature_name=hCoV.229E.S1_S2	feature_id[35].value <= threshold=42044.208984375
node_47: feature_name=HuIgM_0.30	feature_id[8].value > threshold=1223.5
node_53: feature_name=hCoV.HKU1.S1_AA1.760	feature_id[39].value <= threshold=11187.500366210938
Class: healthcare workers within 60 days after vaccination	
Rules_172	passed counts:1

node_0: feature_name=HuIgG_0.10	feature_id[2].value <= threshold=19120.166015625
node_1: feature_name=SARS.CoV.2.S1.RBD.mFc	feature_id[28].value > threshold=3773.8334350585938
node_9: feature_name=HuIgM_0.03	feature_id[14].value > threshold=-88.33333206176758
node_27: feature_name=hCoV.HKU1.S1_S2	feature_id[32].value <= threshold=42767.0
node_28: feature_name=HuIgG_0.03	feature_id[0].value > threshold=1005.0
node_38: feature_name=a-HuIgG_0.30	feature_id[4].value <= threshold=33596.333984375
node_39: feature_name=SARS.CoV.S1.HisTag	feature_id[18].value > threshold=400.3000030517578
node_43: feature_name=HuIgM_0.10	feature_id[6].value <= threshold=109.66666793823242
node_44: feature_name=MERS.CoV.NP	feature_id[15].value <= threshold=-50.125
Class: healthcare workers over 180 days after vaccination	
Rules_173	passed counts:1
node_0: feature_name=HuIgG_0.10	feature_id[2].value <= threshold=19120.166015625
node_1: feature_name=SARS.CoV.2.S1.RBD.mFc	feature_id[28].value > threshold=3773.8334350585938
node_9: feature_name=HuIgM_0.03	feature_id[14].value > threshold=-88.33333206176758
node_27: feature_name=hCoV.HKU1.S1_S2	feature_id[32].value <= threshold=42767.0
node_28: feature_name=HuIgG_0.03	feature_id[0].value > threshold=1005.0
node_38: feature_name=a-HuIgG_0.30	feature_id[4].value <= threshold=33596.333984375
node_39: feature_name=SARS.CoV.S1.HisTag	feature_id[18].value <= threshold=400.3000030517578
node_40: feature_name=HuIgA_0.03	feature_id[26].value > threshold=448.0
Class: healthcare workers between 60 and 180 days after vaccination	
Rules_174	passed counts:1
node_0: feature_name=HuIgG_0.10	feature_id[2].value <= threshold=19120.166015625
node_1: feature_name=SARS.CoV.2.S1.RBD.mFc	feature_id[28].value > threshold=3773.8334350585938

node_9: feature_name=HuIgM_0.03	feature_id[14].value > threshold=-88.33333206176758
node_27: feature_name=hCoV.HKU1.S1_S2	feature_id[32].value <= threshold=42767.0
node_28: feature_name=HuIgG_0.03	feature_id[0].value <= threshold=1005.0
node_29: feature_name=SARS.CoV.2.S1.mFcTag	feature_id[21].value <= threshold=34194.916015625
node_30: feature_name=SARS.CoV.2.S2	feature_id[29].value > threshold=7509.916748046875
node_32: feature_name=MoIgG_0.03	feature_id[20].value > threshold=-249.83333349227905
Class: healthcare workers between 60 and 180 days after vaccination	
Rules_175	passed counts:1
node_0: feature_name=HuIgG_0.10	feature_id[2].value <= threshold=19120.166015625
node_1: feature_name=SARS.CoV.2.S1.RBD.mFc	feature_id[28].value > threshold=3773.8334350585938
node_9: feature_name=HuIgM_0.03	feature_id[14].value > threshold=-88.33333206176758
node_27: feature_name=hCoV.HKU1.S1_S2	feature_id[32].value <= threshold=42767.0
node_28: feature_name=HuIgG_0.03	feature_id[0].value <= threshold=1005.0
node_29: feature_name=SARS.CoV.2.S1.mFcTag	feature_id[21].value <= threshold=34194.916015625
node_30: feature_name=SARS.CoV.2.S2	feature_id[29].value > threshold=7509.916748046875
node_32: feature_name=MoIgG_0.03	feature_id[20].value <= threshold=-249.83333349227905
Class: healthcare workers over 180 days after vaccination	
Rules_176	passed counts:1
node_0: feature_name=HuIgG_0.10	feature_id[2].value <= threshold=19120.166015625
node_1: feature_name=SARS.CoV.2.S1.RBD.mFc	feature_id[28].value > threshold=3773.8334350585938
node_9: feature_name=HuIgM_0.03	feature_id[14].value <= threshold=-88.33333206176758
node_10: feature_name=HuIgA_0.03	feature_id[26].value <= threshold=304.5
node_11: feature_name=Flu.H1N1.HA1+HA2	feature_id[10].value > threshold=53444.666015625

node_19: feature_name=a-MoIgA_0.03	feature_id[24].value > threshold=-97.16666793823242
node_21: feature_name=a-MoIgA_0.03	feature_id[24].value > threshold=0.6666666269302368
node_23: feature_name=Flu.H3N2.HA1	feature_id[25].value > threshold=26045.533203125
Class: healthcare workers within 60 days after vaccination	
Rules_177	passed counts:1
node_0: feature_name=HuIgG_0.10	feature_id[2].value <= threshold=19120.166015625
node_1: feature_name=SARS.CoV.2.S1.RBD.mFc	feature_id[28].value > threshold=3773.8334350585938
node_9: feature_name=HuIgM_0.03	feature_id[14].value <= threshold=-88.33333206176758
node_10: feature_name=HuIgA_0.03	feature_id[26].value <= threshold=304.5
node_11: feature_name=Flu.H1N1.HA1+HA2	feature_id[10].value > threshold=53444.666015625
node_19: feature_name=a-MoIgA_0.03	feature_id[24].value > threshold=-97.16666793823242
node_21: feature_name=a-MoIgA_0.03	feature_id[24].value > threshold=0.6666666269302368
node_23: feature_name=Flu.H3N2.HA1	feature_id[25].value <= threshold=26045.533203125
Class: healthcare workers between 60 and 180 days after vaccination	
Rules_178	passed counts:1
node_0: feature_name=HuIgG_0.10	feature_id[2].value <= threshold=19120.166015625
node_1: feature_name=SARS.CoV.2.S1.RBD.mFc	feature_id[28].value > threshold=3773.8334350585938
node_9: feature_name=HuIgM_0.03	feature_id[14].value <= threshold=-88.33333206176758
node_10: feature_name=HuIgA_0.03	feature_id[26].value <= threshold=304.5
node_11: feature_name=Flu.H1N1.HA1+HA2	feature_id[10].value <= threshold=53444.666015625
node_12: feature_name=MERS.CoV.S1.RBD.367.606.rFcTag	feature_id[5].value > threshold=-2425.0
node_14: feature_name=Flu.B_Mal/.HA1+HA2	feature_id[22].value > threshold=53898.0
node_16: feature_name=hCoV.OC43.HE	feature_id[33].value > threshold=18505.49951171875

Class: healthcare workers between 60 and 180 days after vaccination	
Rules_179	passed counts:1
node_0: feature_name=HuIgG_0.10	feature_id[2].value <= threshold=19120.166015625
node_1: feature_name=SARS.CoV.2.S1.RBD.mFc	feature_id[28].value > threshold=3773.8334350585938
node_9: feature_name=HuIgM_0.03	feature_id[14].value <= threshold=-88.33333206176758
node_10: feature_name=HuIgA_0.03	feature_id[26].value <= threshold=304.5
node_11: feature_name=Flu.H1N1.HA1+HA2	feature_id[10].value <= threshold=53444.666015625
node_12: feature_name=MERS.CoV.S1.RBD.367.606.rFcTag	feature_id[5].value > threshold=-2425.0
node_14: feature_name=Flu.B_Mal/.HA1+HA2	feature_id[22].value > threshold=53898.0
node_16: feature_name=hCoV.OC43.HE	feature_id[33].value <= threshold=18505.49951171875
Class: healthcare workers over 180 days after vaccination	
Rules_180	passed counts:1
node_0: feature_name=HuIgG_0.10	feature_id[2].value <= threshold=19120.166015625
node_1: feature_name=SARS.CoV.2.S1.RBD.mFc	feature_id[28].value > threshold=3773.8334350585938
node_9: feature_name=HuIgM_0.03	feature_id[14].value <= threshold=-88.33333206176758
node_10: feature_name=HuIgA_0.03	feature_id[26].value <= threshold=304.5
node_11: feature_name=Flu.H1N1.HA1+HA2	feature_id[10].value <= threshold=53444.666015625
node_12: feature_name=MERS.CoV.S1.RBD.367.606.rFcTag	feature_id[5].value <= threshold=-2425.0
Class: healthcare workers between 60 and 180 days after vaccination	
Rules_181	passed counts:1
node_0: feature_name=HuIgG_0.10	feature_id[2].value <= threshold=19120.166015625
node_1: feature_name=SARS.CoV.2.S1.RBD.mFc	feature_id[28].value <= threshold=3773.8334350585938

node_2: feature_name=SARS.CoV.2.S1.RBD.mFc	feature_id[28].value > threshold=-414.2999954223633
node_4: feature_name=hCoV.OC43.HE	feature_id[33].value <= threshold=414.5370330810547
node_5: feature_name=Flu.H1N1.HA1+HA2	feature_id[10].value > threshold=29887.27734375
Class: healthcare workers within 60 days after vaccination	
Rules_182	passed counts:1
node_0: feature_name=HuIgG_0.10	feature_id[2].value <= threshold=19120.166015625
node_1: feature_name=SARS.CoV.2.S1.RBD.mFc	feature_id[28].value <= threshold=3773.8334350585938
node_2: feature_name=SARS.CoV.2.S1.RBD.mFc	feature_id[28].value <= threshold=-414.2999954223633
Class: healthcare workers between 60 and 180 days after vaccination	

(3) Rules on the MCFS feature list

Rules_0	passed counts:169
node_0: feature_name=HuIgG_0.10	feature_id[9].value <= threshold=19120.166015625
node_1: feature_name=SARS.CoV.2.S1.RBD.mFc	feature_id[1].value > threshold=3773.8334350585938
node_9: feature_name=a-HuIgG_0.30	feature_id[11].value <= threshold=33726.833984375
node_10: feature_name=HuIgG_0.03	feature_id[8].value > threshold=1023.1666564941406
node_20: feature_name=SARS.CoV.S1.HisTag	feature_id[15].value > threshold=400.3000030517578
node_24: feature_name=SARS.CoV.2.Spike.RBD.His.Bac	feature_id[3].value <= threshold=36933.69921875
node_25: feature_name=SARS.CoV.2.S1.RBD.mFc	feature_id[1].value <= threshold=40856.734375
node_26: feature_name=SARS.CoV.S1.HisTag	feature_id[15].value <= threshold=15087.41650390625
node_27: feature_name=SARS.CoV.2.Spike.RBD.His.HEK	feature_id[4].value > threshold=10178.375
Class: healthcare workers between 60 and 180 days after vaccination	
Rules_1	passed counts:73

node_0: feature_name=HuIgG_0.10	feature_id[9].value <= threshold=19120.166015625
node_1: feature_name=SARS.CoV.2.S1.RBD.mFc	feature_id[1].value <= threshold=3773.8334350585938
node_2: feature_name=SARS.CoV.2.S1.HisTag	feature_id[2].value > threshold=-383.86944580078125
node_4: feature_name=SARS.CoV.2.S1	feature_id[6].value > threshold=435.629638671875
Class: unvaccinated healthcare workers	
Rules_2	passed counts:68
node_0: feature_name=HuIgG_0.10	feature_id[9].value <= threshold=19120.166015625
node_1: feature_name=SARS.CoV.2.S1.RBD.mFc	feature_id[1].value > threshold=3773.8334350585938
node_9: feature_name=a-HuIgG_0.30	feature_id[11].value <= threshold=33726.833984375
node_10: feature_name=HuIgG_0.03	feature_id[8].value > threshold=1023.1666564941406
node_20: feature_name=SARS.CoV.S1.HisTag	feature_id[15].value > threshold=400.3000030517578
node_24: feature_name=SARS.CoV.2.Spike.RBD.His.Bac	feature_id[3].value <= threshold=36933.69921875
node_25: feature_name=SARS.CoV.2.S1.RBD.mFc	feature_id[1].value <= threshold=40856.734375
node_26: feature_name=SARS.CoV.S1.HisTag	feature_id[15].value <= threshold=15087.41650390625
node_27: feature_name=SARS.CoV.2.Spike.RBD.His.HEK	feature_id[4].value <= threshold=10178.375
node_28: feature_name=SARS.CoV.2.S1.RBD.mFc	feature_id[1].value <= threshold=33458.650390625
node_29: feature_name=SARS.CoV.2.Spike.RBD.His.HEK	feature_id[4].value <= threshold=10129.44140625
Class: healthcare workers between 60 and 180 days after vaccination	
Rules_3	passed counts:54
node_0: feature_name=HuIgG_0.10	feature_id[9].value > threshold=19120.166015625
node_92: feature_name=a-HuIgG_0.03	feature_id[14].value <= threshold=19665.666015625
node_93: feature_name=SARS.CoV.2.Spike.RBD.His.HEK	feature_id[4].value > threshold=3.0166666507720947
node_105: feature_name=SARS.CoV.2.S1.mFcTag	feature_id[0].value <= threshold=54010.166015625

node_106: feature_name=SARS.CoV.2.S1+S2	feature_id[7].value > threshold=48317.708984375
node_228: feature_name=a-MoIgG_0.10	feature_id[16].value > threshold=1652.5
node_230: feature_name=HuIgG_0.10	feature_id[9].value <= threshold=30107.666015625
node_231: feature_name=HuIgG_0.03	feature_id[8].value <= threshold=5533.83349609375
node_232: feature_name=SARS.CoV.2.Spike.RBD.His.HEK	feature_id[4].value > threshold=11116.333374023438
node_234: feature_name=SARS.CoV.2.S1+S2	feature_id[7].value > threshold=48882.58203125
node_240: feature_name=a-HuIgG_0.03	feature_id[14].value > threshold=10994.83349609375
Class: healthcare workers within 60 days after vaccination	
Rules_4	passed counts:42
node_0: feature_name=HuIgG_0.10	feature_id[9].value > threshold=19120.166015625
node_92: feature_name=a-HuIgG_0.03	feature_id[14].value <= threshold=19665.666015625
node_93: feature_name=SARS.CoV.2.Spike.RBD.His.HEK	feature_id[4].value > threshold=3.0166666507720947
node_105: feature_name=SARS.CoV.2.S1.mFcTag	feature_id[0].value <= threshold=54010.166015625
node_106: feature_name=SARS.CoV.2.S1+S2	feature_id[7].value > threshold=48317.708984375
node_228: feature_name=a-MoIgG_0.10	feature_id[16].value > threshold=1652.5
node_230: feature_name=HuIgG_0.10	feature_id[9].value <= threshold=30107.666015625
node_231: feature_name=HuIgG_0.03	feature_id[8].value > threshold=5533.83349609375
node_247: feature_name=HuIgG_0.10	feature_id[9].value > threshold=24231.0
node_261: feature_name=SARS.CoV.S1.RBD.HisTag	feature_id[10].value > threshold=24179.0419921875
node_293: feature_name=SARS.CoV.2.S1.HisTag	feature_id[2].value <= threshold=54233.25
node_294: feature_name=SARS.CoV.2.S1.mFcTag	feature_id[0].value > threshold=50885.83203125
node_298: feature_name=a-HuIgG_0.30	feature_id[11].value > threshold=40480.16796875
Class: healthcare workers within 60 days after vaccination	

Rules_5	passed counts:31
node_0: feature_name=HuIgG_0.10	feature_id[9].value > threshold=19120.166015625
node_92: feature_name=a-HuIgG_0.03	feature_id[14].value <= threshold=19665.666015625
node_93: feature_name=SARS.CoV.2.Spike.RBD.His.HEK	feature_id[4].value > threshold=3.0166666507720947
node_105: feature_name=SARS.CoV.2.S1.mFcTag	feature_id[0].value <= threshold=54010.166015625
node_106: feature_name=SARS.CoV.2.S1+S2	feature_id[7].value <= threshold=48317.708984375
node_107: feature_name=a-HuIgG_0.03	feature_id[14].value <= threshold=12099.83349609375
node_108: feature_name=SARS.CoV.2.Spike.RBD.His.Bac	feature_id[3].value <= threshold=23841.4169921875
node_109: feature_name=SARS.CoV.2.S1+S2	feature_id[7].value > threshold=36783.25
Class: healthcare workers within 60 days after vaccination	
Rules_6	passed counts:30
node_0: feature_name=HuIgG_0.10	feature_id[9].value > threshold=19120.166015625
node_92: feature_name=a-HuIgG_0.03	feature_id[14].value <= threshold=19665.666015625
node_93: feature_name=SARS.CoV.2.Spike.RBD.His.HEK	feature_id[4].value > threshold=3.0166666507720947
node_105: feature_name=SARS.CoV.2.S1.mFcTag	feature_id[0].value <= threshold=54010.166015625
node_106: feature_name=SARS.CoV.2.S1+S2	feature_id[7].value > threshold=48317.708984375
node_228: feature_name=a-MoIgG_0.10	feature_id[16].value > threshold=1652.5
node_230: feature_name=HuIgG_0.10	feature_id[9].value <= threshold=30107.666015625
node_231: feature_name=HuIgG_0.03	feature_id[8].value > threshold=5533.83349609375
node_247: feature_name=HuIgG_0.10	feature_id[9].value > threshold=24231.0
node_261: feature_name=SARS.CoV.S1.RBD.HisTag	feature_id[10].value <= threshold=24179.0419921875
node_262: feature_name=HuIgG_0.10	feature_id[9].value > threshold=25555.333984375
node_266: feature_name=HuIgG_0.10	feature_id[9].value > threshold=25659.6669921875
node_270: feature_name=SARS.CoV.S1.HisTag	feature_id[15].value <= threshold=10000.5830078125

node_271: feature_name=SARS.CoV.2.Spike.RBD.His.Bac	feature_id[3].value > threshold=29395.400390625
Class: healthcare workers within 60 days after vaccination	
Rules_7	passed counts:26
node_0: feature_name=HuIgG_0.10	feature_id[9].value > threshold=19120.166015625
node_92: feature_name=a-HuIgG_0.03	feature_id[14].value <= threshold=19665.666015625
node_93: feature_name=SARS.CoV.2.Spike.RBD.His.HEK	feature_id[4].value > threshold=3.0166666507720947
node_105: feature_name=SARS.CoV.2.S1.mFcTag	feature_id[0].value <= threshold=54010.166015625
node_106: feature_name=SARS.CoV.2.S1+S2	feature_id[7].value <= threshold=48317.708984375
node_107: feature_name=a-HuIgG_0.03	feature_id[14].value > threshold=12099.83349609375
node_155: feature_name=SARS.CoV.2.Spike.RBD.His.Bac	feature_id[3].value > threshold=18979.9912109375
node_167: feature_name=a-MoIgG_0.10	feature_id[16].value <= threshold=4979.5
node_168: feature_name=SARS.CoV.2.S1+S2	feature_id[7].value > threshold=36298.75
node_172: feature_name=SARS.CoV.2.S1.RBD.mFc	feature_id[1].value <= threshold=40153.283203125
node_173: feature_name=SARS.CoV.2.S1.HisTag	feature_id[2].value > threshold=25186.375
node_175: feature_name=a-HuIgG_0.10	feature_id[13].value > threshold=27831.166015625
node_177: feature_name=SARS.CoV.2.S1	feature_id[6].value > threshold=29819.333984375
Class: healthcare workers between 60 and 180 days after vaccination	
Rules_8	passed counts:26
node_0: feature_name=HuIgG_0.10	feature_id[9].value <= threshold=19120.166015625
node_1: feature_name=SARS.CoV.2.S1.RBD.mFc	feature_id[1].value > threshold=3773.8334350585938
node_9: feature_name=a-HuIgG_0.30	feature_id[11].value <= threshold=33726.833984375
node_10: feature_name=HuIgG_0.03	feature_id[8].value <= threshold=1023.1666564941406
node_11: feature_name=SARS.CoV.2.S1.mFcTag	feature_id[0].value <= threshold=34194.916015625

node_12: feature_name=SARS.CoV.2.S1+S2	feature_id[7].value <= threshold=26709.75
Class: healthcare workers over 180 days after vaccination	
Rules_9	passed counts:25
node_0: feature_name=HuIgG_0.10	feature_id[9].value > threshold=19120.166015625
node_92: feature_name=a-HuIgG_0.03	feature_id[14].value <= threshold=19665.666015625
node_93: feature_name=SARS.CoV.2.Spike.RBD.His.HEK	feature_id[4].value > threshold=3.0166666507720947
node_105: feature_name=SARS.CoV.2.S1.mFcTag	feature_id[0].value <= threshold=54010.166015625
node_106: feature_name=SARS.CoV.2.S1+S2	feature_id[7].value <= threshold=48317.708984375
node_107: feature_name=a-HuIgG_0.03	feature_id[14].value <= threshold=12099.83349609375
node_108: feature_name=SARS.CoV.2.Spike.RBD.His.Bac	feature_id[3].value > threshold=23841.4169921875
node_118: feature_name=SARS.CoV.2.Spike.RBD.rFc	feature_id[5].value > threshold=34554.69140625
node_120: feature_name=SARS.CoV.2.S1	feature_id[6].value > threshold=36508.716796875
node_142: feature_name=HuIgG_0.30	feature_id[12].value <= threshold=40622.83203125
node_143: feature_name=HuIgG_0.10	feature_id[9].value > threshold=21439.1669921875
Class: healthcare workers within 60 days after vaccination	
Rules_10	passed counts:23
node_0: feature_name=HuIgG_0.10	feature_id[9].value > threshold=19120.166015625
node_92: feature_name=a-HuIgG_0.03	feature_id[14].value <= threshold=19665.666015625
node_93: feature_name=SARS.CoV.2.Spike.RBD.His.HEK	feature_id[4].value > threshold=3.0166666507720947
node_105: feature_name=SARS.CoV.2.S1.mFcTag	feature_id[0].value <= threshold=54010.166015625
node_106: feature_name=SARS.CoV.2.S1+S2	feature_id[7].value > threshold=48317.708984375
node_228: feature_name=a-MoIgG_0.10	feature_id[16].value > threshold=1652.5
node_230: feature_name=HuIgG_0.10	feature_id[9].value <= threshold=30107.666015625

node_231: feature_name=HuIgG_0.03	feature_id[8].value > threshold=5533.83349609375
node_247: feature_name=HuIgG_0.10	feature_id[9].value > threshold=24231.0
node_261: feature_name=SARS.CoV.S1.RBD.HisTag	feature_id[10].value <= threshold=24179.0419921875
node_262: feature_name=HuIgG_0.10	feature_id[9].value <= threshold=25555.333984375
node_263: feature_name=SARS.CoV.2.S1+S2	feature_id[7].value <= threshold=52323.916015625
Class: healthcare workers within 60 days after vaccination	
Rules_11	passed counts:22
node_0: feature_name=HuIgG_0.10	feature_id[9].value <= threshold=19120.166015625
node_1: feature_name=SARS.CoV.2.S1.RBD.mFc	feature_id[1].value > threshold=3773.8334350585938
node_9: feature_name=a-HuIgG_0.30	feature_id[11].value > threshold=33726.833984375
node_47: feature_name=SARS.CoV.2.S1.mFcTag	feature_id[0].value <= threshold=43185.68359375
node_48: feature_name=SARS.CoV.S1.RBD.HisTag	feature_id[10].value > threshold=3416.129638671875
node_52: feature_name=SARS.CoV.2.S1.HisTag	feature_id[2].value > threshold=7094.0
Class: healthcare workers over 180 days after vaccination	
Rules_12	passed counts:20
node_0: feature_name=HuIgG_0.10	feature_id[9].value > threshold=19120.166015625
node_92: feature_name=a-HuIgG_0.03	feature_id[14].value <= threshold=19665.666015625
node_93: feature_name=SARS.CoV.2.Spike.RBD.His.HEK	feature_id[4].value > threshold=3.0166666507720947
node_105: feature_name=SARS.CoV.2.S1.mFcTag	feature_id[0].value > threshold=54010.166015625
node_305: feature_name=a-MoIgG_0.10	feature_id[16].value > threshold=2382.8333740234375
node_309: feature_name=SARS.CoV.S1.HisTag	feature_id[15].value <= threshold=23460.3330078125
node_310: feature_name=HuIgG_0.03	feature_id[8].value <= threshold=6941.33349609375
node_311: feature_name=a-HuIgG_0.10	feature_id[13].value <= threshold=32620.5

node_312: feature_name=SARS.CoV.2.Spike.RBD.His.Bac	feature_id[3].value > threshold=31266.0
node_320: feature_name=a-HuIgG_0.30	feature_id[11].value > threshold=47574.66796875
node_346: feature_name=a-HuIgG_0.10	feature_id[13].value > threshold=28994.833984375
node_348: feature_name=HuIgG_0.30	feature_id[12].value <= threshold=47369.0
Class: healthcare workers between 60 and 180 days after vaccination	
Rules_13	passed counts:19
node_0: feature_name=HuIgG_0.10	feature_id[9].value > threshold=19120.166015625
node_92: feature_name=a-HuIgG_0.03	feature_id[14].value > threshold=19665.666015625
node_382: feature_name=a-MoIgG_0.10	feature_id[16].value <= threshold=20281.6669921875
node_383: feature_name=a-HuIgG_0.10	feature_id[13].value > threshold=33917.66796875
node_391: feature_name=HuIgG_0.10	feature_id[9].value <= threshold=25959.0
Class: healthcare workers over 180 days after vaccination	
Rules_14	passed counts:18
node_0: feature_name=HuIgG_0.10	feature_id[9].value > threshold=19120.166015625
node_92: feature_name=a-HuIgG_0.03	feature_id[14].value <= threshold=19665.666015625
node_93: feature_name=SARS.CoV.2.Spike.RBD.His.HEK	feature_id[4].value > threshold=3.0166666507720947
node_105: feature_name=SARS.CoV.2.S1.mFcTag	feature_id[0].value <= threshold=54010.166015625
node_106: feature_name=SARS.CoV.2.S1+S2	feature_id[7].value <= threshold=48317.708984375
node_107: feature_name=a-HuIgG_0.03	feature_id[14].value > threshold=12099.83349609375
node_155: feature_name=SARS.CoV.2.Spike.RBD.His.Bac	feature_id[3].value > threshold=18979.9912109375
node_167: feature_name=a-MoIgG_0.10	feature_id[16].value > threshold=4979.5
node_201: feature_name=HuIgG_0.10	feature_id[9].value > threshold=26006.0
node_225: feature_name=SARS.CoV.2.Spike.RBD.His.HEK	feature_id[4].value > threshold=26047.1416015625

Class: healthcare workers within 60 days after vaccination	
Rules_15	passed counts:17
node_0: feature_name=HuIgG_0.10	feature_id[9].value > threshold=19120.166015625
node_92: feature_name=a-HuIgG_0.03	feature_id[14].value <= threshold=19665.666015625
node_93: feature_name=SARS.CoV.2.Spike.RBD.His.HEK	feature_id[4].value > threshold=3.0166666507720947
node_105: feature_name=SARS.CoV.2.S1.mFcTag	feature_id[0].value > threshold=54010.166015625
node_305: feature_name=a-MoIgG_0.10	feature_id[16].value > threshold=2382.8333740234375
node_309: feature_name=SARS.CoV.S1.HisTag	feature_id[15].value > threshold=23460.3330078125
node_365: feature_name=a-HuIgG_0.03	feature_id[14].value > threshold=11128.66650390625
node_367: feature_name=SARS.CoV.2.S1.mFcTag	feature_id[0].value > threshold=54294.0
node_371: feature_name=SARS.CoV.2.S1.mFcTag	feature_id[0].value <= threshold=55620.833984375
node_372: feature_name=a-HuIgG_0.10	feature_id[13].value > threshold=28320.6669921875
node_374: feature_name=SARS.CoV.2.S1	feature_id[6].value > threshold=47335.5
node_376: feature_name=SARS.CoV.2.S1+S2	feature_id[7].value <= threshold=54353.83203125
Class: healthcare workers within 60 days after vaccination	
Rules_16	passed counts:17
node_0: feature_name=HuIgG_0.10	feature_id[9].value > threshold=19120.166015625
node_92: feature_name=a-HuIgG_0.03	feature_id[14].value <= threshold=19665.666015625
node_93: feature_name=SARS.CoV.2.Spike.RBD.His.HEK	feature_id[4].value > threshold=3.0166666507720947
node_105: feature_name=SARS.CoV.2.S1.mFcTag	feature_id[0].value <= threshold=54010.166015625
node_106: feature_name=SARS.CoV.2.S1+S2	feature_id[7].value > threshold=48317.708984375
node_228: feature_name=a-MoIgG_0.10	feature_id[16].value > threshold=1652.5
node_230: feature_name=HuIgG_0.10	feature_id[9].value <= threshold=30107.666015625

node_231: feature_name=HuIgG_0.03	feature_id[8].value > threshold=5533.83349609375
node_247: feature_name=HuIgG_0.10	feature_id[9].value > threshold=24231.0
node_261: feature_name=SARS.CoV.S1.RBD.HisTag	feature_id[10].value <= threshold=24179.0419921875
node_262: feature_name=HuIgG_0.10	feature_id[9].value > threshold=25555.333984375
node_266: feature_name=HuIgG_0.10	feature_id[9].value > threshold=25659.6669921875
node_270: feature_name=SARS.CoV.S1.HisTag	feature_id[15].value > threshold=10000.5830078125
node_276: feature_name=SARS.CoV.S1.HisTag	feature_id[15].value > threshold=13228.125
node_286: feature_name=a-MoIgG_0.10	feature_id[16].value <= threshold=10763.83349609375
node_287: feature_name=SARS.CoV.2.Spike.RBD.His.HEK	feature_id[4].value <= threshold=46379.583984375
Class: healthcare workers within 60 days after vaccination	
Rules_17	passed counts:17
node_0: feature_name=HuIgG_0.10	feature_id[9].value > threshold=19120.166015625
node_92: feature_name=a-HuIgG_0.03	feature_id[14].value <= threshold=19665.666015625
node_93: feature_name=SARS.CoV.2.Spike.RBD.His.HEK	feature_id[4].value > threshold=3.0166666507720947
node_105: feature_name=SARS.CoV.2.S1.mFcTag	feature_id[0].value <= threshold=54010.166015625
node_106: feature_name=SARS.CoV.2.S1+S2	feature_id[7].value <= threshold=48317.708984375
node_107: feature_name=a-HuIgG_0.03	feature_id[14].value > threshold=12099.83349609375
node_155: feature_name=SARS.CoV.2.Spike.RBD.His.Bac	feature_id[3].value > threshold=18979.9912109375
node_167: feature_name=a-MoIgG_0.10	feature_id[16].value > threshold=4979.5
node_201: feature_name=HuIgG_0.10	feature_id[9].value <= threshold=26006.0
node_202: feature_name=HuIgG_0.03	feature_id[8].value <= threshold=6080.0
node_203: feature_name=SARS.CoV.2.S1.HisTag	feature_id[2].value <= threshold=38575.359375
node_204: feature_name=SARS.CoV.2.Spike.RBD.His.HEK	feature_id[4].value > threshold=21567.4501953125
node_208: feature_name=HuIgG_0.03	feature_id[8].value > threshold=4924.333251953125

Class: healthcare workers within 60 days after vaccination	
Rules_18	passed counts:16
node_0: feature_name=HuIgG_0.10	feature_id[9].value > threshold=19120.166015625
node_92: feature_name=a-HuIgG_0.03	feature_id[14].value <= threshold=19665.666015625
node_93: feature_name=SARS.CoV.2.Spike.RBD.His.HEK	feature_id[4].value > threshold=3.0166666507720947
node_105: feature_name=SARS.CoV.2.S1.mFcTag	feature_id[0].value <= threshold=54010.166015625
node_106: feature_name=SARS.CoV.2.S1+S2	feature_id[7].value > threshold=48317.708984375
node_228: feature_name=a-MoIgG_0.10	feature_id[16].value > threshold=1652.5
node_230: feature_name=HuIgG_0.10	feature_id[9].value <= threshold=30107.666015625
node_231: feature_name=HuIgG_0.03	feature_id[8].value <= threshold=5533.83349609375
node_232: feature_name=SARS.CoV.2.Spike.RBD.His.HEK	feature_id[4].value > threshold=11116.333374023438
node_234: feature_name=SARS.CoV.2.S1+S2	feature_id[7].value > threshold=48882.58203125
node_240: feature_name=a-HuIgG_0.03	feature_id[14].value <= threshold=10994.83349609375
node_241: feature_name=a-HuIgG_0.30	feature_id[11].value <= threshold=41039.83203125
Class: healthcare workers within 60 days after vaccination	
Rules_19	passed counts:16
node_0: feature_name=HuIgG_0.10	feature_id[9].value > threshold=19120.166015625
node_92: feature_name=a-HuIgG_0.03	feature_id[14].value <= threshold=19665.666015625
node_93: feature_name=SARS.CoV.2.Spike.RBD.His.HEK	feature_id[4].value > threshold=3.0166666507720947
node_105: feature_name=SARS.CoV.2.S1.mFcTag	feature_id[0].value <= threshold=54010.166015625
node_106: feature_name=SARS.CoV.2.S1+S2	feature_id[7].value <= threshold=48317.708984375
node_107: feature_name=a-HuIgG_0.03	feature_id[14].value > threshold=12099.83349609375
node_155: feature_name=SARS.CoV.2.Spike.RBD.His.Bac	feature_id[3].value <= threshold=18979.9912109375

node_156: feature_name=a-MoIgG_0.10	feature_id[16].value > threshold=1739.1666259765625
node_160: feature_name=HuIgG_0.30	feature_id[12].value > threshold=34829.83203125
node_162: feature_name=HuIgG_0.03	feature_id[8].value <= threshold=6681.166748046875
Class: healthcare workers within 60 days after vaccination	
Rules_20	passed counts:15
node_0: feature_name=HuIgG_0.10	feature_id[9].value > threshold=19120.166015625
node_92: feature_name=a-HuIgG_0.03	feature_id[14].value <= threshold=19665.666015625
node_93: feature_name=SARS.CoV.2.Spike.RBD.His.HEK	feature_id[4].value > threshold=3.0166666507720947
node_105: feature_name=SARS.CoV.2.S1.mFcTag	feature_id[0].value <= threshold=54010.166015625
node_106: feature_name=SARS.CoV.2.S1+S2	feature_id[7].value <= threshold=48317.708984375
node_107: feature_name=a-HuIgG_0.03	feature_id[14].value <= threshold=12099.83349609375
node_108: feature_name=SARS.CoV.2.Spike.RBD.His.Bac	feature_id[3].value <= threshold=23841.4169921875
node_109: feature_name=SARS.CoV.2.S1+S2	feature_id[7].value <= threshold=36783.25
node_110: feature_name=SARS.CoV.2.S1	feature_id[6].value <= threshold=26291.7998046875
node_111: feature_name=HuIgG_0.10	feature_id[9].value > threshold=20106.3330078125
node_113: feature_name=HuIgG_0.03	feature_id[8].value <= threshold=5848.666748046875
Class: healthcare workers within 60 days after vaccination	
Rules_21	passed counts:14
node_0: feature_name=HuIgG_0.10	feature_id[9].value > threshold=19120.166015625
node_92: feature_name=a-HuIgG_0.03	feature_id[14].value <= threshold=19665.666015625
node_93: feature_name=SARS.CoV.2.Spike.RBD.His.HEK	feature_id[4].value > threshold=3.0166666507720947
node_105: feature_name=SARS.CoV.2.S1.mFcTag	feature_id[0].value <= threshold=54010.166015625
node_106: feature_name=SARS.CoV.2.S1+S2	feature_id[7].value > threshold=48317.708984375

node_228: feature_name=a-MoIgG_0.10	feature_id[16].value > threshold=1652.5
node_230: feature_name=HuIgG_0.10	feature_id[9].value <= threshold=30107.666015625
node_231: feature_name=HuIgG_0.03	feature_id[8].value > threshold=5533.83349609375
node_247: feature_name=HuIgG_0.10	feature_id[9].value <= threshold=24231.0
node_248: feature_name=SARS.CoV.2.S1.HisTag	feature_id[2].value > threshold=43585.92578125
node_256: feature_name=SARS.CoV.2.S1+S2	feature_id[7].value <= threshold=52592.591796875
node_257: feature_name=SARS.CoV.2.S1.RBD.mFc	feature_id[1].value > threshold=43424.416015625
Class: healthcare workers within 60 days after vaccination	
Rules_22	passed counts:14
node_0: feature_name=HuIgG_0.10	feature_id[9].value > threshold=19120.166015625
node_92: feature_name=a-HuIgG_0.03	feature_id[14].value <= threshold=19665.666015625
node_93: feature_name=SARS.CoV.2.Spike.RBD.His.HEK	feature_id[4].value > threshold=3.0166666507720947
node_105: feature_name=SARS.CoV.2.S1.mFcTag	feature_id[0].value <= threshold=54010.166015625
node_106: feature_name=SARS.CoV.2.S1+S2	feature_id[7].value <= threshold=48317.708984375
node_107: feature_name=a-HuIgG_0.03	feature_id[14].value > threshold=12099.83349609375
node_155: feature_name=SARS.CoV.2.Spike.RBD.His.Bac	feature_id[3].value > threshold=18979.9912109375
node_167: feature_name=a-MoIgG_0.10	feature_id[16].value > threshold=4979.5
node_201: feature_name=HuIgG_0.10	feature_id[9].value <= threshold=26006.0
node_202: feature_name=HuIgG_0.03	feature_id[8].value > threshold=6080.0
node_216: feature_name=SARS.CoV.2.Spike.RBD.rFc	feature_id[5].value <= threshold=43169.25
node_217: feature_name=SARS.CoV.2.Spike.RBD.His.HEK	feature_id[4].value > threshold=12896.658203125
Class: healthcare workers between 60 and 180 days after vaccination	
Rules_23	passed counts:14

node_0: feature_name=HuIgG_0.10	feature_id[9].value > threshold=19120.166015625
node_92: feature_name=a-HuIgG_0.03	feature_id[14].value <= threshold=19665.666015625
node_93: feature_name=SARS.CoV.2.Spike.RBD.His.HEK	feature_id[4].value > threshold=3.0166666507720947
node_105: feature_name=SARS.CoV.2.S1.mFcTag	feature_id[0].value <= threshold=54010.166015625
node_106: feature_name=SARS.CoV.2.S1+S2	feature_id[7].value <= threshold=48317.708984375
node_107: feature_name=a-HuIgG_0.03	feature_id[14].value > threshold=12099.83349609375
node_155: feature_name=SARS.CoV.2.Spike.RBD.His.Bac	feature_id[3].value > threshold=18979.9912109375
node_167: feature_name=a-MoIgG_0.10	feature_id[16].value <= threshold=4979.5
node_168: feature_name=SARS.CoV.2.S1+S2	feature_id[7].value > threshold=36298.75
node_172: feature_name=SARS.CoV.2.S1.RBD.mFc	feature_id[1].value > threshold=40153.283203125
node_182: feature_name=HuIgG_0.10	feature_id[9].value > threshold=21919.833984375
node_186: feature_name=a-HuIgG_0.10	feature_id[13].value > threshold=28919.0
node_192: feature_name=a-HuIgG_0.10	feature_id[13].value <= threshold=31482.6669921875
node_193: feature_name=a-HuIgG_0.03	feature_id[14].value > threshold=12541.0
node_195: feature_name=a-HuIgG_0.30	feature_id[11].value > threshold=40626.0
Class: healthcare workers between 60 and 180 days after vaccination	
Rules_24	passed counts:14
node_0: feature_name=HuIgG_0.10	feature_id[9].value > threshold=19120.166015625
node_92: feature_name=a-HuIgG_0.03	feature_id[14].value <= threshold=19665.666015625
node_93: feature_name=SARS.CoV.2.Spike.RBD.His.HEK	feature_id[4].value <= threshold=3.0166666507720947
node_94: feature_name=SARS.CoV.2.Spike.RBD.His.Bac	feature_id[3].value > threshold=-51.85277557373047
node_100: feature_name=SARS.CoV.2.S1.mFcTag	feature_id[0].value <= threshold=488.56666564941406
node_101: feature_name=a-HuIgG_0.30	feature_id[11].value > threshold=34723.5
Class: unvaccinated healthcare workers	

Rules_25	passed counts:13
node_0: feature_name=HuIgG_0.10	feature_id[9].value > threshold=19120.166015625
node_92: feature_name=a-HuIgG_0.03	feature_id[14].value <= threshold=19665.666015625
node_93: feature_name=SARS.CoV.2.Spike.RBD.His.HEK	feature_id[4].value > threshold=3.0166666507720947
node_105: feature_name=SARS.CoV.2.S1.mFcTag	feature_id[0].value <= threshold=54010.166015625
node_106: feature_name=SARS.CoV.2.S1+S2	feature_id[7].value <= threshold=48317.708984375
node_107: feature_name=a-HuIgG_0.03	feature_id[14].value <= threshold=12099.83349609375
node_108: feature_name=SARS.CoV.2.Spike.RBD.His.Bac	feature_id[3].value > threshold=23841.4169921875
node_118: feature_name=SARS.CoV.2.Spike.RBD.rFc	feature_id[5].value > threshold=34554.69140625
node_120: feature_name=SARS.CoV.2.S1	feature_id[6].value <= threshold=36508.716796875
node_121: feature_name=HuIgG_0.10	feature_id[9].value > threshold=24763.3330078125
node_139: feature_name=SARS.CoV.2.Spike.RBD.His.Bac	feature_id[3].value <= threshold=33242.83203125
Class: healthcare workers within 60 days after vaccination	
Rules_26	passed counts:12
node_0: feature_name=HuIgG_0.10	feature_id[9].value > threshold=19120.166015625
node_92: feature_name=a-HuIgG_0.03	feature_id[14].value <= threshold=19665.666015625
node_93: feature_name=SARS.CoV.2.Spike.RBD.His.HEK	feature_id[4].value > threshold=3.0166666507720947
node_105: feature_name=SARS.CoV.2.S1.mFcTag	feature_id[0].value > threshold=54010.166015625
node_305: feature_name=a-MoIgG_0.10	feature_id[16].value > threshold=2382.8333740234375
node_309: feature_name=SARS.CoV.S1.HisTag	feature_id[15].value <= threshold=23460.3330078125
node_310: feature_name=HuIgG_0.03	feature_id[8].value > threshold=6941.33349609375
node_362: feature_name=SARS.CoV.2.Spike.RBD.His.Bac	feature_id[3].value <= threshold=43627.66796875
Class: healthcare workers between 60 and 180 days after vaccination	

Rules_27	passed counts:12
node_0: feature_name=HuIgG_0.10	feature_id[9].value > threshold=19120.166015625
node_92: feature_name=a-HuIgG_0.03	feature_id[14].value <= threshold=19665.666015625
node_93: feature_name=SARS.CoV.2.Spike.RBD.His.HEK	feature_id[4].value > threshold=3.0166666507720947
node_105: feature_name=SARS.CoV.2.S1.mFcTag	feature_id[0].value > threshold=54010.166015625
node_305: feature_name=a-MoIgG_0.10	feature_id[16].value > threshold=2382.8333740234375
node_309: feature_name=SARS.CoV.S1.HisTag	feature_id[15].value <= threshold=23460.3330078125
node_310: feature_name=HuIgG_0.03	feature_id[8].value <= threshold=6941.33349609375
node_311: feature_name=a-HuIgG_0.10	feature_id[13].value > threshold=32620.5
node_351: feature_name=SARS.CoV.S1.RBD.HisTag	feature_id[10].value > threshold=13834.0
node_359: feature_name=HuIgG_0.30	feature_id[12].value > threshold=39323.666015625
Class: healthcare workers within 60 days after vaccination	
Rules_28	passed counts:12
node_0: feature_name=HuIgG_0.10	feature_id[9].value <= threshold=19120.166015625
node_1: feature_name=SARS.CoV.2.S1.RBD.mFc	feature_id[1].value > threshold=3773.8334350585938
node_9: feature_name=a-HuIgG_0.30	feature_id[11].value > threshold=33726.833984375
node_47: feature_name=SARS.CoV.2.S1.mFcTag	feature_id[0].value > threshold=43185.68359375
node_57: feature_name=a-HuIgG_0.03	feature_id[14].value > threshold=10858.0
node_71: feature_name=HuIgG_0.03	feature_id[8].value <= threshold=3796.0
node_72: feature_name=SARS.CoV.2.S1.mFcTag	feature_id[0].value <= threshold=55206.517578125
node_73: feature_name=SARS.CoV.2.S1.mFcTag	feature_id[0].value > threshold=46065.833984375
node_75: feature_name=SARS.CoV.2.S1	feature_id[6].value <= threshold=35627.0
Class: healthcare workers over 180 days after vaccination	

Rules_29	passed counts:12
node_0: feature_name=HuIgG_0.10	feature_id[9].value <= threshold=19120.166015625
node_1: feature_name=SARS.CoV.2.S1.RBD.mFc	feature_id[1].value > threshold=3773.8334350585938
node_9: feature_name=a-HuIgG_0.30	feature_id[11].value > threshold=33726.833984375
node_47: feature_name=SARS.CoV.2.S1.mFcTag	feature_id[0].value > threshold=43185.68359375
node_57: feature_name=a-HuIgG_0.03	feature_id[14].value <= threshold=10858.0
node_58: feature_name=HuIgG_0.30	feature_id[12].value <= threshold=36767.33203125
node_59: feature_name=a-HuIgG_0.10	feature_id[13].value > threshold=21868.0
node_61: feature_name=a-HuIgG_0.03	feature_id[14].value <= threshold=10179.0
node_62: feature_name=a-HuIgG_0.30	feature_id[11].value > threshold=34348.833984375
Class: healthcare workers within 60 days after vaccination	
Rules_30	passed counts:12
node_0: feature_name=HuIgG_0.10	feature_id[9].value <= threshold=19120.166015625
node_1: feature_name=SARS.CoV.2.S1.RBD.mFc	feature_id[1].value > threshold=3773.8334350585938
node_9: feature_name=a-HuIgG_0.30	feature_id[11].value <= threshold=33726.833984375
node_10: feature_name=HuIgG_0.03	feature_id[8].value > threshold=1023.1666564941406
node_20: feature_name=SARS.CoV.S1.HisTag	feature_id[15].value > threshold=400.3000030517578
node_24: feature_name=SARS.CoV.2.Spike.RBD.His.Bac	feature_id[3].value <= threshold=36933.69921875
node_25: feature_name=SARS.CoV.2.S1.RBD.mFc	feature_id[1].value <= threshold=40856.734375
node_26: feature_name=SARS.CoV.S1.HisTag	feature_id[15].value > threshold=15087.41650390625
node_34: feature_name=SARS.CoV.S1.HisTag	feature_id[15].value > threshold=15476.41650390625
node_36: feature_name=a-MoIgG_0.10	feature_id[16].value <= threshold=4103.6666259765625
Class: healthcare workers between 60 and 180 days after vaccination	

Rules_31	passed counts:11
node_0: feature_name=HuIgG_0.10	feature_id[9].value > threshold=19120.166015625
node_92: feature_name=a-HuIgG_0.03	feature_id[14].value <= threshold=19665.666015625
node_93: feature_name=SARS.CoV.2.Spike.RBD.His.HEK	feature_id[4].value > threshold=3.0166666507720947
node_105: feature_name=SARS.CoV.2.S1.mFcTag	feature_id[0].value > threshold=54010.166015625
node_305: feature_name=a-MoIgG_0.10	feature_id[16].value > threshold=2382.8333740234375
node_309: feature_name=SARS.CoV.S1.HisTag	feature_id[15].value <= threshold=23460.3330078125
node_310: feature_name=HuIgG_0.03	feature_id[8].value <= threshold=6941.33349609375
node_311: feature_name=a-HuIgG_0.10	feature_id[13].value <= threshold=32620.5
node_312: feature_name=SARS.CoV.2.Spike.RBD.His.Bac	feature_id[3].value > threshold=31266.0
node_320: feature_name=a-HuIgG_0.30	feature_id[11].value <= threshold=47574.66796875
node_321: feature_name=SARS.CoV.2.S1.RBD.mFc	feature_id[1].value > threshold=47171.166015625
node_323: feature_name=SARS.CoV.2.Spike.RBD.His.Bac	feature_id[3].value > threshold=32929.0
node_325: feature_name=SARS.CoV.2.Spike.RBD.His.Bac	feature_id[3].value > threshold=37127.798828125
node_333: feature_name=a-HuIgG_0.10	feature_id[13].value > threshold=30243.0
node_339: feature_name=a-HuIgG_0.10	feature_id[13].value <= threshold=31594.5
node_340: feature_name=SARS.CoV.2.S1.RBD.mFc	feature_id[1].value <= threshold=53514.666015625
Class: healthcare workers between 60 and 180 days after vaccination	
Rules_32	passed counts:11
node_0: feature_name=HuIgG_0.10	feature_id[9].value > threshold=19120.166015625
node_92: feature_name=a-HuIgG_0.03	feature_id[14].value <= threshold=19665.666015625
node_93: feature_name=SARS.CoV.2.Spike.RBD.His.HEK	feature_id[4].value > threshold=3.0166666507720947
node_105: feature_name=SARS.CoV.2.S1.mFcTag	feature_id[0].value <= threshold=54010.166015625

node_106: feature_name=SARS.CoV.2.S1+S2	feature_id[7].value <= threshold=48317.708984375
node_107: feature_name=a-HuIgG_0.03	feature_id[14].value > threshold=12099.83349609375
node_155: feature_name=SARS.CoV.2.Spike.RBD.His.Bac	feature_id[3].value > threshold=18979.9912109375
node_167: feature_name=a-MoIgG_0.10	feature_id[16].value <= threshold=4979.5
node_168: feature_name=SARS.CoV.2.S1+S2	feature_id[7].value > threshold=36298.75
node_172: feature_name=SARS.CoV.2.S1.RBD.mFc	feature_id[1].value > threshold=40153.283203125
node_182: feature_name=HuIgG_0.10	feature_id[9].value > threshold=21919.833984375
node_186: feature_name=a-HuIgG_0.10	feature_id[13].value <= threshold=28919.0
node_187: feature_name=SARS.CoV.S1.HisTag	feature_id[15].value > threshold=4488.5833740234375
node_189: feature_name=a-HuIgG_0.10	feature_id[13].value > threshold=26194.3330078125
Class: healthcare workers within 60 days after vaccination	
Rules_33	passed counts:11
node_0: feature_name=HuIgG_0.10	feature_id[9].value <= threshold=19120.166015625
node_1: feature_name=SARS.CoV.2.S1.RBD.mFc	feature_id[1].value > threshold=3773.8334350585938
node_9: feature_name=a-HuIgG_0.30	feature_id[11].value > threshold=33726.833984375
node_47: feature_name=SARS.CoV.2.S1.mFcTag	feature_id[0].value > threshold=43185.68359375
node_57: feature_name=a-HuIgG_0.03	feature_id[14].value > threshold=10858.0
node_71: feature_name=HuIgG_0.03	feature_id[8].value > threshold=3796.0
node_83: feature_name=SARS.CoV.2.S1+S2	feature_id[7].value <= threshold=49967.91796875
node_84: feature_name=HuIgG_0.10	feature_id[9].value > threshold=17143.833984375
Class: healthcare workers between 60 and 180 days after vaccination	
Rules_34	passed counts:10
node_0: feature_name=HuIgG_0.10	feature_id[9].value > threshold=19120.166015625

node_92: feature_name=a-HuIgG_0.03	feature_id[14].value <= threshold=19665.666015625
node_93: feature_name=SARS.CoV.2.Spike.RBD.His.HEK	feature_id[4].value > threshold=3.0166666507720947
node_105: feature_name=SARS.CoV.2.S1.mFcTag	feature_id[0].value > threshold=54010.166015625
node_305: feature_name=a-MoIgG_0.10	feature_id[16].value > threshold=2382.8333740234375
node_309: feature_name=SARS.CoV.S1.HisTag	feature_id[15].value <= threshold=23460.3330078125
node_310: feature_name=HuIgG_0.03	feature_id[8].value <= threshold=6941.33349609375
node_311: feature_name=a-HuIgG_0.10	feature_id[13].value <= threshold=32620.5
node_312: feature_name=SARS.CoV.2.Spike.RBD.His.Bac	feature_id[3].value > threshold=31266.0
node_320: feature_name=a-HuIgG_0.30	feature_id[11].value <= threshold=47574.66796875
node_321: feature_name=SARS.CoV.2.S1.RBD.mFc	feature_id[1].value > threshold=47171.166015625
node_323: feature_name=SARS.CoV.2.Spike.RBD.His.Bac	feature_id[3].value > threshold=32929.0
node_325: feature_name=SARS.CoV.2.Spike.RBD.His.Bac	feature_id[3].value <= threshold=37127.798828125
node_326: feature_name=SARS.CoV.2.S1+S2	feature_id[7].value > threshold=49134.966796875
node_328: feature_name=SARS.CoV.2.Spike.RBD.His.HEK	feature_id[4].value <= threshold=35038.25
Class: healthcare workers within 60 days after vaccination	
Rules_35	passed counts:10
node_0: feature_name=HuIgG_0.10	feature_id[9].value > threshold=19120.166015625
node_92: feature_name=a-HuIgG_0.03	feature_id[14].value <= threshold=19665.666015625
node_93: feature_name=SARS.CoV.2.Spike.RBD.His.HEK	feature_id[4].value > threshold=3.0166666507720947
node_105: feature_name=SARS.CoV.2.S1.mFcTag	feature_id[0].value > threshold=54010.166015625
node_305: feature_name=a-MoIgG_0.10	feature_id[16].value > threshold=2382.8333740234375
node_309: feature_name=SARS.CoV.S1.HisTag	feature_id[15].value <= threshold=23460.3330078125
node_310: feature_name=HuIgG_0.03	feature_id[8].value <= threshold=6941.33349609375
node_311: feature_name=a-HuIgG_0.10	feature_id[13].value <= threshold=32620.5

node_312: feature_name=SARS.CoV.2.Spike.RBD.His.Bac	feature_id[3].value > threshold=31266.0
node_320: feature_name=a-HuIgG_0.30	feature_id[11].value <= threshold=47574.66796875
node_321: feature_name=SARS.CoV.2.S1.RBD.mFc	feature_id[1].value <= threshold=47171.166015625
Class: healthcare workers between 60 and 180 days after vaccination	
Rules_36	passed counts:10
node_0: feature_name=HuIgG_0.10	feature_id[9].value > threshold=19120.166015625
node_92: feature_name=a-HuIgG_0.03	feature_id[14].value <= threshold=19665.666015625
node_93: feature_name=SARS.CoV.2.Spike.RBD.His.HEK	feature_id[4].value > threshold=3.0166666507720947
node_105: feature_name=SARS.CoV.2.S1.mFcTag	feature_id[0].value > threshold=54010.166015625
node_305: feature_name=a-MoIgG_0.10	feature_id[16].value > threshold=2382.8333740234375
node_309: feature_name=SARS.CoV.S1.HisTag	feature_id[15].value <= threshold=23460.3330078125
node_310: feature_name=HuIgG_0.03	feature_id[8].value <= threshold=6941.33349609375
node_311: feature_name=a-HuIgG_0.10	feature_id[13].value <= threshold=32620.5
node_312: feature_name=SARS.CoV.2.Spike.RBD.His.Bac	feature_id[3].value <= threshold=31266.0
node_313: feature_name=HuIgG_0.10	feature_id[9].value > threshold=22308.5
node_317: feature_name=SARS.CoV.2.Spike.RBD.rFc	feature_id[5].value <= threshold=47206.232421875
Class: healthcare workers within 60 days after vaccination	
Rules_37	passed counts:10
node_0: feature_name=HuIgG_0.10	feature_id[9].value > threshold=19120.166015625
node_92: feature_name=a-HuIgG_0.03	feature_id[14].value <= threshold=19665.666015625
node_93: feature_name=SARS.CoV.2.Spike.RBD.His.HEK	feature_id[4].value > threshold=3.0166666507720947
node_105: feature_name=SARS.CoV.2.S1.mFcTag	feature_id[0].value <= threshold=54010.166015625
node_106: feature_name=SARS.CoV.2.S1+S2	feature_id[7].value <= threshold=48317.708984375

node_107: feature_name=a-HuIgG_0.03	feature_id[14].value <= threshold=12099.83349609375
node_108: feature_name=SARS.CoV.2.Spike.RBD.His.Bac	feature_id[3].value > threshold=23841.4169921875
node_118: feature_name=SARS.CoV.2.Spike.RBD.rFc	feature_id[5].value > threshold=34554.69140625
node_120: feature_name=SARS.CoV.2.S1	feature_id[6].value <= threshold=36508.716796875
node_121: feature_name=HuIgG_0.10	feature_id[9].value <= threshold=24763.3330078125
node_122: feature_name=SARS.CoV.2.Spike.RBD.rFc	feature_id[5].value <= threshold=42966.541015625
node_123: feature_name=SARS.CoV.2.S1+S2	feature_id[7].value > threshold=43434.54296875
node_135: feature_name=HuIgG_0.10	feature_id[9].value <= threshold=24375.1669921875
Class: healthcare workers within 60 days after vaccination	
Rules_38	passed counts:10
node_0: feature_name=HuIgG_0.10	feature_id[9].value <= threshold=19120.166015625
node_1: feature_name=SARS.CoV.2.S1.RBD.mFc	feature_id[1].value > threshold=3773.8334350585938
node_9: feature_name=a-HuIgG_0.30	feature_id[11].value <= threshold=33726.833984375
node_10: feature_name=HuIgG_0.03	feature_id[8].value <= threshold=1023.1666564941406
node_11: feature_name=SARS.CoV.2.S1.mFcTag	feature_id[0].value > threshold=34194.916015625
node_17: feature_name=HuIgG_0.10	feature_id[9].value <= threshold=7582.83349609375
Class: healthcare workers between 60 and 180 days after vaccination	
Rules_39	passed counts:9
node_0: feature_name=HuIgG_0.10	feature_id[9].value > threshold=19120.166015625
node_92: feature_name=a-HuIgG_0.03	feature_id[14].value > threshold=19665.666015625
node_382: feature_name=a-MoIgG_0.10	feature_id[16].value <= threshold=20281.6669921875
node_383: feature_name=a-HuIgG_0.10	feature_id[13].value <= threshold=33917.66796875
node_384: feature_name=SARS.CoV.2.S1.mFcTag	feature_id[0].value > threshold=52180.75

node_388: feature_name=SARS.CoV.S1.RBD.HisTag	feature_id[10].value <= threshold=52228.166015625
Class: healthcare workers between 60 and 180 days after vaccination	
Rules_40	passed counts:9
node_0: feature_name=HuIgG_0.10	feature_id[9].value > threshold=19120.166015625
node_92: feature_name=a-HuIgG_0.03	feature_id[14].value <= threshold=19665.666015625
node_93: feature_name=SARS.CoV.2.Spike.RBD.His.HEK	feature_id[4].value > threshold=3.0166666507720947
node_105: feature_name=SARS.CoV.2.S1.mFcTag	feature_id[0].value <= threshold=54010.166015625
node_106: feature_name=SARS.CoV.2.S1+S2	feature_id[7].value <= threshold=48317.708984375
node_107: feature_name=a-HuIgG_0.03	feature_id[14].value > threshold=12099.83349609375
node_155: feature_name=SARS.CoV.2.Spike.RBD.His.Bac	feature_id[3].value > threshold=18979.9912109375
node_167: feature_name=a-MoIgG_0.10	feature_id[16].value <= threshold=4979.5
node_168: feature_name=SARS.CoV.2.S1+S2	feature_id[7].value > threshold=36298.75
node_172: feature_name=SARS.CoV.2.S1.RBD.mFc	feature_id[1].value > threshold=40153.283203125
node_182: feature_name=HuIgG_0.10	feature_id[9].value <= threshold=21919.833984375
node_183: feature_name=a-HuIgG_0.30	feature_id[11].value <= threshold=51690.5
Class: healthcare workers between 60 and 180 days after vaccination	
Rules_41	passed counts:9
node_0: feature_name=HuIgG_0.10	feature_id[9].value <= threshold=19120.166015625
node_1: feature_name=SARS.CoV.2.S1.RBD.mFc	feature_id[1].value > threshold=3773.8334350585938
node_9: feature_name=a-HuIgG_0.30	feature_id[11].value <= threshold=33726.833984375
node_10: feature_name=HuIgG_0.03	feature_id[8].value > threshold=1023.1666564941406
node_20: feature_name=SARS.CoV.S1.HisTag	feature_id[15].value > threshold=400.3000030517578
node_24: feature_name=SARS.CoV.2.Spike.RBD.His.Bac	feature_id[3].value <= threshold=36933.69921875

node_25: feature_name=SARS.CoV.2.S1.RBD.mFc	feature_id[1].value > threshold=40856.734375
node_41: feature_name=SARS.CoV.2.S1+S2	feature_id[7].value > threshold=36853.75
Class: healthcare workers between 60 and 180 days after vaccination	
Rules_42	passed counts:8
node_0: feature_name=HuIgG_0.10	feature_id[9].value > threshold=19120.166015625
node_92: feature_name=a-HuIgG_0.03	feature_id[14].value > threshold=19665.666015625
node_382: feature_name=a-MoIgG_0.10	feature_id[16].value <= threshold=20281.6669921875
node_383: feature_name=a-HuIgG_0.10	feature_id[13].value > threshold=33917.66796875
node_391: feature_name=HuIgG_0.10	feature_id[9].value > threshold=25959.0
node_393: feature_name=HuIgG_0.30	feature_id[12].value <= threshold=52448.166015625
node_394: feature_name=a-HuIgG_0.10	feature_id[13].value <= threshold=38348.5
node_395: feature_name=a-HuIgG_0.30	feature_id[11].value > threshold=53098.833984375
Class: healthcare workers between 60 and 180 days after vaccination	
Rules_43	passed counts:8
node_0: feature_name=HuIgG_0.10	feature_id[9].value > threshold=19120.166015625
node_92: feature_name=a-HuIgG_0.03	feature_id[14].value <= threshold=19665.666015625
node_93: feature_name=SARS.CoV.2.Spike.RBD.His.HEK	feature_id[4].value > threshold=3.0166666507720947
node_105: feature_name=SARS.CoV.2.S1.mFcTag	feature_id[0].value <= threshold=54010.166015625
node_106: feature_name=SARS.CoV.2.S1+S2	feature_id[7].value > threshold=48317.708984375
node_228: feature_name=a-MoIgG_0.10	feature_id[16].value > threshold=1652.5
node_230: feature_name=HuIgG_0.10	feature_id[9].value <= threshold=30107.666015625
node_231: feature_name=HuIgG_0.03	feature_id[8].value > threshold=5533.83349609375
node_247: feature_name=HuIgG_0.10	feature_id[9].value > threshold=24231.0

node_261: feature_name=SARS.CoV.S1.RBD.HisTag	feature_id[10].value <= threshold=24179.0419921875
node_262: feature_name=HuIgG_0.10	feature_id[9].value > threshold=25555.333984375
node_266: feature_name=HuIgG_0.10	feature_id[9].value > threshold=25659.6669921875
node_270: feature_name=SARS.CoV.S1.HisTag	feature_id[15].value > threshold=10000.5830078125
node_276: feature_name=SARS.CoV.S1.HisTag	feature_id[15].value <= threshold=13228.125
node_277: feature_name=SARS.CoV.2.Spike.RBD.His.Bac	feature_id[3].value > threshold=33671.91796875
node_279: feature_name=SARS.CoV.2.S1.RBD.mFc	feature_id[1].value <= threshold=52697.666015625
node_280: feature_name=SARS.CoV.2.Spike.RBD.His.Bac	feature_id[3].value > threshold=34984.25
Class: healthcare workers between 60 and 180 days after vaccination	
Rules_44	passed counts:8
node_0: feature_name=HuIgG_0.10	feature_id[9].value > threshold=19120.166015625
node_92: feature_name=a-HuIgG_0.03	feature_id[14].value <= threshold=19665.666015625
node_93: feature_name=SARS.CoV.2.Spike.RBD.His.HEK	feature_id[4].value > threshold=3.0166666507720947
node_105: feature_name=SARS.CoV.2.S1.mFcTag	feature_id[0].value <= threshold=54010.166015625
node_106: feature_name=SARS.CoV.2.S1+S2	feature_id[7].value > threshold=48317.708984375
node_228: feature_name=a-MoIgG_0.10	feature_id[16].value > threshold=1652.5
node_230: feature_name=HuIgG_0.10	feature_id[9].value <= threshold=30107.666015625
node_231: feature_name=HuIgG_0.03	feature_id[8].value > threshold=5533.83349609375
node_247: feature_name=HuIgG_0.10	feature_id[9].value <= threshold=24231.0
node_248: feature_name=SARS.CoV.2.S1.HisTag	feature_id[2].value <= threshold=43585.92578125
node_249: feature_name=HuIgG_0.10	feature_id[9].value > threshold=23668.3330078125
Class: healthcare workers between 60 and 180 days after vaccination	
Rules_45	passed counts:8

node_0: feature_name=HuIgG_0.10	feature_id[9].value > threshold=19120.166015625
node_92: feature_name=a-HuIgG_0.03	feature_id[14].value <= threshold=19665.666015625
node_93: feature_name=SARS.CoV.2.Spike.RBD.His.HEK	feature_id[4].value > threshold=3.0166666507720947
node_105: feature_name=SARS.CoV.2.S1.mFcTag	feature_id[0].value <= threshold=54010.166015625
node_106: feature_name=SARS.CoV.2.S1+S2	feature_id[7].value <= threshold=48317.708984375
node_107: feature_name=a-HuIgG_0.03	feature_id[14].value <= threshold=12099.83349609375
node_108: feature_name=SARS.CoV.2.Spike.RBD.His.Bac	feature_id[3].value > threshold=23841.4169921875
node_118: feature_name=SARS.CoV.2.Spike.RBD.rFc	feature_id[5].value > threshold=34554.69140625
node_120: feature_name=SARS.CoV.2.S1	feature_id[6].value <= threshold=36508.716796875
node_121: feature_name=HuIgG_0.10	feature_id[9].value <= threshold=24763.3330078125
node_122: feature_name=SARS.CoV.2.Spike.RBD.rFc	feature_id[5].value <= threshold=42966.541015625
node_123: feature_name=SARS.CoV.2.S1+S2	feature_id[7].value <= threshold=43434.54296875
node_124: feature_name=SARS.CoV.2.S1+S2	feature_id[7].value <= threshold=42800.94140625
node_125: feature_name=SARS.CoV.2.S1.mFcTag	feature_id[0].value > threshold=47427.1328125
node_129: feature_name=a-MoIgG_0.10	feature_id[16].value <= threshold=5634.0
Class: healthcare workers within 60 days after vaccination	
Rules_46	passed counts:8
node_0: feature_name=HuIgG_0.10	feature_id[9].value <= threshold=19120.166015625
node_1: feature_name=SARS.CoV.2.S1.RBD.mFc	feature_id[1].value > threshold=3773.8334350585938
node_9: feature_name=a-HuIgG_0.30	feature_id[11].value > threshold=33726.833984375
node_47: feature_name=SARS.CoV.2.S1.mFcTag	feature_id[0].value > threshold=43185.68359375
node_57: feature_name=a-HuIgG_0.03	feature_id[14].value > threshold=10858.0
node_71: feature_name=HuIgG_0.03	feature_id[8].value <= threshold=3796.0
node_72: feature_name=SARS.CoV.2.S1.mFcTag	feature_id[0].value <= threshold=55206.517578125

node_73: feature_name=SARS.CoV.2.S1.mFcTag	feature_id[0].value > threshold=46065.833984375
node_75: feature_name=SARS.CoV.2.S1	feature_id[6].value > threshold=35627.0
node_77: feature_name=SARS.CoV.S1.HisTag	feature_id[15].value > threshold=21926.9169921875
Class: healthcare workers over 180 days after vaccination	
Rules_47	passed counts:8
node_0: feature_name=HuIgG_0.10	feature_id[9].value <= threshold=19120.166015625
node_1: feature_name=SARS.CoV.2.S1.RBD.mFc	feature_id[1].value > threshold=3773.8334350585938
node_9: feature_name=a-HuIgG_0.30	feature_id[11].value > threshold=33726.833984375
node_47: feature_name=SARS.CoV.2.S1.mFcTag	feature_id[0].value > threshold=43185.68359375
node_57: feature_name=a-HuIgG_0.03	feature_id[14].value > threshold=10858.0
node_71: feature_name=HuIgG_0.03	feature_id[8].value <= threshold=3796.0
node_72: feature_name=SARS.CoV.2.S1.mFcTag	feature_id[0].value <= threshold=55206.517578125
node_73: feature_name=SARS.CoV.2.S1.mFcTag	feature_id[0].value > threshold=46065.833984375
node_75: feature_name=SARS.CoV.2.S1	feature_id[6].value > threshold=35627.0
node_77: feature_name=SARS.CoV.S1.HisTag	feature_id[15].value <= threshold=21926.9169921875
node_78: feature_name=SARS.CoV.2.S1+S2	feature_id[7].value > threshold=40211.734375
Class: healthcare workers between 60 and 180 days after vaccination	
Rules_48	passed counts:7
node_0: feature_name=HuIgG_0.10	feature_id[9].value > threshold=19120.166015625
node_92: feature_name=a-HuIgG_0.03	feature_id[14].value <= threshold=19665.666015625
node_93: feature_name=SARS.CoV.2.Spike.RBD.His.HEK	feature_id[4].value > threshold=3.0166666507720947
node_105: feature_name=SARS.CoV.2.S1.mFcTag	feature_id[0].value <= threshold=54010.166015625
node_106: feature_name=SARS.CoV.2.S1+S2	feature_id[7].value <= threshold=48317.708984375

node_107: feature_name=a-HuIgG_0.03	feature_id[14].value <= threshold=12099.83349609375
node_108: feature_name=SARS.CoV.2.Spike.RBD.His.Bac	feature_id[3].value > threshold=23841.4169921875
node_118: feature_name=SARS.CoV.2.Spike.RBD.rFc	feature_id[5].value > threshold=34554.69140625
node_120: feature_name=SARS.CoV.2.S1	feature_id[6].value > threshold=36508.716796875
node_142: feature_name=HuIgG_0.30	feature_id[12].value <= threshold=40622.83203125
node_143: feature_name=HuIgG_0.10	feature_id[9].value <= threshold=21439.1669921875
node_144: feature_name=SARS.CoV.2.S1.mFcTag	feature_id[0].value <= threshold=51094.66796875
Class: healthcare workers within 60 days after vaccination	
Rules_49	passed counts:7
node_0: feature_name=HuIgG_0.10	feature_id[9].value > threshold=19120.166015625
node_92: feature_name=a-HuIgG_0.03	feature_id[14].value <= threshold=19665.666015625
node_93: feature_name=SARS.CoV.2.Spike.RBD.His.HEK	feature_id[4].value > threshold=3.0166666507720947
node_105: feature_name=SARS.CoV.2.S1.mFcTag	feature_id[0].value <= threshold=54010.166015625
node_106: feature_name=SARS.CoV.2.S1+S2	feature_id[7].value <= threshold=48317.708984375
node_107: feature_name=a-HuIgG_0.03	feature_id[14].value <= threshold=12099.83349609375
node_108: feature_name=SARS.CoV.2.Spike.RBD.His.Bac	feature_id[3].value > threshold=23841.4169921875
node_118: feature_name=SARS.CoV.2.Spike.RBD.rFc	feature_id[5].value <= threshold=34554.69140625
Class: healthcare workers between 60 and 180 days after vaccination	
Rules_50	passed counts:6
node_0: feature_name=HuIgG_0.10	feature_id[9].value > threshold=19120.166015625
node_92: feature_name=a-HuIgG_0.03	feature_id[14].value <= threshold=19665.666015625
node_93: feature_name=SARS.CoV.2.Spike.RBD.His.HEK	feature_id[4].value > threshold=3.0166666507720947
node_105: feature_name=SARS.CoV.2.S1.mFcTag	feature_id[0].value <= threshold=54010.166015625

node_106: feature_name=SARS.CoV.2.S1+S2	feature_id[7].value > threshold=48317.708984375
node_228: feature_name=a-MoIgG_0.10	feature_id[16].value > threshold=1652.5
node_230: feature_name=HuIgG_0.10	feature_id[9].value <= threshold=30107.666015625
node_231: feature_name=HuIgG_0.03	feature_id[8].value > threshold=5533.83349609375
node_247: feature_name=HuIgG_0.10	feature_id[9].value > threshold=24231.0
node_261: feature_name=SARS.CoV.S1.RBD.HisTag	feature_id[10].value <= threshold=24179.0419921875
node_262: feature_name=HuIgG_0.10	feature_id[9].value > threshold=25555.333984375
node_266: feature_name=HuIgG_0.10	feature_id[9].value <= threshold=25659.6669921875
node_267: feature_name=a-MoIgG_0.10	feature_id[16].value > threshold=4330.1666259765625
Class: healthcare workers between 60 and 180 days after vaccination	
Rules_51	passed counts:6
node_0: feature_name=HuIgG_0.10	feature_id[9].value > threshold=19120.166015625
node_92: feature_name=a-HuIgG_0.03	feature_id[14].value <= threshold=19665.666015625
node_93: feature_name=SARS.CoV.2.Spike.RBD.His.HEK	feature_id[4].value > threshold=3.0166666507720947
node_105: feature_name=SARS.CoV.2.S1.mFcTag	feature_id[0].value <= threshold=54010.166015625
node_106: feature_name=SARS.CoV.2.S1+S2	feature_id[7].value > threshold=48317.708984375
node_228: feature_name=a-MoIgG_0.10	feature_id[16].value > threshold=1652.5
node_230: feature_name=HuIgG_0.10	feature_id[9].value <= threshold=30107.666015625
node_231: feature_name=HuIgG_0.03	feature_id[8].value <= threshold=5533.83349609375
node_232: feature_name=SARS.CoV.2.Spike.RBD.His.HEK	feature_id[4].value > threshold=11116.333374023438
node_234: feature_name=SARS.CoV.2.S1+S2	feature_id[7].value <= threshold=48882.58203125
node_235: feature_name=SARS.CoV.2.Spike.RBD.rFc	feature_id[5].value > threshold=45170.75
Class: healthcare workers within 60 days after vaccination	

Rules_52	passed counts:6
node_0: feature_name=HuIgG_0.10	feature_id[9].value > threshold=19120.166015625
node_92: feature_name=a-HuIgG_0.03	feature_id[14].value <= threshold=19665.666015625
node_93: feature_name=SARS.CoV.2.Spike.RBD.His.HEK	feature_id[4].value > threshold=3.0166666507720947
node_105: feature_name=SARS.CoV.2.S1.mFcTag	feature_id[0].value <= threshold=54010.166015625
node_106: feature_name=SARS.CoV.2.S1+S2	feature_id[7].value <= threshold=48317.708984375
node_107: feature_name=a-HuIgG_0.03	feature_id[14].value > threshold=12099.83349609375
node_155: feature_name=SARS.CoV.2.Spike.RBD.His.Bac	feature_id[3].value > threshold=18979.9912109375
node_167: feature_name=a-MoIgG_0.10	feature_id[16].value > threshold=4979.5
node_201: feature_name=HuIgG_0.10	feature_id[9].value <= threshold=26006.0
node_202: feature_name=HuIgG_0.03	feature_id[8].value > threshold=6080.0
node_216: feature_name=SARS.CoV.2.Spike.RBD.rFc	feature_id[5].value > threshold=43169.25
node_220: feature_name=SARS.CoV.2.Spike.RBD.His.Bac	feature_id[3].value > threshold=34138.75
Class: healthcare workers between 60 and 180 days after vaccination	
Rules_53	passed counts:6
node_0: feature_name=HuIgG_0.10	feature_id[9].value > threshold=19120.166015625
node_92: feature_name=a-HuIgG_0.03	feature_id[14].value <= threshold=19665.666015625
node_93: feature_name=SARS.CoV.2.Spike.RBD.His.HEK	feature_id[4].value > threshold=3.0166666507720947
node_105: feature_name=SARS.CoV.2.S1.mFcTag	feature_id[0].value <= threshold=54010.166015625
node_106: feature_name=SARS.CoV.2.S1+S2	feature_id[7].value <= threshold=48317.708984375
node_107: feature_name=a-HuIgG_0.03	feature_id[14].value > threshold=12099.83349609375
node_155: feature_name=SARS.CoV.2.Spike.RBD.His.Bac	feature_id[3].value > threshold=18979.9912109375
node_167: feature_name=a-MoIgG_0.10	feature_id[16].value > threshold=4979.5
node_201: feature_name=HuIgG_0.10	feature_id[9].value <= threshold=26006.0

node_202: feature_name=HuIgG_0.03	feature_id[8].value > threshold=6080.0
node_216: feature_name=SARS.CoV.2.Spike.RBD.rFc	feature_id[5].value > threshold=43169.25
node_220: feature_name=SARS.CoV.2.Spike.RBD.His.Bac	feature_id[3].value <= threshold=34138.75
node_221: feature_name=SARS.CoV.2.Spike.RBD.His.HEK	feature_id[4].value <= threshold=37141.416015625
Class: healthcare workers within 60 days after vaccination	
Rules_54	passed counts:6
node_0: feature_name=HuIgG_0.10	feature_id[9].value > threshold=19120.166015625
node_92: feature_name=a-HuIgG_0.03	feature_id[14].value <= threshold=19665.666015625
node_93: feature_name=SARS.CoV.2.Spike.RBD.His.HEK	feature_id[4].value > threshold=3.0166666507720947
node_105: feature_name=SARS.CoV.2.S1.mFcTag	feature_id[0].value <= threshold=54010.166015625
node_106: feature_name=SARS.CoV.2.S1+S2	feature_id[7].value <= threshold=48317.708984375
node_107: feature_name=a-HuIgG_0.03	feature_id[14].value > threshold=12099.83349609375
node_155: feature_name=SARS.CoV.2.Spike.RBD.His.Bac	feature_id[3].value > threshold=18979.9912109375
node_167: feature_name=a-MoIgG_0.10	feature_id[16].value <= threshold=4979.5
node_168: feature_name=SARS.CoV.2.S1+S2	feature_id[7].value > threshold=36298.75
node_172: feature_name=SARS.CoV.2.S1.RBD.mFc	feature_id[1].value > threshold=40153.283203125
node_182: feature_name=HuIgG_0.10	feature_id[9].value > threshold=21919.833984375
node_186: feature_name=a-HuIgG_0.10	feature_id[13].value > threshold=28919.0
node_192: feature_name=a-HuIgG_0.10	feature_id[13].value > threshold=31482.6669921875
node_198: feature_name=SARS.CoV.2.S1.mFcTag	feature_id[0].value > threshold=50108.16796875
Class: healthcare workers within 60 days after vaccination	
Rules_55	passed counts:6
node_0: feature_name=HuIgG_0.10	feature_id[9].value > threshold=19120.166015625

node_92: feature_name=a-HuIgG_0.03	feature_id[14].value <= threshold=19665.666015625
node_93: feature_name=SARS.CoV.2.Spike.RBD.His.HEK	feature_id[4].value > threshold=3.0166666507720947
node_105: feature_name=SARS.CoV.2.S1.mFcTag	feature_id[0].value <= threshold=54010.166015625
node_106: feature_name=SARS.CoV.2.S1+S2	feature_id[7].value <= threshold=48317.708984375
node_107: feature_name=a-HuIgG_0.03	feature_id[14].value > threshold=12099.83349609375
node_155: feature_name=SARS.CoV.2.Spike.RBD.His.Bac	feature_id[3].value > threshold=18979.9912109375
node_167: feature_name=a-MoIgG_0.10	feature_id[16].value <= threshold=4979.5
node_168: feature_name=SARS.CoV.2.S1+S2	feature_id[7].value <= threshold=36298.75
node_169: feature_name=SARS.CoV.S1.RBD.HisTag	feature_id[10].value > threshold=6739.833251953125
Class: healthcare workers over 180 days after vaccination	
Rules_56	passed counts:6
node_0: feature_name=HuIgG_0.10	feature_id[9].value > threshold=19120.166015625
node_92: feature_name=a-HuIgG_0.03	feature_id[14].value <= threshold=19665.666015625
node_93: feature_name=SARS.CoV.2.Spike.RBD.His.HEK	feature_id[4].value > threshold=3.0166666507720947
node_105: feature_name=SARS.CoV.2.S1.mFcTag	feature_id[0].value <= threshold=54010.166015625
node_106: feature_name=SARS.CoV.2.S1+S2	feature_id[7].value <= threshold=48317.708984375
node_107: feature_name=a-HuIgG_0.03	feature_id[14].value <= threshold=12099.83349609375
node_108: feature_name=SARS.CoV.2.Spike.RBD.His.Bac	feature_id[3].value > threshold=23841.4169921875
node_118: feature_name=SARS.CoV.2.Spike.RBD.rFc	feature_id[5].value > threshold=34554.69140625
node_120: feature_name=SARS.CoV.2.S1	feature_id[6].value <= threshold=36508.716796875
node_121: feature_name=HuIgG_0.10	feature_id[9].value <= threshold=24763.3330078125
node_122: feature_name=SARS.CoV.2.Spike.RBD.rFc	feature_id[5].value > threshold=42966.541015625
Class: healthcare workers between 60 and 180 days after vaccination	

Rules_57	passed counts:6
node_0: feature_name=HuIgG_0.10	feature_id[9].value > threshold=19120.166015625
node_92: feature_name=a-HuIgG_0.03	feature_id[14].value <= threshold=19665.666015625
node_93: feature_name=SARS.CoV.2.Spike.RBD.His.HEK	feature_id[4].value > threshold=3.0166666507720947
node_105: feature_name=SARS.CoV.2.S1.mFcTag	feature_id[0].value <= threshold=54010.166015625
node_106: feature_name=SARS.CoV.2.S1+S2	feature_id[7].value <= threshold=48317.708984375
node_107: feature_name=a-HuIgG_0.03	feature_id[14].value <= threshold=12099.83349609375
node_108: feature_name=SARS.CoV.2.Spike.RBD.His.Bac	feature_id[3].value > threshold=23841.4169921875
node_118: feature_name=SARS.CoV.2.Spike.RBD.rFc	feature_id[5].value > threshold=34554.69140625
node_120: feature_name=SARS.CoV.2.S1	feature_id[6].value <= threshold=36508.716796875
node_121: feature_name=HuIgG_0.10	feature_id[9].value <= threshold=24763.3330078125
node_122: feature_name=SARS.CoV.2.Spike.RBD.rFc	feature_id[5].value <= threshold=42966.541015625
node_123: feature_name=SARS.CoV.2.S1+S2	feature_id[7].value <= threshold=43434.54296875
node_124: feature_name=SARS.CoV.2.S1+S2	feature_id[7].value > threshold=42800.94140625
Class: healthcare workers between 60 and 180 days after vaccination	
Rules_58	passed counts:6
node_0: feature_name=HuIgG_0.10	feature_id[9].value <= threshold=19120.166015625
node_1: feature_name=SARS.CoV.2.S1.RBD.mFc	feature_id[1].value <= threshold=3773.8334350585938
node_2: feature_name=SARS.CoV.2.S1.HisTag	feature_id[2].value > threshold=-383.86944580078125
node_4: feature_name=SARS.CoV.2.S1	feature_id[6].value <= threshold=435.629638671875
node_5: feature_name=SARS.CoV.2.S1	feature_id[6].value <= threshold=410.7962951660156
Class: unvaccinated healthcare workers	
Rules_59	passed counts:5

node_0: feature_name=HuIgG_0.10	feature_id[9].value > threshold=19120.166015625
node_92: feature_name=a-HuIgG_0.03	feature_id[14].value <= threshold=19665.666015625
node_93: feature_name=SARS.CoV.2.Spike.RBD.His.HEK	feature_id[4].value > threshold=3.0166666507720947
node_105: feature_name=SARS.CoV.2.S1.mFcTag	feature_id[0].value > threshold=54010.166015625
node_305: feature_name=a-MoIgG_0.10	feature_id[16].value > threshold=2382.8333740234375
node_309: feature_name=SARS.CoV.S1.HisTag	feature_id[15].value > threshold=23460.3330078125
node_365: feature_name=a-HuIgG_0.03	feature_id[14].value > threshold=11128.66650390625
node_367: feature_name=SARS.CoV.2.S1.mFcTag	feature_id[0].value > threshold=54294.0
node_371: feature_name=SARS.CoV.2.S1.mFcTag	feature_id[0].value <= threshold=55620.833984375
node_372: feature_name=a-HuIgG_0.10	feature_id[13].value > threshold=28320.6669921875
node_374: feature_name=SARS.CoV.2.S1	feature_id[6].value > threshold=47335.5
node_376: feature_name=SARS.CoV.2.S1+S2	feature_id[7].value > threshold=54353.83203125
node_378: feature_name=SARS.CoV.2.S1+S2	feature_id[7].value > threshold=54529.3828125
Class: healthcare workers within 60 days after vaccination	
Rules_60	passed counts:5
node_0: feature_name=HuIgG_0.10	feature_id[9].value > threshold=19120.166015625
node_92: feature_name=a-HuIgG_0.03	feature_id[14].value <= threshold=19665.666015625
node_93: feature_name=SARS.CoV.2.Spike.RBD.His.HEK	feature_id[4].value > threshold=3.0166666507720947
node_105: feature_name=SARS.CoV.2.S1.mFcTag	feature_id[0].value > threshold=54010.166015625
node_305: feature_name=a-MoIgG_0.10	feature_id[16].value <= threshold=2382.8333740234375
node_306: feature_name=SARS.CoV.2.S1	feature_id[6].value > threshold=34987.400390625
Class: healthcare workers over 180 days after vaccination	
Rules_61	passed counts:5

node_0: feature_name=HuIgG_0.10	feature_id[9].value > threshold=19120.166015625
node_92: feature_name=a-HuIgG_0.03	feature_id[14].value <= threshold=19665.666015625
node_93: feature_name=SARS.CoV.2.Spike.RBD.His.HEK	feature_id[4].value > threshold=3.0166666507720947
node_105: feature_name=SARS.CoV.2.S1.mFcTag	feature_id[0].value <= threshold=54010.166015625
node_106: feature_name=SARS.CoV.2.S1+S2	feature_id[7].value <= threshold=48317.708984375
node_107: feature_name=a-HuIgG_0.03	feature_id[14].value <= threshold=12099.83349609375
node_108: feature_name=SARS.CoV.2.Spike.RBD.His.Bac	feature_id[3].value > threshold=23841.4169921875
node_118: feature_name=SARS.CoV.2.Spike.RBD.rFc	feature_id[5].value > threshold=34554.69140625
node_120: feature_name=SARS.CoV.2.S1	feature_id[6].value <= threshold=36508.716796875
node_121: feature_name=HuIgG_0.10	feature_id[9].value <= threshold=24763.3330078125
node_122: feature_name=SARS.CoV.2.Spike.RBD.rFc	feature_id[5].value <= threshold=42966.541015625
node_123: feature_name=SARS.CoV.2.S1+S2	feature_id[7].value <= threshold=43434.54296875
node_124: feature_name=SARS.CoV.2.S1+S2	feature_id[7].value <= threshold=42800.94140625
node_125: feature_name=SARS.CoV.2.S1.mFcTag	feature_id[0].value <= threshold=47427.1328125
node_126: feature_name=SARS.CoV.2.Spike.RBD.rFc	feature_id[5].value <= threshold=37955.708984375
Class: healthcare workers between 60 and 180 days after vaccination	
Rules_62	passed counts:5
node_0: feature_name=HuIgG_0.10	feature_id[9].value > threshold=19120.166015625
node_92: feature_name=a-HuIgG_0.03	feature_id[14].value <= threshold=19665.666015625
node_93: feature_name=SARS.CoV.2.Spike.RBD.His.HEK	feature_id[4].value > threshold=3.0166666507720947
node_105: feature_name=SARS.CoV.2.S1.mFcTag	feature_id[0].value <= threshold=54010.166015625
node_106: feature_name=SARS.CoV.2.S1+S2	feature_id[7].value <= threshold=48317.708984375
node_107: feature_name=a-HuIgG_0.03	feature_id[14].value <= threshold=12099.83349609375
node_108: feature_name=SARS.CoV.2.Spike.RBD.His.Bac	feature_id[3].value <= threshold=23841.4169921875

node_109: feature_name=SARS.CoV.2.S1+S2	feature_id[7].value <= threshold=36783.25
node_110: feature_name=SARS.CoV.2.S1	feature_id[6].value > threshold=26291.7998046875
Class: healthcare workers between 60 and 180 days after vaccination	
Rules_63	passed counts:5
node_0: feature_name=HuIgG_0.10	feature_id[9].value <= threshold=19120.166015625
node_1: feature_name=SARS.CoV.2.S1.RBD.mFc	feature_id[1].value > threshold=3773.8334350585938
node_9: feature_name=a-HuIgG_0.30	feature_id[11].value <= threshold=33726.833984375
node_10: feature_name=HuIgG_0.03	feature_id[8].value <= threshold=1023.1666564941406
node_11: feature_name=SARS.CoV.2.S1.mFcTag	feature_id[0].value > threshold=34194.916015625
node_17: feature_name=HuIgG_0.10	feature_id[9].value > threshold=7582.83349609375
Class: healthcare workers over 180 days after vaccination	
Rules_64	passed counts:4
node_0: feature_name=HuIgG_0.10	feature_id[9].value > threshold=19120.166015625
node_92: feature_name=a-HuIgG_0.03	feature_id[14].value <= threshold=19665.666015625
node_93: feature_name=SARS.CoV.2.Spike.RBD.His.HEK	feature_id[4].value > threshold=3.0166666507720947
node_105: feature_name=SARS.CoV.2.S1.mFcTag	feature_id[0].value > threshold=54010.166015625
node_305: feature_name=a-MoIgG_0.10	feature_id[16].value > threshold=2382.8333740234375
node_309: feature_name=SARS.CoV.S1.HisTag	feature_id[15].value <= threshold=23460.3330078125
node_310: feature_name=HuIgG_0.03	feature_id[8].value <= threshold=6941.33349609375
node_311: feature_name=a-HuIgG_0.10	feature_id[13].value > threshold=32620.5
node_351: feature_name=SARS.CoV.S1.RBD.HisTag	feature_id[10].value <= threshold=13834.0
node_352: feature_name=SARS.CoV.2.S1	feature_id[6].value > threshold=47365.833984375
Class: healthcare workers between 60 and 180 days after vaccination	

Rules_65	passed counts:4
node_0: feature_name=HuIgG_0.10	feature_id[9].value > threshold=19120.166015625
node_92: feature_name=a-HuIgG_0.03	feature_id[14].value <= threshold=19665.666015625
node_93: feature_name=SARS.CoV.2.Spike.RBD.His.HEK	feature_id[4].value > threshold=3.0166666507720947
node_105: feature_name=SARS.CoV.2.S1.mFcTag	feature_id[0].value > threshold=54010.166015625
node_305: feature_name=a-MoIgG_0.10	feature_id[16].value > threshold=2382.8333740234375
node_309: feature_name=SARS.CoV.S1.HisTag	feature_id[15].value <= threshold=23460.3330078125
node_310: feature_name=HuIgG_0.03	feature_id[8].value <= threshold=6941.33349609375
node_311: feature_name=a-HuIgG_0.10	feature_id[13].value > threshold=32620.5
node_351: feature_name=SARS.CoV.S1.RBD.HisTag	feature_id[10].value <= threshold=13834.0
node_352: feature_name=SARS.CoV.2.S1	feature_id[6].value <= threshold=47365.833984375
node_353: feature_name=SARS.CoV.2.Spike.RBD.His.HEK	feature_id[4].value > threshold=29192.4169921875
Class: healthcare workers within 60 days after vaccination	
Rules_66	passed counts:4
node_0: feature_name=HuIgG_0.10	feature_id[9].value > threshold=19120.166015625
node_92: feature_name=a-HuIgG_0.03	feature_id[14].value <= threshold=19665.666015625
node_93: feature_name=SARS.CoV.2.Spike.RBD.His.HEK	feature_id[4].value > threshold=3.0166666507720947
node_105: feature_name=SARS.CoV.2.S1.mFcTag	feature_id[0].value > threshold=54010.166015625
node_305: feature_name=a-MoIgG_0.10	feature_id[16].value > threshold=2382.8333740234375
node_309: feature_name=SARS.CoV.S1.HisTag	feature_id[15].value <= threshold=23460.3330078125
node_310: feature_name=HuIgG_0.03	feature_id[8].value <= threshold=6941.33349609375
node_311: feature_name=a-HuIgG_0.10	feature_id[13].value <= threshold=32620.5
node_312: feature_name=SARS.CoV.2.Spike.RBD.His.Bac	feature_id[3].value > threshold=31266.0

node_320: feature_name=a-HuIgG_0.30	feature_id[11].value <= threshold=47574.66796875
node_321: feature_name=SARS.CoV.2.S1.RBD.mFc	feature_id[1].value > threshold=47171.166015625
node_323: feature_name=SARS.CoV.2.Spike.RBD.His.Bac	feature_id[3].value > threshold=32929.0
node_325: feature_name=SARS.CoV.2.Spike.RBD.His.Bac	feature_id[3].value > threshold=37127.798828125
node_333: feature_name=a-HuIgG_0.10	feature_id[13].value <= threshold=30243.0
node_334: feature_name=HuIgG_0.10	feature_id[9].value > threshold=22263.333984375
Class: healthcare workers within 60 days after vaccination	
Rules_67	passed counts:4
node_0: feature_name=HuIgG_0.10	feature_id[9].value > threshold=19120.166015625
node_92: feature_name=a-HuIgG_0.03	feature_id[14].value <= threshold=19665.666015625
node_93: feature_name=SARS.CoV.2.Spike.RBD.His.HEK	feature_id[4].value > threshold=3.0166666507720947
node_105: feature_name=SARS.CoV.2.S1.mFcTag	feature_id[0].value > threshold=54010.166015625
node_305: feature_name=a-MoIgG_0.10	feature_id[16].value > threshold=2382.8333740234375
node_309: feature_name=SARS.CoV.S1.HisTag	feature_id[15].value <= threshold=23460.3330078125
node_310: feature_name=HuIgG_0.03	feature_id[8].value <= threshold=6941.33349609375
node_311: feature_name=a-HuIgG_0.10	feature_id[13].value <= threshold=32620.5
node_312: feature_name=SARS.CoV.2.Spike.RBD.His.Bac	feature_id[3].value > threshold=31266.0
node_320: feature_name=a-HuIgG_0.30	feature_id[11].value <= threshold=47574.66796875
node_321: feature_name=SARS.CoV.2.S1.RBD.mFc	feature_id[1].value > threshold=47171.166015625
node_323: feature_name=SARS.CoV.2.Spike.RBD.His.Bac	feature_id[3].value <= threshold=32929.0
Class: healthcare workers between 60 and 180 days after vaccination	
Rules_68	passed counts:4
node_0: feature_name=HuIgG_0.10	feature_id[9].value > threshold=19120.166015625

node_92: feature_name=a-HuIgG_0.03	feature_id[14].value <= threshold=19665.666015625
node_93: feature_name=SARS.CoV.2.Spike.RBD.His.HEK	feature_id[4].value > threshold=3.0166666507720947
node_105: feature_name=SARS.CoV.2.S1.mFcTag	feature_id[0].value > threshold=54010.166015625
node_305: feature_name=a-MoIgG_0.10	feature_id[16].value > threshold=2382.8333740234375
node_309: feature_name=SARS.CoV.S1.HisTag	feature_id[15].value <= threshold=23460.3330078125
node_310: feature_name=HuIgG_0.03	feature_id[8].value <= threshold=6941.33349609375
node_311: feature_name=a-HuIgG_0.10	feature_id[13].value <= threshold=32620.5
node_312: feature_name=SARS.CoV.2.Spike.RBD.His.Bac	feature_id[3].value <= threshold=31266.0
node_313: feature_name=HuIgG_0.10	feature_id[9].value <= threshold=22308.5
node_314: feature_name=a-MoIgG_0.10	feature_id[16].value <= threshold=7511.833251953125
Class: healthcare workers between 60 and 180 days after vaccination	
Rules_69	passed counts:4
node_0: feature_name=HuIgG_0.10	feature_id[9].value > threshold=19120.166015625
node_92: feature_name=a-HuIgG_0.03	feature_id[14].value <= threshold=19665.666015625
node_93: feature_name=SARS.CoV.2.Spike.RBD.His.HEK	feature_id[4].value > threshold=3.0166666507720947
node_105: feature_name=SARS.CoV.2.S1.mFcTag	feature_id[0].value <= threshold=54010.166015625
node_106: feature_name=SARS.CoV.2.S1+S2	feature_id[7].value > threshold=48317.708984375
node_228: feature_name=a-MoIgG_0.10	feature_id[16].value > threshold=1652.5
node_230: feature_name=HuIgG_0.10	feature_id[9].value <= threshold=30107.666015625
node_231: feature_name=HuIgG_0.03	feature_id[8].value > threshold=5533.83349609375
node_247: feature_name=HuIgG_0.10	feature_id[9].value > threshold=24231.0
node_261: feature_name=SARS.CoV.S1.RBD.HisTag	feature_id[10].value <= threshold=24179.0419921875
node_262: feature_name=HuIgG_0.10	feature_id[9].value > threshold=25555.333984375
node_266: feature_name=HuIgG_0.10	feature_id[9].value > threshold=25659.6669921875

node_270: feature_name=SARS.CoV.S1.HisTag	feature_id[15].value <= threshold=10000.5830078125
node_271: feature_name=SARS.CoV.2.Spike.RBD.His.Bac	feature_id[3].value <= threshold=29395.400390625
node_272: feature_name=SARS.CoV.2.S1.HisTag	feature_id[2].value <= threshold=40026.783203125
Class: healthcare workers within 60 days after vaccination	
Rules_70	passed counts:4
node_0: feature_name=HuIgG_0.10	feature_id[9].value > threshold=19120.166015625
node_92: feature_name=a-HuIgG_0.03	feature_id[14].value <= threshold=19665.666015625
node_93: feature_name=SARS.CoV.2.Spike.RBD.His.HEK	feature_id[4].value > threshold=3.0166666507720947
node_105: feature_name=SARS.CoV.2.S1.mFcTag	feature_id[0].value <= threshold=54010.166015625
node_106: feature_name=SARS.CoV.2.S1+S2	feature_id[7].value <= threshold=48317.708984375
node_107: feature_name=a-HuIgG_0.03	feature_id[14].value > threshold=12099.83349609375
node_155: feature_name=SARS.CoV.2.Spike.RBD.His.Bac	feature_id[3].value > threshold=18979.9912109375
node_167: feature_name=a-MoIgG_0.10	feature_id[16].value > threshold=4979.5
node_201: feature_name=HuIgG_0.10	feature_id[9].value <= threshold=26006.0
node_202: feature_name=HuIgG_0.03	feature_id[8].value <= threshold=6080.0
node_203: feature_name=SARS.CoV.2.S1.HisTag	feature_id[2].value > threshold=38575.359375
node_213: feature_name=SARS.CoV.2.Spike.RBD.His.HEK	feature_id[4].value <= threshold=39411.75
Class: healthcare workers between 60 and 180 days after vaccination	
Rules_71	passed counts:4
node_0: feature_name=HuIgG_0.10	feature_id[9].value <= threshold=19120.166015625
node_1: feature_name=SARS.CoV.2.S1.RBD.mFc	feature_id[1].value > threshold=3773.8334350585938
node_9: feature_name=a-HuIgG_0.30	feature_id[11].value > threshold=33726.833984375
node_47: feature_name=SARS.CoV.2.S1.mFcTag	feature_id[0].value > threshold=43185.68359375

node_57: feature_name=a-HuIgG_0.03	feature_id[14].value > threshold=10858.0
node_71: feature_name=HuIgG_0.03	feature_id[8].value <= threshold=3796.0
node_72: feature_name=SARS.CoV.2.S1.mFcTag	feature_id[0].value > threshold=55206.517578125
Class: healthcare workers between 60 and 180 days after vaccination	
Rules_72	passed counts:4
node_0: feature_name=HuIgG_0.10	feature_id[9].value <= threshold=19120.166015625
node_1: feature_name=SARS.CoV.2.S1.RBD.mFc	feature_id[1].value > threshold=3773.8334350585938
node_9: feature_name=a-HuIgG_0.30	feature_id[11].value > threshold=33726.833984375
node_47: feature_name=SARS.CoV.2.S1.mFcTag	feature_id[0].value > threshold=43185.68359375
node_57: feature_name=a-HuIgG_0.03	feature_id[14].value <= threshold=10858.0
node_58: feature_name=HuIgG_0.30	feature_id[12].value <= threshold=36767.33203125
node_59: feature_name=a-HuIgG_0.10	feature_id[13].value > threshold=21868.0
node_61: feature_name=a-HuIgG_0.03	feature_id[14].value > threshold=10179.0
node_65: feature_name=SARS.CoV.2.S1	feature_id[6].value > threshold=34029.666015625
node_67: feature_name=HuIgG_0.10	feature_id[9].value <= threshold=18956.5
Class: healthcare workers between 60 and 180 days after vaccination	
Rules_73	passed counts:4
node_0: feature_name=HuIgG_0.10	feature_id[9].value <= threshold=19120.166015625
node_1: feature_name=SARS.CoV.2.S1.RBD.mFc	feature_id[1].value > threshold=3773.8334350585938
node_9: feature_name=a-HuIgG_0.30	feature_id[11].value <= threshold=33726.833984375
node_10: feature_name=HuIgG_0.03	feature_id[8].value > threshold=1023.1666564941406
node_20: feature_name=SARS.CoV.S1.HisTag	feature_id[15].value > threshold=400.3000030517578
node_24: feature_name=SARS.CoV.2.Spike.RBD.His.Bac	feature_id[3].value > threshold=36933.69921875

node_44: feature_name=SARS.CoV.2.Spike.RBD.His.Bac	feature_id[3].value > threshold=38377.58203125
Class: healthcare workers between 60 and 180 days after vaccination	
Rules_74	passed counts:4
node_0: feature_name=HuIgG_0.10	feature_id[9].value <= threshold=19120.166015625
node_1: feature_name=SARS.CoV.2.S1.RBD.mFc	feature_id[1].value > threshold=3773.8334350585938
node_9: feature_name=a-HuIgG_0.30	feature_id[11].value <= threshold=33726.833984375
node_10: feature_name=HuIgG_0.03	feature_id[8].value > threshold=1023.1666564941406
node_20: feature_name=SARS.CoV.S1.HisTag	feature_id[15].value > threshold=400.3000030517578
node_24: feature_name=SARS.CoV.2.Spike.RBD.His.Bac	feature_id[3].value <= threshold=36933.69921875
node_25: feature_name=SARS.CoV.2.S1.RBD.mFc	feature_id[1].value > threshold=40856.734375
node_41: feature_name=SARS.CoV.2.S1+S2	feature_id[7].value <= threshold=36853.75
Class: healthcare workers over 180 days after vaccination	
Rules_75	passed counts:4
node_0: feature_name=HuIgG_0.10	feature_id[9].value <= threshold=19120.166015625
node_1: feature_name=SARS.CoV.2.S1.RBD.mFc	feature_id[1].value > threshold=3773.8334350585938
node_9: feature_name=a-HuIgG_0.30	feature_id[11].value <= threshold=33726.833984375
node_10: feature_name=HuIgG_0.03	feature_id[8].value > threshold=1023.1666564941406
node_20: feature_name=SARS.CoV.S1.HisTag	feature_id[15].value > threshold=400.3000030517578
node_24: feature_name=SARS.CoV.2.Spike.RBD.His.Bac	feature_id[3].value <= threshold=36933.69921875
node_25: feature_name=SARS.CoV.2.S1.RBD.mFc	feature_id[1].value <= threshold=40856.734375
node_26: feature_name=SARS.CoV.S1.HisTag	feature_id[15].value <= threshold=15087.41650390625
node_27: feature_name=SARS.CoV.2.Spike.RBD.His.HEK	feature_id[4].value <= threshold=10178.375
node_28: feature_name=SARS.CoV.2.S1.RBD.mFc	feature_id[1].value > threshold=33458.650390625

Class: healthcare workers over 180 days after vaccination	
Rules_76	passed counts:3
node_0: feature_name=HuIgG_0.10	feature_id[9].value > threshold=19120.166015625
node_92: feature_name=a-HuIgG_0.03	feature_id[14].value > threshold=19665.666015625
node_382: feature_name=a-MoIgG_0.10	feature_id[16].value > threshold=20281.6669921875
Class: healthcare workers within 60 days after vaccination	
Rules_77	passed counts:3
node_0: feature_name=HuIgG_0.10	feature_id[9].value > threshold=19120.166015625
node_92: feature_name=a-HuIgG_0.03	feature_id[14].value > threshold=19665.666015625
node_382: feature_name=a-MoIgG_0.10	feature_id[16].value <= threshold=20281.6669921875
node_383: feature_name=a-HuIgG_0.10	feature_id[13].value > threshold=33917.66796875
node_391: feature_name=HuIgG_0.10	feature_id[9].value > threshold=25959.0
node_393: feature_name=HuIgG_0.30	feature_id[12].value > threshold=52448.166015625
Class: healthcare workers over 180 days after vaccination	
Rules_78	passed counts:3
node_0: feature_name=HuIgG_0.10	feature_id[9].value > threshold=19120.166015625
node_92: feature_name=a-HuIgG_0.03	feature_id[14].value > threshold=19665.666015625
node_382: feature_name=a-MoIgG_0.10	feature_id[16].value <= threshold=20281.6669921875
node_383: feature_name=a-HuIgG_0.10	feature_id[13].value <= threshold=33917.66796875
node_384: feature_name=SARS.CoV.2.S1.mFcTag	feature_id[0].value <= threshold=52180.75
node_385: feature_name=SARS.CoV.S1.HisTag	feature_id[15].value > threshold=6022.0166015625
Class: healthcare workers over 180 days after vaccination	

Rules_79	passed counts:3
node_0: feature_name=HuIgG_0.10	feature_id[9].value > threshold=19120.166015625
node_92: feature_name=a-HuIgG_0.03	feature_id[14].value <= threshold=19665.666015625
node_93: feature_name=SARS.CoV.2.Spike.RBD.His.HEK	feature_id[4].value > threshold=3.0166666507720947
node_105: feature_name=SARS.CoV.2.S1.mFcTag	feature_id[0].value > threshold=54010.166015625
node_305: feature_name=a-MoIgG_0.10	feature_id[16].value > threshold=2382.8333740234375
node_309: feature_name=SARS.CoV.S1.HisTag	feature_id[15].value > threshold=23460.3330078125
node_365: feature_name=a-HuIgG_0.03	feature_id[14].value > threshold=11128.66650390625
node_367: feature_name=SARS.CoV.2.S1.mFcTag	feature_id[0].value <= threshold=54294.0
node_368: feature_name=SARS.CoV.2.Spike.RBD.His.HEK	feature_id[4].value > threshold=48797.25
Class: healthcare workers between 60 and 180 days after vaccination	
Rules_80	passed counts:3
node_0: feature_name=HuIgG_0.10	feature_id[9].value > threshold=19120.166015625
node_92: feature_name=a-HuIgG_0.03	feature_id[14].value <= threshold=19665.666015625
node_93: feature_name=SARS.CoV.2.Spike.RBD.His.HEK	feature_id[4].value > threshold=3.0166666507720947
node_105: feature_name=SARS.CoV.2.S1.mFcTag	feature_id[0].value > threshold=54010.166015625
node_305: feature_name=a-MoIgG_0.10	feature_id[16].value > threshold=2382.8333740234375
node_309: feature_name=SARS.CoV.S1.HisTag	feature_id[15].value <= threshold=23460.3330078125
node_310: feature_name=HuIgG_0.03	feature_id[8].value <= threshold=6941.33349609375
node_311: feature_name=a-HuIgG_0.10	feature_id[13].value <= threshold=32620.5
node_312: feature_name=SARS.CoV.2.Spike.RBD.His.Bac	feature_id[3].value > threshold=31266.0
node_320: feature_name=a-HuIgG_0.30	feature_id[11].value <= threshold=47574.66796875
node_321: feature_name=SARS.CoV.2.S1.RBD.mFc	feature_id[1].value > threshold=47171.166015625

node_323: feature_name=SARS.CoV.2.Spike.RBD.His.Bac	feature_id[3].value > threshold=32929.0
node_325: feature_name=SARS.CoV.2.Spike.RBD.His.Bac	feature_id[3].value > threshold=37127.798828125
node_333: feature_name=a-HuIgG_0.10	feature_id[13].value <= threshold=30243.0
node_334: feature_name=HuIgG_0.10	feature_id[9].value <= threshold=22263.333984375
node_335: feature_name=a-MoIgG_0.10	feature_id[16].value > threshold=4199.8333740234375
Class: healthcare workers between 60 and 180 days after vaccination	
Rules_81	passed counts:3
node_0: feature_name=HuIgG_0.10	feature_id[9].value > threshold=19120.166015625
node_92: feature_name=a-HuIgG_0.03	feature_id[14].value <= threshold=19665.666015625
node_93: feature_name=SARS.CoV.2.Spike.RBD.His.HEK	feature_id[4].value > threshold=3.0166666507720947
node_105: feature_name=SARS.CoV.2.S1.mFcTag	feature_id[0].value <= threshold=54010.166015625
node_106: feature_name=SARS.CoV.2.S1+S2	feature_id[7].value > threshold=48317.708984375
node_228: feature_name=a-MoIgG_0.10	feature_id[16].value > threshold=1652.5
node_230: feature_name=HuIgG_0.10	feature_id[9].value <= threshold=30107.666015625
node_231: feature_name=HuIgG_0.03	feature_id[8].value > threshold=5533.83349609375
node_247: feature_name=HuIgG_0.10	feature_id[9].value > threshold=24231.0
node_261: feature_name=SARS.CoV.S1.RBD.HisTag	feature_id[10].value <= threshold=24179.0419921875
node_262: feature_name=HuIgG_0.10	feature_id[9].value > threshold=25555.333984375
node_266: feature_name=HuIgG_0.10	feature_id[9].value > threshold=25659.6669921875
node_270: feature_name=SARS.CoV.S1.HisTag	feature_id[15].value > threshold=10000.5830078125
node_276: feature_name=SARS.CoV.S1.HisTag	feature_id[15].value > threshold=13228.125
node_286: feature_name=a-MoIgG_0.10	feature_id[16].value > threshold=10763.83349609375
node_290: feature_name=HuIgG_0.10	feature_id[9].value <= threshold=27228.1669921875
Class: healthcare workers between 60 and 180 days after vaccination	

Rules_82	passed counts:3
node_0: feature_name=HuIgG_0.10	feature_id[9].value > threshold=19120.166015625
node_92: feature_name=a-HuIgG_0.03	feature_id[14].value <= threshold=19665.666015625
node_93: feature_name=SARS.CoV.2.Spike.RBD.His.HEK	feature_id[4].value > threshold=3.0166666507720947
node_105: feature_name=SARS.CoV.2.S1.mFcTag	feature_id[0].value <= threshold=54010.166015625
node_106: feature_name=SARS.CoV.2.S1+S2	feature_id[7].value > threshold=48317.708984375
node_228: feature_name=a-MoIgG_0.10	feature_id[16].value > threshold=1652.5
node_230: feature_name=HuIgG_0.10	feature_id[9].value <= threshold=30107.666015625
node_231: feature_name=HuIgG_0.03	feature_id[8].value > threshold=5533.83349609375
node_247: feature_name=HuIgG_0.10	feature_id[9].value > threshold=24231.0
node_261: feature_name=SARS.CoV.S1.RBD.HisTag	feature_id[10].value <= threshold=24179.0419921875
node_262: feature_name=HuIgG_0.10	feature_id[9].value > threshold=25555.333984375
node_266: feature_name=HuIgG_0.10	feature_id[9].value > threshold=25659.6669921875
node_270: feature_name=SARS.CoV.S1.HisTag	feature_id[15].value <= threshold=10000.5830078125
node_271: feature_name=SARS.CoV.2.Spike.RBD.His.Bac	feature_id[3].value <= threshold=29395.400390625
node_272: feature_name=SARS.CoV.2.S1.HisTag	feature_id[2].value > threshold=40026.783203125
Class: healthcare workers between 60 and 180 days after vaccination	
Rules_83	passed counts:3
node_0: feature_name=HuIgG_0.10	feature_id[9].value > threshold=19120.166015625
node_92: feature_name=a-HuIgG_0.03	feature_id[14].value <= threshold=19665.666015625
node_93: feature_name=SARS.CoV.2.Spike.RBD.His.HEK	feature_id[4].value > threshold=3.0166666507720947
node_105: feature_name=SARS.CoV.2.S1.mFcTag	feature_id[0].value <= threshold=54010.166015625
node_106: feature_name=SARS.CoV.2.S1+S2	feature_id[7].value > threshold=48317.708984375

node_228: feature_name=a-MoIgG_0.10	feature_id[16].value > threshold=1652.5
node_230: feature_name=HuIgG_0.10	feature_id[9].value <= threshold=30107.666015625
node_231: feature_name=HuIgG_0.03	feature_id[8].value > threshold=5533.83349609375
node_247: feature_name=HuIgG_0.10	feature_id[9].value <= threshold=24231.0
node_248: feature_name=SARS.CoV.2.S1.HisTag	feature_id[2].value <= threshold=43585.92578125
node_249: feature_name=HuIgG_0.10	feature_id[9].value <= threshold=23668.3330078125
node_250: feature_name=SARS.CoV.2.S1+S2	feature_id[7].value <= threshold=49682.4921875
Class: healthcare workers within 60 days after vaccination	
Rules_84	passed counts:3
node_0: feature_name=HuIgG_0.10	feature_id[9].value > threshold=19120.166015625
node_92: feature_name=a-HuIgG_0.03	feature_id[14].value <= threshold=19665.666015625
node_93: feature_name=SARS.CoV.2.Spike.RBD.His.HEK	feature_id[4].value > threshold=3.0166666507720947
node_105: feature_name=SARS.CoV.2.S1.mFcTag	feature_id[0].value <= threshold=54010.166015625
node_106: feature_name=SARS.CoV.2.S1+S2	feature_id[7].value > threshold=48317.708984375
node_228: feature_name=a-MoIgG_0.10	feature_id[16].value > threshold=1652.5
node_230: feature_name=HuIgG_0.10	feature_id[9].value <= threshold=30107.666015625
node_231: feature_name=HuIgG_0.03	feature_id[8].value <= threshold=5533.83349609375
node_232: feature_name=SARS.CoV.2.Spike.RBD.His.HEK	feature_id[4].value > threshold=11116.333374023438
node_234: feature_name=SARS.CoV.2.S1+S2	feature_id[7].value > threshold=48882.58203125
node_240: feature_name=a-HuIgG_0.03	feature_id[14].value <= threshold=10994.83349609375
node_241: feature_name=a-HuIgG_0.30	feature_id[11].value > threshold=41039.83203125
node_243: feature_name=SARS.CoV.2.Spike.RBD.rFc	feature_id[5].value > threshold=48191.583984375
Class: healthcare workers within 60 days after vaccination	

Rules_85	passed counts:3
node_0: feature_name=HuIgG_0.10	feature_id[9].value > threshold=19120.166015625
node_92: feature_name=a-HuIgG_0.03	feature_id[14].value <= threshold=19665.666015625
node_93: feature_name=SARS.CoV.2.Spike.RBD.His.HEK	feature_id[4].value > threshold=3.0166666507720947
node_105: feature_name=SARS.CoV.2.S1.mFcTag	feature_id[0].value <= threshold=54010.166015625
node_106: feature_name=SARS.CoV.2.S1+S2	feature_id[7].value > threshold=48317.708984375
node_228: feature_name=a-MoIgG_0.10	feature_id[16].value > threshold=1652.5
node_230: feature_name=HuIgG_0.10	feature_id[9].value <= threshold=30107.666015625
node_231: feature_name=HuIgG_0.03	feature_id[8].value <= threshold=5533.83349609375
node_232: feature_name=SARS.CoV.2.Spike.RBD.His.HEK	feature_id[4].value > threshold=11116.333374023438
node_234: feature_name=SARS.CoV.2.S1+S2	feature_id[7].value > threshold=48882.58203125
node_240: feature_name=a-HuIgG_0.03	feature_id[14].value <= threshold=10994.83349609375
node_241: feature_name=a-HuIgG_0.30	feature_id[11].value > threshold=41039.83203125
node_243: feature_name=SARS.CoV.2.Spike.RBD.rFc	feature_id[5].value <= threshold=48191.583984375
Class: healthcare workers between 60 and 180 days after vaccination	
Rules_86	passed counts:3
node_0: feature_name=HuIgG_0.10	feature_id[9].value > threshold=19120.166015625
node_92: feature_name=a-HuIgG_0.03	feature_id[14].value <= threshold=19665.666015625
node_93: feature_name=SARS.CoV.2.Spike.RBD.His.HEK	feature_id[4].value > threshold=3.0166666507720947
node_105: feature_name=SARS.CoV.2.S1.mFcTag	feature_id[0].value <= threshold=54010.166015625
node_106: feature_name=SARS.CoV.2.S1+S2	feature_id[7].value > threshold=48317.708984375
node_228: feature_name=a-MoIgG_0.10	feature_id[16].value > threshold=1652.5
node_230: feature_name=HuIgG_0.10	feature_id[9].value <= threshold=30107.666015625
node_231: feature_name=HuIgG_0.03	feature_id[8].value <= threshold=5533.83349609375

node_232: feature_name=SARS.CoV.2.Spike.RBD.His.HEK	feature_id[4].value > threshold=11116.333374023438
node_234: feature_name=SARS.CoV.2.S1+S2	feature_id[7].value <= threshold=48882.58203125
node_235: feature_name=SARS.CoV.2.Spike.RBD.rFc	feature_id[5].value <= threshold=45170.75
node_236: feature_name=a-HuIgG_0.03	feature_id[14].value > threshold=11110.0
Class: healthcare workers between 60 and 180 days after vaccination	
Rules_87	passed counts:3
node_0: feature_name=HuIgG_0.10	feature_id[9].value > threshold=19120.166015625
node_92: feature_name=a-HuIgG_0.03	feature_id[14].value <= threshold=19665.666015625
node_93: feature_name=SARS.CoV.2.Spike.RBD.His.HEK	feature_id[4].value > threshold=3.0166666507720947
node_105: feature_name=SARS.CoV.2.S1.mFcTag	feature_id[0].value <= threshold=54010.166015625
node_106: feature_name=SARS.CoV.2.S1+S2	feature_id[7].value <= threshold=48317.708984375
node_107: feature_name=a-HuIgG_0.03	feature_id[14].value > threshold=12099.83349609375
node_155: feature_name=SARS.CoV.2.Spike.RBD.His.Bac	feature_id[3].value > threshold=18979.9912109375
node_167: feature_name=a-MoIgG_0.10	feature_id[16].value <= threshold=4979.5
node_168: feature_name=SARS.CoV.2.S1+S2	feature_id[7].value > threshold=36298.75
node_172: feature_name=SARS.CoV.2.S1.RBD.mFc	feature_id[1].value > threshold=40153.283203125
node_182: feature_name=HuIgG_0.10	feature_id[9].value > threshold=21919.833984375
node_186: feature_name=a-HuIgG_0.10	feature_id[13].value > threshold=28919.0
node_192: feature_name=a-HuIgG_0.10	feature_id[13].value <= threshold=31482.6669921875
node_193: feature_name=a-HuIgG_0.03	feature_id[14].value <= threshold=12541.0
Class: healthcare workers within 60 days after vaccination	
Rules_88	passed counts:3
node_0: feature_name=HuIgG_0.10	feature_id[9].value > threshold=19120.166015625

node_92: feature_name=a-HuIgG_0.03	feature_id[14].value <= threshold=19665.666015625
node_93: feature_name=SARS.CoV.2.Spike.RBD.His.HEK	feature_id[4].value > threshold=3.0166666507720947
node_105: feature_name=SARS.CoV.2.S1.mFcTag	feature_id[0].value <= threshold=54010.166015625
node_106: feature_name=SARS.CoV.2.S1+S2	feature_id[7].value <= threshold=48317.708984375
node_107: feature_name=a-HuIgG_0.03	feature_id[14].value > threshold=12099.83349609375
node_155: feature_name=SARS.CoV.2.Spike.RBD.His.Bac	feature_id[3].value <= threshold=18979.9912109375
node_156: feature_name=a-MoIgG_0.10	feature_id[16].value > threshold=1739.1666259765625
node_160: feature_name=HuIgG_0.30	feature_id[12].value > threshold=34829.83203125
node_162: feature_name=HuIgG_0.03	feature_id[8].value > threshold=6681.166748046875
node_164: feature_name=HuIgG_0.30	feature_id[12].value <= threshold=40459.166015625
Class: healthcare workers within 60 days after vaccination	
Rules_89	passed counts:3
node_0: feature_name=HuIgG_0.10	feature_id[9].value > threshold=19120.166015625
node_92: feature_name=a-HuIgG_0.03	feature_id[14].value <= threshold=19665.666015625
node_93: feature_name=SARS.CoV.2.Spike.RBD.His.HEK	feature_id[4].value > threshold=3.0166666507720947
node_105: feature_name=SARS.CoV.2.S1.mFcTag	feature_id[0].value <= threshold=54010.166015625
node_106: feature_name=SARS.CoV.2.S1+S2	feature_id[7].value <= threshold=48317.708984375
node_107: feature_name=a-HuIgG_0.03	feature_id[14].value <= threshold=12099.83349609375
node_108: feature_name=SARS.CoV.2.Spike.RBD.His.Bac	feature_id[3].value > threshold=23841.4169921875
node_118: feature_name=SARS.CoV.2.Spike.RBD.rFc	feature_id[5].value > threshold=34554.69140625
node_120: feature_name=SARS.CoV.2.S1	feature_id[6].value > threshold=36508.716796875
node_142: feature_name=HuIgG_0.30	feature_id[12].value > threshold=40622.83203125
node_150: feature_name=SARS.CoV.S1.RBD.HisTag	feature_id[10].value > threshold=9578.58349609375
node_152: feature_name=SARS.CoV.2.Spike.RBD.His.Bac	feature_id[3].value <= threshold=30984.7919921875

Class: healthcare workers between 60 and 180 days after vaccination	
Rules_90	passed counts:3
node_0: feature_name=HuIgG_0.10	feature_id[9].value > threshold=19120.166015625
node_92: feature_name=a-HuIgG_0.03	feature_id[14].value <= threshold=19665.666015625
node_93: feature_name=SARS.CoV.2.Spike.RBD.His.HEK	feature_id[4].value > threshold=3.0166666507720947
node_105: feature_name=SARS.CoV.2.S1.mFcTag	feature_id[0].value <= threshold=54010.166015625
node_106: feature_name=SARS.CoV.2.S1+S2	feature_id[7].value <= threshold=48317.708984375
node_107: feature_name=a-HuIgG_0.03	feature_id[14].value <= threshold=12099.83349609375
node_108: feature_name=SARS.CoV.2.Spike.RBD.His.Bac	feature_id[3].value > threshold=23841.4169921875
node_118: feature_name=SARS.CoV.2.Spike.RBD.rFc	feature_id[5].value > threshold=34554.69140625
node_120: feature_name=SARS.CoV.2.S1	feature_id[6].value > threshold=36508.716796875
node_142: feature_name=HuIgG_0.30	feature_id[12].value <= threshold=40622.83203125
node_143: feature_name=HuIgG_0.10	feature_id[9].value <= threshold=21439.1669921875
node_144: feature_name=SARS.CoV.2.S1.mFcTag	feature_id[0].value > threshold=51094.66796875
node_146: feature_name=a-HuIgG_0.03	feature_id[14].value > threshold=9792.33349609375
Class: healthcare workers between 60 and 180 days after vaccination	
Rules_91	passed counts:3
node_0: feature_name=HuIgG_0.10	feature_id[9].value > threshold=19120.166015625
node_92: feature_name=a-HuIgG_0.03	feature_id[14].value <= threshold=19665.666015625
node_93: feature_name=SARS.CoV.2.Spike.RBD.His.HEK	feature_id[4].value <= threshold=3.0166666507720947
node_94: feature_name=SARS.CoV.2.Spike.RBD.His.Bac	feature_id[3].value <= threshold=-51.85277557373047
node_95: feature_name=SARS.CoV.2.S1.RBD.mFc	feature_id[1].value <= threshold=-141.8333387374878
Class: healthcare workers within 60 days after vaccination	

Rules_92	passed counts:3
node_0: feature_name=HuIgG_0.10	feature_id[9].value <= threshold=19120.166015625
node_1: feature_name=SARS.CoV.2.S1.RBD.mFc	feature_id[1].value > threshold=3773.8334350585938
node_9: feature_name=a-HuIgG_0.30	feature_id[11].value > threshold=33726.833984375
node_47: feature_name=SARS.CoV.2.S1.mFcTag	feature_id[0].value > threshold=43185.68359375
node_57: feature_name=a-HuIgG_0.03	feature_id[14].value > threshold=10858.0
node_71: feature_name=HuIgG_0.03	feature_id[8].value > threshold=3796.0
node_83: feature_name=SARS.CoV.2.S1+S2	feature_id[7].value > threshold=49967.91796875
node_87: feature_name=HuIgG_0.10	feature_id[9].value <= threshold=18981.666015625
Class: healthcare workers within 60 days after vaccination	
Rules_93	passed counts:3
node_0: feature_name=HuIgG_0.10	feature_id[9].value <= threshold=19120.166015625
node_1: feature_name=SARS.CoV.2.S1.RBD.mFc	feature_id[1].value > threshold=3773.8334350585938
node_9: feature_name=a-HuIgG_0.30	feature_id[11].value > threshold=33726.833984375
node_47: feature_name=SARS.CoV.2.S1.mFcTag	feature_id[0].value > threshold=43185.68359375
node_57: feature_name=a-HuIgG_0.03	feature_id[14].value > threshold=10858.0
node_71: feature_name=HuIgG_0.03	feature_id[8].value <= threshold=3796.0
node_72: feature_name=SARS.CoV.2.S1.mFcTag	feature_id[0].value <= threshold=55206.517578125
node_73: feature_name=SARS.CoV.2.S1.mFcTag	feature_id[0].value <= threshold=46065.833984375
Class: healthcare workers between 60 and 180 days after vaccination	
Rules_94	passed counts:3
node_0: feature_name=HuIgG_0.10	feature_id[9].value <= threshold=19120.166015625

node_1: feature_name=SARS.CoV.2.S1.RBD.mFc	feature_id[1].value > threshold=3773.8334350585938
node_9: feature_name=a-HuIgG_0.30	feature_id[11].value > threshold=33726.833984375
node_47: feature_name=SARS.CoV.2.S1.mFcTag	feature_id[0].value > threshold=43185.68359375
node_57: feature_name=a-HuIgG_0.03	feature_id[14].value <= threshold=10858.0
node_58: feature_name=HuIgG_0.30	feature_id[12].value > threshold=36767.33203125
Class: healthcare workers between 60 and 180 days after vaccination	
Rules_95	passed counts:3
node_0: feature_name=HuIgG_0.10	feature_id[9].value <= threshold=19120.166015625
node_1: feature_name=SARS.CoV.2.S1.RBD.mFc	feature_id[1].value > threshold=3773.8334350585938
node_9: feature_name=a-HuIgG_0.30	feature_id[11].value > threshold=33726.833984375
node_47: feature_name=SARS.CoV.2.S1.mFcTag	feature_id[0].value > threshold=43185.68359375
node_57: feature_name=a-HuIgG_0.03	feature_id[14].value <= threshold=10858.0
node_58: feature_name=HuIgG_0.30	feature_id[12].value <= threshold=36767.33203125
node_59: feature_name=a-HuIgG_0.10	feature_id[13].value > threshold=21868.0
node_61: feature_name=a-HuIgG_0.03	feature_id[14].value > threshold=10179.0
node_65: feature_name=SARS.CoV.2.S1	feature_id[6].value <= threshold=34029.666015625
Class: healthcare workers within 60 days after vaccination	
Rules_96	passed counts:3
node_0: feature_name=HuIgG_0.10	feature_id[9].value <= threshold=19120.166015625
node_1: feature_name=SARS.CoV.2.S1.RBD.mFc	feature_id[1].value > threshold=3773.8334350585938
node_9: feature_name=a-HuIgG_0.30	feature_id[11].value > threshold=33726.833984375
node_47: feature_name=SARS.CoV.2.S1.mFcTag	feature_id[0].value <= threshold=43185.68359375
node_48: feature_name=SARS.CoV.S1.RBD.HisTag	feature_id[10].value > threshold=3416.129638671875

node_52: feature_name=SARS.CoV.2.S1.HisTag	feature_id[2].value <= threshold=7094.0
node_53: feature_name=a-HuIgG_0.30	feature_id[11].value <= threshold=42229.833984375
Class: healthcare workers over 180 days after vaccination	
Rules_97	passed counts:3
node_0: feature_name=HuIgG_0.10	feature_id[9].value <= threshold=19120.166015625
node_1: feature_name=SARS.CoV.2.S1.RBD.mFc	feature_id[1].value > threshold=3773.8334350585938
node_9: feature_name=a-HuIgG_0.30	feature_id[11].value > threshold=33726.833984375
node_47: feature_name=SARS.CoV.2.S1.mFcTag	feature_id[0].value <= threshold=43185.68359375
node_48: feature_name=SARS.CoV.S1.RBD.HisTag	feature_id[10].value <= threshold=3416.129638671875
node_49: feature_name=SARS.CoV.2.S1.HisTag	feature_id[2].value > threshold=4582.9832763671875
Class: unvaccinated healthcare workers	
Rules_98	passed counts:3
node_0: feature_name=HuIgG_0.10	feature_id[9].value <= threshold=19120.166015625
node_1: feature_name=SARS.CoV.2.S1.RBD.mFc	feature_id[1].value > threshold=3773.8334350585938
node_9: feature_name=a-HuIgG_0.30	feature_id[11].value <= threshold=33726.833984375
node_10: feature_name=HuIgG_0.03	feature_id[8].value > threshold=1023.1666564941406
node_20: feature_name=SARS.CoV.S1.HisTag	feature_id[15].value > threshold=400.3000030517578
node_24: feature_name=SARS.CoV.2.Spike.RBD.His.Bac	feature_id[3].value > threshold=36933.69921875
node_44: feature_name=SARS.CoV.2.Spike.RBD.His.Bac	feature_id[3].value <= threshold=38377.58203125
Class: healthcare workers within 60 days after vaccination	
Rules_99	passed counts:3
node_0: feature_name=HuIgG_0.10	feature_id[9].value <= threshold=19120.166015625

node_1: feature_name=SARS.CoV.2.S1.RBD.mFc	feature_id[1].value > threshold=3773.8334350585938
node_9: feature_name=a-HuIgG_0.30	feature_id[11].value <= threshold=33726.833984375
node_10: feature_name=HuIgG_0.03	feature_id[8].value <= threshold=1023.1666564941406
node_11: feature_name=SARS.CoV.2.S1.mFcTag	feature_id[0].value <= threshold=34194.916015625
node_12: feature_name=SARS.CoV.2.S1+S2	feature_id[7].value > threshold=26709.75
node_14: feature_name=SARS.CoV.2.Spike.RBD.His.HEK	feature_id[4].value > threshold=8029.208251953125
Class: healthcare workers over 180 days after vaccination	
Rules_100	passed counts:2
node_0: feature_name=HuIgG_0.10	feature_id[9].value > threshold=19120.166015625
node_92: feature_name=a-HuIgG_0.03	feature_id[14].value > threshold=19665.666015625
node_382: feature_name=a-MoIgG_0.10	feature_id[16].value <= threshold=20281.6669921875
node_383: feature_name=a-HuIgG_0.10	feature_id[13].value > threshold=33917.66796875
node_391: feature_name=HuIgG_0.10	feature_id[9].value > threshold=25959.0
node_393: feature_name=HuIgG_0.30	feature_id[12].value <= threshold=52448.166015625
node_394: feature_name=a-HuIgG_0.10	feature_id[13].value > threshold=38348.5
Class: healthcare workers over 180 days after vaccination	
Rules_101	passed counts:2
node_0: feature_name=HuIgG_0.10	feature_id[9].value > threshold=19120.166015625
node_92: feature_name=a-HuIgG_0.03	feature_id[14].value <= threshold=19665.666015625
node_93: feature_name=SARS.CoV.2.Spike.RBD.His.HEK	feature_id[4].value > threshold=3.0166666507720947
node_105: feature_name=SARS.CoV.2.S1.mFcTag	feature_id[0].value > threshold=54010.166015625
node_305: feature_name=a-MoIgG_0.10	feature_id[16].value > threshold=2382.8333740234375
node_309: feature_name=SARS.CoV.S1.HisTag	feature_id[15].value > threshold=23460.3330078125

node_365: feature_name=a-HuIgG_0.03	feature_id[14].value > threshold=11128.66650390625
node_367: feature_name=SARS.CoV.2.S1.mFcTag	feature_id[0].value > threshold=54294.0
node_371: feature_name=SARS.CoV.2.S1.mFcTag	feature_id[0].value <= threshold=55620.833984375
node_372: feature_name=a-HuIgG_0.10	feature_id[13].value > threshold=28320.6669921875
node_374: feature_name=SARS.CoV.2.S1	feature_id[6].value > threshold=47335.5
node_376: feature_name=SARS.CoV.2.S1+S2	feature_id[7].value > threshold=54353.83203125
node_378: feature_name=SARS.CoV.2.S1+S2	feature_id[7].value <= threshold=54529.3828125
Class: healthcare workers between 60 and 180 days after vaccination	
Rules_102	passed counts:2
node_0: feature_name=HuIgG_0.10	feature_id[9].value > threshold=19120.166015625
node_92: feature_name=a-HuIgG_0.03	feature_id[14].value <= threshold=19665.666015625
node_93: feature_name=SARS.CoV.2.Spike.RBD.His.HEK	feature_id[4].value > threshold=3.0166666507720947
node_105: feature_name=SARS.CoV.2.S1.mFcTag	feature_id[0].value > threshold=54010.166015625
node_305: feature_name=a-MoIgG_0.10	feature_id[16].value > threshold=2382.8333740234375
node_309: feature_name=SARS.CoV.S1.HisTag	feature_id[15].value > threshold=23460.3330078125
node_365: feature_name=a-HuIgG_0.03	feature_id[14].value > threshold=11128.66650390625
node_367: feature_name=SARS.CoV.2.S1.mFcTag	feature_id[0].value <= threshold=54294.0
node_368: feature_name=SARS.CoV.2.Spike.RBD.His.HEK	feature_id[4].value <= threshold=48797.25
Class: healthcare workers within 60 days after vaccination	
Rules_103	passed counts:2
node_0: feature_name=HuIgG_0.10	feature_id[9].value > threshold=19120.166015625
node_92: feature_name=a-HuIgG_0.03	feature_id[14].value <= threshold=19665.666015625
node_93: feature_name=SARS.CoV.2.Spike.RBD.His.HEK	feature_id[4].value > threshold=3.0166666507720947

node_105: feature_name=SARS.CoV.2.S1.mFcTag	feature_id[0].value > threshold=54010.166015625
node_305: feature_name=a-MoIgG_0.10	feature_id[16].value > threshold=2382.8333740234375
node_309: feature_name=SARS.CoV.S1.HisTag	feature_id[15].value > threshold=23460.3330078125
node_365: feature_name=a-HuIgG_0.03	feature_id[14].value <= threshold=11128.66650390625
Class: healthcare workers between 60 and 180 days after vaccination	
Rules_104	passed counts:2
node_0: feature_name=HuIgG_0.10	feature_id[9].value > threshold=19120.166015625
node_92: feature_name=a-HuIgG_0.03	feature_id[14].value <= threshold=19665.666015625
node_93: feature_name=SARS.CoV.2.Spike.RBD.His.HEK	feature_id[4].value > threshold=3.0166666507720947
node_105: feature_name=SARS.CoV.2.S1.mFcTag	feature_id[0].value > threshold=54010.166015625
node_305: feature_name=a-MoIgG_0.10	feature_id[16].value > threshold=2382.8333740234375
node_309: feature_name=SARS.CoV.S1.HisTag	feature_id[15].value <= threshold=23460.3330078125
node_310: feature_name=HuIgG_0.03	feature_id[8].value <= threshold=6941.33349609375
node_311: feature_name=a-HuIgG_0.10	feature_id[13].value > threshold=32620.5
node_351: feature_name=SARS.CoV.S1.RBD.HisTag	feature_id[10].value <= threshold=13834.0
node_352: feature_name=SARS.CoV.2.S1	feature_id[6].value <= threshold=47365.833984375
node_353: feature_name=SARS.CoV.2.Spike.RBD.His.HEK	feature_id[4].value <= threshold=29192.4169921875
node_354: feature_name=a-MoIgG_0.10	feature_id[16].value > threshold=3888.6666259765625
Class: healthcare workers between 60 and 180 days after vaccination	
Rules_105	passed counts:2
node_0: feature_name=HuIgG_0.10	feature_id[9].value > threshold=19120.166015625
node_92: feature_name=a-HuIgG_0.03	feature_id[14].value <= threshold=19665.666015625
node_93: feature_name=SARS.CoV.2.Spike.RBD.His.HEK	feature_id[4].value > threshold=3.0166666507720947

node_105: feature_name=SARS.CoV.2.S1.mFcTag	feature_id[0].value > threshold=54010.166015625
node_305: feature_name=a-MoIgG_0.10	feature_id[16].value > threshold=2382.8333740234375
node_309: feature_name=SARS.CoV.S1.HisTag	feature_id[15].value <= threshold=23460.3330078125
node_310: feature_name=HuIgG_0.03	feature_id[8].value <= threshold=6941.33349609375
node_311: feature_name=a-HuIgG_0.10	feature_id[13].value <= threshold=32620.5
node_312: feature_name=SARS.CoV.2.Spike.RBD.His.Bac	feature_id[3].value > threshold=31266.0
node_320: feature_name=a-HuIgG_0.30	feature_id[11].value <= threshold=47574.66796875
node_321: feature_name=SARS.CoV.2.S1.RBD.mFc	feature_id[1].value > threshold=47171.166015625
node_323: feature_name=SARS.CoV.2.Spike.RBD.His.Bac	feature_id[3].value > threshold=32929.0
node_325: feature_name=SARS.CoV.2.Spike.RBD.His.Bac	feature_id[3].value > threshold=37127.798828125
node_333: feature_name=a-HuIgG_0.10	feature_id[13].value > threshold=30243.0
node_339: feature_name=a-HuIgG_0.10	feature_id[13].value > threshold=31594.5
node_343: feature_name=SARS.CoV.2.Spike.RBD.His.HEK	feature_id[4].value > threshold=38606.166015625
Class: healthcare workers within 60 days after vaccination	
Rules_106	passed counts:2
node_0: feature_name=HuIgG_0.10	feature_id[9].value > threshold=19120.166015625
node_92: feature_name=a-HuIgG_0.03	feature_id[14].value <= threshold=19665.666015625
node_93: feature_name=SARS.CoV.2.Spike.RBD.His.HEK	feature_id[4].value > threshold=3.0166666507720947
node_105: feature_name=SARS.CoV.2.S1.mFcTag	feature_id[0].value > threshold=54010.166015625
node_305: feature_name=a-MoIgG_0.10	feature_id[16].value > threshold=2382.8333740234375
node_309: feature_name=SARS.CoV.S1.HisTag	feature_id[15].value <= threshold=23460.3330078125
node_310: feature_name=HuIgG_0.03	feature_id[8].value <= threshold=6941.33349609375
node_311: feature_name=a-HuIgG_0.10	feature_id[13].value <= threshold=32620.5
node_312: feature_name=SARS.CoV.2.Spike.RBD.His.Bac	feature_id[3].value > threshold=31266.0

node_320: feature_name=a-HuIgG_0.30	feature_id[11].value <= threshold=47574.66796875
node_321: feature_name=SARS.CoV.2.S1.RBD.mFc	feature_id[1].value > threshold=47171.166015625
node_323: feature_name=SARS.CoV.2.Spike.RBD.His.Bac	feature_id[3].value > threshold=32929.0
node_325: feature_name=SARS.CoV.2.Spike.RBD.His.Bac	feature_id[3].value > threshold=37127.798828125
node_333: feature_name=a-HuIgG_0.10	feature_id[13].value <= threshold=30243.0
node_334: feature_name=HuIgG_0.10	feature_id[9].value <= threshold=22263.333984375
node_335: feature_name=a-MoIgG_0.10	feature_id[16].value <= threshold=4199.8333740234375
Class: healthcare workers within 60 days after vaccination	
Rules_107	passed counts:2
node_0: feature_name=HuIgG_0.10	feature_id[9].value > threshold=19120.166015625
node_92: feature_name=a-HuIgG_0.03	feature_id[14].value <= threshold=19665.666015625
node_93: feature_name=SARS.CoV.2.Spike.RBD.His.HEK	feature_id[4].value > threshold=3.0166666507720947
node_105: feature_name=SARS.CoV.2.S1.mFcTag	feature_id[0].value > threshold=54010.166015625
node_305: feature_name=a-MoIgG_0.10	feature_id[16].value > threshold=2382.8333740234375
node_309: feature_name=SARS.CoV.S1.HisTag	feature_id[15].value <= threshold=23460.3330078125
node_310: feature_name=HuIgG_0.03	feature_id[8].value <= threshold=6941.33349609375
node_311: feature_name=a-HuIgG_0.10	feature_id[13].value <= threshold=32620.5
node_312: feature_name=SARS.CoV.2.Spike.RBD.His.Bac	feature_id[3].value > threshold=31266.0
node_320: feature_name=a-HuIgG_0.30	feature_id[11].value <= threshold=47574.66796875
node_321: feature_name=SARS.CoV.2.S1.RBD.mFc	feature_id[1].value > threshold=47171.166015625
node_323: feature_name=SARS.CoV.2.Spike.RBD.His.Bac	feature_id[3].value > threshold=32929.0
node_325: feature_name=SARS.CoV.2.Spike.RBD.His.Bac	feature_id[3].value <= threshold=37127.798828125
node_326: feature_name=SARS.CoV.2.S1+S2	feature_id[7].value > threshold=49134.966796875
node_328: feature_name=SARS.CoV.2.Spike.RBD.His.HEK	feature_id[4].value > threshold=35038.25

node_330: feature_name=SARS.CoV.2.S1+S2	feature_id[7].value > threshold=52119.966796875
Class: healthcare workers within 60 days after vaccination	
Rules_108	passed counts:2
node_0: feature_name=HuIgG_0.10	feature_id[9].value > threshold=19120.166015625
node_92: feature_name=a-HuIgG_0.03	feature_id[14].value <= threshold=19665.666015625
node_93: feature_name=SARS.CoV.2.Spike.RBD.His.HEK	feature_id[4].value > threshold=3.0166666507720947
node_105: feature_name=SARS.CoV.2.S1.mFcTag	feature_id[0].value <= threshold=54010.166015625
node_106: feature_name=SARS.CoV.2.S1+S2	feature_id[7].value > threshold=48317.708984375
node_228: feature_name=a-MoIgG_0.10	feature_id[16].value > threshold=1652.5
node_230: feature_name=HuIgG_0.10	feature_id[9].value > threshold=30107.666015625
Class: healthcare workers between 60 and 180 days after vaccination	
Rules_109	passed counts:2
node_0: feature_name=HuIgG_0.10	feature_id[9].value > threshold=19120.166015625
node_92: feature_name=a-HuIgG_0.03	feature_id[14].value <= threshold=19665.666015625
node_93: feature_name=SARS.CoV.2.Spike.RBD.His.HEK	feature_id[4].value > threshold=3.0166666507720947
node_105: feature_name=SARS.CoV.2.S1.mFcTag	feature_id[0].value <= threshold=54010.166015625
node_106: feature_name=SARS.CoV.2.S1+S2	feature_id[7].value > threshold=48317.708984375
node_228: feature_name=a-MoIgG_0.10	feature_id[16].value > threshold=1652.5
node_230: feature_name=HuIgG_0.10	feature_id[9].value <= threshold=30107.666015625
node_231: feature_name=HuIgG_0.03	feature_id[8].value > threshold=5533.83349609375
node_247: feature_name=HuIgG_0.10	feature_id[9].value > threshold=24231.0
node_261: feature_name=SARS.CoV.S1.RBD.HisTag	feature_id[10].value > threshold=24179.0419921875
node_293: feature_name=SARS.CoV.2.S1.HisTag	feature_id[2].value <= threshold=54233.25

node_294: feature_name=SARS.CoV.2.S1.mFcTag	feature_id[0].value > threshold=50885.83203125
node_298: feature_name=a-HuIgG_0.30	feature_id[11].value <= threshold=40480.16796875
node_299: feature_name=SARS.CoV.2.S1+S2	feature_id[7].value <= threshold=52487.333984375
Class: healthcare workers within 60 days after vaccination	
Rules_110	passed counts:2
node_0: feature_name=HuIgG_0.10	feature_id[9].value > threshold=19120.166015625
node_92: feature_name=a-HuIgG_0.03	feature_id[14].value <= threshold=19665.666015625
node_93: feature_name=SARS.CoV.2.Spike.RBD.His.HEK	feature_id[4].value > threshold=3.0166666507720947
node_105: feature_name=SARS.CoV.2.S1.mFcTag	feature_id[0].value <= threshold=54010.166015625
node_106: feature_name=SARS.CoV.2.S1+S2	feature_id[7].value > threshold=48317.708984375
node_228: feature_name=a-MoIgG_0.10	feature_id[16].value > threshold=1652.5
node_230: feature_name=HuIgG_0.10	feature_id[9].value <= threshold=30107.666015625
node_231: feature_name=HuIgG_0.03	feature_id[8].value > threshold=5533.83349609375
node_247: feature_name=HuIgG_0.10	feature_id[9].value > threshold=24231.0
node_261: feature_name=SARS.CoV.S1.RBD.HisTag	feature_id[10].value <= threshold=24179.0419921875
node_262: feature_name=HuIgG_0.10	feature_id[9].value > threshold=25555.333984375
node_266: feature_name=HuIgG_0.10	feature_id[9].value > threshold=25659.6669921875
node_270: feature_name=SARS.CoV.S1.HisTag	feature_id[15].value > threshold=10000.5830078125
node_276: feature_name=SARS.CoV.S1.HisTag	feature_id[15].value <= threshold=13228.125
node_277: feature_name=SARS.CoV.2.Spike.RBD.His.Bac	feature_id[3].value <= threshold=33671.91796875
Class: healthcare workers within 60 days after vaccination	
Rules_111	passed counts:2
node_0: feature_name=HuIgG_0.10	feature_id[9].value > threshold=19120.166015625

node_92: feature_name=a-HuIgG_0.03	feature_id[14].value <= threshold=19665.666015625
node_93: feature_name=SARS.CoV.2.Spike.RBD.His.HEK	feature_id[4].value > threshold=3.0166666507720947
node_105: feature_name=SARS.CoV.2.S1.mFcTag	feature_id[0].value <= threshold=54010.166015625
node_106: feature_name=SARS.CoV.2.S1+S2	feature_id[7].value > threshold=48317.708984375
node_228: feature_name=a-MoIgG_0.10	feature_id[16].value > threshold=1652.5
node_230: feature_name=HuIgG_0.10	feature_id[9].value <= threshold=30107.666015625
node_231: feature_name=HuIgG_0.03	feature_id[8].value > threshold=5533.83349609375
node_247: feature_name=HuIgG_0.10	feature_id[9].value <= threshold=24231.0
node_248: feature_name=SARS.CoV.2.S1.HisTag	feature_id[2].value > threshold=43585.92578125
node_256: feature_name=SARS.CoV.2.S1+S2	feature_id[7].value > threshold=52592.591796875
Class: healthcare workers between 60 and 180 days after vaccination	
Rules_112	passed counts:2
node_0: feature_name=HuIgG_0.10	feature_id[9].value > threshold=19120.166015625
node_92: feature_name=a-HuIgG_0.03	feature_id[14].value <= threshold=19665.666015625
node_93: feature_name=SARS.CoV.2.Spike.RBD.His.HEK	feature_id[4].value > threshold=3.0166666507720947
node_105: feature_name=SARS.CoV.2.S1.mFcTag	feature_id[0].value <= threshold=54010.166015625
node_106: feature_name=SARS.CoV.2.S1+S2	feature_id[7].value > threshold=48317.708984375
node_228: feature_name=a-MoIgG_0.10	feature_id[16].value > threshold=1652.5
node_230: feature_name=HuIgG_0.10	feature_id[9].value <= threshold=30107.666015625
node_231: feature_name=HuIgG_0.03	feature_id[8].value > threshold=5533.83349609375
node_247: feature_name=HuIgG_0.10	feature_id[9].value <= threshold=24231.0
node_248: feature_name=SARS.CoV.2.S1.HisTag	feature_id[2].value <= threshold=43585.92578125
node_249: feature_name=HuIgG_0.10	feature_id[9].value <= threshold=23668.3330078125
node_250: feature_name=SARS.CoV.2.S1+S2	feature_id[7].value > threshold=49682.4921875

node_252: feature_name=a-HuIgG_0.30	feature_id[11].value <= threshold=39201.833984375
Class: healthcare workers between 60 and 180 days after vaccination	
Rules_113	passed counts:2
node_0: feature_name=HuIgG_0.10	feature_id[9].value > threshold=19120.166015625
node_92: feature_name=a-HuIgG_0.03	feature_id[14].value <= threshold=19665.666015625
node_93: feature_name=SARS.CoV.2.Spike.RBD.His.HEK	feature_id[4].value > threshold=3.0166666507720947
node_105: feature_name=SARS.CoV.2.S1.mFcTag	feature_id[0].value <= threshold=54010.166015625
node_106: feature_name=SARS.CoV.2.S1+S2	feature_id[7].value > threshold=48317.708984375
node_228: feature_name=a-MoIgG_0.10	feature_id[16].value <= threshold=1652.5
Class: healthcare workers over 180 days after vaccination	
Rules_114	passed counts:2
node_0: feature_name=HuIgG_0.10	feature_id[9].value > threshold=19120.166015625
node_92: feature_name=a-HuIgG_0.03	feature_id[14].value <= threshold=19665.666015625
node_93: feature_name=SARS.CoV.2.Spike.RBD.His.HEK	feature_id[4].value > threshold=3.0166666507720947
node_105: feature_name=SARS.CoV.2.S1.mFcTag	feature_id[0].value <= threshold=54010.166015625
node_106: feature_name=SARS.CoV.2.S1+S2	feature_id[7].value <= threshold=48317.708984375
node_107: feature_name=a-HuIgG_0.03	feature_id[14].value > threshold=12099.83349609375
node_155: feature_name=SARS.CoV.2.Spike.RBD.His.Bac	feature_id[3].value > threshold=18979.9912109375
node_167: feature_name=a-MoIgG_0.10	feature_id[16].value > threshold=4979.5
node_201: feature_name=HuIgG_0.10	feature_id[9].value > threshold=26006.0
node_225: feature_name=SARS.CoV.2.Spike.RBD.His.HEK	feature_id[4].value <= threshold=26047.1416015625
Class: healthcare workers between 60 and 180 days after vaccination	

Rules_115	passed counts:2
node_0: feature_name=HuIgG_0.10	feature_id[9].value > threshold=19120.166015625
node_92: feature_name=a-HuIgG_0.03	feature_id[14].value <= threshold=19665.666015625
node_93: feature_name=SARS.CoV.2.Spike.RBD.His.HEK	feature_id[4].value > threshold=3.0166666507720947
node_105: feature_name=SARS.CoV.2.S1.mFcTag	feature_id[0].value <= threshold=54010.166015625
node_106: feature_name=SARS.CoV.2.S1+S2	feature_id[7].value <= threshold=48317.708984375
node_107: feature_name=a-HuIgG_0.03	feature_id[14].value > threshold=12099.83349609375
node_155: feature_name=SARS.CoV.2.Spike.RBD.His.Bac	feature_id[3].value > threshold=18979.9912109375
node_167: feature_name=a-MoIgG_0.10	feature_id[16].value > threshold=4979.5
node_201: feature_name=HuIgG_0.10	feature_id[9].value <= threshold=26006.0
node_202: feature_name=HuIgG_0.03	feature_id[8].value <= threshold=6080.0
node_203: feature_name=SARS.CoV.2.S1.HisTag	feature_id[2].value <= threshold=38575.359375
node_204: feature_name=SARS.CoV.2.Spike.RBD.His.HEK	feature_id[4].value > threshold=21567.4501953125
node_208: feature_name=HuIgG_0.03	feature_id[8].value <= threshold=4924.333251953125
node_209: feature_name=SARS.CoV.2.Spike.RBD.His.Bac	feature_id[3].value <= threshold=31771.125
Class: healthcare workers between 60 and 180 days after vaccination	
Rules_116	passed counts:2
node_0: feature_name=HuIgG_0.10	feature_id[9].value > threshold=19120.166015625
node_92: feature_name=a-HuIgG_0.03	feature_id[14].value <= threshold=19665.666015625
node_93: feature_name=SARS.CoV.2.Spike.RBD.His.HEK	feature_id[4].value > threshold=3.0166666507720947
node_105: feature_name=SARS.CoV.2.S1.mFcTag	feature_id[0].value <= threshold=54010.166015625
node_106: feature_name=SARS.CoV.2.S1+S2	feature_id[7].value <= threshold=48317.708984375
node_107: feature_name=a-HuIgG_0.03	feature_id[14].value > threshold=12099.83349609375
node_155: feature_name=SARS.CoV.2.Spike.RBD.His.Bac	feature_id[3].value > threshold=18979.9912109375

node_167: feature_name=a-MoIgG_0.10	feature_id[16].value > threshold=4979.5
node_201: feature_name=HuIgG_0.10	feature_id[9].value <= threshold=26006.0
node_202: feature_name=HuIgG_0.03	feature_id[8].value <= threshold=6080.0
node_203: feature_name=SARS.CoV.2.S1.HisTag	feature_id[2].value <= threshold=38575.359375
node_204: feature_name=SARS.CoV.2.Spike.RBD.His.HEK	feature_id[4].value <= threshold=21567.4501953125
node_205: feature_name=HuIgG_0.10	feature_id[9].value > threshold=23045.6669921875
Class: healthcare workers between 60 and 180 days after vaccination	
Rules_117	passed counts:2
node_0: feature_name=HuIgG_0.10	feature_id[9].value > threshold=19120.166015625
node_92: feature_name=a-HuIgG_0.03	feature_id[14].value <= threshold=19665.666015625
node_93: feature_name=SARS.CoV.2.Spike.RBD.His.HEK	feature_id[4].value > threshold=3.0166666507720947
node_105: feature_name=SARS.CoV.2.S1.mFcTag	feature_id[0].value <= threshold=54010.166015625
node_106: feature_name=SARS.CoV.2.S1+S2	feature_id[7].value <= threshold=48317.708984375
node_107: feature_name=a-HuIgG_0.03	feature_id[14].value > threshold=12099.83349609375
node_155: feature_name=SARS.CoV.2.Spike.RBD.His.Bac	feature_id[3].value > threshold=18979.9912109375
node_167: feature_name=a-MoIgG_0.10	feature_id[16].value <= threshold=4979.5
node_168: feature_name=SARS.CoV.2.S1+S2	feature_id[7].value > threshold=36298.75
node_172: feature_name=SARS.CoV.2.S1.RBD.mFc	feature_id[1].value > threshold=40153.283203125
node_182: feature_name=HuIgG_0.10	feature_id[9].value > threshold=21919.833984375
node_186: feature_name=a-HuIgG_0.10	feature_id[13].value > threshold=28919.0
node_192: feature_name=a-HuIgG_0.10	feature_id[13].value > threshold=31482.6669921875
node_198: feature_name=SARS.CoV.2.S1.mFcTag	feature_id[0].value <= threshold=50108.16796875
Class: healthcare workers between 60 and 180 days after vaccination	

Rules_118	passed counts:2
node_0: feature_name=HuIgG_0.10	feature_id[9].value > threshold=19120.166015625
node_92: feature_name=a-HuIgG_0.03	feature_id[14].value <= threshold=19665.666015625
node_93: feature_name=SARS.CoV.2.Spike.RBD.His.HEK	feature_id[4].value > threshold=3.0166666507720947
node_105: feature_name=SARS.CoV.2.S1.mFcTag	feature_id[0].value <= threshold=54010.166015625
node_106: feature_name=SARS.CoV.2.S1+S2	feature_id[7].value <= threshold=48317.708984375
node_107: feature_name=a-HuIgG_0.03	feature_id[14].value > threshold=12099.83349609375
node_155: feature_name=SARS.CoV.2.Spike.RBD.His.Bac	feature_id[3].value > threshold=18979.9912109375
node_167: feature_name=a-MoIgG_0.10	feature_id[16].value <= threshold=4979.5
node_168: feature_name=SARS.CoV.2.S1+S2	feature_id[7].value > threshold=36298.75
node_172: feature_name=SARS.CoV.2.S1.RBD.mFc	feature_id[1].value > threshold=40153.283203125
node_182: feature_name=HuIgG_0.10	feature_id[9].value > threshold=21919.833984375
node_186: feature_name=a-HuIgG_0.10	feature_id[13].value <= threshold=28919.0
node_187: feature_name=SARS.CoV.S1.HisTag	feature_id[15].value <= threshold=4488.5833740234375
Class: healthcare workers between 60 and 180 days after vaccination	
Rules_119	passed counts:2
node_0: feature_name=HuIgG_0.10	feature_id[9].value > threshold=19120.166015625
node_92: feature_name=a-HuIgG_0.03	feature_id[14].value <= threshold=19665.666015625
node_93: feature_name=SARS.CoV.2.Spike.RBD.His.HEK	feature_id[4].value > threshold=3.0166666507720947
node_105: feature_name=SARS.CoV.2.S1.mFcTag	feature_id[0].value <= threshold=54010.166015625
node_106: feature_name=SARS.CoV.2.S1+S2	feature_id[7].value <= threshold=48317.708984375
node_107: feature_name=a-HuIgG_0.03	feature_id[14].value > threshold=12099.83349609375
node_155: feature_name=SARS.CoV.2.Spike.RBD.His.Bac	feature_id[3].value > threshold=18979.9912109375
node_167: feature_name=a-MoIgG_0.10	feature_id[16].value <= threshold=4979.5

node_168: feature_name=SARS.CoV.2.S1+S2	feature_id[7].value > threshold=36298.75
node_172: feature_name=SARS.CoV.2.S1.RBD.mFc	feature_id[1].value <= threshold=40153.283203125
node_173: feature_name=SARS.CoV.2.S1.HisTag	feature_id[2].value > threshold=25186.375
node_175: feature_name=a-HuIgG_0.10	feature_id[13].value > threshold=27831.166015625
node_177: feature_name=SARS.CoV.2.S1	feature_id[6].value <= threshold=29819.333984375
node_178: feature_name=SARS.CoV.2.S1.HisTag	feature_id[2].value <= threshold=28188.974609375
Class: healthcare workers between 60 and 180 days after vaccination	
Rules_120	passed counts:2
node_0: feature_name=HuIgG_0.10	feature_id[9].value > threshold=19120.166015625
node_92: feature_name=a-HuIgG_0.03	feature_id[14].value <= threshold=19665.666015625
node_93: feature_name=SARS.CoV.2.Spike.RBD.His.HEK	feature_id[4].value > threshold=3.0166666507720947
node_105: feature_name=SARS.CoV.2.S1.mFcTag	feature_id[0].value <= threshold=54010.166015625
node_106: feature_name=SARS.CoV.2.S1+S2	feature_id[7].value <= threshold=48317.708984375
node_107: feature_name=a-HuIgG_0.03	feature_id[14].value > threshold=12099.83349609375
node_155: feature_name=SARS.CoV.2.Spike.RBD.His.Bac	feature_id[3].value > threshold=18979.9912109375
node_167: feature_name=a-MoIgG_0.10	feature_id[16].value <= threshold=4979.5
node_168: feature_name=SARS.CoV.2.S1+S2	feature_id[7].value <= threshold=36298.75
node_169: feature_name=SARS.CoV.S1.RBD.HisTag	feature_id[10].value <= threshold=6739.833251953125
Class: healthcare workers between 60 and 180 days after vaccination	
Rules_121	passed counts:2
node_0: feature_name=HuIgG_0.10	feature_id[9].value > threshold=19120.166015625
node_92: feature_name=a-HuIgG_0.03	feature_id[14].value <= threshold=19665.666015625
node_93: feature_name=SARS.CoV.2.Spike.RBD.His.HEK	feature_id[4].value > threshold=3.0166666507720947

node_105: feature_name=SARS.CoV.2.S1.mFcTag	feature_id[0].value <= threshold=54010.166015625
node_106: feature_name=SARS.CoV.2.S1+S2	feature_id[7].value <= threshold=48317.708984375
node_107: feature_name=a-HuIgG_0.03	feature_id[14].value > threshold=12099.83349609375
node_155: feature_name=SARS.CoV.2.Spike.RBD.His.Bac	feature_id[3].value <= threshold=18979.9912109375
node_156: feature_name=a-MoIgG_0.10	feature_id[16].value <= threshold=1739.1666259765625
node_157: feature_name=SARS.CoV.S1.RBD.HisTag	feature_id[10].value > threshold=1304.0555419921875
Class: healthcare workers over 180 days after vaccination	
Rules_122	passed counts:2
node_0: feature_name=HuIgG_0.10	feature_id[9].value > threshold=19120.166015625
node_92: feature_name=a-HuIgG_0.03	feature_id[14].value <= threshold=19665.666015625
node_93: feature_name=SARS.CoV.2.Spike.RBD.His.HEK	feature_id[4].value > threshold=3.0166666507720947
node_105: feature_name=SARS.CoV.2.S1.mFcTag	feature_id[0].value <= threshold=54010.166015625
node_106: feature_name=SARS.CoV.2.S1+S2	feature_id[7].value <= threshold=48317.708984375
node_107: feature_name=a-HuIgG_0.03	feature_id[14].value <= threshold=12099.83349609375
node_108: feature_name=SARS.CoV.2.Spike.RBD.His.Bac	feature_id[3].value > threshold=23841.4169921875
node_118: feature_name=SARS.CoV.2.Spike.RBD.rFc	feature_id[5].value > threshold=34554.69140625
node_120: feature_name=SARS.CoV.2.S1	feature_id[6].value > threshold=36508.716796875
node_142: feature_name=HuIgG_0.30	feature_id[12].value > threshold=40622.83203125
node_150: feature_name=SARS.CoV.S1.RBD.HisTag	feature_id[10].value <= threshold=9578.58349609375
Class: healthcare workers within 60 days after vaccination	
Rules_123	passed counts:2
node_0: feature_name=HuIgG_0.10	feature_id[9].value > threshold=19120.166015625
node_92: feature_name=a-HuIgG_0.03	feature_id[14].value <= threshold=19665.666015625

node_93: feature_name=SARS.CoV.2.Spike.RBD.His.HEK	feature_id[4].value > threshold=3.0166666507720947
node_105: feature_name=SARS.CoV.2.S1.mFcTag	feature_id[0].value <= threshold=54010.166015625
node_106: feature_name=SARS.CoV.2.S1+S2	feature_id[7].value <= threshold=48317.708984375
node_107: feature_name=a-HuIgG_0.03	feature_id[14].value <= threshold=12099.83349609375
node_108: feature_name=SARS.CoV.2.Spike.RBD.His.Bac	feature_id[3].value > threshold=23841.4169921875
node_118: feature_name=SARS.CoV.2.Spike.RBD.rFc	feature_id[5].value > threshold=34554.69140625
node_120: feature_name=SARS.CoV.2.S1	feature_id[6].value <= threshold=36508.716796875
node_121: feature_name=HuIgG_0.10	feature_id[9].value <= threshold=24763.3330078125
node_122: feature_name=SARS.CoV.2.Spike.RBD.rFc	feature_id[5].value <= threshold=42966.541015625
node_123: feature_name=SARS.CoV.2.S1+S2	feature_id[7].value <= threshold=43434.54296875
node_124: feature_name=SARS.CoV.2.S1+S2	feature_id[7].value <= threshold=42800.94140625
node_125: feature_name=SARS.CoV.2.S1.mFcTag	feature_id[0].value > threshold=47427.1328125
node_129: feature_name=a-MoIgG_0.10	feature_id[16].value > threshold=5634.0
node_131: feature_name=SARS.CoV.2.S1.RBD.mFc	feature_id[1].value > threshold=39372.166015625
Class: healthcare workers between 60 and 180 days after vaccination	
Rules_124	passed counts:2
node_0: feature_name=HuIgG_0.10	feature_id[9].value <= threshold=19120.166015625
node_1: feature_name=SARS.CoV.2.S1.RBD.mFc	feature_id[1].value > threshold=3773.8334350585938
node_9: feature_name=a-HuIgG_0.30	feature_id[11].value > threshold=33726.833984375
node_47: feature_name=SARS.CoV.2.S1.mFcTag	feature_id[0].value > threshold=43185.68359375
node_57: feature_name=a-HuIgG_0.03	feature_id[14].value > threshold=10858.0
node_71: feature_name=HuIgG_0.03	feature_id[8].value <= threshold=3796.0
node_72: feature_name=SARS.CoV.2.S1.mFcTag	feature_id[0].value <= threshold=55206.517578125
node_73: feature_name=SARS.CoV.2.S1.mFcTag	feature_id[0].value > threshold=46065.833984375

node_75: feature_name=SARS.CoV.2.S1	feature_id[6].value > threshold=35627.0
node_77: feature_name=SARS.CoV.S1.HisTag	feature_id[15].value <= threshold=21926.9169921875
node_78: feature_name=SARS.CoV.2.S1+S2	feature_id[7].value <= threshold=40211.734375
Class: healthcare workers over 180 days after vaccination	
Rules_125	passed counts:2
node_0: feature_name=HuIgG_0.10	feature_id[9].value <= threshold=19120.166015625
node_1: feature_name=SARS.CoV.2.S1.RBD.mFc	feature_id[1].value > threshold=3773.8334350585938
node_9: feature_name=a-HuIgG_0.30	feature_id[11].value > threshold=33726.833984375
node_47: feature_name=SARS.CoV.2.S1.mFcTag	feature_id[0].value > threshold=43185.68359375
node_57: feature_name=a-HuIgG_0.03	feature_id[14].value <= threshold=10858.0
node_58: feature_name=HuIgG_0.30	feature_id[12].value <= threshold=36767.33203125
node_59: feature_name=a-HuIgG_0.10	feature_id[13].value <= threshold=21868.0
Class: healthcare workers over 180 days after vaccination	
Rules_126	passed counts:2
node_0: feature_name=HuIgG_0.10	feature_id[9].value <= threshold=19120.166015625
node_1: feature_name=SARS.CoV.2.S1.RBD.mFc	feature_id[1].value > threshold=3773.8334350585938
node_9: feature_name=a-HuIgG_0.30	feature_id[11].value <= threshold=33726.833984375
node_10: feature_name=HuIgG_0.03	feature_id[8].value > threshold=1023.1666564941406
node_20: feature_name=SARS.CoV.S1.HisTag	feature_id[15].value <= threshold=400.3000030517578
node_21: feature_name=SARS.CoV.2.Spike.RBD.His.HEK	feature_id[4].value > threshold=2291.9304809570312
Class: unvaccinated healthcare workers	
Rules_127	passed counts:1

node_0: feature_name=HuIgG_0.10	feature_id[9].value > threshold=19120.166015625
node_92: feature_name=a-HuIgG_0.03	feature_id[14].value > threshold=19665.666015625
node_382: feature_name=a-MoIgG_0.10	feature_id[16].value <= threshold=20281.6669921875
node_383: feature_name=a-HuIgG_0.10	feature_id[13].value > threshold=33917.66796875
node_391: feature_name=HuIgG_0.10	feature_id[9].value > threshold=25959.0
node_393: feature_name=HuIgG_0.30	feature_id[12].value <= threshold=52448.166015625
node_394: feature_name=a-HuIgG_0.10	feature_id[13].value <= threshold=38348.5
node_395: feature_name=a-HuIgG_0.30	feature_id[11].value <= threshold=53098.833984375
node_396: feature_name=SARS.CoV.S1.RBD.HisTag	feature_id[10].value > threshold=36882.4677734375
Class: healthcare workers between 60 and 180 days after vaccination	
Rules_128	passed counts:1
node_0: feature_name=HuIgG_0.10	feature_id[9].value > threshold=19120.166015625
node_92: feature_name=a-HuIgG_0.03	feature_id[14].value > threshold=19665.666015625
node_382: feature_name=a-MoIgG_0.10	feature_id[16].value <= threshold=20281.6669921875
node_383: feature_name=a-HuIgG_0.10	feature_id[13].value > threshold=33917.66796875
node_391: feature_name=HuIgG_0.10	feature_id[9].value > threshold=25959.0
node_393: feature_name=HuIgG_0.30	feature_id[12].value <= threshold=52448.166015625
node_394: feature_name=a-HuIgG_0.10	feature_id[13].value <= threshold=38348.5
node_395: feature_name=a-HuIgG_0.30	feature_id[11].value <= threshold=53098.833984375
node_396: feature_name=SARS.CoV.S1.RBD.HisTag	feature_id[10].value <= threshold=36882.4677734375
Class: healthcare workers over 180 days after vaccination	
Rules_129	passed counts:1
node_0: feature_name=HuIgG_0.10	feature_id[9].value > threshold=19120.166015625

node_92: feature_name=a-HuIgG_0.03	feature_id[14].value > threshold=19665.666015625
node_382: feature_name=a-MoIgG_0.10	feature_id[16].value <= threshold=20281.6669921875
node_383: feature_name=a-HuIgG_0.10	feature_id[13].value <= threshold=33917.66796875
node_384: feature_name=SARS.CoV.2.S1.mFcTag	feature_id[0].value > threshold=52180.75
node_388: feature_name=SARS.CoV.S1.RBD.HisTag	feature_id[10].value > threshold=52228.166015625
Class: healthcare workers over 180 days after vaccination	
Rules_130	passed counts:1
node_0: feature_name=HuIgG_0.10	feature_id[9].value > threshold=19120.166015625
node_92: feature_name=a-HuIgG_0.03	feature_id[14].value > threshold=19665.666015625
node_382: feature_name=a-MoIgG_0.10	feature_id[16].value <= threshold=20281.6669921875
node_383: feature_name=a-HuIgG_0.10	feature_id[13].value <= threshold=33917.66796875
node_384: feature_name=SARS.CoV.2.S1.mFcTag	feature_id[0].value <= threshold=52180.75
node_385: feature_name=SARS.CoV.S1.HisTag	feature_id[15].value <= threshold=6022.0166015625
Class: healthcare workers between 60 and 180 days after vaccination	
Rules_131	passed counts:1
node_0: feature_name=HuIgG_0.10	feature_id[9].value > threshold=19120.166015625
node_92: feature_name=a-HuIgG_0.03	feature_id[14].value <= threshold=19665.666015625
node_93: feature_name=SARS.CoV.2.Spike.RBD.His.HEK	feature_id[4].value > threshold=3.0166666507720947
node_105: feature_name=SARS.CoV.2.S1.mFcTag	feature_id[0].value > threshold=54010.166015625
node_305: feature_name=a-MoIgG_0.10	feature_id[16].value > threshold=2382.8333740234375
node_309: feature_name=SARS.CoV.S1.HisTag	feature_id[15].value > threshold=23460.3330078125
node_365: feature_name=a-HuIgG_0.03	feature_id[14].value > threshold=11128.66650390625
node_367: feature_name=SARS.CoV.2.S1.mFcTag	feature_id[0].value > threshold=54294.0

node_371: feature_name=SARS.CoV.2.S1.mFcTag	feature_id[0].value > threshold=55620.833984375
Class: healthcare workers between 60 and 180 days after vaccination	
Rules_132	passed counts:1
node_0: feature_name=HuIgG_0.10	feature_id[9].value > threshold=19120.166015625
node_92: feature_name=a-HuIgG_0.03	feature_id[14].value <= threshold=19665.666015625
node_93: feature_name=SARS.CoV.2.Spike.RBD.His.HEK	feature_id[4].value > threshold=3.0166666507720947
node_105: feature_name=SARS.CoV.2.S1.mFcTag	feature_id[0].value > threshold=54010.166015625
node_305: feature_name=a-MoIgG_0.10	feature_id[16].value > threshold=2382.8333740234375
node_309: feature_name=SARS.CoV.S1.HisTag	feature_id[15].value > threshold=23460.3330078125
node_365: feature_name=a-HuIgG_0.03	feature_id[14].value > threshold=11128.66650390625
node_367: feature_name=SARS.CoV.2.S1.mFcTag	feature_id[0].value > threshold=54294.0
node_371: feature_name=SARS.CoV.2.S1.mFcTag	feature_id[0].value <= threshold=55620.833984375
node_372: feature_name=a-HuIgG_0.10	feature_id[13].value > threshold=28320.6669921875
node_374: feature_name=SARS.CoV.2.S1	feature_id[6].value <= threshold=47335.5
Class: healthcare workers between 60 and 180 days after vaccination	
Rules_133	passed counts:1
node_0: feature_name=HuIgG_0.10	feature_id[9].value > threshold=19120.166015625
node_92: feature_name=a-HuIgG_0.03	feature_id[14].value <= threshold=19665.666015625
node_93: feature_name=SARS.CoV.2.Spike.RBD.His.HEK	feature_id[4].value > threshold=3.0166666507720947
node_105: feature_name=SARS.CoV.2.S1.mFcTag	feature_id[0].value > threshold=54010.166015625
node_305: feature_name=a-MoIgG_0.10	feature_id[16].value > threshold=2382.8333740234375
node_309: feature_name=SARS.CoV.S1.HisTag	feature_id[15].value > threshold=23460.3330078125
node_365: feature_name=a-HuIgG_0.03	feature_id[14].value > threshold=11128.66650390625

node_367: feature_name=SARS.CoV.2.S1.mFcTag	feature_id[0].value > threshold=54294.0
node_371: feature_name=SARS.CoV.2.S1.mFcTag	feature_id[0].value <= threshold=55620.833984375
node_372: feature_name=a-HuIgG_0.10	feature_id[13].value <= threshold=28320.6669921875
Class: healthcare workers between 60 and 180 days after vaccination	
Rules_134	passed counts:1
node_0: feature_name=HuIgG_0.10	feature_id[9].value > threshold=19120.166015625
node_92: feature_name=a-HuIgG_0.03	feature_id[14].value <= threshold=19665.666015625
node_93: feature_name=SARS.CoV.2.Spike.RBD.His.HEK	feature_id[4].value > threshold=3.0166666507720947
node_105: feature_name=SARS.CoV.2.S1.mFcTag	feature_id[0].value > threshold=54010.166015625
node_305: feature_name=a-MoIgG_0.10	feature_id[16].value > threshold=2382.8333740234375
node_309: feature_name=SARS.CoV.S1.HisTag	feature_id[15].value <= threshold=23460.3330078125
node_310: feature_name=HuIgG_0.03	feature_id[8].value > threshold=6941.33349609375
node_362: feature_name=SARS.CoV.2.Spike.RBD.His.Bac	feature_id[3].value > threshold=43627.66796875
Class: unvaccinated healthcare workers	
Rules_135	passed counts:1
node_0: feature_name=HuIgG_0.10	feature_id[9].value > threshold=19120.166015625
node_92: feature_name=a-HuIgG_0.03	feature_id[14].value <= threshold=19665.666015625
node_93: feature_name=SARS.CoV.2.Spike.RBD.His.HEK	feature_id[4].value > threshold=3.0166666507720947
node_105: feature_name=SARS.CoV.2.S1.mFcTag	feature_id[0].value > threshold=54010.166015625
node_305: feature_name=a-MoIgG_0.10	feature_id[16].value > threshold=2382.8333740234375
node_309: feature_name=SARS.CoV.S1.HisTag	feature_id[15].value <= threshold=23460.3330078125
node_310: feature_name=HuIgG_0.03	feature_id[8].value <= threshold=6941.33349609375
node_311: feature_name=a-HuIgG_0.10	feature_id[13].value > threshold=32620.5

node_351: feature_name=SARS.CoV.S1.RBD.HisTag	feature_id[10].value > threshold=13834.0
node_359: feature_name=HuIgG_0.30	feature_id[12].value <= threshold=39323.666015625
Class: unvaccinated healthcare workers	
Rules_136	passed counts:1
node_0: feature_name=HuIgG_0.10	feature_id[9].value > threshold=19120.166015625
node_92: feature_name=a-HuIgG_0.03	feature_id[14].value <= threshold=19665.666015625
node_93: feature_name=SARS.CoV.2.Spike.RBD.His.HEK	feature_id[4].value > threshold=3.0166666507720947
node_105: feature_name=SARS.CoV.2.S1.mFcTag	feature_id[0].value > threshold=54010.166015625
node_305: feature_name=a-MoIgG_0.10	feature_id[16].value > threshold=2382.8333740234375
node_309: feature_name=SARS.CoV.S1.HisTag	feature_id[15].value <= threshold=23460.3330078125
node_310: feature_name=HuIgG_0.03	feature_id[8].value <= threshold=6941.33349609375
node_311: feature_name=a-HuIgG_0.10	feature_id[13].value > threshold=32620.5
node_351: feature_name=SARS.CoV.S1.RBD.HisTag	feature_id[10].value <= threshold=13834.0
node_352: feature_name=SARS.CoV.2.S1	feature_id[6].value <= threshold=47365.833984375
node_353: feature_name=SARS.CoV.2.Spike.RBD.His.HEK	feature_id[4].value <= threshold=29192.4169921875
node_354: feature_name=a-MoIgG_0.10	feature_id[16].value <= threshold=3888.6666259765625
Class: healthcare workers within 60 days after vaccination	
Rules_137	passed counts:1
node_0: feature_name=HuIgG_0.10	feature_id[9].value > threshold=19120.166015625
node_92: feature_name=a-HuIgG_0.03	feature_id[14].value <= threshold=19665.666015625
node_93: feature_name=SARS.CoV.2.Spike.RBD.His.HEK	feature_id[4].value > threshold=3.0166666507720947
node_105: feature_name=SARS.CoV.2.S1.mFcTag	feature_id[0].value > threshold=54010.166015625
node_305: feature_name=a-MoIgG_0.10	feature_id[16].value > threshold=2382.8333740234375

node_309: feature_name=SARS.CoV.S1.HisTag	feature_id[15].value <= threshold=23460.3330078125
node_310: feature_name=HuIgG_0.03	feature_id[8].value <= threshold=6941.33349609375
node_311: feature_name=a-HuIgG_0.10	feature_id[13].value <= threshold=32620.5
node_312: feature_name=SARS.CoV.2.Spike.RBD.His.Bac	feature_id[3].value > threshold=31266.0
node_320: feature_name=a-HuIgG_0.30	feature_id[11].value > threshold=47574.66796875
node_346: feature_name=a-HuIgG_0.10	feature_id[13].value > threshold=28994.833984375
node_348: feature_name=HuIgG_0.30	feature_id[12].value > threshold=47369.0
Class: healthcare workers within 60 days after vaccination	
Rules_138	passed counts:1
node_0: feature_name=HuIgG_0.10	feature_id[9].value > threshold=19120.166015625
node_92: feature_name=a-HuIgG_0.03	feature_id[14].value <= threshold=19665.666015625
node_93: feature_name=SARS.CoV.2.Spike.RBD.His.HEK	feature_id[4].value > threshold=3.0166666507720947
node_105: feature_name=SARS.CoV.2.S1.mFcTag	feature_id[0].value > threshold=54010.166015625
node_305: feature_name=a-MoIgG_0.10	feature_id[16].value > threshold=2382.8333740234375
node_309: feature_name=SARS.CoV.S1.HisTag	feature_id[15].value <= threshold=23460.3330078125
node_310: feature_name=HuIgG_0.03	feature_id[8].value <= threshold=6941.33349609375
node_311: feature_name=a-HuIgG_0.10	feature_id[13].value <= threshold=32620.5
node_312: feature_name=SARS.CoV.2.Spike.RBD.His.Bac	feature_id[3].value > threshold=31266.0
node_320: feature_name=a-HuIgG_0.30	feature_id[11].value > threshold=47574.66796875
node_346: feature_name=a-HuIgG_0.10	feature_id[13].value <= threshold=28994.833984375
Class: healthcare workers within 60 days after vaccination	
Rules_139	passed counts:1
node_0: feature_name=HuIgG_0.10	feature_id[9].value > threshold=19120.166015625

node_92: feature_name=a-HuIgG_0.03	feature_id[14].value <= threshold=19665.666015625
node_93: feature_name=SARS.CoV.2.Spike.RBD.His.HEK	feature_id[4].value > threshold=3.0166666507720947
node_105: feature_name=SARS.CoV.2.S1.mFcTag	feature_id[0].value > threshold=54010.166015625
node_305: feature_name=a-MoIgG_0.10	feature_id[16].value > threshold=2382.8333740234375
node_309: feature_name=SARS.CoV.S1.HisTag	feature_id[15].value <= threshold=23460.3330078125
node_310: feature_name=HuIgG_0.03	feature_id[8].value <= threshold=6941.33349609375
node_311: feature_name=a-HuIgG_0.10	feature_id[13].value <= threshold=32620.5
node_312: feature_name=SARS.CoV.2.Spike.RBD.His.Bac	feature_id[3].value > threshold=31266.0
node_320: feature_name=a-HuIgG_0.30	feature_id[11].value <= threshold=47574.66796875
node_321: feature_name=SARS.CoV.2.S1.RBD.mFc	feature_id[1].value > threshold=47171.166015625
node_323: feature_name=SARS.CoV.2.Spike.RBD.His.Bac	feature_id[3].value > threshold=32929.0
node_325: feature_name=SARS.CoV.2.Spike.RBD.His.Bac	feature_id[3].value > threshold=37127.798828125
node_333: feature_name=a-HuIgG_0.10	feature_id[13].value > threshold=30243.0
node_339: feature_name=a-HuIgG_0.10	feature_id[13].value > threshold=31594.5
node_343: feature_name=SARS.CoV.2.Spike.RBD.His.HEK	feature_id[4].value <= threshold=38606.166015625
Class: healthcare workers between 60 and 180 days after vaccination	
Rules_140	passed counts:1
node_0: feature_name=HuIgG_0.10	feature_id[9].value > threshold=19120.166015625
node_92: feature_name=a-HuIgG_0.03	feature_id[14].value <= threshold=19665.666015625
node_93: feature_name=SARS.CoV.2.Spike.RBD.His.HEK	feature_id[4].value > threshold=3.0166666507720947
node_105: feature_name=SARS.CoV.2.S1.mFcTag	feature_id[0].value > threshold=54010.166015625
node_305: feature_name=a-MoIgG_0.10	feature_id[16].value > threshold=2382.8333740234375
node_309: feature_name=SARS.CoV.S1.HisTag	feature_id[15].value <= threshold=23460.3330078125
node_310: feature_name=HuIgG_0.03	feature_id[8].value <= threshold=6941.33349609375

node_311: feature_name=a-HuIgG_0.10	feature_id[13].value <= threshold=32620.5
node_312: feature_name=SARS.CoV.2.Spike.RBD.His.Bac	feature_id[3].value > threshold=31266.0
node_320: feature_name=a-HuIgG_0.30	feature_id[11].value <= threshold=47574.66796875
node_321: feature_name=SARS.CoV.2.S1.RBD.mFc	feature_id[1].value > threshold=47171.166015625
node_323: feature_name=SARS.CoV.2.Spike.RBD.His.Bac	feature_id[3].value > threshold=32929.0
node_325: feature_name=SARS.CoV.2.Spike.RBD.His.Bac	feature_id[3].value > threshold=37127.798828125
node_333: feature_name=a-HuIgG_0.10	feature_id[13].value > threshold=30243.0
node_339: feature_name=a-HuIgG_0.10	feature_id[13].value <= threshold=31594.5
node_340: feature_name=SARS.CoV.2.S1.RBD.mFc	feature_id[1].value > threshold=53514.666015625
Class: healthcare workers within 60 days after vaccination	
Rules_141	passed counts:1
node_0: feature_name=HuIgG_0.10	feature_id[9].value > threshold=19120.166015625
node_92: feature_name=a-HuIgG_0.03	feature_id[14].value <= threshold=19665.666015625
node_93: feature_name=SARS.CoV.2.Spike.RBD.His.HEK	feature_id[4].value > threshold=3.0166666507720947
node_105: feature_name=SARS.CoV.2.S1.mFcTag	feature_id[0].value > threshold=54010.166015625
node_305: feature_name=a-MoIgG_0.10	feature_id[16].value > threshold=2382.8333740234375
node_309: feature_name=SARS.CoV.S1.HisTag	feature_id[15].value <= threshold=23460.3330078125
node_310: feature_name=HuIgG_0.03	feature_id[8].value <= threshold=6941.33349609375
node_311: feature_name=a-HuIgG_0.10	feature_id[13].value <= threshold=32620.5
node_312: feature_name=SARS.CoV.2.Spike.RBD.His.Bac	feature_id[3].value > threshold=31266.0
node_320: feature_name=a-HuIgG_0.30	feature_id[11].value <= threshold=47574.66796875
node_321: feature_name=SARS.CoV.2.S1.RBD.mFc	feature_id[1].value > threshold=47171.166015625
node_323: feature_name=SARS.CoV.2.Spike.RBD.His.Bac	feature_id[3].value > threshold=32929.0
node_325: feature_name=SARS.CoV.2.Spike.RBD.His.Bac	feature_id[3].value <= threshold=37127.798828125

node_326: feature_name=SARS.CoV.2.S1+S2	feature_id[7].value > threshold=49134.966796875
node_328: feature_name=SARS.CoV.2.Spike.RBD.His.HEK	feature_id[4].value > threshold=35038.25
node_330: feature_name=SARS.CoV.2.S1+S2	feature_id[7].value <= threshold=52119.966796875
Class: healthcare workers between 60 and 180 days after vaccination	
Rules_142	passed counts:1
node_0: feature_name=HuIgG_0.10	feature_id[9].value > threshold=19120.166015625
node_92: feature_name=a-HuIgG_0.03	feature_id[14].value <= threshold=19665.666015625
node_93: feature_name=SARS.CoV.2.Spike.RBD.His.HEK	feature_id[4].value > threshold=3.0166666507720947
node_105: feature_name=SARS.CoV.2.S1.mFcTag	feature_id[0].value > threshold=54010.166015625
node_305: feature_name=a-MoIgG_0.10	feature_id[16].value > threshold=2382.8333740234375
node_309: feature_name=SARS.CoV.S1.HisTag	feature_id[15].value <= threshold=23460.3330078125
node_310: feature_name=HuIgG_0.03	feature_id[8].value <= threshold=6941.33349609375
node_311: feature_name=a-HuIgG_0.10	feature_id[13].value <= threshold=32620.5
node_312: feature_name=SARS.CoV.2.Spike.RBD.His.Bac	feature_id[3].value > threshold=31266.0
node_320: feature_name=a-HuIgG_0.30	feature_id[11].value <= threshold=47574.66796875
node_321: feature_name=SARS.CoV.2.S1.RBD.mFc	feature_id[1].value > threshold=47171.166015625
node_323: feature_name=SARS.CoV.2.Spike.RBD.His.Bac	feature_id[3].value > threshold=32929.0
node_325: feature_name=SARS.CoV.2.Spike.RBD.His.Bac	feature_id[3].value <= threshold=37127.798828125
node_326: feature_name=SARS.CoV.2.S1+S2	feature_id[7].value <= threshold=49134.966796875
Class: healthcare workers between 60 and 180 days after vaccination	
Rules_143	passed counts:1
node_0: feature_name=HuIgG_0.10	feature_id[9].value > threshold=19120.166015625
node_92: feature_name=a-HuIgG_0.03	feature_id[14].value <= threshold=19665.666015625

node_93: feature_name=SARS.CoV.2.Spike.RBD.His.HEK	feature_id[4].value > threshold=3.0166666507720947
node_105: feature_name=SARS.CoV.2.S1.mFcTag	feature_id[0].value > threshold=54010.166015625
node_305: feature_name=a-MoIgG_0.10	feature_id[16].value > threshold=2382.8333740234375
node_309: feature_name=SARS.CoV.S1.HisTag	feature_id[15].value <= threshold=23460.3330078125
node_310: feature_name=HuIgG_0.03	feature_id[8].value <= threshold=6941.33349609375
node_311: feature_name=a-HuIgG_0.10	feature_id[13].value <= threshold=32620.5
node_312: feature_name=SARS.CoV.2.Spike.RBD.His.Bac	feature_id[3].value <= threshold=31266.0
node_313: feature_name=HuIgG_0.10	feature_id[9].value > threshold=22308.5
node_317: feature_name=SARS.CoV.2.Spike.RBD.rFc	feature_id[5].value > threshold=47206.232421875
Class: healthcare workers between 60 and 180 days after vaccination	
Rules_144	passed counts:1
node_0: feature_name=HuIgG_0.10	feature_id[9].value > threshold=19120.166015625
node_92: feature_name=a-HuIgG_0.03	feature_id[14].value <= threshold=19665.666015625
node_93: feature_name=SARS.CoV.2.Spike.RBD.His.HEK	feature_id[4].value > threshold=3.0166666507720947
node_105: feature_name=SARS.CoV.2.S1.mFcTag	feature_id[0].value > threshold=54010.166015625
node_305: feature_name=a-MoIgG_0.10	feature_id[16].value > threshold=2382.8333740234375
node_309: feature_name=SARS.CoV.S1.HisTag	feature_id[15].value <= threshold=23460.3330078125
node_310: feature_name=HuIgG_0.03	feature_id[8].value <= threshold=6941.33349609375
node_311: feature_name=a-HuIgG_0.10	feature_id[13].value <= threshold=32620.5
node_312: feature_name=SARS.CoV.2.Spike.RBD.His.Bac	feature_id[3].value <= threshold=31266.0
node_313: feature_name=HuIgG_0.10	feature_id[9].value <= threshold=22308.5
node_314: feature_name=a-MoIgG_0.10	feature_id[16].value > threshold=7511.833251953125
Class: healthcare workers within 60 days after vaccination	

Rules_145	passed counts:1
node_0: feature_name=HuIgG_0.10	feature_id[9].value > threshold=19120.166015625
node_92: feature_name=a-HuIgG_0.03	feature_id[14].value <= threshold=19665.666015625
node_93: feature_name=SARS.CoV.2.Spike.RBD.His.HEK	feature_id[4].value > threshold=3.0166666507720947
node_105: feature_name=SARS.CoV.2.S1.mFcTag	feature_id[0].value > threshold=54010.166015625
node_305: feature_name=a-MoIgG_0.10	feature_id[16].value <= threshold=2382.8333740234375
node_306: feature_name=SARS.CoV.2.S1	feature_id[6].value <= threshold=34987.400390625
Class: healthcare workers between 60 and 180 days after vaccination	
Rules_146	passed counts:1
node_0: feature_name=HuIgG_0.10	feature_id[9].value > threshold=19120.166015625
node_92: feature_name=a-HuIgG_0.03	feature_id[14].value <= threshold=19665.666015625
node_93: feature_name=SARS.CoV.2.Spike.RBD.His.HEK	feature_id[4].value > threshold=3.0166666507720947
node_105: feature_name=SARS.CoV.2.S1.mFcTag	feature_id[0].value <= threshold=54010.166015625
node_106: feature_name=SARS.CoV.2.S1+S2	feature_id[7].value > threshold=48317.708984375
node_228: feature_name=a-MoIgG_0.10	feature_id[16].value > threshold=1652.5
node_230: feature_name=HuIgG_0.10	feature_id[9].value <= threshold=30107.666015625
node_231: feature_name=HuIgG_0.03	feature_id[8].value > threshold=5533.83349609375
node_247: feature_name=HuIgG_0.10	feature_id[9].value > threshold=24231.0
node_261: feature_name=SARS.CoV.S1.RBD.HisTag	feature_id[10].value > threshold=24179.0419921875
node_293: feature_name=SARS.CoV.2.S1.HisTag	feature_id[2].value > threshold=54233.25
Class: healthcare workers between 60 and 180 days after vaccination	
Rules_147	passed counts:1
node_0: feature_name=HuIgG_0.10	feature_id[9].value > threshold=19120.166015625

node_92: feature_name=a-HuIgG_0.03	feature_id[14].value <= threshold=19665.666015625
node_93: feature_name=SARS.CoV.2.Spike.RBD.His.HEK	feature_id[4].value > threshold=3.0166666507720947
node_105: feature_name=SARS.CoV.2.S1.mFcTag	feature_id[0].value <= threshold=54010.166015625
node_106: feature_name=SARS.CoV.2.S1+S2	feature_id[7].value > threshold=48317.708984375
node_228: feature_name=a-MoIgG_0.10	feature_id[16].value > threshold=1652.5
node_230: feature_name=HuIgG_0.10	feature_id[9].value <= threshold=30107.666015625
node_231: feature_name=HuIgG_0.03	feature_id[8].value > threshold=5533.83349609375
node_247: feature_name=HuIgG_0.10	feature_id[9].value > threshold=24231.0
node_261: feature_name=SARS.CoV.S1.RBD.HisTag	feature_id[10].value > threshold=24179.0419921875
node_293: feature_name=SARS.CoV.2.S1.HisTag	feature_id[2].value <= threshold=54233.25
node_294: feature_name=SARS.CoV.2.S1.mFcTag	feature_id[0].value > threshold=50885.83203125
node_298: feature_name=a-HuIgG_0.30	feature_id[11].value <= threshold=40480.16796875
node_299: feature_name=SARS.CoV.2.S1+S2	feature_id[7].value > threshold=52487.333984375
Class: healthcare workers between 60 and 180 days after vaccination	
Rules_148	passed counts:1
node_0: feature_name=HuIgG_0.10	feature_id[9].value > threshold=19120.166015625
node_92: feature_name=a-HuIgG_0.03	feature_id[14].value <= threshold=19665.666015625
node_93: feature_name=SARS.CoV.2.Spike.RBD.His.HEK	feature_id[4].value > threshold=3.0166666507720947
node_105: feature_name=SARS.CoV.2.S1.mFcTag	feature_id[0].value <= threshold=54010.166015625
node_106: feature_name=SARS.CoV.2.S1+S2	feature_id[7].value > threshold=48317.708984375
node_228: feature_name=a-MoIgG_0.10	feature_id[16].value > threshold=1652.5
node_230: feature_name=HuIgG_0.10	feature_id[9].value <= threshold=30107.666015625
node_231: feature_name=HuIgG_0.03	feature_id[8].value > threshold=5533.83349609375
node_247: feature_name=HuIgG_0.10	feature_id[9].value > threshold=24231.0

node_261: feature_name=SARS.CoV.S1.RBD.HisTag	feature_id[10].value > threshold=24179.0419921875
node_293: feature_name=SARS.CoV.2.S1.HisTag	feature_id[2].value <= threshold=54233.25
node_294: feature_name=SARS.CoV.2.S1.mFcTag	feature_id[0].value <= threshold=50885.83203125
node_295: feature_name=SARS.CoV.2.S1	feature_id[6].value > threshold=50233.8828125
Class: healthcare workers over 180 days after vaccination	
Rules_149	passed counts:1
node_0: feature_name=HuIgG_0.10	feature_id[9].value > threshold=19120.166015625
node_92: feature_name=a-HuIgG_0.03	feature_id[14].value <= threshold=19665.666015625
node_93: feature_name=SARS.CoV.2.Spike.RBD.His.HEK	feature_id[4].value > threshold=3.0166666507720947
node_105: feature_name=SARS.CoV.2.S1.mFcTag	feature_id[0].value <= threshold=54010.166015625
node_106: feature_name=SARS.CoV.2.S1+S2	feature_id[7].value > threshold=48317.708984375
node_228: feature_name=a-MoIgG_0.10	feature_id[16].value > threshold=1652.5
node_230: feature_name=HuIgG_0.10	feature_id[9].value <= threshold=30107.666015625
node_231: feature_name=HuIgG_0.03	feature_id[8].value > threshold=5533.83349609375
node_247: feature_name=HuIgG_0.10	feature_id[9].value > threshold=24231.0
node_261: feature_name=SARS.CoV.S1.RBD.HisTag	feature_id[10].value > threshold=24179.0419921875
node_293: feature_name=SARS.CoV.2.S1.HisTag	feature_id[2].value <= threshold=54233.25
node_294: feature_name=SARS.CoV.2.S1.mFcTag	feature_id[0].value <= threshold=50885.83203125
node_295: feature_name=SARS.CoV.2.S1	feature_id[6].value <= threshold=50233.8828125
Class: healthcare workers within 60 days after vaccination	
Rules_150	passed counts:1
node_0: feature_name=HuIgG_0.10	feature_id[9].value > threshold=19120.166015625
node_92: feature_name=a-HuIgG_0.03	feature_id[14].value <= threshold=19665.666015625

node_93: feature_name=SARS.CoV.2.Spike.RBD.His.HEK	feature_id[4].value > threshold=3.0166666507720947
node_105: feature_name=SARS.CoV.2.S1.mFcTag	feature_id[0].value <= threshold=54010.166015625
node_106: feature_name=SARS.CoV.2.S1+S2	feature_id[7].value > threshold=48317.708984375
node_228: feature_name=a-MoIgG_0.10	feature_id[16].value > threshold=1652.5
node_230: feature_name=HuIgG_0.10	feature_id[9].value <= threshold=30107.666015625
node_231: feature_name=HuIgG_0.03	feature_id[8].value > threshold=5533.83349609375
node_247: feature_name=HuIgG_0.10	feature_id[9].value > threshold=24231.0
node_261: feature_name=SARS.CoV.S1.RBD.HisTag	feature_id[10].value <= threshold=24179.0419921875
node_262: feature_name=HuIgG_0.10	feature_id[9].value > threshold=25555.333984375
node_266: feature_name=HuIgG_0.10	feature_id[9].value > threshold=25659.6669921875
node_270: feature_name=SARS.CoV.S1.HisTag	feature_id[15].value > threshold=10000.5830078125
node_276: feature_name=SARS.CoV.S1.HisTag	feature_id[15].value > threshold=13228.125
node_286: feature_name=a-MoIgG_0.10	feature_id[16].value > threshold=10763.83349609375
node_290: feature_name=HuIgG_0.10	feature_id[9].value > threshold=27228.1669921875
Class: healthcare workers within 60 days after vaccination	
Rules_151	passed counts:1
node_0: feature_name=HuIgG_0.10	feature_id[9].value > threshold=19120.166015625
node_92: feature_name=a-HuIgG_0.03	feature_id[14].value <= threshold=19665.666015625
node_93: feature_name=SARS.CoV.2.Spike.RBD.His.HEK	feature_id[4].value > threshold=3.0166666507720947
node_105: feature_name=SARS.CoV.2.S1.mFcTag	feature_id[0].value <= threshold=54010.166015625
node_106: feature_name=SARS.CoV.2.S1+S2	feature_id[7].value > threshold=48317.708984375
node_228: feature_name=a-MoIgG_0.10	feature_id[16].value > threshold=1652.5
node_230: feature_name=HuIgG_0.10	feature_id[9].value <= threshold=30107.666015625
node_231: feature_name=HuIgG_0.03	feature_id[8].value > threshold=5533.83349609375

node_247: feature_name=HuIgG_0.10	feature_id[9].value > threshold=24231.0
node_261: feature_name=SARS.CoV.S1.RBD.HisTag	feature_id[10].value <= threshold=24179.0419921875
node_262: feature_name=HuIgG_0.10	feature_id[9].value > threshold=25555.333984375
node_266: feature_name=HuIgG_0.10	feature_id[9].value > threshold=25659.6669921875
node_270: feature_name=SARS.CoV.S1.HisTag	feature_id[15].value > threshold=10000.5830078125
node_276: feature_name=SARS.CoV.S1.HisTag	feature_id[15].value > threshold=13228.125
node_286: feature_name=a-MoIgG_0.10	feature_id[16].value <= threshold=10763.83349609375
node_287: feature_name=SARS.CoV.2.Spike.RBD.His.HEK	feature_id[4].value > threshold=46379.583984375
Class: healthcare workers between 60 and 180 days after vaccination	
Rules_152	passed counts:1
node_0: feature_name=HuIgG_0.10	feature_id[9].value > threshold=19120.166015625
node_92: feature_name=a-HuIgG_0.03	feature_id[14].value <= threshold=19665.666015625
node_93: feature_name=SARS.CoV.2.Spike.RBD.His.HEK	feature_id[4].value > threshold=3.0166666507720947
node_105: feature_name=SARS.CoV.2.S1.mFcTag	feature_id[0].value <= threshold=54010.166015625
node_106: feature_name=SARS.CoV.2.S1+S2	feature_id[7].value > threshold=48317.708984375
node_228: feature_name=a-MoIgG_0.10	feature_id[16].value > threshold=1652.5
node_230: feature_name=HuIgG_0.10	feature_id[9].value <= threshold=30107.666015625
node_231: feature_name=HuIgG_0.03	feature_id[8].value > threshold=5533.83349609375
node_247: feature_name=HuIgG_0.10	feature_id[9].value > threshold=24231.0
node_261: feature_name=SARS.CoV.S1.RBD.HisTag	feature_id[10].value <= threshold=24179.0419921875
node_262: feature_name=HuIgG_0.10	feature_id[9].value > threshold=25555.333984375
node_266: feature_name=HuIgG_0.10	feature_id[9].value > threshold=25659.6669921875
node_270: feature_name=SARS.CoV.S1.HisTag	feature_id[15].value > threshold=10000.5830078125
node_276: feature_name=SARS.CoV.S1.HisTag	feature_id[15].value <= threshold=13228.125

node_277: feature_name=SARS.CoV.2.Spike.RBD.His.Bac	feature_id[3].value > threshold=33671.91796875
node_279: feature_name=SARS.CoV.2.S1.RBD.mFc	feature_id[1].value > threshold=52697.666015625
Class: healthcare workers within 60 days after vaccination	
Rules_153	passed counts:1
node_0: feature_name=HuIgG_0.10	feature_id[9].value > threshold=19120.166015625
node_92: feature_name=a-HuIgG_0.03	feature_id[14].value <= threshold=19665.666015625
node_93: feature_name=SARS.CoV.2.Spike.RBD.His.HEK	feature_id[4].value > threshold=3.0166666507720947
node_105: feature_name=SARS.CoV.2.S1.mFcTag	feature_id[0].value <= threshold=54010.166015625
node_106: feature_name=SARS.CoV.2.S1+S2	feature_id[7].value > threshold=48317.708984375
node_228: feature_name=a-MoIgG_0.10	feature_id[16].value > threshold=1652.5
node_230: feature_name=HuIgG_0.10	feature_id[9].value <= threshold=30107.666015625
node_231: feature_name=HuIgG_0.03	feature_id[8].value > threshold=5533.83349609375
node_247: feature_name=HuIgG_0.10	feature_id[9].value > threshold=24231.0
node_261: feature_name=SARS.CoV.S1.RBD.HisTag	feature_id[10].value <= threshold=24179.0419921875
node_262: feature_name=HuIgG_0.10	feature_id[9].value > threshold=25555.333984375
node_266: feature_name=HuIgG_0.10	feature_id[9].value > threshold=25659.6669921875
node_270: feature_name=SARS.CoV.S1.HisTag	feature_id[15].value > threshold=10000.5830078125
node_276: feature_name=SARS.CoV.S1.HisTag	feature_id[15].value <= threshold=13228.125
node_277: feature_name=SARS.CoV.2.Spike.RBD.His.Bac	feature_id[3].value > threshold=33671.91796875
node_279: feature_name=SARS.CoV.2.S1.RBD.mFc	feature_id[1].value <= threshold=52697.666015625
node_280: feature_name=SARS.CoV.2.Spike.RBD.His.Bac	feature_id[3].value <= threshold=34984.25
node_281: feature_name=a-HuIgG_0.03	feature_id[14].value > threshold=14962.0
Class: healthcare workers within 60 days after vaccination	

Rules_154	passed counts:1
node_0: feature_name=HuIgG_0.10	feature_id[9].value > threshold=19120.166015625
node_92: feature_name=a-HuIgG_0.03	feature_id[14].value <= threshold=19665.666015625
node_93: feature_name=SARS.CoV.2.Spike.RBD.His.HEK	feature_id[4].value > threshold=3.0166666507720947
node_105: feature_name=SARS.CoV.2.S1.mFcTag	feature_id[0].value <= threshold=54010.166015625
node_106: feature_name=SARS.CoV.2.S1+S2	feature_id[7].value > threshold=48317.708984375
node_228: feature_name=a-MoIgG_0.10	feature_id[16].value > threshold=1652.5
node_230: feature_name=HuIgG_0.10	feature_id[9].value <= threshold=30107.666015625
node_231: feature_name=HuIgG_0.03	feature_id[8].value > threshold=5533.83349609375
node_247: feature_name=HuIgG_0.10	feature_id[9].value > threshold=24231.0
node_261: feature_name=SARS.CoV.S1.RBD.HisTag	feature_id[10].value <= threshold=24179.0419921875
node_262: feature_name=HuIgG_0.10	feature_id[9].value > threshold=25555.333984375
node_266: feature_name=HuIgG_0.10	feature_id[9].value > threshold=25659.6669921875
node_270: feature_name=SARS.CoV.S1.HisTag	feature_id[15].value > threshold=10000.5830078125
node_276: feature_name=SARS.CoV.S1.HisTag	feature_id[15].value <= threshold=13228.125
node_277: feature_name=SARS.CoV.2.Spike.RBD.His.Bac	feature_id[3].value > threshold=33671.91796875
node_279: feature_name=SARS.CoV.2.S1.RBD.mFc	feature_id[1].value <= threshold=52697.666015625
node_280: feature_name=SARS.CoV.2.Spike.RBD.His.Bac	feature_id[3].value <= threshold=34984.25
node_281: feature_name=a-HuIgG_0.03	feature_id[14].value <= threshold=14962.0
Class: healthcare workers between 60 and 180 days after vaccination	
Rules_155	passed counts:1
node_0: feature_name=HuIgG_0.10	feature_id[9].value > threshold=19120.166015625
node_92: feature_name=a-HuIgG_0.03	feature_id[14].value <= threshold=19665.666015625
node_93: feature_name=SARS.CoV.2.Spike.RBD.His.HEK	feature_id[4].value > threshold=3.0166666507720947

node_105: feature_name=SARS.CoV.2.S1.mFcTag	feature_id[0].value <= threshold=54010.166015625
node_106: feature_name=SARS.CoV.2.S1+S2	feature_id[7].value > threshold=48317.708984375
node_228: feature_name=a-MoIgG_0.10	feature_id[16].value > threshold=1652.5
node_230: feature_name=HuIgG_0.10	feature_id[9].value <= threshold=30107.666015625
node_231: feature_name=HuIgG_0.03	feature_id[8].value > threshold=5533.83349609375
node_247: feature_name=HuIgG_0.10	feature_id[9].value > threshold=24231.0
node_261: feature_name=SARS.CoV.S1.RBD.HisTag	feature_id[10].value <= threshold=24179.0419921875
node_262: feature_name=HuIgG_0.10	feature_id[9].value > threshold=25555.333984375
node_266: feature_name=HuIgG_0.10	feature_id[9].value <= threshold=25659.6669921875
node_267: feature_name=a-MoIgG_0.10	feature_id[16].value <= threshold=4330.1666259765625
Class: healthcare workers within 60 days after vaccination	
Rules_156	passed counts:1
node_0: feature_name=HuIgG_0.10	feature_id[9].value > threshold=19120.166015625
node_92: feature_name=a-HuIgG_0.03	feature_id[14].value <= threshold=19665.666015625
node_93: feature_name=SARS.CoV.2.Spike.RBD.His.HEK	feature_id[4].value > threshold=3.0166666507720947
node_105: feature_name=SARS.CoV.2.S1.mFcTag	feature_id[0].value <= threshold=54010.166015625
node_106: feature_name=SARS.CoV.2.S1+S2	feature_id[7].value > threshold=48317.708984375
node_228: feature_name=a-MoIgG_0.10	feature_id[16].value > threshold=1652.5
node_230: feature_name=HuIgG_0.10	feature_id[9].value <= threshold=30107.666015625
node_231: feature_name=HuIgG_0.03	feature_id[8].value > threshold=5533.83349609375
node_247: feature_name=HuIgG_0.10	feature_id[9].value > threshold=24231.0
node_261: feature_name=SARS.CoV.S1.RBD.HisTag	feature_id[10].value <= threshold=24179.0419921875
node_262: feature_name=HuIgG_0.10	feature_id[9].value <= threshold=25555.333984375
node_263: feature_name=SARS.CoV.2.S1+S2	feature_id[7].value > threshold=52323.916015625

Class: healthcare workers between 60 and 180 days after vaccination	
Rules_157	passed counts:1
node_0: feature_name=HuIgG_0.10	feature_id[9].value > threshold=19120.166015625
node_92: feature_name=a-HuIgG_0.03	feature_id[14].value <= threshold=19665.666015625
node_93: feature_name=SARS.CoV.2.Spike.RBD.His.HEK	feature_id[4].value > threshold=3.0166666507720947
node_105: feature_name=SARS.CoV.2.S1.mFcTag	feature_id[0].value <= threshold=54010.166015625
node_106: feature_name=SARS.CoV.2.S1+S2	feature_id[7].value > threshold=48317.708984375
node_228: feature_name=a-MoIgG_0.10	feature_id[16].value > threshold=1652.5
node_230: feature_name=HuIgG_0.10	feature_id[9].value <= threshold=30107.666015625
node_231: feature_name=HuIgG_0.03	feature_id[8].value > threshold=5533.83349609375
node_247: feature_name=HuIgG_0.10	feature_id[9].value <= threshold=24231.0
node_248: feature_name=SARS.CoV.2.S1.HisTag	feature_id[2].value > threshold=43585.92578125
node_256: feature_name=SARS.CoV.2.S1+S2	feature_id[7].value <= threshold=52592.591796875
node_257: feature_name=SARS.CoV.2.S1.RBD.mFc	feature_id[1].value <= threshold=43424.416015625
Class: healthcare workers between 60 and 180 days after vaccination	
Rules_158	passed counts:1
node_0: feature_name=HuIgG_0.10	feature_id[9].value > threshold=19120.166015625
node_92: feature_name=a-HuIgG_0.03	feature_id[14].value <= threshold=19665.666015625
node_93: feature_name=SARS.CoV.2.Spike.RBD.His.HEK	feature_id[4].value > threshold=3.0166666507720947
node_105: feature_name=SARS.CoV.2.S1.mFcTag	feature_id[0].value <= threshold=54010.166015625
node_106: feature_name=SARS.CoV.2.S1+S2	feature_id[7].value > threshold=48317.708984375
node_228: feature_name=a-MoIgG_0.10	feature_id[16].value > threshold=1652.5
node_230: feature_name=HuIgG_0.10	feature_id[9].value <= threshold=30107.666015625

node_231: feature_name=HuIgG_0.03	feature_id[8].value > threshold=5533.83349609375
node_247: feature_name=HuIgG_0.10	feature_id[9].value <= threshold=24231.0
node_248: feature_name=SARS.CoV.2.S1.HisTag	feature_id[2].value <= threshold=43585.92578125
node_249: feature_name=HuIgG_0.10	feature_id[9].value <= threshold=23668.3330078125
node_250: feature_name=SARS.CoV.2.S1+S2	feature_id[7].value > threshold=49682.4921875
node_252: feature_name=a-HuIgG_0.30	feature_id[11].value > threshold=39201.833984375
Class: healthcare workers within 60 days after vaccination	
Rules_159	passed counts:1
node_0: feature_name=HuIgG_0.10	feature_id[9].value > threshold=19120.166015625
node_92: feature_name=a-HuIgG_0.03	feature_id[14].value <= threshold=19665.666015625
node_93: feature_name=SARS.CoV.2.Spike.RBD.His.HEK	feature_id[4].value > threshold=3.0166666507720947
node_105: feature_name=SARS.CoV.2.S1.mFcTag	feature_id[0].value <= threshold=54010.166015625
node_106: feature_name=SARS.CoV.2.S1+S2	feature_id[7].value > threshold=48317.708984375
node_228: feature_name=a-MoIgG_0.10	feature_id[16].value > threshold=1652.5
node_230: feature_name=HuIgG_0.10	feature_id[9].value <= threshold=30107.666015625
node_231: feature_name=HuIgG_0.03	feature_id[8].value <= threshold=5533.83349609375
node_232: feature_name=SARS.CoV.2.Spike.RBD.His.HEK	feature_id[4].value > threshold=11116.333374023438
node_234: feature_name=SARS.CoV.2.S1+S2	feature_id[7].value <= threshold=48882.58203125
node_235: feature_name=SARS.CoV.2.Spike.RBD.rFc	feature_id[5].value <= threshold=45170.75
node_236: feature_name=a-HuIgG_0.03	feature_id[14].value <= threshold=11110.0
Class: healthcare workers within 60 days after vaccination	
Rules_160	passed counts:1
node_0: feature_name=HuIgG_0.10	feature_id[9].value > threshold=19120.166015625

node_92: feature_name=a-HuIgG_0.03	feature_id[14].value <= threshold=19665.666015625
node_93: feature_name=SARS.CoV.2.Spike.RBD.His.HEK	feature_id[4].value > threshold=3.0166666507720947
node_105: feature_name=SARS.CoV.2.S1.mFcTag	feature_id[0].value <= threshold=54010.166015625
node_106: feature_name=SARS.CoV.2.S1+S2	feature_id[7].value > threshold=48317.708984375
node_228: feature_name=a-MoIgG_0.10	feature_id[16].value > threshold=1652.5
node_230: feature_name=HuIgG_0.10	feature_id[9].value <= threshold=30107.666015625
node_231: feature_name=HuIgG_0.03	feature_id[8].value <= threshold=5533.83349609375
node_232: feature_name=SARS.CoV.2.Spike.RBD.His.HEK	feature_id[4].value <= threshold=11116.333374023438
Class: unvaccinated healthcare workers	
Rules_161	passed counts:1
node_0: feature_name=HuIgG_0.10	feature_id[9].value > threshold=19120.166015625
node_92: feature_name=a-HuIgG_0.03	feature_id[14].value <= threshold=19665.666015625
node_93: feature_name=SARS.CoV.2.Spike.RBD.His.HEK	feature_id[4].value > threshold=3.0166666507720947
node_105: feature_name=SARS.CoV.2.S1.mFcTag	feature_id[0].value <= threshold=54010.166015625
node_106: feature_name=SARS.CoV.2.S1+S2	feature_id[7].value <= threshold=48317.708984375
node_107: feature_name=a-HuIgG_0.03	feature_id[14].value > threshold=12099.83349609375
node_155: feature_name=SARS.CoV.2.Spike.RBD.His.Bac	feature_id[3].value > threshold=18979.9912109375
node_167: feature_name=a-MoIgG_0.10	feature_id[16].value > threshold=4979.5
node_201: feature_name=HuIgG_0.10	feature_id[9].value <= threshold=26006.0
node_202: feature_name=HuIgG_0.03	feature_id[8].value > threshold=6080.0
node_216: feature_name=SARS.CoV.2.Spike.RBD.rFc	feature_id[5].value > threshold=43169.25
node_220: feature_name=SARS.CoV.2.Spike.RBD.His.Bac	feature_id[3].value <= threshold=34138.75
node_221: feature_name=SARS.CoV.2.Spike.RBD.His.HEK	feature_id[4].value > threshold=37141.416015625
Class: healthcare workers between 60 and 180 days after vaccination	

Rules_162	passed counts:1
node_0: feature_name=HuIgG_0.10	feature_id[9].value > threshold=19120.166015625
node_92: feature_name=a-HuIgG_0.03	feature_id[14].value <= threshold=19665.666015625
node_93: feature_name=SARS.CoV.2.Spike.RBD.His.HEK	feature_id[4].value > threshold=3.0166666507720947
node_105: feature_name=SARS.CoV.2.S1.mFcTag	feature_id[0].value <= threshold=54010.166015625
node_106: feature_name=SARS.CoV.2.S1+S2	feature_id[7].value <= threshold=48317.708984375
node_107: feature_name=a-HuIgG_0.03	feature_id[14].value > threshold=12099.83349609375
node_155: feature_name=SARS.CoV.2.Spike.RBD.His.Bac	feature_id[3].value > threshold=18979.9912109375
node_167: feature_name=a-MoIgG_0.10	feature_id[16].value > threshold=4979.5
node_201: feature_name=HuIgG_0.10	feature_id[9].value <= threshold=26006.0
node_202: feature_name=HuIgG_0.03	feature_id[8].value > threshold=6080.0
node_216: feature_name=SARS.CoV.2.Spike.RBD.rFc	feature_id[5].value <= threshold=43169.25
node_217: feature_name=SARS.CoV.2.Spike.RBD.His.HEK	feature_id[4].value <= threshold=12896.658203125
Class: healthcare workers within 60 days after vaccination	
Rules_163	passed counts:1
node_0: feature_name=HuIgG_0.10	feature_id[9].value > threshold=19120.166015625
node_92: feature_name=a-HuIgG_0.03	feature_id[14].value <= threshold=19665.666015625
node_93: feature_name=SARS.CoV.2.Spike.RBD.His.HEK	feature_id[4].value > threshold=3.0166666507720947
node_105: feature_name=SARS.CoV.2.S1.mFcTag	feature_id[0].value <= threshold=54010.166015625
node_106: feature_name=SARS.CoV.2.S1+S2	feature_id[7].value <= threshold=48317.708984375
node_107: feature_name=a-HuIgG_0.03	feature_id[14].value > threshold=12099.83349609375
node_155: feature_name=SARS.CoV.2.Spike.RBD.His.Bac	feature_id[3].value > threshold=18979.9912109375
node_167: feature_name=a-MoIgG_0.10	feature_id[16].value > threshold=4979.5

node_201: feature_name=HuIgG_0.10	feature_id[9].value <= threshold=26006.0
node_202: feature_name=HuIgG_0.03	feature_id[8].value <= threshold=6080.0
node_203: feature_name=SARS.CoV.2.S1.HisTag	feature_id[2].value > threshold=38575.359375
node_213: feature_name=SARS.CoV.2.Spike.RBD.His.HEK	feature_id[4].value > threshold=39411.75
Class: healthcare workers within 60 days after vaccination	
Rules_164	passed counts:1
node_0: feature_name=HuIgG_0.10	feature_id[9].value > threshold=19120.166015625
node_92: feature_name=a-HuIgG_0.03	feature_id[14].value <= threshold=19665.666015625
node_93: feature_name=SARS.CoV.2.Spike.RBD.His.HEK	feature_id[4].value > threshold=3.0166666507720947
node_105: feature_name=SARS.CoV.2.S1.mFcTag	feature_id[0].value <= threshold=54010.166015625
node_106: feature_name=SARS.CoV.2.S1+S2	feature_id[7].value <= threshold=48317.708984375
node_107: feature_name=a-HuIgG_0.03	feature_id[14].value > threshold=12099.83349609375
node_155: feature_name=SARS.CoV.2.Spike.RBD.His.Bac	feature_id[3].value > threshold=18979.9912109375
node_167: feature_name=a-MoIgG_0.10	feature_id[16].value > threshold=4979.5
node_201: feature_name=HuIgG_0.10	feature_id[9].value <= threshold=26006.0
node_202: feature_name=HuIgG_0.03	feature_id[8].value <= threshold=6080.0
node_203: feature_name=SARS.CoV.2.S1.HisTag	feature_id[2].value <= threshold=38575.359375
node_204: feature_name=SARS.CoV.2.Spike.RBD.His.HEK	feature_id[4].value > threshold=21567.4501953125
node_208: feature_name=HuIgG_0.03	feature_id[8].value <= threshold=4924.333251953125
node_209: feature_name=SARS.CoV.2.Spike.RBD.His.Bac	feature_id[3].value > threshold=31771.125
Class: healthcare workers within 60 days after vaccination	
Rules_165	passed counts:1
node_0: feature_name=HuIgG_0.10	feature_id[9].value > threshold=19120.166015625

node_92: feature_name=a-HuIgG_0.03	feature_id[14].value <= threshold=19665.666015625
node_93: feature_name=SARS.CoV.2.Spike.RBD.His.HEK	feature_id[4].value > threshold=3.0166666507720947
node_105: feature_name=SARS.CoV.2.S1.mFcTag	feature_id[0].value <= threshold=54010.166015625
node_106: feature_name=SARS.CoV.2.S1+S2	feature_id[7].value <= threshold=48317.708984375
node_107: feature_name=a-HuIgG_0.03	feature_id[14].value > threshold=12099.83349609375
node_155: feature_name=SARS.CoV.2.Spike.RBD.His.Bac	feature_id[3].value > threshold=18979.9912109375
node_167: feature_name=a-MoIgG_0.10	feature_id[16].value > threshold=4979.5
node_201: feature_name=HuIgG_0.10	feature_id[9].value <= threshold=26006.0
node_202: feature_name=HuIgG_0.03	feature_id[8].value <= threshold=6080.0
node_203: feature_name=SARS.CoV.2.S1.HisTag	feature_id[2].value <= threshold=38575.359375
node_204: feature_name=SARS.CoV.2.Spike.RBD.His.HEK	feature_id[4].value <= threshold=21567.4501953125
node_205: feature_name=HuIgG_0.10	feature_id[9].value <= threshold=23045.6669921875
Class: healthcare workers within 60 days after vaccination	
Rules_166	passed counts:1
node_0: feature_name=HuIgG_0.10	feature_id[9].value > threshold=19120.166015625
node_92: feature_name=a-HuIgG_0.03	feature_id[14].value <= threshold=19665.666015625
node_93: feature_name=SARS.CoV.2.Spike.RBD.His.HEK	feature_id[4].value > threshold=3.0166666507720947
node_105: feature_name=SARS.CoV.2.S1.mFcTag	feature_id[0].value <= threshold=54010.166015625
node_106: feature_name=SARS.CoV.2.S1+S2	feature_id[7].value <= threshold=48317.708984375
node_107: feature_name=a-HuIgG_0.03	feature_id[14].value > threshold=12099.83349609375
node_155: feature_name=SARS.CoV.2.Spike.RBD.His.Bac	feature_id[3].value > threshold=18979.9912109375
node_167: feature_name=a-MoIgG_0.10	feature_id[16].value <= threshold=4979.5
node_168: feature_name=SARS.CoV.2.S1+S2	feature_id[7].value > threshold=36298.75
node_172: feature_name=SARS.CoV.2.S1.RBD.mFc	feature_id[1].value > threshold=40153.283203125

node_182: feature_name=HuIgG_0.10	feature_id[9].value > threshold=21919.833984375
node_186: feature_name=a-HuIgG_0.10	feature_id[13].value > threshold=28919.0
node_192: feature_name=a-HuIgG_0.10	feature_id[13].value <= threshold=31482.6669921875
node_193: feature_name=a-HuIgG_0.03	feature_id[14].value > threshold=12541.0
node_195: feature_name=a-HuIgG_0.30	feature_id[11].value <= threshold=40626.0
Class: healthcare workers within 60 days after vaccination	
Rules_167	passed counts:1
node_0: feature_name=HuIgG_0.10	feature_id[9].value > threshold=19120.166015625
node_92: feature_name=a-HuIgG_0.03	feature_id[14].value <= threshold=19665.666015625
node_93: feature_name=SARS.CoV.2.Spike.RBD.His.HEK	feature_id[4].value > threshold=3.0166666507720947
node_105: feature_name=SARS.CoV.2.S1.mFcTag	feature_id[0].value <= threshold=54010.166015625
node_106: feature_name=SARS.CoV.2.S1+S2	feature_id[7].value <= threshold=48317.708984375
node_107: feature_name=a-HuIgG_0.03	feature_id[14].value > threshold=12099.83349609375
node_155: feature_name=SARS.CoV.2.Spike.RBD.His.Bac	feature_id[3].value > threshold=18979.9912109375
node_167: feature_name=a-MoIgG_0.10	feature_id[16].value <= threshold=4979.5
node_168: feature_name=SARS.CoV.2.S1+S2	feature_id[7].value > threshold=36298.75
node_172: feature_name=SARS.CoV.2.S1.RBD.mFc	feature_id[1].value > threshold=40153.283203125
node_182: feature_name=HuIgG_0.10	feature_id[9].value > threshold=21919.833984375
node_186: feature_name=a-HuIgG_0.10	feature_id[13].value <= threshold=28919.0
node_187: feature_name=SARS.CoV.S1.HisTag	feature_id[15].value > threshold=4488.5833740234375
node_189: feature_name=a-HuIgG_0.10	feature_id[13].value <= threshold=26194.3330078125
Class: healthcare workers between 60 and 180 days after vaccination	
Rules_168	passed counts:1

node_0: feature_name=HuIgG_0.10	feature_id[9].value > threshold=19120.166015625
node_92: feature_name=a-HuIgG_0.03	feature_id[14].value <= threshold=19665.666015625
node_93: feature_name=SARS.CoV.2.Spike.RBD.His.HEK	feature_id[4].value > threshold=3.0166666507720947
node_105: feature_name=SARS.CoV.2.S1.mFcTag	feature_id[0].value <= threshold=54010.166015625
node_106: feature_name=SARS.CoV.2.S1+S2	feature_id[7].value <= threshold=48317.708984375
node_107: feature_name=a-HuIgG_0.03	feature_id[14].value > threshold=12099.83349609375
node_155: feature_name=SARS.CoV.2.Spike.RBD.His.Bac	feature_id[3].value > threshold=18979.9912109375
node_167: feature_name=a-MoIgG_0.10	feature_id[16].value <= threshold=4979.5
node_168: feature_name=SARS.CoV.2.S1+S2	feature_id[7].value > threshold=36298.75
node_172: feature_name=SARS.CoV.2.S1.RBD.mFc	feature_id[1].value > threshold=40153.283203125
node_182: feature_name=HuIgG_0.10	feature_id[9].value <= threshold=21919.833984375
node_183: feature_name=a-HuIgG_0.30	feature_id[11].value > threshold=51690.5
Class: healthcare workers over 180 days after vaccination	
Rules_169	passed counts:1
node_0: feature_name=HuIgG_0.10	feature_id[9].value > threshold=19120.166015625
node_92: feature_name=a-HuIgG_0.03	feature_id[14].value <= threshold=19665.666015625
node_93: feature_name=SARS.CoV.2.Spike.RBD.His.HEK	feature_id[4].value > threshold=3.0166666507720947
node_105: feature_name=SARS.CoV.2.S1.mFcTag	feature_id[0].value <= threshold=54010.166015625
node_106: feature_name=SARS.CoV.2.S1+S2	feature_id[7].value <= threshold=48317.708984375
node_107: feature_name=a-HuIgG_0.03	feature_id[14].value > threshold=12099.83349609375
node_155: feature_name=SARS.CoV.2.Spike.RBD.His.Bac	feature_id[3].value > threshold=18979.9912109375
node_167: feature_name=a-MoIgG_0.10	feature_id[16].value <= threshold=4979.5
node_168: feature_name=SARS.CoV.2.S1+S2	feature_id[7].value > threshold=36298.75
node_172: feature_name=SARS.CoV.2.S1.RBD.mFc	feature_id[1].value <= threshold=40153.283203125

node_173: feature_name=SARS.CoV.2.S1.HisTag	feature_id[2].value > threshold=25186.375
node_175: feature_name=a-HuIgG_0.10	feature_id[13].value > threshold=27831.166015625
node_177: feature_name=SARS.CoV.2.S1	feature_id[6].value <= threshold=29819.333984375
node_178: feature_name=SARS.CoV.2.S1.HisTag	feature_id[2].value > threshold=28188.974609375
Class: healthcare workers within 60 days after vaccination	
Rules_170	passed counts:1
node_0: feature_name=HuIgG_0.10	feature_id[9].value > threshold=19120.166015625
node_92: feature_name=a-HuIgG_0.03	feature_id[14].value <= threshold=19665.666015625
node_93: feature_name=SARS.CoV.2.Spike.RBD.His.HEK	feature_id[4].value > threshold=3.0166666507720947
node_105: feature_name=SARS.CoV.2.S1.mFcTag	feature_id[0].value <= threshold=54010.166015625
node_106: feature_name=SARS.CoV.2.S1+S2	feature_id[7].value <= threshold=48317.708984375
node_107: feature_name=a-HuIgG_0.03	feature_id[14].value > threshold=12099.83349609375
node_155: feature_name=SARS.CoV.2.Spike.RBD.His.Bac	feature_id[3].value > threshold=18979.9912109375
node_167: feature_name=a-MoIgG_0.10	feature_id[16].value <= threshold=4979.5
node_168: feature_name=SARS.CoV.2.S1+S2	feature_id[7].value > threshold=36298.75
node_172: feature_name=SARS.CoV.2.S1.RBD.mFc	feature_id[1].value <= threshold=40153.283203125
node_173: feature_name=SARS.CoV.2.S1.HisTag	feature_id[2].value > threshold=25186.375
node_175: feature_name=a-HuIgG_0.10	feature_id[13].value <= threshold=27831.166015625
Class: healthcare workers within 60 days after vaccination	
Rules_171	passed counts:1
node_0: feature_name=HuIgG_0.10	feature_id[9].value > threshold=19120.166015625
node_92: feature_name=a-HuIgG_0.03	feature_id[14].value <= threshold=19665.666015625
node_93: feature_name=SARS.CoV.2.Spike.RBD.His.HEK	feature_id[4].value > threshold=3.0166666507720947

node_105: feature_name=SARS.CoV.2.S1.mFcTag	feature_id[0].value <= threshold=54010.166015625
node_106: feature_name=SARS.CoV.2.S1+S2	feature_id[7].value <= threshold=48317.708984375
node_107: feature_name=a-HuIgG_0.03	feature_id[14].value > threshold=12099.83349609375
node_155: feature_name=SARS.CoV.2.Spike.RBD.His.Bac	feature_id[3].value > threshold=18979.9912109375
node_167: feature_name=a-MoIgG_0.10	feature_id[16].value <= threshold=4979.5
node_168: feature_name=SARS.CoV.2.S1+S2	feature_id[7].value > threshold=36298.75
node_172: feature_name=SARS.CoV.2.S1.RBD.mFc	feature_id[1].value <= threshold=40153.283203125
node_173: feature_name=SARS.CoV.2.S1.HisTag	feature_id[2].value <= threshold=25186.375
Class: healthcare workers within 60 days after vaccination	
Rules_172	passed counts:1
node_0: feature_name=HuIgG_0.10	feature_id[9].value > threshold=19120.166015625
node_92: feature_name=a-HuIgG_0.03	feature_id[14].value <= threshold=19665.666015625
node_93: feature_name=SARS.CoV.2.Spike.RBD.His.HEK	feature_id[4].value > threshold=3.0166666507720947
node_105: feature_name=SARS.CoV.2.S1.mFcTag	feature_id[0].value <= threshold=54010.166015625
node_106: feature_name=SARS.CoV.2.S1+S2	feature_id[7].value <= threshold=48317.708984375
node_107: feature_name=a-HuIgG_0.03	feature_id[14].value > threshold=12099.83349609375
node_155: feature_name=SARS.CoV.2.Spike.RBD.His.Bac	feature_id[3].value <= threshold=18979.9912109375
node_156: feature_name=a-MoIgG_0.10	feature_id[16].value > threshold=1739.1666259765625
node_160: feature_name=HuIgG_0.30	feature_id[12].value > threshold=34829.83203125
node_162: feature_name=HuIgG_0.03	feature_id[8].value > threshold=6681.166748046875
node_164: feature_name=HuIgG_0.30	feature_id[12].value > threshold=40459.166015625
Class: unvaccinated healthcare workers	
Rules_173	passed counts:1

node_0: feature_name=HuIgG_0.10	feature_id[9].value > threshold=19120.166015625
node_92: feature_name=a-HuIgG_0.03	feature_id[14].value <= threshold=19665.666015625
node_93: feature_name=SARS.CoV.2.Spike.RBD.His.HEK	feature_id[4].value > threshold=3.0166666507720947
node_105: feature_name=SARS.CoV.2.S1.mFcTag	feature_id[0].value <= threshold=54010.166015625
node_106: feature_name=SARS.CoV.2.S1+S2	feature_id[7].value <= threshold=48317.708984375
node_107: feature_name=a-HuIgG_0.03	feature_id[14].value > threshold=12099.83349609375
node_155: feature_name=SARS.CoV.2.Spike.RBD.His.Bac	feature_id[3].value <= threshold=18979.9912109375
node_156: feature_name=a-MoIgG_0.10	feature_id[16].value > threshold=1739.1666259765625
node_160: feature_name=HuIgG_0.30	feature_id[12].value <= threshold=34829.83203125
Class: unvaccinated healthcare workers	
Rules_174	passed counts:1
node_0: feature_name=HuIgG_0.10	feature_id[9].value > threshold=19120.166015625
node_92: feature_name=a-HuIgG_0.03	feature_id[14].value <= threshold=19665.666015625
node_93: feature_name=SARS.CoV.2.Spike.RBD.His.HEK	feature_id[4].value > threshold=3.0166666507720947
node_105: feature_name=SARS.CoV.2.S1.mFcTag	feature_id[0].value <= threshold=54010.166015625
node_106: feature_name=SARS.CoV.2.S1+S2	feature_id[7].value <= threshold=48317.708984375
node_107: feature_name=a-HuIgG_0.03	feature_id[14].value > threshold=12099.83349609375
node_155: feature_name=SARS.CoV.2.Spike.RBD.His.Bac	feature_id[3].value <= threshold=18979.9912109375
node_156: feature_name=a-MoIgG_0.10	feature_id[16].value <= threshold=1739.1666259765625
node_157: feature_name=SARS.CoV.S1.RBD.HisTag	feature_id[10].value <= threshold=1304.0555419921875
Class: unvaccinated healthcare workers	
Rules_175	passed counts:1
node_0: feature_name=HuIgG_0.10	feature_id[9].value > threshold=19120.166015625

node_92: feature_name=a-HuIgG_0.03	feature_id[14].value <= threshold=19665.666015625
node_93: feature_name=SARS.CoV.2.Spike.RBD.His.HEK	feature_id[4].value > threshold=3.0166666507720947
node_105: feature_name=SARS.CoV.2.S1.mFcTag	feature_id[0].value <= threshold=54010.166015625
node_106: feature_name=SARS.CoV.2.S1+S2	feature_id[7].value <= threshold=48317.708984375
node_107: feature_name=a-HuIgG_0.03	feature_id[14].value <= threshold=12099.83349609375
node_108: feature_name=SARS.CoV.2.Spike.RBD.His.Bac	feature_id[3].value > threshold=23841.4169921875
node_118: feature_name=SARS.CoV.2.Spike.RBD.rFc	feature_id[5].value > threshold=34554.69140625
node_120: feature_name=SARS.CoV.2.S1	feature_id[6].value > threshold=36508.716796875
node_142: feature_name=HuIgG_0.30	feature_id[12].value > threshold=40622.83203125
node_150: feature_name=SARS.CoV.S1.RBD.HisTag	feature_id[10].value > threshold=9578.58349609375
node_152: feature_name=SARS.CoV.2.Spike.RBD.His.Bac	feature_id[3].value > threshold=30984.7919921875
Class: healthcare workers within 60 days after vaccination	
Rules_176	passed counts:1
node_0: feature_name=HuIgG_0.10	feature_id[9].value > threshold=19120.166015625
node_92: feature_name=a-HuIgG_0.03	feature_id[14].value <= threshold=19665.666015625
node_93: feature_name=SARS.CoV.2.Spike.RBD.His.HEK	feature_id[4].value > threshold=3.0166666507720947
node_105: feature_name=SARS.CoV.2.S1.mFcTag	feature_id[0].value <= threshold=54010.166015625
node_106: feature_name=SARS.CoV.2.S1+S2	feature_id[7].value <= threshold=48317.708984375
node_107: feature_name=a-HuIgG_0.03	feature_id[14].value <= threshold=12099.83349609375
node_108: feature_name=SARS.CoV.2.Spike.RBD.His.Bac	feature_id[3].value > threshold=23841.4169921875
node_118: feature_name=SARS.CoV.2.Spike.RBD.rFc	feature_id[5].value > threshold=34554.69140625
node_120: feature_name=SARS.CoV.2.S1	feature_id[6].value > threshold=36508.716796875
node_142: feature_name=HuIgG_0.30	feature_id[12].value <= threshold=40622.83203125
node_143: feature_name=HuIgG_0.10	feature_id[9].value <= threshold=21439.1669921875

node_144: feature_name=SARS.CoV.2.S1.mFcTag	feature_id[0].value > threshold=51094.66796875
node_146: feature_name=a-HuIgG_0.03	feature_id[14].value <= threshold=9792.33349609375
Class: healthcare workers within 60 days after vaccination	
Rules_177	passed counts:1
node_0: feature_name=HuIgG_0.10	feature_id[9].value > threshold=19120.166015625
node_92: feature_name=a-HuIgG_0.03	feature_id[14].value <= threshold=19665.666015625
node_93: feature_name=SARS.CoV.2.Spike.RBD.His.HEK	feature_id[4].value > threshold=3.0166666507720947
node_105: feature_name=SARS.CoV.2.S1.mFcTag	feature_id[0].value <= threshold=54010.166015625
node_106: feature_name=SARS.CoV.2.S1+S2	feature_id[7].value <= threshold=48317.708984375
node_107: feature_name=a-HuIgG_0.03	feature_id[14].value <= threshold=12099.83349609375
node_108: feature_name=SARS.CoV.2.Spike.RBD.His.Bac	feature_id[3].value > threshold=23841.4169921875
node_118: feature_name=SARS.CoV.2.Spike.RBD.rFc	feature_id[5].value > threshold=34554.69140625
node_120: feature_name=SARS.CoV.2.S1	feature_id[6].value <= threshold=36508.716796875
node_121: feature_name=HuIgG_0.10	feature_id[9].value > threshold=24763.3330078125
node_139: feature_name=SARS.CoV.2.Spike.RBD.His.Bac	feature_id[3].value > threshold=33242.83203125
Class: healthcare workers between 60 and 180 days after vaccination	
Rules_178	passed counts:1
node_0: feature_name=HuIgG_0.10	feature_id[9].value > threshold=19120.166015625
node_92: feature_name=a-HuIgG_0.03	feature_id[14].value <= threshold=19665.666015625
node_93: feature_name=SARS.CoV.2.Spike.RBD.His.HEK	feature_id[4].value > threshold=3.0166666507720947
node_105: feature_name=SARS.CoV.2.S1.mFcTag	feature_id[0].value <= threshold=54010.166015625
node_106: feature_name=SARS.CoV.2.S1+S2	feature_id[7].value <= threshold=48317.708984375
node_107: feature_name=a-HuIgG_0.03	feature_id[14].value <= threshold=12099.83349609375

node_108: feature_name=SARS.CoV.2.Spike.RBD.His.Bac	feature_id[3].value > threshold=23841.4169921875
node_118: feature_name=SARS.CoV.2.Spike.RBD.rFc	feature_id[5].value > threshold=34554.69140625
node_120: feature_name=SARS.CoV.2.S1	feature_id[6].value <= threshold=36508.716796875
node_121: feature_name=HuIgG_0.10	feature_id[9].value <= threshold=24763.3330078125
node_122: feature_name=SARS.CoV.2.Spike.RBD.rFc	feature_id[5].value <= threshold=42966.541015625
node_123: feature_name=SARS.CoV.2.S1+S2	feature_id[7].value > threshold=43434.54296875
node_135: feature_name=HuIgG_0.10	feature_id[9].value > threshold=24375.1669921875
Class: healthcare workers between 60 and 180 days after vaccination	
Rules_179	passed counts:1
node_0: feature_name=HuIgG_0.10	feature_id[9].value > threshold=19120.166015625
node_92: feature_name=a-HuIgG_0.03	feature_id[14].value <= threshold=19665.666015625
node_93: feature_name=SARS.CoV.2.Spike.RBD.His.HEK	feature_id[4].value > threshold=3.0166666507720947
node_105: feature_name=SARS.CoV.2.S1.mFcTag	feature_id[0].value <= threshold=54010.166015625
node_106: feature_name=SARS.CoV.2.S1+S2	feature_id[7].value <= threshold=48317.708984375
node_107: feature_name=a-HuIgG_0.03	feature_id[14].value <= threshold=12099.83349609375
node_108: feature_name=SARS.CoV.2.Spike.RBD.His.Bac	feature_id[3].value > threshold=23841.4169921875
node_118: feature_name=SARS.CoV.2.Spike.RBD.rFc	feature_id[5].value > threshold=34554.69140625
node_120: feature_name=SARS.CoV.2.S1	feature_id[6].value <= threshold=36508.716796875
node_121: feature_name=HuIgG_0.10	feature_id[9].value <= threshold=24763.3330078125
node_122: feature_name=SARS.CoV.2.Spike.RBD.rFc	feature_id[5].value <= threshold=42966.541015625
node_123: feature_name=SARS.CoV.2.S1+S2	feature_id[7].value <= threshold=43434.54296875
node_124: feature_name=SARS.CoV.2.S1+S2	feature_id[7].value <= threshold=42800.94140625
node_125: feature_name=SARS.CoV.2.S1.mFcTag	feature_id[0].value > threshold=47427.1328125
node_129: feature_name=a-MoIgG_0.10	feature_id[16].value > threshold=5634.0

node_131: feature_name=SARS.CoV.2.S1.RBD.mFc	feature_id[1].value <= threshold=39372.166015625
Class: healthcare workers over 180 days after vaccination	
Rules_180	passed counts:1
node_0: feature_name=HuIgG_0.10	feature_id[9].value > threshold=19120.166015625
node_92: feature_name=a-HuIgG_0.03	feature_id[14].value <= threshold=19665.666015625
node_93: feature_name=SARS.CoV.2.Spike.RBD.His.HEK	feature_id[4].value > threshold=3.0166666507720947
node_105: feature_name=SARS.CoV.2.S1.mFcTag	feature_id[0].value <= threshold=54010.166015625
node_106: feature_name=SARS.CoV.2.S1+S2	feature_id[7].value <= threshold=48317.708984375
node_107: feature_name=a-HuIgG_0.03	feature_id[14].value <= threshold=12099.83349609375
node_108: feature_name=SARS.CoV.2.Spike.RBD.His.Bac	feature_id[3].value > threshold=23841.4169921875
node_118: feature_name=SARS.CoV.2.Spike.RBD.rFc	feature_id[5].value > threshold=34554.69140625
node_120: feature_name=SARS.CoV.2.S1	feature_id[6].value <= threshold=36508.716796875
node_121: feature_name=HuIgG_0.10	feature_id[9].value <= threshold=24763.3330078125
node_122: feature_name=SARS.CoV.2.Spike.RBD.rFc	feature_id[5].value <= threshold=42966.541015625
node_123: feature_name=SARS.CoV.2.S1+S2	feature_id[7].value <= threshold=43434.54296875
node_124: feature_name=SARS.CoV.2.S1+S2	feature_id[7].value <= threshold=42800.94140625
node_125: feature_name=SARS.CoV.2.S1.mFcTag	feature_id[0].value <= threshold=47427.1328125
node_126: feature_name=SARS.CoV.2.Spike.RBD.rFc	feature_id[5].value > threshold=37955.708984375
Class: healthcare workers within 60 days after vaccination	
Rules_181	passed counts:1
node_0: feature_name=HuIgG_0.10	feature_id[9].value > threshold=19120.166015625
node_92: feature_name=a-HuIgG_0.03	feature_id[14].value <= threshold=19665.666015625
node_93: feature_name=SARS.CoV.2.Spike.RBD.His.HEK	feature_id[4].value > threshold=3.0166666507720947

node_105: feature_name=SARS.CoV.2.S1.mFcTag	feature_id[0].value <= threshold=54010.166015625
node_106: feature_name=SARS.CoV.2.S1+S2	feature_id[7].value <= threshold=48317.708984375
node_107: feature_name=a-HuIgG_0.03	feature_id[14].value <= threshold=12099.83349609375
node_108: feature_name=SARS.CoV.2.Spike.RBD.His.Bac	feature_id[3].value <= threshold=23841.4169921875
node_109: feature_name=SARS.CoV.2.S1+S2	feature_id[7].value <= threshold=36783.25
node_110: feature_name=SARS.CoV.2.S1	feature_id[6].value <= threshold=26291.7998046875
node_111: feature_name=HuIgG_0.10	feature_id[9].value > threshold=20106.3330078125
node_113: feature_name=HuIgG_0.03	feature_id[8].value > threshold=5848.666748046875
Class: healthcare workers between 60 and 180 days after vaccination	
Rules_182	passed counts:1
node_0: feature_name=HuIgG_0.10	feature_id[9].value > threshold=19120.166015625
node_92: feature_name=a-HuIgG_0.03	feature_id[14].value <= threshold=19665.666015625
node_93: feature_name=SARS.CoV.2.Spike.RBD.His.HEK	feature_id[4].value > threshold=3.0166666507720947
node_105: feature_name=SARS.CoV.2.S1.mFcTag	feature_id[0].value <= threshold=54010.166015625
node_106: feature_name=SARS.CoV.2.S1+S2	feature_id[7].value <= threshold=48317.708984375
node_107: feature_name=a-HuIgG_0.03	feature_id[14].value <= threshold=12099.83349609375
node_108: feature_name=SARS.CoV.2.Spike.RBD.His.Bac	feature_id[3].value <= threshold=23841.4169921875
node_109: feature_name=SARS.CoV.2.S1+S2	feature_id[7].value <= threshold=36783.25
node_110: feature_name=SARS.CoV.2.S1	feature_id[6].value <= threshold=26291.7998046875
node_111: feature_name=HuIgG_0.10	feature_id[9].value <= threshold=20106.3330078125
Class: healthcare workers between 60 and 180 days after vaccination	
Rules_183	passed counts:1
node_0: feature_name=HuIgG_0.10	feature_id[9].value > threshold=19120.166015625

node_92: feature_name=a-HuIgG_0.03	feature_id[14].value <= threshold=19665.666015625
node_93: feature_name=SARS.CoV.2.Spike.RBD.His.HEK	feature_id[4].value <= threshold=3.0166666507720947
node_94: feature_name=SARS.CoV.2.Spike.RBD.His.Bac	feature_id[3].value > threshold=-51.85277557373047
node_100: feature_name=SARS.CoV.2.S1.mFcTag	feature_id[0].value > threshold=488.56666564941406
Class: healthcare workers within 60 days after vaccination	
Rules_184	passed counts:1
node_0: feature_name=HuIgG_0.10	feature_id[9].value > threshold=19120.166015625
node_92: feature_name=a-HuIgG_0.03	feature_id[14].value <= threshold=19665.666015625
node_93: feature_name=SARS.CoV.2.Spike.RBD.His.HEK	feature_id[4].value <= threshold=3.0166666507720947
node_94: feature_name=SARS.CoV.2.Spike.RBD.His.Bac	feature_id[3].value > threshold=-51.85277557373047
node_100: feature_name=SARS.CoV.2.S1.mFcTag	feature_id[0].value <= threshold=488.56666564941406
node_101: feature_name=a-HuIgG_0.30	feature_id[11].value <= threshold=34723.5
Class: healthcare workers within 60 days after vaccination	
Rules_185	passed counts:1
node_0: feature_name=HuIgG_0.10	feature_id[9].value > threshold=19120.166015625
node_92: feature_name=a-HuIgG_0.03	feature_id[14].value <= threshold=19665.666015625
node_93: feature_name=SARS.CoV.2.Spike.RBD.His.HEK	feature_id[4].value <= threshold=3.0166666507720947
node_94: feature_name=SARS.CoV.2.Spike.RBD.His.Bac	feature_id[3].value <= threshold=-51.85277557373047
node_95: feature_name=SARS.CoV.2.S1.RBD.mFc	feature_id[1].value > threshold=-141.8333387374878
node_97: feature_name=a-HuIgG_0.30	feature_id[11].value > threshold=1290.8333435058594
Class: healthcare workers between 60 and 180 days after vaccination	
Rules_186	passed counts:1

node_0: feature_name=HuIgG_0.10	feature_id[9].value > threshold=19120.166015625
node_92: feature_name=a-HuIgG_0.03	feature_id[14].value <= threshold=19665.666015625
node_93: feature_name=SARS.CoV.2.Spike.RBD.His.HEK	feature_id[4].value <= threshold=3.0166666507720947
node_94: feature_name=SARS.CoV.2.Spike.RBD.His.Bac	feature_id[3].value <= threshold=-51.85277557373047
node_95: feature_name=SARS.CoV.2.S1.RBD.mFc	feature_id[1].value > threshold=-141.8333387374878
node_97: feature_name=a-HuIgG_0.30	feature_id[11].value <= threshold=1290.8333435058594
Class: healthcare workers over 180 days after vaccination	
Rules_187	passed counts:1
node_0: feature_name=HuIgG_0.10	feature_id[9].value <= threshold=19120.166015625
node_1: feature_name=SARS.CoV.2.S1.RBD.mFc	feature_id[1].value > threshold=3773.8334350585938
node_9: feature_name=a-HuIgG_0.30	feature_id[11].value > threshold=33726.833984375
node_47: feature_name=SARS.CoV.2.S1.mFcTag	feature_id[0].value > threshold=43185.68359375
node_57: feature_name=a-HuIgG_0.03	feature_id[14].value > threshold=10858.0
node_71: feature_name=HuIgG_0.03	feature_id[8].value > threshold=3796.0
node_83: feature_name=SARS.CoV.2.S1+S2	feature_id[7].value > threshold=49967.91796875
node_87: feature_name=HuIgG_0.10	feature_id[9].value > threshold=18981.666015625
node_89: feature_name=HuIgG_0.30	feature_id[12].value > threshold=38480.5
Class: healthcare workers over 180 days after vaccination	
Rules_188	passed counts:1
node_0: feature_name=HuIgG_0.10	feature_id[9].value <= threshold=19120.166015625
node_1: feature_name=SARS.CoV.2.S1.RBD.mFc	feature_id[1].value > threshold=3773.8334350585938
node_9: feature_name=a-HuIgG_0.30	feature_id[11].value > threshold=33726.833984375
node_47: feature_name=SARS.CoV.2.S1.mFcTag	feature_id[0].value > threshold=43185.68359375

node_57: feature_name=a-HuIgG_0.03	feature_id[14].value > threshold=10858.0
node_71: feature_name=HuIgG_0.03	feature_id[8].value > threshold=3796.0
node_83: feature_name=SARS.CoV.2.S1+S2	feature_id[7].value > threshold=49967.91796875
node_87: feature_name=HuIgG_0.10	feature_id[9].value > threshold=18981.666015625
node_89: feature_name=HuIgG_0.30	feature_id[12].value <= threshold=38480.5
Class: healthcare workers between 60 and 180 days after vaccination	
Rules_189	passed counts:1
node_0: feature_name=HuIgG_0.10	feature_id[9].value <= threshold=19120.166015625
node_1: feature_name=SARS.CoV.2.S1.RBD.mFc	feature_id[1].value > threshold=3773.8334350585938
node_9: feature_name=a-HuIgG_0.30	feature_id[11].value > threshold=33726.833984375
node_47: feature_name=SARS.CoV.2.S1.mFcTag	feature_id[0].value > threshold=43185.68359375
node_57: feature_name=a-HuIgG_0.03	feature_id[14].value > threshold=10858.0
node_71: feature_name=HuIgG_0.03	feature_id[8].value > threshold=3796.0
node_83: feature_name=SARS.CoV.2.S1+S2	feature_id[7].value <= threshold=49967.91796875
node_84: feature_name=HuIgG_0.10	feature_id[9].value <= threshold=17143.833984375
Class: healthcare workers within 60 days after vaccination	
Rules_190	passed counts:1
node_0: feature_name=HuIgG_0.10	feature_id[9].value <= threshold=19120.166015625
node_1: feature_name=SARS.CoV.2.S1.RBD.mFc	feature_id[1].value > threshold=3773.8334350585938
node_9: feature_name=a-HuIgG_0.30	feature_id[11].value > threshold=33726.833984375
node_47: feature_name=SARS.CoV.2.S1.mFcTag	feature_id[0].value > threshold=43185.68359375
node_57: feature_name=a-HuIgG_0.03	feature_id[14].value <= threshold=10858.0
node_58: feature_name=HuIgG_0.30	feature_id[12].value <= threshold=36767.33203125

node_59: feature_name=a-HuIgG_0.10	feature_id[13].value > threshold=21868.0
node_61: feature_name=a-HuIgG_0.03	feature_id[14].value > threshold=10179.0
node_65: feature_name=SARS.CoV.2.S1	feature_id[6].value > threshold=34029.666015625
node_67: feature_name=HuIgG_0.10	feature_id[9].value > threshold=18956.5
Class: healthcare workers within 60 days after vaccination	
Rules_191	passed counts:1
node_0: feature_name=HuIgG_0.10	feature_id[9].value <= threshold=19120.166015625
node_1: feature_name=SARS.CoV.2.S1.RBD.mFc	feature_id[1].value > threshold=3773.8334350585938
node_9: feature_name=a-HuIgG_0.30	feature_id[11].value > threshold=33726.833984375
node_47: feature_name=SARS.CoV.2.S1.mFcTag	feature_id[0].value > threshold=43185.68359375
node_57: feature_name=a-HuIgG_0.03	feature_id[14].value <= threshold=10858.0
node_58: feature_name=HuIgG_0.30	feature_id[12].value <= threshold=36767.33203125
node_59: feature_name=a-HuIgG_0.10	feature_id[13].value > threshold=21868.0
node_61: feature_name=a-HuIgG_0.03	feature_id[14].value <= threshold=10179.0
node_62: feature_name=a-HuIgG_0.30	feature_id[11].value <= threshold=34348.833984375
Class: healthcare workers between 60 and 180 days after vaccination	
Rules_192	passed counts:1
node_0: feature_name=HuIgG_0.10	feature_id[9].value <= threshold=19120.166015625
node_1: feature_name=SARS.CoV.2.S1.RBD.mFc	feature_id[1].value > threshold=3773.8334350585938
node_9: feature_name=a-HuIgG_0.30	feature_id[11].value > threshold=33726.833984375
node_47: feature_name=SARS.CoV.2.S1.mFcTag	feature_id[0].value <= threshold=43185.68359375
node_48: feature_name=SARS.CoV.S1.RBD.HisTag	feature_id[10].value > threshold=3416.129638671875
node_52: feature_name=SARS.CoV.2.S1.HisTag	feature_id[2].value <= threshold=7094.0

node_53: feature_name=a-HuIgG_0.30	feature_id[11].value > threshold=42229.833984375
Class: healthcare workers between 60 and 180 days after vaccination	
Rules_193	passed counts:1
node_0: feature_name=HuIgG_0.10	feature_id[9].value <= threshold=19120.166015625
node_1: feature_name=SARS.CoV.2.S1.RBD.mFc	feature_id[1].value > threshold=3773.8334350585938
node_9: feature_name=a-HuIgG_0.30	feature_id[11].value > threshold=33726.833984375
node_47: feature_name=SARS.CoV.2.S1.mFcTag	feature_id[0].value <= threshold=43185.68359375
node_48: feature_name=SARS.CoV.S1.RBD.HisTag	feature_id[10].value <= threshold=3416.129638671875
node_49: feature_name=SARS.CoV.2.S1.HisTag	feature_id[2].value <= threshold=4582.9832763671875
Class: healthcare workers between 60 and 180 days after vaccination	
Rules_194	passed counts:1
node_0: feature_name=HuIgG_0.10	feature_id[9].value <= threshold=19120.166015625
node_1: feature_name=SARS.CoV.2.S1.RBD.mFc	feature_id[1].value > threshold=3773.8334350585938
node_9: feature_name=a-HuIgG_0.30	feature_id[11].value <= threshold=33726.833984375
node_10: feature_name=HuIgG_0.03	feature_id[8].value > threshold=1023.1666564941406
node_20: feature_name=SARS.CoV.S1.HisTag	feature_id[15].value > threshold=400.3000030517578
node_24: feature_name=SARS.CoV.2.Spike.RBD.His.Bac	feature_id[3].value <= threshold=36933.69921875
node_25: feature_name=SARS.CoV.2.S1.RBD.mFc	feature_id[1].value <= threshold=40856.734375
node_26: feature_name=SARS.CoV.S1.HisTag	feature_id[15].value > threshold=15087.41650390625
node_34: feature_name=SARS.CoV.S1.HisTag	feature_id[15].value > threshold=15476.41650390625
node_36: feature_name=a-MoIgG_0.10	feature_id[16].value > threshold=4103.6666259765625
node_38: feature_name=SARS.CoV.2.Spike.RBD.His.HEK	feature_id[4].value > threshold=22606.25048828125
Class: healthcare workers between 60 and 180 days after vaccination	

Rules_195	passed counts:1
node_0: feature_name=HuIgG_0.10	feature_id[9].value <= threshold=19120.166015625
node_1: feature_name=SARS.CoV.2.S1.RBD.mFc	feature_id[1].value > threshold=3773.8334350585938
node_9: feature_name=a-HuIgG_0.30	feature_id[11].value <= threshold=33726.833984375
node_10: feature_name=HuIgG_0.03	feature_id[8].value > threshold=1023.1666564941406
node_20: feature_name=SARS.CoV.S1.HisTag	feature_id[15].value > threshold=400.3000030517578
node_24: feature_name=SARS.CoV.2.Spike.RBD.His.Bac	feature_id[3].value <= threshold=36933.69921875
node_25: feature_name=SARS.CoV.2.S1.RBD.mFc	feature_id[1].value <= threshold=40856.734375
node_26: feature_name=SARS.CoV.S1.HisTag	feature_id[15].value > threshold=15087.41650390625
node_34: feature_name=SARS.CoV.S1.HisTag	feature_id[15].value > threshold=15476.41650390625
node_36: feature_name=a-MoIgG_0.10	feature_id[16].value > threshold=4103.6666259765625
node_38: feature_name=SARS.CoV.2.Spike.RBD.His.HEK	feature_id[4].value <= threshold=22606.25048828125
Class: healthcare workers within 60 days after vaccination	
Rules_196	passed counts:1
node_0: feature_name=HuIgG_0.10	feature_id[9].value <= threshold=19120.166015625
node_1: feature_name=SARS.CoV.2.S1.RBD.mFc	feature_id[1].value > threshold=3773.8334350585938
node_9: feature_name=a-HuIgG_0.30	feature_id[11].value <= threshold=33726.833984375
node_10: feature_name=HuIgG_0.03	feature_id[8].value > threshold=1023.1666564941406
node_20: feature_name=SARS.CoV.S1.HisTag	feature_id[15].value > threshold=400.3000030517578
node_24: feature_name=SARS.CoV.2.Spike.RBD.His.Bac	feature_id[3].value <= threshold=36933.69921875
node_25: feature_name=SARS.CoV.2.S1.RBD.mFc	feature_id[1].value <= threshold=40856.734375
node_26: feature_name=SARS.CoV.S1.HisTag	feature_id[15].value > threshold=15087.41650390625
node_34: feature_name=SARS.CoV.S1.HisTag	feature_id[15].value <= threshold=15476.41650390625

Class: healthcare workers within 60 days after vaccination	
Rules_197	passed counts:1
node_0: feature_name=HuIgG_0.10	feature_id[9].value <= threshold=19120.166015625
node_1: feature_name=SARS.CoV.2.S1.RBD.mFc	feature_id[1].value > threshold=3773.8334350585938
node_9: feature_name=a-HuIgG_0.30	feature_id[11].value <= threshold=33726.833984375
node_10: feature_name=HuIgG_0.03	feature_id[8].value > threshold=1023.1666564941406
node_20: feature_name=SARS.CoV.S1.HisTag	feature_id[15].value > threshold=400.3000030517578
node_24: feature_name=SARS.CoV.2.Spike.RBD.His.Bac	feature_id[3].value <= threshold=36933.69921875
node_25: feature_name=SARS.CoV.2.S1.RBD.mFc	feature_id[1].value <= threshold=40856.734375
node_26: feature_name=SARS.CoV.S1.HisTag	feature_id[15].value <= threshold=15087.41650390625
node_27: feature_name=SARS.CoV.2.Spike.RBD.His.HEK	feature_id[4].value <= threshold=10178.375
node_28: feature_name=SARS.CoV.2.S1.RBD.mFc	feature_id[1].value <= threshold=33458.650390625
node_29: feature_name=SARS.CoV.2.Spike.RBD.His.HEK	feature_id[4].value > threshold=10129.44140625
Class: healthcare workers over 180 days after vaccination	
Rules_198	passed counts:1
node_0: feature_name=HuIgG_0.10	feature_id[9].value <= threshold=19120.166015625
node_1: feature_name=SARS.CoV.2.S1.RBD.mFc	feature_id[1].value > threshold=3773.8334350585938
node_9: feature_name=a-HuIgG_0.30	feature_id[11].value <= threshold=33726.833984375
node_10: feature_name=HuIgG_0.03	feature_id[8].value > threshold=1023.1666564941406
node_20: feature_name=SARS.CoV.S1.HisTag	feature_id[15].value <= threshold=400.3000030517578
node_21: feature_name=SARS.CoV.2.Spike.RBD.His.HEK	feature_id[4].value <= threshold=2291.9304809570312
Class: healthcare workers between 60 and 180 days after vaccination	

Rules_199	passed counts:1
node_0: feature_name=HuIgG_0.10	feature_id[9].value <= threshold=19120.166015625
node_1: feature_name=SARS.CoV.2.S1.RBD.mFc	feature_id[1].value > threshold=3773.8334350585938
node_9: feature_name=a-HuIgG_0.30	feature_id[11].value <= threshold=33726.833984375
node_10: feature_name=HuIgG_0.03	feature_id[8].value <= threshold=1023.1666564941406
node_11: feature_name=SARS.CoV.2.S1.mFcTag	feature_id[0].value <= threshold=34194.916015625
node_12: feature_name=SARS.CoV.2.S1+S2	feature_id[7].value > threshold=26709.75
node_14: feature_name=SARS.CoV.2.Spike.RBD.His.HEK	feature_id[4].value <= threshold=8029.208251953125
Class: healthcare workers between 60 and 180 days after vaccination	
Rules_200	passed counts:1
node_0: feature_name=HuIgG_0.10	feature_id[9].value <= threshold=19120.166015625
node_1: feature_name=SARS.CoV.2.S1.RBD.mFc	feature_id[1].value <= threshold=3773.8334350585938
node_2: feature_name=SARS.CoV.2.S1.HisTag	feature_id[2].value > threshold=-383.86944580078125
node_4: feature_name=SARS.CoV.2.S1	feature_id[6].value <= threshold=435.629638671875
node_5: feature_name=SARS.CoV.2.S1	feature_id[6].value > threshold=410.7962951660156
Class: healthcare workers within 60 days after vaccination	
Rules_201	passed counts:1
node_0: feature_name=HuIgG_0.10	feature_id[9].value <= threshold=19120.166015625
node_1: feature_name=SARS.CoV.2.S1.RBD.mFc	feature_id[1].value <= threshold=3773.8334350585938
node_2: feature_name=SARS.CoV.2.S1.HisTag	feature_id[2].value <= threshold=-383.86944580078125
Class: healthcare workers between 60 and 180 days after vaccination	

(4) Rules on the mRMR feature list

Rules_0	passed counts:168
node_0: feature_name=HuIgG_0.10	feature_id[1].value <= threshold=19120.166015625
node_1: feature_name=SARS.CoV.2.S1.mFcTag	feature_id[3].value > threshold=5354.388916015625
node_9: feature_name=a-HuIgG_0.30	feature_id[4].value <= threshold=33726.833984375
node_10: feature_name=HuIgG_0.03	feature_id[5].value > threshold=1023.1666564941406
node_20: feature_name=hCoV.HKU1.NP	feature_id[10].value > threshold=-20.05833387374878
node_22: feature_name=SARS.CoV.S1.HisTag	feature_id[6].value > threshold=400.3000030517578
node_26: feature_name=SARS.CoV.S1.HisTag	feature_id[6].value <= threshold=15087.41650390625
node_27: feature_name=SARS.CoV.2.S1.RBD.mFc	feature_id[0].value <= threshold=43991.083984375
node_28: feature_name=SARS.CoV.2.S1.RBD.mFc	feature_id[0].value <= threshold=33656.484375
Class: healthcare workers between 60 and 180 days after vaccination	
Rules_1	passed counts:75
node_0: feature_name=HuIgG_0.10	feature_id[1].value <= threshold=19120.166015625
node_1: feature_name=SARS.CoV.2.S1.mFcTag	feature_id[3].value <= threshold=5354.388916015625
node_2: feature_name=SARS.CoV.S1.HisTag	feature_id[6].value > threshold=-423.2833251953125
node_4: feature_name=SARS.CoV.2.S2	feature_id[2].value > threshold=566.3889007568359
Class: unvaccinated healthcare workers	
Rules_2	passed counts:71
node_0: feature_name=HuIgG_0.10	feature_id[1].value <= threshold=19120.166015625
node_1: feature_name=SARS.CoV.2.S1.mFcTag	feature_id[3].value > threshold=5354.388916015625
node_9: feature_name=a-HuIgG_0.30	feature_id[4].value <= threshold=33726.833984375
node_10: feature_name=HuIgG_0.03	feature_id[5].value > threshold=1023.1666564941406
node_20: feature_name=hCoV.HKU1.NP	feature_id[10].value > threshold=-20.05833387374878

node_22: feature_name=SARS.CoV.S1.HisTag	feature_id[6].value > threshold=400.3000030517578
node_26: feature_name=SARS.CoV.S1.HisTag	feature_id[6].value <= threshold=15087.41650390625
node_27: feature_name=SARS.CoV.2.S1.RBD.mFc	feature_id[0].value <= threshold=43991.083984375
node_28: feature_name=SARS.CoV.2.S1.RBD.mFc	feature_id[0].value > threshold=33656.484375
node_30: feature_name=SARS.CoV.2.S1+S2	feature_id[12].value > threshold=30126.75
Class: healthcare workers between 60 and 180 days after vaccination	
Rules_3	passed counts:59
node_0: feature_name=HuIgG_0.10	feature_id[1].value > threshold=19120.166015625
node_112: feature_name=a-HuIgG_0.03	feature_id[8].value <= threshold=19665.666015625
node_113: feature_name=SARS.CoV.2.S1.mFcTag	feature_id[3].value <= threshold=54010.166015625
node_114: feature_name=SARS.CoV.2.S1+S2	feature_id[12].value > threshold=47258.43359375
node_240: feature_name=HuIgG_0.03	feature_id[5].value > threshold=5455.5
node_266: feature_name=HuIgG_0.10	feature_id[1].value > threshold=24231.0
node_284: feature_name=HuIgG_0.03	feature_id[5].value > threshold=5469.0
node_286: feature_name=HuIgG_0.10	feature_id[1].value <= threshold=30107.666015625
node_287: feature_name=SARS.CoV.2.S1+S2	feature_id[12].value > threshold=47536.224609375
node_291: feature_name=SARS.CoV.2.S2	feature_id[2].value > threshold=37653.75
node_329: feature_name=SARS.CoV.2.S1.mFcTag	feature_id[3].value <= threshold=53346.333984375
node_330: feature_name=Flu.B_Phu/.HA1+HA2	feature_id[11].value <= threshold=53733.0
node_331: feature_name=MoIgA_0.10	feature_id[13].value > threshold=10.833333015441895
node_335: feature_name=a-HuIgG_0.03	feature_id[8].value > threshold=11890.0
Class: healthcare workers within 60 days after vaccination	
Rules_4	passed counts:56

node_0: feature_name=HuIgG_0.10	feature_id[1].value > threshold=19120.166015625
node_112: feature_name=a-HuIgG_0.03	feature_id[8].value <= threshold=19665.666015625
node_113: feature_name=SARS.CoV.2.S1.mFcTag	feature_id[3].value <= threshold=54010.166015625
node_114: feature_name=SARS.CoV.2.S1+S2	feature_id[12].value > threshold=47258.43359375
node_240: feature_name=HuIgG_0.03	feature_id[5].value <= threshold=5455.5
node_241: feature_name=SARS.CoV.S1.HisTag	feature_id[6].value > threshold=745.7222290039062
node_243: feature_name=SARS.CoV.2.S1+S2	feature_id[12].value > threshold=48812.91796875
node_253: feature_name=SARS.CoV.S1.HisTag	feature_id[6].value > threshold=2772.300048828125
node_257: feature_name=HuIgG_0.10	feature_id[1].value <= threshold=26991.8330078125
node_258: feature_name=SARS.CoV.2.S1.RBD.mFc	feature_id[0].value > threshold=41406.666015625
Class: healthcare workers within 60 days after vaccination	
Rules_5	passed counts:39
node_0: feature_name=HuIgG_0.10	feature_id[1].value > threshold=19120.166015625
node_112: feature_name=a-HuIgG_0.03	feature_id[8].value <= threshold=19665.666015625
node_113: feature_name=SARS.CoV.2.S1.mFcTag	feature_id[3].value <= threshold=54010.166015625
node_114: feature_name=SARS.CoV.2.S1+S2	feature_id[12].value <= threshold=47258.43359375
node_115: feature_name=SARS.CoV.2.S1.RBD.mFc	feature_id[0].value > threshold=301.73333740234375
node_131: feature_name=a-HuIgG_0.03	feature_id[8].value <= threshold=12099.83349609375
node_132: feature_name=SARS.CoV.2.S2	feature_id[2].value > threshold=8858.0751953125
node_134: feature_name=Flu.B_Phu/.HA1+HA2	feature_id[11].value <= threshold=53839.16796875
node_135: feature_name=hCoV.HKU1.NP	feature_id[10].value > threshold=1010.1666564941406
node_145: feature_name=hCoV.HKU1.NP	feature_id[10].value <= threshold=15374.16650390625
node_146: feature_name=SARS.CoV.2.S1.mFcTag	feature_id[3].value <= threshold=50683.0
node_147: feature_name=SARS.CoV.2.Spike.RBD.rFc	feature_id[9].value <= threshold=45822.583984375

node_148: feature_name=SARS.CoV.2.S2	feature_id[2].value > threshold=10212.125
node_154: feature_name=Flu.B_Phu/.HA1+HA2	feature_id[11].value > threshold=49488.1171875
node_162: feature_name=MoIgA_0.10	feature_id[13].value <= threshold=2877.1666259765625
Class: healthcare workers within 60 days after vaccination	
Rules_6	passed counts:31
node_0: feature_name=HuIgG_0.10	feature_id[1].value > threshold=19120.166015625
node_112: feature_name=a-HuIgG_0.03	feature_id[8].value <= threshold=19665.666015625
node_113: feature_name=SARS.CoV.2.S1.mFcTag	feature_id[3].value <= threshold=54010.166015625
node_114: feature_name=SARS.CoV.2.S1+S2	feature_id[12].value > threshold=47258.43359375
node_240: feature_name=HuIgG_0.03	feature_id[5].value > threshold=5455.5
node_266: feature_name=HuIgG_0.10	feature_id[1].value > threshold=24231.0
node_284: feature_name=HuIgG_0.03	feature_id[5].value > threshold=5469.0
node_286: feature_name=HuIgG_0.10	feature_id[1].value <= threshold=30107.666015625
node_287: feature_name=SARS.CoV.2.S1+S2	feature_id[12].value > threshold=47536.224609375
node_291: feature_name=SARS.CoV.2.S2	feature_id[2].value <= threshold=37653.75
node_292: feature_name=SARS.CoV.2.S2	feature_id[2].value <= threshold=36044.29296875
node_293: feature_name=HuIgG_0.03	feature_id[5].value <= threshold=6329.5
node_294: feature_name=Flu.B_Phu/.HA1+HA2	feature_id[11].value > threshold=50410.16796875
node_296: feature_name=HuIgG_0.03	feature_id[5].value > threshold=5639.0
Class: healthcare workers within 60 days after vaccination	
Rules_7	passed counts:28
node_0: feature_name=HuIgG_0.10	feature_id[1].value <= threshold=19120.166015625
node_1: feature_name=SARS.CoV.2.S1.mFcTag	feature_id[3].value > threshold=5354.388916015625

node_9: feature_name=a-HuIgG_0.30	feature_id[4].value <= threshold=33726.833984375
node_10: feature_name=HuIgG_0.03	feature_id[5].value <= threshold=1023.1666564941406
node_11: feature_name=SARS.CoV.2.S1.mFcTag	feature_id[3].value <= threshold=34194.916015625
node_12: feature_name=hCoV.NL63.S1_S2	feature_id[7].value <= threshold=23953.708984375
Class: healthcare workers over 180 days after vaccination	
Rules_8	passed counts:23
node_0: feature_name=HuIgG_0.10	feature_id[1].value > threshold=19120.166015625
node_112: feature_name=a-HuIgG_0.03	feature_id[8].value <= threshold=19665.666015625
node_113: feature_name=SARS.CoV.2.S1.mFcTag	feature_id[3].value > threshold=54010.166015625
node_351: feature_name=SARS.CoV.2.S2	feature_id[2].value <= threshold=37075.75
node_352: feature_name=a-HuIgG_0.03	feature_id[8].value <= threshold=17721.1669921875
node_353: feature_name=MoIgA_0.10	feature_id[13].value > threshold=536.0
node_367: feature_name=HuIgG_0.03	feature_id[5].value > threshold=5634.333251953125
node_381: feature_name=MoIgA_0.10	feature_id[13].value <= threshold=15050.5
Class: healthcare workers between 60 and 180 days after vaccination	
Rules_9	passed counts:23
node_0: feature_name=HuIgG_0.10	feature_id[1].value > threshold=19120.166015625
node_112: feature_name=a-HuIgG_0.03	feature_id[8].value <= threshold=19665.666015625
node_113: feature_name=SARS.CoV.2.S1.mFcTag	feature_id[3].value <= threshold=54010.166015625
node_114: feature_name=SARS.CoV.2.S1+S2	feature_id[12].value > threshold=47258.43359375
node_240: feature_name=HuIgG_0.03	feature_id[5].value > threshold=5455.5
node_266: feature_name=HuIgG_0.10	feature_id[1].value > threshold=24231.0
node_284: feature_name=HuIgG_0.03	feature_id[5].value > threshold=5469.0

node_286: feature_name=HuIgG_0.10	feature_id[1].value <= threshold=30107.666015625
node_287: feature_name=SARS.CoV.2.S1+S2	feature_id[12].value > threshold=47536.224609375
node_291: feature_name=SARS.CoV.2.S2	feature_id[2].value <= threshold=37653.75
node_292: feature_name=SARS.CoV.2.S2	feature_id[2].value <= threshold=36044.29296875
node_293: feature_name=HuIgG_0.03	feature_id[5].value > threshold=6329.5
node_301: feature_name=HuIgG_0.03	feature_id[5].value > threshold=6562.16650390625
node_307: feature_name=Flu.B_Phu/.HA1+HA2	feature_id[11].value <= threshold=54123.5
node_308: feature_name=MoIgA_0.10	feature_id[13].value > threshold=21.166666984558105
node_310: feature_name=hCoV.NL63.S1_S2	feature_id[7].value > threshold=33291.458984375
node_314: feature_name=SARS.CoV.2.S1.mFcTag	feature_id[3].value > threshold=50604.0
Class: healthcare workers within 60 days after vaccination	
Rules_10	passed counts:21
node_0: feature_name=HuIgG_0.10	feature_id[1].value > threshold=19120.166015625
node_112: feature_name=a-HuIgG_0.03	feature_id[8].value > threshold=19665.666015625
node_428: feature_name=SARS.CoV.2.Spike.RBD.rFc	feature_id[9].value <= threshold=55147.1484375
node_429: feature_name=a-HuIgG_0.30	feature_id[4].value > threshold=51289.166015625
node_437: feature_name=HuIgG_0.10	feature_id[1].value <= threshold=25959.0
node_438: feature_name=HuIgG_0.03	feature_id[5].value <= threshold=7564.833251953125
Class: healthcare workers over 180 days after vaccination	
Rules_11	passed counts:21
node_0: feature_name=HuIgG_0.10	feature_id[1].value <= threshold=19120.166015625
node_1: feature_name=SARS.CoV.2.S1.mFcTag	feature_id[3].value > threshold=5354.388916015625
node_9: feature_name=a-HuIgG_0.30	feature_id[4].value > threshold=33726.833984375

node_55: feature_name=SARS.CoV.2.S1.mFcTag	feature_id[3].value <= threshold=43185.68359375
node_56: feature_name=SARS.CoV.S1.HisTag	feature_id[6].value > threshold=1493.4166870117188
node_62: feature_name=SARS.CoV.2.S1+S2	feature_id[12].value > threshold=22078.1669921875
Class: healthcare workers over 180 days after vaccination	
Rules_12	passed counts:20
node_0: feature_name=HuIgG_0.10	feature_id[1].value > threshold=19120.166015625
node_112: feature_name=a-HuIgG_0.03	feature_id[8].value <= threshold=19665.666015625
node_113: feature_name=SARS.CoV.2.S1.mFcTag	feature_id[3].value <= threshold=54010.166015625
node_114: feature_name=SARS.CoV.2.S1+S2	feature_id[12].value <= threshold=47258.43359375
node_115: feature_name=SARS.CoV.2.S1.RBD.mFc	feature_id[0].value > threshold=301.73333740234375
node_131: feature_name=a-HuIgG_0.03	feature_id[8].value > threshold=12099.83349609375
node_183: feature_name=Flu.B_Phu/.HA1+HA2	feature_id[11].value > threshold=41918.533203125
node_187: feature_name=SARS.CoV.2.Spike.RBD.rFc	feature_id[9].value > threshold=27438.341796875
node_197: feature_name=SARS.CoV.2.S2	feature_id[2].value <= threshold=35177.640625
node_198: feature_name=hCoV.HKU1.NP	feature_id[10].value <= threshold=2645.916748046875
node_199: feature_name=hCoV.HKU1.NP	feature_id[10].value > threshold=666.6666870117188
node_203: feature_name=HuIgG_0.10	feature_id[1].value <= threshold=24756.5
Class: healthcare workers between 60 and 180 days after vaccination	
Rules_13	passed counts:18
node_0: feature_name=HuIgG_0.10	feature_id[1].value > threshold=19120.166015625
node_112: feature_name=a-HuIgG_0.03	feature_id[8].value <= threshold=19665.666015625
node_113: feature_name=SARS.CoV.2.S1.mFcTag	feature_id[3].value > threshold=54010.166015625
node_351: feature_name=SARS.CoV.2.S2	feature_id[2].value > threshold=37075.75

node_391: feature_name=SARS.CoV.2.S1.mFcTag	feature_id[3].value <= threshold=55462.833984375
node_392: feature_name=SARS.CoV.2.S1.mFcTag	feature_id[3].value <= threshold=55163.634765625
node_393: feature_name=hCoV.HKU1.NP	feature_id[10].value <= threshold=14561.3330078125
node_394: feature_name=hCoV.HKU1.NP	feature_id[10].value <= threshold=6453.333251953125
node_395: feature_name=hCoV.HKU1.NP	feature_id[10].value > threshold=1990.1666259765625
node_407: feature_name=SARS.CoV.2.S1.RBD.mFc	feature_id[0].value > threshold=48385.75
node_409: feature_name=MoIgA_0.10	feature_id[13].value <= threshold=21414.99951171875
Class: healthcare workers within 60 days after vaccination	
Rules_14	passed counts:17
node_0: feature_name=HuIgG_0.10	feature_id[1].value > threshold=19120.166015625
node_112: feature_name=a-HuIgG_0.03	feature_id[8].value <= threshold=19665.666015625
node_113: feature_name=SARS.CoV.2.S1.mFcTag	feature_id[3].value <= threshold=54010.166015625
node_114: feature_name=SARS.CoV.2.S1+S2	feature_id[12].value <= threshold=47258.43359375
node_115: feature_name=SARS.CoV.2.S1.RBD.mFc	feature_id[0].value > threshold=301.73333740234375
node_131: feature_name=a-HuIgG_0.03	feature_id[8].value <= threshold=12099.83349609375
node_132: feature_name=SARS.CoV.2.S2	feature_id[2].value > threshold=8858.0751953125
node_134: feature_name=Flu.B_Phu/.HA1+HA2	feature_id[11].value <= threshold=53839.16796875
node_135: feature_name=hCoV.HKU1.NP	feature_id[10].value > threshold=1010.1666564941406
node_145: feature_name=hCoV.HKU1.NP	feature_id[10].value <= threshold=15374.16650390625
node_146: feature_name=SARS.CoV.2.S1.mFcTag	feature_id[3].value > threshold=50683.0
node_168: feature_name=HuIgG_0.10	feature_id[1].value > threshold=20669.833984375
node_170: feature_name=SARS.CoV.2.S1.mFcTag	feature_id[3].value > threshold=50874.666015625
node_172: feature_name=SARS.CoV.S1.HisTag	feature_id[6].value > threshold=3796.25
Class: healthcare workers within 60 days after vaccination	

Rules_15	passed counts:15
node_0: feature_name=HuIgG_0.10	feature_id[1].value > threshold=19120.166015625
node_112: feature_name=a-HuIgG_0.03	feature_id[8].value <= threshold=19665.666015625
node_113: feature_name=SARS.CoV.2.S1.mFcTag	feature_id[3].value <= threshold=54010.166015625
node_114: feature_name=SARS.CoV.2.S1+S2	feature_id[12].value > threshold=47258.43359375
node_240: feature_name=HuIgG_0.03	feature_id[5].value <= threshold=5455.5
node_241: feature_name=SARS.CoV.S1.HisTag	feature_id[6].value > threshold=745.7222290039062
node_243: feature_name=SARS.CoV.2.S1+S2	feature_id[12].value <= threshold=48812.91796875
node_244: feature_name=SARS.CoV.2.S2	feature_id[2].value <= threshold=26981.416015625
Class: healthcare workers within 60 days after vaccination	
Rules_16	passed counts:15
node_0: feature_name=HuIgG_0.10	feature_id[1].value > threshold=19120.166015625
node_112: feature_name=a-HuIgG_0.03	feature_id[8].value <= threshold=19665.666015625
node_113: feature_name=SARS.CoV.2.S1.mFcTag	feature_id[3].value <= threshold=54010.166015625
node_114: feature_name=SARS.CoV.2.S1+S2	feature_id[12].value <= threshold=47258.43359375
node_115: feature_name=SARS.CoV.2.S1.RBD.mFc	feature_id[0].value > threshold=301.73333740234375
node_131: feature_name=a-HuIgG_0.03	feature_id[8].value > threshold=12099.83349609375
node_183: feature_name=Flu.B_Phu/.HA1+HA2	feature_id[11].value > threshold=41918.533203125
node_187: feature_name=SARS.CoV.2.Spike.RBD.rFc	feature_id[9].value > threshold=27438.341796875
node_197: feature_name=SARS.CoV.2.S2	feature_id[2].value <= threshold=35177.640625
node_198: feature_name=hCoV.HKU1.NP	feature_id[10].value > threshold=2645.916748046875
node_210: feature_name=Flu.B_Phu/.HA1+HA2	feature_id[11].value <= threshold=54029.166015625
node_211: feature_name=HuIgG_0.10	feature_id[1].value > threshold=21359.5

node_213: feature_name=hCoV.NL63.S1_S2	feature_id[7].value > threshold=32523.4248046875
node_215: feature_name=HuIgG_0.10	feature_id[1].value > threshold=23274.166015625
node_217: feature_name=HuIgG_0.10	feature_id[1].value <= threshold=25231.1669921875
node_218: feature_name=hCoV.HKU1.NP	feature_id[10].value > threshold=3712.083251953125
node_220: feature_name=SARS.CoV.S1.HisTag	feature_id[6].value <= threshold=11696.75
node_221: feature_name=MoIgA_0.10	feature_id[13].value <= threshold=2253.6666259765625
Class: healthcare workers between 60 and 180 days after vaccination	
Rules_17	passed counts:15
node_0: feature_name=HuIgG_0.10	feature_id[1].value <= threshold=19120.166015625
node_1: feature_name=SARS.CoV.2.S1.mFcTag	feature_id[3].value > threshold=5354.388916015625
node_9: feature_name=a-HuIgG_0.30	feature_id[4].value <= threshold=33726.833984375
node_10: feature_name=HuIgG_0.03	feature_id[5].value > threshold=1023.1666564941406
node_20: feature_name=hCoV.HKU1.NP	feature_id[10].value > threshold=-20.05833387374878
node_22: feature_name=SARS.CoV.S1.HisTag	feature_id[6].value > threshold=400.3000030517578
node_26: feature_name=SARS.CoV.S1.HisTag	feature_id[6].value > threshold=15087.41650390625
node_38: feature_name=hCoV.HKU1.NP	feature_id[10].value > threshold=348.6666564941406
node_46: feature_name=SARS.CoV.2.S2	feature_id[2].value > threshold=21127.650390625
node_50: feature_name=a-HuIgG_0.03	feature_id[8].value <= threshold=11226.33349609375
Class: healthcare workers between 60 and 180 days after vaccination	
Rules_18	passed counts:14
node_0: feature_name=HuIgG_0.10	feature_id[1].value > threshold=19120.166015625
node_112: feature_name=a-HuIgG_0.03	feature_id[8].value <= threshold=19665.666015625
node_113: feature_name=SARS.CoV.2.S1.mFcTag	feature_id[3].value > threshold=54010.166015625

node_351: feature_name=SARS.CoV.2.S2	feature_id[2].value > threshold=37075.75
node_391: feature_name=SARS.CoV.2.S1.mFcTag	feature_id[3].value <= threshold=55462.833984375
node_392: feature_name=SARS.CoV.2.S1.mFcTag	feature_id[3].value <= threshold=55163.634765625
node_393: feature_name=hCoV.HKU1.NP	feature_id[10].value <= threshold=14561.3330078125
node_394: feature_name=hCoV.HKU1.NP	feature_id[10].value > threshold=6453.333251953125
node_412: feature_name=HuIgG_0.03	feature_id[5].value > threshold=4766.166748046875
node_414: feature_name=MoIgA_0.10	feature_id[13].value <= threshold=2918.0
Class: healthcare workers between 60 and 180 days after vaccination	
Rules_19	passed counts:14
node_0: feature_name=HuIgG_0.10	feature_id[1].value > threshold=19120.166015625
node_112: feature_name=a-HuIgG_0.03	feature_id[8].value <= threshold=19665.666015625
node_113: feature_name=SARS.CoV.2.S1.mFcTag	feature_id[3].value <= threshold=54010.166015625
node_114: feature_name=SARS.CoV.2.S1+S2	feature_id[12].value <= threshold=47258.43359375
node_115: feature_name=SARS.CoV.2.S1.RBD.mFc	feature_id[0].value > threshold=301.73333740234375
node_131: feature_name=a-HuIgG_0.03	feature_id[8].value > threshold=12099.83349609375
node_183: feature_name=Flu.B_Phu/.HA1+HA2	feature_id[11].value > threshold=41918.533203125
node_187: feature_name=SARS.CoV.2.Spike.RBD.rFc	feature_id[9].value > threshold=27438.341796875
node_197: feature_name=SARS.CoV.2.S2	feature_id[2].value <= threshold=35177.640625
node_198: feature_name=hCoV.HKU1.NP	feature_id[10].value > threshold=2645.916748046875
node_210: feature_name=Flu.B_Phu/.HA1+HA2	feature_id[11].value > threshold=54029.166015625
node_236: feature_name=SARS.CoV.2.S1.RBD.mFc	feature_id[0].value <= threshold=47021.16796875
Class: healthcare workers between 60 and 180 days after vaccination	
Rules_20	passed counts:14

node_0: feature_name=HuIgG_0.10	feature_id[1].value > threshold=19120.166015625
node_112: feature_name=a-HuIgG_0.03	feature_id[8].value <= threshold=19665.666015625
node_113: feature_name=SARS.CoV.2.S1.mFcTag	feature_id[3].value <= threshold=54010.166015625
node_114: feature_name=SARS.CoV.2.S1+S2	feature_id[12].value <= threshold=47258.43359375
node_115: feature_name=SARS.CoV.2.S1.RBD.mFc	feature_id[0].value > threshold=301.73333740234375
node_131: feature_name=a-HuIgG_0.03	feature_id[8].value > threshold=12099.83349609375
node_183: feature_name=Flu.B_Phu/.HA1+HA2	feature_id[11].value > threshold=41918.533203125
node_187: feature_name=SARS.CoV.2.Spike.RBD.rFc	feature_id[9].value > threshold=27438.341796875
node_197: feature_name=SARS.CoV.2.S2	feature_id[2].value <= threshold=35177.640625
node_198: feature_name=hCoV.HKU1.NP	feature_id[10].value > threshold=2645.916748046875
node_210: feature_name=Flu.B_Phu/.HA1+HA2	feature_id[11].value <= threshold=54029.166015625
node_211: feature_name=HuIgG_0.10	feature_id[1].value > threshold=21359.5
node_213: feature_name=hCoV.NL63.S1_S2	feature_id[7].value > threshold=32523.4248046875
node_215: feature_name=HuIgG_0.10	feature_id[1].value > threshold=23274.166015625
node_217: feature_name=HuIgG_0.10	feature_id[1].value > threshold=25231.1669921875
node_227: feature_name=SARS.CoV.2.S2	feature_id[2].value > threshold=18038.125
node_231: feature_name=a-HuIgG_0.03	feature_id[8].value > threshold=12541.1669921875
node_233: feature_name=SARS.CoV.2.S1+S2	feature_id[12].value <= threshold=47171.75
Class: healthcare workers within 60 days after vaccination	
Rules_21	passed counts:13
node_0: feature_name=HuIgG_0.10	feature_id[1].value > threshold=19120.166015625
node_112: feature_name=a-HuIgG_0.03	feature_id[8].value <= threshold=19665.666015625
node_113: feature_name=SARS.CoV.2.S1.mFcTag	feature_id[3].value <= threshold=54010.166015625
node_114: feature_name=SARS.CoV.2.S1+S2	feature_id[12].value <= threshold=47258.43359375

node_115: feature_name=SARS.CoV.2.S1.RBD.mFc	feature_id[0].value > threshold=301.73333740234375
node_131: feature_name=a-HuIgG_0.03	feature_id[8].value <= threshold=12099.83349609375
node_132: feature_name=SARS.CoV.2.S2	feature_id[2].value > threshold=8858.0751953125
node_134: feature_name=Flu.B_Phu/.HA1+HA2	feature_id[11].value <= threshold=53839.16796875
node_135: feature_name=hCoV.HKU1.NP	feature_id[10].value > threshold=1010.1666564941406
node_145: feature_name=hCoV.HKU1.NP	feature_id[10].value <= threshold=15374.16650390625
node_146: feature_name=SARS.CoV.2.S1.mFcTag	feature_id[3].value <= threshold=50683.0
node_147: feature_name=SARS.CoV.2.Spike.RBD.rFc	feature_id[9].value <= threshold=45822.583984375
node_148: feature_name=SARS.CoV.2.S2	feature_id[2].value > threshold=10212.125
node_154: feature_name=Flu.B_Phu/.HA1+HA2	feature_id[11].value <= threshold=49488.1171875
node_155: feature_name=MoIgA_0.10	feature_id[13].value > threshold=177.66666412353516
node_157: feature_name=a-HuIgG_0.03	feature_id[8].value <= threshold=11251.0
Class: healthcare workers within 60 days after vaccination	
Rules_22	passed counts:12
node_0: feature_name=HuIgG_0.10	feature_id[1].value > threshold=19120.166015625
node_112: feature_name=a-HuIgG_0.03	feature_id[8].value <= threshold=19665.666015625
node_113: feature_name=SARS.CoV.2.S1.mFcTag	feature_id[3].value > threshold=54010.166015625
node_351: feature_name=SARS.CoV.2.S2	feature_id[2].value > threshold=37075.75
node_391: feature_name=SARS.CoV.2.S1.mFcTag	feature_id[3].value <= threshold=55462.833984375
node_392: feature_name=SARS.CoV.2.S1.mFcTag	feature_id[3].value > threshold=55163.634765625
node_420: feature_name=SARS.CoV.2.S1+S2	feature_id[12].value <= threshold=54621.0
Class: healthcare workers within 60 days after vaccination	
Rules_23	passed counts:12

node_0: feature_name=HuIgG_0.10	feature_id[1].value > threshold=19120.166015625
node_112: feature_name=a-HuIgG_0.03	feature_id[8].value <= threshold=19665.666015625
node_113: feature_name=SARS.CoV.2.S1.mFcTag	feature_id[3].value <= threshold=54010.166015625
node_114: feature_name=SARS.CoV.2.S1+S2	feature_id[12].value <= threshold=47258.43359375
node_115: feature_name=SARS.CoV.2.S1.RBD.mFc	feature_id[0].value > threshold=301.73333740234375
node_131: feature_name=a-HuIgG_0.03	feature_id[8].value > threshold=12099.83349609375
node_183: feature_name=Flu.B_Phu/.HA1+HA2	feature_id[11].value > threshold=41918.533203125
node_187: feature_name=SARS.CoV.2.Spike.RBD.rFc	feature_id[9].value > threshold=27438.341796875
node_197: feature_name=SARS.CoV.2.S2	feature_id[2].value <= threshold=35177.640625
node_198: feature_name=hCoV.HKU1.NP	feature_id[10].value > threshold=2645.916748046875
node_210: feature_name=Flu.B_Phu/.HA1+HA2	feature_id[11].value <= threshold=54029.166015625
node_211: feature_name=HuIgG_0.10	feature_id[1].value > threshold=21359.5
node_213: feature_name=hCoV.NL63.S1_S2	feature_id[7].value <= threshold=32523.4248046875
Class: healthcare workers within 60 days after vaccination	
Rules_24	passed counts:12
node_0: feature_name=HuIgG_0.10	feature_id[1].value > threshold=19120.166015625
node_112: feature_name=a-HuIgG_0.03	feature_id[8].value <= threshold=19665.666015625
node_113: feature_name=SARS.CoV.2.S1.mFcTag	feature_id[3].value <= threshold=54010.166015625
node_114: feature_name=SARS.CoV.2.S1+S2	feature_id[12].value <= threshold=47258.43359375
node_115: feature_name=SARS.CoV.2.S1.RBD.mFc	feature_id[0].value <= threshold=301.73333740234375
node_116: feature_name=SARS.CoV.2.S1+S2	feature_id[12].value > threshold=3298.25
node_122: feature_name=SARS.CoV.2.S1.mFcTag	feature_id[3].value > threshold=-1493.1666870117188
node_124: feature_name=hCoV.HKU1.NP	feature_id[10].value <= threshold=11908.138671875
node_125: feature_name=SARS.CoV.2.S1.RBD.mFc	feature_id[0].value <= threshold=82.01666641235352

Class: unvaccinated healthcare workers	
Rules_25	passed counts:12
node_0: feature_name=HuIgG_0.10	feature_id[1].value <= threshold=19120.166015625
node_1: feature_name=SARS.CoV.2.S1.mFcTag	feature_id[3].value > threshold=5354.388916015625
node_9: feature_name=a-HuIgG_0.30	feature_id[4].value > threshold=33726.833984375
node_55: feature_name=SARS.CoV.2.S1.mFcTag	feature_id[3].value > threshold=43185.68359375
node_67: feature_name=a-HuIgG_0.03	feature_id[8].value > threshold=10858.0
node_83: feature_name=HuIgG_0.03	feature_id[5].value <= threshold=3796.0
node_84: feature_name=hCoV.NL63.S1_S2	feature_id[7].value > threshold=29814.5
node_96: feature_name=hCoV.NL63.S1_S2	feature_id[7].value <= threshold=34078.0
Class: healthcare workers over 180 days after vaccination	
Rules_26	passed counts:11
node_0: feature_name=HuIgG_0.10	feature_id[1].value > threshold=19120.166015625
node_112: feature_name=a-HuIgG_0.03	feature_id[8].value <= threshold=19665.666015625
node_113: feature_name=SARS.CoV.2.S1.mFcTag	feature_id[3].value <= threshold=54010.166015625
node_114: feature_name=SARS.CoV.2.S1+S2	feature_id[12].value > threshold=47258.43359375
node_240: feature_name=HuIgG_0.03	feature_id[5].value <= threshold=5455.5
node_241: feature_name=SARS.CoV.S1.HisTag	feature_id[6].value > threshold=745.7222290039062
node_243: feature_name=SARS.CoV.2.S1+S2	feature_id[12].value <= threshold=48812.91796875
node_244: feature_name=SARS.CoV.2.S2	feature_id[2].value > threshold=26981.416015625
node_246: feature_name=Flu.B_Phu/.HA1+HA2	feature_id[11].value <= threshold=52223.0
node_247: feature_name=Flu.B_Phu/.HA1+HA2	feature_id[11].value > threshold=47753.833984375
Class: healthcare workers within 60 days after vaccination	

Rules_27	passed counts:11
node_0: feature_name=HuIgG_0.10	feature_id[1].value <= threshold=19120.166015625
node_1: feature_name=SARS.CoV.2.S1.mFcTag	feature_id[3].value > threshold=5354.388916015625
node_9: feature_name=a-HuIgG_0.30	feature_id[4].value > threshold=33726.833984375
node_55: feature_name=SARS.CoV.2.S1.mFcTag	feature_id[3].value > threshold=43185.68359375
node_67: feature_name=a-HuIgG_0.03	feature_id[8].value > threshold=10858.0
node_83: feature_name=HuIgG_0.03	feature_id[5].value > threshold=3796.0
node_103: feature_name=SARS.CoV.2.S1+S2	feature_id[12].value <= threshold=49967.91796875
node_104: feature_name=HuIgG_0.10	feature_id[1].value > threshold=17143.833984375
Class: healthcare workers between 60 and 180 days after vaccination	
Rules_28	passed counts:10
node_0: feature_name=HuIgG_0.10	feature_id[1].value > threshold=19120.166015625
node_112: feature_name=a-HuIgG_0.03	feature_id[8].value <= threshold=19665.666015625
node_113: feature_name=SARS.CoV.2.S1.mFcTag	feature_id[3].value > threshold=54010.166015625
node_351: feature_name=SARS.CoV.2.S2	feature_id[2].value <= threshold=37075.75
node_352: feature_name=a-HuIgG_0.03	feature_id[8].value <= threshold=17721.1669921875
node_353: feature_name=MoIgA_0.10	feature_id[13].value <= threshold=536.0
node_354: feature_name=hCoV.HKU1.NP	feature_id[10].value <= threshold=7098.5
node_355: feature_name=MoIgA_0.10	feature_id[13].value <= threshold=462.0
node_356: feature_name=hCoV.HKU1.NP	feature_id[10].value > threshold=2790.583251953125
Class: healthcare workers between 60 and 180 days after vaccination	
Rules_29	passed counts:10

node_0: feature_name=HuIgG_0.10	feature_id[1].value > threshold=19120.166015625
node_112: feature_name=a-HuIgG_0.03	feature_id[8].value <= threshold=19665.666015625
node_113: feature_name=SARS.CoV.2.S1.mFcTag	feature_id[3].value <= threshold=54010.166015625
node_114: feature_name=SARS.CoV.2.S1+S2	feature_id[12].value <= threshold=47258.43359375
node_115: feature_name=SARS.CoV.2.S1.RBD.mFc	feature_id[0].value > threshold=301.73333740234375
node_131: feature_name=a-HuIgG_0.03	feature_id[8].value > threshold=12099.83349609375
node_183: feature_name=Flu.B_Phu/.HA1+HA2	feature_id[11].value > threshold=41918.533203125
node_187: feature_name=SARS.CoV.2.Spike.RBD.rFc	feature_id[9].value <= threshold=27438.341796875
node_188: feature_name=Flu.B_Phu/.HA1+HA2	feature_id[11].value > threshold=48840.19921875
node_192: feature_name=MoIgA_0.10	feature_id[13].value > threshold=154.99999618530273
Class: healthcare workers within 60 days after vaccination	
Rules_30	passed counts:10
node_0: feature_name=HuIgG_0.10	feature_id[1].value <= threshold=19120.166015625
node_1: feature_name=SARS.CoV.2.S1.mFcTag	feature_id[3].value > threshold=5354.388916015625
node_9: feature_name=a-HuIgG_0.30	feature_id[4].value > threshold=33726.833984375
node_55: feature_name=SARS.CoV.2.S1.mFcTag	feature_id[3].value > threshold=43185.68359375
node_67: feature_name=a-HuIgG_0.03	feature_id[8].value <= threshold=10858.0
node_68: feature_name=MoIgA_0.10	feature_id[13].value > threshold=246.83333587646484
node_76: feature_name=SARS.CoV.2.Spike.RBD.rFc	feature_id[9].value <= threshold=54060.41796875
node_77: feature_name=a-HuIgG_0.03	feature_id[8].value <= threshold=10179.0
Class: healthcare workers within 60 days after vaccination	
Rules_31	passed counts:10
node_0: feature_name=HuIgG_0.10	feature_id[1].value <= threshold=19120.166015625

node_1: feature_name=SARS.CoV.2.S1.mFcTag	feature_id[3].value > threshold=5354.388916015625
node_9: feature_name=a-HuIgG_0.30	feature_id[4].value <= threshold=33726.833984375
node_10: feature_name=HuIgG_0.03	feature_id[5].value <= threshold=1023.1666564941406
node_11: feature_name=SARS.CoV.2.S1.mFcTag	feature_id[3].value > threshold=34194.916015625
node_17: feature_name=HuIgG_0.10	feature_id[1].value <= threshold=7582.83349609375
Class: healthcare workers between 60 and 180 days after vaccination	
Rules_32	passed counts:9
node_0: feature_name=HuIgG_0.10	feature_id[1].value > threshold=19120.166015625
node_112: feature_name=a-HuIgG_0.03	feature_id[8].value <= threshold=19665.666015625
node_113: feature_name=SARS.CoV.2.S1.mFcTag	feature_id[3].value > threshold=54010.166015625
node_351: feature_name=SARS.CoV.2.S2	feature_id[2].value <= threshold=37075.75
node_352: feature_name=a-HuIgG_0.03	feature_id[8].value <= threshold=17721.1669921875
node_353: feature_name=MoIgA_0.10	feature_id[13].value > threshold=536.0
node_367: feature_name=HuIgG_0.03	feature_id[5].value <= threshold=5634.333251953125
node_368: feature_name=SARS.CoV.2.S1+S2	feature_id[12].value <= threshold=52432.75
node_369: feature_name=SARS.CoV.S1.HisTag	feature_id[6].value <= threshold=17773.60009765625
node_370: feature_name=SARS.CoV.2.S2	feature_id[2].value <= threshold=35666.966796875
node_371: feature_name=hCoV.HKU1.NP	feature_id[10].value <= threshold=14106.56640625
Class: healthcare workers between 60 and 180 days after vaccination	
Rules_33	passed counts:9
node_0: feature_name=HuIgG_0.10	feature_id[1].value > threshold=19120.166015625
node_112: feature_name=a-HuIgG_0.03	feature_id[8].value <= threshold=19665.666015625
node_113: feature_name=SARS.CoV.2.S1.mFcTag	feature_id[3].value <= threshold=54010.166015625

node_114: feature_name=SARS.CoV.2.S1+S2	feature_id[12].value <= threshold=47258.43359375
node_115: feature_name=SARS.CoV.2.S1.RBD.mFc	feature_id[0].value > threshold=301.73333740234375
node_131: feature_name=a-HuIgG_0.03	feature_id[8].value > threshold=12099.83349609375
node_183: feature_name=Flu.B_Phu/.HA1+HA2	feature_id[11].value > threshold=41918.533203125
node_187: feature_name=SARS.CoV.2.Spike.RBD.rFc	feature_id[9].value > threshold=27438.341796875
node_197: feature_name=SARS.CoV.2.S2	feature_id[2].value <= threshold=35177.640625
node_198: feature_name=hCoV.HKU1.NP	feature_id[10].value > threshold=2645.916748046875
node_210: feature_name=Flu.B_Phu/.HA1+HA2	feature_id[11].value <= threshold=54029.166015625
node_211: feature_name=HuIgG_0.10	feature_id[1].value > threshold=21359.5
node_213: feature_name=hCoV.NL63.S1_S2	feature_id[7].value > threshold=32523.4248046875
node_215: feature_name=HuIgG_0.10	feature_id[1].value <= threshold=23274.166015625
Class: healthcare workers within 60 days after vaccination	
Rules_34	passed counts:9
node_0: feature_name=HuIgG_0.10	feature_id[1].value <= threshold=19120.166015625
node_1: feature_name=SARS.CoV.2.S1.mFcTag	feature_id[3].value > threshold=5354.388916015625
node_9: feature_name=a-HuIgG_0.30	feature_id[4].value > threshold=33726.833984375
node_55: feature_name=SARS.CoV.2.S1.mFcTag	feature_id[3].value > threshold=43185.68359375
node_67: feature_name=a-HuIgG_0.03	feature_id[8].value > threshold=10858.0
node_83: feature_name=HuIgG_0.03	feature_id[5].value <= threshold=3796.0
node_84: feature_name=hCoV.NL63.S1_S2	feature_id[7].value <= threshold=29814.5
node_85: feature_name=hCoV.NL63.S1_S2	feature_id[7].value > threshold=22280.4169921875
node_89: feature_name=Flu.B_Phu/.HA1+HA2	feature_id[11].value > threshold=41117.93359375
node_91: feature_name=SARS.CoV.S1.HisTag	feature_id[6].value <= threshold=18680.75
Class: healthcare workers between 60 and 180 days after vaccination	

Rules_35	passed counts:8
node_0: feature_name=HuIgG_0.10	feature_id[1].value > threshold=19120.166015625
node_112: feature_name=a-HuIgG_0.03	feature_id[8].value <= threshold=19665.666015625
node_113: feature_name=SARS.CoV.2.S1.mFcTag	feature_id[3].value <= threshold=54010.166015625
node_114: feature_name=SARS.CoV.2.S1+S2	feature_id[12].value > threshold=47258.43359375
node_240: feature_name=HuIgG_0.03	feature_id[5].value > threshold=5455.5
node_266: feature_name=HuIgG_0.10	feature_id[1].value <= threshold=24231.0
node_267: feature_name=hCoV.HKU1.NP	feature_id[10].value > threshold=2123.0000610351562
node_271: feature_name=SARS.CoV.S1.HisTag	feature_id[6].value > threshold=8281.83349609375
node_273: feature_name=Flu.B_Phu/.HA1+HA2	feature_id[11].value > threshold=51424.19921875
node_275: feature_name=a-HuIgG_0.30	feature_id[4].value > threshold=41169.833984375
node_277: feature_name=a-HuIgG_0.30	feature_id[4].value > threshold=42934.333984375
node_279: feature_name=a-HuIgG_0.03	feature_id[8].value <= threshold=17284.5
node_280: feature_name=SARS.CoV.2.S1.mFcTag	feature_id[3].value > threshold=51719.75
Class: healthcare workers within 60 days after vaccination	
Rules_36	passed counts:8
node_0: feature_name=HuIgG_0.10	feature_id[1].value > threshold=19120.166015625
node_112: feature_name=a-HuIgG_0.03	feature_id[8].value <= threshold=19665.666015625
node_113: feature_name=SARS.CoV.2.S1.mFcTag	feature_id[3].value <= threshold=54010.166015625
node_114: feature_name=SARS.CoV.2.S1+S2	feature_id[12].value > threshold=47258.43359375
node_240: feature_name=HuIgG_0.03	feature_id[5].value > threshold=5455.5
node_266: feature_name=HuIgG_0.10	feature_id[1].value <= threshold=24231.0
node_267: feature_name=hCoV.HKU1.NP	feature_id[10].value <= threshold=2123.0000610351562

node_268: feature_name=hCoV.NL63.S1_S2	feature_id[7].value > threshold=25279.30859375
Class: healthcare workers within 60 days after vaccination	
Rules_37	passed counts:8
node_0: feature_name=HuIgG_0.10	feature_id[1].value > threshold=19120.166015625
node_112: feature_name=a-HuIgG_0.03	feature_id[8].value <= threshold=19665.666015625
node_113: feature_name=SARS.CoV.2.S1.mFcTag	feature_id[3].value <= threshold=54010.166015625
node_114: feature_name=SARS.CoV.2.S1+S2	feature_id[12].value <= threshold=47258.43359375
node_115: feature_name=SARS.CoV.2.S1.RBD.mFc	feature_id[0].value > threshold=301.73333740234375
node_131: feature_name=a-HuIgG_0.03	feature_id[8].value > threshold=12099.83349609375
node_183: feature_name=Flu.B_Phu/.HA1+HA2	feature_id[11].value > threshold=41918.533203125
node_187: feature_name=SARS.CoV.2.Spike.RBD.rFc	feature_id[9].value > threshold=27438.341796875
node_197: feature_name=SARS.CoV.2.S2	feature_id[2].value > threshold=35177.640625
Class: healthcare workers within 60 days after vaccination	
Rules_38	passed counts:8
node_0: feature_name=HuIgG_0.10	feature_id[1].value > threshold=19120.166015625
node_112: feature_name=a-HuIgG_0.03	feature_id[8].value <= threshold=19665.666015625
node_113: feature_name=SARS.CoV.2.S1.mFcTag	feature_id[3].value <= threshold=54010.166015625
node_114: feature_name=SARS.CoV.2.S1+S2	feature_id[12].value <= threshold=47258.43359375
node_115: feature_name=SARS.CoV.2.S1.RBD.mFc	feature_id[0].value > threshold=301.73333740234375
node_131: feature_name=a-HuIgG_0.03	feature_id[8].value > threshold=12099.83349609375
node_183: feature_name=Flu.B_Phu/.HA1+HA2	feature_id[11].value > threshold=41918.533203125
node_187: feature_name=SARS.CoV.2.Spike.RBD.rFc	feature_id[9].value > threshold=27438.341796875
node_197: feature_name=SARS.CoV.2.S2	feature_id[2].value <= threshold=35177.640625

node_198: feature_name=hCoV.HKU1.NP	feature_id[10].value <= threshold=2645.916748046875
node_199: feature_name=hCoV.HKU1.NP	feature_id[10].value > threshold=666.6666870117188
node_203: feature_name=HuIgG_0.10	feature_id[1].value > threshold=24756.5
node_205: feature_name=SARS.CoV.2.S1+S2	feature_id[12].value <= threshold=44218.416015625
Class: healthcare workers between 60 and 180 days after vaccination	
Rules_39	passed counts:8
node_0: feature_name=HuIgG_0.10	feature_id[1].value > threshold=19120.166015625
node_112: feature_name=a-HuIgG_0.03	feature_id[8].value <= threshold=19665.666015625
node_113: feature_name=SARS.CoV.2.S1.mFcTag	feature_id[3].value <= threshold=54010.166015625
node_114: feature_name=SARS.CoV.2.S1+S2	feature_id[12].value <= threshold=47258.43359375
node_115: feature_name=SARS.CoV.2.S1.RBD.mFc	feature_id[0].value > threshold=301.73333740234375
node_131: feature_name=a-HuIgG_0.03	feature_id[8].value <= threshold=12099.83349609375
node_132: feature_name=SARS.CoV.2.S2	feature_id[2].value > threshold=8858.0751953125
node_134: feature_name=Flu.B_Phu/.HA1+HA2	feature_id[11].value <= threshold=53839.16796875
node_135: feature_name=hCoV.HKU1.NP	feature_id[10].value <= threshold=1010.1666564941406
node_136: feature_name=MoIgA_0.10	feature_id[13].value > threshold=497.8333282470703
node_142: feature_name=SARS.CoV.2.S1.mFcTag	feature_id[3].value <= threshold=50462.283203125
Class: healthcare workers between 60 and 180 days after vaccination	
Rules_40	passed counts:7
node_0: feature_name=HuIgG_0.10	feature_id[1].value > threshold=19120.166015625
node_112: feature_name=a-HuIgG_0.03	feature_id[8].value <= threshold=19665.666015625
node_113: feature_name=SARS.CoV.2.S1.mFcTag	feature_id[3].value > threshold=54010.166015625
node_351: feature_name=SARS.CoV.2.S2	feature_id[2].value > threshold=37075.75

node_391: feature_name=SARS.CoV.2.S1.mFcTag	feature_id[3].value <= threshold=55462.833984375
node_392: feature_name=SARS.CoV.2.S1.mFcTag	feature_id[3].value <= threshold=55163.634765625
node_393: feature_name=hCoV.HKU1.NP	feature_id[10].value > threshold=14561.3330078125
Class: healthcare workers within 60 days after vaccination	
Rules_41	passed counts:7
node_0: feature_name=HuIgG_0.10	feature_id[1].value > threshold=19120.166015625
node_112: feature_name=a-HuIgG_0.03	feature_id[8].value <= threshold=19665.666015625
node_113: feature_name=SARS.CoV.2.S1.mFcTag	feature_id[3].value <= threshold=54010.166015625
node_114: feature_name=SARS.CoV.2.S1+S2	feature_id[12].value > threshold=47258.43359375
node_240: feature_name=HuIgG_0.03	feature_id[5].value > threshold=5455.5
node_266: feature_name=HuIgG_0.10	feature_id[1].value > threshold=24231.0
node_284: feature_name=HuIgG_0.03	feature_id[5].value > threshold=5469.0
node_286: feature_name=HuIgG_0.10	feature_id[1].value <= threshold=30107.666015625
node_287: feature_name=SARS.CoV.2.S1+S2	feature_id[12].value > threshold=47536.224609375
node_291: feature_name=SARS.CoV.2.S2	feature_id[2].value <= threshold=37653.75
node_292: feature_name=SARS.CoV.2.S2	feature_id[2].value <= threshold=36044.29296875
node_293: feature_name=HuIgG_0.03	feature_id[5].value > threshold=6329.5
node_301: feature_name=HuIgG_0.03	feature_id[5].value <= threshold=6562.16650390625
node_302: feature_name=Flu.B_Phu/.HA1+HA2	feature_id[11].value <= threshold=53338.533203125
Class: healthcare workers between 60 and 180 days after vaccination	
Rules_42	passed counts:7
node_0: feature_name=HuIgG_0.10	feature_id[1].value > threshold=19120.166015625
node_112: feature_name=a-HuIgG_0.03	feature_id[8].value <= threshold=19665.666015625

node_113: feature_name=SARS.CoV.2.S1.mFcTag	feature_id[3].value <= threshold=54010.166015625
node_114: feature_name=SARS.CoV.2.S1+S2	feature_id[12].value <= threshold=47258.43359375
node_115: feature_name=SARS.CoV.2.S1.RBD.mFc	feature_id[0].value > threshold=301.73333740234375
node_131: feature_name=a-HuIgG_0.03	feature_id[8].value <= threshold=12099.83349609375
node_132: feature_name=SARS.CoV.2.S2	feature_id[2].value > threshold=8858.0751953125
node_134: feature_name=Flu.B_Phu/.HA1+HA2	feature_id[11].value <= threshold=53839.16796875
node_135: feature_name=hCoV.HKU1.NP	feature_id[10].value > threshold=1010.1666564941406
node_145: feature_name=hCoV.HKU1.NP	feature_id[10].value <= threshold=15374.16650390625
node_146: feature_name=SARS.CoV.2.S1.mFcTag	feature_id[3].value <= threshold=50683.0
node_147: feature_name=SARS.CoV.2.Spike.RBD.rFc	feature_id[9].value <= threshold=45822.583984375
node_148: feature_name=SARS.CoV.2.S2	feature_id[2].value > threshold=10212.125
node_154: feature_name=Flu.B_Phu/.HA1+HA2	feature_id[11].value > threshold=49488.1171875
node_162: feature_name=MoIgA_0.10	feature_id[13].value > threshold=2877.1666259765625
node_164: feature_name=SARS.CoV.2.Spike.RBD.rFc	feature_id[9].value <= threshold=38138.451171875
Class: healthcare workers within 60 days after vaccination	
Rules_43	passed counts:7
node_0: feature_name=HuIgG_0.10	feature_id[1].value > threshold=19120.166015625
node_112: feature_name=a-HuIgG_0.03	feature_id[8].value <= threshold=19665.666015625
node_113: feature_name=SARS.CoV.2.S1.mFcTag	feature_id[3].value <= threshold=54010.166015625
node_114: feature_name=SARS.CoV.2.S1+S2	feature_id[12].value <= threshold=47258.43359375
node_115: feature_name=SARS.CoV.2.S1.RBD.mFc	feature_id[0].value > threshold=301.73333740234375
node_131: feature_name=a-HuIgG_0.03	feature_id[8].value <= threshold=12099.83349609375
node_132: feature_name=SARS.CoV.2.S2	feature_id[2].value > threshold=8858.0751953125
node_134: feature_name=Flu.B_Phu/.HA1+HA2	feature_id[11].value <= threshold=53839.16796875

node_135: feature_name=hCoV.HKU1.NP	feature_id[10].value <= threshold=1010.1666564941406
node_136: feature_name=MoIgA_0.10	feature_id[13].value <= threshold=497.8333282470703
node_137: feature_name=MoIgA_0.10	feature_id[13].value > threshold=153.0
Class: healthcare workers within 60 days after vaccination	
Rules_44	passed counts:6
node_0: feature_name=HuIgG_0.10	feature_id[1].value > threshold=19120.166015625
node_112: feature_name=a-HuIgG_0.03	feature_id[8].value > threshold=19665.666015625
node_428: feature_name=SARS.CoV.2.Spike.RBD.rFc	feature_id[9].value <= threshold=55147.1484375
node_429: feature_name=a-HuIgG_0.30	feature_id[4].value <= threshold=51289.166015625
node_430: feature_name=Flu.B_Phu/.HA1+HA2	feature_id[11].value > threshold=48173.93359375
node_434: feature_name=a-HuIgG_0.30	feature_id[4].value > threshold=47010.66796875
Class: healthcare workers between 60 and 180 days after vaccination	
Rules_45	passed counts:6
node_0: feature_name=HuIgG_0.10	feature_id[1].value > threshold=19120.166015625
node_112: feature_name=a-HuIgG_0.03	feature_id[8].value <= threshold=19665.666015625
node_113: feature_name=SARS.CoV.2.S1.mFcTag	feature_id[3].value > threshold=54010.166015625
node_351: feature_name=SARS.CoV.2.S2	feature_id[2].value > threshold=37075.75
node_391: feature_name=SARS.CoV.2.S1.mFcTag	feature_id[3].value > threshold=55462.833984375
node_425: feature_name=hCoV.NL63.S1_S2	feature_id[7].value > threshold=29337.0
Class: healthcare workers between 60 and 180 days after vaccination	
Rules_46	passed counts:6
node_0: feature_name=HuIgG_0.10	feature_id[1].value > threshold=19120.166015625

node_112: feature_name=a-HuIgG_0.03	feature_id[8].value <= threshold=19665.666015625
node_113: feature_name=SARS.CoV.2.S1.mFcTag	feature_id[3].value > threshold=54010.166015625
node_351: feature_name=SARS.CoV.2.S2	feature_id[2].value > threshold=37075.75
node_391: feature_name=SARS.CoV.2.S1.mFcTag	feature_id[3].value <= threshold=55462.833984375
node_392: feature_name=SARS.CoV.2.S1.mFcTag	feature_id[3].value <= threshold=55163.634765625
node_393: feature_name=hCoV.HKU1.NP	feature_id[10].value <= threshold=14561.3330078125
node_394: feature_name=hCoV.HKU1.NP	feature_id[10].value <= threshold=6453.333251953125
node_395: feature_name=hCoV.HKU1.NP	feature_id[10].value <= threshold=1990.1666259765625
node_396: feature_name=SARS.CoV.S1.HisTag	feature_id[6].value > threshold=12607.2998046875
node_398: feature_name=a-HuIgG_0.03	feature_id[8].value <= threshold=15419.83349609375
node_399: feature_name=SARS.CoV.2.S1.mFcTag	feature_id[3].value <= threshold=55018.716796875
Class: healthcare workers between 60 and 180 days after vaccination	
Rules_47	passed counts:6
node_0: feature_name=HuIgG_0.10	feature_id[1].value > threshold=19120.166015625
node_112: feature_name=a-HuIgG_0.03	feature_id[8].value <= threshold=19665.666015625
node_113: feature_name=SARS.CoV.2.S1.mFcTag	feature_id[3].value > threshold=54010.166015625
node_351: feature_name=SARS.CoV.2.S2	feature_id[2].value <= threshold=37075.75
node_352: feature_name=a-HuIgG_0.03	feature_id[8].value <= threshold=17721.1669921875
node_353: feature_name=MoIgA_0.10	feature_id[13].value <= threshold=536.0
node_354: feature_name=hCoV.HKU1.NP	feature_id[10].value > threshold=7098.5
Class: healthcare workers within 60 days after vaccination	
Rules_48	passed counts:6
node_0: feature_name=HuIgG_0.10	feature_id[1].value > threshold=19120.166015625

node_112: feature_name=a-HuIgG_0.03	feature_id[8].value <= threshold=19665.666015625
node_113: feature_name=SARS.CoV.2.S1.mFcTag	feature_id[3].value > threshold=54010.166015625
node_351: feature_name=SARS.CoV.2.S2	feature_id[2].value <= threshold=37075.75
node_352: feature_name=a-HuIgG_0.03	feature_id[8].value <= threshold=17721.1669921875
node_353: feature_name=MoIgA_0.10	feature_id[13].value <= threshold=536.0
node_354: feature_name=hCoV.HKU1.NP	feature_id[10].value <= threshold=7098.5
node_355: feature_name=MoIgA_0.10	feature_id[13].value <= threshold=462.0
node_356: feature_name=hCoV.HKU1.NP	feature_id[10].value <= threshold=2790.583251953125
node_357: feature_name=hCoV.NL63.S1_S2	feature_id[7].value > threshold=36099.0
node_359: feature_name=hCoV.HKU1.NP	feature_id[10].value > threshold=1348.6666870117188
Class: healthcare workers within 60 days after vaccination	
Rules_49	passed counts:6
node_0: feature_name=HuIgG_0.10	feature_id[1].value > threshold=19120.166015625
node_112: feature_name=a-HuIgG_0.03	feature_id[8].value <= threshold=19665.666015625
node_113: feature_name=SARS.CoV.2.S1.mFcTag	feature_id[3].value <= threshold=54010.166015625
node_114: feature_name=SARS.CoV.2.S1+S2	feature_id[12].value > threshold=47258.43359375
node_240: feature_name=HuIgG_0.03	feature_id[5].value > threshold=5455.5
node_266: feature_name=HuIgG_0.10	feature_id[1].value > threshold=24231.0
node_284: feature_name=HuIgG_0.03	feature_id[5].value > threshold=5469.0
node_286: feature_name=HuIgG_0.10	feature_id[1].value <= threshold=30107.666015625
node_287: feature_name=SARS.CoV.2.S1+S2	feature_id[12].value > threshold=47536.224609375
node_291: feature_name=SARS.CoV.2.S2	feature_id[2].value > threshold=37653.75
node_329: feature_name=SARS.CoV.2.S1.mFcTag	feature_id[3].value > threshold=53346.333984375
node_345: feature_name=SARS.CoV.2.S1.mFcTag	feature_id[3].value > threshold=53555.333984375

Class: healthcare workers within 60 days after vaccination	
Rules_50	passed counts:6
node_0: feature_name=HuIgG_0.10	feature_id[1].value > threshold=19120.166015625
node_112: feature_name=a-HuIgG_0.03	feature_id[8].value <= threshold=19665.666015625
node_113: feature_name=SARS.CoV.2.S1.mFcTag	feature_id[3].value <= threshold=54010.166015625
node_114: feature_name=SARS.CoV.2.S1+S2	feature_id[12].value > threshold=47258.43359375
node_240: feature_name=HuIgG_0.03	feature_id[5].value > threshold=5455.5
node_266: feature_name=HuIgG_0.10	feature_id[1].value <= threshold=24231.0
node_267: feature_name=hCoV.HKU1.NP	feature_id[10].value > threshold=2123.0000610351562
node_271: feature_name=SARS.CoV.S1.HisTag	feature_id[6].value > threshold=8281.83349609375
node_273: feature_name=Flu.B_Phu/.HA1+HA2	feature_id[11].value > threshold=51424.19921875
node_275: feature_name=a-HuIgG_0.30	feature_id[4].value <= threshold=41169.833984375
Class: healthcare workers within 60 days after vaccination	
Rules_51	passed counts:6
node_0: feature_name=HuIgG_0.10	feature_id[1].value > threshold=19120.166015625
node_112: feature_name=a-HuIgG_0.03	feature_id[8].value <= threshold=19665.666015625
node_113: feature_name=SARS.CoV.2.S1.mFcTag	feature_id[3].value <= threshold=54010.166015625
node_114: feature_name=SARS.CoV.2.S1+S2	feature_id[12].value > threshold=47258.43359375
node_240: feature_name=HuIgG_0.03	feature_id[5].value > threshold=5455.5
node_266: feature_name=HuIgG_0.10	feature_id[1].value <= threshold=24231.0
node_267: feature_name=hCoV.HKU1.NP	feature_id[10].value > threshold=2123.0000610351562
node_271: feature_name=SARS.CoV.S1.HisTag	feature_id[6].value <= threshold=8281.83349609375
Class: healthcare workers between 60 and 180 days after vaccination	

Rules_52	passed counts:6
node_0: feature_name=HuIgG_0.10	feature_id[1].value > threshold=19120.166015625
node_112: feature_name=a-HuIgG_0.03	feature_id[8].value <= threshold=19665.666015625
node_113: feature_name=SARS.CoV.2.S1.mFcTag	feature_id[3].value <= threshold=54010.166015625
node_114: feature_name=SARS.CoV.2.S1+S2	feature_id[12].value > threshold=47258.43359375
node_240: feature_name=HuIgG_0.03	feature_id[5].value <= threshold=5455.5
node_241: feature_name=SARS.CoV.S1.HisTag	feature_id[6].value > threshold=745.7222290039062
node_243: feature_name=SARS.CoV.2.S1+S2	feature_id[12].value > threshold=48812.91796875
node_253: feature_name=SARS.CoV.S1.HisTag	feature_id[6].value > threshold=2772.300048828125
node_257: feature_name=HuIgG_0.10	feature_id[1].value <= threshold=26991.8330078125
node_258: feature_name=SARS.CoV.2.S1.RBD.mFc	feature_id[0].value <= threshold=41406.666015625
node_259: feature_name=SARS.CoV.2.S1.RBD.mFc	feature_id[0].value <= threshold=40954.3828125
Class: healthcare workers within 60 days after vaccination	
Rules_53	passed counts:6
node_0: feature_name=HuIgG_0.10	feature_id[1].value > threshold=19120.166015625
node_112: feature_name=a-HuIgG_0.03	feature_id[8].value <= threshold=19665.666015625
node_113: feature_name=SARS.CoV.2.S1.mFcTag	feature_id[3].value <= threshold=54010.166015625
node_114: feature_name=SARS.CoV.2.S1+S2	feature_id[12].value <= threshold=47258.43359375
node_115: feature_name=SARS.CoV.2.S1.RBD.mFc	feature_id[0].value > threshold=301.73333740234375
node_131: feature_name=a-HuIgG_0.03	feature_id[8].value > threshold=12099.83349609375
node_183: feature_name=Flu.B_Phu/.HA1+HA2	feature_id[11].value > threshold=41918.533203125
node_187: feature_name=SARS.CoV.2.Spike.RBD.rFc	feature_id[9].value > threshold=27438.341796875
node_197: feature_name=SARS.CoV.2.S2	feature_id[2].value <= threshold=35177.640625

node_198: feature_name=hCoV.HKU1.NP	feature_id[10].value > threshold=2645.916748046875
node_210: feature_name=Flu.B_Phu/.HA1+HA2	feature_id[11].value <= threshold=54029.166015625
node_211: feature_name=HuIgG_0.10	feature_id[1].value > threshold=21359.5
node_213: feature_name=hCoV.NL63.S1_S2	feature_id[7].value > threshold=32523.4248046875
node_215: feature_name=HuIgG_0.10	feature_id[1].value > threshold=23274.166015625
node_217: feature_name=HuIgG_0.10	feature_id[1].value > threshold=25231.1669921875
node_227: feature_name=SARS.CoV.2.S2	feature_id[2].value <= threshold=18038.125
node_228: feature_name=hCoV.HKU1.NP	feature_id[10].value <= threshold=11390.08349609375
Class: healthcare workers between 60 and 180 days after vaccination	
Rules_54	passed counts:6
node_0: feature_name=HuIgG_0.10	feature_id[1].value > threshold=19120.166015625
node_112: feature_name=a-HuIgG_0.03	feature_id[8].value <= threshold=19665.666015625
node_113: feature_name=SARS.CoV.2.S1.mFcTag	feature_id[3].value <= threshold=54010.166015625
node_114: feature_name=SARS.CoV.2.S1+S2	feature_id[12].value <= threshold=47258.43359375
node_115: feature_name=SARS.CoV.2.S1.RBD.mFc	feature_id[0].value > threshold=301.73333740234375
node_131: feature_name=a-HuIgG_0.03	feature_id[8].value > threshold=12099.83349609375
node_183: feature_name=Flu.B_Phu/.HA1+HA2	feature_id[11].value > threshold=41918.533203125
node_187: feature_name=SARS.CoV.2.Spike.RBD.rFc	feature_id[9].value > threshold=27438.341796875
node_197: feature_name=SARS.CoV.2.S2	feature_id[2].value <= threshold=35177.640625
node_198: feature_name=hCoV.HKU1.NP	feature_id[10].value > threshold=2645.916748046875
node_210: feature_name=Flu.B_Phu/.HA1+HA2	feature_id[11].value <= threshold=54029.166015625
node_211: feature_name=HuIgG_0.10	feature_id[1].value <= threshold=21359.5
Class: healthcare workers between 60 and 180 days after vaccination	

Rules_55	passed counts:6
node_0: feature_name=HuIgG_0.10	feature_id[1].value > threshold=19120.166015625
node_112: feature_name=a-HuIgG_0.03	feature_id[8].value <= threshold=19665.666015625
node_113: feature_name=SARS.CoV.2.S1.mFcTag	feature_id[3].value <= threshold=54010.166015625
node_114: feature_name=SARS.CoV.2.S1+S2	feature_id[12].value <= threshold=47258.43359375
node_115: feature_name=SARS.CoV.2.S1.RBD.mFc	feature_id[0].value > threshold=301.73333740234375
node_131: feature_name=a-HuIgG_0.03	feature_id[8].value > threshold=12099.83349609375
node_183: feature_name=Flu.B_Phu/.HA1+HA2	feature_id[11].value <= threshold=41918.533203125
node_184: feature_name=a-HuIgG_0.03	feature_id[8].value > threshold=14683.6669921875
Class: healthcare workers over 180 days after vaccination	
Rules_56	passed counts:6
node_0: feature_name=HuIgG_0.10	feature_id[1].value > threshold=19120.166015625
node_112: feature_name=a-HuIgG_0.03	feature_id[8].value <= threshold=19665.666015625
node_113: feature_name=SARS.CoV.2.S1.mFcTag	feature_id[3].value <= threshold=54010.166015625
node_114: feature_name=SARS.CoV.2.S1+S2	feature_id[12].value <= threshold=47258.43359375
node_115: feature_name=SARS.CoV.2.S1.RBD.mFc	feature_id[0].value > threshold=301.73333740234375
node_131: feature_name=a-HuIgG_0.03	feature_id[8].value <= threshold=12099.83349609375
node_132: feature_name=SARS.CoV.2.S2	feature_id[2].value > threshold=8858.0751953125
node_134: feature_name=Flu.B_Phu/.HA1+HA2	feature_id[11].value > threshold=53839.16796875
node_180: feature_name=a-HuIgG_0.30	feature_id[4].value > threshold=40436.333984375
Class: healthcare workers between 60 and 180 days after vaccination	
Rules_57	passed counts:6
node_0: feature_name=HuIgG_0.10	feature_id[1].value <= threshold=19120.166015625

node_1: feature_name=SARS.CoV.2.S1.mFcTag	feature_id[3].value > threshold=5354.388916015625
node_9: feature_name=a-HuIgG_0.30	feature_id[4].value > threshold=33726.833984375
node_55: feature_name=SARS.CoV.2.S1.mFcTag	feature_id[3].value > threshold=43185.68359375
node_67: feature_name=a-HuIgG_0.03	feature_id[8].value <= threshold=10858.0
node_68: feature_name=MoIgA_0.10	feature_id[13].value <= threshold=246.83333587646484
node_69: feature_name=hCoV.HKU1.NP	feature_id[10].value > threshold=1960.7500610351562
node_73: feature_name=hCoV.NL63.S1_S2	feature_id[7].value > threshold=25846.875
Class: healthcare workers between 60 and 180 days after vaccination	
Rules_58	passed counts:5
node_0: feature_name=HuIgG_0.10	feature_id[1].value > threshold=19120.166015625
node_112: feature_name=a-HuIgG_0.03	feature_id[8].value <= threshold=19665.666015625
node_113: feature_name=SARS.CoV.2.S1.mFcTag	feature_id[3].value > threshold=54010.166015625
node_351: feature_name=SARS.CoV.2.S2	feature_id[2].value <= threshold=37075.75
node_352: feature_name=a-HuIgG_0.03	feature_id[8].value <= threshold=17721.1669921875
node_353: feature_name=MoIgA_0.10	feature_id[13].value > threshold=536.0
node_367: feature_name=HuIgG_0.03	feature_id[5].value <= threshold=5634.333251953125
node_368: feature_name=SARS.CoV.2.S1+S2	feature_id[12].value > threshold=52432.75
node_378: feature_name=a-HuIgG_0.30	feature_id[4].value <= threshold=49923.66796875
Class: healthcare workers within 60 days after vaccination	
Rules_59	passed counts:5
node_0: feature_name=HuIgG_0.10	feature_id[1].value > threshold=19120.166015625
node_112: feature_name=a-HuIgG_0.03	feature_id[8].value <= threshold=19665.666015625
node_113: feature_name=SARS.CoV.2.S1.mFcTag	feature_id[3].value > threshold=54010.166015625

node_351: feature_name=SARS.CoV.2.S2	feature_id[2].value <= threshold=37075.75
node_352: feature_name=a-HuIgG_0.03	feature_id[8].value <= threshold=17721.1669921875
node_353: feature_name=MoIgA_0.10	feature_id[13].value <= threshold=536.0
node_354: feature_name=hCoV.HKU1.NP	feature_id[10].value <= threshold=7098.5
node_355: feature_name=MoIgA_0.10	feature_id[13].value <= threshold=462.0
node_356: feature_name=hCoV.HKU1.NP	feature_id[10].value <= threshold=2790.583251953125
node_357: feature_name=hCoV.NL63.S1_S2	feature_id[7].value <= threshold=36099.0
Class: healthcare workers between 60 and 180 days after vaccination	
Rules_60	passed counts:5
node_0: feature_name=HuIgG_0.10	feature_id[1].value > threshold=19120.166015625
node_112: feature_name=a-HuIgG_0.03	feature_id[8].value <= threshold=19665.666015625
node_113: feature_name=SARS.CoV.2.S1.mFcTag	feature_id[3].value <= threshold=54010.166015625
node_114: feature_name=SARS.CoV.2.S1+S2	feature_id[12].value > threshold=47258.43359375
node_240: feature_name=HuIgG_0.03	feature_id[5].value > threshold=5455.5
node_266: feature_name=HuIgG_0.10	feature_id[1].value <= threshold=24231.0
node_267: feature_name=hCoV.HKU1.NP	feature_id[10].value > threshold=2123.0000610351562
node_271: feature_name=SARS.CoV.S1.HisTag	feature_id[6].value > threshold=8281.83349609375
node_273: feature_name=Flu.B_Phu/.HA1+HA2	feature_id[11].value > threshold=51424.19921875
node_275: feature_name=a-HuIgG_0.30	feature_id[4].value > threshold=41169.833984375
node_277: feature_name=a-HuIgG_0.30	feature_id[4].value <= threshold=42934.333984375
Class: healthcare workers between 60 and 180 days after vaccination	
Rules_61	passed counts:5
node_0: feature_name=HuIgG_0.10	feature_id[1].value > threshold=19120.166015625

node_112: feature_name=a-HuIgG_0.03	feature_id[8].value <= threshold=19665.666015625
node_113: feature_name=SARS.CoV.2.S1.mFcTag	feature_id[3].value <= threshold=54010.166015625
node_114: feature_name=SARS.CoV.2.S1+S2	feature_id[12].value > threshold=47258.43359375
node_240: feature_name=HuIgG_0.03	feature_id[5].value <= threshold=5455.5
node_241: feature_name=SARS.CoV.S1.HisTag	feature_id[6].value > threshold=745.7222290039062
node_243: feature_name=SARS.CoV.2.S1+S2	feature_id[12].value <= threshold=48812.91796875
node_244: feature_name=SARS.CoV.2.S2	feature_id[2].value > threshold=26981.416015625
node_246: feature_name=Flu.B_Phu/.HA1+HA2	feature_id[11].value > threshold=52223.0
node_250: feature_name=SARS.CoV.2.S1.mFcTag	feature_id[3].value > threshold=49570.5
Class: healthcare workers between 60 and 180 days after vaccination	
Rules_62	passed counts:5
node_0: feature_name=HuIgG_0.10	feature_id[1].value > threshold=19120.166015625
node_112: feature_name=a-HuIgG_0.03	feature_id[8].value <= threshold=19665.666015625
node_113: feature_name=SARS.CoV.2.S1.mFcTag	feature_id[3].value <= threshold=54010.166015625
node_114: feature_name=SARS.CoV.2.S1+S2	feature_id[12].value <= threshold=47258.43359375
node_115: feature_name=SARS.CoV.2.S1.RBD.mFc	feature_id[0].value > threshold=301.73333740234375
node_131: feature_name=a-HuIgG_0.03	feature_id[8].value > threshold=12099.83349609375
node_183: feature_name=Flu.B_Phu/.HA1+HA2	feature_id[11].value > threshold=41918.533203125
node_187: feature_name=SARS.CoV.2.Spike.RBD.rFc	feature_id[9].value > threshold=27438.341796875
node_197: feature_name=SARS.CoV.2.S2	feature_id[2].value <= threshold=35177.640625
node_198: feature_name=hCoV.HKU1.NP	feature_id[10].value <= threshold=2645.916748046875
node_199: feature_name=hCoV.HKU1.NP	feature_id[10].value > threshold=666.6666870117188
node_203: feature_name=HuIgG_0.10	feature_id[1].value > threshold=24756.5
node_205: feature_name=SARS.CoV.2.S1+S2	feature_id[12].value > threshold=44218.416015625

node_207: feature_name=SARS.CoV.2.Spike.RBD.rFc	feature_id[9].value <= threshold=46720.900390625
Class: healthcare workers within 60 days after vaccination	
Rules_63	passed counts:5
node_0: feature_name=HuIgG_0.10	feature_id[1].value > threshold=19120.166015625
node_112: feature_name=a-HuIgG_0.03	feature_id[8].value <= threshold=19665.666015625
node_113: feature_name=SARS.CoV.2.S1.mFcTag	feature_id[3].value <= threshold=54010.166015625
node_114: feature_name=SARS.CoV.2.S1+S2	feature_id[12].value <= threshold=47258.43359375
node_115: feature_name=SARS.CoV.2.S1.RBD.mFc	feature_id[0].value > threshold=301.73333740234375
node_131: feature_name=a-HuIgG_0.03	feature_id[8].value <= threshold=12099.83349609375
node_132: feature_name=SARS.CoV.2.S2	feature_id[2].value <= threshold=8858.0751953125
Class: healthcare workers between 60 and 180 days after vaccination	
Rules_64	passed counts:5
node_0: feature_name=HuIgG_0.10	feature_id[1].value <= threshold=19120.166015625
node_1: feature_name=SARS.CoV.2.S1.mFcTag	feature_id[3].value > threshold=5354.388916015625
node_9: feature_name=a-HuIgG_0.30	feature_id[4].value <= threshold=33726.833984375
node_10: feature_name=HuIgG_0.03	feature_id[5].value <= threshold=1023.1666564941406
node_11: feature_name=SARS.CoV.2.S1.mFcTag	feature_id[3].value > threshold=34194.916015625
node_17: feature_name=HuIgG_0.10	feature_id[1].value > threshold=7582.83349609375
Class: healthcare workers over 180 days after vaccination	
Rules_65	passed counts:4
node_0: feature_name=HuIgG_0.10	feature_id[1].value > threshold=19120.166015625
node_112: feature_name=a-HuIgG_0.03	feature_id[8].value > threshold=19665.666015625

node_428: feature_name=SARS.CoV.2.Spike.RBD.rFc	feature_id[9].value > threshold=55147.1484375
Class: healthcare workers between 60 and 180 days after vaccination	
Rules_66	passed counts:4
node_0: feature_name=HuIgG_0.10	feature_id[1].value > threshold=19120.166015625
node_112: feature_name=a-HuIgG_0.03	feature_id[8].value > threshold=19665.666015625
node_428: feature_name=SARS.CoV.2.Spike.RBD.rFc	feature_id[9].value <= threshold=55147.1484375
node_429: feature_name=a-HuIgG_0.30	feature_id[4].value > threshold=51289.166015625
node_437: feature_name=HuIgG_0.10	feature_id[1].value > threshold=25959.0
node_441: feature_name=hCoV.NL63.S1_S2	feature_id[7].value <= threshold=43930.5234375
node_442: feature_name=a-HuIgG_0.03	feature_id[8].value > threshold=23272.1669921875
Class: healthcare workers between 60 and 180 days after vaccination	
Rules_67	passed counts:4
node_0: feature_name=HuIgG_0.10	feature_id[1].value > threshold=19120.166015625
node_112: feature_name=a-HuIgG_0.03	feature_id[8].value <= threshold=19665.666015625
node_113: feature_name=SARS.CoV.2.S1.mFcTag	feature_id[3].value <= threshold=54010.166015625
node_114: feature_name=SARS.CoV.2.S1+S2	feature_id[12].value > threshold=47258.43359375
node_240: feature_name=HuIgG_0.03	feature_id[5].value > threshold=5455.5
node_266: feature_name=HuIgG_0.10	feature_id[1].value > threshold=24231.0
node_284: feature_name=HuIgG_0.03	feature_id[5].value > threshold=5469.0
node_286: feature_name=HuIgG_0.10	feature_id[1].value <= threshold=30107.666015625
node_287: feature_name=SARS.CoV.2.S1+S2	feature_id[12].value > threshold=47536.224609375
node_291: feature_name=SARS.CoV.2.S2	feature_id[2].value > threshold=37653.75
node_329: feature_name=SARS.CoV.2.S1.mFcTag	feature_id[3].value > threshold=53346.333984375

node_345: feature_name=SARS.CoV.2.S1.mFcTag	feature_id[3].value <= threshold=53555.333984375
node_346: feature_name=SARS.CoV.2.S2	feature_id[2].value > threshold=45480.583984375
Class: healthcare workers between 60 and 180 days after vaccination	
Rules_68	passed counts:4
node_0: feature_name=HuIgG_0.10	feature_id[1].value > threshold=19120.166015625
node_112: feature_name=a-HuIgG_0.03	feature_id[8].value <= threshold=19665.666015625
node_113: feature_name=SARS.CoV.2.S1.mFcTag	feature_id[3].value <= threshold=54010.166015625
node_114: feature_name=SARS.CoV.2.S1+S2	feature_id[12].value > threshold=47258.43359375
node_240: feature_name=HuIgG_0.03	feature_id[5].value > threshold=5455.5
node_266: feature_name=HuIgG_0.10	feature_id[1].value > threshold=24231.0
node_284: feature_name=HuIgG_0.03	feature_id[5].value > threshold=5469.0
node_286: feature_name=HuIgG_0.10	feature_id[1].value <= threshold=30107.666015625
node_287: feature_name=SARS.CoV.2.S1+S2	feature_id[12].value > threshold=47536.224609375
node_291: feature_name=SARS.CoV.2.S2	feature_id[2].value > threshold=37653.75
node_329: feature_name=SARS.CoV.2.S1.mFcTag	feature_id[3].value <= threshold=53346.333984375
node_330: feature_name=Flu.B_Phu/.HA1+HA2	feature_id[11].value > threshold=53733.0
node_340: feature_name=SARS.CoV.2.S1+S2	feature_id[12].value > threshold=51770.29296875
Class: healthcare workers within 60 days after vaccination	
Rules_69	passed counts:4
node_0: feature_name=HuIgG_0.10	feature_id[1].value > threshold=19120.166015625
node_112: feature_name=a-HuIgG_0.03	feature_id[8].value <= threshold=19665.666015625
node_113: feature_name=SARS.CoV.2.S1.mFcTag	feature_id[3].value <= threshold=54010.166015625
node_114: feature_name=SARS.CoV.2.S1+S2	feature_id[12].value > threshold=47258.43359375

node_240: feature_name=HuIgG_0.03	feature_id[5].value > threshold=5455.5
node_266: feature_name=HuIgG_0.10	feature_id[1].value > threshold=24231.0
node_284: feature_name=HuIgG_0.03	feature_id[5].value > threshold=5469.0
node_286: feature_name=HuIgG_0.10	feature_id[1].value <= threshold=30107.666015625
node_287: feature_name=SARS.CoV.2.S1+S2	feature_id[12].value > threshold=47536.224609375
node_291: feature_name=SARS.CoV.2.S2	feature_id[2].value <= threshold=37653.75
node_292: feature_name=SARS.CoV.2.S2	feature_id[2].value > threshold=36044.29296875
node_322: feature_name=hCoV.HKU1.NP	feature_id[10].value > threshold=2383.1666870117188
node_324: feature_name=SARS.CoV.2.S1+S2	feature_id[12].value > threshold=49449.75
Class: healthcare workers between 60 and 180 days after vaccination	
Rules_70	passed counts:4
node_0: feature_name=HuIgG_0.10	feature_id[1].value > threshold=19120.166015625
node_112: feature_name=a-HuIgG_0.03	feature_id[8].value <= threshold=19665.666015625
node_113: feature_name=SARS.CoV.2.S1.mFcTag	feature_id[3].value <= threshold=54010.166015625
node_114: feature_name=SARS.CoV.2.S1+S2	feature_id[12].value > threshold=47258.43359375
node_240: feature_name=HuIgG_0.03	feature_id[5].value > threshold=5455.5
node_266: feature_name=HuIgG_0.10	feature_id[1].value > threshold=24231.0
node_284: feature_name=HuIgG_0.03	feature_id[5].value > threshold=5469.0
node_286: feature_name=HuIgG_0.10	feature_id[1].value <= threshold=30107.666015625
node_287: feature_name=SARS.CoV.2.S1+S2	feature_id[12].value > threshold=47536.224609375
node_291: feature_name=SARS.CoV.2.S2	feature_id[2].value <= threshold=37653.75
node_292: feature_name=SARS.CoV.2.S2	feature_id[2].value <= threshold=36044.29296875
node_293: feature_name=HuIgG_0.03	feature_id[5].value > threshold=6329.5
node_301: feature_name=HuIgG_0.03	feature_id[5].value > threshold=6562.16650390625

node_307: feature_name=Flu.B_Phu/.HA1+HA2	feature_id[11].value <= threshold=54123.5
node_308: feature_name=MoIgA_0.10	feature_id[13].value > threshold=21.166666984558105
node_310: feature_name=hCoV.NL63.S1_S2	feature_id[7].value <= threshold=33291.458984375
node_311: feature_name=SARS.CoV.2.S1+S2	feature_id[12].value <= threshold=49482.583984375
Class: healthcare workers within 60 days after vaccination	
Rules_71	passed counts:4
node_0: feature_name=HuIgG_0.10	feature_id[1].value > threshold=19120.166015625
node_112: feature_name=a-HuIgG_0.03	feature_id[8].value <= threshold=19665.666015625
node_113: feature_name=SARS.CoV.2.S1.mFcTag	feature_id[3].value <= threshold=54010.166015625
node_114: feature_name=SARS.CoV.2.S1+S2	feature_id[12].value > threshold=47258.43359375
node_240: feature_name=HuIgG_0.03	feature_id[5].value > threshold=5455.5
node_266: feature_name=HuIgG_0.10	feature_id[1].value > threshold=24231.0
node_284: feature_name=HuIgG_0.03	feature_id[5].value > threshold=5469.0
node_286: feature_name=HuIgG_0.10	feature_id[1].value <= threshold=30107.666015625
node_287: feature_name=SARS.CoV.2.S1+S2	feature_id[12].value > threshold=47536.224609375
node_291: feature_name=SARS.CoV.2.S2	feature_id[2].value <= threshold=37653.75
node_292: feature_name=SARS.CoV.2.S2	feature_id[2].value <= threshold=36044.29296875
node_293: feature_name=HuIgG_0.03	feature_id[5].value <= threshold=6329.5
node_294: feature_name=Flu.B_Phu/.HA1+HA2	feature_id[11].value > threshold=50410.16796875
node_296: feature_name=HuIgG_0.03	feature_id[5].value <= threshold=5639.0
node_297: feature_name=SARS.CoV.2.S2	feature_id[2].value <= threshold=30354.591796875
Class: healthcare workers within 60 days after vaccination	
Rules_72	passed counts:4

node_0: feature_name=HuIgG_0.10	feature_id[1].value > threshold=19120.166015625
node_112: feature_name=a-HuIgG_0.03	feature_id[8].value <= threshold=19665.666015625
node_113: feature_name=SARS.CoV.2.S1.mFcTag	feature_id[3].value <= threshold=54010.166015625
node_114: feature_name=SARS.CoV.2.S1+S2	feature_id[12].value > threshold=47258.43359375
node_240: feature_name=HuIgG_0.03	feature_id[5].value <= threshold=5455.5
node_241: feature_name=SARS.CoV.S1.HisTag	feature_id[6].value > threshold=745.7222290039062
node_243: feature_name=SARS.CoV.2.S1+S2	feature_id[12].value > threshold=48812.91796875
node_253: feature_name=SARS.CoV.S1.HisTag	feature_id[6].value > threshold=2772.300048828125
node_257: feature_name=HuIgG_0.10	feature_id[1].value > threshold=26991.8330078125
node_263: feature_name=a-HuIgG_0.03	feature_id[8].value > threshold=11532.0
Class: healthcare workers within 60 days after vaccination	
Rules_73	passed counts:4
node_0: feature_name=HuIgG_0.10	feature_id[1].value > threshold=19120.166015625
node_112: feature_name=a-HuIgG_0.03	feature_id[8].value <= threshold=19665.666015625
node_113: feature_name=SARS.CoV.2.S1.mFcTag	feature_id[3].value <= threshold=54010.166015625
node_114: feature_name=SARS.CoV.2.S1+S2	feature_id[12].value <= threshold=47258.43359375
node_115: feature_name=SARS.CoV.2.S1.RBD.mFc	feature_id[0].value <= threshold=301.73333740234375
node_116: feature_name=SARS.CoV.2.S1+S2	feature_id[12].value <= threshold=3298.25
node_117: feature_name=a-HuIgG_0.30	feature_id[4].value > threshold=2425.3333129882812
Class: healthcare workers within 60 days after vaccination	
Rules_74	passed counts:4
node_0: feature_name=HuIgG_0.10	feature_id[1].value <= threshold=19120.166015625
node_1: feature_name=SARS.CoV.2.S1.mFcTag	feature_id[3].value > threshold=5354.388916015625

node_9: feature_name=a-HuIgG_0.30	feature_id[4].value > threshold=33726.833984375
node_55: feature_name=SARS.CoV.2.S1.mFcTag	feature_id[3].value > threshold=43185.68359375
node_67: feature_name=a-HuIgG_0.03	feature_id[8].value > threshold=10858.0
node_83: feature_name=HuIgG_0.03	feature_id[5].value <= threshold=3796.0
node_84: feature_name=hCoV.NL63.S1_S2	feature_id[7].value > threshold=29814.5
node_96: feature_name=hCoV.NL63.S1_S2	feature_id[7].value > threshold=34078.0
node_98: feature_name=SARS.CoV.S1.HisTag	feature_id[6].value > threshold=9674.150390625
node_100: feature_name=SARS.CoV.2.S1.mFcTag	feature_id[3].value <= threshold=55180.015625
Class: healthcare workers over 180 days after vaccination	
Rules_75	passed counts:4
node_0: feature_name=HuIgG_0.10	feature_id[1].value <= threshold=19120.166015625
node_1: feature_name=SARS.CoV.2.S1.mFcTag	feature_id[3].value > threshold=5354.388916015625
node_9: feature_name=a-HuIgG_0.30	feature_id[4].value > threshold=33726.833984375
node_55: feature_name=SARS.CoV.2.S1.mFcTag	feature_id[3].value > threshold=43185.68359375
node_67: feature_name=a-HuIgG_0.03	feature_id[8].value > threshold=10858.0
node_83: feature_name=HuIgG_0.03	feature_id[5].value <= threshold=3796.0
node_84: feature_name=hCoV.NL63.S1_S2	feature_id[7].value <= threshold=29814.5
node_85: feature_name=hCoV.NL63.S1_S2	feature_id[7].value <= threshold=22280.4169921875
node_86: feature_name=Flu.B_Phu/.HA1+HA2	feature_id[11].value <= threshold=50957.43359375
Class: healthcare workers over 180 days after vaccination	
Rules_76	passed counts:4
node_0: feature_name=HuIgG_0.10	feature_id[1].value <= threshold=19120.166015625
node_1: feature_name=SARS.CoV.2.S1.mFcTag	feature_id[3].value > threshold=5354.388916015625

node_9: feature_name=a-HuIgG_0.30	feature_id[4].value <= threshold=33726.833984375
node_10: feature_name=HuIgG_0.03	feature_id[5].value > threshold=1023.1666564941406
node_20: feature_name=hCoV.HKU1.NP	feature_id[10].value > threshold=-20.05833387374878
node_22: feature_name=SARS.CoV.S1.HisTag	feature_id[6].value > threshold=400.3000030517578
node_26: feature_name=SARS.CoV.S1.HisTag	feature_id[6].value <= threshold=15087.41650390625
node_27: feature_name=SARS.CoV.2.S1.RBD.mFc	feature_id[0].value <= threshold=43991.083984375
node_28: feature_name=SARS.CoV.2.S1.RBD.mFc	feature_id[0].value > threshold=33656.484375
node_30: feature_name=SARS.CoV.2.S1+S2	feature_id[12].value <= threshold=30126.75
node_31: feature_name=SARS.CoV.S1.HisTag	feature_id[6].value > threshold=3654.1666259765625
Class: healthcare workers over 180 days after vaccination	
Rules_77	passed counts:4
node_0: feature_name=HuIgG_0.10	feature_id[1].value <= threshold=19120.166015625
node_1: feature_name=SARS.CoV.2.S1.mFcTag	feature_id[3].value <= threshold=5354.388916015625
node_2: feature_name=SARS.CoV.S1.HisTag	feature_id[6].value > threshold=-423.2833251953125
node_4: feature_name=SARS.CoV.2.S2	feature_id[2].value <= threshold=566.3889007568359
node_5: feature_name=SARS.CoV.2.S2	feature_id[2].value <= threshold=462.55555725097656
Class: unvaccinated healthcare workers	
Rules_78	passed counts:3
node_0: feature_name=HuIgG_0.10	feature_id[1].value > threshold=19120.166015625
node_112: feature_name=a-HuIgG_0.03	feature_id[8].value > threshold=19665.666015625
node_428: feature_name=SARS.CoV.2.Spike.RBD.rFc	feature_id[9].value <= threshold=55147.1484375
node_429: feature_name=a-HuIgG_0.30	feature_id[4].value > threshold=51289.166015625
node_437: feature_name=HuIgG_0.10	feature_id[1].value > threshold=25959.0

node_441: feature_name=hCoV.NL63.S1_S2	feature_id[7].value <= threshold=43930.5234375
node_442: feature_name=a-HuIgG_0.03	feature_id[8].value <= threshold=23272.1669921875
node_443: feature_name=hCoV.HKU1.NP	feature_id[10].value > threshold=1474.3833312988281
Class: healthcare workers over 180 days after vaccination	
Rules_79	passed counts:3
node_0: feature_name=HuIgG_0.10	feature_id[1].value > threshold=19120.166015625
node_112: feature_name=a-HuIgG_0.03	feature_id[8].value > threshold=19665.666015625
node_428: feature_name=SARS.CoV.2.Spike.RBD.rFc	feature_id[9].value <= threshold=55147.1484375
node_429: feature_name=a-HuIgG_0.30	feature_id[4].value > threshold=51289.166015625
node_437: feature_name=HuIgG_0.10	feature_id[1].value > threshold=25959.0
node_441: feature_name=hCoV.NL63.S1_S2	feature_id[7].value <= threshold=43930.5234375
node_442: feature_name=a-HuIgG_0.03	feature_id[8].value <= threshold=23272.1669921875
node_443: feature_name=hCoV.HKU1.NP	feature_id[10].value <= threshold=1474.3833312988281
node_444: feature_name=a-HuIgG_0.03	feature_id[8].value <= threshold=22815.1669921875
Class: healthcare workers between 60 and 180 days after vaccination	
Rules_80	passed counts:3
node_0: feature_name=HuIgG_0.10	feature_id[1].value > threshold=19120.166015625
node_112: feature_name=a-HuIgG_0.03	feature_id[8].value > threshold=19665.666015625
node_428: feature_name=SARS.CoV.2.Spike.RBD.rFc	feature_id[9].value <= threshold=55147.1484375
node_429: feature_name=a-HuIgG_0.30	feature_id[4].value <= threshold=51289.166015625
node_430: feature_name=Flu.B_Phu/.HA1+HA2	feature_id[11].value <= threshold=48173.93359375
node_431: feature_name=SARS.CoV.2.S1+S2	feature_id[12].value > threshold=39605.06640625
Class: healthcare workers over 180 days after vaccination	

Rules_81	passed counts:3
node_0: feature_name=HuIgG_0.10	feature_id[1].value > threshold=19120.166015625
node_112: feature_name=a-HuIgG_0.03	feature_id[8].value <= threshold=19665.666015625
node_113: feature_name=SARS.CoV.2.S1.mFcTag	feature_id[3].value > threshold=54010.166015625
node_351: feature_name=SARS.CoV.2.S2	feature_id[2].value > threshold=37075.75
node_391: feature_name=SARS.CoV.2.S1.mFcTag	feature_id[3].value <= threshold=55462.833984375
node_392: feature_name=SARS.CoV.2.S1.mFcTag	feature_id[3].value <= threshold=55163.634765625
node_393: feature_name=hCoV.HKU1.NP	feature_id[10].value <= threshold=14561.3330078125
node_394: feature_name=hCoV.HKU1.NP	feature_id[10].value > threshold=6453.333251953125
node_412: feature_name=HuIgG_0.03	feature_id[5].value > threshold=4766.166748046875
node_414: feature_name=MoIgA_0.10	feature_id[13].value > threshold=2918.0
node_416: feature_name=HuIgG_0.03	feature_id[5].value <= threshold=6174.0
Class: healthcare workers within 60 days after vaccination	
Rules_82	passed counts:3
node_0: feature_name=HuIgG_0.10	feature_id[1].value > threshold=19120.166015625
node_112: feature_name=a-HuIgG_0.03	feature_id[8].value <= threshold=19665.666015625
node_113: feature_name=SARS.CoV.2.S1.mFcTag	feature_id[3].value > threshold=54010.166015625
node_351: feature_name=SARS.CoV.2.S2	feature_id[2].value > threshold=37075.75
node_391: feature_name=SARS.CoV.2.S1.mFcTag	feature_id[3].value <= threshold=55462.833984375
node_392: feature_name=SARS.CoV.2.S1.mFcTag	feature_id[3].value <= threshold=55163.634765625
node_393: feature_name=hCoV.HKU1.NP	feature_id[10].value <= threshold=14561.3330078125
node_394: feature_name=hCoV.HKU1.NP	feature_id[10].value <= threshold=6453.333251953125
node_395: feature_name=hCoV.HKU1.NP	feature_id[10].value > threshold=1990.1666259765625

node_407: feature_name=SARS.CoV.2.S1.RBD.mFc	feature_id[0].value <= threshold=48385.75
Class: healthcare workers between 60 and 180 days after vaccination	
Rules_83	passed counts:3
node_0: feature_name=HuIgG_0.10	feature_id[1].value > threshold=19120.166015625
node_112: feature_name=a-HuIgG_0.03	feature_id[8].value <= threshold=19665.666015625
node_113: feature_name=SARS.CoV.2.S1.mFcTag	feature_id[3].value > threshold=54010.166015625
node_351: feature_name=SARS.CoV.2.S2	feature_id[2].value > threshold=37075.75
node_391: feature_name=SARS.CoV.2.S1.mFcTag	feature_id[3].value <= threshold=55462.833984375
node_392: feature_name=SARS.CoV.2.S1.mFcTag	feature_id[3].value <= threshold=55163.634765625
node_393: feature_name=hCoV.HKU1.NP	feature_id[10].value <= threshold=14561.3330078125
node_394: feature_name=hCoV.HKU1.NP	feature_id[10].value <= threshold=6453.333251953125
node_395: feature_name=hCoV.HKU1.NP	feature_id[10].value <= threshold=1990.1666259765625
node_396: feature_name=SARS.CoV.S1.HisTag	feature_id[6].value <= threshold=12607.2998046875
Class: healthcare workers within 60 days after vaccination	
Rules_84	passed counts:3
node_0: feature_name=HuIgG_0.10	feature_id[1].value > threshold=19120.166015625
node_112: feature_name=a-HuIgG_0.03	feature_id[8].value <= threshold=19665.666015625
node_113: feature_name=SARS.CoV.2.S1.mFcTag	feature_id[3].value > threshold=54010.166015625
node_351: feature_name=SARS.CoV.2.S2	feature_id[2].value <= threshold=37075.75
node_352: feature_name=a-HuIgG_0.03	feature_id[8].value > threshold=17721.1669921875
node_386: feature_name=hCoV.HKU1.NP	feature_id[10].value > threshold=4175.4998779296875
Class: healthcare workers within 60 days after vaccination	

Rules_85	passed counts:3
node_0: feature_name=HuIgG_0.10	feature_id[1].value > threshold=19120.166015625
node_112: feature_name=a-HuIgG_0.03	feature_id[8].value <= threshold=19665.666015625
node_113: feature_name=SARS.CoV.2.S1.mFcTag	feature_id[3].value > threshold=54010.166015625
node_351: feature_name=SARS.CoV.2.S2	feature_id[2].value <= threshold=37075.75
node_352: feature_name=a-HuIgG_0.03	feature_id[8].value <= threshold=17721.1669921875
node_353: feature_name=MoIgA_0.10	feature_id[13].value <= threshold=536.0
node_354: feature_name=hCoV.HKU1.NP	feature_id[10].value <= threshold=7098.5
node_355: feature_name=MoIgA_0.10	feature_id[13].value > threshold=462.0
Class: healthcare workers within 60 days after vaccination	
Rules_86	passed counts:3
node_0: feature_name=HuIgG_0.10	feature_id[1].value > threshold=19120.166015625
node_112: feature_name=a-HuIgG_0.03	feature_id[8].value <= threshold=19665.666015625
node_113: feature_name=SARS.CoV.2.S1.mFcTag	feature_id[3].value <= threshold=54010.166015625
node_114: feature_name=SARS.CoV.2.S1+S2	feature_id[12].value > threshold=47258.43359375
node_240: feature_name=HuIgG_0.03	feature_id[5].value > threshold=5455.5
node_266: feature_name=HuIgG_0.10	feature_id[1].value > threshold=24231.0
node_284: feature_name=HuIgG_0.03	feature_id[5].value > threshold=5469.0
node_286: feature_name=HuIgG_0.10	feature_id[1].value <= threshold=30107.666015625
node_287: feature_name=SARS.CoV.2.S1+S2	feature_id[12].value > threshold=47536.224609375
node_291: feature_name=SARS.CoV.2.S2	feature_id[2].value <= threshold=37653.75
node_292: feature_name=SARS.CoV.2.S2	feature_id[2].value <= threshold=36044.29296875
node_293: feature_name=HuIgG_0.03	feature_id[5].value > threshold=6329.5
node_301: feature_name=HuIgG_0.03	feature_id[5].value > threshold=6562.16650390625

node_307: feature_name=Flu.B_Phu/.HA1+HA2	feature_id[11].value <= threshold=54123.5
node_308: feature_name=MoIgA_0.10	feature_id[13].value > threshold=21.166666984558105
node_310: feature_name=hCoV.NL63.S1_S2	feature_id[7].value <= threshold=33291.458984375
node_311: feature_name=SARS.CoV.2.S1+S2	feature_id[12].value > threshold=49482.583984375
Class: healthcare workers between 60 and 180 days after vaccination	
Rules_87	passed counts:3
node_0: feature_name=HuIgG_0.10	feature_id[1].value > threshold=19120.166015625
node_112: feature_name=a-HuIgG_0.03	feature_id[8].value <= threshold=19665.666015625
node_113: feature_name=SARS.CoV.2.S1.mFcTag	feature_id[3].value <= threshold=54010.166015625
node_114: feature_name=SARS.CoV.2.S1+S2	feature_id[12].value > threshold=47258.43359375
node_240: feature_name=HuIgG_0.03	feature_id[5].value > threshold=5455.5
node_266: feature_name=HuIgG_0.10	feature_id[1].value > threshold=24231.0
node_284: feature_name=HuIgG_0.03	feature_id[5].value > threshold=5469.0
node_286: feature_name=HuIgG_0.10	feature_id[1].value <= threshold=30107.666015625
node_287: feature_name=SARS.CoV.2.S1+S2	feature_id[12].value <= threshold=47536.224609375
node_288: feature_name=MoIgA_0.10	feature_id[13].value > threshold=1044.0
Class: healthcare workers between 60 and 180 days after vaccination	
Rules_88	passed counts:3
node_0: feature_name=HuIgG_0.10	feature_id[1].value > threshold=19120.166015625
node_112: feature_name=a-HuIgG_0.03	feature_id[8].value <= threshold=19665.666015625
node_113: feature_name=SARS.CoV.2.S1.mFcTag	feature_id[3].value <= threshold=54010.166015625
node_114: feature_name=SARS.CoV.2.S1+S2	feature_id[12].value > threshold=47258.43359375
node_240: feature_name=HuIgG_0.03	feature_id[5].value > threshold=5455.5

node_266: feature_name=HuIgG_0.10	feature_id[1].value <= threshold=24231.0
node_267: feature_name=hCoV.HKU1.NP	feature_id[10].value > threshold=2123.0000610351562
node_271: feature_name=SARS.CoV.S1.HisTag	feature_id[6].value > threshold=8281.83349609375
node_273: feature_name=Flu.B_Phu/.HA1+HA2	feature_id[11].value <= threshold=51424.19921875
Class: healthcare workers between 60 and 180 days after vaccination	
Rules_89	passed counts:3
node_0: feature_name=HuIgG_0.10	feature_id[1].value > threshold=19120.166015625
node_112: feature_name=a-HuIgG_0.03	feature_id[8].value <= threshold=19665.666015625
node_113: feature_name=SARS.CoV.2.S1.mFcTag	feature_id[3].value <= threshold=54010.166015625
node_114: feature_name=SARS.CoV.2.S1+S2	feature_id[12].value <= threshold=47258.43359375
node_115: feature_name=SARS.CoV.2.S1.RBD.mFc	feature_id[0].value > threshold=301.73333740234375
node_131: feature_name=a-HuIgG_0.03	feature_id[8].value > threshold=12099.83349609375
node_183: feature_name=Flu.B_Phu/.HA1+HA2	feature_id[11].value > threshold=41918.533203125
node_187: feature_name=SARS.CoV.2.Spike.RBD.rFc	feature_id[9].value > threshold=27438.341796875
node_197: feature_name=SARS.CoV.2.S2	feature_id[2].value <= threshold=35177.640625
node_198: feature_name=hCoV.HKU1.NP	feature_id[10].value > threshold=2645.916748046875
node_210: feature_name=Flu.B_Phu/.HA1+HA2	feature_id[11].value <= threshold=54029.166015625
node_211: feature_name=HuIgG_0.10	feature_id[1].value > threshold=21359.5
node_213: feature_name=hCoV.NL63.S1_S2	feature_id[7].value > threshold=32523.4248046875
node_215: feature_name=HuIgG_0.10	feature_id[1].value > threshold=23274.166015625
node_217: feature_name=HuIgG_0.10	feature_id[1].value <= threshold=25231.1669921875
node_218: feature_name=hCoV.HKU1.NP	feature_id[10].value <= threshold=3712.083251953125
Class: healthcare workers within 60 days after vaccination	

Rules_90	passed counts:3
node_0: feature_name=HuIgG_0.10	feature_id[1].value > threshold=19120.166015625
node_112: feature_name=a-HuIgG_0.03	feature_id[8].value <= threshold=19665.666015625
node_113: feature_name=SARS.CoV.2.S1.mFcTag	feature_id[3].value <= threshold=54010.166015625
node_114: feature_name=SARS.CoV.2.S1+S2	feature_id[12].value <= threshold=47258.43359375
node_115: feature_name=SARS.CoV.2.S1.RBD.mFc	feature_id[0].value > threshold=301.73333740234375
node_131: feature_name=a-HuIgG_0.03	feature_id[8].value <= threshold=12099.83349609375
node_132: feature_name=SARS.CoV.2.S2	feature_id[2].value > threshold=8858.0751953125
node_134: feature_name=Flu.B_Phu/.HA1+HA2	feature_id[11].value <= threshold=53839.16796875
node_135: feature_name=hCoV.HKU1.NP	feature_id[10].value > threshold=1010.1666564941406
node_145: feature_name=hCoV.HKU1.NP	feature_id[10].value > threshold=15374.16650390625
node_177: feature_name=a-HuIgG_0.03	feature_id[8].value <= threshold=11866.5
Class: healthcare workers between 60 and 180 days after vaccination	
Rules_91	passed counts:3
node_0: feature_name=HuIgG_0.10	feature_id[1].value > threshold=19120.166015625
node_112: feature_name=a-HuIgG_0.03	feature_id[8].value <= threshold=19665.666015625
node_113: feature_name=SARS.CoV.2.S1.mFcTag	feature_id[3].value <= threshold=54010.166015625
node_114: feature_name=SARS.CoV.2.S1+S2	feature_id[12].value <= threshold=47258.43359375
node_115: feature_name=SARS.CoV.2.S1.RBD.mFc	feature_id[0].value > threshold=301.73333740234375
node_131: feature_name=a-HuIgG_0.03	feature_id[8].value <= threshold=12099.83349609375
node_132: feature_name=SARS.CoV.2.S2	feature_id[2].value > threshold=8858.0751953125
node_134: feature_name=Flu.B_Phu/.HA1+HA2	feature_id[11].value <= threshold=53839.16796875
node_135: feature_name=hCoV.HKU1.NP	feature_id[10].value > threshold=1010.1666564941406
node_145: feature_name=hCoV.HKU1.NP	feature_id[10].value <= threshold=15374.16650390625

node_146: feature_name=SARS.CoV.2.S1.mFcTag	feature_id[3].value > threshold=50683.0
node_168: feature_name=HuIgG_0.10	feature_id[1].value > threshold=20669.833984375
node_170: feature_name=SARS.CoV.2.S1.mFcTag	feature_id[3].value <= threshold=50874.666015625
Class: healthcare workers between 60 and 180 days after vaccination	
Rules_92	passed counts:3
node_0: feature_name=HuIgG_0.10	feature_id[1].value > threshold=19120.166015625
node_112: feature_name=a-HuIgG_0.03	feature_id[8].value <= threshold=19665.666015625
node_113: feature_name=SARS.CoV.2.S1.mFcTag	feature_id[3].value <= threshold=54010.166015625
node_114: feature_name=SARS.CoV.2.S1+S2	feature_id[12].value <= threshold=47258.43359375
node_115: feature_name=SARS.CoV.2.S1.RBD.mFc	feature_id[0].value > threshold=301.73333740234375
node_131: feature_name=a-HuIgG_0.03	feature_id[8].value <= threshold=12099.83349609375
node_132: feature_name=SARS.CoV.2.S2	feature_id[2].value > threshold=8858.0751953125
node_134: feature_name=Flu.B_Phu/.HA1+HA2	feature_id[11].value <= threshold=53839.16796875
node_135: feature_name=hCoV.HKU1.NP	feature_id[10].value > threshold=1010.1666564941406
node_145: feature_name=hCoV.HKU1.NP	feature_id[10].value <= threshold=15374.16650390625
node_146: feature_name=SARS.CoV.2.S1.mFcTag	feature_id[3].value > threshold=50683.0
node_168: feature_name=HuIgG_0.10	feature_id[1].value <= threshold=20669.833984375
Class: healthcare workers between 60 and 180 days after vaccination	
Rules_93	passed counts:3
node_0: feature_name=HuIgG_0.10	feature_id[1].value > threshold=19120.166015625
node_112: feature_name=a-HuIgG_0.03	feature_id[8].value <= threshold=19665.666015625
node_113: feature_name=SARS.CoV.2.S1.mFcTag	feature_id[3].value <= threshold=54010.166015625
node_114: feature_name=SARS.CoV.2.S1+S2	feature_id[12].value <= threshold=47258.43359375

node_115: feature_name=SARS.CoV.2.S1.RBD.mFc	feature_id[0].value > threshold=301.73333740234375
node_131: feature_name=a-HuIgG_0.03	feature_id[8].value <= threshold=12099.83349609375
node_132: feature_name=SARS.CoV.2.S2	feature_id[2].value > threshold=8858.0751953125
node_134: feature_name=Flu.B_Phu/.HA1+HA2	feature_id[11].value <= threshold=53839.16796875
node_135: feature_name=hCoV.HKU1.NP	feature_id[10].value <= threshold=1010.1666564941406
node_136: feature_name=MoIgA_0.10	feature_id[13].value <= threshold=497.8333282470703
node_137: feature_name=MoIgA_0.10	feature_id[13].value <= threshold=153.0
node_138: feature_name=SARS.CoV.S1.HisTag	feature_id[6].value <= threshold=14632.4169921875
Class: healthcare workers between 60 and 180 days after vaccination	
Rules_94	passed counts:3
node_0: feature_name=HuIgG_0.10	feature_id[1].value > threshold=19120.166015625
node_112: feature_name=a-HuIgG_0.03	feature_id[8].value <= threshold=19665.666015625
node_113: feature_name=SARS.CoV.2.S1.mFcTag	feature_id[3].value <= threshold=54010.166015625
node_114: feature_name=SARS.CoV.2.S1+S2	feature_id[12].value <= threshold=47258.43359375
node_115: feature_name=SARS.CoV.2.S1.RBD.mFc	feature_id[0].value <= threshold=301.73333740234375
node_116: feature_name=SARS.CoV.2.S1+S2	feature_id[12].value > threshold=3298.25
node_122: feature_name=SARS.CoV.2.S1.mFcTag	feature_id[3].value > threshold=-1493.1666870117188
node_124: feature_name=hCoV.HKU1.NP	feature_id[10].value <= threshold=11908.138671875
node_125: feature_name=SARS.CoV.2.S1.RBD.mFc	feature_id[0].value > threshold=82.01666641235352
node_127: feature_name=a-HuIgG_0.30	feature_id[4].value > threshold=39543.0
Class: unvaccinated healthcare workers	
Rules_95	passed counts:3
node_0: feature_name=HuIgG_0.10	feature_id[1].value <= threshold=19120.166015625

node_1: feature_name=SARS.CoV.2.S1.mFcTag	feature_id[3].value > threshold=5354.388916015625
node_9: feature_name=a-HuIgG_0.30	feature_id[4].value > threshold=33726.833984375
node_55: feature_name=SARS.CoV.2.S1.mFcTag	feature_id[3].value > threshold=43185.68359375
node_67: feature_name=a-HuIgG_0.03	feature_id[8].value > threshold=10858.0
node_83: feature_name=HuIgG_0.03	feature_id[5].value > threshold=3796.0
node_103: feature_name=SARS.CoV.2.S1+S2	feature_id[12].value > threshold=49967.91796875
node_107: feature_name=hCoV.NL63.S1_S2	feature_id[7].value > threshold=28891.8330078125
Class: healthcare workers within 60 days after vaccination	
Rules_96	passed counts:3
node_0: feature_name=HuIgG_0.10	feature_id[1].value <= threshold=19120.166015625
node_1: feature_name=SARS.CoV.2.S1.mFcTag	feature_id[3].value > threshold=5354.388916015625
node_9: feature_name=a-HuIgG_0.30	feature_id[4].value > threshold=33726.833984375
node_55: feature_name=SARS.CoV.2.S1.mFcTag	feature_id[3].value > threshold=43185.68359375
node_67: feature_name=a-HuIgG_0.03	feature_id[8].value > threshold=10858.0
node_83: feature_name=HuIgG_0.03	feature_id[5].value <= threshold=3796.0
node_84: feature_name=hCoV.NL63.S1_S2	feature_id[7].value > threshold=29814.5
node_96: feature_name=hCoV.NL63.S1_S2	feature_id[7].value > threshold=34078.0
node_98: feature_name=SARS.CoV.S1.HisTag	feature_id[6].value <= threshold=9674.150390625
Class: healthcare workers between 60 and 180 days after vaccination	
Rules_97	passed counts:3
node_0: feature_name=HuIgG_0.10	feature_id[1].value <= threshold=19120.166015625
node_1: feature_name=SARS.CoV.2.S1.mFcTag	feature_id[3].value > threshold=5354.388916015625
node_9: feature_name=a-HuIgG_0.30	feature_id[4].value > threshold=33726.833984375

node_55: feature_name=SARS.CoV.2.S1.mFcTag	feature_id[3].value > threshold=43185.68359375
node_67: feature_name=a-HuIgG_0.03	feature_id[8].value <= threshold=10858.0
node_68: feature_name=MoIgA_0.10	feature_id[13].value > threshold=246.83333587646484
node_76: feature_name=SARS.CoV.2.Spike.RBD.rFc	feature_id[9].value <= threshold=54060.41796875
node_77: feature_name=a-HuIgG_0.03	feature_id[8].value > threshold=10179.0
node_79: feature_name=hCoV.HKU1.NP	feature_id[10].value > threshold=2203.3333129882812
Class: healthcare workers within 60 days after vaccination	
Rules_98	passed counts:3
node_0: feature_name=HuIgG_0.10	feature_id[1].value <= threshold=19120.166015625
node_1: feature_name=SARS.CoV.2.S1.mFcTag	feature_id[3].value > threshold=5354.388916015625
node_9: feature_name=a-HuIgG_0.30	feature_id[4].value > threshold=33726.833984375
node_55: feature_name=SARS.CoV.2.S1.mFcTag	feature_id[3].value <= threshold=43185.68359375
node_56: feature_name=SARS.CoV.S1.HisTag	feature_id[6].value > threshold=1493.4166870117188
node_62: feature_name=SARS.CoV.2.S1+S2	feature_id[12].value <= threshold=22078.1669921875
node_63: feature_name=SARS.CoV.2.S1+S2	feature_id[12].value <= threshold=21810.333984375
Class: healthcare workers over 180 days after vaccination	
Rules_99	passed counts:3
node_0: feature_name=HuIgG_0.10	feature_id[1].value <= threshold=19120.166015625
node_1: feature_name=SARS.CoV.2.S1.mFcTag	feature_id[3].value > threshold=5354.388916015625
node_9: feature_name=a-HuIgG_0.30	feature_id[4].value > threshold=33726.833984375
node_55: feature_name=SARS.CoV.2.S1.mFcTag	feature_id[3].value <= threshold=43185.68359375
node_56: feature_name=SARS.CoV.S1.HisTag	feature_id[6].value <= threshold=1493.4166870117188
node_57: feature_name=SARS.CoV.2.S1.mFcTag	feature_id[3].value > threshold=24427.2880859375

Class: unvaccinated healthcare workers	
Rules_100	passed counts:3
node_0: feature_name=HuIgG_0.10	feature_id[1].value <= threshold=19120.166015625
node_1: feature_name=SARS.CoV.2.S1.mFcTag	feature_id[3].value > threshold=5354.388916015625
node_9: feature_name=a-HuIgG_0.30	feature_id[4].value <= threshold=33726.833984375
node_10: feature_name=HuIgG_0.03	feature_id[5].value > threshold=1023.1666564941406
node_20: feature_name=hCoV.HKU1.NP	feature_id[10].value > threshold=-20.05833387374878
node_22: feature_name=SARS.CoV.S1.HisTag	feature_id[6].value > threshold=400.3000030517578
node_26: feature_name=SARS.CoV.S1.HisTag	feature_id[6].value > threshold=15087.41650390625
node_38: feature_name=hCoV.HKU1.NP	feature_id[10].value <= threshold=348.6666564941406
node_39: feature_name=hCoV.NL63.S1_S2	feature_id[7].value > threshold=26557.333984375
Class: healthcare workers within 60 days after vaccination	
Rules_101	passed counts:3
node_0: feature_name=HuIgG_0.10	feature_id[1].value <= threshold=19120.166015625
node_1: feature_name=SARS.CoV.2.S1.mFcTag	feature_id[3].value > threshold=5354.388916015625
node_9: feature_name=a-HuIgG_0.30	feature_id[4].value <= threshold=33726.833984375
node_10: feature_name=HuIgG_0.03	feature_id[5].value > threshold=1023.1666564941406
node_20: feature_name=hCoV.HKU1.NP	feature_id[10].value > threshold=-20.05833387374878
node_22: feature_name=SARS.CoV.S1.HisTag	feature_id[6].value > threshold=400.3000030517578
node_26: feature_name=SARS.CoV.S1.HisTag	feature_id[6].value <= threshold=15087.41650390625
node_27: feature_name=SARS.CoV.2.S1.RBD.mFc	feature_id[0].value > threshold=43991.083984375
node_35: feature_name=SARS.CoV.S1.HisTag	feature_id[6].value > threshold=5744.75
Class: healthcare workers between 60 and 180 days after vaccination	

Rules_102	passed counts:2
node_0: feature_name=HuIgG_0.10	feature_id[1].value > threshold=19120.166015625
node_112: feature_name=a-HuIgG_0.03	feature_id[8].value > threshold=19665.666015625
node_428: feature_name=SARS.CoV.2.Spike.RBD.rFc	feature_id[9].value <= threshold=55147.1484375
node_429: feature_name=a-HuIgG_0.30	feature_id[4].value > threshold=51289.166015625
node_437: feature_name=HuIgG_0.10	feature_id[1].value > threshold=25959.0
node_441: feature_name=hCoV.NL63.S1_S2	feature_id[7].value > threshold=43930.5234375
Class: healthcare workers within 60 days after vaccination	
Rules_103	passed counts:2
node_0: feature_name=HuIgG_0.10	feature_id[1].value > threshold=19120.166015625
node_112: feature_name=a-HuIgG_0.03	feature_id[8].value > threshold=19665.666015625
node_428: feature_name=SARS.CoV.2.Spike.RBD.rFc	feature_id[9].value <= threshold=55147.1484375
node_429: feature_name=a-HuIgG_0.30	feature_id[4].value > threshold=51289.166015625
node_437: feature_name=HuIgG_0.10	feature_id[1].value > threshold=25959.0
node_441: feature_name=hCoV.NL63.S1_S2	feature_id[7].value <= threshold=43930.5234375
node_442: feature_name=a-HuIgG_0.03	feature_id[8].value <= threshold=23272.1669921875
node_443: feature_name=hCoV.HKU1.NP	feature_id[10].value <= threshold=1474.3833312988281
node_444: feature_name=a-HuIgG_0.03	feature_id[8].value > threshold=22815.1669921875
Class: healthcare workers over 180 days after vaccination	
Rules_104	passed counts:2
node_0: feature_name=HuIgG_0.10	feature_id[1].value > threshold=19120.166015625
node_112: feature_name=a-HuIgG_0.03	feature_id[8].value <= threshold=19665.666015625

node_113: feature_name=SARS.CoV.2.S1.mFcTag	feature_id[3].value > threshold=54010.166015625
node_351: feature_name=SARS.CoV.2.S2	feature_id[2].value > threshold=37075.75
node_391: feature_name=SARS.CoV.2.S1.mFcTag	feature_id[3].value <= threshold=55462.833984375
node_392: feature_name=SARS.CoV.2.S1.mFcTag	feature_id[3].value <= threshold=55163.634765625
node_393: feature_name=hCoV.HKU1.NP	feature_id[10].value <= threshold=14561.3330078125
node_394: feature_name=hCoV.HKU1.NP	feature_id[10].value > threshold=6453.333251953125
node_412: feature_name=HuIgG_0.03	feature_id[5].value <= threshold=4766.166748046875
Class: healthcare workers within 60 days after vaccination	
Rules_105	passed counts:2
node_0: feature_name=HuIgG_0.10	feature_id[1].value > threshold=19120.166015625
node_112: feature_name=a-HuIgG_0.03	feature_id[8].value <= threshold=19665.666015625
node_113: feature_name=SARS.CoV.2.S1.mFcTag	feature_id[3].value > threshold=54010.166015625
node_351: feature_name=SARS.CoV.2.S2	feature_id[2].value <= threshold=37075.75
node_352: feature_name=a-HuIgG_0.03	feature_id[8].value > threshold=17721.1669921875
node_386: feature_name=hCoV.HKU1.NP	feature_id[10].value <= threshold=4175.4998779296875
node_387: feature_name=hCoV.HKU1.NP	feature_id[10].value <= threshold=3016.9666748046875
Class: healthcare workers over 180 days after vaccination	
Rules_106	passed counts:2
node_0: feature_name=HuIgG_0.10	feature_id[1].value > threshold=19120.166015625
node_112: feature_name=a-HuIgG_0.03	feature_id[8].value <= threshold=19665.666015625
node_113: feature_name=SARS.CoV.2.S1.mFcTag	feature_id[3].value > threshold=54010.166015625
node_351: feature_name=SARS.CoV.2.S2	feature_id[2].value <= threshold=37075.75
node_352: feature_name=a-HuIgG_0.03	feature_id[8].value <= threshold=17721.1669921875

node_353: feature_name=MoIgA_0.10	feature_id[13].value > threshold=536.0
node_367: feature_name=HuIgG_0.03	feature_id[5].value <= threshold=5634.333251953125
node_368: feature_name=SARS.CoV.2.S1+S2	feature_id[12].value <= threshold=52432.75
node_369: feature_name=SARS.CoV.S1.HisTag	feature_id[6].value > threshold=17773.60009765625
Class: healthcare workers over 180 days after vaccination	
Rules_107	passed counts:2
node_0: feature_name=HuIgG_0.10	feature_id[1].value > threshold=19120.166015625
node_112: feature_name=a-HuIgG_0.03	feature_id[8].value <= threshold=19665.666015625
node_113: feature_name=SARS.CoV.2.S1.mFcTag	feature_id[3].value > threshold=54010.166015625
node_351: feature_name=SARS.CoV.2.S2	feature_id[2].value <= threshold=37075.75
node_352: feature_name=a-HuIgG_0.03	feature_id[8].value <= threshold=17721.1669921875
node_353: feature_name=MoIgA_0.10	feature_id[13].value <= threshold=536.0
node_354: feature_name=hCoV.HKU1.NP	feature_id[10].value <= threshold=7098.5
node_355: feature_name=MoIgA_0.10	feature_id[13].value <= threshold=462.0
node_356: feature_name=hCoV.HKU1.NP	feature_id[10].value <= threshold=2790.583251953125
node_357: feature_name=hCoV.NL63.S1_S2	feature_id[7].value > threshold=36099.0
node_359: feature_name=hCoV.HKU1.NP	feature_id[10].value <= threshold=1348.6666870117188
node_360: feature_name=SARS.CoV.S1.HisTag	feature_id[6].value > threshold=5850.833251953125
Class: healthcare workers between 60 and 180 days after vaccination	
Rules_108	passed counts:2
node_0: feature_name=HuIgG_0.10	feature_id[1].value > threshold=19120.166015625
node_112: feature_name=a-HuIgG_0.03	feature_id[8].value <= threshold=19665.666015625
node_113: feature_name=SARS.CoV.2.S1.mFcTag	feature_id[3].value <= threshold=54010.166015625

node_114: feature_name=SARS.CoV.2.S1+S2	feature_id[12].value > threshold=47258.43359375
node_240: feature_name=HuIgG_0.03	feature_id[5].value > threshold=5455.5
node_266: feature_name=HuIgG_0.10	feature_id[1].value > threshold=24231.0
node_284: feature_name=HuIgG_0.03	feature_id[5].value > threshold=5469.0
node_286: feature_name=HuIgG_0.10	feature_id[1].value > threshold=30107.666015625
Class: healthcare workers between 60 and 180 days after vaccination	
Rules_109	passed counts:2
node_0: feature_name=HuIgG_0.10	feature_id[1].value > threshold=19120.166015625
node_112: feature_name=a-HuIgG_0.03	feature_id[8].value <= threshold=19665.666015625
node_113: feature_name=SARS.CoV.2.S1.mFcTag	feature_id[3].value <= threshold=54010.166015625
node_114: feature_name=SARS.CoV.2.S1+S2	feature_id[12].value > threshold=47258.43359375
node_240: feature_name=HuIgG_0.03	feature_id[5].value > threshold=5455.5
node_266: feature_name=HuIgG_0.10	feature_id[1].value > threshold=24231.0
node_284: feature_name=HuIgG_0.03	feature_id[5].value > threshold=5469.0
node_286: feature_name=HuIgG_0.10	feature_id[1].value <= threshold=30107.666015625
node_287: feature_name=SARS.CoV.2.S1+S2	feature_id[12].value > threshold=47536.224609375
node_291: feature_name=SARS.CoV.2.S2	feature_id[2].value > threshold=37653.75
node_329: feature_name=SARS.CoV.2.S1.mFcTag	feature_id[3].value <= threshold=53346.333984375
node_330: feature_name=Flu.B_Phu/.HA1+HA2	feature_id[11].value <= threshold=53733.0
node_331: feature_name=MoIgA_0.10	feature_id[13].value > threshold=10.833333015441895
node_335: feature_name=a-HuIgG_0.03	feature_id[8].value <= threshold=11890.0
node_336: feature_name=HuIgG_0.10	feature_id[1].value <= threshold=25504.333984375
Class: healthcare workers within 60 days after vaccination	

Rules_110	passed counts:2
node_0: feature_name=HuIgG_0.10	feature_id[1].value > threshold=19120.166015625
node_112: feature_name=a-HuIgG_0.03	feature_id[8].value <= threshold=19665.666015625
node_113: feature_name=SARS.CoV.2.S1.mFcTag	feature_id[3].value <= threshold=54010.166015625
node_114: feature_name=SARS.CoV.2.S1+S2	feature_id[12].value > threshold=47258.43359375
node_240: feature_name=HuIgG_0.03	feature_id[5].value > threshold=5455.5
node_266: feature_name=HuIgG_0.10	feature_id[1].value > threshold=24231.0
node_284: feature_name=HuIgG_0.03	feature_id[5].value > threshold=5469.0
node_286: feature_name=HuIgG_0.10	feature_id[1].value <= threshold=30107.666015625
node_287: feature_name=SARS.CoV.2.S1+S2	feature_id[12].value > threshold=47536.224609375
node_291: feature_name=SARS.CoV.2.S2	feature_id[2].value <= threshold=37653.75
node_292: feature_name=SARS.CoV.2.S2	feature_id[2].value > threshold=36044.29296875
node_322: feature_name=hCoV.HKU1.NP	feature_id[10].value <= threshold=2383.1666870117188
Class: healthcare workers within 60 days after vaccination	
Rules_111	passed counts:2
node_0: feature_name=HuIgG_0.10	feature_id[1].value > threshold=19120.166015625
node_112: feature_name=a-HuIgG_0.03	feature_id[8].value <= threshold=19665.666015625
node_113: feature_name=SARS.CoV.2.S1.mFcTag	feature_id[3].value <= threshold=54010.166015625
node_114: feature_name=SARS.CoV.2.S1+S2	feature_id[12].value > threshold=47258.43359375
node_240: feature_name=HuIgG_0.03	feature_id[5].value > threshold=5455.5
node_266: feature_name=HuIgG_0.10	feature_id[1].value > threshold=24231.0
node_284: feature_name=HuIgG_0.03	feature_id[5].value > threshold=5469.0
node_286: feature_name=HuIgG_0.10	feature_id[1].value <= threshold=30107.666015625
node_287: feature_name=SARS.CoV.2.S1+S2	feature_id[12].value > threshold=47536.224609375

node_291: feature_name=SARS.CoV.2.S2	feature_id[2].value <= threshold=37653.75
node_292: feature_name=SARS.CoV.2.S2	feature_id[2].value <= threshold=36044.29296875
node_293: feature_name=HuIgG_0.03	feature_id[5].value > threshold=6329.5
node_301: feature_name=HuIgG_0.03	feature_id[5].value > threshold=6562.16650390625
node_307: feature_name=Flu.B_Phu/.HA1+HA2	feature_id[11].value > threshold=54123.5
node_319: feature_name=hCoV.NL63.S1_S2	feature_id[7].value <= threshold=43715.833984375
Class: healthcare workers between 60 and 180 days after vaccination	
Rules_112	passed counts:2
node_0: feature_name=HuIgG_0.10	feature_id[1].value > threshold=19120.166015625
node_112: feature_name=a-HuIgG_0.03	feature_id[8].value <= threshold=19665.666015625
node_113: feature_name=SARS.CoV.2.S1.mFcTag	feature_id[3].value <= threshold=54010.166015625
node_114: feature_name=SARS.CoV.2.S1+S2	feature_id[12].value > threshold=47258.43359375
node_240: feature_name=HuIgG_0.03	feature_id[5].value > threshold=5455.5
node_266: feature_name=HuIgG_0.10	feature_id[1].value > threshold=24231.0
node_284: feature_name=HuIgG_0.03	feature_id[5].value > threshold=5469.0
node_286: feature_name=HuIgG_0.10	feature_id[1].value <= threshold=30107.666015625
node_287: feature_name=SARS.CoV.2.S1+S2	feature_id[12].value > threshold=47536.224609375
node_291: feature_name=SARS.CoV.2.S2	feature_id[2].value <= threshold=37653.75
node_292: feature_name=SARS.CoV.2.S2	feature_id[2].value <= threshold=36044.29296875
node_293: feature_name=HuIgG_0.03	feature_id[5].value > threshold=6329.5
node_301: feature_name=HuIgG_0.03	feature_id[5].value > threshold=6562.16650390625
node_307: feature_name=Flu.B_Phu/.HA1+HA2	feature_id[11].value <= threshold=54123.5
node_308: feature_name=MoIgA_0.10	feature_id[13].value > threshold=21.166666984558105
node_310: feature_name=hCoV.NL63.S1_S2	feature_id[7].value > threshold=33291.458984375

node_314: feature_name=SARS.CoV.2.S1.mFcTag	feature_id[3].value <= threshold=50604.0
node_315: feature_name=SARS.CoV.2.Spike.RBD.rFc	feature_id[9].value <= threshold=43138.767578125
Class: healthcare workers within 60 days after vaccination	
Rules_113	passed counts:2
node_0: feature_name=HuIgG_0.10	feature_id[1].value > threshold=19120.166015625
node_112: feature_name=a-HuIgG_0.03	feature_id[8].value <= threshold=19665.666015625
node_113: feature_name=SARS.CoV.2.S1.mFcTag	feature_id[3].value <= threshold=54010.166015625
node_114: feature_name=SARS.CoV.2.S1+S2	feature_id[12].value > threshold=47258.43359375
node_240: feature_name=HuIgG_0.03	feature_id[5].value > threshold=5455.5
node_266: feature_name=HuIgG_0.10	feature_id[1].value > threshold=24231.0
node_284: feature_name=HuIgG_0.03	feature_id[5].value > threshold=5469.0
node_286: feature_name=HuIgG_0.10	feature_id[1].value <= threshold=30107.666015625
node_287: feature_name=SARS.CoV.2.S1+S2	feature_id[12].value > threshold=47536.224609375
node_291: feature_name=SARS.CoV.2.S2	feature_id[2].value <= threshold=37653.75
node_292: feature_name=SARS.CoV.2.S2	feature_id[2].value <= threshold=36044.29296875
node_293: feature_name=HuIgG_0.03	feature_id[5].value > threshold=6329.5
node_301: feature_name=HuIgG_0.03	feature_id[5].value <= threshold=6562.16650390625
node_302: feature_name=Flu.B_Phu/.HA1+HA2	feature_id[11].value > threshold=53338.533203125
node_304: feature_name=hCoV.HKU1.NP	feature_id[10].value <= threshold=12278.249755859375
Class: healthcare workers within 60 days after vaccination	
Rules_114	passed counts:2
node_0: feature_name=HuIgG_0.10	feature_id[1].value > threshold=19120.166015625
node_112: feature_name=a-HuIgG_0.03	feature_id[8].value <= threshold=19665.666015625

node_113: feature_name=SARS.CoV.2.S1.mFcTag	feature_id[3].value <= threshold=54010.166015625
node_114: feature_name=SARS.CoV.2.S1+S2	feature_id[12].value > threshold=47258.43359375
node_240: feature_name=HuIgG_0.03	feature_id[5].value > threshold=5455.5
node_266: feature_name=HuIgG_0.10	feature_id[1].value > threshold=24231.0
node_284: feature_name=HuIgG_0.03	feature_id[5].value <= threshold=5469.0
Class: healthcare workers between 60 and 180 days after vaccination	
Rules_115	passed counts:2
node_0: feature_name=HuIgG_0.10	feature_id[1].value > threshold=19120.166015625
node_112: feature_name=a-HuIgG_0.03	feature_id[8].value <= threshold=19665.666015625
node_113: feature_name=SARS.CoV.2.S1.mFcTag	feature_id[3].value <= threshold=54010.166015625
node_114: feature_name=SARS.CoV.2.S1+S2	feature_id[12].value > threshold=47258.43359375
node_240: feature_name=HuIgG_0.03	feature_id[5].value > threshold=5455.5
node_266: feature_name=HuIgG_0.10	feature_id[1].value <= threshold=24231.0
node_267: feature_name=hCoV.HKU1.NP	feature_id[10].value > threshold=2123.0000610351562
node_271: feature_name=SARS.CoV.S1.HisTag	feature_id[6].value > threshold=8281.83349609375
node_273: feature_name=Flu.B_Phu/.HA1+HA2	feature_id[11].value > threshold=51424.19921875
node_275: feature_name=a-HuIgG_0.30	feature_id[4].value > threshold=41169.833984375
node_277: feature_name=a-HuIgG_0.30	feature_id[4].value > threshold=42934.333984375
node_279: feature_name=a-HuIgG_0.03	feature_id[8].value > threshold=17284.5
Class: healthcare workers between 60 and 180 days after vaccination	
Rules_116	passed counts:2
node_0: feature_name=HuIgG_0.10	feature_id[1].value > threshold=19120.166015625
node_112: feature_name=a-HuIgG_0.03	feature_id[8].value <= threshold=19665.666015625

node_113: feature_name=SARS.CoV.2.S1.mFcTag	feature_id[3].value <= threshold=54010.166015625
node_114: feature_name=SARS.CoV.2.S1+S2	feature_id[12].value > threshold=47258.43359375
node_240: feature_name=HuIgG_0.03	feature_id[5].value > threshold=5455.5
node_266: feature_name=HuIgG_0.10	feature_id[1].value <= threshold=24231.0
node_267: feature_name=hCoV.HKU1.NP	feature_id[10].value <= threshold=2123.0000610351562
node_268: feature_name=hCoV.NL63.S1_S2	feature_id[7].value <= threshold=25279.30859375
Class: healthcare workers over 180 days after vaccination	
Rules_117	passed counts:2
node_0: feature_name=HuIgG_0.10	feature_id[1].value > threshold=19120.166015625
node_112: feature_name=a-HuIgG_0.03	feature_id[8].value <= threshold=19665.666015625
node_113: feature_name=SARS.CoV.2.S1.mFcTag	feature_id[3].value <= threshold=54010.166015625
node_114: feature_name=SARS.CoV.2.S1+S2	feature_id[12].value <= threshold=47258.43359375
node_115: feature_name=SARS.CoV.2.S1.RBD.mFc	feature_id[0].value > threshold=301.73333740234375
node_131: feature_name=a-HuIgG_0.03	feature_id[8].value > threshold=12099.83349609375
node_183: feature_name=Flu.B_Phu/.HA1+HA2	feature_id[11].value > threshold=41918.533203125
node_187: feature_name=SARS.CoV.2.Spike.RBD.rFc	feature_id[9].value > threshold=27438.341796875
node_197: feature_name=SARS.CoV.2.S2	feature_id[2].value <= threshold=35177.640625
node_198: feature_name=hCoV.HKU1.NP	feature_id[10].value <= threshold=2645.916748046875
node_199: feature_name=hCoV.HKU1.NP	feature_id[10].value > threshold=666.6666870117188
node_203: feature_name=HuIgG_0.10	feature_id[1].value > threshold=24756.5
node_205: feature_name=SARS.CoV.2.S1+S2	feature_id[12].value > threshold=44218.416015625
node_207: feature_name=SARS.CoV.2.Spike.RBD.rFc	feature_id[9].value > threshold=46720.900390625
Class: healthcare workers between 60 and 180 days after vaccination	

Rules_118	passed counts:2
node_0: feature_name=HuIgG_0.10	feature_id[1].value > threshold=19120.166015625
node_112: feature_name=a-HuIgG_0.03	feature_id[8].value <= threshold=19665.666015625
node_113: feature_name=SARS.CoV.2.S1.mFcTag	feature_id[3].value <= threshold=54010.166015625
node_114: feature_name=SARS.CoV.2.S1+S2	feature_id[12].value <= threshold=47258.43359375
node_115: feature_name=SARS.CoV.2.S1.RBD.mFc	feature_id[0].value > threshold=301.73333740234375
node_131: feature_name=a-HuIgG_0.03	feature_id[8].value > threshold=12099.83349609375
node_183: feature_name=Flu.B_Phu/.HA1+HA2	feature_id[11].value > threshold=41918.533203125
node_187: feature_name=SARS.CoV.2.Spike.RBD.rFc	feature_id[9].value > threshold=27438.341796875
node_197: feature_name=SARS.CoV.2.S2	feature_id[2].value <= threshold=35177.640625
node_198: feature_name=hCoV.HKU1.NP	feature_id[10].value <= threshold=2645.916748046875
node_199: feature_name=hCoV.HKU1.NP	feature_id[10].value <= threshold=666.6666870117188
node_200: feature_name=hCoV.HKU1.NP	feature_id[10].value > threshold=613.4166564941406
Class: healthcare workers within 60 days after vaccination	
Rules_119	passed counts:2
node_0: feature_name=HuIgG_0.10	feature_id[1].value > threshold=19120.166015625
node_112: feature_name=a-HuIgG_0.03	feature_id[8].value <= threshold=19665.666015625
node_113: feature_name=SARS.CoV.2.S1.mFcTag	feature_id[3].value <= threshold=54010.166015625
node_114: feature_name=SARS.CoV.2.S1+S2	feature_id[12].value <= threshold=47258.43359375
node_115: feature_name=SARS.CoV.2.S1.RBD.mFc	feature_id[0].value > threshold=301.73333740234375
node_131: feature_name=a-HuIgG_0.03	feature_id[8].value > threshold=12099.83349609375
node_183: feature_name=Flu.B_Phu/.HA1+HA2	feature_id[11].value > threshold=41918.533203125
node_187: feature_name=SARS.CoV.2.Spike.RBD.rFc	feature_id[9].value > threshold=27438.341796875
node_197: feature_name=SARS.CoV.2.S2	feature_id[2].value <= threshold=35177.640625

node_198: feature_name=hCoV.HKU1.NP	feature_id[10].value <= threshold=2645.916748046875
node_199: feature_name=hCoV.HKU1.NP	feature_id[10].value <= threshold=666.6666870117188
node_200: feature_name=hCoV.HKU1.NP	feature_id[10].value <= threshold=613.4166564941406
Class: healthcare workers over 180 days after vaccination	
Rules_120	passed counts:2
node_0: feature_name=HuIgG_0.10	feature_id[1].value > threshold=19120.166015625
node_112: feature_name=a-HuIgG_0.03	feature_id[8].value <= threshold=19665.666015625
node_113: feature_name=SARS.CoV.2.S1.mFcTag	feature_id[3].value <= threshold=54010.166015625
node_114: feature_name=SARS.CoV.2.S1+S2	feature_id[12].value <= threshold=47258.43359375
node_115: feature_name=SARS.CoV.2.S1.RBD.mFc	feature_id[0].value > threshold=301.73333740234375
node_131: feature_name=a-HuIgG_0.03	feature_id[8].value <= threshold=12099.83349609375
node_132: feature_name=SARS.CoV.2.S2	feature_id[2].value > threshold=8858.0751953125
node_134: feature_name=Flu.B_Phu/.HA1+HA2	feature_id[11].value <= threshold=53839.16796875
node_135: feature_name=hCoV.HKU1.NP	feature_id[10].value > threshold=1010.1666564941406
node_145: feature_name=hCoV.HKU1.NP	feature_id[10].value <= threshold=15374.16650390625
node_146: feature_name=SARS.CoV.2.S1.mFcTag	feature_id[3].value > threshold=50683.0
node_168: feature_name=HuIgG_0.10	feature_id[1].value > threshold=20669.833984375
node_170: feature_name=SARS.CoV.2.S1.mFcTag	feature_id[3].value > threshold=50874.666015625
node_172: feature_name=SARS.CoV.S1.HisTag	feature_id[6].value <= threshold=3796.25
node_173: feature_name=a-HuIgG_0.03	feature_id[8].value > threshold=10618.0
Class: healthcare workers between 60 and 180 days after vaccination	
Rules_121	passed counts:2
node_0: feature_name=HuIgG_0.10	feature_id[1].value > threshold=19120.166015625

node_112: feature_name=a-HuIgG_0.03	feature_id[8].value <= threshold=19665.666015625
node_113: feature_name=SARS.CoV.2.S1.mFcTag	feature_id[3].value <= threshold=54010.166015625
node_114: feature_name=SARS.CoV.2.S1+S2	feature_id[12].value <= threshold=47258.43359375
node_115: feature_name=SARS.CoV.2.S1.RBD.mFc	feature_id[0].value > threshold=301.73333740234375
node_131: feature_name=a-HuIgG_0.03	feature_id[8].value <= threshold=12099.83349609375
node_132: feature_name=SARS.CoV.2.S2	feature_id[2].value > threshold=8858.0751953125
node_134: feature_name=Flu.B_Phu/.HA1+HA2	feature_id[11].value <= threshold=53839.16796875
node_135: feature_name=hCoV.HKU1.NP	feature_id[10].value > threshold=1010.1666564941406
node_145: feature_name=hCoV.HKU1.NP	feature_id[10].value <= threshold=15374.16650390625
node_146: feature_name=SARS.CoV.2.S1.mFcTag	feature_id[3].value <= threshold=50683.0
node_147: feature_name=SARS.CoV.2.Spike.RBD.rFc	feature_id[9].value <= threshold=45822.583984375
node_148: feature_name=SARS.CoV.2.S2	feature_id[2].value > threshold=10212.125
node_154: feature_name=Flu.B_Phu/.HA1+HA2	feature_id[11].value <= threshold=49488.1171875
node_155: feature_name=MoIgA_0.10	feature_id[13].value <= threshold=177.66666412353516
Class: healthcare workers between 60 and 180 days after vaccination	
Rules_122	passed counts:2
node_0: feature_name=HuIgG_0.10	feature_id[1].value > threshold=19120.166015625
node_112: feature_name=a-HuIgG_0.03	feature_id[8].value <= threshold=19665.666015625
node_113: feature_name=SARS.CoV.2.S1.mFcTag	feature_id[3].value <= threshold=54010.166015625
node_114: feature_name=SARS.CoV.2.S1+S2	feature_id[12].value <= threshold=47258.43359375
node_115: feature_name=SARS.CoV.2.S1.RBD.mFc	feature_id[0].value > threshold=301.73333740234375
node_131: feature_name=a-HuIgG_0.03	feature_id[8].value <= threshold=12099.83349609375
node_132: feature_name=SARS.CoV.2.S2	feature_id[2].value > threshold=8858.0751953125
node_134: feature_name=Flu.B_Phu/.HA1+HA2	feature_id[11].value <= threshold=53839.16796875

node_135: feature_name=hCoV.HKU1.NP	feature_id[10].value > threshold=1010.1666564941406
node_145: feature_name=hCoV.HKU1.NP	feature_id[10].value <= threshold=15374.16650390625
node_146: feature_name=SARS.CoV.2.S1.mFcTag	feature_id[3].value <= threshold=50683.0
node_147: feature_name=SARS.CoV.2.Spike.RBD.rFc	feature_id[9].value <= threshold=45822.583984375
node_148: feature_name=SARS.CoV.2.S2	feature_id[2].value <= threshold=10212.125
node_149: feature_name=HuIgG_0.03	feature_id[5].value > threshold=4160.666748046875
node_151: feature_name=SARS.CoV.2.Spike.RBD.rFc	feature_id[9].value > threshold=20605.1162109375
Class: healthcare workers between 60 and 180 days after vaccination	
Rules_123	passed counts:2
node_0: feature_name=HuIgG_0.10	feature_id[1].value > threshold=19120.166015625
node_112: feature_name=a-HuIgG_0.03	feature_id[8].value <= threshold=19665.666015625
node_113: feature_name=SARS.CoV.2.S1.mFcTag	feature_id[3].value <= threshold=54010.166015625
node_114: feature_name=SARS.CoV.2.S1+S2	feature_id[12].value <= threshold=47258.43359375
node_115: feature_name=SARS.CoV.2.S1.RBD.mFc	feature_id[0].value > threshold=301.73333740234375
node_131: feature_name=a-HuIgG_0.03	feature_id[8].value <= threshold=12099.83349609375
node_132: feature_name=SARS.CoV.2.S2	feature_id[2].value > threshold=8858.0751953125
node_134: feature_name=Flu.B_Phu/.HA1+HA2	feature_id[11].value <= threshold=53839.16796875
node_135: feature_name=hCoV.HKU1.NP	feature_id[10].value > threshold=1010.1666564941406
node_145: feature_name=hCoV.HKU1.NP	feature_id[10].value <= threshold=15374.16650390625
node_146: feature_name=SARS.CoV.2.S1.mFcTag	feature_id[3].value <= threshold=50683.0
node_147: feature_name=SARS.CoV.2.Spike.RBD.rFc	feature_id[9].value <= threshold=45822.583984375
node_148: feature_name=SARS.CoV.2.S2	feature_id[2].value <= threshold=10212.125
node_149: feature_name=HuIgG_0.03	feature_id[5].value <= threshold=4160.666748046875
Class: healthcare workers within 60 days after vaccination	

Rules_124	passed counts:2
node_0: feature_name=HuIgG_0.10	feature_id[1].value > threshold=19120.166015625
node_112: feature_name=a-HuIgG_0.03	feature_id[8].value <= threshold=19665.666015625
node_113: feature_name=SARS.CoV.2.S1.mFcTag	feature_id[3].value <= threshold=54010.166015625
node_114: feature_name=SARS.CoV.2.S1+S2	feature_id[12].value <= threshold=47258.43359375
node_115: feature_name=SARS.CoV.2.S1.RBD.mFc	feature_id[0].value <= threshold=301.73333740234375
node_116: feature_name=SARS.CoV.2.S1+S2	feature_id[12].value > threshold=3298.25
node_122: feature_name=SARS.CoV.2.S1.mFcTag	feature_id[3].value > threshold=-1493.1666870117188
node_124: feature_name=hCoV.HKU1.NP	feature_id[10].value > threshold=11908.138671875
Class: healthcare workers within 60 days after vaccination	
Rules_125	passed counts:2
node_0: feature_name=HuIgG_0.10	feature_id[1].value > threshold=19120.166015625
node_112: feature_name=a-HuIgG_0.03	feature_id[8].value <= threshold=19665.666015625
node_113: feature_name=SARS.CoV.2.S1.mFcTag	feature_id[3].value <= threshold=54010.166015625
node_114: feature_name=SARS.CoV.2.S1+S2	feature_id[12].value <= threshold=47258.43359375
node_115: feature_name=SARS.CoV.2.S1.RBD.mFc	feature_id[0].value <= threshold=301.73333740234375
node_116: feature_name=SARS.CoV.2.S1+S2	feature_id[12].value > threshold=3298.25
node_122: feature_name=SARS.CoV.2.S1.mFcTag	feature_id[3].value > threshold=-1493.1666870117188
node_124: feature_name=hCoV.HKU1.NP	feature_id[10].value <= threshold=11908.138671875
node_125: feature_name=SARS.CoV.2.S1.RBD.mFc	feature_id[0].value > threshold=82.01666641235352
node_127: feature_name=a-HuIgG_0.30	feature_id[4].value <= threshold=39543.0
Class: healthcare workers within 60 days after vaccination	

Rules_126	passed counts:2
node_0: feature_name=HuIgG_0.10	feature_id[1].value > threshold=19120.166015625
node_112: feature_name=a-HuIgG_0.03	feature_id[8].value <= threshold=19665.666015625
node_113: feature_name=SARS.CoV.2.S1.mFcTag	feature_id[3].value <= threshold=54010.166015625
node_114: feature_name=SARS.CoV.2.S1+S2	feature_id[12].value <= threshold=47258.43359375
node_115: feature_name=SARS.CoV.2.S1.RBD.mFc	feature_id[0].value <= threshold=301.73333740234375
node_116: feature_name=SARS.CoV.2.S1+S2	feature_id[12].value > threshold=3298.25
node_122: feature_name=SARS.CoV.2.S1.mFcTag	feature_id[3].value <= threshold=-1493.1666870117188
Class: healthcare workers within 60 days after vaccination	
Rules_127	passed counts:2
node_0: feature_name=HuIgG_0.10	feature_id[1].value <= threshold=19120.166015625
node_1: feature_name=SARS.CoV.2.S1.mFcTag	feature_id[3].value > threshold=5354.388916015625
node_9: feature_name=a-HuIgG_0.30	feature_id[4].value > threshold=33726.833984375
node_55: feature_name=SARS.CoV.2.S1.mFcTag	feature_id[3].value > threshold=43185.68359375
node_67: feature_name=a-HuIgG_0.03	feature_id[8].value <= threshold=10858.0
node_68: feature_name=MoIgA_0.10	feature_id[13].value > threshold=246.83333587646484
node_76: feature_name=SARS.CoV.2.Spike.RBD.rFc	feature_id[9].value <= threshold=54060.41796875
node_77: feature_name=a-HuIgG_0.03	feature_id[8].value > threshold=10179.0
node_79: feature_name=hCoV.HKU1.NP	feature_id[10].value <= threshold=2203.3333129882812
Class: healthcare workers between 60 and 180 days after vaccination	
Rules_128	passed counts:2
node_0: feature_name=HuIgG_0.10	feature_id[1].value <= threshold=19120.166015625
node_1: feature_name=SARS.CoV.2.S1.mFcTag	feature_id[3].value > threshold=5354.388916015625

node_9: feature_name=a-HuIgG_0.30	feature_id[4].value > threshold=33726.833984375
node_55: feature_name=SARS.CoV.2.S1.mFcTag	feature_id[3].value > threshold=43185.68359375
node_67: feature_name=a-HuIgG_0.03	feature_id[8].value <= threshold=10858.0
node_68: feature_name=MoIgA_0.10	feature_id[13].value <= threshold=246.83333587646484
node_69: feature_name=hCoV.HKU1.NP	feature_id[10].value <= threshold=1960.7500610351562
node_70: feature_name=hCoV.HKU1.NP	feature_id[10].value > threshold=1023.75
Class: healthcare workers within 60 days after vaccination	
Rules_129	passed counts:2
node_0: feature_name=HuIgG_0.10	feature_id[1].value <= threshold=19120.166015625
node_1: feature_name=SARS.CoV.2.S1.mFcTag	feature_id[3].value > threshold=5354.388916015625
node_9: feature_name=a-HuIgG_0.30	feature_id[4].value <= threshold=33726.833984375
node_10: feature_name=HuIgG_0.03	feature_id[5].value > threshold=1023.1666564941406
node_20: feature_name=hCoV.HKU1.NP	feature_id[10].value > threshold=-20.05833387374878
node_22: feature_name=SARS.CoV.S1.HisTag	feature_id[6].value > threshold=400.3000030517578
node_26: feature_name=SARS.CoV.S1.HisTag	feature_id[6].value > threshold=15087.41650390625
node_38: feature_name=hCoV.HKU1.NP	feature_id[10].value > threshold=348.6666564941406
node_46: feature_name=SARS.CoV.2.S2	feature_id[2].value <= threshold=21127.650390625
node_47: feature_name=SARS.CoV.2.Spike.RBD.rFc	feature_id[9].value > threshold=38684.56640625
Class: healthcare workers over 180 days after vaccination	
Rules_130	passed counts:2
node_0: feature_name=HuIgG_0.10	feature_id[1].value <= threshold=19120.166015625
node_1: feature_name=SARS.CoV.2.S1.mFcTag	feature_id[3].value > threshold=5354.388916015625
node_9: feature_name=a-HuIgG_0.30	feature_id[4].value <= threshold=33726.833984375

node_10: feature_name=HuIgG_0.03	feature_id[5].value > threshold=1023.1666564941406
node_20: feature_name=hCoV.HKU1.NP	feature_id[10].value > threshold=-20.05833387374878
node_22: feature_name=SARS.CoV.S1.HisTag	feature_id[6].value > threshold=400.3000030517578
node_26: feature_name=SARS.CoV.S1.HisTag	feature_id[6].value > threshold=15087.41650390625
node_38: feature_name=hCoV.HKU1.NP	feature_id[10].value <= threshold=348.6666564941406
node_39: feature_name=hCoV.NL63.S1_S2	feature_id[7].value <= threshold=26557.333984375
node_40: feature_name=SARS.CoV.S1.HisTag	feature_id[6].value <= threshold=25028.0
Class: healthcare workers between 60 and 180 days after vaccination	
Rules_131	passed counts:2
node_0: feature_name=HuIgG_0.10	feature_id[1].value <= threshold=19120.166015625
node_1: feature_name=SARS.CoV.2.S1.mFcTag	feature_id[3].value > threshold=5354.388916015625
node_9: feature_name=a-HuIgG_0.30	feature_id[4].value <= threshold=33726.833984375
node_10: feature_name=HuIgG_0.03	feature_id[5].value > threshold=1023.1666564941406
node_20: feature_name=hCoV.HKU1.NP	feature_id[10].value > threshold=-20.05833387374878
node_22: feature_name=SARS.CoV.S1.HisTag	feature_id[6].value <= threshold=400.3000030517578
node_23: feature_name=hCoV.NL63.S1_S2	feature_id[7].value > threshold=25709.0419921875
Class: unvaccinated healthcare workers	
Rules_132	passed counts:2
node_0: feature_name=HuIgG_0.10	feature_id[1].value <= threshold=19120.166015625
node_1: feature_name=SARS.CoV.2.S1.mFcTag	feature_id[3].value > threshold=5354.388916015625
node_9: feature_name=a-HuIgG_0.30	feature_id[4].value <= threshold=33726.833984375
node_10: feature_name=HuIgG_0.03	feature_id[5].value > threshold=1023.1666564941406
node_20: feature_name=hCoV.HKU1.NP	feature_id[10].value <= threshold=-20.05833387374878

Class: healthcare workers over 180 days after vaccination	
Rules_133	passed counts:1
node_0: feature_name=HuIgG_0.10	feature_id[1].value > threshold=19120.166015625
node_112: feature_name=a-HuIgG_0.03	feature_id[8].value > threshold=19665.666015625
node_428: feature_name=SARS.CoV.2.Spike.RBD.rFc	feature_id[9].value <= threshold=55147.1484375
node_429: feature_name=a-HuIgG_0.30	feature_id[4].value > threshold=51289.166015625
node_437: feature_name=HuIgG_0.10	feature_id[1].value <= threshold=25959.0
node_438: feature_name=HuIgG_0.03	feature_id[5].value > threshold=7564.833251953125
Class: healthcare workers between 60 and 180 days after vaccination	
Rules_134	passed counts:1
node_0: feature_name=HuIgG_0.10	feature_id[1].value > threshold=19120.166015625
node_112: feature_name=a-HuIgG_0.03	feature_id[8].value > threshold=19665.666015625
node_428: feature_name=SARS.CoV.2.Spike.RBD.rFc	feature_id[9].value <= threshold=55147.1484375
node_429: feature_name=a-HuIgG_0.30	feature_id[4].value <= threshold=51289.166015625
node_430: feature_name=Flu.B_Phu/.HA1+HA2	feature_id[11].value > threshold=48173.93359375
node_434: feature_name=a-HuIgG_0.30	feature_id[4].value <= threshold=47010.66796875
Class: healthcare workers within 60 days after vaccination	
Rules_135	passed counts:1
node_0: feature_name=HuIgG_0.10	feature_id[1].value > threshold=19120.166015625
node_112: feature_name=a-HuIgG_0.03	feature_id[8].value > threshold=19665.666015625
node_428: feature_name=SARS.CoV.2.Spike.RBD.rFc	feature_id[9].value <= threshold=55147.1484375
node_429: feature_name=a-HuIgG_0.30	feature_id[4].value <= threshold=51289.166015625

node_430: feature_name=Flu.B_Phu/.HA1+HA2	feature_id[11].value <= threshold=48173.93359375
node_431: feature_name=SARS.CoV.2.S1+S2	feature_id[12].value <= threshold=39605.06640625
Class: healthcare workers between 60 and 180 days after vaccination	
Rules_136	passed counts:1
node_0: feature_name=HuIgG_0.10	feature_id[1].value > threshold=19120.166015625
node_112: feature_name=a-HuIgG_0.03	feature_id[8].value <= threshold=19665.666015625
node_113: feature_name=SARS.CoV.2.S1.mFcTag	feature_id[3].value > threshold=54010.166015625
node_351: feature_name=SARS.CoV.2.S2	feature_id[2].value > threshold=37075.75
node_391: feature_name=SARS.CoV.2.S1.mFcTag	feature_id[3].value > threshold=55462.833984375
node_425: feature_name=hCoV.NL63.S1_S2	feature_id[7].value <= threshold=29337.0
Class: healthcare workers within 60 days after vaccination	
Rules_137	passed counts:1
node_0: feature_name=HuIgG_0.10	feature_id[1].value > threshold=19120.166015625
node_112: feature_name=a-HuIgG_0.03	feature_id[8].value <= threshold=19665.666015625
node_113: feature_name=SARS.CoV.2.S1.mFcTag	feature_id[3].value > threshold=54010.166015625
node_351: feature_name=SARS.CoV.2.S2	feature_id[2].value > threshold=37075.75
node_391: feature_name=SARS.CoV.2.S1.mFcTag	feature_id[3].value <= threshold=55462.833984375
node_392: feature_name=SARS.CoV.2.S1.mFcTag	feature_id[3].value > threshold=55163.634765625
node_420: feature_name=SARS.CoV.2.S1+S2	feature_id[12].value > threshold=54621.0
node_422: feature_name=SARS.CoV.2.Spike.RBD.rFc	feature_id[9].value > threshold=51908.0
Class: healthcare workers within 60 days after vaccination	
Rules_138	passed counts:1

node_0: feature_name=HuIgG_0.10	feature_id[1].value > threshold=19120.166015625
node_112: feature_name=a-HuIgG_0.03	feature_id[8].value <= threshold=19665.666015625
node_113: feature_name=SARS.CoV.2.S1.mFcTag	feature_id[3].value > threshold=54010.166015625
node_351: feature_name=SARS.CoV.2.S2	feature_id[2].value > threshold=37075.75
node_391: feature_name=SARS.CoV.2.S1.mFcTag	feature_id[3].value <= threshold=55462.833984375
node_392: feature_name=SARS.CoV.2.S1.mFcTag	feature_id[3].value > threshold=55163.634765625
node_420: feature_name=SARS.CoV.2.S1+S2	feature_id[12].value > threshold=54621.0
node_422: feature_name=SARS.CoV.2.Spike.RBD.rFc	feature_id[9].value <= threshold=51908.0
Class: healthcare workers between 60 and 180 days after vaccination	
Rules_139	passed counts:1
node_0: feature_name=HuIgG_0.10	feature_id[1].value > threshold=19120.166015625
node_112: feature_name=a-HuIgG_0.03	feature_id[8].value <= threshold=19665.666015625
node_113: feature_name=SARS.CoV.2.S1.mFcTag	feature_id[3].value > threshold=54010.166015625
node_351: feature_name=SARS.CoV.2.S2	feature_id[2].value > threshold=37075.75
node_391: feature_name=SARS.CoV.2.S1.mFcTag	feature_id[3].value <= threshold=55462.833984375
node_392: feature_name=SARS.CoV.2.S1.mFcTag	feature_id[3].value <= threshold=55163.634765625
node_393: feature_name=hCoV.HKU1.NP	feature_id[10].value <= threshold=14561.3330078125
node_394: feature_name=hCoV.HKU1.NP	feature_id[10].value > threshold=6453.333251953125
node_412: feature_name=HuIgG_0.03	feature_id[5].value > threshold=4766.166748046875
node_414: feature_name=MoIgA_0.10	feature_id[13].value > threshold=2918.0
node_416: feature_name=HuIgG_0.03	feature_id[5].value > threshold=6174.0
Class: healthcare workers between 60 and 180 days after vaccination	
Rules_140	passed counts:1

node_0: feature_name=HuIgG_0.10	feature_id[1].value > threshold=19120.166015625
node_112: feature_name=a-HuIgG_0.03	feature_id[8].value <= threshold=19665.666015625
node_113: feature_name=SARS.CoV.2.S1.mFcTag	feature_id[3].value > threshold=54010.166015625
node_351: feature_name=SARS.CoV.2.S2	feature_id[2].value > threshold=37075.75
node_391: feature_name=SARS.CoV.2.S1.mFcTag	feature_id[3].value <= threshold=55462.833984375
node_392: feature_name=SARS.CoV.2.S1.mFcTag	feature_id[3].value <= threshold=55163.634765625
node_393: feature_name=hCoV.HKU1.NP	feature_id[10].value <= threshold=14561.3330078125
node_394: feature_name=hCoV.HKU1.NP	feature_id[10].value <= threshold=6453.333251953125
node_395: feature_name=hCoV.HKU1.NP	feature_id[10].value > threshold=1990.1666259765625
node_407: feature_name=SARS.CoV.2.S1.RBD.mFc	feature_id[0].value > threshold=48385.75
node_409: feature_name=MoIgA_0.10	feature_id[13].value > threshold=21414.99951171875
Class: healthcare workers between 60 and 180 days after vaccination	
Rules_141	passed counts:1
node_0: feature_name=HuIgG_0.10	feature_id[1].value > threshold=19120.166015625
node_112: feature_name=a-HuIgG_0.03	feature_id[8].value <= threshold=19665.666015625
node_113: feature_name=SARS.CoV.2.S1.mFcTag	feature_id[3].value > threshold=54010.166015625
node_351: feature_name=SARS.CoV.2.S2	feature_id[2].value > threshold=37075.75
node_391: feature_name=SARS.CoV.2.S1.mFcTag	feature_id[3].value <= threshold=55462.833984375
node_392: feature_name=SARS.CoV.2.S1.mFcTag	feature_id[3].value <= threshold=55163.634765625
node_393: feature_name=hCoV.HKU1.NP	feature_id[10].value <= threshold=14561.3330078125
node_394: feature_name=hCoV.HKU1.NP	feature_id[10].value <= threshold=6453.333251953125
node_395: feature_name=hCoV.HKU1.NP	feature_id[10].value <= threshold=1990.1666259765625
node_396: feature_name=SARS.CoV.S1.HisTag	feature_id[6].value > threshold=12607.2998046875
node_398: feature_name=a-HuIgG_0.03	feature_id[8].value > threshold=15419.83349609375

node_404: feature_name=SARS.CoV.2.S1.RBD.mFc	feature_id[0].value > threshold=52143.06640625
Class: healthcare workers over 180 days after vaccination	
Rules_142	passed counts:1
node_0: feature_name=HuIgG_0.10	feature_id[1].value > threshold=19120.166015625
node_112: feature_name=a-HuIgG_0.03	feature_id[8].value <= threshold=19665.666015625
node_113: feature_name=SARS.CoV.2.S1.mFcTag	feature_id[3].value > threshold=54010.166015625
node_351: feature_name=SARS.CoV.2.S2	feature_id[2].value > threshold=37075.75
node_391: feature_name=SARS.CoV.2.S1.mFcTag	feature_id[3].value <= threshold=55462.833984375
node_392: feature_name=SARS.CoV.2.S1.mFcTag	feature_id[3].value <= threshold=55163.634765625
node_393: feature_name=hCoV.HKU1.NP	feature_id[10].value <= threshold=14561.3330078125
node_394: feature_name=hCoV.HKU1.NP	feature_id[10].value <= threshold=6453.333251953125
node_395: feature_name=hCoV.HKU1.NP	feature_id[10].value <= threshold=1990.1666259765625
node_396: feature_name=SARS.CoV.S1.HisTag	feature_id[6].value > threshold=12607.2998046875
node_398: feature_name=a-HuIgG_0.03	feature_id[8].value > threshold=15419.83349609375
node_404: feature_name=SARS.CoV.2.S1.RBD.mFc	feature_id[0].value <= threshold=52143.06640625
Class: unvaccinated healthcare workers	
Rules_143	passed counts:1
node_0: feature_name=HuIgG_0.10	feature_id[1].value > threshold=19120.166015625
node_112: feature_name=a-HuIgG_0.03	feature_id[8].value <= threshold=19665.666015625
node_113: feature_name=SARS.CoV.2.S1.mFcTag	feature_id[3].value > threshold=54010.166015625
node_351: feature_name=SARS.CoV.2.S2	feature_id[2].value > threshold=37075.75
node_391: feature_name=SARS.CoV.2.S1.mFcTag	feature_id[3].value <= threshold=55462.833984375
node_392: feature_name=SARS.CoV.2.S1.mFcTag	feature_id[3].value <= threshold=55163.634765625

node_393: feature_name=hCoV.HKU1.NP	feature_id[10].value <= threshold=14561.3330078125
node_394: feature_name=hCoV.HKU1.NP	feature_id[10].value <= threshold=6453.333251953125
node_395: feature_name=hCoV.HKU1.NP	feature_id[10].value <= threshold=1990.1666259765625
node_396: feature_name=SARS.CoV.S1.HisTag	feature_id[6].value > threshold=12607.2998046875
node_398: feature_name=a-HuIgG_0.03	feature_id[8].value <= threshold=15419.83349609375
node_399: feature_name=SARS.CoV.2.S1.mFcTag	feature_id[3].value > threshold=55018.716796875
node_401: feature_name=hCoV.NL63.S1_S2	feature_id[7].value > threshold=31181.3837890625
Class: healthcare workers within 60 days after vaccination	
Rules_144	passed counts:1
node_0: feature_name=HuIgG_0.10	feature_id[1].value > threshold=19120.166015625
node_112: feature_name=a-HuIgG_0.03	feature_id[8].value <= threshold=19665.666015625
node_113: feature_name=SARS.CoV.2.S1.mFcTag	feature_id[3].value > threshold=54010.166015625
node_351: feature_name=SARS.CoV.2.S2	feature_id[2].value > threshold=37075.75
node_391: feature_name=SARS.CoV.2.S1.mFcTag	feature_id[3].value <= threshold=55462.833984375
node_392: feature_name=SARS.CoV.2.S1.mFcTag	feature_id[3].value <= threshold=55163.634765625
node_393: feature_name=hCoV.HKU1.NP	feature_id[10].value <= threshold=14561.3330078125
node_394: feature_name=hCoV.HKU1.NP	feature_id[10].value <= threshold=6453.333251953125
node_395: feature_name=hCoV.HKU1.NP	feature_id[10].value <= threshold=1990.1666259765625
node_396: feature_name=SARS.CoV.S1.HisTag	feature_id[6].value > threshold=12607.2998046875
node_398: feature_name=a-HuIgG_0.03	feature_id[8].value <= threshold=15419.83349609375
node_399: feature_name=SARS.CoV.2.S1.mFcTag	feature_id[3].value > threshold=55018.716796875
node_401: feature_name=hCoV.NL63.S1_S2	feature_id[7].value <= threshold=31181.3837890625
Class: healthcare workers between 60 and 180 days after vaccination	

Rules_145	passed counts:1
node_0: feature_name=HuIgG_0.10	feature_id[1].value > threshold=19120.166015625
node_112: feature_name=a-HuIgG_0.03	feature_id[8].value <= threshold=19665.666015625
node_113: feature_name=SARS.CoV.2.S1.mFcTag	feature_id[3].value > threshold=54010.166015625
node_351: feature_name=SARS.CoV.2.S2	feature_id[2].value <= threshold=37075.75
node_352: feature_name=a-HuIgG_0.03	feature_id[8].value > threshold=17721.1669921875
node_386: feature_name=hCoV.HKU1.NP	feature_id[10].value <= threshold=4175.4998779296875
node_387: feature_name=hCoV.HKU1.NP	feature_id[10].value > threshold=3016.9666748046875
Class: unvaccinated healthcare workers	
Rules_146	passed counts:1
node_0: feature_name=HuIgG_0.10	feature_id[1].value > threshold=19120.166015625
node_112: feature_name=a-HuIgG_0.03	feature_id[8].value <= threshold=19665.666015625
node_113: feature_name=SARS.CoV.2.S1.mFcTag	feature_id[3].value > threshold=54010.166015625
node_351: feature_name=SARS.CoV.2.S2	feature_id[2].value <= threshold=37075.75
node_352: feature_name=a-HuIgG_0.03	feature_id[8].value <= threshold=17721.1669921875
node_353: feature_name=MoIgA_0.10	feature_id[13].value > threshold=536.0
node_367: feature_name=HuIgG_0.03	feature_id[5].value > threshold=5634.333251953125
node_381: feature_name=MoIgA_0.10	feature_id[13].value > threshold=15050.5
node_383: feature_name=SARS.CoV.2.Spike.RBD.rFc	feature_id[9].value > threshold=50235.5
Class: healthcare workers within 60 days after vaccination	
Rules_147	passed counts:1
node_0: feature_name=HuIgG_0.10	feature_id[1].value > threshold=19120.166015625
node_112: feature_name=a-HuIgG_0.03	feature_id[8].value <= threshold=19665.666015625

node_113: feature_name=SARS.CoV.2.S1.mFcTag	feature_id[3].value > threshold=54010.166015625
node_351: feature_name=SARS.CoV.2.S2	feature_id[2].value <= threshold=37075.75
node_352: feature_name=a-HuIgG_0.03	feature_id[8].value <= threshold=17721.1669921875
node_353: feature_name=MoIgA_0.10	feature_id[13].value > threshold=536.0
node_367: feature_name=HuIgG_0.03	feature_id[5].value > threshold=5634.333251953125
node_381: feature_name=MoIgA_0.10	feature_id[13].value > threshold=15050.5
node_383: feature_name=SARS.CoV.2.Spike.RBD.rFc	feature_id[9].value <= threshold=50235.5
Class: healthcare workers between 60 and 180 days after vaccination	
Rules_148	passed counts:1
node_0: feature_name=HuIgG_0.10	feature_id[1].value > threshold=19120.166015625
node_112: feature_name=a-HuIgG_0.03	feature_id[8].value <= threshold=19665.666015625
node_113: feature_name=SARS.CoV.2.S1.mFcTag	feature_id[3].value > threshold=54010.166015625
node_351: feature_name=SARS.CoV.2.S2	feature_id[2].value <= threshold=37075.75
node_352: feature_name=a-HuIgG_0.03	feature_id[8].value <= threshold=17721.1669921875
node_353: feature_name=MoIgA_0.10	feature_id[13].value > threshold=536.0
node_367: feature_name=HuIgG_0.03	feature_id[5].value <= threshold=5634.333251953125
node_368: feature_name=SARS.CoV.2.S1+S2	feature_id[12].value > threshold=52432.75
node_378: feature_name=a-HuIgG_0.30	feature_id[4].value > threshold=49923.66796875
Class: healthcare workers between 60 and 180 days after vaccination	
Rules_149	passed counts:1
node_0: feature_name=HuIgG_0.10	feature_id[1].value > threshold=19120.166015625
node_112: feature_name=a-HuIgG_0.03	feature_id[8].value <= threshold=19665.666015625
node_113: feature_name=SARS.CoV.2.S1.mFcTag	feature_id[3].value > threshold=54010.166015625

node_351: feature_name=SARS.CoV.2.S2	feature_id[2].value <= threshold=37075.75
node_352: feature_name=a-HuIgG_0.03	feature_id[8].value <= threshold=17721.1669921875
node_353: feature_name=MoIgA_0.10	feature_id[13].value > threshold=536.0
node_367: feature_name=HuIgG_0.03	feature_id[5].value <= threshold=5634.333251953125
node_368: feature_name=SARS.CoV.2.S1+S2	feature_id[12].value <= threshold=52432.75
node_369: feature_name=SARS.CoV.S1.HisTag	feature_id[6].value <= threshold=17773.60009765625
node_370: feature_name=SARS.CoV.2.S2	feature_id[2].value > threshold=35666.966796875
Class: healthcare workers within 60 days after vaccination	
Rules_150	passed counts:1
node_0: feature_name=HuIgG_0.10	feature_id[1].value > threshold=19120.166015625
node_112: feature_name=a-HuIgG_0.03	feature_id[8].value <= threshold=19665.666015625
node_113: feature_name=SARS.CoV.2.S1.mFcTag	feature_id[3].value > threshold=54010.166015625
node_351: feature_name=SARS.CoV.2.S2	feature_id[2].value <= threshold=37075.75
node_352: feature_name=a-HuIgG_0.03	feature_id[8].value <= threshold=17721.1669921875
node_353: feature_name=MoIgA_0.10	feature_id[13].value > threshold=536.0
node_367: feature_name=HuIgG_0.03	feature_id[5].value <= threshold=5634.333251953125
node_368: feature_name=SARS.CoV.2.S1+S2	feature_id[12].value <= threshold=52432.75
node_369: feature_name=SARS.CoV.S1.HisTag	feature_id[6].value <= threshold=17773.60009765625
node_370: feature_name=SARS.CoV.2.S2	feature_id[2].value <= threshold=35666.966796875
node_371: feature_name=hCoV.HKU1.NP	feature_id[10].value > threshold=14106.56640625
node_373: feature_name=hCoV.NL63.S1_S2	feature_id[7].value > threshold=44287.798828125
Class: healthcare workers between 60 and 180 days after vaccination	
Rules_151	passed counts:1

node_0: feature_name=HuIgG_0.10	feature_id[1].value > threshold=19120.166015625
node_112: feature_name=a-HuIgG_0.03	feature_id[8].value <= threshold=19665.666015625
node_113: feature_name=SARS.CoV.2.S1.mFcTag	feature_id[3].value > threshold=54010.166015625
node_351: feature_name=SARS.CoV.2.S2	feature_id[2].value <= threshold=37075.75
node_352: feature_name=a-HuIgG_0.03	feature_id[8].value <= threshold=17721.1669921875
node_353: feature_name=MoIgA_0.10	feature_id[13].value > threshold=536.0
node_367: feature_name=HuIgG_0.03	feature_id[5].value <= threshold=5634.333251953125
node_368: feature_name=SARS.CoV.2.S1+S2	feature_id[12].value <= threshold=52432.75
node_369: feature_name=SARS.CoV.S1.HisTag	feature_id[6].value <= threshold=17773.60009765625
node_370: feature_name=SARS.CoV.2.S2	feature_id[2].value <= threshold=35666.966796875
node_371: feature_name=hCoV.HKU1.NP	feature_id[10].value > threshold=14106.56640625
node_373: feature_name=hCoV.NL63.S1_S2	feature_id[7].value <= threshold=44287.798828125
Class: healthcare workers within 60 days after vaccination	
Rules_152	passed counts:1
node_0: feature_name=HuIgG_0.10	feature_id[1].value > threshold=19120.166015625
node_112: feature_name=a-HuIgG_0.03	feature_id[8].value <= threshold=19665.666015625
node_113: feature_name=SARS.CoV.2.S1.mFcTag	feature_id[3].value > threshold=54010.166015625
node_351: feature_name=SARS.CoV.2.S2	feature_id[2].value <= threshold=37075.75
node_352: feature_name=a-HuIgG_0.03	feature_id[8].value <= threshold=17721.1669921875
node_353: feature_name=MoIgA_0.10	feature_id[13].value <= threshold=536.0
node_354: feature_name=hCoV.HKU1.NP	feature_id[10].value <= threshold=7098.5
node_355: feature_name=MoIgA_0.10	feature_id[13].value <= threshold=462.0
node_356: feature_name=hCoV.HKU1.NP	feature_id[10].value <= threshold=2790.583251953125
node_357: feature_name=hCoV.NL63.S1_S2	feature_id[7].value > threshold=36099.0

node_359: feature_name=hCoV.HKU1.NP	feature_id[10].value <= threshold=1348.6666870117188
node_360: feature_name=SARS.CoV.S1.HisTag	feature_id[6].value <= threshold=5850.833251953125
Class: healthcare workers within 60 days after vaccination	
Rules_153	passed counts:1
node_0: feature_name=HuIgG_0.10	feature_id[1].value > threshold=19120.166015625
node_112: feature_name=a-HuIgG_0.03	feature_id[8].value <= threshold=19665.666015625
node_113: feature_name=SARS.CoV.2.S1.mFcTag	feature_id[3].value <= threshold=54010.166015625
node_114: feature_name=SARS.CoV.2.S1+S2	feature_id[12].value > threshold=47258.43359375
node_240: feature_name=HuIgG_0.03	feature_id[5].value > threshold=5455.5
node_266: feature_name=HuIgG_0.10	feature_id[1].value > threshold=24231.0
node_284: feature_name=HuIgG_0.03	feature_id[5].value > threshold=5469.0
node_286: feature_name=HuIgG_0.10	feature_id[1].value <= threshold=30107.666015625
node_287: feature_name=SARS.CoV.2.S1+S2	feature_id[12].value > threshold=47536.224609375
node_291: feature_name=SARS.CoV.2.S2	feature_id[2].value > threshold=37653.75
node_329: feature_name=SARS.CoV.2.S1.mFcTag	feature_id[3].value > threshold=53346.333984375
node_345: feature_name=SARS.CoV.2.S1.mFcTag	feature_id[3].value <= threshold=53555.333984375
node_346: feature_name=SARS.CoV.2.S2	feature_id[2].value <= threshold=45480.583984375
Class: healthcare workers within 60 days after vaccination	
Rules_154	passed counts:1
node_0: feature_name=HuIgG_0.10	feature_id[1].value > threshold=19120.166015625
node_112: feature_name=a-HuIgG_0.03	feature_id[8].value <= threshold=19665.666015625
node_113: feature_name=SARS.CoV.2.S1.mFcTag	feature_id[3].value <= threshold=54010.166015625
node_114: feature_name=SARS.CoV.2.S1+S2	feature_id[12].value > threshold=47258.43359375

node_240: feature_name=HuIgG_0.03	feature_id[5].value > threshold=5455.5
node_266: feature_name=HuIgG_0.10	feature_id[1].value > threshold=24231.0
node_284: feature_name=HuIgG_0.03	feature_id[5].value > threshold=5469.0
node_286: feature_name=HuIgG_0.10	feature_id[1].value <= threshold=30107.666015625
node_287: feature_name=SARS.CoV.2.S1+S2	feature_id[12].value > threshold=47536.224609375
node_291: feature_name=SARS.CoV.2.S2	feature_id[2].value > threshold=37653.75
node_329: feature_name=SARS.CoV.2.S1.mFcTag	feature_id[3].value <= threshold=53346.333984375
node_330: feature_name=Flu.B_Phu/.HA1+HA2	feature_id[11].value > threshold=53733.0
node_340: feature_name=SARS.CoV.2.S1+S2	feature_id[12].value <= threshold=51770.29296875
node_341: feature_name=Flu.B_Phu/.HA1+HA2	feature_id[11].value > threshold=53805.5
Class: healthcare workers between 60 and 180 days after vaccination	
Rules_155	passed counts:1
node_0: feature_name=HuIgG_0.10	feature_id[1].value > threshold=19120.166015625
node_112: feature_name=a-HuIgG_0.03	feature_id[8].value <= threshold=19665.666015625
node_113: feature_name=SARS.CoV.2.S1.mFcTag	feature_id[3].value <= threshold=54010.166015625
node_114: feature_name=SARS.CoV.2.S1+S2	feature_id[12].value > threshold=47258.43359375
node_240: feature_name=HuIgG_0.03	feature_id[5].value > threshold=5455.5
node_266: feature_name=HuIgG_0.10	feature_id[1].value > threshold=24231.0
node_284: feature_name=HuIgG_0.03	feature_id[5].value > threshold=5469.0
node_286: feature_name=HuIgG_0.10	feature_id[1].value <= threshold=30107.666015625
node_287: feature_name=SARS.CoV.2.S1+S2	feature_id[12].value > threshold=47536.224609375
node_291: feature_name=SARS.CoV.2.S2	feature_id[2].value > threshold=37653.75
node_329: feature_name=SARS.CoV.2.S1.mFcTag	feature_id[3].value <= threshold=53346.333984375
node_330: feature_name=Flu.B_Phu/.HA1+HA2	feature_id[11].value > threshold=53733.0

node_340: feature_name=SARS.CoV.2.S1+S2	feature_id[12].value <= threshold=51770.29296875
node_341: feature_name=Flu.B_Phu/.HA1+HA2	feature_id[11].value <= threshold=53805.5
Class: healthcare workers over 180 days after vaccination	
Rules_156	passed counts:1
node_0: feature_name=HuIgG_0.10	feature_id[1].value > threshold=19120.166015625
node_112: feature_name=a-HuIgG_0.03	feature_id[8].value <= threshold=19665.666015625
node_113: feature_name=SARS.CoV.2.S1.mFcTag	feature_id[3].value <= threshold=54010.166015625
node_114: feature_name=SARS.CoV.2.S1+S2	feature_id[12].value > threshold=47258.43359375
node_240: feature_name=HuIgG_0.03	feature_id[5].value > threshold=5455.5
node_266: feature_name=HuIgG_0.10	feature_id[1].value > threshold=24231.0
node_284: feature_name=HuIgG_0.03	feature_id[5].value > threshold=5469.0
node_286: feature_name=HuIgG_0.10	feature_id[1].value <= threshold=30107.666015625
node_287: feature_name=SARS.CoV.2.S1+S2	feature_id[12].value > threshold=47536.224609375
node_291: feature_name=SARS.CoV.2.S2	feature_id[2].value > threshold=37653.75
node_329: feature_name=SARS.CoV.2.S1.mFcTag	feature_id[3].value <= threshold=53346.333984375
node_330: feature_name=Flu.B_Phu/.HA1+HA2	feature_id[11].value <= threshold=53733.0
node_331: feature_name=MoIgA_0.10	feature_id[13].value > threshold=10.833333015441895
node_335: feature_name=a-HuIgG_0.03	feature_id[8].value <= threshold=11890.0
node_336: feature_name=HuIgG_0.10	feature_id[1].value > threshold=25504.333984375
Class: healthcare workers between 60 and 180 days after vaccination	
Rules_157	passed counts:1
node_0: feature_name=HuIgG_0.10	feature_id[1].value > threshold=19120.166015625
node_112: feature_name=a-HuIgG_0.03	feature_id[8].value <= threshold=19665.666015625

node_113: feature_name=SARS.CoV.2.S1.mFcTag	feature_id[3].value <= threshold=54010.166015625
node_114: feature_name=SARS.CoV.2.S1+S2	feature_id[12].value > threshold=47258.43359375
node_240: feature_name=HuIgG_0.03	feature_id[5].value > threshold=5455.5
node_266: feature_name=HuIgG_0.10	feature_id[1].value > threshold=24231.0
node_284: feature_name=HuIgG_0.03	feature_id[5].value > threshold=5469.0
node_286: feature_name=HuIgG_0.10	feature_id[1].value <= threshold=30107.666015625
node_287: feature_name=SARS.CoV.2.S1+S2	feature_id[12].value > threshold=47536.224609375
node_291: feature_name=SARS.CoV.2.S2	feature_id[2].value > threshold=37653.75
node_329: feature_name=SARS.CoV.2.S1.mFcTag	feature_id[3].value <= threshold=53346.333984375
node_330: feature_name=Flu.B_Phu/.HA1+HA2	feature_id[11].value <= threshold=53733.0
node_331: feature_name=MoIgA_0.10	feature_id[13].value <= threshold=10.833333015441895
node_332: feature_name=SARS.CoV.2.S2	feature_id[2].value > threshold=50911.75
Class: healthcare workers within 60 days after vaccination	
Rules_158	passed counts:1
node_0: feature_name=HuIgG_0.10	feature_id[1].value > threshold=19120.166015625
node_112: feature_name=a-HuIgG_0.03	feature_id[8].value <= threshold=19665.666015625
node_113: feature_name=SARS.CoV.2.S1.mFcTag	feature_id[3].value <= threshold=54010.166015625
node_114: feature_name=SARS.CoV.2.S1+S2	feature_id[12].value > threshold=47258.43359375
node_240: feature_name=HuIgG_0.03	feature_id[5].value > threshold=5455.5
node_266: feature_name=HuIgG_0.10	feature_id[1].value > threshold=24231.0
node_284: feature_name=HuIgG_0.03	feature_id[5].value > threshold=5469.0
node_286: feature_name=HuIgG_0.10	feature_id[1].value <= threshold=30107.666015625
node_287: feature_name=SARS.CoV.2.S1+S2	feature_id[12].value > threshold=47536.224609375
node_291: feature_name=SARS.CoV.2.S2	feature_id[2].value > threshold=37653.75

node_329: feature_name=SARS.CoV.2.S1.mFcTag	feature_id[3].value <= threshold=53346.333984375
node_330: feature_name=Flu.B_Phu/.HA1+HA2	feature_id[11].value <= threshold=53733.0
node_331: feature_name=MoIgA_0.10	feature_id[13].value <= threshold=10.833333015441895
node_332: feature_name=SARS.CoV.2.S2	feature_id[2].value <= threshold=50911.75
Class: healthcare workers between 60 and 180 days after vaccination	
Rules_159	passed counts:1
node_0: feature_name=HuIgG_0.10	feature_id[1].value > threshold=19120.166015625
node_112: feature_name=a-HuIgG_0.03	feature_id[8].value <= threshold=19665.666015625
node_113: feature_name=SARS.CoV.2.S1.mFcTag	feature_id[3].value <= threshold=54010.166015625
node_114: feature_name=SARS.CoV.2.S1+S2	feature_id[12].value > threshold=47258.43359375
node_240: feature_name=HuIgG_0.03	feature_id[5].value > threshold=5455.5
node_266: feature_name=HuIgG_0.10	feature_id[1].value > threshold=24231.0
node_284: feature_name=HuIgG_0.03	feature_id[5].value > threshold=5469.0
node_286: feature_name=HuIgG_0.10	feature_id[1].value <= threshold=30107.666015625
node_287: feature_name=SARS.CoV.2.S1+S2	feature_id[12].value > threshold=47536.224609375
node_291: feature_name=SARS.CoV.2.S2	feature_id[2].value <= threshold=37653.75
node_292: feature_name=SARS.CoV.2.S2	feature_id[2].value > threshold=36044.29296875
node_322: feature_name=hCoV.HKU1.NP	feature_id[10].value > threshold=2383.1666870117188
node_324: feature_name=SARS.CoV.2.S1+S2	feature_id[12].value <= threshold=49449.75
node_325: feature_name=hCoV.HKU1.NP	feature_id[10].value > threshold=9806.416748046875
Class: healthcare workers between 60 and 180 days after vaccination	
Rules_160	passed counts:1
node_0: feature_name=HuIgG_0.10	feature_id[1].value > threshold=19120.166015625

node_112: feature_name=a-HuIgG_0.03	feature_id[8].value <= threshold=19665.666015625
node_113: feature_name=SARS.CoV.2.S1.mFcTag	feature_id[3].value <= threshold=54010.166015625
node_114: feature_name=SARS.CoV.2.S1+S2	feature_id[12].value > threshold=47258.43359375
node_240: feature_name=HuIgG_0.03	feature_id[5].value > threshold=5455.5
node_266: feature_name=HuIgG_0.10	feature_id[1].value > threshold=24231.0
node_284: feature_name=HuIgG_0.03	feature_id[5].value > threshold=5469.0
node_286: feature_name=HuIgG_0.10	feature_id[1].value <= threshold=30107.666015625
node_287: feature_name=SARS.CoV.2.S1+S2	feature_id[12].value > threshold=47536.224609375
node_291: feature_name=SARS.CoV.2.S2	feature_id[2].value <= threshold=37653.75
node_292: feature_name=SARS.CoV.2.S2	feature_id[2].value > threshold=36044.29296875
node_322: feature_name=hCoV.HKU1.NP	feature_id[10].value > threshold=2383.1666870117188
node_324: feature_name=SARS.CoV.2.S1+S2	feature_id[12].value <= threshold=49449.75
node_325: feature_name=hCoV.HKU1.NP	feature_id[10].value <= threshold=9806.416748046875
Class: healthcare workers within 60 days after vaccination	
Rules_161	passed counts:1
node_0: feature_name=HuIgG_0.10	feature_id[1].value > threshold=19120.166015625
node_112: feature_name=a-HuIgG_0.03	feature_id[8].value <= threshold=19665.666015625
node_113: feature_name=SARS.CoV.2.S1.mFcTag	feature_id[3].value <= threshold=54010.166015625
node_114: feature_name=SARS.CoV.2.S1+S2	feature_id[12].value > threshold=47258.43359375
node_240: feature_name=HuIgG_0.03	feature_id[5].value > threshold=5455.5
node_266: feature_name=HuIgG_0.10	feature_id[1].value > threshold=24231.0
node_284: feature_name=HuIgG_0.03	feature_id[5].value > threshold=5469.0
node_286: feature_name=HuIgG_0.10	feature_id[1].value <= threshold=30107.666015625
node_287: feature_name=SARS.CoV.2.S1+S2	feature_id[12].value > threshold=47536.224609375

node_291: feature_name=SARS.CoV.2.S2	feature_id[2].value <= threshold=37653.75
node_292: feature_name=SARS.CoV.2.S2	feature_id[2].value <= threshold=36044.29296875
node_293: feature_name=HuIgG_0.03	feature_id[5].value > threshold=6329.5
node_301: feature_name=HuIgG_0.03	feature_id[5].value > threshold=6562.16650390625
node_307: feature_name=Flu.B_Phu/.HA1+HA2	feature_id[11].value > threshold=54123.5
node_319: feature_name=hCoV.NL63.S1_S2	feature_id[7].value > threshold=43715.833984375
Class: healthcare workers within 60 days after vaccination	
Rules_162	passed counts:1
node_0: feature_name=HuIgG_0.10	feature_id[1].value > threshold=19120.166015625
node_112: feature_name=a-HuIgG_0.03	feature_id[8].value <= threshold=19665.666015625
node_113: feature_name=SARS.CoV.2.S1.mFcTag	feature_id[3].value <= threshold=54010.166015625
node_114: feature_name=SARS.CoV.2.S1+S2	feature_id[12].value > threshold=47258.43359375
node_240: feature_name=HuIgG_0.03	feature_id[5].value > threshold=5455.5
node_266: feature_name=HuIgG_0.10	feature_id[1].value > threshold=24231.0
node_284: feature_name=HuIgG_0.03	feature_id[5].value > threshold=5469.0
node_286: feature_name=HuIgG_0.10	feature_id[1].value <= threshold=30107.666015625
node_287: feature_name=SARS.CoV.2.S1+S2	feature_id[12].value > threshold=47536.224609375
node_291: feature_name=SARS.CoV.2.S2	feature_id[2].value <= threshold=37653.75
node_292: feature_name=SARS.CoV.2.S2	feature_id[2].value <= threshold=36044.29296875
node_293: feature_name=HuIgG_0.03	feature_id[5].value > threshold=6329.5
node_301: feature_name=HuIgG_0.03	feature_id[5].value > threshold=6562.16650390625
node_307: feature_name=Flu.B_Phu/.HA1+HA2	feature_id[11].value <= threshold=54123.5
node_308: feature_name=MoIgA_0.10	feature_id[13].value > threshold=21.166666984558105
node_310: feature_name=hCoV.NL63.S1_S2	feature_id[7].value > threshold=33291.458984375

node_314: feature_name=SARS.CoV.2.S1.mFcTag	feature_id[3].value <= threshold=50604.0
node_315: feature_name=SARS.CoV.2.Spike.RBD.rFc	feature_id[9].value > threshold=43138.767578125
Class: healthcare workers between 60 and 180 days after vaccination	
Rules_163	passed counts:1
node_0: feature_name=HuIgG_0.10	feature_id[1].value > threshold=19120.166015625
node_112: feature_name=a-HuIgG_0.03	feature_id[8].value <= threshold=19665.666015625
node_113: feature_name=SARS.CoV.2.S1.mFcTag	feature_id[3].value <= threshold=54010.166015625
node_114: feature_name=SARS.CoV.2.S1+S2	feature_id[12].value > threshold=47258.43359375
node_240: feature_name=HuIgG_0.03	feature_id[5].value > threshold=5455.5
node_266: feature_name=HuIgG_0.10	feature_id[1].value > threshold=24231.0
node_284: feature_name=HuIgG_0.03	feature_id[5].value > threshold=5469.0
node_286: feature_name=HuIgG_0.10	feature_id[1].value <= threshold=30107.666015625
node_287: feature_name=SARS.CoV.2.S1+S2	feature_id[12].value > threshold=47536.224609375
node_291: feature_name=SARS.CoV.2.S2	feature_id[2].value <= threshold=37653.75
node_292: feature_name=SARS.CoV.2.S2	feature_id[2].value <= threshold=36044.29296875
node_293: feature_name=HuIgG_0.03	feature_id[5].value > threshold=6329.5
node_301: feature_name=HuIgG_0.03	feature_id[5].value > threshold=6562.16650390625
node_307: feature_name=Flu.B_Phu/.HA1+HA2	feature_id[11].value <= threshold=54123.5
node_308: feature_name=MoIgA_0.10	feature_id[13].value <= threshold=21.166666984558105
Class: healthcare workers between 60 and 180 days after vaccination	
Rules_164	passed counts:1
node_0: feature_name=HuIgG_0.10	feature_id[1].value > threshold=19120.166015625
node_112: feature_name=a-HuIgG_0.03	feature_id[8].value <= threshold=19665.666015625

node_113: feature_name=SARS.CoV.2.S1.mFcTag	feature_id[3].value <= threshold=54010.166015625
node_114: feature_name=SARS.CoV.2.S1+S2	feature_id[12].value > threshold=47258.43359375
node_240: feature_name=HuIgG_0.03	feature_id[5].value > threshold=5455.5
node_266: feature_name=HuIgG_0.10	feature_id[1].value > threshold=24231.0
node_284: feature_name=HuIgG_0.03	feature_id[5].value > threshold=5469.0
node_286: feature_name=HuIgG_0.10	feature_id[1].value <= threshold=30107.666015625
node_287: feature_name=SARS.CoV.2.S1+S2	feature_id[12].value > threshold=47536.224609375
node_291: feature_name=SARS.CoV.2.S2	feature_id[2].value <= threshold=37653.75
node_292: feature_name=SARS.CoV.2.S2	feature_id[2].value <= threshold=36044.29296875
node_293: feature_name=HuIgG_0.03	feature_id[5].value > threshold=6329.5
node_301: feature_name=HuIgG_0.03	feature_id[5].value <= threshold=6562.16650390625
node_302: feature_name=Flu.B_Phu/.HA1+HA2	feature_id[11].value > threshold=53338.533203125
node_304: feature_name=hCoV.HKU1.NP	feature_id[10].value > threshold=12278.249755859375
Class: healthcare workers between 60 and 180 days after vaccination	
Rules_165	passed counts:1
node_0: feature_name=HuIgG_0.10	feature_id[1].value > threshold=19120.166015625
node_112: feature_name=a-HuIgG_0.03	feature_id[8].value <= threshold=19665.666015625
node_113: feature_name=SARS.CoV.2.S1.mFcTag	feature_id[3].value <= threshold=54010.166015625
node_114: feature_name=SARS.CoV.2.S1+S2	feature_id[12].value > threshold=47258.43359375
node_240: feature_name=HuIgG_0.03	feature_id[5].value > threshold=5455.5
node_266: feature_name=HuIgG_0.10	feature_id[1].value > threshold=24231.0
node_284: feature_name=HuIgG_0.03	feature_id[5].value > threshold=5469.0
node_286: feature_name=HuIgG_0.10	feature_id[1].value <= threshold=30107.666015625
node_287: feature_name=SARS.CoV.2.S1+S2	feature_id[12].value > threshold=47536.224609375

node_291: feature_name=SARS.CoV.2.S2	feature_id[2].value <= threshold=37653.75
node_292: feature_name=SARS.CoV.2.S2	feature_id[2].value <= threshold=36044.29296875
node_293: feature_name=HuIgG_0.03	feature_id[5].value <= threshold=6329.5
node_294: feature_name=Flu.B_Phu/.HA1+HA2	feature_id[11].value > threshold=50410.16796875
node_296: feature_name=HuIgG_0.03	feature_id[5].value <= threshold=5639.0
node_297: feature_name=SARS.CoV.2.S2	feature_id[2].value > threshold=30354.591796875
Class: healthcare workers between 60 and 180 days after vaccination	
Rules_166	passed counts:1
node_0: feature_name=HuIgG_0.10	feature_id[1].value > threshold=19120.166015625
node_112: feature_name=a-HuIgG_0.03	feature_id[8].value <= threshold=19665.666015625
node_113: feature_name=SARS.CoV.2.S1.mFcTag	feature_id[3].value <= threshold=54010.166015625
node_114: feature_name=SARS.CoV.2.S1+S2	feature_id[12].value > threshold=47258.43359375
node_240: feature_name=HuIgG_0.03	feature_id[5].value > threshold=5455.5
node_266: feature_name=HuIgG_0.10	feature_id[1].value > threshold=24231.0
node_284: feature_name=HuIgG_0.03	feature_id[5].value > threshold=5469.0
node_286: feature_name=HuIgG_0.10	feature_id[1].value <= threshold=30107.666015625
node_287: feature_name=SARS.CoV.2.S1+S2	feature_id[12].value > threshold=47536.224609375
node_291: feature_name=SARS.CoV.2.S2	feature_id[2].value <= threshold=37653.75
node_292: feature_name=SARS.CoV.2.S2	feature_id[2].value <= threshold=36044.29296875
node_293: feature_name=HuIgG_0.03	feature_id[5].value <= threshold=6329.5
node_294: feature_name=Flu.B_Phu/.HA1+HA2	feature_id[11].value <= threshold=50410.16796875
Class: healthcare workers between 60 and 180 days after vaccination	
Rules_167	passed counts:1

node_0: feature_name=HuIgG_0.10	feature_id[1].value > threshold=19120.166015625
node_112: feature_name=a-HuIgG_0.03	feature_id[8].value <= threshold=19665.666015625
node_113: feature_name=SARS.CoV.2.S1.mFcTag	feature_id[3].value <= threshold=54010.166015625
node_114: feature_name=SARS.CoV.2.S1+S2	feature_id[12].value > threshold=47258.43359375
node_240: feature_name=HuIgG_0.03	feature_id[5].value > threshold=5455.5
node_266: feature_name=HuIgG_0.10	feature_id[1].value > threshold=24231.0
node_284: feature_name=HuIgG_0.03	feature_id[5].value > threshold=5469.0
node_286: feature_name=HuIgG_0.10	feature_id[1].value <= threshold=30107.666015625
node_287: feature_name=SARS.CoV.2.S1+S2	feature_id[12].value <= threshold=47536.224609375
node_288: feature_name=MoIgA_0.10	feature_id[13].value <= threshold=1044.0
Class: healthcare workers within 60 days after vaccination	
Rules_168	passed counts:1
node_0: feature_name=HuIgG_0.10	feature_id[1].value > threshold=19120.166015625
node_112: feature_name=a-HuIgG_0.03	feature_id[8].value <= threshold=19665.666015625
node_113: feature_name=SARS.CoV.2.S1.mFcTag	feature_id[3].value <= threshold=54010.166015625
node_114: feature_name=SARS.CoV.2.S1+S2	feature_id[12].value > threshold=47258.43359375
node_240: feature_name=HuIgG_0.03	feature_id[5].value > threshold=5455.5
node_266: feature_name=HuIgG_0.10	feature_id[1].value <= threshold=24231.0
node_267: feature_name=hCoV.HKU1.NP	feature_id[10].value > threshold=2123.0000610351562
node_271: feature_name=SARS.CoV.S1.HisTag	feature_id[6].value > threshold=8281.83349609375
node_273: feature_name=Flu.B_Phu/.HA1+HA2	feature_id[11].value > threshold=51424.19921875
node_275: feature_name=a-HuIgG_0.30	feature_id[4].value > threshold=41169.833984375
node_277: feature_name=a-HuIgG_0.30	feature_id[4].value > threshold=42934.333984375
node_279: feature_name=a-HuIgG_0.03	feature_id[8].value <= threshold=17284.5

node_280: feature_name=SARS.CoV.2.S1.mFcTag	feature_id[3].value <= threshold=51719.75
Class: healthcare workers between 60 and 180 days after vaccination	
Rules_169	passed counts:1
node_0: feature_name=HuIgG_0.10	feature_id[1].value > threshold=19120.166015625
node_112: feature_name=a-HuIgG_0.03	feature_id[8].value <= threshold=19665.666015625
node_113: feature_name=SARS.CoV.2.S1.mFcTag	feature_id[3].value <= threshold=54010.166015625
node_114: feature_name=SARS.CoV.2.S1+S2	feature_id[12].value > threshold=47258.43359375
node_240: feature_name=HuIgG_0.03	feature_id[5].value <= threshold=5455.5
node_241: feature_name=SARS.CoV.S1.HisTag	feature_id[6].value > threshold=745.7222290039062
node_243: feature_name=SARS.CoV.2.S1+S2	feature_id[12].value > threshold=48812.91796875
node_253: feature_name=SARS.CoV.S1.HisTag	feature_id[6].value > threshold=2772.300048828125
node_257: feature_name=HuIgG_0.10	feature_id[1].value > threshold=26991.8330078125
node_263: feature_name=a-HuIgG_0.03	feature_id[8].value <= threshold=11532.0
Class: healthcare workers between 60 and 180 days after vaccination	
Rules_170	passed counts:1
node_0: feature_name=HuIgG_0.10	feature_id[1].value > threshold=19120.166015625
node_112: feature_name=a-HuIgG_0.03	feature_id[8].value <= threshold=19665.666015625
node_113: feature_name=SARS.CoV.2.S1.mFcTag	feature_id[3].value <= threshold=54010.166015625
node_114: feature_name=SARS.CoV.2.S1+S2	feature_id[12].value > threshold=47258.43359375
node_240: feature_name=HuIgG_0.03	feature_id[5].value <= threshold=5455.5
node_241: feature_name=SARS.CoV.S1.HisTag	feature_id[6].value > threshold=745.7222290039062
node_243: feature_name=SARS.CoV.2.S1+S2	feature_id[12].value > threshold=48812.91796875
node_253: feature_name=SARS.CoV.S1.HisTag	feature_id[6].value > threshold=2772.300048828125

node_257: feature_name=HuIgG_0.10	feature_id[1].value <= threshold=26991.8330078125
node_258: feature_name=SARS.CoV.2.S1.RBD.mFc	feature_id[0].value <= threshold=41406.666015625
node_259: feature_name=SARS.CoV.2.S1.RBD.mFc	feature_id[0].value > threshold=40954.3828125
Class: healthcare workers between 60 and 180 days after vaccination	
Rules_171	passed counts:1
node_0: feature_name=HuIgG_0.10	feature_id[1].value > threshold=19120.166015625
node_112: feature_name=a-HuIgG_0.03	feature_id[8].value <= threshold=19665.666015625
node_113: feature_name=SARS.CoV.2.S1.mFcTag	feature_id[3].value <= threshold=54010.166015625
node_114: feature_name=SARS.CoV.2.S1+S2	feature_id[12].value > threshold=47258.43359375
node_240: feature_name=HuIgG_0.03	feature_id[5].value <= threshold=5455.5
node_241: feature_name=SARS.CoV.S1.HisTag	feature_id[6].value > threshold=745.7222290039062
node_243: feature_name=SARS.CoV.2.S1+S2	feature_id[12].value > threshold=48812.91796875
node_253: feature_name=SARS.CoV.S1.HisTag	feature_id[6].value <= threshold=2772.300048828125
node_254: feature_name=hCoV.HKU1.NP	feature_id[10].value > threshold=3223.1332397460938
Class: healthcare workers between 60 and 180 days after vaccination	
Rules_172	passed counts:1
node_0: feature_name=HuIgG_0.10	feature_id[1].value > threshold=19120.166015625
node_112: feature_name=a-HuIgG_0.03	feature_id[8].value <= threshold=19665.666015625
node_113: feature_name=SARS.CoV.2.S1.mFcTag	feature_id[3].value <= threshold=54010.166015625
node_114: feature_name=SARS.CoV.2.S1+S2	feature_id[12].value > threshold=47258.43359375
node_240: feature_name=HuIgG_0.03	feature_id[5].value <= threshold=5455.5
node_241: feature_name=SARS.CoV.S1.HisTag	feature_id[6].value > threshold=745.7222290039062
node_243: feature_name=SARS.CoV.2.S1+S2	feature_id[12].value > threshold=48812.91796875

node_253: feature_name=SARS.CoV.S1.HisTag	feature_id[6].value <= threshold=2772.300048828125
node_254: feature_name=hCoV.HKU1.NP	feature_id[10].value <= threshold=3223.1332397460938
Class: healthcare workers within 60 days after vaccination	
Rules_173	passed counts:1
node_0: feature_name=HuIgG_0.10	feature_id[1].value > threshold=19120.166015625
node_112: feature_name=a-HuIgG_0.03	feature_id[8].value <= threshold=19665.666015625
node_113: feature_name=SARS.CoV.2.S1.mFcTag	feature_id[3].value <= threshold=54010.166015625
node_114: feature_name=SARS.CoV.2.S1+S2	feature_id[12].value > threshold=47258.43359375
node_240: feature_name=HuIgG_0.03	feature_id[5].value <= threshold=5455.5
node_241: feature_name=SARS.CoV.S1.HisTag	feature_id[6].value > threshold=745.7222290039062
node_243: feature_name=SARS.CoV.2.S1+S2	feature_id[12].value <= threshold=48812.91796875
node_244: feature_name=SARS.CoV.2.S2	feature_id[2].value > threshold=26981.416015625
node_246: feature_name=Flu.B_Phu/.HA1+HA2	feature_id[11].value > threshold=52223.0
node_250: feature_name=SARS.CoV.2.S1.mFcTag	feature_id[3].value <= threshold=49570.5
Class: healthcare workers within 60 days after vaccination	
Rules_174	passed counts:1
node_0: feature_name=HuIgG_0.10	feature_id[1].value > threshold=19120.166015625
node_112: feature_name=a-HuIgG_0.03	feature_id[8].value <= threshold=19665.666015625
node_113: feature_name=SARS.CoV.2.S1.mFcTag	feature_id[3].value <= threshold=54010.166015625
node_114: feature_name=SARS.CoV.2.S1+S2	feature_id[12].value > threshold=47258.43359375
node_240: feature_name=HuIgG_0.03	feature_id[5].value <= threshold=5455.5
node_241: feature_name=SARS.CoV.S1.HisTag	feature_id[6].value > threshold=745.7222290039062
node_243: feature_name=SARS.CoV.2.S1+S2	feature_id[12].value <= threshold=48812.91796875

node_244: feature_name=SARS.CoV.2.S2	feature_id[2].value > threshold=26981.416015625
node_246: feature_name=Flu.B_Phu/.HA1+HA2	feature_id[11].value <= threshold=52223.0
node_247: feature_name=Flu.B_Phu/.HA1+HA2	feature_id[11].value <= threshold=47753.833984375
Class: healthcare workers between 60 and 180 days after vaccination	
Rules_175	passed counts:1
node_0: feature_name=HuIgG_0.10	feature_id[1].value > threshold=19120.166015625
node_112: feature_name=a-HuIgG_0.03	feature_id[8].value <= threshold=19665.666015625
node_113: feature_name=SARS.CoV.2.S1.mFcTag	feature_id[3].value <= threshold=54010.166015625
node_114: feature_name=SARS.CoV.2.S1+S2	feature_id[12].value > threshold=47258.43359375
node_240: feature_name=HuIgG_0.03	feature_id[5].value <= threshold=5455.5
node_241: feature_name=SARS.CoV.S1.HisTag	feature_id[6].value <= threshold=745.7222290039062
Class: unvaccinated healthcare workers	
Rules_176	passed counts:1
node_0: feature_name=HuIgG_0.10	feature_id[1].value > threshold=19120.166015625
node_112: feature_name=a-HuIgG_0.03	feature_id[8].value <= threshold=19665.666015625
node_113: feature_name=SARS.CoV.2.S1.mFcTag	feature_id[3].value <= threshold=54010.166015625
node_114: feature_name=SARS.CoV.2.S1+S2	feature_id[12].value <= threshold=47258.43359375
node_115: feature_name=SARS.CoV.2.S1.RBD.mFc	feature_id[0].value > threshold=301.73333740234375
node_131: feature_name=a-HuIgG_0.03	feature_id[8].value > threshold=12099.83349609375
node_183: feature_name=Flu.B_Phu/.HA1+HA2	feature_id[11].value > threshold=41918.533203125
node_187: feature_name=SARS.CoV.2.Spike.RBD.rFc	feature_id[9].value > threshold=27438.341796875
node_197: feature_name=SARS.CoV.2.S2	feature_id[2].value <= threshold=35177.640625
node_198: feature_name=hCoV.HKU1.NP	feature_id[10].value > threshold=2645.916748046875

node_210: feature_name=Flu.B_Phu/.HA1+HA2	feature_id[11].value > threshold=54029.166015625
node_236: feature_name=SARS.CoV.2.S1.RBD.mFc	feature_id[0].value > threshold=47021.16796875
Class: healthcare workers within 60 days after vaccination	
Rules_177	passed counts:1
node_0: feature_name=HuIgG_0.10	feature_id[1].value > threshold=19120.166015625
node_112: feature_name=a-HuIgG_0.03	feature_id[8].value <= threshold=19665.666015625
node_113: feature_name=SARS.CoV.2.S1.mFcTag	feature_id[3].value <= threshold=54010.166015625
node_114: feature_name=SARS.CoV.2.S1+S2	feature_id[12].value <= threshold=47258.43359375
node_115: feature_name=SARS.CoV.2.S1.RBD.mFc	feature_id[0].value > threshold=301.73333740234375
node_131: feature_name=a-HuIgG_0.03	feature_id[8].value > threshold=12099.83349609375
node_183: feature_name=Flu.B_Phu/.HA1+HA2	feature_id[11].value > threshold=41918.533203125
node_187: feature_name=SARS.CoV.2.Spike.RBD.rFc	feature_id[9].value > threshold=27438.341796875
node_197: feature_name=SARS.CoV.2.S2	feature_id[2].value <= threshold=35177.640625
node_198: feature_name=hCoV.HKU1.NP	feature_id[10].value > threshold=2645.916748046875
node_210: feature_name=Flu.B_Phu/.HA1+HA2	feature_id[11].value <= threshold=54029.166015625
node_211: feature_name=HuIgG_0.10	feature_id[1].value > threshold=21359.5
node_213: feature_name=hCoV.NL63.S1_S2	feature_id[7].value > threshold=32523.4248046875
node_215: feature_name=HuIgG_0.10	feature_id[1].value > threshold=23274.166015625
node_217: feature_name=HuIgG_0.10	feature_id[1].value > threshold=25231.1669921875
node_227: feature_name=SARS.CoV.2.S2	feature_id[2].value > threshold=18038.125
node_231: feature_name=a-HuIgG_0.03	feature_id[8].value > threshold=12541.1669921875
node_233: feature_name=SARS.CoV.2.S1+S2	feature_id[12].value > threshold=47171.75
Class: healthcare workers between 60 and 180 days after vaccination	

Rules_178	passed counts:1
node_0: feature_name=HuIgG_0.10	feature_id[1].value > threshold=19120.166015625
node_112: feature_name=a-HuIgG_0.03	feature_id[8].value <= threshold=19665.666015625
node_113: feature_name=SARS.CoV.2.S1.mFcTag	feature_id[3].value <= threshold=54010.166015625
node_114: feature_name=SARS.CoV.2.S1+S2	feature_id[12].value <= threshold=47258.43359375
node_115: feature_name=SARS.CoV.2.S1.RBD.mFc	feature_id[0].value > threshold=301.73333740234375
node_131: feature_name=a-HuIgG_0.03	feature_id[8].value > threshold=12099.83349609375
node_183: feature_name=Flu.B_Phu/.HA1+HA2	feature_id[11].value > threshold=41918.533203125
node_187: feature_name=SARS.CoV.2.Spike.RBD.rFc	feature_id[9].value > threshold=27438.341796875
node_197: feature_name=SARS.CoV.2.S2	feature_id[2].value <= threshold=35177.640625
node_198: feature_name=hCoV.HKU1.NP	feature_id[10].value > threshold=2645.916748046875
node_210: feature_name=Flu.B_Phu/.HA1+HA2	feature_id[11].value <= threshold=54029.166015625
node_211: feature_name=HuIgG_0.10	feature_id[1].value > threshold=21359.5
node_213: feature_name=hCoV.NL63.S1_S2	feature_id[7].value > threshold=32523.4248046875
node_215: feature_name=HuIgG_0.10	feature_id[1].value > threshold=23274.166015625
node_217: feature_name=HuIgG_0.10	feature_id[1].value > threshold=25231.1669921875
node_227: feature_name=SARS.CoV.2.S2	feature_id[2].value > threshold=18038.125
node_231: feature_name=a-HuIgG_0.03	feature_id[8].value <= threshold=12541.1669921875
Class: healthcare workers between 60 and 180 days after vaccination	
Rules_179	passed counts:1
node_0: feature_name=HuIgG_0.10	feature_id[1].value > threshold=19120.166015625
node_112: feature_name=a-HuIgG_0.03	feature_id[8].value <= threshold=19665.666015625
node_113: feature_name=SARS.CoV.2.S1.mFcTag	feature_id[3].value <= threshold=54010.166015625
node_114: feature_name=SARS.CoV.2.S1+S2	feature_id[12].value <= threshold=47258.43359375

node_115: feature_name=SARS.CoV.2.S1.RBD.mFc	feature_id[0].value > threshold=301.73333740234375
node_131: feature_name=a-HuIgG_0.03	feature_id[8].value > threshold=12099.83349609375
node_183: feature_name=Flu.B_Phu/.HA1+HA2	feature_id[11].value > threshold=41918.533203125
node_187: feature_name=SARS.CoV.2.Spike.RBD.rFc	feature_id[9].value > threshold=27438.341796875
node_197: feature_name=SARS.CoV.2.S2	feature_id[2].value <= threshold=35177.640625
node_198: feature_name=hCoV.HKU1.NP	feature_id[10].value > threshold=2645.916748046875
node_210: feature_name=Flu.B_Phu/.HA1+HA2	feature_id[11].value <= threshold=54029.166015625
node_211: feature_name=HuIgG_0.10	feature_id[1].value > threshold=21359.5
node_213: feature_name=hCoV.NL63.S1_S2	feature_id[7].value > threshold=32523.4248046875
node_215: feature_name=HuIgG_0.10	feature_id[1].value > threshold=23274.166015625
node_217: feature_name=HuIgG_0.10	feature_id[1].value > threshold=25231.1669921875
node_227: feature_name=SARS.CoV.2.S2	feature_id[2].value <= threshold=18038.125
node_228: feature_name=hCoV.HKU1.NP	feature_id[10].value > threshold=11390.08349609375
Class: healthcare workers within 60 days after vaccination	
Rules_180	passed counts:1
node_0: feature_name=HuIgG_0.10	feature_id[1].value > threshold=19120.166015625
node_112: feature_name=a-HuIgG_0.03	feature_id[8].value <= threshold=19665.666015625
node_113: feature_name=SARS.CoV.2.S1.mFcTag	feature_id[3].value <= threshold=54010.166015625
node_114: feature_name=SARS.CoV.2.S1+S2	feature_id[12].value <= threshold=47258.43359375
node_115: feature_name=SARS.CoV.2.S1.RBD.mFc	feature_id[0].value > threshold=301.73333740234375
node_131: feature_name=a-HuIgG_0.03	feature_id[8].value > threshold=12099.83349609375
node_183: feature_name=Flu.B_Phu/.HA1+HA2	feature_id[11].value > threshold=41918.533203125
node_187: feature_name=SARS.CoV.2.Spike.RBD.rFc	feature_id[9].value > threshold=27438.341796875
node_197: feature_name=SARS.CoV.2.S2	feature_id[2].value <= threshold=35177.640625

node_198: feature_name=hCoV.HKU1.NP	feature_id[10].value > threshold=2645.916748046875
node_210: feature_name=Flu.B_Phu/.HA1+HA2	feature_id[11].value <= threshold=54029.166015625
node_211: feature_name=HuIgG_0.10	feature_id[1].value > threshold=21359.5
node_213: feature_name=hCoV.NL63.S1_S2	feature_id[7].value > threshold=32523.4248046875
node_215: feature_name=HuIgG_0.10	feature_id[1].value > threshold=23274.166015625
node_217: feature_name=HuIgG_0.10	feature_id[1].value <= threshold=25231.1669921875
node_218: feature_name=hCoV.HKU1.NP	feature_id[10].value > threshold=3712.083251953125
node_220: feature_name=SARS.CoV.S1.HisTag	feature_id[6].value > threshold=11696.75
Class: healthcare workers within 60 days after vaccination	
Rules_181	passed counts:1
node_0: feature_name=HuIgG_0.10	feature_id[1].value > threshold=19120.166015625
node_112: feature_name=a-HuIgG_0.03	feature_id[8].value <= threshold=19665.666015625
node_113: feature_name=SARS.CoV.2.S1.mFcTag	feature_id[3].value <= threshold=54010.166015625
node_114: feature_name=SARS.CoV.2.S1+S2	feature_id[12].value <= threshold=47258.43359375
node_115: feature_name=SARS.CoV.2.S1.RBD.mFc	feature_id[0].value > threshold=301.73333740234375
node_131: feature_name=a-HuIgG_0.03	feature_id[8].value > threshold=12099.83349609375
node_183: feature_name=Flu.B_Phu/.HA1+HA2	feature_id[11].value > threshold=41918.533203125
node_187: feature_name=SARS.CoV.2.Spike.RBD.rFc	feature_id[9].value > threshold=27438.341796875
node_197: feature_name=SARS.CoV.2.S2	feature_id[2].value <= threshold=35177.640625
node_198: feature_name=hCoV.HKU1.NP	feature_id[10].value > threshold=2645.916748046875
node_210: feature_name=Flu.B_Phu/.HA1+HA2	feature_id[11].value <= threshold=54029.166015625
node_211: feature_name=HuIgG_0.10	feature_id[1].value > threshold=21359.5
node_213: feature_name=hCoV.NL63.S1_S2	feature_id[7].value > threshold=32523.4248046875
node_215: feature_name=HuIgG_0.10	feature_id[1].value > threshold=23274.166015625

node_217: feature_name=HuIgG_0.10	feature_id[1].value <= threshold=25231.1669921875
node_218: feature_name=hCoV.HKU1.NP	feature_id[10].value > threshold=3712.083251953125
node_220: feature_name=SARS.CoV.S1.HisTag	feature_id[6].value <= threshold=11696.75
node_221: feature_name=MoIgA_0.10	feature_id[13].value > threshold=2253.6666259765625
node_223: feature_name=SARS.CoV.2.S1.RBD.mFc	feature_id[0].value > threshold=42955.33203125
Class: healthcare workers between 60 and 180 days after vaccination	
Rules_182	passed counts:1
node_0: feature_name=HuIgG_0.10	feature_id[1].value > threshold=19120.166015625
node_112: feature_name=a-HuIgG_0.03	feature_id[8].value <= threshold=19665.666015625
node_113: feature_name=SARS.CoV.2.S1.mFcTag	feature_id[3].value <= threshold=54010.166015625
node_114: feature_name=SARS.CoV.2.S1+S2	feature_id[12].value <= threshold=47258.43359375
node_115: feature_name=SARS.CoV.2.S1.RBD.mFc	feature_id[0].value > threshold=301.73333740234375
node_131: feature_name=a-HuIgG_0.03	feature_id[8].value > threshold=12099.83349609375
node_183: feature_name=Flu.B_Phu/.HA1+HA2	feature_id[11].value > threshold=41918.533203125
node_187: feature_name=SARS.CoV.2.Spike.RBD.rFc	feature_id[9].value > threshold=27438.341796875
node_197: feature_name=SARS.CoV.2.S2	feature_id[2].value <= threshold=35177.640625
node_198: feature_name=hCoV.HKU1.NP	feature_id[10].value > threshold=2645.916748046875
node_210: feature_name=Flu.B_Phu/.HA1+HA2	feature_id[11].value <= threshold=54029.166015625
node_211: feature_name=HuIgG_0.10	feature_id[1].value > threshold=21359.5
node_213: feature_name=hCoV.NL63.S1_S2	feature_id[7].value > threshold=32523.4248046875
node_215: feature_name=HuIgG_0.10	feature_id[1].value > threshold=23274.166015625
node_217: feature_name=HuIgG_0.10	feature_id[1].value <= threshold=25231.1669921875
node_218: feature_name=hCoV.HKU1.NP	feature_id[10].value > threshold=3712.083251953125
node_220: feature_name=SARS.CoV.S1.HisTag	feature_id[6].value <= threshold=11696.75

node_221: feature_name=MoIgA_0.10	feature_id[13].value > threshold=2253.6666259765625
node_223: feature_name=SARS.CoV.2.S1.RBD.mFc	feature_id[0].value <= threshold=42955.33203125
Class: healthcare workers within 60 days after vaccination	
Rules_183	passed counts:1
node_0: feature_name=HuIgG_0.10	feature_id[1].value > threshold=19120.166015625
node_112: feature_name=a-HuIgG_0.03	feature_id[8].value <= threshold=19665.666015625
node_113: feature_name=SARS.CoV.2.S1.mFcTag	feature_id[3].value <= threshold=54010.166015625
node_114: feature_name=SARS.CoV.2.S1+S2	feature_id[12].value <= threshold=47258.43359375
node_115: feature_name=SARS.CoV.2.S1.RBD.mFc	feature_id[0].value > threshold=301.73333740234375
node_131: feature_name=a-HuIgG_0.03	feature_id[8].value > threshold=12099.83349609375
node_183: feature_name=Flu.B_Phu/.HA1+HA2	feature_id[11].value > threshold=41918.533203125
node_187: feature_name=SARS.CoV.2.Spike.RBD.rFc	feature_id[9].value <= threshold=27438.341796875
node_188: feature_name=Flu.B_Phu/.HA1+HA2	feature_id[11].value > threshold=48840.19921875
node_192: feature_name=MoIgA_0.10	feature_id[13].value <= threshold=154.99999618530273
node_193: feature_name=MoIgA_0.10	feature_id[13].value > threshold=88.99999809265137
Class: unvaccinated healthcare workers	
Rules_184	passed counts:1
node_0: feature_name=HuIgG_0.10	feature_id[1].value > threshold=19120.166015625
node_112: feature_name=a-HuIgG_0.03	feature_id[8].value <= threshold=19665.666015625
node_113: feature_name=SARS.CoV.2.S1.mFcTag	feature_id[3].value <= threshold=54010.166015625
node_114: feature_name=SARS.CoV.2.S1+S2	feature_id[12].value <= threshold=47258.43359375
node_115: feature_name=SARS.CoV.2.S1.RBD.mFc	feature_id[0].value > threshold=301.73333740234375
node_131: feature_name=a-HuIgG_0.03	feature_id[8].value > threshold=12099.83349609375

node_183: feature_name=Flu.B_Phu/.HA1+HA2	feature_id[11].value > threshold=41918.533203125
node_187: feature_name=SARS.CoV.2.Spike.RBD.rFc	feature_id[9].value <= threshold=27438.341796875
node_188: feature_name=Flu.B_Phu/.HA1+HA2	feature_id[11].value > threshold=48840.19921875
node_192: feature_name=MoIgA_0.10	feature_id[13].value <= threshold=154.99999618530273
node_193: feature_name=MoIgA_0.10	feature_id[13].value <= threshold=88.99999809265137
Class: healthcare workers within 60 days after vaccination	
Rules_185	passed counts:1
node_0: feature_name=HuIgG_0.10	feature_id[1].value > threshold=19120.166015625
node_112: feature_name=a-HuIgG_0.03	feature_id[8].value <= threshold=19665.666015625
node_113: feature_name=SARS.CoV.2.S1.mFcTag	feature_id[3].value <= threshold=54010.166015625
node_114: feature_name=SARS.CoV.2.S1+S2	feature_id[12].value <= threshold=47258.43359375
node_115: feature_name=SARS.CoV.2.S1.RBD.mFc	feature_id[0].value > threshold=301.73333740234375
node_131: feature_name=a-HuIgG_0.03	feature_id[8].value > threshold=12099.83349609375
node_183: feature_name=Flu.B_Phu/.HA1+HA2	feature_id[11].value > threshold=41918.533203125
node_187: feature_name=SARS.CoV.2.Spike.RBD.rFc	feature_id[9].value <= threshold=27438.341796875
node_188: feature_name=Flu.B_Phu/.HA1+HA2	feature_id[11].value <= threshold=48840.19921875
node_189: feature_name=SARS.CoV.2.Spike.RBD.rFc	feature_id[9].value > threshold=22062.0673828125
Class: healthcare workers over 180 days after vaccination	
Rules_186	passed counts:1
node_0: feature_name=HuIgG_0.10	feature_id[1].value > threshold=19120.166015625
node_112: feature_name=a-HuIgG_0.03	feature_id[8].value <= threshold=19665.666015625
node_113: feature_name=SARS.CoV.2.S1.mFcTag	feature_id[3].value <= threshold=54010.166015625
node_114: feature_name=SARS.CoV.2.S1+S2	feature_id[12].value <= threshold=47258.43359375

node_115: feature_name=SARS.CoV.2.S1.RBD.mFc	feature_id[0].value > threshold=301.73333740234375
node_131: feature_name=a-HuIgG_0.03	feature_id[8].value > threshold=12099.83349609375
node_183: feature_name=Flu.B_Phu/.HA1+HA2	feature_id[11].value > threshold=41918.533203125
node_187: feature_name=SARS.CoV.2.Spike.RBD.rFc	feature_id[9].value <= threshold=27438.341796875
node_188: feature_name=Flu.B_Phu/.HA1+HA2	feature_id[11].value <= threshold=48840.19921875
node_189: feature_name=SARS.CoV.2.Spike.RBD.rFc	feature_id[9].value <= threshold=22062.0673828125
Class: unvaccinated healthcare workers	
Rules_187	passed counts:1
node_0: feature_name=HuIgG_0.10	feature_id[1].value > threshold=19120.166015625
node_112: feature_name=a-HuIgG_0.03	feature_id[8].value <= threshold=19665.666015625
node_113: feature_name=SARS.CoV.2.S1.mFcTag	feature_id[3].value <= threshold=54010.166015625
node_114: feature_name=SARS.CoV.2.S1+S2	feature_id[12].value <= threshold=47258.43359375
node_115: feature_name=SARS.CoV.2.S1.RBD.mFc	feature_id[0].value > threshold=301.73333740234375
node_131: feature_name=a-HuIgG_0.03	feature_id[8].value > threshold=12099.83349609375
node_183: feature_name=Flu.B_Phu/.HA1+HA2	feature_id[11].value <= threshold=41918.533203125
node_184: feature_name=a-HuIgG_0.03	feature_id[8].value <= threshold=14683.6669921875
Class: healthcare workers between 60 and 180 days after vaccination	
Rules_188	passed counts:1
node_0: feature_name=HuIgG_0.10	feature_id[1].value > threshold=19120.166015625
node_112: feature_name=a-HuIgG_0.03	feature_id[8].value <= threshold=19665.666015625
node_113: feature_name=SARS.CoV.2.S1.mFcTag	feature_id[3].value <= threshold=54010.166015625
node_114: feature_name=SARS.CoV.2.S1+S2	feature_id[12].value <= threshold=47258.43359375
node_115: feature_name=SARS.CoV.2.S1.RBD.mFc	feature_id[0].value > threshold=301.73333740234375

node_131: feature_name=a-HuIgG_0.03	feature_id[8].value <= threshold=12099.83349609375
node_132: feature_name=SARS.CoV.2.S2	feature_id[2].value > threshold=8858.0751953125
node_134: feature_name=Flu.B_Phu/.HA1+HA2	feature_id[11].value > threshold=53839.16796875
node_180: feature_name=a-HuIgG_0.30	feature_id[4].value <= threshold=40436.333984375
Class: healthcare workers within 60 days after vaccination	
Rules_189	passed counts:1
node_0: feature_name=HuIgG_0.10	feature_id[1].value > threshold=19120.166015625
node_112: feature_name=a-HuIgG_0.03	feature_id[8].value <= threshold=19665.666015625
node_113: feature_name=SARS.CoV.2.S1.mFcTag	feature_id[3].value <= threshold=54010.166015625
node_114: feature_name=SARS.CoV.2.S1+S2	feature_id[12].value <= threshold=47258.43359375
node_115: feature_name=SARS.CoV.2.S1.RBD.mFc	feature_id[0].value > threshold=301.73333740234375
node_131: feature_name=a-HuIgG_0.03	feature_id[8].value <= threshold=12099.83349609375
node_132: feature_name=SARS.CoV.2.S2	feature_id[2].value > threshold=8858.0751953125
node_134: feature_name=Flu.B_Phu/.HA1+HA2	feature_id[11].value <= threshold=53839.16796875
node_135: feature_name=hCoV.HKU1.NP	feature_id[10].value > threshold=1010.1666564941406
node_145: feature_name=hCoV.HKU1.NP	feature_id[10].value > threshold=15374.16650390625
node_177: feature_name=a-HuIgG_0.03	feature_id[8].value > threshold=11866.5
Class: healthcare workers within 60 days after vaccination	
Rules_190	passed counts:1
node_0: feature_name=HuIgG_0.10	feature_id[1].value > threshold=19120.166015625
node_112: feature_name=a-HuIgG_0.03	feature_id[8].value <= threshold=19665.666015625
node_113: feature_name=SARS.CoV.2.S1.mFcTag	feature_id[3].value <= threshold=54010.166015625
node_114: feature_name=SARS.CoV.2.S1+S2	feature_id[12].value <= threshold=47258.43359375

node_115: feature_name=SARS.CoV.2.S1.RBD.mFc	feature_id[0].value > threshold=301.73333740234375
node_131: feature_name=a-HuIgG_0.03	feature_id[8].value <= threshold=12099.83349609375
node_132: feature_name=SARS.CoV.2.S2	feature_id[2].value > threshold=8858.0751953125
node_134: feature_name=Flu.B_Phu/.HA1+HA2	feature_id[11].value <= threshold=53839.16796875
node_135: feature_name=hCoV.HKU1.NP	feature_id[10].value > threshold=1010.1666564941406
node_145: feature_name=hCoV.HKU1.NP	feature_id[10].value <= threshold=15374.16650390625
node_146: feature_name=SARS.CoV.2.S1.mFcTag	feature_id[3].value > threshold=50683.0
node_168: feature_name=HuIgG_0.10	feature_id[1].value > threshold=20669.833984375
node_170: feature_name=SARS.CoV.2.S1.mFcTag	feature_id[3].value > threshold=50874.666015625
node_172: feature_name=SARS.CoV.S1.HisTag	feature_id[6].value <= threshold=3796.25
node_173: feature_name=a-HuIgG_0.03	feature_id[8].value <= threshold=10618.0
Class: healthcare workers within 60 days after vaccination	
Rules_191	passed counts:1
node_0: feature_name=HuIgG_0.10	feature_id[1].value > threshold=19120.166015625
node_112: feature_name=a-HuIgG_0.03	feature_id[8].value <= threshold=19665.666015625
node_113: feature_name=SARS.CoV.2.S1.mFcTag	feature_id[3].value <= threshold=54010.166015625
node_114: feature_name=SARS.CoV.2.S1+S2	feature_id[12].value <= threshold=47258.43359375
node_115: feature_name=SARS.CoV.2.S1.RBD.mFc	feature_id[0].value > threshold=301.73333740234375
node_131: feature_name=a-HuIgG_0.03	feature_id[8].value <= threshold=12099.83349609375
node_132: feature_name=SARS.CoV.2.S2	feature_id[2].value > threshold=8858.0751953125
node_134: feature_name=Flu.B_Phu/.HA1+HA2	feature_id[11].value <= threshold=53839.16796875
node_135: feature_name=hCoV.HKU1.NP	feature_id[10].value > threshold=1010.1666564941406
node_145: feature_name=hCoV.HKU1.NP	feature_id[10].value <= threshold=15374.16650390625
node_146: feature_name=SARS.CoV.2.S1.mFcTag	feature_id[3].value <= threshold=50683.0

node_147: feature_name=SARS.CoV.2.Spike.RBD.rFc	feature_id[9].value > threshold=45822.583984375
Class: healthcare workers between 60 and 180 days after vaccination	
Rules_192	passed counts:1
node_0: feature_name=HuIgG_0.10	feature_id[1].value > threshold=19120.166015625
node_112: feature_name=a-HuIgG_0.03	feature_id[8].value <= threshold=19665.666015625
node_113: feature_name=SARS.CoV.2.S1.mFcTag	feature_id[3].value <= threshold=54010.166015625
node_114: feature_name=SARS.CoV.2.S1+S2	feature_id[12].value <= threshold=47258.43359375
node_115: feature_name=SARS.CoV.2.S1.RBD.mFc	feature_id[0].value > threshold=301.73333740234375
node_131: feature_name=a-HuIgG_0.03	feature_id[8].value <= threshold=12099.83349609375
node_132: feature_name=SARS.CoV.2.S2	feature_id[2].value > threshold=8858.0751953125
node_134: feature_name=Flu.B_Phu/.HA1+HA2	feature_id[11].value <= threshold=53839.16796875
node_135: feature_name=hCoV.HKU1.NP	feature_id[10].value > threshold=1010.1666564941406
node_145: feature_name=hCoV.HKU1.NP	feature_id[10].value <= threshold=15374.16650390625
node_146: feature_name=SARS.CoV.2.S1.mFcTag	feature_id[3].value <= threshold=50683.0
node_147: feature_name=SARS.CoV.2.Spike.RBD.rFc	feature_id[9].value <= threshold=45822.583984375
node_148: feature_name=SARS.CoV.2.S2	feature_id[2].value > threshold=10212.125
node_154: feature_name=Flu.B_Phu/.HA1+HA2	feature_id[11].value > threshold=49488.1171875
node_162: feature_name=MoIgA_0.10	feature_id[13].value > threshold=2877.1666259765625
node_164: feature_name=SARS.CoV.2.Spike.RBD.rFc	feature_id[9].value > threshold=38138.451171875
Class: healthcare workers over 180 days after vaccination	
Rules_193	passed counts:1
node_0: feature_name=HuIgG_0.10	feature_id[1].value > threshold=19120.166015625
node_112: feature_name=a-HuIgG_0.03	feature_id[8].value <= threshold=19665.666015625

node_113: feature_name=SARS.CoV.2.S1.mFcTag	feature_id[3].value <= threshold=54010.166015625
node_114: feature_name=SARS.CoV.2.S1+S2	feature_id[12].value <= threshold=47258.43359375
node_115: feature_name=SARS.CoV.2.S1.RBD.mFc	feature_id[0].value > threshold=301.73333740234375
node_131: feature_name=a-HuIgG_0.03	feature_id[8].value <= threshold=12099.83349609375
node_132: feature_name=SARS.CoV.2.S2	feature_id[2].value > threshold=8858.0751953125
node_134: feature_name=Flu.B_Phu/.HA1+HA2	feature_id[11].value <= threshold=53839.16796875
node_135: feature_name=hCoV.HKU1.NP	feature_id[10].value > threshold=1010.1666564941406
node_145: feature_name=hCoV.HKU1.NP	feature_id[10].value <= threshold=15374.16650390625
node_146: feature_name=SARS.CoV.2.S1.mFcTag	feature_id[3].value <= threshold=50683.0
node_147: feature_name=SARS.CoV.2.Spike.RBD.rFc	feature_id[9].value <= threshold=45822.583984375
node_148: feature_name=SARS.CoV.2.S2	feature_id[2].value > threshold=10212.125
node_154: feature_name=Flu.B_Phu/.HA1+HA2	feature_id[11].value <= threshold=49488.1171875
node_155: feature_name=MoIgA_0.10	feature_id[13].value > threshold=177.66666412353516
node_157: feature_name=a-HuIgG_0.03	feature_id[8].value > threshold=11251.0
node_159: feature_name=SARS.CoV.S1.HisTag	feature_id[6].value > threshold=5051.91650390625
Class: healthcare workers between 60 and 180 days after vaccination	
Rules_194	passed counts:1
node_0: feature_name=HuIgG_0.10	feature_id[1].value > threshold=19120.166015625
node_112: feature_name=a-HuIgG_0.03	feature_id[8].value <= threshold=19665.666015625
node_113: feature_name=SARS.CoV.2.S1.mFcTag	feature_id[3].value <= threshold=54010.166015625
node_114: feature_name=SARS.CoV.2.S1+S2	feature_id[12].value <= threshold=47258.43359375
node_115: feature_name=SARS.CoV.2.S1.RBD.mFc	feature_id[0].value > threshold=301.73333740234375
node_131: feature_name=a-HuIgG_0.03	feature_id[8].value <= threshold=12099.83349609375
node_132: feature_name=SARS.CoV.2.S2	feature_id[2].value > threshold=8858.0751953125

node_134: feature_name=Flu.B_Phu/.HA1+HA2	feature_id[11].value <= threshold=53839.16796875
node_135: feature_name=hCoV.HKU1.NP	feature_id[10].value > threshold=1010.1666564941406
node_145: feature_name=hCoV.HKU1.NP	feature_id[10].value <= threshold=15374.16650390625
node_146: feature_name=SARS.CoV.2.S1.mFcTag	feature_id[3].value <= threshold=50683.0
node_147: feature_name=SARS.CoV.2.Spike.RBD.rFc	feature_id[9].value <= threshold=45822.583984375
node_148: feature_name=SARS.CoV.2.S2	feature_id[2].value > threshold=10212.125
node_154: feature_name=Flu.B_Phu/.HA1+HA2	feature_id[11].value <= threshold=49488.1171875
node_155: feature_name=MoIgA_0.10	feature_id[13].value > threshold=177.66666412353516
node_157: feature_name=a-HuIgG_0.03	feature_id[8].value > threshold=11251.0
node_159: feature_name=SARS.CoV.S1.HisTag	feature_id[6].value <= threshold=5051.91650390625
Class: healthcare workers within 60 days after vaccination	
Rules_195	passed counts:1
node_0: feature_name=HuIgG_0.10	feature_id[1].value > threshold=19120.166015625
node_112: feature_name=a-HuIgG_0.03	feature_id[8].value <= threshold=19665.666015625
node_113: feature_name=SARS.CoV.2.S1.mFcTag	feature_id[3].value <= threshold=54010.166015625
node_114: feature_name=SARS.CoV.2.S1+S2	feature_id[12].value <= threshold=47258.43359375
node_115: feature_name=SARS.CoV.2.S1.RBD.mFc	feature_id[0].value > threshold=301.73333740234375
node_131: feature_name=a-HuIgG_0.03	feature_id[8].value <= threshold=12099.83349609375
node_132: feature_name=SARS.CoV.2.S2	feature_id[2].value > threshold=8858.0751953125
node_134: feature_name=Flu.B_Phu/.HA1+HA2	feature_id[11].value <= threshold=53839.16796875
node_135: feature_name=hCoV.HKU1.NP	feature_id[10].value > threshold=1010.1666564941406
node_145: feature_name=hCoV.HKU1.NP	feature_id[10].value <= threshold=15374.16650390625
node_146: feature_name=SARS.CoV.2.S1.mFcTag	feature_id[3].value <= threshold=50683.0
node_147: feature_name=SARS.CoV.2.Spike.RBD.rFc	feature_id[9].value <= threshold=45822.583984375

node_148: feature_name=SARS.CoV.2.S2	feature_id[2].value <= threshold=10212.125
node_149: feature_name=HuIgG_0.03	feature_id[5].value > threshold=4160.666748046875
node_151: feature_name=SARS.CoV.2.Spike.RBD.rFc	feature_id[9].value <= threshold=20605.1162109375
Class: healthcare workers within 60 days after vaccination	
Rules_196	passed counts:1
node_0: feature_name=HuIgG_0.10	feature_id[1].value > threshold=19120.166015625
node_112: feature_name=a-HuIgG_0.03	feature_id[8].value <= threshold=19665.666015625
node_113: feature_name=SARS.CoV.2.S1.mFcTag	feature_id[3].value <= threshold=54010.166015625
node_114: feature_name=SARS.CoV.2.S1+S2	feature_id[12].value <= threshold=47258.43359375
node_115: feature_name=SARS.CoV.2.S1.RBD.mFc	feature_id[0].value > threshold=301.73333740234375
node_131: feature_name=a-HuIgG_0.03	feature_id[8].value <= threshold=12099.83349609375
node_132: feature_name=SARS.CoV.2.S2	feature_id[2].value > threshold=8858.0751953125
node_134: feature_name=Flu.B_Phu/.HA1+HA2	feature_id[11].value <= threshold=53839.16796875
node_135: feature_name=hCoV.HKU1.NP	feature_id[10].value <= threshold=1010.1666564941406
node_136: feature_name=MoIgA_0.10	feature_id[13].value > threshold=497.8333282470703
node_142: feature_name=SARS.CoV.2.S1.mFcTag	feature_id[3].value > threshold=50462.283203125
Class: healthcare workers within 60 days after vaccination	
Rules_197	passed counts:1
node_0: feature_name=HuIgG_0.10	feature_id[1].value > threshold=19120.166015625
node_112: feature_name=a-HuIgG_0.03	feature_id[8].value <= threshold=19665.666015625
node_113: feature_name=SARS.CoV.2.S1.mFcTag	feature_id[3].value <= threshold=54010.166015625
node_114: feature_name=SARS.CoV.2.S1+S2	feature_id[12].value <= threshold=47258.43359375
node_115: feature_name=SARS.CoV.2.S1.RBD.mFc	feature_id[0].value > threshold=301.73333740234375

node_131: feature_name=a-HuIgG_0.03	feature_id[8].value <= threshold=12099.83349609375
node_132: feature_name=SARS.CoV.2.S2	feature_id[2].value > threshold=8858.0751953125
node_134: feature_name=Flu.B_Phu/.HA1+HA2	feature_id[11].value <= threshold=53839.16796875
node_135: feature_name=hCoV.HKU1.NP	feature_id[10].value <= threshold=1010.1666564941406
node_136: feature_name=MoIgA_0.10	feature_id[13].value <= threshold=497.8333282470703
node_137: feature_name=MoIgA_0.10	feature_id[13].value <= threshold=153.0
node_138: feature_name=SARS.CoV.S1.HisTag	feature_id[6].value > threshold=14632.4169921875
Class: healthcare workers within 60 days after vaccination	
Rules_198	passed counts:1
node_0: feature_name=HuIgG_0.10	feature_id[1].value > threshold=19120.166015625
node_112: feature_name=a-HuIgG_0.03	feature_id[8].value <= threshold=19665.666015625
node_113: feature_name=SARS.CoV.2.S1.mFcTag	feature_id[3].value <= threshold=54010.166015625
node_114: feature_name=SARS.CoV.2.S1+S2	feature_id[12].value <= threshold=47258.43359375
node_115: feature_name=SARS.CoV.2.S1.RBD.mFc	feature_id[0].value <= threshold=301.73333740234375
node_116: feature_name=SARS.CoV.2.S1+S2	feature_id[12].value <= threshold=3298.25
node_117: feature_name=a-HuIgG_0.30	feature_id[4].value <= threshold=2425.3333129882812
node_118: feature_name=SARS.CoV.2.S2	feature_id[2].value > threshold=-520.3333435058594
Class: healthcare workers between 60 and 180 days after vaccination	
Rules_199	passed counts:1
node_0: feature_name=HuIgG_0.10	feature_id[1].value > threshold=19120.166015625
node_112: feature_name=a-HuIgG_0.03	feature_id[8].value <= threshold=19665.666015625
node_113: feature_name=SARS.CoV.2.S1.mFcTag	feature_id[3].value <= threshold=54010.166015625
node_114: feature_name=SARS.CoV.2.S1+S2	feature_id[12].value <= threshold=47258.43359375

node_115: feature_name=SARS.CoV.2.S1.RBD.mFc	feature_id[0].value <= threshold=301.73333740234375
node_116: feature_name=SARS.CoV.2.S1+S2	feature_id[12].value <= threshold=3298.25
node_117: feature_name=a-HuIgG_0.30	feature_id[4].value <= threshold=2425.3333129882812
node_118: feature_name=SARS.CoV.2.S2	feature_id[2].value <= threshold=-520.3333435058594
Class: healthcare workers over 180 days after vaccination	
Rules_200	passed counts:1
node_0: feature_name=HuIgG_0.10	feature_id[1].value <= threshold=19120.166015625
node_1: feature_name=SARS.CoV.2.S1.mFcTag	feature_id[3].value > threshold=5354.388916015625
node_9: feature_name=a-HuIgG_0.30	feature_id[4].value > threshold=33726.833984375
node_55: feature_name=SARS.CoV.2.S1.mFcTag	feature_id[3].value > threshold=43185.68359375
node_67: feature_name=a-HuIgG_0.03	feature_id[8].value > threshold=10858.0
node_83: feature_name=HuIgG_0.03	feature_id[5].value > threshold=3796.0
node_103: feature_name=SARS.CoV.2.S1+S2	feature_id[12].value > threshold=49967.91796875
node_107: feature_name=hCoV.NL63.S1_S2	feature_id[7].value <= threshold=28891.8330078125
node_108: feature_name=HuIgG_0.10	feature_id[1].value > threshold=19058.166015625
Class: healthcare workers over 180 days after vaccination	
Rules_201	passed counts:1
node_0: feature_name=HuIgG_0.10	feature_id[1].value <= threshold=19120.166015625
node_1: feature_name=SARS.CoV.2.S1.mFcTag	feature_id[3].value > threshold=5354.388916015625
node_9: feature_name=a-HuIgG_0.30	feature_id[4].value > threshold=33726.833984375
node_55: feature_name=SARS.CoV.2.S1.mFcTag	feature_id[3].value > threshold=43185.68359375
node_67: feature_name=a-HuIgG_0.03	feature_id[8].value > threshold=10858.0
node_83: feature_name=HuIgG_0.03	feature_id[5].value > threshold=3796.0

node_103: feature_name=SARS.CoV.2.S1+S2	feature_id[12].value > threshold=49967.91796875
node_107: feature_name=hCoV.NL63.S1_S2	feature_id[7].value <= threshold=28891.8330078125
node_108: feature_name=HuIgG_0.10	feature_id[1].value <= threshold=19058.166015625
Class: healthcare workers between 60 and 180 days after vaccination	
Rules_202	passed counts:1
node_0: feature_name=HuIgG_0.10	feature_id[1].value <= threshold=19120.166015625
node_1: feature_name=SARS.CoV.2.S1.mFcTag	feature_id[3].value > threshold=5354.388916015625
node_9: feature_name=a-HuIgG_0.30	feature_id[4].value > threshold=33726.833984375
node_55: feature_name=SARS.CoV.2.S1.mFcTag	feature_id[3].value > threshold=43185.68359375
node_67: feature_name=a-HuIgG_0.03	feature_id[8].value > threshold=10858.0
node_83: feature_name=HuIgG_0.03	feature_id[5].value > threshold=3796.0
node_103: feature_name=SARS.CoV.2.S1+S2	feature_id[12].value <= threshold=49967.91796875
node_104: feature_name=HuIgG_0.10	feature_id[1].value <= threshold=17143.833984375
Class: healthcare workers within 60 days after vaccination	
Rules_203	passed counts:1
node_0: feature_name=HuIgG_0.10	feature_id[1].value <= threshold=19120.166015625
node_1: feature_name=SARS.CoV.2.S1.mFcTag	feature_id[3].value > threshold=5354.388916015625
node_9: feature_name=a-HuIgG_0.30	feature_id[4].value > threshold=33726.833984375
node_55: feature_name=SARS.CoV.2.S1.mFcTag	feature_id[3].value > threshold=43185.68359375
node_67: feature_name=a-HuIgG_0.03	feature_id[8].value > threshold=10858.0
node_83: feature_name=HuIgG_0.03	feature_id[5].value <= threshold=3796.0
node_84: feature_name=hCoV.NL63.S1_S2	feature_id[7].value > threshold=29814.5
node_96: feature_name=hCoV.NL63.S1_S2	feature_id[7].value > threshold=34078.0

node_98: feature_name=SARS.CoV.S1.HisTag	feature_id[6].value > threshold=9674.150390625
node_100: feature_name=SARS.CoV.2.S1.mFcTag	feature_id[3].value > threshold=55180.015625
Class: healthcare workers between 60 and 180 days after vaccination	
Rules_204	passed counts:1
node_0: feature_name=HuIgG_0.10	feature_id[1].value <= threshold=19120.166015625
node_1: feature_name=SARS.CoV.2.S1.mFcTag	feature_id[3].value > threshold=5354.388916015625
node_9: feature_name=a-HuIgG_0.30	feature_id[4].value > threshold=33726.833984375
node_55: feature_name=SARS.CoV.2.S1.mFcTag	feature_id[3].value > threshold=43185.68359375
node_67: feature_name=a-HuIgG_0.03	feature_id[8].value > threshold=10858.0
node_83: feature_name=HuIgG_0.03	feature_id[5].value <= threshold=3796.0
node_84: feature_name=hCoV.NL63.S1_S2	feature_id[7].value <= threshold=29814.5
node_85: feature_name=hCoV.NL63.S1_S2	feature_id[7].value > threshold=22280.4169921875
node_89: feature_name=Flu.B_Phu/.HA1+HA2	feature_id[11].value > threshold=41117.93359375
node_91: feature_name=SARS.CoV.S1.HisTag	feature_id[6].value > threshold=18680.75
node_93: feature_name=SARS.CoV.2.S1.mFcTag	feature_id[3].value > threshold=53523.583984375
Class: healthcare workers between 60 and 180 days after vaccination	
Rules_205	passed counts:1
node_0: feature_name=HuIgG_0.10	feature_id[1].value <= threshold=19120.166015625
node_1: feature_name=SARS.CoV.2.S1.mFcTag	feature_id[3].value > threshold=5354.388916015625
node_9: feature_name=a-HuIgG_0.30	feature_id[4].value > threshold=33726.833984375
node_55: feature_name=SARS.CoV.2.S1.mFcTag	feature_id[3].value > threshold=43185.68359375
node_67: feature_name=a-HuIgG_0.03	feature_id[8].value > threshold=10858.0
node_83: feature_name=HuIgG_0.03	feature_id[5].value <= threshold=3796.0

node_84: feature_name=hCoV.NL63.S1_S2	feature_id[7].value <= threshold=29814.5
node_85: feature_name=hCoV.NL63.S1_S2	feature_id[7].value > threshold=22280.4169921875
node_89: feature_name=Flu.B_Phu/.HA1+HA2	feature_id[11].value > threshold=41117.93359375
node_91: feature_name=SARS.CoV.S1.HisTag	feature_id[6].value > threshold=18680.75
node_93: feature_name=SARS.CoV.2.S1.mFcTag	feature_id[3].value <= threshold=53523.583984375
Class: healthcare workers over 180 days after vaccination	
Rules_206	passed counts:1
node_0: feature_name=HuIgG_0.10	feature_id[1].value <= threshold=19120.166015625
node_1: feature_name=SARS.CoV.2.S1.mFcTag	feature_id[3].value > threshold=5354.388916015625
node_9: feature_name=a-HuIgG_0.30	feature_id[4].value > threshold=33726.833984375
node_55: feature_name=SARS.CoV.2.S1.mFcTag	feature_id[3].value > threshold=43185.68359375
node_67: feature_name=a-HuIgG_0.03	feature_id[8].value > threshold=10858.0
node_83: feature_name=HuIgG_0.03	feature_id[5].value <= threshold=3796.0
node_84: feature_name=hCoV.NL63.S1_S2	feature_id[7].value <= threshold=29814.5
node_85: feature_name=hCoV.NL63.S1_S2	feature_id[7].value > threshold=22280.4169921875
node_89: feature_name=Flu.B_Phu/.HA1+HA2	feature_id[11].value <= threshold=41117.93359375
Class: healthcare workers over 180 days after vaccination	
Rules_207	passed counts:1
node_0: feature_name=HuIgG_0.10	feature_id[1].value <= threshold=19120.166015625
node_1: feature_name=SARS.CoV.2.S1.mFcTag	feature_id[3].value > threshold=5354.388916015625
node_9: feature_name=a-HuIgG_0.30	feature_id[4].value > threshold=33726.833984375
node_55: feature_name=SARS.CoV.2.S1.mFcTag	feature_id[3].value > threshold=43185.68359375
node_67: feature_name=a-HuIgG_0.03	feature_id[8].value > threshold=10858.0

node_83: feature_name=HuIgG_0.03	feature_id[5].value <= threshold=3796.0
node_84: feature_name=hCoV.NL63.S1_S2	feature_id[7].value <= threshold=29814.5
node_85: feature_name=hCoV.NL63.S1_S2	feature_id[7].value <= threshold=22280.4169921875
node_86: feature_name=Flu.B_Phu/.HA1+HA2	feature_id[11].value > threshold=50957.43359375
Class: healthcare workers between 60 and 180 days after vaccination	
Rules_208	passed counts:1
node_0: feature_name=HuIgG_0.10	feature_id[1].value <= threshold=19120.166015625
node_1: feature_name=SARS.CoV.2.S1.mFcTag	feature_id[3].value > threshold=5354.388916015625
node_9: feature_name=a-HuIgG_0.30	feature_id[4].value > threshold=33726.833984375
node_55: feature_name=SARS.CoV.2.S1.mFcTag	feature_id[3].value > threshold=43185.68359375
node_67: feature_name=a-HuIgG_0.03	feature_id[8].value <= threshold=10858.0
node_68: feature_name=MoIgA_0.10	feature_id[13].value > threshold=246.83333587646484
node_76: feature_name=SARS.CoV.2.Spike.RBD.rFc	feature_id[9].value > threshold=54060.41796875
Class: healthcare workers over 180 days after vaccination	
Rules_209	passed counts:1
node_0: feature_name=HuIgG_0.10	feature_id[1].value <= threshold=19120.166015625
node_1: feature_name=SARS.CoV.2.S1.mFcTag	feature_id[3].value > threshold=5354.388916015625
node_9: feature_name=a-HuIgG_0.30	feature_id[4].value > threshold=33726.833984375
node_55: feature_name=SARS.CoV.2.S1.mFcTag	feature_id[3].value > threshold=43185.68359375
node_67: feature_name=a-HuIgG_0.03	feature_id[8].value <= threshold=10858.0
node_68: feature_name=MoIgA_0.10	feature_id[13].value <= threshold=246.83333587646484
node_69: feature_name=hCoV.HKU1.NP	feature_id[10].value > threshold=1960.7500610351562
node_73: feature_name=hCoV.NL63.S1_S2	feature_id[7].value <= threshold=25846.875

Class: healthcare workers within 60 days after vaccination	
Rules_210	passed counts:1
node_0: feature_name=HuIgG_0.10	feature_id[1].value <= threshold=19120.166015625
node_1: feature_name=SARS.CoV.2.S1.mFcTag	feature_id[3].value > threshold=5354.388916015625
node_9: feature_name=a-HuIgG_0.30	feature_id[4].value > threshold=33726.833984375
node_55: feature_name=SARS.CoV.2.S1.mFcTag	feature_id[3].value > threshold=43185.68359375
node_67: feature_name=a-HuIgG_0.03	feature_id[8].value <= threshold=10858.0
node_68: feature_name=MoIgA_0.10	feature_id[13].value <= threshold=246.83333587646484
node_69: feature_name=hCoV.HKU1.NP	feature_id[10].value <= threshold=1960.7500610351562
node_70: feature_name=hCoV.HKU1.NP	feature_id[10].value <= threshold=1023.75
Class: healthcare workers over 180 days after vaccination	
Rules_211	passed counts:1
node_0: feature_name=HuIgG_0.10	feature_id[1].value <= threshold=19120.166015625
node_1: feature_name=SARS.CoV.2.S1.mFcTag	feature_id[3].value > threshold=5354.388916015625
node_9: feature_name=a-HuIgG_0.30	feature_id[4].value > threshold=33726.833984375
node_55: feature_name=SARS.CoV.2.S1.mFcTag	feature_id[3].value <= threshold=43185.68359375
node_56: feature_name=SARS.CoV.S1.HisTag	feature_id[6].value > threshold=1493.4166870117188
node_62: feature_name=SARS.CoV.2.S1+S2	feature_id[12].value <= threshold=22078.1669921875
node_63: feature_name=SARS.CoV.2.S1+S2	feature_id[12].value > threshold=21810.333984375
Class: healthcare workers between 60 and 180 days after vaccination	
Rules_212	passed counts:1
node_0: feature_name=HuIgG_0.10	feature_id[1].value <= threshold=19120.166015625

node_1: feature_name=SARS.CoV.2.S1.mFcTag	feature_id[3].value > threshold=5354.388916015625
node_9: feature_name=a-HuIgG_0.30	feature_id[4].value > threshold=33726.833984375
node_55: feature_name=SARS.CoV.2.S1.mFcTag	feature_id[3].value <= threshold=43185.68359375
node_56: feature_name=SARS.CoV.S1.HisTag	feature_id[6].value <= threshold=1493.4166870117188
node_57: feature_name=SARS.CoV.2.S1.mFcTag	feature_id[3].value <= threshold=24427.2880859375
node_58: feature_name=HuIgG_0.03	feature_id[5].value > threshold=1711.6666870117188
Class: healthcare workers over 180 days after vaccination	
Rules_213	passed counts:1
node_0: feature_name=HuIgG_0.10	feature_id[1].value <= threshold=19120.166015625
node_1: feature_name=SARS.CoV.2.S1.mFcTag	feature_id[3].value > threshold=5354.388916015625
node_9: feature_name=a-HuIgG_0.30	feature_id[4].value > threshold=33726.833984375
node_55: feature_name=SARS.CoV.2.S1.mFcTag	feature_id[3].value <= threshold=43185.68359375
node_56: feature_name=SARS.CoV.S1.HisTag	feature_id[6].value <= threshold=1493.4166870117188
node_57: feature_name=SARS.CoV.2.S1.mFcTag	feature_id[3].value <= threshold=24427.2880859375
node_58: feature_name=HuIgG_0.03	feature_id[5].value <= threshold=1711.6666870117188
Class: healthcare workers between 60 and 180 days after vaccination	
Rules_214	passed counts:1
node_0: feature_name=HuIgG_0.10	feature_id[1].value <= threshold=19120.166015625
node_1: feature_name=SARS.CoV.2.S1.mFcTag	feature_id[3].value > threshold=5354.388916015625
node_9: feature_name=a-HuIgG_0.30	feature_id[4].value <= threshold=33726.833984375
node_10: feature_name=HuIgG_0.03	feature_id[5].value > threshold=1023.1666564941406
node_20: feature_name=hCoV.HKU1.NP	feature_id[10].value > threshold=-20.05833387374878
node_22: feature_name=SARS.CoV.S1.HisTag	feature_id[6].value > threshold=400.3000030517578

node_26: feature_name=SARS.CoV.S1.HisTag	feature_id[6].value > threshold=15087.41650390625
node_38: feature_name=hCoV.HKU1.NP	feature_id[10].value > threshold=348.6666564941406
node_46: feature_name=SARS.CoV.2.S2	feature_id[2].value > threshold=21127.650390625
node_50: feature_name=a-HuIgG_0.03	feature_id[8].value > threshold=11226.33349609375
node_52: feature_name=a-HuIgG_0.03	feature_id[8].value > threshold=11766.0
Class: healthcare workers between 60 and 180 days after vaccination	
Rules_215	passed counts:1
node_0: feature_name=HuIgG_0.10	feature_id[1].value <= threshold=19120.166015625
node_1: feature_name=SARS.CoV.2.S1.mFcTag	feature_id[3].value > threshold=5354.388916015625
node_9: feature_name=a-HuIgG_0.30	feature_id[4].value <= threshold=33726.833984375
node_10: feature_name=HuIgG_0.03	feature_id[5].value > threshold=1023.1666564941406
node_20: feature_name=hCoV.HKU1.NP	feature_id[10].value > threshold=-20.05833387374878
node_22: feature_name=SARS.CoV.S1.HisTag	feature_id[6].value > threshold=400.3000030517578
node_26: feature_name=SARS.CoV.S1.HisTag	feature_id[6].value > threshold=15087.41650390625
node_38: feature_name=hCoV.HKU1.NP	feature_id[10].value > threshold=348.6666564941406
node_46: feature_name=SARS.CoV.2.S2	feature_id[2].value > threshold=21127.650390625
node_50: feature_name=a-HuIgG_0.03	feature_id[8].value > threshold=11226.33349609375
node_52: feature_name=a-HuIgG_0.03	feature_id[8].value <= threshold=11766.0
Class: healthcare workers within 60 days after vaccination	
Rules_216	passed counts:1
node_0: feature_name=HuIgG_0.10	feature_id[1].value <= threshold=19120.166015625
node_1: feature_name=SARS.CoV.2.S1.mFcTag	feature_id[3].value > threshold=5354.388916015625
node_9: feature_name=a-HuIgG_0.30	feature_id[4].value <= threshold=33726.833984375

node_10: feature_name=HuIgG_0.03	feature_id[5].value > threshold=1023.1666564941406
node_20: feature_name=hCoV.HKU1.NP	feature_id[10].value > threshold=-20.05833387374878
node_22: feature_name=SARS.CoV.S1.HisTag	feature_id[6].value > threshold=400.3000030517578
node_26: feature_name=SARS.CoV.S1.HisTag	feature_id[6].value > threshold=15087.41650390625
node_38: feature_name=hCoV.HKU1.NP	feature_id[10].value > threshold=348.6666564941406
node_46: feature_name=SARS.CoV.2.S2	feature_id[2].value <= threshold=21127.650390625
node_47: feature_name=SARS.CoV.2.Spike.RBD.rFc	feature_id[9].value <= threshold=38684.56640625
Class: healthcare workers between 60 and 180 days after vaccination	
Rules_217	passed counts:1
node_0: feature_name=HuIgG_0.10	feature_id[1].value <= threshold=19120.166015625
node_1: feature_name=SARS.CoV.2.S1.mFcTag	feature_id[3].value > threshold=5354.388916015625
node_9: feature_name=a-HuIgG_0.30	feature_id[4].value <= threshold=33726.833984375
node_10: feature_name=HuIgG_0.03	feature_id[5].value > threshold=1023.1666564941406
node_20: feature_name=hCoV.HKU1.NP	feature_id[10].value > threshold=-20.05833387374878
node_22: feature_name=SARS.CoV.S1.HisTag	feature_id[6].value > threshold=400.3000030517578
node_26: feature_name=SARS.CoV.S1.HisTag	feature_id[6].value > threshold=15087.41650390625
node_38: feature_name=hCoV.HKU1.NP	feature_id[10].value <= threshold=348.6666564941406
node_39: feature_name=hCoV.NL63.S1_S2	feature_id[7].value <= threshold=26557.333984375
node_40: feature_name=SARS.CoV.S1.HisTag	feature_id[6].value > threshold=25028.0
node_42: feature_name=a-HuIgG_0.03	feature_id[8].value > threshold=7501.16650390625
Class: healthcare workers within 60 days after vaccination	
Rules_218	passed counts:1
node_0: feature_name=HuIgG_0.10	feature_id[1].value <= threshold=19120.166015625

node_1: feature_name=SARS.CoV.2.S1.mFcTag	feature_id[3].value > threshold=5354.388916015625
node_9: feature_name=a-HuIgG_0.30	feature_id[4].value <= threshold=33726.833984375
node_10: feature_name=HuIgG_0.03	feature_id[5].value > threshold=1023.1666564941406
node_20: feature_name=hCoV.HKU1.NP	feature_id[10].value > threshold=-20.05833387374878
node_22: feature_name=SARS.CoV.S1.HisTag	feature_id[6].value > threshold=400.3000030517578
node_26: feature_name=SARS.CoV.S1.HisTag	feature_id[6].value > threshold=15087.41650390625
node_38: feature_name=hCoV.HKU1.NP	feature_id[10].value <= threshold=348.6666564941406
node_39: feature_name=hCoV.NL63.S1_S2	feature_id[7].value <= threshold=26557.333984375
node_40: feature_name=SARS.CoV.S1.HisTag	feature_id[6].value > threshold=25028.0
node_42: feature_name=a-HuIgG_0.03	feature_id[8].value <= threshold=7501.16650390625
Class: healthcare workers between 60 and 180 days after vaccination	
Rules_219	passed counts:1
node_0: feature_name=HuIgG_0.10	feature_id[1].value <= threshold=19120.166015625
node_1: feature_name=SARS.CoV.2.S1.mFcTag	feature_id[3].value > threshold=5354.388916015625
node_9: feature_name=a-HuIgG_0.30	feature_id[4].value <= threshold=33726.833984375
node_10: feature_name=HuIgG_0.03	feature_id[5].value > threshold=1023.1666564941406
node_20: feature_name=hCoV.HKU1.NP	feature_id[10].value > threshold=-20.05833387374878
node_22: feature_name=SARS.CoV.S1.HisTag	feature_id[6].value > threshold=400.3000030517578
node_26: feature_name=SARS.CoV.S1.HisTag	feature_id[6].value <= threshold=15087.41650390625
node_27: feature_name=SARS.CoV.2.S1.RBD.mFc	feature_id[0].value > threshold=43991.083984375
node_35: feature_name=SARS.CoV.S1.HisTag	feature_id[6].value <= threshold=5744.75
Class: healthcare workers over 180 days after vaccination	
Rules_220	passed counts:1

node_0: feature_name=HuIgG_0.10	feature_id[1].value <= threshold=19120.166015625
node_1: feature_name=SARS.CoV.2.S1.mFcTag	feature_id[3].value > threshold=5354.388916015625
node_9: feature_name=a-HuIgG_0.30	feature_id[4].value <= threshold=33726.833984375
node_10: feature_name=HuIgG_0.03	feature_id[5].value > threshold=1023.1666564941406
node_20: feature_name=hCoV.HKU1.NP	feature_id[10].value > threshold=-20.05833387374878
node_22: feature_name=SARS.CoV.S1.HisTag	feature_id[6].value > threshold=400.3000030517578
node_26: feature_name=SARS.CoV.S1.HisTag	feature_id[6].value <= threshold=15087.41650390625
node_27: feature_name=SARS.CoV.2.S1.RBD.mFc	feature_id[0].value <= threshold=43991.083984375
node_28: feature_name=SARS.CoV.2.S1.RBD.mFc	feature_id[0].value > threshold=33656.484375
node_30: feature_name=SARS.CoV.2.S1+S2	feature_id[12].value <= threshold=30126.75
node_31: feature_name=SARS.CoV.S1.HisTag	feature_id[6].value <= threshold=3654.1666259765625
Class: healthcare workers between 60 and 180 days after vaccination	
Rules_221	passed counts:1
node_0: feature_name=HuIgG_0.10	feature_id[1].value <= threshold=19120.166015625
node_1: feature_name=SARS.CoV.2.S1.mFcTag	feature_id[3].value > threshold=5354.388916015625
node_9: feature_name=a-HuIgG_0.30	feature_id[4].value <= threshold=33726.833984375
node_10: feature_name=HuIgG_0.03	feature_id[5].value > threshold=1023.1666564941406
node_20: feature_name=hCoV.HKU1.NP	feature_id[10].value > threshold=-20.05833387374878
node_22: feature_name=SARS.CoV.S1.HisTag	feature_id[6].value <= threshold=400.3000030517578
node_23: feature_name=hCoV.NL63.S1_S2	feature_id[7].value <= threshold=25709.0419921875
Class: healthcare workers between 60 and 180 days after vaccination	
Rules_222	passed counts:1
node_0: feature_name=HuIgG_0.10	feature_id[1].value <= threshold=19120.166015625

node_1: feature_name=SARS.CoV.2.S1.mFcTag	feature_id[3].value > threshold=5354.388916015625
node_9: feature_name=a-HuIgG_0.30	feature_id[4].value <= threshold=33726.833984375
node_10: feature_name=HuIgG_0.03	feature_id[5].value <= threshold=1023.1666564941406
node_11: feature_name=SARS.CoV.2.S1.mFcTag	feature_id[3].value <= threshold=34194.916015625
node_12: feature_name=hCoV.NL63.S1_S2	feature_id[7].value > threshold=23953.708984375
node_14: feature_name=HuIgG_0.03	feature_id[5].value > threshold=786.3333435058594
Class: healthcare workers over 180 days after vaccination	
Rules_223	passed counts:1
node_0: feature_name=HuIgG_0.10	feature_id[1].value <= threshold=19120.166015625
node_1: feature_name=SARS.CoV.2.S1.mFcTag	feature_id[3].value > threshold=5354.388916015625
node_9: feature_name=a-HuIgG_0.30	feature_id[4].value <= threshold=33726.833984375
node_10: feature_name=HuIgG_0.03	feature_id[5].value <= threshold=1023.1666564941406
node_11: feature_name=SARS.CoV.2.S1.mFcTag	feature_id[3].value <= threshold=34194.916015625
node_12: feature_name=hCoV.NL63.S1_S2	feature_id[7].value > threshold=23953.708984375
node_14: feature_name=HuIgG_0.03	feature_id[5].value <= threshold=786.3333435058594
Class: healthcare workers between 60 and 180 days after vaccination	
Rules_224	passed counts:1
node_0: feature_name=HuIgG_0.10	feature_id[1].value <= threshold=19120.166015625
node_1: feature_name=SARS.CoV.2.S1.mFcTag	feature_id[3].value <= threshold=5354.388916015625
node_2: feature_name=SARS.CoV.S1.HisTag	feature_id[6].value > threshold=-423.2833251953125
node_4: feature_name=SARS.CoV.2.S2	feature_id[2].value <= threshold=566.3889007568359
node_5: feature_name=SARS.CoV.2.S2	feature_id[2].value > threshold=462.55555725097656
Class: healthcare workers within 60 days after vaccination	

Rules_225	passed counts:1
node_0: feature_name=HuIgG_0.10	feature_id[1].value <= threshold=19120.166015625
node_1: feature_name=SARS.CoV.2.S1.mFcTag	feature_id[3].value <= threshold=5354.388916015625
node_2: feature_name=SARS.CoV.S1.HisTag	feature_id[6].value <= threshold=-423.2833251953125
Class: healthcare workers between 60 and 180 days after vaccination	
