# Supplementary material for: Machine Learning Classification of Time since BNT162b2 COVID-19 Vaccination Based on Array-Measured Antibody Activity
Source: Life (Basel). 2023 May 31;13(6):1304. doi: 10.3390/life13061304 (PMC10305362; doi:10.3390/life13061304)
Supplement: Supplementary file 1 [file life-13-01304-s001.zip › Table S1.pdf]

Table S1. Feature names and their descriptions

Antigen ID	Virus	Antigen
SARS.CoV.2.NP	SARS-CoV-2	SARS-CoV-2_NP
SARS.CoV.2.S1+S2	SARS-CoV-2	2019-nCoV Spike Protein(S1+S2 ECD, His tag)
SARS.CoV.2.S2	SARS-CoV-2	2019-nCoV Spike Protein (S2 ECD, His tag)
SARS.CoV.2.S1.mFcTag	SARS-CoV-2	2019-nCoV Spike Protein S1 (mFc Tag)
SARS.CoV.2.S1	SARS-CoV-2	SARS-CoV-2_S1(His)
SARS.CoV.2.S1.HisTag	SARS-CoV-2	2019-nCoV Spike Protein S1 (His Tag)
SARS.CoV.2.S1.RBD.mFc	SARS-CoV-2	SARS-CoV-2 (2019-nCoV) Spike RBD-mFc Recombinant Protein (HPLC-verified)
SARS.CoV.2.Spike.RBD.His.Bac	SARS-CoV-2	SARS-CoV-2 (2019-nCoV) Spike RBD-His Recombinant Protein, Baculovirus-Insect Cells
SARS.CoV.2.Spike.RBD.His.HEK	SARS-CoV-2	SARS-CoV-2 (2019-nCoV) Spike RBD-His Recombinant Protein, HEK293
SARS.CoV.2.Spike.RBD.rFc	SARS-CoV-2	SARS-CoV-2 (2019-nCoV) Spike RBD-rFc Recombinant Protein
MERS.CoV.NP	MERS	MERS-CoV (NCoV / Novel coronavirus) Nucleoprotein / NP protein (His Tag)
MERS.CoV.S1.ECD.1-1297.HisTag	MERS	MERS-CoV (NCoV / Novel coronavirus) Spike Protein (ECD, aa 1-1297, His Tag)
MERS.CoV.S1.RBD.367.606.rFcTag	MERS	MERS-CoV_S1-RBD,N-(AA367-606,rFcTag)
SARS.CoV.NP	SARS	SARS-CoV_NP(His)
SARS.CoV.S1.HisTag	SARS	SARS-CoV_S1,(HisTag)
SARS.CoV.S1.RBD.HisTag	SARS	SARS-CoV_S1-RBD,(HisTag)
SARS.CoV.S1.RBD.rFcTag	SARS	SARS-CoV_S1-RBD,rFcTag
hCoV.NL63.S1	Common Cold CoV	HCoV-NL63_S1
hCoV.229E.S1	Common Cold CoV	HCoV-229E_S1
hCoV.OC43.HE	Common Cold CoV	HCoV-OC43_HE
hCoV.NL63.S1_S2	Common Cold CoV	HCoV-NL63_S1+S2
hCoV.229E.S1_S2	Common Cold CoV	HCoV-229E_S1+S2
hCoV.HKU1.S1_S2	Common Cold CoV	HCoV-HKU1_S1+S2
hCoV.OC43.S1_S2ECD.HisTag	Common Cold CoV	HCoV-OC43_S1+S2 ECD, (His Tag)
hCoV.NL63.NP	Common Cold CoV	HCoV-NL63_NP, (His Tag)
hCoV.HKU1.NP	Common Cold CoV	HCoV-HKU1_NP, (His Tag)
hCoV.OC43.NP	Common Cold CoV	HCoV-OC43_Hemagglutinin esterase Protein (His Tag)

DcCoV.HKU23.NP	Common Cold CoV	DcCoV_HKU23-368F_NP
hCoV.HKU1.S1_AA1.760	Common Cold CoV	HCoV-HKU1_S1,N-(AA1-760)
Flu.B_Mal/.HA1+HA2	Influenza	B_B/Malaysia/2506/2004_HA1+HA2
Flu.B_Mal/.HA1	Influenza	B_B/Malaysia/2506/2004_HA1
Flu.H1N1.HA1+HA2	Influenza	H1N1_A/Beijing/22808/2009_HA1+HA2
Flu.H1N1.HA1	Influenza	H1N1_A/Beijing/22808/2009_HA1
Flu.H3N2.HA1+HA2	Influenza	H3N2_A/Texas/50/2012_HA1+HA2
Flu.H3N2.HA1	Influenza	H3N2_A/Texas/50/2012_HA1
Flu.B_Phu/.HA1+HA2	Influenza	B_B/Phuket/3073/2013_HA1+HA2
Flu.B_Phu/.HA1	Influenza	B_B/Phuket/3073/2013_HA1
a-HuIgA_0.30	non-viral immunoglobulin	Anti-Human Immunoglobulin A with a concentration of 0.30 µg/µL
a-HuIgA_0.10	non-viral immunoglobulin	Anti-Human Immunoglobulin A with a concentration of 0.10 µg/µL
a-HuIgA_0.03	non-viral immunoglobulin	Anti-Human Immunoglobulin A with a concentration of 0.03 µg/µL
a-HuIgG_0.30	non-viral immunoglobulin	Anti-Human Immunoglobulin G with a concentration of 0.30 µg/µL
a-HuIgG_0.10	non-viral immunoglobulin	Anti-Human Immunoglobulin G with a concentration of 0.10 µg/µL
a-HuIgG_0.03	non-viral immunoglobulin	Anti-Human Immunoglobulin G with a concentration of 0.03 µg/µL
a-HuIgM_0.30	non-viral immunoglobulin	Anti-Human Immunoglobulin M with a concentration of 0.30 µg/µL
a-HuIgM_0.10	non-viral immunoglobulin	Anti-Human Immunoglobulin M with a concentration of 0.10 µg/µL
a-HuIgM_0.03	non-viral immunoglobulin	Anti-Human Immunoglobulin M with a concentration of 0.03 µg/µL
a-MoIgA_0.30	non-viral immunoglobulin	Anti-Mouse Immunoglobulin A with a concentration of 0.30 µg/µL
a-MoIgA_0.10	non-viral immunoglobulin	Anti-Mouse Immunoglobulin A with a concentration of 0.10 µg/µL
a-MoIgA_0.03	non-viral immunoglobulin	Anti-Mouse Immunoglobulin A with a concentration of 0.03 µg/µL
a-MoIgG_0.30	non-viral immunoglobulin	Anti-Mouse Immunoglobulin G with a concentration of 0.30 µg/µL
a-MoIgG_0.10	non-viral immunoglobulin	Anti-Mouse Immunoglobulin G with a concentration of 0.10 µg/µL
a-MoIgG_0.03	non-viral immunoglobulin	Anti-Mouse Immunoglobulin G with a concentration of 0.03 µg/µL
a-MoIgM_0.30	non-viral immunoglobulin	Anti-Mouse Immunoglobulin M with a concentration of 0.30 µg/µL
a-MoIgM_0.10	non-viral immunoglobulin	Anti-Mouse Immunoglobulin M with a concentration of 0.10 µg/µL
a-MoIgM_0.03	non-viral immunoglobulin	Anti-Mouse Immunoglobulin M with a concentration of 0.03 µg/µL
HuIgA_0.30	non-viral immunoglobulin	Human Immunoglobulin A with a concentration of 0.30 µg/µL
HuIgA_0.10	non-viral immunoglobulin	Human Immunoglobulin A with a concentration of 0.10 µg/µL

HuIgA_0.03	non-viral immunoglobulin	Human Immunoglobulin A with a concentration of 0.03 μg/μL
HuIgG_0.30	non-viral immunoglobulin	Human Immunoglobulin G with a concentration of 0.30 μg/μL
HuIgG_0.10	non-viral immunoglobulin	Human Immunoglobulin G with a concentration of 0.10 μg/μL
HuIgG_0.03	non-viral immunoglobulin	Human Immunoglobulin G with a concentration of 0.03 μg/μL
HuIgM_0.30	non-viral immunoglobulin	Human Immunoglobulin M with a concentration of 0.30 μg/μL
HuIgM_0.10	non-viral immunoglobulin	Human Immunoglobulin M with a concentration of 0.10 μg/μL
HuIgM_0.03	non-viral immunoglobulin	Human Immunoglobulin M with a concentration of 0.03 μg/μL
MoIgA_0.30	non-viral immunoglobulin	Mouse Immunoglobulin A with a concentration of 0.30 μg/μL
MoIgA_0.10	non-viral immunoglobulin	Mouse Immunoglobulin A with a concentration of 0.10 μg/μL
MoIgA_0.03	non-viral immunoglobulin	Mouse Immunoglobulin A with a concentration of 0.03 μg/μL
MoIgG_0.30	non-viral immunoglobulin	Mouse Immunoglobulin G with a concentration of 0.30 μg/μL
MoIgG_0.10	non-viral immunoglobulin	Mouse Immunoglobulin G with a concentration of 0.10 μg/μL
MoIgG_0.03	non-viral immunoglobulin	Mouse Immunoglobulin G with a concentration of 0.03 μg/μL
MoIgM_0.30	non-viral immunoglobulin	Mouse Immunoglobulin M with a concentration of 0.30 μg/μL
MoIgM_0.10	non-viral immunoglobulin	Mouse Immunoglobulin M with a concentration of 0.10 μg/μL
MoIgM_0.03	non-viral immunoglobulin	Mouse Immunoglobulin M with a concentration of 0.03 μg/μL
