# Supplementary material for: Machine Learning Classification of Time since BNT162b2 COVID-19 Vaccination Based on Array-Measured Antibody Activity
Source: Life (Basel). 2023 May 31;13(6):1304. doi: 10.3390/life13061304 (PMC10305362; doi:10.3390/life13061304)
Supplement: Supplementary file 1 [file life-13-01304-s001.zip › Table S2.pdf]

Table S2. Feature lists obtained by LASSO, LightGBM, MCFS, and mRMR methods.

Rank	LASSO feature list	LightGBM feature list	MCFS feature list	mRMR feature list
1	SARS.CoV.2.S1.mFcTag	HuIgG_0.03	SARS.CoV.2.S1.mFcTag	SARS.CoV.2.S1.RBD.mFc
2	a-HuIgG_0.03	SARS.CoV.2.Spike.RBD.His.Bac	SARS.CoV.2.S1.RBD.mFc	HuIgG_0.10
3	hCoV.229E.S1	HuIgG_0.10	SARS.CoV.2.S1.HisTag	SARS.CoV.2.S2
4	MERS.CoV.S1.RBD.367.606.rFcTag	a-HuIgG_0.10	SARS.CoV.2.Spike.RBD.His.Bac	SARS.CoV.2.S1.mFcTag
5	Flu.B_Mal/.HA1	a-HuIgG_0.30	SARS.CoV.2.Spike.RBD.His.HEK	a-HuIgG_0.30
6	hCoV.NL63.S1	MERS.CoV.S1.RBD.367.606.rFcTag	SARS.CoV.2.Spike.RBD.rFc	HuIgG_0.03
7	Flu.H1N1.HA1+HA2	HuIgM_0.10	SARS.CoV.2.S1	SARS.CoV.S1.HisTag
8	SARS.CoV.2.Spike.RBD.rFc	a-HuIgG_0.03	SARS.CoV.2.S1+S2	hCoV.NL63.S1_S2
9	hCoV.HKU1.S1_AA1.760	HuIgM_0.30	HuIgG_0.03	a-HuIgG_0.03
10	SARS.CoV.2.Spike.RBD.His.Bac	Flu.B_Mal/.HA1	HuIgG_0.10	SARS.CoV.2.Spike.RBD.rFc
11	hCoV.OC43.HE	Flu.H1N1.HA1+HA2	SARS.CoV.S1.RBD.HisTag	hCoV.HKU1.NP
12	Flu.B_Phu/.HA1	hCoV.HKU1.NP	a-HuIgG_0.30	Flu.B_Phu/.HA1+HA2

13	MERS.CoV.S1.ECD.1-1297.HisTag	a-MoIgG_0.03	HuIgG_0.30	SARS.CoV.2.S1+S2
14	HuIgM_0.30	hCoV.NL63.S1_S2	a-HuIgG_0.10	MoIgA_0.10
15	SARS.CoV.S1.HisTag	HuIgM_0.03	a-HuIgG_0.03	HuIgG_0.30
16	SARS.CoV.2.S1	MERS.CoV.NP	SARS.CoV.S1.HisTag	SARS.CoV.2.Spike.RBD.His.Bac
17	SARS.CoV.2.S1.HisTag	SARS.CoV.S1.RBD.rFcTag	a-MoIgG_0.10	HuIgA_0.30
18	SARS.CoV.2.S1.RBD.mFc	SARS.CoV.2.S1+S2	HuIgM_0.10	a-MoIgG_0.30
19	SARS.CoV.2.S1+S2	SARS.CoV.S1.HisTag	Flu.B_Phu/.HA1+HA2	Flu.B_Mal/.HA1
20	SARS.CoV.NP	Flu.B_Phu/.HA1+HA2	hCoV.HKU1.NP	SARS.CoV.S1.RBD.HisTag
21	SARS.CoV.S1.RBD.HisTag	MoIgG_0.03	SARS.CoV.2.S2	MERS.CoV.S1.RBD.367.606.rFcTag
22	DcCoV.HKU23.NP	SARS.CoV.2.S1.mFcTag	HuIgM_0.03	Flu.H3N2.HA1+HA2
23	hCoV.HKU1.S1_S2	Flu.B_Mal/.HA1+HA2	a-MoIgA_0.30	SARS.CoV.2.S1.HisTag
24	hCoV.NL63.NP	hCoV.OC43.NP	Flu.B_Mal/.HA1	HuIgM_0.30
25	hCoV.OC43.S1_S2ECD.HisTag	a-MoIgA_0.03	hCoV.NL63.S1_S2	a-HuIgG_0.10
26	Flu.B_Mal/.HA1+HA2	Flu.H3N2.HA1	MoIgG_0.03	MoIgG_0.03

27	Flu.H1N1.HA1	HuIgA_0.03	Flu.H1N1.HA1	SARS.CoV.2.S1
28	a-HuIgA_0.30	a-MoIgG_0.10	a-MoIgG_0.30	a-HuIgM_0.03
29	a-HuIgA_0.10	SARS.CoV.2.S1.RBD.mFc	Flu.H3N2.HA1+HA2	a-MoIgG_0.03
30	a-HuIgA_0.03	SARS.CoV.2.S2	MERS.CoV.NP	SARS.CoV.2.Spike.RBD.His.HEK
31	a-HuIgG_0.30	hCoV.229E.S1	SARS.CoV.S1.RBD.rFcTag	HuIgM_0.03
32	a-HuIgG_0.10	SARS.CoV.NP	Flu.H1N1.HA1+HA2	MERS.CoV.S1.ECD.1-1297.HisTag
33	a-HuIgM_0.30	hCoV.HKU1.S1_S2	Flu.B_Mal/.HA1+HA2	HuIgM_0.10
34	a-HuIgM_0.10	hCoV.OC43.HE	HuIgA_0.03	hCoV.NL63.NP
35	a-HuIgM_0.03	MoIgG_0.30	HuIgM_0.30	HuIgA_0.03
36	a-MoIgA_0.30	hCoV.229E.S1_S2	a-MoIgA_0.03	hCoV.HKU1.S1_AA1.760
37	a-MoIgA_0.10	HuIgG_0.30	MERS.CoV.S1.RBD.367.606.rFcTag	Flu.H1N1.HA1+HA2
38	a-MoIgA_0.03	a-MoIgA_0.30	a-MoIgG_0.03	a-MoIgA_0.03
39	a-MoIgG_0.10	Flu.H3N2.HA1+HA2	hCoV.229E.S1	Flu.H3N2.HA1
40	a-MoIgM_0.30	hCoV.HKU1.S1_AA1.760	a-HuIgA_0.03	a-MoIgA_0.10

41	a-MoIgM_0.10	a-HuIgM_0.03	hCoV.HKU1.S1_S2	a-MoIgM_0.03
42	a-MoIgM_0.03	HuIgA_0.30	hCoV.HKU1.S1_AA1.760	HuIgA_0.10
43	HuIgA_0.10	Flu.H1N1.HA1	a-MoIgA_0.10	hCoV.OC43.NP
44	HuIgA_0.03	hCoV.NL63.S1	Flu.H3N2.HA1	SARS.CoV.S1.RBD.rFcTag
45	HuIgG_0.03	a-HuIgA_0.03	hCoV.OC43.S1_S2ECD.HisTag	hCoV.OC43.HE
46	HuIgM_0.03	hCoV.OC43.S1_S2ECD.HisTag	SARS.CoV.NP	MoIgM_0.03 - 0.00221
47	MoIgA_0.30	a-MoIgA_0.10	MERS.CoV.S1.ECD.1-1297.HisTag	hCoV.HKU1.S1_S2
48	MoIgA_0.03	MERS.CoV.S1.ECD.1-1297.HisTag	a-MoIgM_0.30	SARS.CoV.2.NP
49	MoIgG_0.30	SARS.CoV.2.NP	Flu.B_Phu/.HA1	a-HuIgA_0.03
50	MoIgG_0.10	DcCoV.HKU23.NP	SARS.CoV.2.NP	Flu.B_Mal/.HA1+HA2
51	MoIgM_0.30	SARS.CoV.2.S1	hCoV.NL63.NP	MERS.CoV.NP
52	MoIgM_0.10	MoIgA_0.03	hCoV.OC43.HE	hCoV.229E.S1
53	MoIgM_0.03	HuIgA_0.10	MoIgG_0.30	a-MoIgG_0.10
54	MoIgG_0.03	MoIgA_0.10	hCoV.OC43.NP	MoIgA_0.03

55	hCoV.OC43.NP	Flu.B_Phu/.HA1	HuIgA_0.30	MoIgG_0.10
56	MoIgA_0.10	hCoV.NL63.NP	hCoV.NL63.S1	a-MoIgA_0.30
57	HuIgA_0.30	SARS.CoV.2.Spike.RBD.rFc	a-MoIgM_0.03	hCoV.OC43.S1_S2 ECD.HisTag
58	HuIgM_0.10	SARS.CoV.2.S1.HisTag	a-MoIgM_0.10	MoIgM_0.10
59	a-MoIgG_0.30	MoIgG_0.10	hCoV.229E.S1_S2	MoIgG_0.30
60	SARS.CoV.2.NP	a-MoIgM_0.03	MoIgG_0.10	a-HuIgM_0.10
61	Flu.H3N2.HA1	MoIgM_0.10	a-HuIgM_0.03	DcCoV.HKU23.NP
62	MERS.CoV.NP	SARS.CoV.2.Spike.RBD.His.HEK	a-HuIgM_0.30	hCoV.NL63.S1
63	HuIgG_0.30	MoIgM_0.30	HuIgA_0.10	SARS.CoV.NP
64	hCoV.HKU1.NP	MoIgM_0.03	MoIgM_0.03	MoIgA_0.30
65	a-MoIgG_0.03	SARS.CoV.S1.RBD.HisTag	MoIgA_0.03	a-MoIgM_0.10
66	SARS.CoV.2.Spike.RBD.His.HEK	MoIgA_0.30	DcCoV.HKU23.NP	a-HuIgA_0.10
67	SARS.CoV.2.S2	a-HuIgM_0.10	a-HuIgA_0.30	Flu.H1N1.HA1
68	SARS.CoV.S1.RBD.rFcTag	a-HuIgA_0.10	MoIgM_0.10	MoIgM_0.30

69	Flu.H3N2.HA1+HA2	a-HuIgA_0.30	MoIgM_0.30	Flu.B_Phu/.HA1
70	Flu.B_Phu/.HA1+HA2	a-MoIgG_0.30	a-HuIgA_0.10	a-HuIgM_0.30
71	hCoV.NL63.S1_S2	a-MoIgM_0.30	a-HuIgM_0.10	hCoV.229E.S1_S2
72	hCoV.229E.S1_S2	a-HuIgM_0.30	MoIgA_0.10	a-HuIgA_0.30
73	HuIgG_0.10	a-MoIgM_0.10	MoIgA_0.30	a-MoIgM_0.30
