# Supplementary material for: Machine Learning Classification of Time since BNT162b2 COVID-19 Vaccination Based on Array-Measured Antibody Activity
Source: Life (Basel). 2023 May 31;13(6):1304. doi: 10.3390/life13061304 (PMC10305362; doi:10.3390/life13061304)
Supplement: Supplementary file 1 [file life-13-01304-s001.zip › Table S3.pdf]

Table S3. IFS results with different classification algorithms on four feature lists.

(1) IFS results on the LASSO feature list

Classification algorithms	Number of features	Unvaccinated healthcare workers	Healthcare workers within 60 days after vaccination	Healthcare workers between 60 and180 days after vaccination	Healthcare workers over 180 days after vaccination	ACC	MCC	Macro F1	Weighted F1
DT	1	0.777	0.537	0.521	0.328	0.519	0.310	0.541	0.527
DT	2	0.847	0.617	0.591	0.505	0.610	0.426	0.640	0.612
DT	3	0.797	0.578	0.581	0.554	0.595	0.406	0.628	0.593
DT	4	0.865	0.601	0.595	0.552	0.613	0.430	0.653	0.613
DT	5	0.853	0.606	0.587	0.644	0.623	0.438	0.673	0.620
DT	6	0.838	0.616	0.606	0.648	0.635	0.457	0.677	0.632
DT	7	0.830	0.600	0.579	0.632	0.615	0.423	0.660	0.612
DT	8	0.884	0.597	0.601	0.630	0.626	0.440	0.678	0.624
DT	9	0.900	0.647	0.637	0.646	0.663	0.493	0.707	0.662
DT	10	0.855	0.611	0.625	0.641	0.640	0.458	0.683	0.639
DT	11	0.844	0.634	0.642	0.665	0.659	0.493	0.696	0.656
DT	12	0.864	0.611	0.631	0.627	0.643	0.468	0.683	0.641
DT	13	0.903	0.616	0.646	0.655	0.657	0.484	0.705	0.655
DT	14	0.847	0.609	0.633	0.652	0.645	0.470	0.685	0.642
DT	15	0.895	0.636	0.649	0.692	0.670	0.504	0.718	0.667
DT	16	0.876	0.635	0.648	0.680	0.666	0.499	0.710	0.664
DT	17	0.885	0.664	0.657	0.700	0.682	0.518	0.726	0.681
DT	18	0.832	0.637	0.634	0.643	0.653	0.478	0.687	0.651

DT	19	0.899	0.657	0.644	0.623	0.666	0.500	0.706	0.666
DT	20	0.909	0.640	0.641	0.677	0.666	0.497	0.717	0.665
DT	21	0.898	0.647	0.631	0.699	0.668	0.502	0.719	0.665
DT	22	0.871	0.664	0.668	0.646	0.681	0.526	0.712	0.680
DT	23	0.912	0.640	0.628	0.637	0.656	0.481	0.704	0.655
DT	24	0.867	0.640	0.638	0.657	0.660	0.490	0.700	0.658
DT	25	0.888	0.648	0.626	0.665	0.661	0.491	0.707	0.658
DT	26	0.889	0.668	0.651	0.728	0.686	0.529	0.734	0.684
DT	27	0.888	0.657	0.647	0.669	0.673	0.508	0.715	0.671
DT	28	0.906	0.658	0.656	0.683	0.680	0.517	0.726	0.678
DT	29	0.833	0.642	0.660	0.696	0.672	0.510	0.708	0.670
DT	30	0.912	0.628	0.635	0.649	0.656	0.481	0.706	0.655
DT	31	0.878	0.651	0.655	0.663	0.672	0.506	0.712	0.671
DT	32	0.874	0.638	0.656	0.671	0.669	0.499	0.710	0.667
DT	33	0.898	0.676	0.663	0.674	0.688	0.532	0.728	0.687
DT	34	0.888	0.661	0.641	0.644	0.669	0.500	0.709	0.668
DT	35	0.883	0.657	0.654	0.678	0.677	0.515	0.718	0.675
DT	36	0.886	0.663	0.672	0.638	0.682	0.520	0.715	0.681
DT	37	0.904	0.643	0.653	0.696	0.674	0.509	0.724	0.673
DT	38	0.904	0.662	0.653	0.633	0.674	0.508	0.713	0.673
DT	39	0.868	0.668	0.662	0.665	0.682	0.520	0.716	0.680
DT	40	0.887	0.651	0.651	0.689	0.674	0.506	0.720	0.673
DT	41	0.857	0.676	0.664	0.673	0.685	0.527	0.717	0.684
DT	42	0.876	0.653	0.669	0.700	0.684	0.524	0.725	0.682
DT	43	0.895	0.657	0.647	0.698	0.677	0.512	0.724	0.675

DT	44	0.878	0.647	0.636	0.690	0.666	0.498	0.713	0.664
DT	45	0.878	0.656	0.644	0.694	0.674	0.511	0.718	0.672
DT	46	0.852	0.658	0.671	0.720	0.687	0.528	0.725	0.685
DT	47	0.883	0.689	0.677	0.730	0.704	0.554	0.744	0.702
DT	48	0.862	0.655	0.655	0.744	0.682	0.520	0.729	0.680
DT	49	0.870	0.664	0.668	0.749	0.693	0.536	0.738	0.690
DT	50	0.836	0.681	0.676	0.745	0.699	0.545	0.734	0.697
DT	51	0.880	0.668	0.660	0.729	0.689	0.529	0.734	0.687
DT	52	0.884	0.653	0.642	0.743	0.679	0.517	0.731	0.675
DT	53	0.870	0.657	0.659	0.708	0.682	0.519	0.723	0.679
DT	54	0.870	0.669	0.645	0.708	0.680	0.518	0.723	0.678
DT	55	0.882	0.650	0.646	0.706	0.674	0.508	0.721	0.671
DT	56	0.861	0.667	0.665	0.751	0.692	0.535	0.736	0.689
DT	57	0.884	0.649	0.648	0.716	0.676	0.512	0.724	0.673
DT	58	0.869	0.671	0.652	0.756	0.689	0.529	0.737	0.686
DT	59	0.884	0.667	0.655	0.710	0.685	0.523	0.729	0.682
DT	60	0.884	0.646	0.656	0.730	0.680	0.514	0.729	0.677
DT	61	0.850	0.640	0.624	0.716	0.660	0.488	0.707	0.657
DT	62	0.868	0.646	0.655	0.738	0.680	0.518	0.727	0.676
DT	63	0.848	0.647	0.665	0.711	0.678	0.512	0.718	0.676
DT	64	0.843	0.651	0.647	0.710	0.672	0.506	0.713	0.670
DT	65	0.856	0.652	0.646	0.738	0.677	0.513	0.723	0.673
DT	66	0.850	0.655	0.662	0.707	0.680	0.518	0.718	0.678
DT	67	0.866	0.657	0.660	0.738	0.685	0.521	0.730	0.682
DT	68	0.852	0.685	0.664	0.729	0.696	0.541	0.733	0.693

DT	69	0.880	0.660	0.659	0.756	0.688	0.526	0.739	0.686
DT	70	0.866	0.659	0.673	0.773	0.696	0.539	0.743	0.692
DT	71	0.866	0.664	0.674	0.714	0.690	0.532	0.730	0.689
DT	72	0.861	0.636	0.653	0.730	0.673	0.507	0.720	0.670
DT	73	0.880	0.656	0.663	0.727	0.685	0.522	0.731	0.683
KNN	1	0.792	0.536	0.527	0.337	0.522	0.316	0.548	0.531
KNN	2	0.843	0.649	0.602	0.596	0.640	0.472	0.673	0.638
KNN	3	0.822	0.620	0.613	0.677	0.642	0.476	0.683	0.638
KNN	4	0.874	0.613	0.626	0.719	0.654	0.486	0.708	0.649
KNN	5	0.895	0.624	0.607	0.734	0.654	0.486	0.715	0.648
KNN	6	0.880	0.624	0.613	0.764	0.659	0.493	0.720	0.653
KNN	7	0.893	0.652	0.637	0.779	0.682	0.528	0.740	0.677
KNN	8	0.872	0.615	0.618	0.772	0.658	0.492	0.719	0.652
KNN	9	0.892	0.612	0.617	0.735	0.654	0.487	0.714	0.648
KNN	10	0.877	0.599	0.611	0.743	0.647	0.477	0.707	0.640
KNN	11	0.896	0.608	0.601	0.755	0.648	0.476	0.715	0.642
KNN	12	0.908	0.642	0.620	0.739	0.669	0.511	0.727	0.663
KNN	13	0.912	0.654	0.639	0.749	0.682	0.528	0.738	0.677
KNN	14	0.904	0.665	0.639	0.755	0.687	0.537	0.741	0.681
KNN	15	0.924	0.665	0.650	0.749	0.691	0.541	0.747	0.687
KNN	16	0.912	0.650	0.632	0.779	0.681	0.524	0.743	0.675
KNN	17	0.929	0.660	0.640	0.786	0.690	0.536	0.754	0.685
KNN	18	0.908	0.686	0.651	0.812	0.706	0.563	0.764	0.701
KNN	19	0.904	0.671	0.657	0.811	0.702	0.554	0.761	0.697
KNN	20	0.900	0.681	0.655	0.781	0.701	0.554	0.754	0.697

KNN	21	0.904	0.680	0.646	0.787	0.699	0.553	0.755	0.693
KNN	22	0.912	0.667	0.634	0.771	0.688	0.535	0.746	0.682
KNN	23	0.924	0.645	0.625	0.764	0.675	0.516	0.740	0.670
KNN	24	0.924	0.639	0.622	0.745	0.669	0.509	0.732	0.664
KNN	25	0.920	0.646	0.633	0.776	0.680	0.523	0.744	0.675
KNN	26	0.924	0.624	0.619	0.776	0.666	0.503	0.736	0.660
KNN	27	0.929	0.643	0.620	0.778	0.674	0.514	0.742	0.669
KNN	28	0.937	0.654	0.627	0.791	0.683	0.525	0.752	0.678
KNN	29	0.941	0.646	0.619	0.787	0.677	0.516	0.748	0.671
KNN	30	0.920	0.649	0.621	0.784	0.677	0.519	0.743	0.671
KNN	31	0.912	0.650	0.631	0.786	0.681	0.524	0.745	0.675
KNN	32	0.924	0.672	0.646	0.805	0.699	0.552	0.762	0.694
KNN	33	0.945	0.671	0.639	0.782	0.694	0.543	0.759	0.689
KNN	34	0.937	0.670	0.639	0.779	0.693	0.540	0.756	0.688
KNN	35	0.924	0.670	0.638	0.784	0.693	0.543	0.754	0.687
KNN	36	0.933	0.665	0.642	0.784	0.693	0.541	0.756	0.688
KNN	37	0.924	0.672	0.645	0.772	0.695	0.546	0.754	0.690
KNN	38	0.924	0.668	0.636	0.774	0.690	0.538	0.750	0.684
KNN	39	0.933	0.678	0.649	0.779	0.700	0.552	0.760	0.695
KNN	40	0.937	0.673	0.636	0.778	0.693	0.544	0.756	0.688
KNN	41	0.933	0.655	0.630	0.778	0.683	0.527	0.749	0.678
KNN	42	0.929	0.669	0.637	0.758	0.689	0.538	0.748	0.684
KNN	43	0.928	0.662	0.636	0.768	0.687	0.532	0.749	0.682
KNN	44	0.933	0.673	0.646	0.773	0.696	0.549	0.756	0.691
KNN	45	0.916	0.660	0.644	0.781	0.690	0.539	0.750	0.685

KNN	46	0.924	0.655	0.631	0.771	0.682	0.526	0.746	0.677
KNN	47	0.916	0.676	0.648	0.775	0.698	0.552	0.754	0.692
KNN	48	0.933	0.665	0.643	0.793	0.694	0.544	0.758	0.689
KNN	49	0.950	0.676	0.644	0.788	0.699	0.551	0.764	0.694
KNN	50	0.933	0.669	0.653	0.807	0.701	0.553	0.766	0.696
KNN	51	0.929	0.672	0.652	0.770	0.697	0.548	0.755	0.692
KNN	52	0.945	0.687	0.647	0.787	0.705	0.562	0.767	0.700
KNN	53	0.941	0.664	0.641	0.776	0.691	0.539	0.755	0.686
KNN	54	0.908	0.679	0.652	0.775	0.700	0.555	0.754	0.695
KNN	55	0.941	0.677	0.642	0.770	0.696	0.547	0.758	0.692
KNN	56	0.937	0.660	0.636	0.770	0.687	0.532	0.751	0.682
KNN	57	0.920	0.680	0.653	0.792	0.703	0.557	0.761	0.698
KNN	58	0.929	0.693	0.655	0.777	0.709	0.569	0.764	0.703
KNN	59	0.920	0.667	0.649	0.779	0.695	0.545	0.754	0.690
KNN	60	0.932	0.668	0.646	0.780	0.694	0.541	0.756	0.690
KNN	61	0.920	0.654	0.640	0.777	0.686	0.532	0.748	0.681
KNN	62	0.937	0.654	0.636	0.763	0.684	0.529	0.748	0.679
KNN	63	0.933	0.673	0.637	0.767	0.692	0.542	0.752	0.687
KNN	64	0.916	0.677	0.647	0.767	0.697	0.552	0.752	0.692
KNN	65	0.924	0.673	0.649	0.782	0.698	0.550	0.757	0.693
KNN	66	0.916	0.681	0.654	0.779	0.702	0.556	0.758	0.697
KNN	67	0.936	0.666	0.642	0.786	0.693	0.542	0.758	0.689
KNN	68	0.924	0.678	0.661	0.787	0.705	0.560	0.763	0.700
KNN	69	0.908	0.675	0.650	0.773	0.697	0.551	0.751	0.692
KNN	70	0.920	0.690	0.661	0.780	0.709	0.565	0.763	0.704

KNN	71	0.920	0.687	0.665	0.806	0.712	0.569	0.770	0.708
KNN	72	0.937	0.676	0.657	0.811	0.706	0.558	0.770	0.701
KNN	73	0.933	0.690	0.668	0.812	0.716	0.574	0.776	0.711
RF	1	0.762	0.540	0.511	0.341	0.518	0.308	0.539	0.524
RF	2	0.845	0.639	0.590	0.588	0.631	0.457	0.665	0.628
RF	3	0.845	0.657	0.610	0.618	0.650	0.486	0.682	0.647
RF	4	0.887	0.651	0.614	0.699	0.663	0.502	0.713	0.658
RF	5	0.898	0.672	0.618	0.698	0.674	0.522	0.721	0.668
RF	6	0.891	0.673	0.606	0.724	0.673	0.520	0.724	0.666
RF	7	0.899	0.678	0.606	0.713	0.674	0.524	0.724	0.667
RF	8	0.888	0.695	0.617	0.736	0.688	0.547	0.734	0.680
RF	9	0.893	0.681	0.622	0.734	0.684	0.538	0.732	0.677
RF	10	0.893	0.698	0.653	0.755	0.705	0.569	0.750	0.699
RF	11	0.897	0.695	0.626	0.736	0.692	0.550	0.738	0.685
RF	12	0.897	0.695	0.624	0.734	0.691	0.551	0.737	0.684
RF	13	0.889	0.691	0.615	0.725	0.685	0.543	0.730	0.676
RF	14	0.889	0.698	0.614	0.741	0.689	0.547	0.735	0.680
RF	15	0.908	0.713	0.630	0.727	0.701	0.568	0.745	0.694
RF	16	0.897	0.714	0.633	0.764	0.706	0.571	0.752	0.698
RF	17	0.900	0.708	0.631	0.769	0.704	0.566	0.752	0.696
RF	18	0.904	0.725	0.659	0.788	0.723	0.594	0.769	0.717
RF	19	0.920	0.735	0.663	0.781	0.729	0.604	0.775	0.722
RF	20	0.924	0.735	0.661	0.785	0.729	0.602	0.776	0.723
RF	21	0.924	0.749	0.674	0.800	0.741	0.622	0.787	0.735
RF	22	0.920	0.729	0.661	0.811	0.729	0.601	0.780	0.722

RF	23	0.916	0.741	0.667	0.810	0.736	0.614	0.783	0.729
RF	24	0.916	0.737	0.657	0.808	0.731	0.608	0.780	0.723
RF	25	0.908	0.732	0.655	0.811	0.728	0.600	0.777	0.720
RF	26	0.912	0.716	0.642	0.817	0.717	0.584	0.772	0.709
RF	27	0.916	0.724	0.644	0.800	0.720	0.589	0.771	0.712
RF	28	0.916	0.733	0.663	0.809	0.731	0.605	0.780	0.724
RF	29	0.920	0.729	0.660	0.816	0.730	0.603	0.781	0.723
RF	30	0.916	0.733	0.656	0.804	0.728	0.604	0.777	0.721
RF	31	0.912	0.740	0.660	0.808	0.733	0.612	0.780	0.725
RF	32	0.916	0.740	0.644	0.808	0.728	0.607	0.777	0.719
RF	33	0.908	0.741	0.651	0.804	0.730	0.607	0.776	0.721
RF	34	0.912	0.734	0.649	0.807	0.727	0.604	0.776	0.718
RF	35	0.912	0.744	0.655	0.815	0.734	0.615	0.781	0.725
RF	36	0.916	0.725	0.637	0.815	0.720	0.595	0.774	0.711
RF	37	0.916	0.734	0.651	0.815	0.728	0.604	0.779	0.720
RF	38	0.912	0.740	0.657	0.821	0.734	0.615	0.783	0.726
RF	39	0.912	0.740	0.650	0.818	0.731	0.610	0.780	0.722
RF	40	0.916	0.737	0.641	0.823	0.728	0.606	0.779	0.718
RF	41	0.920	0.729	0.640	0.832	0.725	0.601	0.780	0.715
RF	42	0.924	0.756	0.658	0.823	0.743	0.631	0.790	0.733
RF	43	0.920	0.733	0.631	0.816	0.722	0.599	0.775	0.711
RF	44	0.924	0.734	0.627	0.809	0.721	0.599	0.774	0.710
RF	45	0.912	0.737	0.622	0.829	0.723	0.604	0.775	0.710
RF	46	0.920	0.749	0.641	0.833	0.736	0.624	0.786	0.724
RF	47	0.908	0.732	0.627	0.833	0.722	0.600	0.775	0.710

RF	48	0.912	0.743	0.634	0.833	0.730	0.614	0.781	0.718
RF	49	0.912	0.750	0.644	0.829	0.736	0.621	0.784	0.725
RF	50	0.920	0.744	0.648	0.843	0.736	0.619	0.789	0.726
RF	51	0.916	0.744	0.635	0.838	0.731	0.615	0.783	0.720
RF	52	0.908	0.748	0.637	0.831	0.732	0.616	0.781	0.721
RF	53	0.907	0.736	0.627	0.836	0.723	0.602	0.776	0.712
RF	54	0.908	0.736	0.616	0.825	0.720	0.601	0.771	0.706
RF	55	0.912	0.740	0.622	0.831	0.724	0.606	0.776	0.711
RF	56	0.912	0.731	0.626	0.833	0.721	0.598	0.776	0.710
RF	57	0.920	0.737	0.619	0.832	0.723	0.605	0.777	0.710
RF	58	0.912	0.735	0.621	0.838	0.723	0.604	0.777	0.710
RF	59	0.912	0.736	0.634	0.828	0.725	0.606	0.778	0.715
RF	60	0.900	0.736	0.629	0.839	0.725	0.607	0.776	0.713
RF	61	0.916	0.735	0.633	0.840	0.726	0.605	0.781	0.715
RF	62	0.900	0.735	0.624	0.833	0.722	0.601	0.773	0.710
RF	63	0.904	0.736	0.612	0.838	0.720	0.602	0.773	0.706
RF	64	0.900	0.745	0.634	0.827	0.729	0.614	0.777	0.717
RF	65	0.908	0.745	0.623	0.819	0.725	0.612	0.774	0.712
RF	66	0.920	0.748	0.639	0.832	0.734	0.621	0.785	0.723
RF	67	0.912	0.746	0.631	0.829	0.730	0.616	0.780	0.717
RF	68	0.912	0.738	0.631	0.835	0.726	0.607	0.779	0.715
RF	69	0.920	0.745	0.633	0.827	0.730	0.614	0.781	0.718
RF	70	0.912	0.745	0.640	0.828	0.732	0.617	0.781	0.721
RF	71	0.920	0.739	0.631	0.833	0.727	0.607	0.781	0.716
RF	72	0.920	0.749	0.650	0.848	0.740	0.627	0.792	0.730

RF	73	0.908	0.749	0.645	0.840	0.736	0.622	0.786	0.725
SVM	1	0.872	0.676	0.517	0.326	0.588	0.405	0.598	0.586
SVM	2	0.879	0.682	0.550	0.530	0.634	0.477	0.660	0.625
SVM	3	0.826	0.682	0.533	0.526	0.625	0.468	0.642	0.612
SVM	4	0.832	0.678	0.533	0.553	0.628	0.473	0.649	0.614
SVM	5	0.855	0.672	0.562	0.615	0.642	0.489	0.676	0.632
SVM	6	0.836	0.680	0.554	0.649	0.647	0.492	0.680	0.634
SVM	7	0.826	0.694	0.574	0.651	0.659	0.512	0.686	0.648
SVM	8	0.855	0.689	0.593	0.686	0.669	0.519	0.706	0.659
SVM	9	0.852	0.663	0.600	0.715	0.663	0.505	0.707	0.655
SVM	10	0.860	0.671	0.620	0.712	0.674	0.519	0.716	0.668
SVM	11	0.870	0.659	0.632	0.738	0.677	0.520	0.725	0.672
SVM	12	0.860	0.655	0.610	0.715	0.663	0.500	0.710	0.657
SVM	13	0.870	0.654	0.616	0.745	0.669	0.505	0.721	0.663
SVM	14	0.860	0.669	0.623	0.749	0.677	0.521	0.725	0.672
SVM	15	0.860	0.669	0.645	0.765	0.688	0.534	0.734	0.683
SVM	16	0.867	0.667	0.650	0.811	0.695	0.542	0.749	0.690
SVM	17	0.878	0.685	0.660	0.806	0.706	0.558	0.757	0.701
SVM	18	0.870	0.680	0.655	0.806	0.701	0.553	0.753	0.696
SVM	19	0.893	0.680	0.660	0.821	0.706	0.559	0.763	0.702
SVM	20	0.893	0.696	0.680	0.830	0.722	0.580	0.775	0.718
SVM	21	0.889	0.686	0.670	0.820	0.712	0.566	0.766	0.708
SVM	22	0.881	0.672	0.662	0.806	0.701	0.550	0.755	0.697
SVM	23	0.924	0.680	0.667	0.820	0.711	0.562	0.773	0.707
SVM	24	0.924	0.667	0.655	0.820	0.701	0.546	0.767	0.697

SVM	25	0.908	0.654	0.636	0.807	0.686	0.526	0.752	0.682
SVM	26	0.920	0.664	0.651	0.816	0.698	0.543	0.763	0.694
SVM	27	0.916	0.673	0.661	0.815	0.705	0.554	0.767	0.701
SVM	28	0.920	0.678	0.647	0.784	0.698	0.547	0.758	0.694
SVM	29	0.920	0.670	0.636	0.796	0.692	0.537	0.756	0.687
SVM	30	0.924	0.673	0.643	0.796	0.696	0.543	0.759	0.692
SVM	31	0.920	0.679	0.655	0.810	0.704	0.554	0.766	0.700
SVM	32	0.912	0.686	0.664	0.815	0.711	0.563	0.769	0.707
SVM	33	0.920	0.678	0.655	0.826	0.706	0.555	0.770	0.702
SVM	34	0.924	0.680	0.658	0.829	0.708	0.558	0.773	0.704
SVM	35	0.924	0.679	0.657	0.832	0.708	0.558	0.773	0.704
SVM	36	0.916	0.675	0.654	0.832	0.704	0.553	0.769	0.700
SVM	37	0.916	0.676	0.653	0.832	0.704	0.553	0.769	0.700
SVM	38	0.916	0.676	0.651	0.829	0.704	0.552	0.768	0.699
SVM	39	0.920	0.683	0.667	0.838	0.714	0.567	0.777	0.710
SVM	40	0.912	0.681	0.664	0.834	0.711	0.562	0.773	0.707
SVM	41	0.912	0.681	0.666	0.838	0.712	0.565	0.774	0.708
SVM	42	0.908	0.683	0.668	0.838	0.714	0.567	0.775	0.710
SVM	43	0.916	0.686	0.668	0.835	0.715	0.569	0.777	0.711
SVM	44	0.916	0.687	0.671	0.835	0.717	0.571	0.778	0.713
SVM	45	0.920	0.691	0.669	0.834	0.717	0.573	0.778	0.713
SVM	46	0.920	0.690	0.670	0.834	0.717	0.573	0.779	0.713
SVM	47	0.916	0.702	0.679	0.826	0.725	0.585	0.781	0.721
SVM	48	0.916	0.701	0.673	0.824	0.722	0.581	0.778	0.718
SVM	49	0.916	0.698	0.673	0.825	0.721	0.580	0.778	0.717

SVM	50	0.916	0.699	0.675	0.822	0.722	0.581	0.778	0.718
SVM	51	0.916	0.701	0.676	0.829	0.723	0.582	0.780	0.719
SVM	52	0.912	0.706	0.687	0.832	0.730	0.591	0.784	0.726
SVM	53	0.912	0.709	0.685	0.821	0.729	0.591	0.782	0.725
SVM	54	0.912	0.709	0.685	0.821	0.729	0.591	0.782	0.725
SVM	55	0.916	0.711	0.683	0.825	0.730	0.592	0.784	0.726
SVM	56	0.916	0.714	0.685	0.821	0.731	0.595	0.784	0.728
SVM	57	0.904	0.710	0.689	0.830	0.732	0.596	0.784	0.728
SVM	58	0.908	0.712	0.689	0.830	0.733	0.597	0.785	0.729
SVM	59	0.912	0.703	0.676	0.821	0.723	0.582	0.778	0.719
SVM	60	0.908	0.699	0.678	0.816	0.722	0.580	0.775	0.718
SVM	61	0.908	0.698	0.680	0.838	0.724	0.583	0.781	0.720
SVM	62	0.908	0.706	0.682	0.832	0.728	0.589	0.782	0.724
SVM	63	0.908	0.696	0.675	0.834	0.721	0.578	0.778	0.717
SVM	64	0.912	0.701	0.673	0.826	0.722	0.580	0.778	0.718
SVM	65	0.912	0.702	0.676	0.826	0.723	0.582	0.779	0.719
SVM	66	0.920	0.705	0.680	0.843	0.728	0.590	0.787	0.724
SVM	67	0.916	0.692	0.672	0.846	0.720	0.578	0.782	0.716
SVM	68	0.908	0.701	0.689	0.850	0.731	0.593	0.787	0.727
SVM	69	0.912	0.705	0.694	0.849	0.734	0.598	0.790	0.731
SVM	70	0.912	0.703	0.687	0.847	0.731	0.594	0.787	0.727
SVM	71	0.920	0.705	0.689	0.847	0.733	0.596	0.790	0.729
SVM	72	0.929	0.703	0.692	0.860	0.735	0.599	0.796	0.731
SVM	73	0.924	0.707	0.694	0.859	0.737	0.603	0.796	0.733

(2) IFS results on the LightGBM feature list

Classification algorithms	Number of features	Unvaccinated healthcare workers	Healthcare workers within 60 days after vaccination	Healthcare workers between 60 and180 days after vaccination	Healthcare workers over 180 days after vaccination	ACC	MCC	Macro F1	Weighted F1
DT	1	0.364	0.542	0.392	0.397	0.444	0.225	0.424	0.449
DT	2	0.852	0.593	0.564	0.493	0.588	0.395	0.625	0.590
DT	3	0.858	0.583	0.581	0.501	0.594	0.400	0.631	0.595
DT	4	0.833	0.637	0.626	0.670	0.653	0.482	0.691	0.650
DT	5	0.884	0.650	0.643	0.689	0.671	0.503	0.716	0.669
DT	6	0.851	0.670	0.663	0.722	0.688	0.530	0.726	0.686
DT	7	0.876	0.663	0.674	0.766	0.697	0.541	0.745	0.694
DT	8	0.848	0.665	0.658	0.705	0.682	0.522	0.719	0.680
DT	9	0.885	0.679	0.660	0.717	0.693	0.537	0.735	0.690
DT	10	0.881	0.667	0.672	0.767	0.698	0.539	0.747	0.696
DT	11	0.850	0.655	0.650	0.738	0.679	0.515	0.723	0.676
DT	12	0.909	0.671	0.667	0.708	0.693	0.534	0.739	0.691
DT	13	0.853	0.687	0.696	0.779	0.715	0.568	0.754	0.713
DT	14	0.869	0.691	0.685	0.770	0.712	0.564	0.754	0.710
DT	15	0.876	0.690	0.674	0.749	0.705	0.553	0.747	0.703
DT	16	0.877	0.653	0.654	0.766	0.685	0.521	0.738	0.682
DT	17	0.867	0.670	0.663	0.751	0.693	0.535	0.738	0.690
DT	18	0.874	0.657	0.661	0.748	0.687	0.525	0.735	0.684
DT	19	0.852	0.689	0.693	0.752	0.712	0.565	0.746	0.710
DT	20	0.852	0.677	0.686	0.768	0.706	0.554	0.746	0.704

DT	21	0.847	0.671	0.682	0.743	0.699	0.545	0.736	0.697
DT	22	0.878	0.670	0.657	0.738	0.690	0.531	0.736	0.687
DT	23	0.862	0.679	0.671	0.733	0.697	0.543	0.736	0.695
DT	24	0.909	0.665	0.654	0.756	0.691	0.534	0.746	0.688
DT	25	0.862	0.685	0.670	0.747	0.701	0.548	0.741	0.698
DT	26	0.856	0.700	0.685	0.727	0.709	0.562	0.742	0.708
DT	27	0.889	0.676	0.692	0.722	0.706	0.554	0.745	0.704
DT	28	0.850	0.660	0.658	0.738	0.685	0.526	0.726	0.681
DT	29	0.888	0.662	0.661	0.738	0.689	0.528	0.737	0.687
DT	30	0.878	0.687	0.682	0.786	0.712	0.564	0.759	0.710
DT	31	0.850	0.690	0.674	0.738	0.703	0.555	0.738	0.700
DT	32	0.848	0.665	0.680	0.739	0.696	0.540	0.733	0.693
DT	33	0.896	0.654	0.675	0.767	0.696	0.540	0.748	0.693
DT	34	0.872	0.683	0.696	0.796	0.717	0.573	0.762	0.715
DT	35	0.852	0.674	0.660	0.739	0.690	0.533	0.731	0.688
DT	36	0.888	0.692	0.695	0.770	0.718	0.572	0.761	0.716
DT	37	0.840	0.661	0.657	0.758	0.686	0.528	0.729	0.683
DT	38	0.844	0.673	0.665	0.724	0.690	0.531	0.727	0.688
DT	39	0.843	0.652	0.650	0.740	0.677	0.514	0.721	0.675
DT	40	0.877	0.704	0.687	0.778	0.720	0.573	0.762	0.717
DT	41	0.901	0.667	0.672	0.766	0.700	0.546	0.752	0.697
DT	42	0.860	0.652	0.666	0.748	0.687	0.527	0.732	0.684
DT	43	0.865	0.677	0.671	0.741	0.697	0.541	0.738	0.695
DT	44	0.906	0.684	0.678	0.746	0.706	0.555	0.753	0.704
DT	45	0.841	0.654	0.653	0.734	0.679	0.516	0.720	0.676

DT	46	0.876	0.677	0.652	0.701	0.685	0.527	0.726	0.684
DT	47	0.873	0.660	0.666	0.759	0.692	0.533	0.740	0.689
DT	48	0.857	0.689	0.661	0.755	0.700	0.548	0.741	0.697
DT	49	0.846	0.677	0.670	0.761	0.698	0.543	0.738	0.695
DT	50	0.839	0.649	0.655	0.731	0.677	0.509	0.719	0.674
DT	51	0.830	0.706	0.677	0.710	0.706	0.562	0.731	0.703
DT	52	0.853	0.661	0.667	0.719	0.687	0.528	0.725	0.684
DT	53	0.842	0.662	0.658	0.725	0.682	0.518	0.722	0.681
DT	54	0.853	0.674	0.661	0.723	0.690	0.533	0.728	0.687
DT	55	0.908	0.674	0.668	0.727	0.696	0.540	0.744	0.694
DT	56	0.865	0.704	0.693	0.745	0.717	0.572	0.752	0.715
DT	57	0.843	0.651	0.641	0.722	0.672	0.506	0.714	0.668
DT	58	0.874	0.686	0.671	0.775	0.706	0.554	0.752	0.703
DT	59	0.852	0.688	0.668	0.738	0.699	0.546	0.737	0.697
DT	60	0.872	0.665	0.670	0.759	0.695	0.537	0.742	0.693
DT	61	0.850	0.682	0.653	0.737	0.690	0.534	0.730	0.688
DT	62	0.877	0.658	0.656	0.712	0.681	0.516	0.726	0.679
DT	63	0.830	0.651	0.649	0.756	0.677	0.510	0.722	0.675
DT	64	0.883	0.670	0.663	0.673	0.685	0.524	0.722	0.683
DT	65	0.850	0.676	0.654	0.711	0.685	0.523	0.723	0.683
DT	66	0.856	0.683	0.678	0.709	0.698	0.546	0.731	0.697
DT	67	0.845	0.638	0.641	0.727	0.667	0.497	0.713	0.664
DT	68	0.844	0.669	0.657	0.716	0.685	0.525	0.721	0.682
DT	69	0.865	0.694	0.696	0.731	0.713	0.565	0.747	0.712
DT	70	0.838	0.652	0.635	0.751	0.672	0.501	0.719	0.669

DT	71	0.824	0.665	0.671	0.727	0.688	0.529	0.722	0.686
DT	72	0.857	0.655	0.669	0.749	0.688	0.528	0.732	0.686
DT	73	0.870	0.677	0.667	0.753	0.698	0.542	0.742	0.695
KNN	1	0.349	0.512	0.371	0.371	0.420	0.190	0.401	0.424
KNN	2	0.871	0.606	0.582	0.546	0.610	0.433	0.651	0.609
KNN	3	0.890	0.629	0.630	0.615	0.649	0.487	0.691	0.648
KNN	4	0.890	0.653	0.651	0.750	0.684	0.527	0.736	0.680
KNN	5	0.915	0.672	0.655	0.772	0.698	0.547	0.754	0.694
KNN	6	0.920	0.681	0.657	0.795	0.705	0.559	0.763	0.700
KNN	7	0.880	0.669	0.665	0.773	0.698	0.550	0.747	0.694
KNN	8	0.895	0.700	0.672	0.783	0.715	0.575	0.762	0.711
KNN	9	0.924	0.714	0.679	0.797	0.728	0.594	0.778	0.723
KNN	10	0.932	0.694	0.678	0.838	0.723	0.581	0.785	0.720
KNN	11	0.887	0.682	0.686	0.811	0.716	0.571	0.766	0.712
KNN	12	0.863	0.678	0.667	0.844	0.709	0.566	0.763	0.704
KNN	13	0.867	0.685	0.675	0.835	0.714	0.573	0.766	0.710
KNN	14	0.872	0.702	0.698	0.858	0.733	0.600	0.783	0.729
KNN	15	0.900	0.708	0.698	0.848	0.736	0.605	0.788	0.732
KNN	16	0.893	0.698	0.680	0.851	0.725	0.589	0.780	0.720
KNN	17	0.884	0.706	0.693	0.828	0.731	0.596	0.778	0.726
KNN	18	0.928	0.723	0.705	0.850	0.747	0.618	0.802	0.744
KNN	19	0.908	0.705	0.695	0.841	0.734	0.600	0.787	0.730
KNN	20	0.928	0.705	0.700	0.854	0.739	0.604	0.797	0.735
KNN	21	0.912	0.698	0.693	0.856	0.732	0.595	0.790	0.728
KNN	22	0.929	0.715	0.699	0.846	0.741	0.610	0.797	0.738

KNN	23	0.950	0.713	0.699	0.859	0.744	0.613	0.805	0.740
KNN	24	0.932	0.690	0.684	0.853	0.726	0.586	0.790	0.722
KNN	25	0.923	0.695	0.687	0.839	0.727	0.587	0.786	0.724
KNN	26	0.912	0.686	0.664	0.840	0.714	0.571	0.776	0.710
KNN	27	0.924	0.683	0.665	0.842	0.714	0.570	0.779	0.710
KNN	28	0.920	0.703	0.680	0.858	0.730	0.593	0.790	0.725
KNN	29	0.924	0.708	0.685	0.860	0.734	0.600	0.794	0.730
KNN	30	0.941	0.714	0.684	0.852	0.736	0.603	0.798	0.732
KNN	31	0.912	0.704	0.686	0.854	0.731	0.595	0.789	0.727
KNN	32	0.920	0.693	0.679	0.862	0.725	0.585	0.788	0.721
KNN	33	0.932	0.706	0.677	0.852	0.730	0.593	0.792	0.726
KNN	34	0.920	0.703	0.689	0.861	0.733	0.597	0.793	0.730
KNN	35	0.912	0.704	0.687	0.843	0.731	0.594	0.787	0.727
KNN	36	0.927	0.714	0.691	0.854	0.739	0.606	0.797	0.735
KNN	37	0.924	0.706	0.679	0.859	0.731	0.596	0.792	0.727
KNN	38	0.929	0.711	0.682	0.858	0.734	0.599	0.795	0.730
KNN	39	0.923	0.717	0.674	0.848	0.732	0.598	0.791	0.727
KNN	40	0.924	0.704	0.673	0.839	0.725	0.584	0.785	0.721
KNN	41	0.897	0.707	0.673	0.854	0.726	0.588	0.782	0.722
KNN	42	0.924	0.698	0.670	0.855	0.724	0.585	0.787	0.719
KNN	43	0.920	0.705	0.673	0.853	0.728	0.591	0.788	0.723
KNN	44	0.924	0.710	0.673	0.836	0.728	0.591	0.786	0.723
KNN	45	0.916	0.713	0.678	0.840	0.731	0.598	0.787	0.726
KNN	46	0.937	0.717	0.675	0.830	0.732	0.599	0.790	0.727
KNN	47	0.912	0.707	0.673	0.837	0.726	0.591	0.782	0.721

KNN	48	0.907	0.706	0.669	0.831	0.723	0.587	0.778	0.718
KNN	49	0.899	0.718	0.680	0.835	0.732	0.600	0.783	0.727
KNN	50	0.904	0.696	0.654	0.813	0.711	0.568	0.767	0.706
KNN	51	0.904	0.705	0.673	0.828	0.724	0.588	0.778	0.719
KNN	52	0.920	0.697	0.668	0.828	0.720	0.579	0.778	0.715
KNN	53	0.904	0.703	0.665	0.815	0.718	0.578	0.772	0.713
KNN	54	0.916	0.696	0.664	0.822	0.717	0.575	0.775	0.712
KNN	55	0.916	0.694	0.674	0.843	0.722	0.581	0.782	0.717
KNN	56	0.908	0.683	0.665	0.835	0.712	0.567	0.773	0.708
KNN	57	0.912	0.671	0.653	0.821	0.702	0.554	0.764	0.697
KNN	58	0.916	0.672	0.653	0.819	0.702	0.553	0.765	0.697
KNN	59	0.920	0.680	0.651	0.815	0.705	0.559	0.767	0.700
KNN	60	0.924	0.686	0.656	0.825	0.710	0.565	0.773	0.705
KNN	61	0.893	0.682	0.673	0.834	0.714	0.572	0.770	0.710
KNN	62	0.920	0.684	0.655	0.810	0.707	0.561	0.767	0.702
KNN	63	0.933	0.699	0.665	0.827	0.720	0.581	0.781	0.715
KNN	64	0.897	0.691	0.664	0.830	0.714	0.573	0.771	0.709
KNN	65	0.916	0.683	0.668	0.837	0.714	0.571	0.776	0.710
KNN	66	0.941	0.695	0.667	0.824	0.719	0.578	0.782	0.714
KNN	67	0.900	0.700	0.669	0.833	0.721	0.584	0.776	0.716
KNN	68	0.916	0.679	0.662	0.820	0.709	0.562	0.769	0.704
KNN	69	0.916	0.696	0.667	0.817	0.717	0.578	0.774	0.712
KNN	70	0.941	0.690	0.656	0.793	0.709	0.564	0.770	0.705
KNN	71	0.924	0.698	0.664	0.813	0.717	0.578	0.775	0.712
KNN	72	0.928	0.689	0.657	0.801	0.709	0.566	0.769	0.705

KNN	73	0.907	0.690	0.667	0.806	0.713	0.571	0.768	0.708
RF	1	0.348	0.510	0.349	0.381	0.413	0.176	0.397	0.415
RF	2	0.852	0.640	0.530	0.475	0.595	0.411	0.624	0.592
RF	3	0.866	0.664	0.573	0.567	0.634	0.468	0.667	0.630
RF	4	0.874	0.689	0.610	0.711	0.678	0.528	0.721	0.671
RF	5	0.899	0.713	0.624	0.731	0.698	0.561	0.742	0.690
RF	6	0.900	0.725	0.646	0.744	0.713	0.581	0.754	0.706
RF	7	0.907	0.741	0.659	0.785	0.731	0.611	0.773	0.722
RF	8	0.908	0.738	0.638	0.782	0.722	0.598	0.767	0.712
RF	9	0.900	0.744	0.645	0.795	0.728	0.606	0.771	0.718
RF	10	0.892	0.731	0.629	0.795	0.716	0.590	0.762	0.706
RF	11	0.896	0.736	0.629	0.811	0.720	0.598	0.768	0.709
RF	12	0.900	0.733	0.632	0.808	0.720	0.597	0.768	0.710
RF	13	0.904	0.734	0.623	0.806	0.717	0.594	0.766	0.706
RF	14	0.920	0.737	0.619	0.818	0.721	0.603	0.774	0.708
RF	15	0.920	0.734	0.612	0.826	0.718	0.599	0.773	0.705
RF	16	0.907	0.746	0.629	0.829	0.729	0.616	0.778	0.716
RF	17	0.896	0.758	0.658	0.840	0.744	0.637	0.788	0.733
RF	18	0.908	0.759	0.660	0.828	0.745	0.636	0.789	0.735
RF	19	0.889	0.751	0.655	0.831	0.739	0.627	0.781	0.728
RF	20	0.897	0.762	0.665	0.825	0.747	0.638	0.787	0.737
RF	21	0.897	0.758	0.656	0.837	0.744	0.635	0.787	0.733
RF	22	0.904	0.746	0.654	0.836	0.737	0.621	0.785	0.727
RF	23	0.908	0.754	0.661	0.836	0.744	0.632	0.790	0.734
RF	24	0.908	0.753	0.651	0.831	0.739	0.626	0.786	0.729

RF	25	0.908	0.751	0.651	0.839	0.739	0.627	0.787	0.729
RF	26	0.912	0.755	0.652	0.831	0.741	0.628	0.788	0.730
RF	27	0.904	0.760	0.660	0.835	0.746	0.638	0.790	0.735
RF	28	0.900	0.741	0.639	0.829	0.729	0.611	0.777	0.718
RF	29	0.904	0.755	0.656	0.830	0.741	0.630	0.786	0.731
RF	30	0.912	0.757	0.663	0.825	0.744	0.631	0.789	0.735
RF	31	0.912	0.765	0.669	0.839	0.752	0.649	0.796	0.742
RF	32	0.916	0.748	0.654	0.840	0.739	0.624	0.790	0.730
RF	33	0.920	0.754	0.665	0.846	0.747	0.634	0.796	0.737
RF	34	0.912	0.761	0.663	0.832	0.747	0.638	0.792	0.738
RF	35	0.916	0.752	0.644	0.836	0.738	0.627	0.787	0.726
RF	36	0.916	0.755	0.659	0.832	0.743	0.630	0.790	0.733
RF	37	0.920	0.754	0.652	0.842	0.742	0.631	0.792	0.731
RF	38	0.916	0.750	0.654	0.833	0.739	0.625	0.788	0.729
RF	39	0.916	0.745	0.643	0.834	0.734	0.619	0.785	0.723
RF	40	0.916	0.749	0.644	0.830	0.736	0.621	0.785	0.725
RF	41	0.916	0.757	0.654	0.839	0.744	0.635	0.791	0.733
RF	42	0.920	0.757	0.647	0.830	0.741	0.631	0.789	0.729
RF	43	0.916	0.739	0.624	0.822	0.723	0.604	0.775	0.711
RF	44	0.912	0.757	0.649	0.831	0.741	0.631	0.787	0.729
RF	45	0.908	0.754	0.656	0.841	0.742	0.630	0.790	0.732
RF	46	0.920	0.759	0.648	0.828	0.741	0.632	0.789	0.730
RF	47	0.916	0.753	0.642	0.834	0.738	0.628	0.787	0.726
RF	48	0.908	0.750	0.639	0.828	0.734	0.624	0.781	0.722
RF	49	0.920	0.763	0.653	0.823	0.744	0.636	0.790	0.733

RF	50	0.908	0.751	0.646	0.840	0.738	0.626	0.786	0.727
RF	51	0.908	0.748	0.645	0.834	0.736	0.622	0.784	0.725
RF	52	0.912	0.752	0.647	0.839	0.739	0.627	0.787	0.727
RF	53	0.904	0.754	0.651	0.835	0.740	0.630	0.786	0.729
RF	54	0.912	0.741	0.630	0.840	0.728	0.613	0.781	0.716
RF	55	0.904	0.758	0.650	0.836	0.742	0.635	0.787	0.730
RF	56	0.908	0.749	0.647	0.835	0.736	0.621	0.785	0.726
RF	57	0.900	0.753	0.640	0.837	0.736	0.627	0.783	0.724
RF	58	0.916	0.748	0.635	0.830	0.733	0.620	0.783	0.721
RF	59	0.912	0.755	0.653	0.841	0.742	0.631	0.790	0.732
RF	60	0.920	0.750	0.641	0.842	0.737	0.626	0.788	0.725
RF	61	0.908	0.751	0.633	0.834	0.733	0.624	0.781	0.720
RF	62	0.920	0.754	0.644	0.839	0.739	0.629	0.789	0.728
RF	63	0.912	0.759	0.644	0.831	0.741	0.634	0.787	0.728
RF	64	0.912	0.755	0.644	0.835	0.739	0.629	0.787	0.728
RF	65	0.916	0.753	0.645	0.838	0.739	0.626	0.788	0.727
RF	66	0.912	0.751	0.643	0.830	0.736	0.625	0.784	0.725
RF	67	0.904	0.763	0.647	0.830	0.743	0.637	0.786	0.731
RF	68	0.908	0.744	0.630	0.833	0.728	0.612	0.779	0.716
RF	69	0.904	0.752	0.640	0.830	0.735	0.623	0.782	0.723
RF	70	0.912	0.744	0.626	0.827	0.727	0.610	0.777	0.714
RF	71	0.916	0.753	0.652	0.833	0.740	0.627	0.788	0.730
RF	72	0.904	0.748	0.638	0.828	0.732	0.617	0.779	0.720
RF	73	0.908	0.747	0.644	0.847	0.736	0.620	0.786	0.725
SVM	1	0.314	0.720	0.000	0.384	0.456	0.320	0.354	0.343

SVM	2	0.810	0.702	0.450	0.344	0.594	0.424	0.576	0.564
SVM	3	0.844	0.696	0.529	0.500	0.634	0.484	0.642	0.615
SVM	4	0.825	0.729	0.599	0.706	0.692	0.569	0.715	0.678
SVM	5	0.893	0.734	0.605	0.745	0.705	0.585	0.744	0.691
SVM	6	0.866	0.720	0.616	0.749	0.699	0.566	0.738	0.689
SVM	7	0.873	0.720	0.613	0.753	0.699	0.567	0.740	0.689
SVM	8	0.877	0.725	0.618	0.791	0.708	0.578	0.753	0.697
SVM	9	0.893	0.729	0.612	0.799	0.710	0.583	0.758	0.698
SVM	10	0.876	0.725	0.630	0.834	0.717	0.591	0.766	0.706
SVM	11	0.874	0.720	0.619	0.809	0.707	0.575	0.755	0.697
SVM	12	0.880	0.712	0.618	0.809	0.704	0.566	0.755	0.694
SVM	13	0.879	0.726	0.635	0.825	0.717	0.587	0.766	0.708
SVM	14	0.887	0.704	0.637	0.828	0.709	0.568	0.764	0.702
SVM	15	0.892	0.704	0.628	0.812	0.704	0.563	0.759	0.697
SVM	16	0.888	0.705	0.630	0.791	0.702	0.559	0.753	0.695
SVM	17	0.871	0.725	0.679	0.814	0.730	0.597	0.772	0.725
SVM	18	0.880	0.735	0.688	0.826	0.740	0.614	0.782	0.735
SVM	19	0.888	0.740	0.697	0.826	0.746	0.622	0.788	0.741
SVM	20	0.878	0.737	0.697	0.818	0.743	0.616	0.782	0.739
SVM	21	0.878	0.736	0.696	0.822	0.742	0.615	0.783	0.738
SVM	22	0.870	0.734	0.695	0.816	0.740	0.613	0.779	0.736
SVM	23	0.878	0.735	0.693	0.814	0.740	0.612	0.780	0.736
SVM	24	0.889	0.741	0.695	0.815	0.744	0.620	0.785	0.740
SVM	25	0.885	0.739	0.694	0.815	0.743	0.618	0.783	0.738
SVM	26	0.893	0.730	0.692	0.851	0.743	0.617	0.791	0.738

SVM	27	0.897	0.731	0.691	0.846	0.743	0.616	0.791	0.738
SVM	28	0.897	0.720	0.679	0.845	0.733	0.601	0.785	0.729
SVM	29	0.878	0.728	0.698	0.855	0.744	0.617	0.790	0.739
SVM	30	0.878	0.731	0.711	0.870	0.752	0.627	0.797	0.748
SVM	31	0.893	0.725	0.708	0.873	0.749	0.623	0.800	0.746
SVM	32	0.885	0.729	0.714	0.864	0.752	0.627	0.798	0.748
SVM	33	0.870	0.730	0.717	0.863	0.752	0.629	0.795	0.749
SVM	34	0.881	0.737	0.726	0.881	0.761	0.641	0.806	0.758
SVM	35	0.885	0.735	0.729	0.875	0.761	0.640	0.806	0.758
SVM	36	0.878	0.729	0.719	0.863	0.753	0.629	0.797	0.750
SVM	37	0.874	0.724	0.716	0.863	0.749	0.623	0.794	0.746
SVM	38	0.870	0.724	0.716	0.862	0.749	0.624	0.793	0.746
SVM	39	0.867	0.723	0.720	0.870	0.751	0.625	0.795	0.747
SVM	40	0.878	0.716	0.714	0.873	0.747	0.619	0.795	0.744
SVM	41	0.878	0.716	0.712	0.866	0.746	0.617	0.793	0.742
SVM	42	0.889	0.719	0.707	0.869	0.746	0.617	0.796	0.742
SVM	43	0.881	0.729	0.714	0.848	0.750	0.626	0.793	0.746
SVM	44	0.897	0.716	0.704	0.864	0.744	0.614	0.795	0.740
SVM	45	0.893	0.718	0.708	0.864	0.746	0.617	0.796	0.742
SVM	46	0.904	0.720	0.707	0.841	0.744	0.615	0.793	0.741
SVM	47	0.900	0.716	0.703	0.841	0.741	0.610	0.790	0.737
SVM	48	0.893	0.714	0.702	0.854	0.741	0.610	0.791	0.737
SVM	49	0.893	0.714	0.699	0.847	0.739	0.607	0.788	0.735
SVM	50	0.908	0.721	0.709	0.849	0.747	0.618	0.797	0.743
SVM	51	0.920	0.720	0.701	0.841	0.743	0.612	0.796	0.739

SVM	52	0.920	0.727	0.709	0.847	0.749	0.622	0.801	0.746
SVM	53	0.920	0.727	0.705	0.844	0.747	0.619	0.799	0.744
SVM	54	0.920	0.726	0.704	0.840	0.747	0.618	0.798	0.743
SVM	55	0.916	0.724	0.700	0.839	0.744	0.614	0.795	0.740
SVM	56	0.916	0.700	0.683	0.842	0.728	0.589	0.785	0.724
SVM	57	0.912	0.700	0.686	0.847	0.729	0.591	0.786	0.725
SVM	58	0.904	0.699	0.694	0.858	0.733	0.596	0.789	0.729
SVM	59	0.904	0.697	0.691	0.858	0.731	0.593	0.788	0.727
SVM	60	0.904	0.699	0.696	0.858	0.733	0.597	0.789	0.730
SVM	61	0.900	0.699	0.695	0.858	0.733	0.596	0.788	0.729
SVM	62	0.908	0.696	0.688	0.863	0.730	0.592	0.789	0.726
SVM	63	0.916	0.686	0.679	0.863	0.723	0.580	0.786	0.719
SVM	64	0.916	0.685	0.678	0.863	0.722	0.579	0.786	0.718
SVM	65	0.916	0.697	0.688	0.863	0.731	0.592	0.791	0.727
SVM	66	0.920	0.696	0.691	0.866	0.732	0.594	0.793	0.728
SVM	67	0.916	0.699	0.692	0.863	0.733	0.596	0.793	0.729
SVM	68	0.916	0.702	0.688	0.858	0.732	0.595	0.791	0.728
SVM	69	0.924	0.699	0.688	0.853	0.731	0.593	0.791	0.727
SVM	70	0.924	0.695	0.687	0.854	0.729	0.591	0.790	0.725
SVM	71	0.924	0.699	0.690	0.862	0.733	0.596	0.794	0.729
SVM	72	0.924	0.701	0.690	0.859	0.733	0.597	0.794	0.730
SVM	73	0.924	0.707	0.694	0.859	0.737	0.603	0.796	0.733

(3) IFS results on the MCFS feature list

Classification algorithms	Number of features	Unvaccinated healthcare workers	Healthcare workers within 60 days after vaccination	Healthcare workers between 60 and180 days after vaccination	Healthcare workers over 180 days after vaccination	ACC	MCC	Macro F1	Weighted F1
DT	1	0.783	0.537	0.529	0.297	0.517	0.304	0.537	0.527
DT	2	0.817	0.583	0.557	0.489	0.579	0.383	0.611	0.579
DT	3	0.853	0.589	0.571	0.610	0.607	0.416	0.656	0.603
DT	4	0.834	0.641	0.621	0.604	0.644	0.465	0.675	0.643
DT	5	0.841	0.625	0.618	0.640	0.642	0.462	0.681	0.640
DT	6	0.863	0.616	0.622	0.677	0.646	0.465	0.694	0.643
DT	7	0.865	0.648	0.617	0.630	0.651	0.482	0.690	0.649
DT	8	0.888	0.655	0.635	0.653	0.666	0.498	0.708	0.664
DT	9	0.875	0.679	0.650	0.673	0.682	0.523	0.719	0.681
DT	10	0.892	0.683	0.662	0.659	0.688	0.534	0.724	0.687
DT	11	0.883	0.659	0.647	0.732	0.681	0.520	0.730	0.678
DT	12	0.911	0.672	0.677	0.713	0.698	0.543	0.743	0.697
DT	13	0.877	0.680	0.677	0.775	0.706	0.553	0.752	0.704
DT	14	0.870	0.686	0.695	0.770	0.714	0.567	0.755	0.712
DT	15	0.883	0.658	0.670	0.744	0.691	0.530	0.739	0.689
DT	16	0.862	0.702	0.694	0.754	0.717	0.571	0.753	0.716
DT	17	0.885	0.709	0.701	0.787	0.729	0.589	0.771	0.727
DT	18	0.857	0.668	0.667	0.743	0.692	0.535	0.734	0.689
DT	19	0.861	0.708	0.682	0.753	0.715	0.571	0.751	0.713
DT	20	0.842	0.689	0.680	0.791	0.710	0.562	0.751	0.707
DT	21	0.840	0.699	0.689	0.744	0.712	0.567	0.743	0.710

DT	22	0.869	0.681	0.673	0.753	0.701	0.549	0.744	0.699
DT	23	0.888	0.695	0.687	0.776	0.717	0.575	0.762	0.715
DT	24	0.873	0.693	0.683	0.752	0.710	0.561	0.750	0.708
DT	25	0.866	0.707	0.696	0.765	0.723	0.581	0.759	0.720
DT	26	0.877	0.683	0.673	0.740	0.701	0.549	0.743	0.699
DT	27	0.828	0.658	0.670	0.790	0.692	0.532	0.736	0.689
DT	28	0.852	0.705	0.681	0.780	0.716	0.571	0.755	0.713
DT	29	0.867	0.656	0.654	0.760	0.685	0.526	0.734	0.682
DT	30	0.853	0.684	0.665	0.765	0.700	0.547	0.742	0.697
DT	31	0.857	0.698	0.687	0.768	0.714	0.568	0.752	0.712
DT	32	0.863	0.653	0.651	0.790	0.685	0.524	0.739	0.682
DT	33	0.866	0.674	0.677	0.791	0.704	0.551	0.752	0.702
DT	34	0.879	0.665	0.656	0.779	0.691	0.530	0.745	0.689
DT	35	0.880	0.699	0.679	0.721	0.708	0.558	0.745	0.706
DT	36	0.886	0.655	0.660	0.767	0.689	0.528	0.742	0.686
DT	37	0.874	0.686	0.684	0.804	0.714	0.565	0.762	0.712
DT	38	0.865	0.682	0.681	0.745	0.704	0.553	0.743	0.702
DT	39	0.878	0.673	0.654	0.749	0.690	0.530	0.739	0.688
DT	40	0.857	0.674	0.660	0.765	0.694	0.538	0.739	0.691
DT	41	0.862	0.699	0.693	0.751	0.716	0.569	0.751	0.714
DT	42	0.840	0.638	0.652	0.751	0.674	0.508	0.720	0.671
DT	43	0.903	0.679	0.679	0.768	0.708	0.556	0.757	0.705
DT	44	0.851	0.643	0.679	0.785	0.692	0.532	0.739	0.689
DT	45	0.895	0.692	0.675	0.748	0.708	0.557	0.753	0.706
DT	46	0.887	0.688	0.679	0.751	0.707	0.556	0.751	0.705

DT	47	0.838	0.678	0.696	0.723	0.704	0.555	0.734	0.702
DT	48	0.870	0.669	0.663	0.713	0.688	0.525	0.729	0.686
DT	49	0.870	0.675	0.666	0.739	0.695	0.539	0.737	0.692
DT	50	0.873	0.676	0.674	0.728	0.697	0.540	0.738	0.695
DT	51	0.879	0.686	0.674	0.748	0.705	0.556	0.747	0.702
DT	52	0.844	0.686	0.677	0.758	0.704	0.551	0.741	0.701
DT	53	0.877	0.682	0.668	0.760	0.701	0.547	0.747	0.699
DT	54	0.878	0.670	0.677	0.762	0.701	0.548	0.747	0.698
DT	55	0.890	0.656	0.656	0.742	0.685	0.522	0.736	0.682
DT	56	0.856	0.672	0.667	0.705	0.689	0.530	0.725	0.687
DT	57	0.851	0.643	0.650	0.747	0.675	0.509	0.723	0.672
DT	58	0.850	0.660	0.671	0.703	0.685	0.524	0.721	0.684
DT	59	0.851	0.679	0.695	0.774	0.711	0.561	0.750	0.708
DT	60	0.847	0.689	0.674	0.728	0.700	0.546	0.734	0.698
DT	61	0.887	0.672	0.678	0.772	0.704	0.549	0.752	0.701
DT	62	0.873	0.675	0.694	0.778	0.712	0.564	0.755	0.709
DT	63	0.878	0.682	0.679	0.745	0.704	0.552	0.746	0.702
DT	64	0.880	0.686	0.674	0.731	0.702	0.548	0.743	0.700
DT	65	0.846	0.689	0.686	0.740	0.706	0.557	0.740	0.704
DT	66	0.871	0.668	0.650	0.729	0.685	0.525	0.729	0.682
DT	67	0.875	0.690	0.698	0.746	0.715	0.571	0.752	0.713
DT	68	0.871	0.685	0.684	0.774	0.711	0.566	0.753	0.708
DT	69	0.815	0.639	0.675	0.742	0.682	0.520	0.718	0.679
DT	70	0.892	0.695	0.667	0.705	0.701	0.548	0.740	0.699
DT	71	0.843	0.659	0.674	0.778	0.694	0.536	0.738	0.691

DT	72	0.880	0.692	0.690	0.728	0.711	0.563	0.747	0.709
DT	73	0.850	0.633	0.630	0.740	0.662	0.489	0.713	0.659
KNN	1	0.797	0.550	0.522	0.318	0.522	0.315	0.547	0.533
KNN	2	0.841	0.577	0.578	0.565	0.598	0.414	0.640	0.596
KNN	3	0.870	0.618	0.596	0.700	0.641	0.466	0.696	0.636
KNN	4	0.896	0.673	0.653	0.759	0.695	0.546	0.745	0.690
KNN	5	0.884	0.684	0.659	0.758	0.701	0.557	0.746	0.696
KNN	6	0.876	0.667	0.643	0.786	0.690	0.539	0.743	0.685
KNN	7	0.876	0.684	0.639	0.756	0.692	0.545	0.739	0.686
KNN	8	0.891	0.686	0.643	0.765	0.696	0.549	0.746	0.691
KNN	9	0.923	0.701	0.651	0.762	0.707	0.564	0.759	0.703
KNN	10	0.933	0.706	0.653	0.794	0.715	0.578	0.772	0.709
KNN	11	0.924	0.697	0.650	0.795	0.709	0.564	0.766	0.704
KNN	12	0.941	0.724	0.687	0.825	0.739	0.607	0.794	0.735
KNN	13	0.916	0.708	0.680	0.830	0.728	0.591	0.784	0.724
KNN	14	0.912	0.701	0.680	0.833	0.726	0.590	0.782	0.722
KNN	15	0.932	0.708	0.681	0.829	0.730	0.594	0.787	0.726
KNN	16	0.900	0.705	0.682	0.808	0.725	0.588	0.774	0.720
KNN	17	0.916	0.716	0.689	0.843	0.736	0.603	0.791	0.732
KNN	18	0.920	0.713	0.691	0.828	0.735	0.601	0.788	0.731
KNN	19	0.929	0.719	0.687	0.846	0.739	0.607	0.795	0.734
KNN	20	0.937	0.715	0.700	0.844	0.742	0.611	0.799	0.739
KNN	21	0.911	0.705	0.695	0.858	0.736	0.601	0.792	0.732
KNN	22	0.900	0.692	0.686	0.843	0.725	0.585	0.780	0.721
KNN	23	0.907	0.706	0.692	0.862	0.735	0.599	0.792	0.731

KNN	24	0.916	0.713	0.702	0.854	0.741	0.609	0.796	0.738
KNN	25	0.929	0.717	0.689	0.870	0.741	0.612	0.801	0.737
KNN	26	0.933	0.712	0.686	0.855	0.736	0.603	0.796	0.732
KNN	27	0.929	0.719	0.697	0.847	0.742	0.611	0.798	0.739
KNN	28	0.920	0.694	0.676	0.853	0.724	0.583	0.786	0.720
KNN	29	0.907	0.710	0.690	0.860	0.736	0.602	0.792	0.731
KNN	30	0.916	0.691	0.665	0.852	0.718	0.576	0.781	0.713
KNN	31	0.924	0.710	0.679	0.838	0.731	0.595	0.788	0.726
KNN	32	0.908	0.705	0.672	0.849	0.726	0.590	0.784	0.721
KNN	33	0.881	0.703	0.674	0.855	0.725	0.589	0.778	0.719
KNN	34	0.916	0.700	0.678	0.843	0.726	0.589	0.784	0.722
KNN	35	0.900	0.706	0.678	0.844	0.728	0.592	0.782	0.723
KNN	36	0.897	0.699	0.673	0.847	0.723	0.586	0.779	0.718
KNN	37	0.904	0.707	0.667	0.836	0.723	0.587	0.779	0.718
KNN	38	0.912	0.706	0.672	0.836	0.725	0.589	0.782	0.720
KNN	39	0.889	0.689	0.662	0.857	0.715	0.573	0.774	0.710
KNN	40	0.904	0.695	0.664	0.859	0.720	0.580	0.780	0.714
KNN	41	0.904	0.696	0.657	0.863	0.717	0.577	0.780	0.712
KNN	42	0.884	0.693	0.656	0.816	0.709	0.566	0.762	0.704
KNN	43	0.889	0.706	0.671	0.828	0.723	0.586	0.774	0.717
KNN	44	0.874	0.705	0.678	0.835	0.725	0.590	0.773	0.720
KNN	45	0.900	0.710	0.682	0.839	0.730	0.594	0.783	0.726
KNN	46	0.924	0.713	0.689	0.840	0.736	0.602	0.792	0.732
KNN	47	0.912	0.713	0.686	0.841	0.734	0.602	0.788	0.730
KNN	48	0.904	0.720	0.690	0.841	0.738	0.607	0.789	0.733

KNN	49	0.908	0.722	0.697	0.840	0.741	0.613	0.792	0.737
KNN	50	0.920	0.708	0.682	0.840	0.731	0.595	0.788	0.726
KNN	51	0.924	0.685	0.657	0.815	0.709	0.565	0.770	0.704
KNN	52	0.920	0.693	0.655	0.829	0.713	0.571	0.774	0.708
KNN	53	0.912	0.684	0.656	0.832	0.710	0.567	0.771	0.705
KNN	54	0.904	0.681	0.646	0.819	0.703	0.557	0.763	0.697
KNN	55	0.912	0.692	0.660	0.811	0.712	0.572	0.769	0.707
KNN	56	0.929	0.690	0.653	0.820	0.711	0.567	0.773	0.706
KNN	57	0.920	0.699	0.656	0.806	0.713	0.572	0.770	0.708
KNN	58	0.904	0.701	0.663	0.823	0.718	0.580	0.773	0.713
KNN	59	0.920	0.699	0.653	0.811	0.713	0.573	0.771	0.707
KNN	60	0.912	0.698	0.655	0.819	0.714	0.574	0.771	0.708
KNN	61	0.904	0.691	0.652	0.815	0.709	0.567	0.765	0.703
KNN	62	0.904	0.690	0.653	0.819	0.709	0.565	0.766	0.703
KNN	63	0.920	0.690	0.654	0.802	0.709	0.564	0.767	0.704
KNN	64	0.900	0.694	0.664	0.822	0.715	0.575	0.770	0.710
KNN	65	0.904	0.687	0.656	0.817	0.709	0.565	0.766	0.703
KNN	66	0.916	0.695	0.661	0.813	0.714	0.572	0.771	0.709
KNN	67	0.912	0.686	0.653	0.786	0.704	0.559	0.759	0.699
KNN	68	0.937	0.690	0.661	0.807	0.713	0.570	0.774	0.708
KNN	69	0.920	0.696	0.668	0.818	0.718	0.579	0.775	0.713
KNN	70	0.933	0.697	0.666	0.806	0.717	0.577	0.775	0.713
KNN	71	0.929	0.677	0.650	0.805	0.703	0.556	0.765	0.698
KNN	72	0.920	0.683	0.663	0.793	0.708	0.562	0.765	0.704
KNN	73	0.924	0.694	0.662	0.799	0.713	0.571	0.770	0.708

RF	1	0.762	0.540	0.511	0.341	0.518	0.308	0.539	0.524
RF	2	0.846	0.594	0.573	0.482	0.591	0.405	0.624	0.592
RF	3	0.862	0.641	0.587	0.659	0.642	0.472	0.688	0.637
RF	4	0.882	0.665	0.601	0.648	0.656	0.492	0.699	0.652
RF	5	0.878	0.686	0.611	0.670	0.672	0.516	0.711	0.666
RF	6	0.874	0.697	0.626	0.708	0.686	0.538	0.726	0.681
RF	7	0.874	0.708	0.630	0.717	0.693	0.547	0.732	0.688
RF	8	0.912	0.713	0.643	0.735	0.705	0.564	0.750	0.700
RF	9	0.919	0.721	0.640	0.751	0.710	0.572	0.758	0.704
RF	10	0.912	0.733	0.659	0.774	0.725	0.595	0.769	0.719
RF	11	0.924	0.734	0.661	0.778	0.728	0.599	0.774	0.721
RF	12	0.915	0.736	0.663	0.812	0.733	0.609	0.781	0.726
RF	13	0.911	0.743	0.665	0.811	0.736	0.616	0.782	0.729
RF	14	0.912	0.747	0.675	0.794	0.740	0.622	0.782	0.733
RF	15	0.920	0.744	0.674	0.821	0.741	0.620	0.790	0.735
RF	16	0.912	0.760	0.684	0.836	0.754	0.642	0.798	0.746
RF	17	0.912	0.751	0.667	0.835	0.744	0.628	0.791	0.735
RF	18	0.920	0.756	0.670	0.847	0.749	0.639	0.798	0.741
RF	19	0.912	0.748	0.668	0.840	0.744	0.628	0.792	0.735
RF	20	0.916	0.755	0.675	0.840	0.749	0.637	0.797	0.741
RF	21	0.912	0.761	0.667	0.832	0.749	0.639	0.793	0.739
RF	22	0.912	0.750	0.656	0.829	0.739	0.625	0.787	0.730
RF	23	0.916	0.765	0.679	0.843	0.756	0.649	0.801	0.747
RF	24	0.916	0.751	0.665	0.841	0.744	0.630	0.793	0.736
RF	25	0.912	0.759	0.670	0.843	0.750	0.642	0.796	0.741

RF	26	0.908	0.749	0.661	0.844	0.742	0.628	0.791	0.733
RF	27	0.912	0.748	0.652	0.841	0.739	0.624	0.788	0.728
RF	28	0.916	0.756	0.669	0.844	0.749	0.638	0.797	0.740
RF	29	0.912	0.748	0.654	0.828	0.738	0.622	0.786	0.728
RF	30	0.920	0.746	0.658	0.848	0.741	0.626	0.793	0.731
RF	31	0.920	0.753	0.661	0.841	0.744	0.632	0.794	0.735
RF	32	0.912	0.755	0.666	0.827	0.744	0.631	0.790	0.736
RF	33	0.916	0.753	0.657	0.834	0.742	0.630	0.790	0.732
RF	34	0.920	0.753	0.660	0.840	0.744	0.630	0.793	0.734
RF	35	0.920	0.754	0.660	0.834	0.744	0.630	0.792	0.734
RF	36	0.916	0.760	0.664	0.839	0.748	0.639	0.795	0.738
RF	37	0.920	0.751	0.653	0.828	0.739	0.625	0.788	0.729
RF	38	0.916	0.762	0.667	0.833	0.749	0.641	0.795	0.740
RF	39	0.916	0.748	0.652	0.829	0.737	0.621	0.786	0.727
RF	40	0.920	0.750	0.653	0.834	0.739	0.625	0.789	0.729
RF	41	0.912	0.752	0.652	0.830	0.739	0.626	0.787	0.729
RF	42	0.924	0.757	0.661	0.837	0.746	0.635	0.795	0.736
RF	43	0.916	0.762	0.662	0.842	0.749	0.641	0.796	0.738
RF	44	0.912	0.759	0.669	0.852	0.750	0.640	0.798	0.741
RF	45	0.916	0.760	0.659	0.852	0.748	0.640	0.797	0.738
RF	46	0.916	0.752	0.646	0.844	0.739	0.628	0.790	0.728
RF	47	0.920	0.748	0.645	0.830	0.736	0.621	0.786	0.725
RF	48	0.924	0.759	0.656	0.839	0.746	0.638	0.795	0.735
RF	49	0.916	0.757	0.655	0.844	0.744	0.635	0.793	0.734
RF	50	0.908	0.749	0.643	0.836	0.736	0.622	0.784	0.724

RF	51	0.924	0.752	0.649	0.836	0.740	0.628	0.791	0.729
RF	52	0.916	0.752	0.636	0.836	0.736	0.625	0.785	0.723
RF	53	0.929	0.762	0.662	0.839	0.749	0.643	0.798	0.739
RF	54	0.916	0.744	0.640	0.833	0.732	0.614	0.783	0.721
RF	55	0.924	0.745	0.641	0.830	0.733	0.617	0.785	0.723
RF	56	0.916	0.765	0.658	0.827	0.747	0.640	0.792	0.737
RF	57	0.920	0.757	0.648	0.824	0.740	0.628	0.787	0.729
RF	58	0.912	0.748	0.636	0.839	0.733	0.620	0.784	0.721
RF	59	0.916	0.739	0.629	0.839	0.727	0.609	0.781	0.715
RF	60	0.904	0.748	0.632	0.834	0.731	0.619	0.779	0.718
RF	61	0.916	0.749	0.640	0.833	0.734	0.619	0.784	0.723
RF	62	0.924	0.743	0.635	0.843	0.732	0.617	0.786	0.720
RF	63	0.912	0.751	0.636	0.834	0.734	0.622	0.783	0.722
RF	64	0.912	0.741	0.631	0.834	0.728	0.610	0.779	0.716
RF	65	0.904	0.750	0.637	0.828	0.733	0.619	0.780	0.721
RF	66	0.912	0.751	0.647	0.823	0.736	0.622	0.783	0.726
RF	67	0.920	0.753	0.643	0.833	0.738	0.627	0.787	0.726
RF	68	0.916	0.750	0.648	0.834	0.738	0.624	0.787	0.727
RF	69	0.920	0.742	0.635	0.833	0.730	0.612	0.783	0.719
RF	70	0.912	0.755	0.651	0.847	0.742	0.632	0.791	0.731
RF	71	0.912	0.745	0.648	0.843	0.736	0.619	0.787	0.725
RF	72	0.912	0.749	0.634	0.830	0.732	0.618	0.781	0.720
RF	73	0.908	0.744	0.629	0.834	0.728	0.613	0.779	0.716
SVM	1	0.872	0.676	0.517	0.326	0.588	0.405	0.598	0.586
SVM	2	0.827	0.660	0.531	0.425	0.600	0.429	0.611	0.593

SVM	3	0.832	0.701	0.549	0.583	0.645	0.495	0.666	0.633
SVM	4	0.872	0.714	0.594	0.615	0.672	0.532	0.699	0.664
SVM	5	0.872	0.709	0.572	0.615	0.663	0.518	0.692	0.653
SVM	6	0.868	0.710	0.575	0.670	0.672	0.530	0.706	0.660
SVM	7	0.857	0.736	0.640	0.716	0.709	0.578	0.737	0.702
SVM	8	0.872	0.734	0.639	0.718	0.709	0.577	0.741	0.702
SVM	9	0.891	0.735	0.620	0.716	0.704	0.568	0.740	0.695
SVM	10	0.877	0.741	0.670	0.793	0.732	0.606	0.770	0.726
SVM	11	0.880	0.755	0.692	0.834	0.752	0.637	0.790	0.745
SVM	12	0.874	0.747	0.696	0.854	0.751	0.633	0.793	0.746
SVM	13	0.885	0.746	0.702	0.852	0.753	0.635	0.796	0.748
SVM	14	0.880	0.743	0.697	0.859	0.750	0.631	0.795	0.745
SVM	15	0.884	0.750	0.705	0.841	0.755	0.636	0.795	0.750
SVM	16	0.892	0.745	0.708	0.855	0.756	0.638	0.800	0.752
SVM	17	0.900	0.742	0.700	0.843	0.751	0.630	0.796	0.746
SVM	18	0.897	0.744	0.706	0.849	0.755	0.636	0.799	0.750
SVM	19	0.893	0.739	0.698	0.852	0.749	0.629	0.795	0.745
SVM	20	0.893	0.736	0.710	0.856	0.753	0.631	0.799	0.749
SVM	21	0.873	0.726	0.695	0.843	0.741	0.614	0.784	0.736
SVM	22	0.873	0.728	0.700	0.846	0.744	0.618	0.787	0.739
SVM	23	0.880	0.733	0.706	0.851	0.749	0.625	0.793	0.745
SVM	24	0.880	0.726	0.698	0.836	0.741	0.612	0.785	0.737
SVM	25	0.889	0.745	0.718	0.839	0.757	0.637	0.798	0.754
SVM	26	0.889	0.739	0.711	0.839	0.752	0.630	0.794	0.748
SVM	27	0.885	0.719	0.705	0.851	0.743	0.613	0.790	0.739

SVM	28	0.885	0.715	0.699	0.843	0.738	0.606	0.785	0.734
SVM	29	0.881	0.731	0.708	0.848	0.749	0.624	0.792	0.745
SVM	30	0.889	0.722	0.701	0.861	0.744	0.615	0.793	0.740
SVM	31	0.889	0.732	0.705	0.851	0.748	0.622	0.794	0.744
SVM	32	0.878	0.731	0.702	0.843	0.745	0.619	0.788	0.741
SVM	33	0.897	0.742	0.719	0.858	0.759	0.637	0.804	0.756
SVM	34	0.893	0.740	0.717	0.855	0.757	0.634	0.801	0.753
SVM	35	0.897	0.743	0.717	0.853	0.758	0.637	0.802	0.755
SVM	36	0.897	0.742	0.713	0.853	0.756	0.633	0.801	0.752
SVM	37	0.897	0.747	0.730	0.859	0.765	0.647	0.808	0.762
SVM	38	0.900	0.743	0.724	0.856	0.762	0.641	0.806	0.759
SVM	39	0.904	0.742	0.718	0.847	0.758	0.637	0.803	0.755
SVM	40	0.904	0.742	0.718	0.847	0.758	0.637	0.803	0.755
SVM	41	0.897	0.750	0.732	0.864	0.768	0.652	0.811	0.765
SVM	42	0.904	0.735	0.718	0.853	0.756	0.633	0.803	0.753
SVM	43	0.904	0.734	0.716	0.855	0.755	0.632	0.803	0.752
SVM	44	0.885	0.730	0.712	0.863	0.752	0.628	0.797	0.748
SVM	45	0.897	0.725	0.708	0.857	0.748	0.621	0.797	0.744
SVM	46	0.900	0.714	0.701	0.868	0.742	0.612	0.796	0.738
SVM	47	0.912	0.716	0.711	0.876	0.749	0.620	0.804	0.745
SVM	48	0.908	0.707	0.704	0.877	0.742	0.610	0.799	0.738
SVM	49	0.912	0.704	0.699	0.871	0.739	0.605	0.797	0.735
SVM	50	0.916	0.714	0.711	0.865	0.747	0.618	0.802	0.744
SVM	51	0.916	0.696	0.693	0.868	0.733	0.595	0.793	0.729
SVM	52	0.908	0.711	0.701	0.863	0.741	0.608	0.796	0.737

SVM	53	0.912	0.711	0.704	0.860	0.742	0.610	0.797	0.739
SVM	54	0.908	0.714	0.703	0.863	0.743	0.611	0.797	0.739
SVM	55	0.908	0.705	0.697	0.854	0.736	0.600	0.791	0.732
SVM	56	0.912	0.701	0.694	0.852	0.733	0.597	0.790	0.730
SVM	57	0.912	0.703	0.693	0.852	0.733	0.597	0.790	0.730
SVM	58	0.912	0.707	0.694	0.848	0.735	0.599	0.790	0.731
SVM	59	0.916	0.702	0.687	0.853	0.731	0.594	0.790	0.727
SVM	60	0.916	0.706	0.690	0.855	0.734	0.599	0.792	0.730
SVM	61	0.916	0.704	0.690	0.855	0.733	0.598	0.791	0.730
SVM	62	0.916	0.701	0.691	0.856	0.733	0.597	0.791	0.729
SVM	63	0.916	0.703	0.692	0.856	0.734	0.599	0.792	0.730
SVM	64	0.912	0.700	0.684	0.851	0.729	0.592	0.787	0.725
SVM	65	0.912	0.703	0.685	0.851	0.731	0.594	0.788	0.726
SVM	66	0.912	0.696	0.687	0.842	0.728	0.589	0.784	0.724
SVM	67	0.916	0.697	0.687	0.845	0.728	0.590	0.786	0.724
SVM	68	0.920	0.699	0.686	0.843	0.729	0.591	0.787	0.725
SVM	69	0.924	0.692	0.682	0.850	0.725	0.585	0.787	0.722
SVM	70	0.924	0.695	0.684	0.853	0.728	0.588	0.789	0.724
SVM	71	0.924	0.695	0.685	0.856	0.728	0.589	0.790	0.724
SVM	72	0.924	0.699	0.688	0.856	0.731	0.594	0.792	0.727
SVM	73	0.924	0.707	0.694	0.859	0.737	0.603	0.796	0.733

(4) IFS results on the mRMR feature list

Classification algorithms	Number of features	Unvaccinated healthcare workers	Healthcare workers within 60 days after vaccination	Healthcare workers between 60 and180 days after vaccination	Healthcare workers over 180 days after vaccination	ACC	MCC	Macro F1	Weighted F1
DT	1	0.797	0.484	0.480	0.331	0.483	0.259	0.523	0.490
DT	2	0.833	0.576	0.545	0.478	0.571	0.369	0.608	0.572
DT	3	0.860	0.586	0.565	0.568	0.597	0.400	0.645	0.596
DT	4	0.836	0.637	0.612	0.601	0.638	0.467	0.671	0.637
DT	5	0.910	0.646	0.625	0.643	0.658	0.491	0.706	0.657
DT	6	0.882	0.667	0.661	0.699	0.686	0.526	0.727	0.684
DT	7	0.880	0.645	0.641	0.707	0.670	0.503	0.718	0.667
DT	8	0.846	0.659	0.667	0.741	0.688	0.527	0.728	0.685
DT	9	0.876	0.686	0.675	0.742	0.704	0.552	0.745	0.701
DT	10	0.851	0.669	0.644	0.720	0.680	0.523	0.721	0.677
DT	11	0.798	0.671	0.668	0.747	0.690	0.537	0.721	0.687
DT	12	0.870	0.701	0.686	0.744	0.714	0.567	0.750	0.712
DT	13	0.869	0.673	0.685	0.802	0.709	0.561	0.757	0.706
DT	14	0.882	0.725	0.699	0.745	0.730	0.594	0.763	0.728
DT	15	0.850	0.663	0.656	0.714	0.682	0.521	0.721	0.679
DT	16	0.879	0.678	0.684	0.727	0.703	0.551	0.742	0.701
DT	17	0.839	0.689	0.668	0.729	0.698	0.545	0.732	0.696
DT	18	0.835	0.694	0.683	0.768	0.710	0.564	0.745	0.707
DT	19	0.815	0.689	0.699	0.767	0.713	0.567	0.743	0.711
DT	20	0.884	0.695	0.687	0.784	0.717	0.572	0.762	0.715
DT	21	0.869	0.679	0.686	0.741	0.704	0.551	0.744	0.703

DT	22	0.851	0.706	0.693	0.757	0.718	0.573	0.752	0.716
DT	23	0.882	0.686	0.689	0.750	0.710	0.558	0.752	0.709
DT	24	0.894	0.687	0.679	0.754	0.708	0.556	0.754	0.706
DT	25	0.886	0.691	0.690	0.770	0.715	0.565	0.759	0.714
DT	26	0.906	0.681	0.686	0.755	0.710	0.561	0.757	0.708
DT	27	0.872	0.676	0.666	0.769	0.699	0.546	0.746	0.696
DT	28	0.838	0.674	0.677	0.763	0.699	0.544	0.738	0.697
DT	29	0.878	0.682	0.687	0.758	0.709	0.558	0.751	0.707
DT	30	0.850	0.675	0.668	0.738	0.694	0.538	0.733	0.692
DT	31	0.874	0.656	0.661	0.782	0.691	0.533	0.743	0.688
DT	32	0.912	0.688	0.677	0.745	0.708	0.557	0.756	0.706
DT	33	0.888	0.687	0.685	0.781	0.713	0.564	0.760	0.711
DT	34	0.889	0.682	0.674	0.760	0.705	0.553	0.751	0.703
DT	35	0.856	0.666	0.665	0.764	0.693	0.534	0.738	0.690
DT	36	0.876	0.685	0.686	0.778	0.712	0.561	0.756	0.709
DT	37	0.861	0.625	0.639	0.747	0.664	0.491	0.718	0.661
DT	38	0.882	0.692	0.673	0.767	0.709	0.560	0.754	0.706
DT	39	0.887	0.688	0.656	0.708	0.693	0.539	0.735	0.691
DT	40	0.870	0.691	0.670	0.752	0.704	0.554	0.746	0.702
DT	41	0.872	0.647	0.668	0.784	0.690	0.532	0.743	0.687
DT	42	0.862	0.663	0.659	0.748	0.688	0.527	0.733	0.685
DT	43	0.866	0.678	0.670	0.718	0.695	0.538	0.733	0.693
DT	44	0.875	0.649	0.663	0.739	0.684	0.522	0.731	0.681
DT	45	0.868	0.656	0.652	0.740	0.682	0.520	0.729	0.679
DT	46	0.866	0.679	0.663	0.734	0.694	0.536	0.735	0.692

DT	47	0.903	0.681	0.682	0.780	0.712	0.564	0.762	0.708
DT	48	0.838	0.648	0.663	0.741	0.682	0.520	0.723	0.679
DT	49	0.860	0.647	0.646	0.767	0.678	0.512	0.730	0.675
DT	50	0.805	0.657	0.663	0.760	0.685	0.525	0.721	0.681
DT	51	0.860	0.678	0.646	0.718	0.685	0.525	0.725	0.682
DT	52	0.885	0.647	0.647	0.715	0.674	0.505	0.724	0.672
DT	53	0.852	0.672	0.650	0.715	0.683	0.523	0.722	0.681
DT	54	0.824	0.657	0.665	0.719	0.682	0.523	0.716	0.680
DT	55	0.863	0.651	0.661	0.782	0.689	0.532	0.740	0.685
DT	56	0.851	0.673	0.652	0.740	0.688	0.529	0.729	0.684
DT	57	0.848	0.670	0.658	0.730	0.687	0.528	0.727	0.684
DT	58	0.845	0.676	0.665	0.745	0.694	0.540	0.733	0.691
DT	59	0.852	0.629	0.626	0.733	0.658	0.484	0.710	0.655
DT	60	0.885	0.676	0.664	0.750	0.697	0.542	0.744	0.694
DT	61	0.847	0.665	0.668	0.747	0.691	0.533	0.732	0.689
DT	62	0.861	0.690	0.684	0.753	0.709	0.563	0.747	0.707
DT	63	0.819	0.674	0.665	0.707	0.687	0.529	0.716	0.685
DT	64	0.856	0.675	0.658	0.715	0.688	0.531	0.726	0.686
DT	65	0.826	0.651	0.639	0.693	0.666	0.495	0.702	0.663
DT	66	0.888	0.657	0.668	0.722	0.688	0.531	0.734	0.686
DT	67	0.867	0.691	0.680	0.744	0.707	0.558	0.745	0.705
DT	68	0.861	0.666	0.668	0.729	0.690	0.535	0.731	0.688
DT	69	0.876	0.660	0.675	0.763	0.696	0.540	0.743	0.693
DT	70	0.853	0.687	0.685	0.752	0.708	0.560	0.744	0.705
DT	71	0.860	0.677	0.664	0.719	0.692	0.536	0.730	0.690

DT	72	0.826	0.656	0.660	0.729	0.681	0.520	0.718	0.678
DT	73	0.860	0.659	0.645	0.723	0.677	0.513	0.722	0.675
KNN	1	0.769	0.454	0.462	0.318	0.461	0.234	0.501	0.467
KNN	2	0.848	0.600	0.555	0.587	0.602	0.423	0.648	0.598
KNN	3	0.873	0.615	0.607	0.686	0.643	0.471	0.696	0.639
KNN	4	0.907	0.683	0.654	0.732	0.696	0.549	0.744	0.692
KNN	5	0.895	0.693	0.676	0.780	0.714	0.572	0.761	0.710
KNN	6	0.895	0.699	0.673	0.788	0.716	0.575	0.764	0.712
KNN	7	0.900	0.709	0.676	0.798	0.723	0.588	0.771	0.719
KNN	8	0.891	0.694	0.658	0.806	0.710	0.568	0.762	0.705
KNN	9	0.900	0.674	0.655	0.815	0.703	0.555	0.761	0.698
KNN	10	0.899	0.699	0.662	0.804	0.714	0.573	0.766	0.709
KNN	11	0.911	0.679	0.640	0.799	0.698	0.550	0.757	0.692
KNN	12	0.907	0.681	0.648	0.788	0.700	0.552	0.756	0.695
KNN	13	0.892	0.697	0.673	0.807	0.717	0.577	0.767	0.713
KNN	14	0.904	0.708	0.675	0.780	0.720	0.583	0.767	0.716
KNN	15	0.916	0.698	0.668	0.799	0.716	0.574	0.770	0.712
KNN	16	0.928	0.714	0.688	0.817	0.733	0.600	0.787	0.730
KNN	17	0.920	0.712	0.673	0.830	0.728	0.594	0.784	0.723
KNN	18	0.908	0.719	0.685	0.817	0.733	0.602	0.782	0.729
KNN	19	0.920	0.721	0.689	0.849	0.739	0.608	0.794	0.735
KNN	20	0.920	0.717	0.689	0.862	0.739	0.608	0.797	0.735
KNN	21	0.920	0.714	0.685	0.851	0.736	0.603	0.793	0.731
KNN	22	0.912	0.714	0.680	0.844	0.733	0.600	0.788	0.728
KNN	23	0.912	0.717	0.686	0.833	0.735	0.603	0.787	0.730

KNN	24	0.929	0.723	0.691	0.844	0.741	0.612	0.797	0.737
KNN	25	0.924	0.719	0.682	0.833	0.735	0.604	0.790	0.730
KNN	26	0.937	0.714	0.683	0.845	0.735	0.601	0.795	0.731
KNN	27	0.924	0.710	0.675	0.829	0.728	0.591	0.785	0.723
KNN	28	0.904	0.715	0.683	0.846	0.733	0.601	0.787	0.729
KNN	29	0.924	0.702	0.676	0.835	0.726	0.589	0.785	0.722
KNN	30	0.920	0.713	0.688	0.846	0.736	0.603	0.792	0.731
KNN	31	0.924	0.715	0.682	0.829	0.733	0.599	0.788	0.728
KNN	32	0.912	0.707	0.691	0.834	0.733	0.599	0.786	0.729
KNN	33	0.908	0.704	0.691	0.843	0.732	0.599	0.786	0.728
KNN	34	0.916	0.691	0.659	0.832	0.714	0.574	0.775	0.709
KNN	35	0.912	0.696	0.669	0.839	0.720	0.581	0.779	0.715
KNN	36	0.929	0.697	0.671	0.825	0.721	0.581	0.781	0.717
KNN	37	0.888	0.704	0.675	0.812	0.721	0.584	0.770	0.716
KNN	38	0.897	0.702	0.674	0.832	0.723	0.588	0.776	0.718
KNN	39	0.900	0.714	0.688	0.817	0.732	0.600	0.780	0.727
KNN	40	0.900	0.721	0.686	0.801	0.732	0.601	0.777	0.728
KNN	41	0.904	0.725	0.691	0.814	0.738	0.610	0.784	0.733
KNN	42	0.908	0.727	0.686	0.821	0.738	0.610	0.786	0.733
KNN	43	0.916	0.709	0.675	0.819	0.726	0.591	0.780	0.721
KNN	44	0.912	0.716	0.682	0.813	0.731	0.598	0.781	0.726
KNN	45	0.892	0.695	0.670	0.794	0.714	0.574	0.763	0.709
KNN	46	0.900	0.718	0.674	0.805	0.727	0.595	0.774	0.722
KNN	47	0.912	0.712	0.673	0.794	0.724	0.590	0.773	0.719
KNN	48	0.916	0.699	0.662	0.824	0.717	0.577	0.775	0.712

KNN	49	0.912	0.693	0.665	0.838	0.718	0.579	0.777	0.713
KNN	50	0.892	0.695	0.672	0.816	0.717	0.575	0.769	0.712
KNN	51	0.898	0.700	0.671	0.818	0.719	0.578	0.772	0.715
KNN	52	0.908	0.712	0.665	0.820	0.723	0.588	0.776	0.718
KNN	53	0.896	0.699	0.665	0.824	0.717	0.579	0.771	0.712
KNN	54	0.924	0.711	0.671	0.830	0.727	0.591	0.784	0.722
KNN	55	0.916	0.703	0.665	0.827	0.721	0.584	0.778	0.716
KNN	56	0.924	0.707	0.664	0.826	0.723	0.586	0.780	0.717
KNN	57	0.920	0.696	0.670	0.829	0.720	0.581	0.779	0.716
KNN	58	0.920	0.708	0.672	0.806	0.723	0.588	0.776	0.718
KNN	59	0.937	0.712	0.674	0.804	0.726	0.590	0.782	0.722
KNN	60	0.920	0.702	0.672	0.813	0.722	0.585	0.777	0.717
KNN	61	0.916	0.685	0.662	0.806	0.710	0.568	0.767	0.705
KNN	62	0.908	0.692	0.662	0.791	0.711	0.570	0.763	0.706
KNN	63	0.904	0.698	0.671	0.814	0.719	0.580	0.772	0.714
KNN	64	0.916	0.695	0.665	0.817	0.716	0.575	0.773	0.711
KNN	65	0.912	0.690	0.663	0.813	0.713	0.572	0.770	0.708
KNN	66	0.908	0.698	0.666	0.804	0.716	0.577	0.769	0.711
KNN	67	0.893	0.690	0.654	0.804	0.707	0.565	0.760	0.701
KNN	68	0.916	0.688	0.659	0.816	0.712	0.570	0.770	0.706
KNN	69	0.929	0.699	0.662	0.814	0.717	0.577	0.776	0.713
KNN	70	0.912	0.694	0.665	0.801	0.714	0.573	0.768	0.709
KNN	71	0.916	0.687	0.665	0.812	0.712	0.570	0.770	0.707
KNN	72	0.916	0.683	0.658	0.807	0.707	0.561	0.766	0.702
KNN	73	0.924	0.694	0.669	0.799	0.716	0.575	0.772	0.712

RF	1	0.765	0.476	0.476	0.336	0.479	0.250	0.513	0.484
RF	2	0.826	0.628	0.539	0.527	0.599	0.420	0.630	0.594
RF	3	0.891	0.674	0.573	0.635	0.650	0.486	0.693	0.643
RF	4	0.877	0.706	0.634	0.676	0.689	0.544	0.723	0.685
RF	5	0.907	0.736	0.666	0.727	0.723	0.591	0.759	0.718
RF	6	0.907	0.729	0.659	0.764	0.722	0.590	0.765	0.716
RF	7	0.911	0.741	0.669	0.779	0.733	0.609	0.775	0.726
RF	8	0.916	0.747	0.676	0.793	0.740	0.620	0.783	0.734
RF	9	0.912	0.751	0.675	0.830	0.745	0.627	0.792	0.738
RF	10	0.900	0.754	0.685	0.811	0.747	0.630	0.787	0.741
RF	11	0.912	0.749	0.667	0.811	0.740	0.622	0.785	0.732
RF	12	0.916	0.753	0.663	0.812	0.741	0.624	0.786	0.732
RF	13	0.907	0.750	0.666	0.824	0.741	0.625	0.787	0.733
RF	14	0.904	0.751	0.663	0.817	0.740	0.624	0.784	0.731
RF	15	0.912	0.750	0.667	0.817	0.741	0.624	0.787	0.734
RF	16	0.912	0.757	0.665	0.810	0.743	0.628	0.786	0.734
RF	17	0.912	0.756	0.668	0.809	0.743	0.626	0.786	0.735
RF	18	0.900	0.755	0.659	0.809	0.739	0.623	0.781	0.730
RF	19	0.908	0.748	0.657	0.833	0.739	0.623	0.786	0.729
RF	20	0.912	0.751	0.650	0.817	0.736	0.620	0.783	0.726
RF	21	0.912	0.758	0.669	0.830	0.747	0.635	0.792	0.739
RF	22	0.897	0.754	0.667	0.817	0.742	0.626	0.784	0.734
RF	23	0.912	0.758	0.672	0.828	0.748	0.635	0.793	0.740
RF	24	0.908	0.751	0.657	0.822	0.739	0.622	0.784	0.729
RF	25	0.916	0.758	0.664	0.832	0.747	0.636	0.793	0.737

RF	26	0.908	0.765	0.677	0.836	0.754	0.646	0.797	0.745
RF	27	0.920	0.758	0.678	0.835	0.752	0.640	0.798	0.744
RF	28	0.912	0.747	0.659	0.838	0.740	0.625	0.789	0.731
RF	29	0.912	0.758	0.673	0.835	0.749	0.638	0.795	0.741
RF	30	0.912	0.755	0.671	0.835	0.747	0.635	0.793	0.739
RF	31	0.916	0.752	0.660	0.831	0.742	0.629	0.790	0.733
RF	32	0.916	0.748	0.651	0.836	0.738	0.623	0.788	0.728
RF	33	0.912	0.751	0.667	0.836	0.744	0.632	0.792	0.736
RF	34	0.916	0.750	0.654	0.840	0.740	0.626	0.790	0.730
RF	35	0.908	0.756	0.657	0.835	0.743	0.632	0.789	0.733
RF	36	0.912	0.756	0.653	0.840	0.743	0.634	0.790	0.732
RF	37	0.912	0.752	0.656	0.835	0.741	0.628	0.789	0.732
RF	38	0.908	0.747	0.640	0.836	0.733	0.620	0.783	0.722
RF	39	0.916	0.756	0.656	0.836	0.744	0.633	0.791	0.733
RF	40	0.916	0.752	0.652	0.841	0.741	0.629	0.790	0.730
RF	41	0.916	0.754	0.642	0.829	0.738	0.628	0.785	0.726
RF	42	0.908	0.758	0.658	0.845	0.746	0.637	0.792	0.735
RF	43	0.912	0.752	0.650	0.837	0.740	0.629	0.788	0.729
RF	44	0.912	0.751	0.648	0.840	0.739	0.625	0.788	0.728
RF	45	0.920	0.754	0.653	0.834	0.741	0.630	0.790	0.731
RF	46	0.912	0.757	0.659	0.845	0.746	0.637	0.793	0.735
RF	47	0.912	0.750	0.645	0.837	0.737	0.625	0.786	0.726
RF	48	0.908	0.742	0.634	0.832	0.729	0.613	0.779	0.717
RF	49	0.920	0.742	0.636	0.840	0.731	0.615	0.785	0.720
RF	50	0.929	0.759	0.649	0.830	0.743	0.635	0.792	0.731

RF	51	0.908	0.745	0.634	0.826	0.730	0.614	0.779	0.718
RF	52	0.912	0.746	0.641	0.841	0.734	0.618	0.785	0.723
RF	53	0.912	0.745	0.647	0.842	0.736	0.620	0.787	0.725
RF	54	0.912	0.739	0.624	0.836	0.725	0.607	0.778	0.712
RF	55	0.916	0.750	0.645	0.843	0.738	0.624	0.789	0.727
RF	56	0.912	0.756	0.649	0.841	0.741	0.631	0.790	0.730
RF	57	0.916	0.738	0.635	0.840	0.728	0.608	0.783	0.718
RF	58	0.920	0.739	0.632	0.817	0.725	0.605	0.777	0.714
RF	59	0.916	0.747	0.635	0.823	0.731	0.617	0.780	0.719
RF	60	0.920	0.750	0.640	0.836	0.736	0.622	0.787	0.724
RF	61	0.904	0.755	0.654	0.840	0.742	0.632	0.788	0.731
RF	62	0.916	0.741	0.637	0.838	0.731	0.614	0.783	0.719
RF	63	0.908	0.754	0.644	0.837	0.739	0.629	0.786	0.727
RF	64	0.908	0.748	0.642	0.847	0.736	0.622	0.786	0.724
RF	65	0.912	0.749	0.645	0.840	0.736	0.621	0.786	0.726
RF	66	0.912	0.750	0.645	0.851	0.739	0.626	0.790	0.727
RF	67	0.912	0.751	0.647	0.844	0.739	0.626	0.788	0.728
RF	68	0.904	0.750	0.647	0.844	0.738	0.625	0.786	0.727
RF	69	0.908	0.743	0.627	0.832	0.727	0.611	0.777	0.714
RF	70	0.904	0.744	0.633	0.830	0.729	0.613	0.778	0.717
RF	71	0.912	0.741	0.622	0.839	0.725	0.608	0.779	0.713
RF	72	0.908	0.755	0.643	0.836	0.739	0.629	0.786	0.726
RF	73	0.916	0.752	0.633	0.839	0.735	0.624	0.785	0.722
SVM	1	0.870	0.631	0.534	0.358	0.581	0.378	0.598	0.579
SVM	2	0.853	0.712	0.540	0.475	0.640	0.498	0.645	0.624

SVM	3	0.878	0.726	0.536	0.563	0.658	0.522	0.676	0.639
SVM	4	0.877	0.724	0.592	0.597	0.676	0.541	0.697	0.665
SVM	5	0.871	0.734	0.636	0.737	0.711	0.583	0.744	0.702
SVM	6	0.883	0.742	0.662	0.785	0.730	0.606	0.768	0.723
SVM	7	0.873	0.731	0.665	0.800	0.727	0.600	0.767	0.720
SVM	8	0.874	0.746	0.676	0.818	0.740	0.620	0.779	0.733
SVM	9	0.885	0.738	0.670	0.832	0.736	0.610	0.781	0.729
SVM	10	0.881	0.727	0.671	0.831	0.731	0.600	0.778	0.725
SVM	11	0.885	0.710	0.663	0.835	0.722	0.586	0.774	0.716
SVM	12	0.881	0.715	0.663	0.829	0.723	0.588	0.772	0.717
SVM	13	0.874	0.711	0.664	0.840	0.722	0.586	0.772	0.716
SVM	14	0.893	0.718	0.665	0.829	0.725	0.592	0.776	0.720
SVM	15	0.893	0.706	0.660	0.842	0.720	0.584	0.775	0.714
SVM	16	0.900	0.740	0.708	0.836	0.752	0.631	0.796	0.748
SVM	17	0.893	0.737	0.722	0.846	0.757	0.635	0.800	0.754
SVM	18	0.889	0.732	0.709	0.847	0.749	0.623	0.794	0.746
SVM	19	0.885	0.730	0.710	0.872	0.751	0.625	0.799	0.748
SVM	20	0.878	0.730	0.712	0.866	0.751	0.625	0.797	0.748
SVM	21	0.885	0.723	0.713	0.853	0.747	0.618	0.794	0.744
SVM	22	0.874	0.733	0.716	0.850	0.752	0.627	0.793	0.748
SVM	23	0.897	0.739	0.718	0.846	0.756	0.633	0.800	0.753
SVM	24	0.897	0.739	0.721	0.849	0.757	0.635	0.801	0.754
SVM	25	0.889	0.731	0.719	0.850	0.753	0.629	0.797	0.750
SVM	26	0.893	0.729	0.719	0.860	0.754	0.629	0.800	0.751
SVM	27	0.889	0.730	0.718	0.851	0.752	0.627	0.797	0.749

SVM	28	0.889	0.732	0.718	0.848	0.753	0.629	0.797	0.750
SVM	29	0.893	0.735	0.719	0.857	0.756	0.634	0.801	0.753
SVM	30	0.893	0.742	0.725	0.861	0.762	0.643	0.805	0.758
SVM	31	0.893	0.741	0.721	0.861	0.760	0.639	0.804	0.756
SVM	32	0.904	0.724	0.717	0.857	0.752	0.625	0.801	0.748
SVM	33	0.900	0.722	0.714	0.854	0.749	0.621	0.798	0.745
SVM	34	0.908	0.708	0.699	0.854	0.738	0.604	0.792	0.734
SVM	35	0.908	0.710	0.700	0.851	0.739	0.605	0.792	0.735
SVM	36	0.912	0.721	0.710	0.863	0.749	0.621	0.802	0.745
SVM	37	0.904	0.698	0.692	0.868	0.733	0.597	0.791	0.729
SVM	38	0.904	0.703	0.695	0.865	0.736	0.601	0.792	0.732
SVM	39	0.897	0.712	0.703	0.863	0.741	0.610	0.794	0.738
SVM	40	0.897	0.710	0.702	0.866	0.741	0.609	0.794	0.737
SVM	41	0.897	0.712	0.700	0.866	0.741	0.610	0.794	0.737
SVM	42	0.897	0.712	0.700	0.866	0.741	0.610	0.794	0.737
SVM	43	0.897	0.710	0.696	0.863	0.738	0.605	0.791	0.734
SVM	44	0.904	0.722	0.712	0.868	0.750	0.623	0.802	0.747
SVM	45	0.897	0.719	0.704	0.836	0.741	0.610	0.789	0.738
SVM	46	0.900	0.727	0.706	0.829	0.745	0.616	0.791	0.742
SVM	47	0.893	0.722	0.711	0.847	0.747	0.618	0.793	0.743
SVM	48	0.904	0.709	0.702	0.855	0.739	0.605	0.793	0.736
SVM	49	0.904	0.710	0.703	0.855	0.740	0.606	0.793	0.737
SVM	50	0.900	0.701	0.689	0.839	0.729	0.591	0.783	0.725
SVM	51	0.900	0.707	0.702	0.852	0.738	0.604	0.790	0.734
SVM	52	0.912	0.716	0.704	0.852	0.743	0.611	0.796	0.739

SVM	53	0.908	0.705	0.693	0.853	0.734	0.598	0.790	0.730
SVM	54	0.908	0.712	0.697	0.853	0.739	0.605	0.792	0.735
SVM	55	0.908	0.714	0.699	0.855	0.741	0.608	0.794	0.737
SVM	56	0.908	0.700	0.691	0.850	0.731	0.594	0.787	0.727
SVM	57	0.916	0.708	0.696	0.854	0.737	0.602	0.794	0.734
SVM	58	0.920	0.724	0.712	0.856	0.750	0.622	0.803	0.747
SVM	59	0.920	0.722	0.713	0.856	0.750	0.621	0.803	0.747
SVM	60	0.920	0.721	0.711	0.856	0.749	0.619	0.802	0.746
SVM	61	0.920	0.703	0.695	0.848	0.734	0.597	0.792	0.731
SVM	62	0.912	0.702	0.693	0.851	0.733	0.595	0.789	0.729
SVM	63	0.920	0.700	0.691	0.857	0.733	0.595	0.792	0.729
SVM	64	0.924	0.710	0.692	0.850	0.736	0.602	0.794	0.733
SVM	65	0.924	0.713	0.699	0.853	0.741	0.608	0.797	0.737
SVM	66	0.924	0.713	0.699	0.853	0.741	0.608	0.797	0.737
SVM	67	0.924	0.712	0.701	0.863	0.742	0.610	0.800	0.739
SVM	68	0.929	0.716	0.708	0.858	0.747	0.616	0.803	0.743
SVM	69	0.920	0.705	0.698	0.854	0.737	0.602	0.794	0.734
SVM	70	0.920	0.704	0.694	0.854	0.735	0.599	0.793	0.731
SVM	71	0.920	0.694	0.683	0.859	0.728	0.589	0.789	0.724
SVM	72	0.924	0.702	0.688	0.856	0.733	0.596	0.793	0.729
SVM	73	0.924	0.707	0.694	0.859	0.737	0.603	0.796	0.733
