# Supplementary material for: Machine Learning Classification of Time since BNT162b2 COVID-19 Vaccination Based on Array-Measured Antibody Activity
Source: Life (Basel). 2023 May 31;13(6):1304. doi: 10.3390/life13061304 (PMC10305362; doi:10.3390/life13061304)
Supplement: Supplementary file 1 [file life-13-01304-s001.zip › Table S4.pdf]

Table S4. Intersection results of the optimal feature subsets identified by LASSO, LightGBM, MCFS, and mRMR methods.

Features in one subset	Features in two subsets	Features in three subsets	Features in four subsets
MoIgA	SARS.CoV.2.Spike.RBD.His.HEK	SARS.CoV.2.S2	SARS.CoV.2.S1.mFcTag
a-HuIgM	HuIgG	hCoV.HKU1.NP	MERS.CoV.S1.RBD.367.606.rFcTag
a-MoIgA	a-HuIgG	hCoV.NL63.S1	SARS.CoV.2.Spike.RBD.His.Bac
Flu.H1N1.HA1	a-MoIgG	SARS.CoV.2.Spike.RBD.rFc	SARS.CoV.S1.HisTag
a-HuIgA	HuIgM	SARS.CoV.2.S1	SARS.CoV.2.S1.RBD.mFc
hCoV.HKU1.S1	Flu.B	SARS.CoV.2.S1.HisTag	SARS.CoV.2.S1+S2
HuIgG_0.03	MoIgG	SARS.CoV.S1.RBD.HisTag	
HuIgG_0.10	Flu.H3N2.HA1+HA2	hCoV.229E.S1	
a-HuIgG_0.10	HuIgA	Flu.H1N1.HA1+HA2	
a-HuIgG_0.30	MERS.CoV.NP		
HuIgM_0.10	SARS.CoV.S1.RBD.rFcTag		
a-MoIgG_0.03	a-HuIgG_0.03		
hCoV.NL63.S1_S2	Flu.B_Mal/.HA1		
HuIgM_0.03	hCoV.OC43.HE		
Flu.B_Phu/.HA1+HA2	HuIgM_0.30		
MoIgG_0.03	SARS.CoV.NP		
Flu.B_Mal/.HA1+HA2			
hCoV.OC43.NP			
a-MoIgA_0.03			
Flu.H3N2.HA1			
HuIgA_0.03			
a-MoIgG_0.10			
hCoV.HKU1.S1_S2			
MoIgG_0.30			
hCoV.HKU1.S1_AA1.760			
Flu.B_Phu/.HA1			
MERS.CoV.S1.ECD.1-1297.HisTag			
