# Supplementary material for: Machine Learning Classification of Time since BNT162b2 COVID-19 Vaccination Based on Array-Measured Antibody Activity
Source: Life (Basel). 2023 May 31;13(6):1304. doi: 10.3390/life13061304 (PMC10305362; doi:10.3390/life13061304)
Supplement: Supplementary file 1 [file life-13-01304-s001.zip › Table S5.pdf]

Table S5. Results of the intersection of top 20 features identified by LASSO, LightGBM, MCFS, and mRMR methods for each class.

(1) Unvaccinated healthcare workers

Features identified by one method	Features identified by two methods	Features identified by three methods	Features identified by four methods
HuIgA_0.30	hCoV.HKU1.NP	HuIgM_0.30	SARS.CoV.2.S1.RBD.mFc
MoIgG_0.03	SARS.CoV.NP	SARS.CoV.2.S1.mFcTag	SARS.CoV.S1.HisTag
a-HuIgM_0.03	hCoV.OC43.HE	SARS.CoV.2.Spike.RBD.rFc	a-HuIgG_0.03
MoIgA_0.10	MERS.CoV.S1.RBD.367.606.rFcTag	SARS.CoV.S1.RBD.HisTag	SARS.CoV.2.Spike.RBD.His.Bac
a-MoIgG_0.03	Flu.B_Mal/.HA1	SARS.CoV.2.S1.HisTag	SARS.CoV.2.S1+S2
Flu.H3N2.HA1+HA2	SARS.CoV.2.S1	hCoV.NL63.S1_S2	
hCoV.OC43.NP	a-HuIgG_0.10		
MERS.CoV.NP	HuIgG_0.03		
HuIgA_0.03	SARS.CoV.S1.RBD.rFcTag		
HuIgM_0.10			
Flu.H3N2.HA1			
a-MoIgA_0.03			
hCoV.HKU1.S1_S2			
hCoV.229E.S1			
hCoV.HKU1.S1_AA1.760			
Flu.H1N1.HA1+HA2			
MERS.CoV.S1.ECD.1-1297.HisTag			
hCoV.NL63.S1			
SARS.CoV.2.Spike.RBD.His.HEK			

SARS.CoV.2.S2			
HuIgG_0.10			
Flu.B_Phu/.HA1+HA2			
HuIgG_0.30			
a-MoIgA_0.30			

(2) Healthcare workers within 60 days after vaccination

**Features identified by one method**	**Features identified by two methods**	**Features identified by three methods**	**Features identified by four methods**
MoIgG_0.03	hCoV.NL63.S1_S2	MERS.CoV.S1.RBD.367.606.rFcTag	SARS.CoV.2.S1.mFcTag
MoIgA_0.10	hCoV.229E.S1	a-HuIgG_0.03	HuIgM_0.30
a-HuIgM_0.03	SARS.CoV.2.Spike.RBD.His.Bac	HuIgG_0.10	
a-MoIgG_0.03	SARS.CoV.NP	SARS.CoV.2.S2	
HuIgA_0.30	a-HuIgG_0.30	HuIgG_0.03	
SARS.CoV.S1.RBD.rFcTag	a-MoIgG_0.30	Flu.B_Phu/.HA1+HA2	
a-MoIgA_0.03	SARS.CoV.2.Spike.RBD.His.HEK	hCoV.HKU1.NP	
MoIgG_0.30	Flu.H3N2.HA1+HA2	a-HuIgG_0.10	
HuIgM_0.10	HuIgG_0.30		
HuIgM_0.03	SARS.CoV.2.S1		
Flu.B_Mal/.HA1	SARS.CoV.2.S1+S2		
hCoV.HKU1.S1_AA1.760	a-MoIgG_0.10		
Flu.H1N1.HA1+HA2			
SARS.CoV.2.Spike.RBD.rFc			
hCoV.OC43.HE			
SARS.CoV.S1.HisTag			

SARS.CoV.2.S1.RBD.mFc			
SARS.CoV.S1.RBD.HisTag			
Flu.B_Phu/.HA1			
hCoV.NL63.S1			
MERS.CoV.S1.ECD.1-1297.HisTag			
a-MoIgA_0.30			
SARS.CoV.2.S1.HisTag			
Flu.H1N1.HA1			

(3) Healthcare workers between 60 and 180 days after vaccination

**Features identified by one method**	**Features identified by two methods**	**Features identified by three methods**	**Features identified by four methods**
MoIgA_0.10	a-MoIgG_0.03	MERS.CoV.S1.RBD.367.606.rFcTag	SARS.CoV.2.S1.mFcTag
a-HuIgM_0.03	hCoV.NL63.S1_S2	SARS.CoV.2.S1+S2	HuIgM_0.30
HuIgA_0.30	SARS.CoV.NP	SARS.CoV.2.Spike.RBD.rFc	SARS.CoV.2.S1.RBD.mFc
hCoV.HKU1.NP	a-HuIgG_0.03	a-HuIgG_0.30	
a-MoIgG_0.30	hCoV.OC43.HE	HuIgG_0.10	
SARS.CoV.S1.RBD.rFcTag	SARS.CoV.S1.HisTag		
MERS.CoV.NP	Flu.B_Mal/.HA1		
MoIgG_0.30	MoIgG_0.03		
a-MoIgA_0.03	Flu.H3N2.HA1+HA2		
SARS.CoV.S1.RBD.HisTag	Flu.B_Phu/.HA1+HA2		
Flu.B_Phu/.HA1	HuIgG_0.30		

MERS.CoV.S1.ECD.1-1297.HisTag	SARS.CoV.2.S1.HisTag		
hCoV.NL63.S1	SARS.CoV.2.S1		
Flu.H1N1.HA1+HA2	SARS.CoV.2.Spike.RBD.His.Bac		
hCoV.229E.S1	HuIgG_0.03		
hCoV.HKU1.S1_AA1.760	HuIgM_0.10		
a-MoIgA_0.30	HuIgM_0.03		
a-HuIgG_0.10			
SARS.CoV.2.Spike.RBD.His.HEK			

(4) Healthcare workers over 180 days after vaccination

**Features identified by one method**	**Features identified by two methods**	**Features identified by three methods**	**Features identified by four methods**
a-HuIgM_0.03	Flu.B_Phu/.HA1+HA2	HuIgM_0.30	MERS.CoV.S1.RBD.367.606.rFcTag
HuIgA_0.30	SARS.CoV.2.S1.mFcTag	Flu.H1N1.HA1+HA2	Flu.B_Mal/.HA1
MoIgA_0.10	SARS.CoV.S1.HisTag	hCoV.229E.S1	a-HuIgG_0.03
a-MoIgG_0.30	SARS.CoV.2.S1+S2	hCoV.NL63.S1_S2	
Flu.H3N2.HA1+HA2	HuIgM_0.03	hCoV.HKU1.NP	
SARS.CoV.2.S2	HuIgM_0.10	MoIgG_0.03	
a-HuIgG_0.10	HuIgA_0.03	a-MoIgG_0.03	
a-MoIgG_0.10	a-MoIgA_0.03	HuIgG_0.03	
SARS.CoV.2.S1	MERS.CoV.NP		
SARS.CoV.2.S1.HisTag	a-HuIgG_0.30		
hCoV.NL63.S1	Flu.B_Mal/.HA1+HA2		

MERS.CoV.S1.ECD.1-1297.HisTag			
Flu.B_Phu/.HA1			
SARS.CoV.2.Spike.RBD.His.Bac			
SARS.CoV.NP			
SARS.CoV.S1.RBD.HisTag			
hCoV.OC43.HE			
hCoV.HKU1.S1_AA1.760			
SARS.CoV.2.Spike.RBD.rFc			
Flu.H1N1.HA1			
HuIgG_0.10			
HuIgG_0.30			
